# Supplementary material for: Amide C–N bonds activation by A new variant of bifunctional N-heterocyclic carbene
Source: Nat Commun. 2024 Jan 12;15:496. doi: 10.1038/s41467-024-44756-8 (PMC10786861; doi:10.1038/s41467-024-44756-8)
Supplement: Supplementary file 1 — Supplementary Information [file 41467_2024_44756_MOESM1_ESM.pdf]

Supplementary Information

Amide C-N Bonds Activation by A New Variant of Bifunctional N-Heterocyclic Carbene

Yuxing Cai,[1,2‡] Yuxin Zhao,[3‡] Kai Tang,[2] Hong Zhang,[2] Xueling Mo,[2] Jiean Chen,[2*] Yong Huang[3*]

[1] State Key Laboratory of Chemical Oncogenomics, Peking University Shenzhen Graduate School, Shenzhen 518055, China
[2] Pingshan Translational Medicine Center, Shenzhen Bay Laboratory, Shenzhen 518118, China
[3] Department of Chemistry, The Hong Kong University of Science and Technology, Clear Water Bay, Kowloon, Hong Kong SAR, China
Email: chenja@szbl.ac.cn; yonghuang@ust.hk

1. Supplementary Notes 2
2. Supplementary Methods 3
2.1 Synthesis of cyclic biaryl lactams 3
2.2 Synthesis of catalyst precursor 11
2.3 Conditions screening 13
2.4 Synthetic derivatization 18
2.5 Computational study 20
2.6 General experiment procedures 48
2.7 Characterization of products 51
2.8 X-ray crystal structure 86
3. Supplementary Figures 90
3.1 NMR spectra 90
3.2 HPLC datas 163
4. Supplementary References 227

1. Supplementary Notes

All solvents were distilled according to general practice prior to use. All reagents were purchased and used without further purification unless specified otherwise. Solvents for flash column chromatography were technical grade and distilled prior to use. Analytical thin-layer chromatography (TLC) was performed using Huanghai silica gel plates with HSGF 254. Visualization of the developed chromatogram was performed by UV absorbance (254 nm) and appropriate stains. Flash column chromatography was performed using Qingdao Haiyang Chemical HG/T2354-92 silica gel (200-300 mesh) with the indicated solvent system according to standard techniques. ^{1}H NMR, ^{13}C NMR and ^{19}F NMR data were recorded on Bruker 400 MHz (101 MHz for ^{13}C, 376 MHz for ^{19}F) nuclear resonance spectrometers unless otherwise specified, respectively. Chemical shifts (δ) in ppm are reported as quoted relative to the residual signals of chloroform (^{1}H 7.26 ppm and ^{13}C 77.16 ppm). Multiplicities are described as: s (singlet), d (doublet), t (triplet), q (quartet), m (multiplet); and coupling constants (*J*) are reported in Hertz (Hz). ^{13}C NMR spectra were recorded with total proton decoupling. HRMS (ESI) analysis was performed by The Analytical Instrumentation Center at Peking University, Shenzhen Graduate School, and (HRMS) data were reported with ion mass/charge (m/z) ratios as values in atomic mass units.

2. Supplementary Methods

2.1 Synthesis of cyclic biaryl lactams

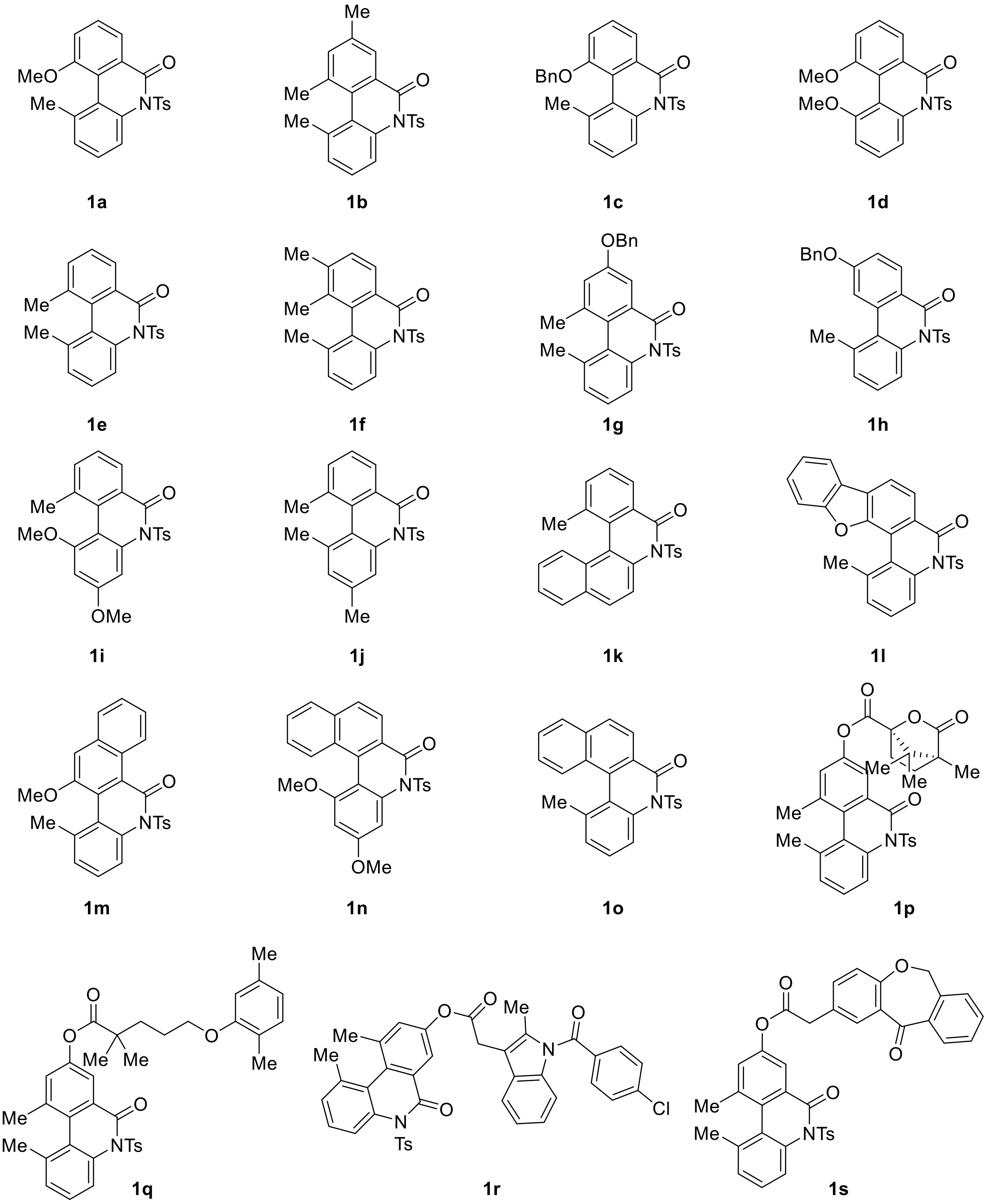

Supplementary Figure 1. Cyclic biaryl lactam **1** involved in the main text.

All alcohols and thiols were purchased from commercial sources. Cyclic biaryl lactams were prepared according to the literature, and their physical and spectroscopic properties agree with those reported.[1] New compounds were characterized as shown below.

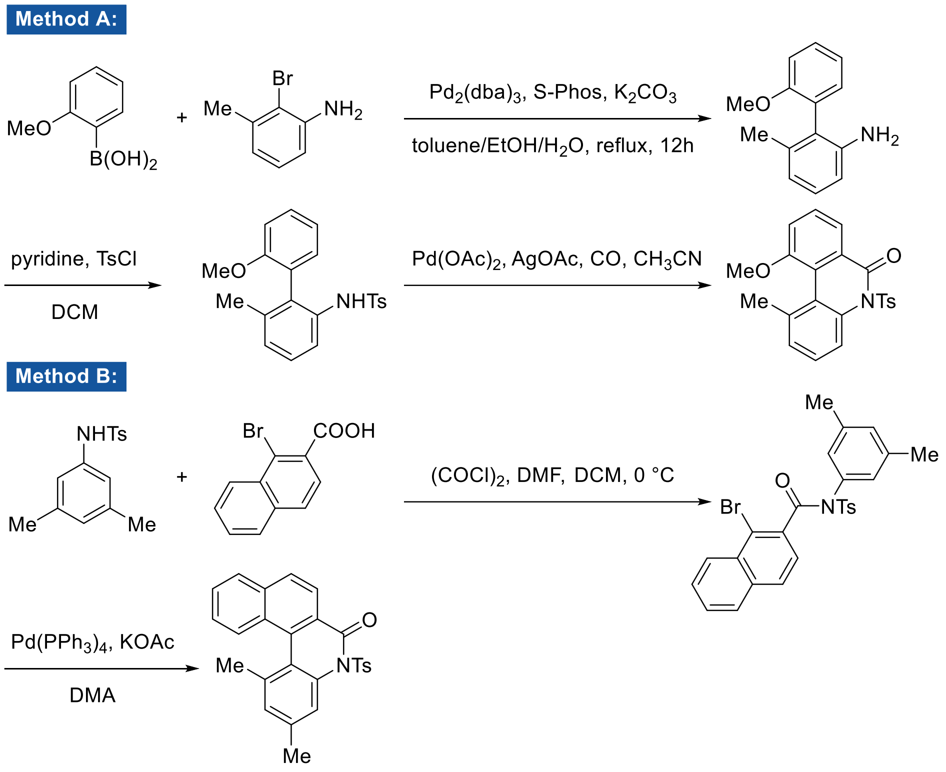

Method A:

An oven-dried round-bottom flask containing a magnetic stir bar was charged with the appropriate boronic acid (15.0 mmol, 1.5 eq.), $Pd_2(dba)_3$ (183 mg, 0.2 mmol, 2.0 mol%), S-Phos (328 mg, 0.8 mmol, 8.0 mol%) and anhydrous K_2CO_3 (5.5 g, 40.0 mmol, 4.0 eq.). The flask was capped with a rubber septum and then evacuated and backfilled with argon (this sequence was repeated three times). 2-Bromo anilines (10 mmol, 1.0 eq.) in toluene/EtOH/H_2O (27 mL/7 mL/7 mL, 8:2:2, 0.25 M) was added through the septum via syringe and the flask was sealed. The reaction was stirred at 90 °C for 12 h under argon atmosphere before being allowed to cool to room temperature. The mixture was extracted with ethyl acetate, and the combined organic layers were washed with H_2O and brine, dried over anhydrous Na_2SO_4, concentrated by rotary evaporation. The crude material was purified by flash chromatography on silica gel to give the product 2-Aminobiaryls.

To a flame-dried round bottom flask containing the solution of 2-Aminobiaryls (5.0 mmol, 1.0 eq.) in pyridine (10 mL, 0.5 M) was added arylsulfonyl chlorides (8.0 mmol,

1.6 eq.) at room temperature and the resulting mixture was refluxed at 120 °C for 2 h. After the completion of the reaction, the reaction mixture was allowed to cool to room temperature and extracted with CH_2Cl_2 for several times. The combined organic layer was washed with brine for two times and dried over anhydrous Na_2SO_4. The organic layer was then concentrated under vacuum, and the crude residue was purified by silica gel column chromatography to afford *N*-sulfonyl-2-aminobiaryls.

To a flame-dried round bottom flask containing *N*-sulfonyl-2-aminobiaryls (2.0 mmol), $Pd(OAc)_2$ (45 mg, 0.2 mmol, 10 mol%), AgOAc (1.6 g, 10.0 mmol, 5.0 eq.) and stir bar was sealed with septum and purged with CO gas. Acetonitrile (20 mL, 0.1 M) was injecteded into the flask and inserted a CO balloon to the reaction system and placed in oil bath at 80 °C with vigorous stirring for 24 h. Upon completion of the reaction, the reaction mixture was cooled to room temperature and diluted with ethyl acetate followed by filtration through a thin pad of celite. The filtrate was concentrated by vacuum and crude residue was purified by silica gel column chromatography to afford the desired cyclic biaryl lactam.

Method B:

To a solution of carboxylic acid (5.0 mmol, 1.0 eq.) in CH_2Cl_2 (10 mL, 0.5 M) were added oxalyl chloride (0.5 mL, 6.0 mmol, 1.2 eq.) and DMF (two drops) at 0 °C. The mixture was stirred until gas evolution stopped. Then it was concentrated under reduced pressure, washed with CH_2Cl_2 and concentrated again. The crude acyl chloride was used directly in the next step.

To a mixture of the corresponding sulfonamide (5.0 mmol, 1.0 eq.), DMAP (3.1 mg, 0.025 mmol, 0.5 mol%) and Et_3N (1.4 mL, 10.0 mmol, 2.0 eq.) in CH_2Cl_2 (10 mL) was added slowly the acyl chloride made above in CH_2Cl_2 (10 mL) at 0 °C. The reaction mixture was stirred at room temperature for 2 h, then washed with 5% HCl, brine and H_2O. The organic layer was dried over Na_2SO_4, filtered and evaporated under reduced pressure to give crude product, which was purified by column chromatography on silica gel to afford *N*-tosylcarboxylic amide.

An oven-dried flask with a stir bar was charged with $Pd(PPh_3)_4$ (144.5 mg, 0.125 mmol, 5 mol%), anhydrous KOAc (540.0 mg, 5.5 mmol, 2.2 eq.) and *N*-tosylcarboxylic amide

(2.5 mmol, 1.0 eq.). The flask was evacuated and backfilled with N_2. Anhydrous *N,N*-dimethylacetamide (12 mL, 0.2 M) was added via syringe, and the mixture stirred for 12 hours at 100 °C. The reaction mixture was diluted with H_2O and extracted with EtOAc. The combined organic layer was washed with brine and then dried over Na_2SO_4. The resulting mixture was filtered, concentrated under reduced pressure and purified by column chromatography on silica gel to afford the desired product.

10-(benzyloxy)-1-methyl-5-tosylphenanthridin-6(5H)-one (**1c**)

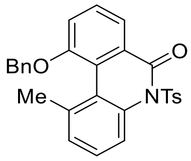

White solid, 2.1 g, 66% yield, **m.p.** 183-185 ℃. **^{1}H NMR** (400 MHz, $CDCl_3$) δ 8.22 – 8.14 (m, 2H), 7.83 (dd, *J* = 7.7, 1.2 Hz, 1H), 7.76 (d, *J* = 8.2 Hz, 1H), 7.49 (t, *J* = 7.9 Hz, 1H), 7.45 – 7.32 (m, 9H), 7.17 (d, *J* = 7.6 Hz, 1H), 5.17 (s, 2H), 2.47 (s, 3H), 2.34 (s, 3H). **^{13}C NMR** (101 MHz, $CDCl_3$) δ 163.1, 155.2, 145.0, 138.0, 137.0, 136.0, 133.5, 132.2, 129.7, 129.0, 128.7, 128.4, 128.2, 128.0, 127.8, 127.3, 124.6, 121.2, 120.7, 118.3, 116.9, 71.3, 23.2, 21.8. **HRMS** (ESI-TOF) $[M+H]^+$ calculated for $[C_{28}H_{24}NO_4S]^+$ 470.1421, found 470.1422.

8-(benzyloxy)-1,10-dimethyl-5-tosylphenanthridin-6(5H)-one (**1g**)

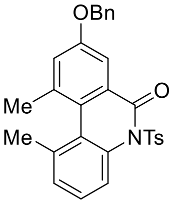

White solid, 4.8 g, 65% yield, **m.p.** 153-154 ℃. **^{1}H NMR** (400 MHz, $CDCl_3$) δ 8.15 (d, *J* = 8.4 Hz, 2H), 7.75 (d, *J* = 8.2 Hz, 1H), 7.61 (d, *J* = 2.8 Hz, 1H), 7.48 – 7.40 (m, 4H), 7.37 (dd, *J* = 7.6, 4.1 Hz, 3H), 7.31 (t, *J* = 8.0 Hz, 1H), 7.23 (d, *J* = 2.8 Hz, 1H), 7.18 (d, *J* = 7.6 Hz, 1H), 5.15 (q, *J* = 11.4 Hz, 2H), 2.45 (s, 3H), 2.40 (s, 3H), 2.33 (s, 3H). **^{13}C NMR** (101 MHz, $CDCl_3$) δ 163.5, 158.0, 145.0, 138.0, 137.0, 136.3, 136.0, 133.3, 132.5, 129.8, 128.7, 128.4, 128.3, 128.0, 128.0, 127.7, 127.3, 123.8, 123.3, 117.3, 109.4, 70.4, 21.7, 21.4. **HRMS** (ESI-TOF) $[M+Na]^+$ calculated for $[C_{29}H_{25}NNaO_4S]^+$ 506.1397, found 506.1397.

1,3-dimethoxy-10-methyl-5-tosylphenanthridin-6(5H)-one (**1i**)

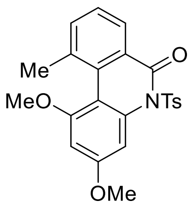

White solid, 430 mg, 24% yield, **m.p.** 152-153 °C. **^{1}H NMR** (400 MHz, $CDCl_3$) δ 8.13 (d, J = 8.4 Hz, 2H), 7.94 (dd, J = 7.6, 1.4 Hz, 1H), 7.55 – 7.51 (m, 1H), 7.37 – 7.30 (m, 3H), 7.10 (d, J = 2.3 Hz, 1H), 6.44 (d, J = 2.3 Hz, 1H), 3.89 (s, 3H), 3.85 (s, 3H), 2.45 (s, 3H), 2.36 (s, 3H). **^{13}C NMR** (101 MHz, $CDCl_3$) δ 163.7, 160.4, 157.4, 145.1, 137.0, 136.9, 136.2, 135.5, 132.4, 129.7, 129.7, 128.5, 126.5, 125.5, 107.1, 97.3, 95.7, 55.7, 55.3, 22.4, 21.8. **HRMS** (ESI-TOF) $[M+Na]^+$ calculated for $[C_{23}H_{21}NNaO_5S]^+$ 446.1033, found 446.1036.

1-methyl-5-tosylbenzofuro[3,2-k]phenanthridin-6(5H)-one (**1l**)

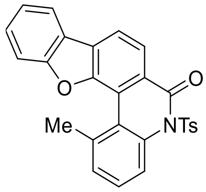

Yellow solid, 1.8 g, 57% yield, **m.p**. 207-208 °C. **^{1}H NMR** (400 MHz, $CDCl_3$) δ 8.24 (d, J = 8.1 Hz, 1H), 8.17 – 8.07 (m, 4H), 7.90 (d, J = 8.3 Hz, 1H), 7.73 (d, J = 8.3 Hz, 1H), 7.63 (t, J = 7.7 Hz, 1H), 7.49 (td, J = 7.8, 2.0 Hz, 2H), 7.42 – 7.32 (m, 3H), 2.59 (s, 3H), 2.45 (s, 3H). **^{13}C NMR** (101 MHz, $CDCl_3$) δ 162.9, 156.7, 150.7, 145.1, 137.8, 136.7, 134.2, 129.7, 129.5, 129.0, 128.9, 128.8, 128.7, 128.5, 123.7, 123.2, 123.1, 121.5, 120.6, 120.3, 119.8, 117.6, 112.0, 23.4, 21.7. **HRMS** (ESI-TOF) $[M+H]^+$ calculated for $[C_{27}H_{20}NO_4S]^+$ 454.1201, found 454.1109.

11-methoxy-10-methyl-6-tosylbenzo[i]phenanthridin-5(6H)-one (**1m**)

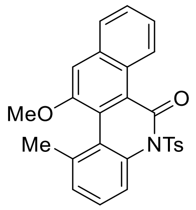

Yellow solid, 1.5 g, 39% yield, **m.p.** 181-183 °C. **^{1}H NMR** (400 MHz, $CDCl_3$) δ 9.40 – 9.34 (m, 1H), 8.18 (d, J = 8.4 Hz, 2H), 7.85 – 7.81 (m, 1H), 7.76 (d, J = 8.3 Hz, 1H), 7.57 – 7.53 (m, 2H), 7.50 (s, 1H), 7.34 (dd, J = 8.2, 6.7 Hz, 3H), 7.18 (d, J = 7.7 Hz, 1H), 4.00 (s, 3H), 2.43 (s, 3H), 2.34 (s, 3H). **^{13}C NMR** (101 MHz, $CDCl_3$) δ 163.7,

152.8, 144.9, 138.1, 137.1, 134.0, 133.1, 129.7, 129.3, 128.4, 128.3, 127.3, 127.2, 126.9, 126.8, 126.2, 126.1, 126.0, 120.7, 115.9, 111.9, 55.3, 22.4, 21.7. **HRMS** (ESI-TOF) $[M+Na]^+$ calculated for $[C_{26}H_{21}NNaO_4S]^+$ 466.1083, found 466.1084.

1,10-dimethyl-6-oxo-5-tosyl-5,6-dihydrophenanthridin-8-yl (1S)-4,7,7-trimethyl-3-oxo-2-oxabicy-clo[2.2.1]heptane-1-carboxylate (**1p**)

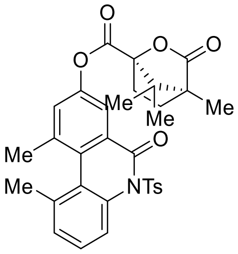

White solid, 0.29 g, 44% yield, **m.p.** 186-188 ℃. **^{1}H NMR** (400 MHz, CD_2Cl_2) δ 8.10 – 8.01 (m, 2H), 7.77 – 7.66 (m, 2H), 7.43 – 7.32 (m, 4H), 7.26 – 7.20 (m, 1H), 2.59 (dddd, *J* = 13.5, 10.8, 4.2, 2.7 Hz, 1H), 2.45 (s, 6H), 2.34 (s, 3H), 2.20 (dddd, *J* = 13.6, 9.3, 4.5, 1.4 Hz, 1H), 2.07 – 1.99 (m, 1H), 1.76 (ddd, *J* = 13.4, 9.4, 4.2 Hz, 1H), 1.17 (s, 3H), 1.15 (s, 3H), 1.10 (d, *J* = 1.1 Hz, 3H). **^{13}C NMR** (101 MHz, CD_2Cl_2) δ 177.6, 165.9, 162.7, 149.0, 145.6, 138.6, 136.8, 136.6, 133.8, 132.7, 132.5, 129.8, 128.5, 128.3, 128.2, 128.1, 122.7, 118.0, 117.9, 117.5, 90.8, 54.9, 54.8, 30.9, 29.0, 21.5, 21.5, 21.3, 16.8, 16.7, 9.5. **HRMS** (ESI-TOF) $[M+Na]^+$ calculated for $[C_{32}H_{31}NNaO_7S]^+$ 596.1713, found 596.1713.

1,10-dimethyl-6-oxo-5-tosyl-5,6-dihydrophenanthridin-8-yl 5-(2,5-dimethylphenoxy)-2,2-dimethyl pentanoate (**1q**)

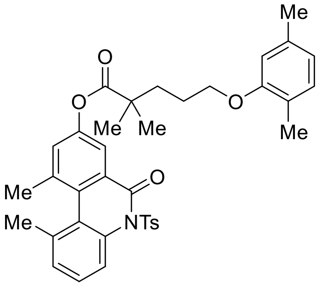

White solid, 0.41 g, 57% yield, **m.p.** 70-72 ℃. **^{1}H NMR** (400 MHz, $CDCl_3$) δ 8.16 (d, *J* = 8.1 Hz, 2H), 7.79 (d, *J* = 8.3 Hz, 1H), 7.75 (d, *J* = 2.5 Hz, 1H), 7.37 (t, *J* = 8.1 Hz, 3H), 7.32 (d, *J* = 2.6 Hz, 1H), 7.22 (d, *J* = 7.6 Hz, 1H), 7.04 (d, *J* = 7.4 Hz, 1H), 6.74 – 6.64 (m, 2H), 4.04 (d, *J* = 4.8 Hz, 2H), 2.44 (d, *J* = 10.6 Hz, 6H), 2.35 (s, 6H), 2.23 (s, 3H), 1.94 (s, 4H), 1.43 (s, 6H). **^{13}C NMR** (101 MHz, $CDCl_3$) δ 175.9, 162.8, 156.9,

150.0, 145.1, 137.8, 136.7, 136.5, 136.4, 133.7, 132.3, 131.9, 130.3, 129.7, 128.8, 128.4, 128.0, 127.9, 123.6, 122.7, 120.8, 118.4, 117.4, 111.9, 67.7, 42.5, 37.1, 25.3, 25.2, 25.1, 21.7, 21.6, 21.4, 21.3, 15.8. **HRMS** (ESI-TOF) $[M+Na]^+$ calculated for $[C_{37}H_{39}NNaO_6S]^+$ 648.2390, found 648.2396.

1,10-dimethyl-6-oxo-5-tosyl-5,6-dihydrophenanthridin-8-yl 2-(1-(4-chlorobenzoyl)-5-methoxy-2-methyl-1H-indol-3-yl)acetate (**1r**)

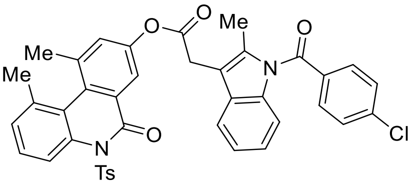

White solid, 0.3 g, 24% yield, **m.p.** 184-185 °C. **^{1}H NMR** (400 MHz, $CDCl_3$) δ 8.13 (d, *J* = 8.0 Hz, 2H), 7.71 (d, *J* = 9.5 Hz, 4H), 7.50 (d, *J* = 8.1 Hz, 2H), 7.35 (d, *J* = 7.2 Hz, 4H), 7.19 (d, *J* = 7.7 Hz, 1H), 7.07 (s, 1H), 6.93 (d, *J* = 9.0 Hz, 1H), 6.72 (d, *J* = 9.0 Hz, 1H), 3.95 (s, 2H), 3.87 (s, 3H), 2.49 (s, 3H), 2.43 (s, 3H), 2.40 (s, 3H), 2.31 (s, 3H). **^{13}C NMR** (101 MHz, $CDCl_3$) δ 168.8, 168.3, 162.7, 156.2, 149.6, 145.2, 139.4, 137.9, 136.6, 136.4, 136.3, 133.8, 133.7, 132.3, 132.1, 131.2, 130.8, 130.4, 129.7, 129.2, 128.6, 128.4, 128.0, 122.6, 118.2, 117.3, 115.1, 111.9, 111.6, 101.1, 55.8, 30.5, 21.7, 21.6, 21.3, 13.5. **HRMS** (ESI-TOF) $[M+Na]^+$ calculated for $[C_{41}H_{33}ClN_2NaO_7S]^+$ 755.1589, found 755.1590.

1,10-dimethyl-6-oxo-5-tosyl-5,6-dihydrophenanthridin-8-yl 2-(11-oxo-6,11-dihydrodibenzo[b,e] oxepin-2-yl)acetate (**1s**)

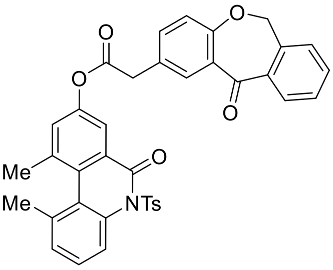

White solid, 0.17 g, 35% yield, **m.p.** 178-180 °C. **^{1}H NMR** (400 MHz, CD_2Cl_2) δ 8.21 (d, *J* = 2.4 Hz, 1H), 8.06 – 8.01 (m, 2H), 7.89 (dd, *J* = 7.6, 1.4 Hz, 1H), 7.69 (d, *J* = 8.3 Hz, 1H), 7.66 (d, *J* = 2.5 Hz, 1H), 7.60 (td, *J* = 7.5, 1.4 Hz, 1H), 7.54 (dd, *J* = 8.4, 2.4 Hz, 1H), 7.50 (td, *J* = 7.6, 1.3 Hz, 1H), 7.43 – 7.39 (m, 1H), 7.38 – 7.32 (m, 4H), 7.22 (d, *J* = 7.7 Hz, 1H), 7.10 (d, *J* = 8.4 Hz, 1H), 5.23 (s, 2H), 3.94 (s, 2H), 2.43 (s, 3H), 2.41 (s, 3H), 2.31 (s, 3H). **^{13}C NMR** (101 MHz, CD_2Cl_2) δ 190.5, 169.6, 162.8, 160.8,

149.8, 145.5, 140.4, 138.3, 136.7, 136.6, 136.4, 135.9, 133.7, 132.9, 132.6, 132.4, 132.2, 129.8, 129.4, 129.3, 128.7, 128.2, 128.1, 128.0, 127.9, 127.1, 125.4, 122.8, 121.3, 118.1, 117.5, 73.8, 40.1, 21.5, 21.5, 21.2. **HRMS** (ESI-TOF) $[M+Na]^+$ calculated for $[C_{38}H_{29}NNaO_7S]^+$ 666.1557, found 666.1559.

2.2 Synthesis of catalyst precursor

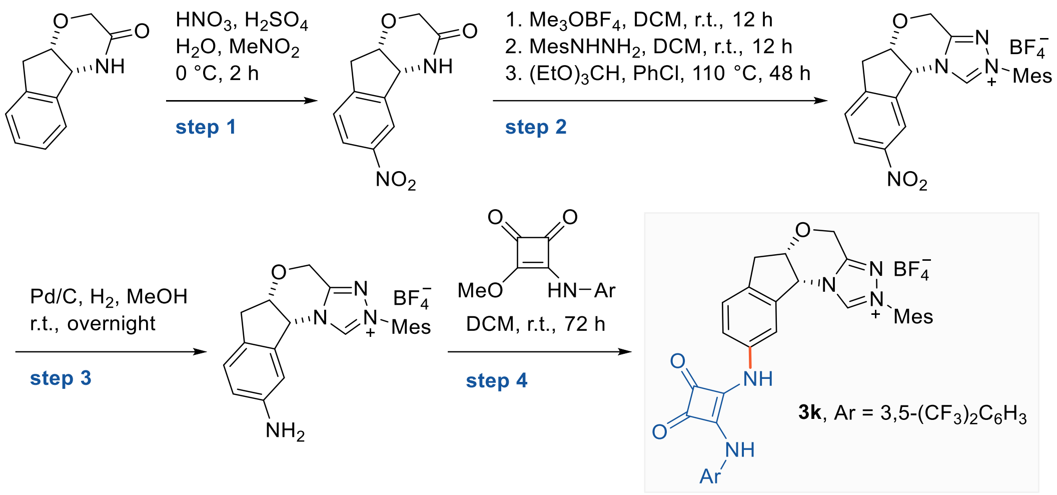

Step 1: To a cooled (-10 °C) solution of HNO_3 (1.6 mL, 33.0 mmol, 1.1 eq.) and distilled water (11 mL), H_2SO_4 (40 mL) was added, which was slowly introduced into a cooled (-10 °C) solution of (4a*R*,9a*S*)-4,4a,9,9a-tetrahydroindeno[2,1-*b*][1,4]oxazin-3(2H)-one (5.7 g, 30.0 mmol, 1.0 eq.) in nitromethane (56 mL) in 1 h. The mixture was stirred for 2 hours at -10 °C and poured into ice-water (1000 mL). The result suspension was stirred for another 1 h, filtered, washed with distilled water and cooled ethyl acetate to afford the nitro compound (4.9 g, 21.0 mmol, 70%).

Step 2: To a solution of nitro compounds (2.3 g, 10.0 mmol, 1.0 eq.) from **step 1** in CH_2Cl_2 (100 mL) was added Me_3OBF_4 (1.63 g, 11.0 mmol, 1.1 eq.), and the mixture was stirred at room temperature for 12 h. Then, $MesNHNH_2$ (1.5 g, 10.0 mmol, 1.0 eq.) was added, and the mixture was stirred at room temperature for another 24 h. The mixture was concentrated in vacuo, and the residue was dissolved in PhCl (100 mL), followed by the addition of $(EtO)_3CH$ (16 mL, 100 mmol). The mixture was heated at 110 °C for 48 h, evaporated under reduced vacuum and purified by flash column chromatography (PE:EA = 1:1 to EA) to afford the corresponding product (2.3 g, 5.0 mmol, 50%).

Step 3: To a solution of the intermediate (928.4 mg, 2.0 mmol, 1.0 eq.) from **step 2** in MeOH (100 mL) was added Pd/C (0.046 mmol, 2.3 mmol%), and the mixture was stirred at room temperature overnight with a H_2 balloon. After the reaction was completed, the mixture was filtered through celite and washed with MeOH. The combined filtrates were evaporated under a reduced vacuum and then purified by flash

column chromatography (PE:EA = 1:1 to DCM:MeOH = 20:1) to afford the amine product (782.0 mg, 1.8 mmol, 90%).

Step 4: To a solution of amine (868.4 mg, 2.0 mmol, 1.0 eq.) from **step 3** in DCM (12 mL) was added 3-((3,5-bis(trifluoromethyl)phenyl)amino)-4-methoxycyclobut-3-ene-1,2-dione (1.3 g, 4.0 mmol, 2.0 eq.). The mixture was stirred at room temperature for 72 h. After the reaction was completed, the mixture was evaporated under a reduced vacuum and then purified by flash column chromatography (PE:EA = 1:1 to DCM:MeOH = 20:1) to afford the NHC precursor **3k** (1.2 g, 1.6 mmol, 80%).

2.3 Conditions screening

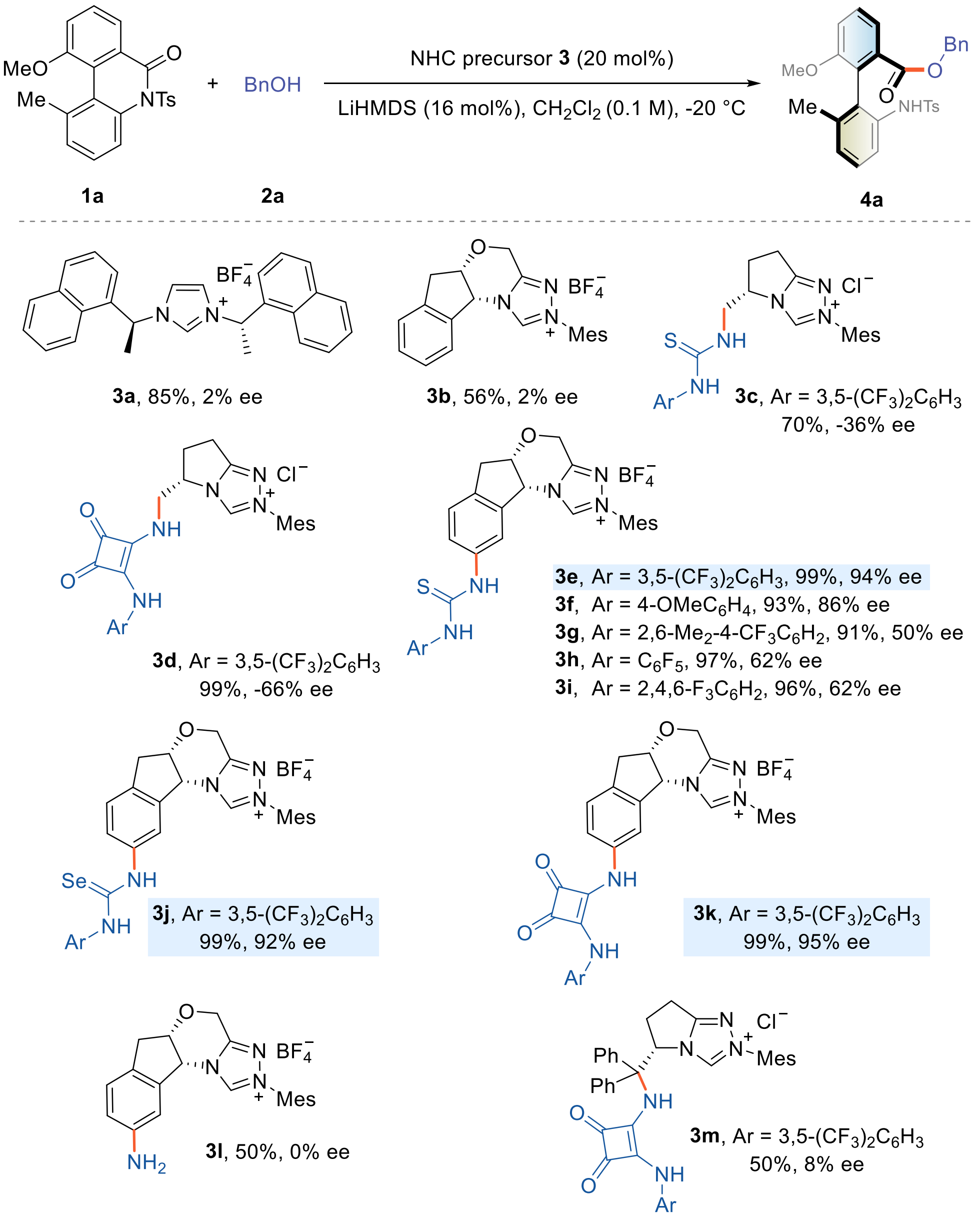

Supplementary Figure 2. Screening of NHC precursors. Reaction conditions: cyclic biaryl lactam **1a** (0.05 mmol), benzyl alcohol **2a** (0.1 mmol), NHC precursor **3** (20 mol%), and LiHMDS (16 mol%) were stirred in DCM (0.5 mL) at -20 °C under argon for 15 h. Yields were determined by NMR using 1,3,5-trimethoxybenzene as an internal standard. The enantiomeric excess (ee) was determined by chiral HPLC.

Supplementary Table 1. Screening of solvents[a] [b]

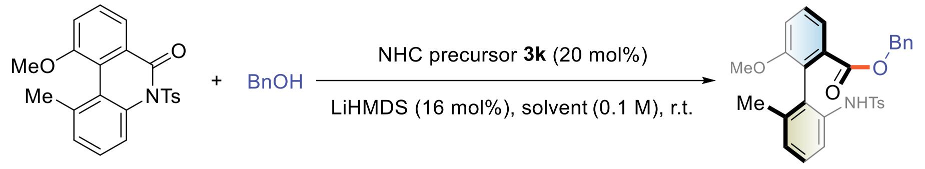

1a **2a** **4a**

entry	solvent	yield (%)	ee (%)
1	*n*-hexane	99	66
2	toluene	94	62
3	MTBE	99	75
4	Et_2O	87	76
5	1,4-dioxane	58	68
6	DMSO	94	76
7	CH_2Cl_2	88	78
8	DCE	98	74
9	$CDCl_3$	99	76
10	CCl_4	99	70

[a] Reaction conditions: cyclic biaryl lactam **1a** (0.05 mmol), benzyl alcohol **2a** (0.1 mmol), NHC precursor **3k** (20 mol%), and LiHMDS (16 mol%) were stirred in solvent (0.5 mL) at r.t. under argon for 15 h. [b] Yields were determined by NMR using 1,3,5-trimethoxybenzene as an internal standard. The enantiomeric excess (ee) was determined by chiral HPLC.

Supplementary Table 2. Screening of bases[a] [b]

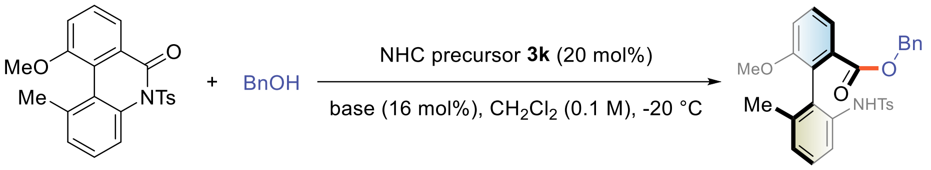

1a 2a 4a

entry	base	yield (%)	ee (%)
1	K_2CO_3	89	84
2	K_3PO_4	99	74
3	LiHMDS	99	95
4	^{n}BuLi	99	93
5	NEt_3	99	90
6	DIPEA	99	92
7	DBU	93	94

[a] Reaction conditions: cyclic biaryl lactam **1a** (0.05 mmol), benzyl alcohol **2a** (0.1 mmol), NHC precursor **3k** (20 mol%), and base (16 mol%) were stirred in DCM (0.5 mL) at -20 °C under argon for 15 h. [b] Yields were determined by NMR using 1,3,5-trimethoxybenzene as an internal standard. The enantiomeric excess (ee) was determined by chiral HPLC.

Supplementary Table 3. Screening of temperatures for the thiol substrate[a] [b]

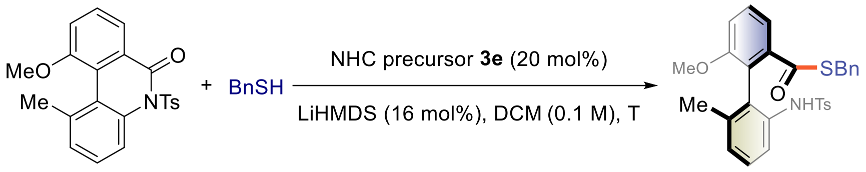

1a 6a

entry	T (ºC)	yield (%)	ee (%)
1	0	99	42
2	-20	98	88
3	-40	99	83
4	-60	99	91
5	-78	97	84
6[c]	-60	99	96

[a] Reaction conditions: cyclic biaryl lactam **1a** (0.05 mmol), benzyl mercaptan (0.1 mmol), NHC precursor **3e** (20 mol%), and LiHMDS (16 mol%) were stirred in DCM (0.5 mL) at T °C under argon for 15 h. [b] Yields were determined by NMR using 1,3,5-trimethoxybenzene as an internal standard. The enantiomeric excess (ee) was determined by chiral HPLC. [c] **3k** was used as the catalyst.

Supplementary Table 4. Conditions screening for *N*-nucleophiles

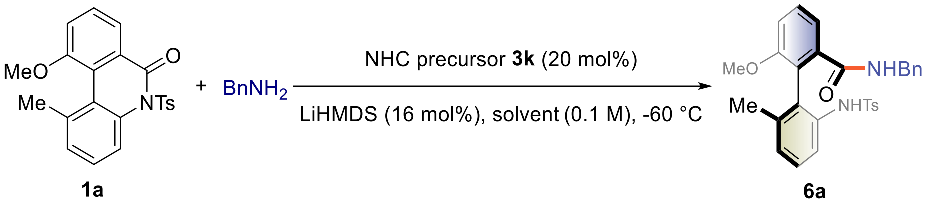

entry	solvent	yield (%)	ee (%)
1	toluene	90	0
2	CH_2Cl_2	99	6
3	Et_2O	99	-18
4	THF	99	4

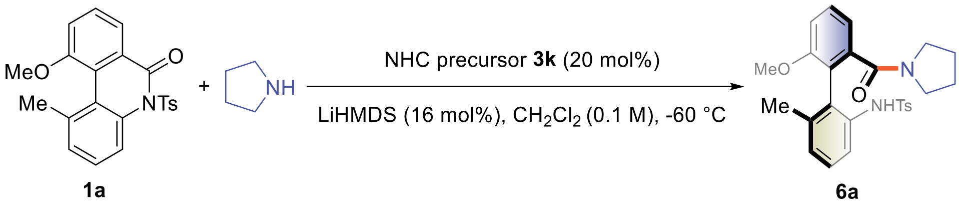

entry	additive	yield (%)	ee (%)
1	-	99	2
2	H_2O (0.5 eq)	57	-14
3	PhCOOH (0.1 eq)	55	-2

2.4 Synthetic derivatization

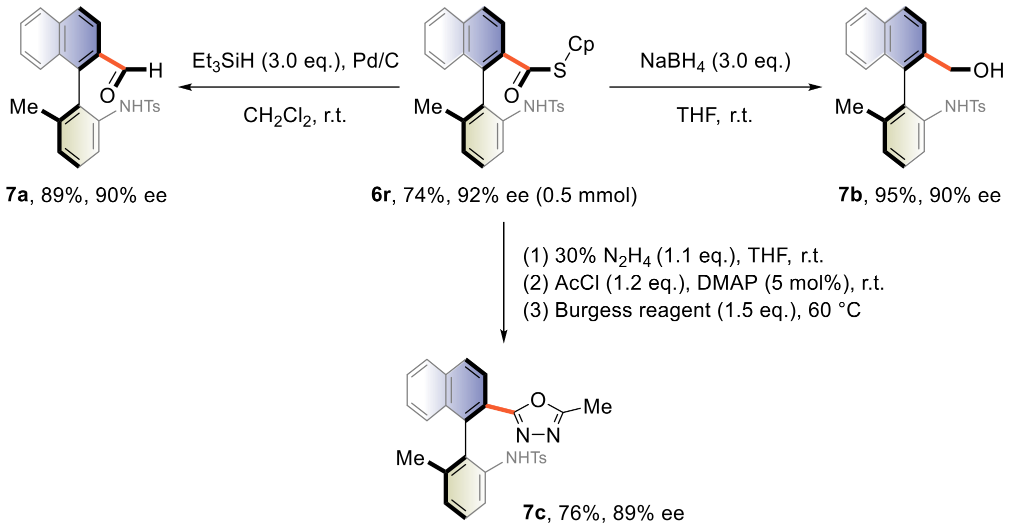

Supplementary Figure 3. Synthetic applications

For **7a**: The compound **6r** (51.5 mg, 0.1 mmol, 1.0 eq.) and Pd/C (0.01 mmol, 0.1 eq.) were added to a reaction tube. The mixture was degassed and back-filled with nitrogen (3x). Then CH_2Cl_2 (0.5 mL, 0.2 M) was added followed by dropwise addition of Et_3SiH (48 uL, 0.3 mmol, 3.0 eq.). The resulting mixture was stirred at room temperature for 12 hours and then filtered through a thin pad of celite. The filtrate was was purified by column chromatography on silica gel to afford compound **7a** as a white solid (36.9 mg, 0.89 mmol, 89% yield, 90% ee).

For **7b**: The compound **6r** (51.5 mg, 0.1 mmol, 1.0 eq.) and anhydrous THF (0.5 mL, 0.2 M) were added to a reaction tube. The mixture was degassed and back-filled with nitrogen (3x). Then $NaBH_4$ (0.3 mmol, 3.0 eq.) was added. The resulting mixture was stirred at room temperature for 12 hours and then quenched with saturated aqueous solution of NH_4Cl, extracted with EtOAc. The combined organic layer was washed with brine and then dried over Na_2SO_4. The resulting mixture was purified by column chromatography on silica gel to afford compound **7b** as a white solid (39.6 mg, 0.95 mmol, 95% yield, 90% ee).

For **7c**: The compound **6r** (51.5 mg, 0.1 mmol, 1.0 eq.) and anhydrous THF (0.5 mL, 0.2 M) were added to a reaction tube. The mixture was degassed and back-filled with nitrogen (3x). Then 30% N_2H_4 (0.11 mmol, 1.1 eq.) was added, and the resulting

mixture was stirred at room temperature for 12 hours. Upon completion, acetyl chloride (9 uL, 0.12 mmol, 1.2 eq.) and DMAP (0.6 mg, 0.005 mmol, 5.0 mol%) were added, and the mixture was stirred at room temperature for 12 hours. Then the reaction tube was taken into glovebox and Burgess reagent (35.7 mg, 0.15 mmol, 1.5 eq.) was added. The reaction mixture was stirred at 60 °C for 2 hours. The concentrated mixture was purified by column chromatography on silica gel to afford compound **7c** as a white solid (35.7 mg, 0.76 mmol, 76% yield, 89% ee).

2.5 Computational study

All calculations were conducted using Gaussian16 software package.[2] Optimization of all stationary points was carried out at M062X-D3/6-31G(d) theoretical level.[3-8] Frequency calculations were performed at the same level to verify whether the stationary points are minima (0 imaginary frequency) or saddle points (only 1 imaginary frequency). Single point calculations were carried out with M062X-D3/6-311+G(d,p) theoretical level for all atoms. Dispersion effects are described using Grimme's D3 corrections. Computed structures were illustrated by CYLView software.[9]

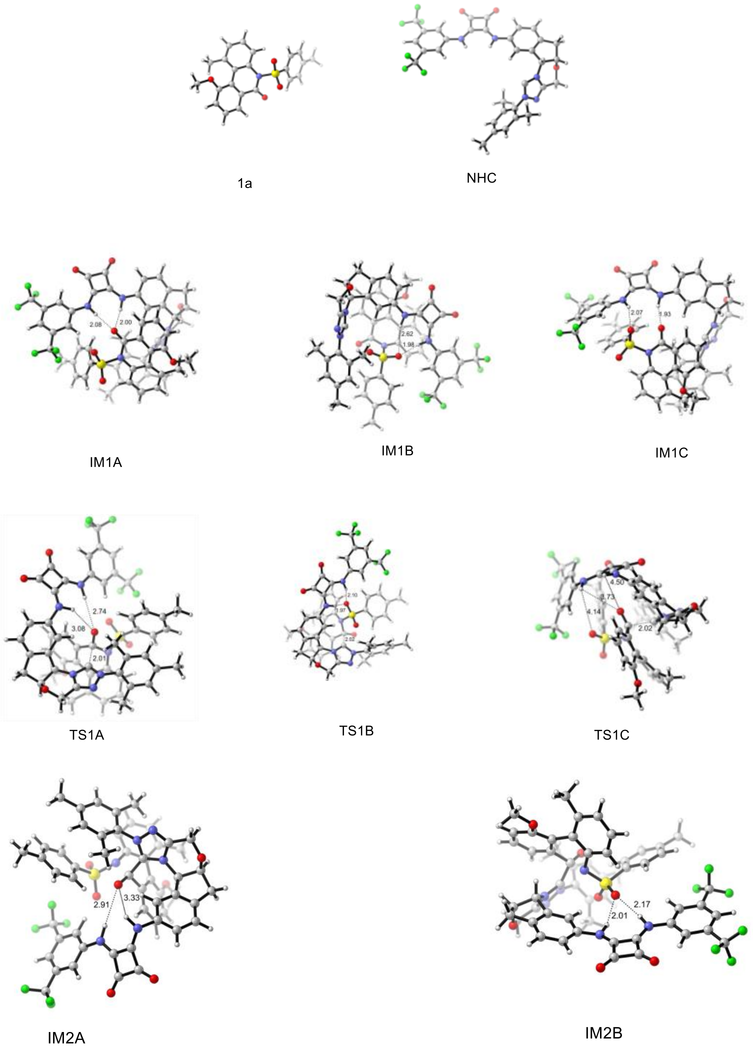

Supplementary Figure 4. Computed structures of 1a, NHC, IM1A, IM1B, IM1C, TS1A, TS1B, TS1C, IM2A, IM2B, selected bond distance (Å) (color code, C: grey, N: blue, O: red, H: white, F: green, S: yellow.).

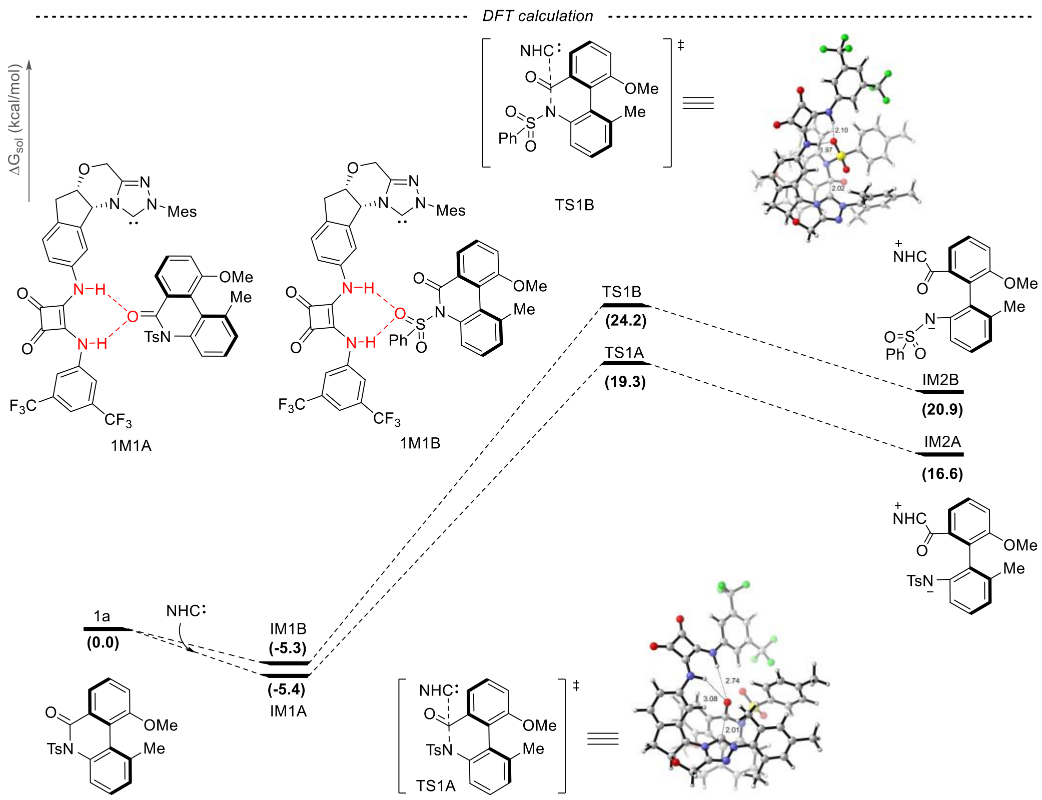

Supplementary Figure 5. DFT calculations. Gibbs free energies were given in kcal/mol, at M062X-D3/6-31G(d)-SMD(DCM)//M062X-D3/6-311+G(d,p)-SMD(DCM) theoretical level. (color code, C: grey, N: blue, O: red, H: white, F: green, Br: reddish brown, S: yellow.).

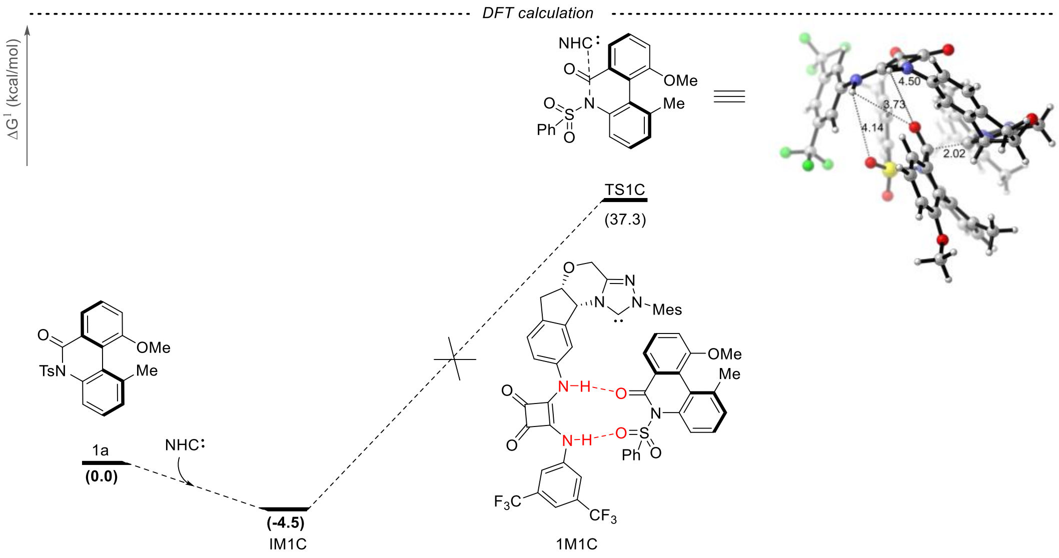

Supplementary Figure 6. DFT calculations. Gibbs free energies were given in kcal/mol, at M062X-D3/6-31G(d)-SMD(DCM) theoretical level. (color code, C: grey, N: blue, O: red, H: white, F: green, Br: reddish brown, S: yellow.).

Supplementary Table 5. Energies and other thermodynamic parameters.

Structures	Eele	Eele(SP)	E0	E	H	G
1a	-1603.077371	-1603.428168	-1602.70636	-1602.682465	-1602.681521	-1602.760219
NHC	-2370.171445	-2370.854797	-2369.62884	-2369.588354	-2369.58741	-2369.710062
IM1A	-3973.288298	-3974.323264	-3972.373523	-3972.308284	-3972.30734	-3972.478075
IM1B	-3973.291368	-3974.324791	-3972.376164	-3972.311203	-3972.310259	-3972.479556
IM1C	-3973.290986	-3974.324982	-3972.375759	-3972.310912	-3972.309968	-3972.477637
TS1A	-3973.256436	-3974.288315	-3972.340749	-3972.277079	-3972.276135	-3972.441754
TS1B	-3973.248127	-3974.278739	-3972.332603	-3972.268536	-3972.267592	-3972.435246
TS1C	-3973.230119	-3974.29895	-3972.313901	-3972.250667	-3972.249723	-3972.410867
IM2A	-3973.262826	-3974.294496	-3972.345829	-3972.281997	-3972.281053	-3972.446279
IM2B	-3973.262826	-3974.283407	-3972.345829	-3972.281997	-3972.281053	-3972.441162

Notes: Eele, E0, E, H, and G were the electronic energies, sum of electronic and zero-point energies, sum of electronic and thermal energies, sum of electronic and thermal enthalpies, and sum of electronic and thermal free energies, respectively, which were given at the M062X-D3/6-31g(d)-SMD(DCM) level. Eele(SP) were single point electronic energies at the M062X-D3/6-311+g(d,p)-SMD(DCM) level.

Coordinates for all stationary points

1a

C	-3.82645200	-0.57225400	-0.04542900
C	-4.40109700	-1.81263700	-0.32976400
C	-2.44715100	-0.36172800	-0.27221200
C	-1.66562000	-1.49189200	-0.55193400
C	-2.22936900	-2.74266500	-0.81199100
C	-3.60568600	-2.88337300	-0.73959300
C	-1.77815700	0.94932200	-0.20335400
C	-2.36364700	2.16318300	-0.63819300
C	-1.63484400	3.34581500	-0.50971900
C	-0.32876000	3.34880500	-0.03688000
C	0.30227100	2.15076100	0.26146800
C	-0.41577000	0.95929200	0.17242200
C	-0.19677700	-1.43067900	-0.37770900
O	0.55992800	-2.32553000	-0.68887900
O	-4.51020700	0.45537800	0.50037200
C	-5.92414400	0.36077900	0.56560600
C	-3.69166200	2.25493300	-1.35171900
N	0.24358500	-0.29566300	0.34089400
C	3.03538700	-0.24388200	0.49795300
C	3.77640800	0.91238700	0.72071900
C	4.95793900	1.09602800	0.00748500
C	5.40036100	0.14077700	-0.91038600
C	4.63608000	-1.01954800	-1.09967800
C	3.45663100	-1.22634700	-0.39947400
C	6.67482600	0.33823000	-1.68508400

S	1.55641300	-0.50275600	1.43760900
O	1.49858700	-1.88249000	1.87653900
O	1.42076000	0.57136300	2.40469000
H	-5.46716700	-1.96003600	-0.20270400
H	-1.57909100	-3.58203200	-1.03128800
H	-4.07077200	-3.84119900	-0.94937200
H	-2.09313700	4.27631500	-0.83379900
H	0.22540200	4.27895700	0.03855900
H	1.34883300	2.14680000	0.53434500
H	-6.26976700	1.31577600	0.96210900
H	-6.35447400	0.19736300	-0.42923400
H	-6.23656600	-0.44415200	1.23941900
H	-3.61994400	3.02053700	-2.12983000
H	-3.97018800	1.31239800	-1.82963900
H	-4.50134900	2.53922200	-0.67440500
H	3.44486100	1.64716400	1.44758300
H	5.54688400	1.99331300	0.17393200
H	4.97731900	-1.77334500	-1.80418400
H	2.86330400	-2.12260800	-0.54403400
H	7.17616700	1.26499800	-1.39635000
H	7.36432100	-0.49521000	-1.51575100
H	6.47054000	0.37787000	-2.76032000

NHC-free

C	1.31117300	-4.21333100	-0.34689100
C	1.16016400	-2.83474700	-0.52600800
C	2.27429800	-2.02261100	-0.77577500
C	3.52247600	-2.61549000	-0.83194600
C	3.69235000	-3.98869400	-0.65046200
C	2.58148700	-4.78691400	-0.40854800
C	4.86506800	-1.96777600	-1.10004300
C	5.86286600	-3.01524700	-0.54289900
C	5.15430300	-4.35716100	-0.75782800
N	5.05340300	-0.67194500	-0.46133900
C	5.84965800	-0.49020000	0.63907100
C	6.64254700	-1.63177100	1.19608800
O	6.00667900	-2.84736200	0.86087000
C	4.44248900	0.50174000	-0.82074600
N	4.94115500	1.32199900	0.12459600
N	5.80447700	0.74425300	1.03407500
N	-0.09144900	-2.18855900	-0.47126900
C	-1.29614200	-2.66776200	-0.12198000
C	-1.90195300	-3.95030500	0.31903200
C	-3.23102200	-3.22532700	0.40445100

C	-2.52828500	-2.00519000	-0.04235500
O	-1.54826600	-5.08747500	0.53006800
O	-4.35303100	-3.55996500	0.71790900
N	-2.81447100	-0.70961600	-0.30849000
C	-4.03880600	-0.04017700	-0.23226000
C	-4.05026700	1.31795200	-0.58135800
C	-5.23281400	2.03481500	-0.51815600
C	-6.42593300	1.43690200	-0.11429400
C	-6.39408000	0.09285200	0.22447700
C	-5.22113400	-0.65773800	0.17376600
C	4.61701500	2.70804400	0.28476300
C	5.22796500	3.65031100	-0.54694800
C	4.89415400	4.99275300	-0.36151500
C	3.98784600	5.39462300	0.62154800
C	3.41044300	4.42043900	1.43932600
C	3.71091500	3.06710000	1.28807400
C	3.09035000	2.01993400	2.17366400
C	6.21525200	3.22589900	-1.60102600
C	3.62089000	6.84721700	0.78291700
C	-5.25779500	3.48068900	-0.92561300
F	-4.02904600	4.00943000	-1.00138600
F	-5.96279200	4.22400000	-0.05935700
F	-5.83277100	3.64479300	-2.12838000
C	-7.64717500	-0.61327300	0.66146000
F	-8.69424300	0.21697800	0.75174700
F	-7.49191500	-1.19371400	1.86161700
F	-7.98579900	-1.59017800	-0.19515600
H	0.44620700	-4.83609700	-0.14674400
H	2.16015800	-0.94963900	-0.91003300
H	2.68934200	-5.85713100	-0.25582000
H	5.02133600	-1.83973500	-2.17618200
H	6.84470200	-2.94705100	-1.02816000
H	5.39100900	-4.75147200	-1.75306300
H	5.48299300	-5.08899500	-0.01517600
H	7.66636200	-1.60578400	0.79568200
H	6.69074200	-1.56915200	2.28455800
H	-0.05353900	-1.20075100	-0.70773400
H	-2.03760200	-0.12932800	-0.61185500
H	-3.13172500	1.80423200	-0.89588100
H	-7.34621500	2.00791900	-0.06373100
H	-5.23487100	-1.70835900	0.45185500
H	5.36025200	5.74263700	-0.99700400
H	2.70834300	4.72005600	2.21447600
H	2.30732400	2.45773900	2.79713000

H	2.65165800	1.21077300	1.57939500
H	3.84016200	1.56582600	2.83024700
H	6.99279100	2.58301900	-1.17473100
H	5.72279800	2.65574400	-2.39555300
H	6.69508400	4.09807400	-2.05126400
H	4.43125200	7.50027300	0.44740400
H	2.73248900	7.09103400	0.18873100
H	3.39338600	7.08456100	1.82601000

IM1A

C	1.45593700	5.12636200	-0.00551900
C	1.17793300	3.75860800	0.07573900
C	2.20116900	2.81503500	-0.06241100
C	3.48685700	3.26446200	-0.29118800
C	3.78576500	4.62313900	-0.38619600
C	2.76409900	5.55552800	-0.24199800
C	4.73698200	2.43815500	-0.49932400
C	5.68805200	3.43939500	-1.20758600
C	5.26073100	4.81053900	-0.66460000
N	4.50415300	1.25186600	-1.31342800
C	4.89178500	1.15396400	-2.62391100
C	5.73637100	2.22905100	-3.23214200
O	5.43043900	3.45832600	-2.60492800
C	3.72601700	0.18197400	-0.95482800
N	3.71621000	-0.50201100	-2.11592400
N	4.41982600	0.07090600	-3.15889300
N	-0.11190400	3.24085100	0.30821100
C	-1.30462800	3.81907200	0.10769100
C	-1.85290500	5.11575000	-0.36322300
C	-3.24050900	4.50113400	-0.23408100
C	-2.58231800	3.26961200	0.24450000
O	-1.42596400	6.19748000	-0.69392000
O	-4.36606400	4.88527700	-0.46830800
N	-2.92643300	2.03905700	0.69242500
C	-4.15936800	1.39017500	0.59413900
C	-4.25833500	0.12277300	1.17678700
C	-5.42716500	-0.61089700	1.04481600
C	-6.53021700	-0.10393300	0.36583100
C	-6.42254300	1.17006300	-0.18033900
C	-5.25613500	1.92436400	-0.08622800
C	2.93784400	-1.67272600	-2.38455000
C	3.59450500	-2.87629400	-2.65749000
C	2.81299500	-3.99481700	-2.94948500
C	1.41871900	-3.93264900	-2.95965200

C	0.80244300	-2.71038600	-2.68546200
C	1.54209500	-1.56010200	-2.40701200
C	0.84655600	-0.24739200	-2.15653200
C	5.09637400	-2.96242800	-2.61836100
C	0.59006500	-5.16363500	-3.22098100
C	-5.43693000	-1.99840800	1.61989400
F	-4.58738000	-2.80680200	0.95970900
F	-6.64882300	-2.56639200	1.55982100
F	-5.05002300	-2.00662300	2.90354000
C	-7.59114200	1.78910700	-0.89366600
F	-8.58603300	0.91488600	-1.09604800
F	-7.23599700	2.27542400	-2.09395700
F	-8.10341700	2.81916600	-0.20087700
H	0.65446800	5.85078800	0.09128900
H	1.98643200	1.75007000	-0.01492300
H	2.96806600	6.61964900	-0.32127900
H	5.16349000	2.12312300	0.45877800
H	6.74418000	3.20138500	-1.02848800
H	5.81178700	5.03774000	0.25557800
H	5.47884500	5.59550000	-1.39357900
H	6.80243700	1.98691100	-3.11229300
H	5.51618800	2.33248700	-4.29575000
H	-0.12947100	2.24451900	0.53502400
H	-2.18190400	1.48418300	1.11454500
H	-3.41188300	-0.29448700	1.71699100
H	-7.44092300	-0.68172100	0.26521400
H	-5.21017900	2.91556900	-0.52868100
H	3.30841100	-4.94038200	-3.16083100
H	-0.28365900	-2.64188900	-2.70857200
H	-0.15326100	-0.26092700	-2.59833200
H	0.73586900	-0.05254300	-1.08332800
H	1.40376300	0.59197700	-2.58403900
H	5.54636800	-2.45674500	-3.47936100
H	5.49103000	-2.47999200	-1.71728100
H	5.42061100	-4.00618300	-2.62501300
H	1.15311200	-5.90879600	-3.78992100
H	0.28110900	-5.63140400	-2.27756900
H	-0.32022000	-4.91787900	-3.77687800
C	3.63238100	-0.27459300	3.68460800
C	3.54883100	1.00552400	4.23389900
C	2.75405000	-0.66025100	2.64512900
C	1.67136100	0.19172800	2.37649800
C	1.55777600	1.46018700	2.95578200
C	2.52824900	1.87688900	3.84980800

C	2.88128800	-1.90199900	1.86949300
C	4.11656200	-2.48850900	1.49755000
C	4.10273500	-3.69418700	0.79919300
C	2.91364600	-4.28471500	0.38501000
C	1.70861500	-3.63587200	0.59961500
C	1.69727800	-2.45128800	1.33488300
C	0.51723600	-0.31633600	1.61724800
O	-0.41509900	0.39215800	1.24609100
O	4.48650400	-1.21803700	4.13076700
C	5.52192600	-0.82192300	5.01628500
C	5.45682500	-1.81873100	1.68529800
N	0.48439800	-1.70471300	1.45771900
C	-1.70338900	-2.64905800	-0.02259000
C	-1.95931200	-3.92381900	-0.52048500
C	-2.61181000	-4.04055300	-1.74383900
C	-3.01305300	-2.90774900	-2.46013500
C	-2.73932900	-1.64051400	-1.92819000
C	-2.08479500	-1.49915000	-0.71140000
C	-3.69106600	-3.04506700	-3.79536000
S	-1.04274100	-2.51149800	1.61068100
O	-1.87158100	-1.62231400	2.40302900
O	-0.73384900	-3.83490200	2.11386400
H	4.25608800	1.31812300	4.99307400
H	0.70009000	2.08295400	2.72459100
H	2.47221800	2.86349400	4.29765800
H	5.05183400	-4.15075900	0.52971900
H	2.92854300	-5.21277200	-0.17741300
H	0.79712000	-4.03324900	0.17081700
H	6.13272500	-1.71039000	5.17799300
H	6.13465300	-0.02786400	4.57424000
H	5.11541700	-0.48422000	5.97551500
H	6.07027700	-2.00993700	0.79926500
H	5.36023200	-0.73642000	1.80090100
H	5.99377100	-2.20677300	2.55495100
H	-1.67085700	-4.80374100	0.04602800
H	-2.82338800	-5.02907400	-2.14199500
H	-3.04840600	-0.75198200	-2.47280800
H	-1.86721400	-0.51667000	-0.30455600
H	-4.23009600	-3.99288400	-3.86993600
H	-4.39318600	-2.22527100	-3.96856700
H	-2.94701300	-3.01973400	-4.60049800

IM1B

C	-1.64193200	-4.32336800	-1.16034900

C	-1.44663100	-2.97017600	-0.86545200
C	-2.53361800	-2.14493400	-0.54529000
C	-3.80085000	-2.70255300	-0.55240400
C	-4.01735600	-4.04318000	-0.87608200
C	-2.93053600	-4.85849700	-1.16455500
C	-5.13002900	-2.05363800	-0.22229400
C	-6.13516100	-2.96158300	-0.96462400
C	-5.49841700	-4.35317200	-0.87626600
N	-5.25849500	-0.67425200	-0.65186800
C	-5.92689400	-0.29791500	-1.78695200
C	-6.70833700	-1.31493200	-2.56343600
O	-6.16812300	-2.60290800	-2.34119300
C	-4.65840700	0.39936600	-0.05548100
N	-5.02053200	1.37605900	-0.91108300
N	-5.80111400	0.97887300	-1.98205400
N	-0.16885400	-2.37843500	-0.88467300
C	1.03552300	-2.95915900	-1.01430300
C	1.62611700	-4.32007500	-1.04293100
C	2.98954400	-3.66246200	-1.14853200
C	2.29507600	-2.36141800	-1.15037300
O	1.24637100	-5.46986800	-1.02453800
O	4.12841000	-4.07857800	-1.19425000
N	2.59669300	-1.04942800	-1.25624900
C	3.84516400	-0.42999200	-1.23723800
C	3.85257100	0.96717700	-1.18964800
C	5.05021000	1.65213700	-1.05819900
C	6.26711800	0.98021600	-1.01017800
C	6.24207600	-0.40860000	-1.09784900
C	5.05506400	-1.12715100	-1.20206800
C	-4.58042200	2.73605500	-0.82983400
C	-4.99465600	3.52775400	0.24377300
C	-4.48376200	4.82572900	0.33128900
C	-3.59347500	5.33088800	-0.61488700
C	-3.21115100	4.50711700	-1.67699600
C	-3.68748800	3.20440700	-1.80246000
C	-3.20639500	2.30672700	-2.91025000
C	-5.93256400	3.00164100	1.29781400
C	-3.04967500	6.73200100	-0.50191000
C	4.97734400	3.13675100	-0.85027300
F	4.16822200	3.73358800	-1.74317000
F	6.17349300	3.72810700	-0.93341600
F	4.47132800	3.42717800	0.36453000
C	7.53792000	-1.17019200	-1.11449800
F	8.52663900	-0.49156900	-0.51432800

F	7.94644000	-1.42561100	-2.36864500
F	7.42943600	-2.35422200	-0.49470400
H	-0.79337100	-4.95656600	-1.39412200
H	-2.38630300	-1.09351200	-0.30110800
H	-3.07156300	-5.90502200	-1.42106300
H	-5.31099000	-2.09048300	0.85758900
H	-7.14485300	-2.90329100	-0.53840000
H	-5.80669000	-4.84514100	0.05411500
H	-5.81579500	-4.98014800	-1.71390400
H	-7.76399500	-1.28086800	-2.25692800
H	-6.64920600	-1.11231300	-3.63416800
H	-0.17129900	-1.36401200	-0.80349400
H	1.80981300	-0.40133200	-1.23227600
H	2.90806800	1.50367400	-1.20492100
H	7.20151200	1.51800700	-0.90026100
H	5.07220600	-2.21325700	-1.23184900
H	-4.78838200	5.45237700	1.16688300
H	-2.50231000	4.88036100	-2.41367200
H	-2.35235100	2.75894200	-3.42107400
H	-2.89517200	1.33654900	-2.50818400
H	-3.99210500	2.12044400	-3.64929200
H	-6.77311800	2.46127700	0.85101800
H	-5.41497800	2.30491800	1.96520500
H	-6.32822000	3.82376100	1.89951300
H	-3.19645500	7.13360500	0.50455400
H	-1.97911900	6.75665000	-0.72967500
H	-3.54888400	7.40604700	-1.20752200
C	-0.73486200	-3.39168200	2.15126800
C	-2.00666200	-3.82535300	2.53808700
C	-0.37429900	-2.03427300	2.29344700
C	-1.40517600	-1.13047700	2.61079600
C	-2.68355600	-1.55361700	2.97539800
C	-2.96354800	-2.91265500	2.97725400
C	0.99380100	-1.49888300	2.12470900
C	2.17880000	-2.16444200	2.51858200
C	3.40591500	-1.51515300	2.34501000
C	3.48980600	-0.20873000	1.88323600
C	2.32826600	0.49584200	1.59906900
C	1.10315600	-0.15298600	1.70831600
C	-1.22046500	0.31692600	2.35113600
O	-1.98369700	1.18963000	2.69029700
O	0.17335800	-4.20767900	1.57322500
C	-0.01110900	-5.61051400	1.71640400
C	2.21239500	-3.49996100	3.22031500

N	-0.10336900	0.58350900	1.50838100
C	0.24883200	3.24359100	0.68403700
C	1.32667300	3.82637400	0.02749200
C	1.70985700	5.11363300	0.39350700
C	1.02210100	5.81210400	1.38837600
C	-0.06722000	5.19469200	2.02262100
C	-0.46653800	3.91214200	1.67865700
C	1.42449400	7.20867600	1.77624500
S	-0.29657500	1.63961500	0.18899500
O	-1.71416000	1.67384300	-0.09990900
O	0.62755100	1.13026000	-0.82749500
H	-2.26649600	-4.87495900	2.46352100
H	-3.44087700	-0.81294300	3.20766400
H	-3.94705000	-3.26925700	3.26713200
H	4.31237000	-2.04024200	2.63398700
H	4.45463100	0.28058100	1.78384500
H	2.37840000	1.54248800	1.31483900
H	0.90190200	-6.07558600	1.34882500
H	-0.17002100	-5.87264100	2.76854300
H	-0.85706700	-5.95788600	1.11362300
H	3.04215100	-3.50531900	3.93260300
H	1.28924500	-3.69930100	3.77035800
H	2.36783100	-4.32259200	2.51654700
H	1.84791400	3.29840700	-0.76418800
H	2.55547900	5.57605800	-0.10712100
H	-0.60937400	5.73270400	2.79561700
H	-1.31054000	3.43054300	2.16464700
H	2.31867400	7.52914500	1.23663400
H	0.61726100	7.91549400	1.55560100
H	1.62654100	7.27034300	2.85039000

IM1C

C	-0.66601200	5.14159600	-1.66814600
C	-0.68782900	3.78035100	-1.34982100
C	-1.89510200	3.13474000	-1.05532700
C	-3.05623400	3.88643300	-1.06546600
C	-3.04930100	5.25203600	-1.35437300
C	-1.84877300	5.88139200	-1.65920900
C	-4.47214500	3.43411400	-0.77569200
C	-5.17798600	4.76413500	-0.41060900
C	-4.44969400	5.81403000	-1.25749000
N	-4.58606100	2.46549300	0.30555500
C	-5.02830400	2.79492300	1.56009700
C	-5.51884200	4.18017700	1.84891400

O	-4.91976100	5.08846600	0.94905400
C	-4.22589500	1.14472900	0.24596700
N	-4.48734700	0.77337100	1.51522800
N	-4.98119300	1.76328500	2.34384600
N	0.49392600	3.00639300	-1.33151200
C	1.71802800	3.43746900	-1.00142200
C	2.30523700	4.68490400	-0.43890000
C	3.65008800	3.96645200	-0.42722000
C	2.95303000	2.78436400	-0.96674400
O	1.91049100	5.78418100	-0.12998200
O	4.77780600	4.24915400	-0.08466300
N	3.32091900	1.54447700	-1.36904900
C	4.43426500	0.83532500	-0.91173400
C	4.81411200	-0.32420600	-1.59416600
C	5.84889900	-1.10889700	-1.09785800
C	6.54234300	-0.75405000	0.05162600
C	6.16936700	0.42037100	0.70077400
C	5.12458000	1.21179400	0.24738700
C	-4.31114800	-0.53791000	2.06382300
C	-5.44958400	-1.26055500	2.44505400
C	-5.26284700	-2.51541100	3.02229800
C	-3.98717600	-3.05207300	3.21653800
C	-2.88261000	-2.30997200	2.80079500
C	-3.01823100	-1.04365300	2.22832700
C	-1.80056100	-0.25447400	1.82708300
C	-6.83177900	-0.70005400	2.23771800
C	-3.81785200	-4.40269200	3.86294000
C	6.13311200	-2.41763700	-1.77543900
F	6.06510800	-2.31837900	-3.11079100
F	5.23663000	-3.35368700	-1.41264700
F	7.34562300	-2.89648500	-1.46709600
C	6.88845900	0.78143100	1.96844500
F	6.86532000	-0.24015100	2.84686400
F	6.34827500	1.84104300	2.58305200
F	8.18212900	1.06154000	1.74950700
H	0.27416800	5.62429600	-1.90844100
H	-1.91058900	2.07635000	-0.80903400
H	-1.81866900	6.94284100	-1.88821900
H	-4.93022000	2.99750500	-1.66949100
H	-6.26055800	4.72108000	-0.58553900
H	-4.91739600	5.88830700	-2.24637100
H	-4.50063900	6.79685300	-0.78162500
H	-6.61460300	4.21112300	1.75950500
H	-5.24220300	4.48235700	2.86043500

H	0.37100600	2.00526000	-1.48451200
H	2.64822000	1.00166000	-1.91050600
H	4.28546700	-0.62050200	-2.49495000
H	7.34245400	-1.37582800	0.43961600
H	4.84112600	2.10011200	0.79746600
H	-6.13655900	-3.08889100	3.32641800
H	-1.88340100	-2.71964300	2.93057600
H	-0.89669700	-0.85340800	1.97172900
H	-1.85752300	0.05712000	0.78050200
H	-1.70782500	0.66067200	2.42507100
H	-7.04281900	0.10685200	2.94684400
H	-6.94077100	-0.28328600	1.23086700
H	-7.58370600	-1.48133300	2.37286200
H	-4.34160900	-5.18237100	3.29798100
H	-2.76224900	-4.67961000	3.92908100
H	-4.23490700	-4.40378000	4.87605300
C	-3.69220500	-3.03581600	-2.67469500
C	-4.34975800	-2.13813700	-3.51668800
C	-2.69167600	-2.57329300	-1.78766200
C	-2.23290800	-1.26152600	-1.97767100
C	-2.87070600	-0.36191200	-2.83720300
C	-3.95871700	-0.79919600	-3.57162900
C	-2.08478100	-3.38234700	-0.71894800
C	-2.78060800	-4.36506900	0.02741400
C	-2.07904300	-5.11336400	0.97213600
C	-0.74826100	-4.84864900	1.27056300
C	-0.10243500	-3.78170400	0.66353400
C	-0.76660600	-3.05851800	-0.32771600
C	-0.93493100	-0.85170200	-1.41641800
O	-0.50638600	0.28850100	-1.49007000
O	-3.91298300	-4.36649700	-2.69311500
C	-5.03684500	-4.85102300	-3.41098600
C	-4.27560800	-4.56678800	-0.02903900
N	-0.15419600	-1.89926800	-0.90049300
C	2.13794900	-1.12328200	0.45640300
C	3.17713400	-1.77986600	1.11108200
C	3.69216900	-1.21298500	2.27275200
C	3.18800800	-0.00787800	2.77258600
C	2.14589800	0.62854700	2.08342100
C	1.61294100	0.08179900	0.92345500
C	3.74654200	0.59916600	4.03048900
S	1.56082900	-1.80420500	-1.06936000
O	1.80408400	-0.87341800	-2.16229600
O	2.03304700	-3.16792000	-1.20024300

H	-5.15125300	-2.48434200	-4.15864200
H	-2.48467600	0.64712400	-2.93153800
H	-4.48190300	-0.11831200	-4.23501600
H	-2.61118300	-5.88476400	1.52253100
H	-0.22881700	-5.42780500	2.02763300
H	0.90112100	-3.51872300	0.97223800
H	-5.07285900	-5.92401800	-3.22078400
H	-5.96199700	-4.38224600	-3.05675600
H	-4.92368100	-4.67927300	-4.48682600
H	-4.63729400	-4.76675300	0.98419600
H	-4.79510200	-3.67918100	-0.39984800
H	-4.55443900	-5.41444800	-0.66040200
H	3.58124500	-2.70483000	0.71100200
H	4.51149900	-1.70634000	2.78755900
H	1.74973600	1.56816600	2.45963300
H	0.80450800	0.57836500	0.39304300
H	4.74849300	0.21991000	4.24266000
H	3.79323400	1.68896400	3.95242200
H	3.10513500	0.35730000	4.88573700

TS1A

C	1.29575400	5.38577000	-0.82556900
C	0.79642300	4.10085100	-1.04285300
C	1.61220100	2.98013900	-0.89510700
C	2.90933900	3.17036300	-0.47873300
C	3.45196800	4.43903700	-0.28601600
C	2.63692000	5.55364300	-0.45858300
C	3.91108000	2.11500900	-0.09263800
C	5.25086100	2.86385400	-0.28968600
C	4.90891600	4.30901300	0.11108800
N	3.82085700	0.90370600	-0.91189200
C	4.67427000	0.70474800	-1.96560900
C	5.86147300	1.59624200	-2.17227900
O	5.58993200	2.88469100	-1.66841600
C	2.86566600	-0.07363200	-0.92093000
N	3.20657000	-0.79708200	-1.99668800
N	4.32124200	-0.33492900	-2.65336400
N	-0.56138300	3.82859600	-1.36343300
C	-1.60934700	4.31759500	-0.64491300
C	-2.10329700	5.63630400	-0.21807900
C	-3.32450400	4.90251200	0.36772000
C	-2.67228600	3.63484300	-0.07105900
O	-1.71835800	6.78096600	-0.25174700
O	-4.34936500	5.22599900	0.91431200

N	-2.82637300	2.30268200	0.06329700
C	-3.91337700	1.60149700	0.59573900
C	-3.70555800	0.26614600	0.94402200
C	-4.76302400	-0.48634000	1.43848300
C	-6.02743600	0.06532800	1.60400100
C	-6.20857400	1.40337900	1.26130400
C	-5.17491000	2.18143000	0.75434400
C	2.57925800	-1.96954300	-2.54228200
C	3.27848500	-3.17971500	-2.46575800
C	2.67127200	-4.30820800	-3.01465100
C	1.43650000	-4.23351500	-3.66445300
C	0.81887200	-2.98917200	-3.78677400
C	1.37437000	-1.83265000	-3.23670100
C	0.72971800	-0.48797400	-3.43679100
C	4.66694600	-3.25171700	-1.88865000
C	0.80485700	-5.47311100	-4.24202000
C	-4.48836100	-1.91333100	1.82544000
F	-3.77690900	-2.55180000	0.88364000
F	-5.61966500	-2.60995600	2.01214600
F	-3.78456700	-1.99119100	2.96368900
C	-7.55822000	2.02644500	1.48312400
F	-7.72337200	2.41176700	2.75929700
F	-8.55098700	1.16559200	1.21005000
F	-7.74691900	3.11145600	0.71994000
H	0.63912800	6.24415200	-0.93714000
H	1.19216100	1.98578600	-1.03043900
H	3.02731200	6.55673100	-0.31206900
H	3.79482300	1.83504500	0.95914600
H	6.06885100	2.42144100	0.29264000
H	5.04225500	4.43664400	1.19173400
H	5.57214000	5.01267200	-0.39906900
H	6.73490000	1.15579900	-1.66978100
H	6.07778900	1.68834900	-3.23767800
H	-0.69177000	2.85489600	-1.63069100
H	-1.99851900	1.73468200	-0.12223300
H	-2.71589700	-0.17733300	0.84844000
H	-6.85174900	-0.52744800	1.98462700
H	-5.34566700	3.22128200	0.49589600
H	3.18500600	-5.26521300	-2.94778600
H	-0.11864000	-2.90968200	-4.33169600
H	-0.09861800	-0.56901600	-4.14570400
H	0.34980600	-0.09521700	-2.48832100
H	1.45414700	0.23238500	-3.83566200
H	5.40060900	-2.94978100	-2.64482200

H	4.78843700	-2.59189300	-1.02550800
H	4.89986000	-4.27274100	-1.57726900
H	1.45565100	-5.93009000	-4.99543600
H	0.63503300	-6.22303900	-3.46185900
H	-0.15610800	-5.24650900	-4.71283500
C	2.94900900	0.41124000	3.52141300
C	2.35499000	1.62158000	3.89100900
C	2.68430300	-0.15416700	2.25217600
C	1.68667600	0.44698100	1.47283000
C	1.03374500	1.60870100	1.87340900
C	1.40421400	2.21707200	3.06436100
C	3.27448000	-1.42957600	1.79021000
C	4.58958600	-1.86376600	2.07367900
C	4.92723900	-3.19132600	1.80400600
C	4.02699700	-4.06685700	1.20287200
C	2.80558200	-3.59119400	0.74627400
C	2.44446700	-2.27355800	1.01838800
C	1.22114500	-0.25373800	0.22106600
O	0.23404000	0.17960800	-0.40917600
O	3.74364600	-0.30055100	4.35110800
C	4.10966200	0.27551900	5.59292900
C	5.68066000	-0.94201200	2.56388800
N	1.25876600	-1.72410000	0.44899400
C	-1.03607400	-2.88029800	-0.63053500
C	-1.06372700	-4.23128300	-0.95668000
C	-1.77124300	-4.63619400	-2.08702500
C	-2.44634000	-3.70981400	-2.88331000
C	-2.40644800	-2.35528400	-2.52218800
C	-1.71024300	-1.93136100	-1.39935900
C	-3.18632300	-4.14061500	-4.12098200
S	-0.24070000	-2.40833200	0.88603500
O	-1.06018400	-1.39706700	1.54235700
O	0.07378700	-3.63002400	1.61333400
H	2.59342200	2.07714700	4.84467900
H	0.24492800	2.01279700	1.24879900
H	0.92863800	3.14086300	3.37931500
H	5.92979200	-3.53333600	2.04981600
H	4.31189400	-5.09703300	1.01175100
H	2.13844600	-4.21856400	0.16514100
H	4.77144700	-0.44604600	6.07302000
H	4.64296200	1.22222400	5.44872300
H	3.23270400	0.44115600	6.22824500
H	6.58342500	-1.11187400	1.96754300
H	5.40166400	0.11043900	2.46715600

H	5.93209000	-1.12542500	3.61210400
H	-0.55720900	-4.95565300	-0.32738900
H	-1.80428700	-5.69077000	-2.34664400
H	-2.93414700	-1.62530800	-3.13137900
H	-1.66993800	-0.88454500	-1.12083700
H	-3.20087600	-5.22915700	-4.21717300
H	-4.22012500	-3.78073200	-4.10523300
H	-2.71291100	-3.72476300	-5.01808800

TS1B

C	-0.38499100	-4.25391000	-2.46767200
C	-0.24612400	-2.93492500	-2.02638300
C	-1.30145300	-2.29140600	-1.37138900
C	-2.46078400	-2.99340300	-1.14011100
C	-2.63219100	-4.30547900	-1.58002900
C	-1.58799800	-4.93333600	-2.24971800
C	-3.67995200	-2.54248700	-0.37851900
C	-4.77594500	-3.48726600	-0.92512700
C	-4.01763800	-4.79171000	-1.21141500
N	-4.04123100	-1.13678500	-0.56336700
C	-5.08666800	-0.75939000	-1.36489400
C	-5.95319700	-1.78275600	-2.03586800
O	-5.24907300	-2.99652200	-2.17101000
C	-3.46183900	-0.02334700	-0.02632300
N	-4.20463800	0.96662200	-0.54821000
N	-5.20968400	0.53093400	-1.38077700
N	0.91884600	-2.16512300	-2.17059200
C	2.11718500	-2.47530800	-2.67937300
C	2.79120300	-3.58292700	-3.39497300
C	4.05277500	-2.72918100	-3.39430200
C	3.27662800	-1.69493200	-2.68102400
O	2.50422000	-4.68356000	-3.80725900
O	5.18201100	-2.88117300	-3.80897000
N	3.43588700	-0.45621600	-2.16006900
C	4.58833700	0.32535800	-2.08903300
C	4.47548200	1.58204400	-1.48802200
C	5.59704200	2.38795600	-1.35013900
C	6.84101100	1.98345700	-1.81826700
C	6.92877700	0.73785000	-2.43461400
C	5.82908100	-0.09942500	-2.57561200
C	-3.99739500	2.39185000	-0.48385600
C	-4.49843600	3.12463300	0.59481800
C	-4.23096900	4.49671600	0.62209700
C	-3.52999200	5.13121000	-0.40285100

C	-3.13039200	4.37250800	-1.50612800
C	-3.37215100	3.00231100	-1.58076800
C	-3.03506000	2.24191600	-2.83626900
C	-5.31852600	2.49110800	1.68458600
C	-3.23702700	6.60840800	-0.34966600
C	5.43899500	3.68828500	-0.61576400
F	4.33053400	4.34355300	-0.99392400
F	6.47838100	4.50973800	-0.80062800
F	5.32531500	3.48773500	0.71201300
C	8.24814500	0.30678800	-3.01206100
F	8.35609600	-1.02650900	-3.08397300
F	9.28102600	0.75578800	-2.28262200
F	8.42038700	0.78085900	-4.25747800
H	0.43508500	-4.74372400	-2.98410700
H	-1.21619800	-1.25582800	-1.06663500
H	-1.69424900	-5.95165100	-2.61305200
H	-3.54527700	-2.72121200	0.69100600
H	-5.61214700	-3.60193300	-0.22298200
H	-3.99462300	-5.41155700	-0.30681300
H	-4.51558600	-5.35973700	-2.00168000
H	-6.86898900	-1.92529900	-1.44404400
H	-6.23118500	-1.44271800	-3.03490700
H	0.86047200	-1.26205800	-1.69851900
H	2.61747300	-0.04418000	-1.71208500
H	3.50921300	1.92601900	-1.12878400
H	7.71463600	2.61559400	-1.70720300
H	5.93587400	-1.06957000	-3.05291100
H	-4.59404200	5.08192700	1.46411300
H	-2.62295300	4.86078700	-2.33574500
H	-2.18073200	2.70124300	-3.34022800
H	-2.79354900	1.19719600	-2.63810000
H	-3.88776600	2.26849900	-3.52495300
H	-5.96718200	1.70519300	1.28353000
H	-4.66604100	2.04576200	2.43970800
H	-5.95101900	3.24301900	2.16382400
H	-3.47346500	7.02465300	0.63324500
H	-2.18141500	6.80534100	-0.56387400
H	-3.82604100	7.15132100	-1.09738300
C	-2.80402300	-3.19407200	3.20387000
C	-4.19409400	-3.27825500	3.28362900
C	-2.18199900	-1.96496000	2.87274400
C	-2.98782000	-0.94738200	2.36113200
C	-4.37953600	-1.01443700	2.46570000
C	-4.97356200	-2.16396300	2.96245400

C	-0.74873700	-1.68523600	3.08481800
C	-0.05109800	-2.14774100	4.22500500
C	1.29854800	-1.82356200	4.37352300
C	1.93988100	-1.01000500	3.45526500
C	1.22938600	-0.44933500	2.40215100
C	-0.11901800	-0.76198800	2.21765000
C	-2.35076300	0.22973200	1.63904200
O	-2.51009500	1.39258800	2.00462400
O	-1.97929900	-4.24819700	3.38629200
C	-2.53951400	-5.45419400	3.87829200
C	-0.69193500	-2.89715500	5.36972400
N	-0.93408900	-0.17031200	1.18803400
C	0.28622400	2.43169600	0.79069400
C	1.63379800	2.70687300	1.00727300
C	2.00086500	3.97972500	1.43502200
C	1.04095500	4.97348600	1.64281700
C	-0.30497300	4.67071000	1.39831200
C	-0.69338700	3.41069900	0.96672900
C	1.43734200	6.34202400	2.12486600
S	-0.21186500	0.87592600	0.08226800
O	-1.22310800	1.16895100	-0.91722900
O	1.00891800	0.19181200	-0.38307900
H	-4.67423600	-4.20187800	3.58414300
H	-4.98498300	-0.18486400	2.11499100
H	-6.05371400	-2.22533200	3.05184000
H	1.83446700	-2.19418300	5.24303100
H	2.99000100	-0.76255800	3.57540700
H	1.75185200	0.21772000	1.73774300
H	-1.70346900	-6.14147300	4.00977700
H	-3.04001700	-5.29385000	4.84034400
H	-3.24963100	-5.88176000	3.16141600
H	-0.22489100	-2.57592200	6.30519200
H	-1.76499500	-2.70426400	5.44654700
H	-0.55120800	-3.97777500	5.27881200
H	2.39489500	1.95569500	0.82373100
H	3.05101100	4.20462300	1.59316500
H	-1.06286800	5.43762100	1.54076000
H	-1.73555700	3.18063500	0.77622300
H	0.87767100	7.12083400	1.59848900
H	1.21986300	6.44797600	3.19379900
H	2.50582200	6.51963300	1.97982700

TS1C

C	-1.70416900	4.99799700	-2.95641800

C	-1.02725400	4.08619700	-2.14380500
C	-1.71317400	3.05187200	-1.50431000
C	-3.06656600	2.93015000	-1.73974200
C	-3.76848600	3.83731900	-2.53134600
C	-3.08305200	4.87948500	-3.14622700
C	-3.98590900	1.80765100	-1.32385500
C	-5.37260400	2.48942000	-1.39258200
C	-5.23299900	3.45489800	-2.57859300
N	-3.72234300	1.25232200	0.00458200
C	-4.45858600	1.64426400	1.09348800
C	-5.66722500	2.51798000	0.94092200
O	-5.57057200	3.28694800	-0.23575400
C	-2.74124800	0.39532000	0.41752800
N	-2.94745300	0.34168000	1.73990300
N	-4.00420000	1.10124700	2.17854500
N	0.39089300	4.13919500	-2.01616800
C	0.98319400	3.65644000	-0.89694600
C	0.62039900	3.78898200	0.52949800
C	1.88415200	2.98817300	0.85179700
C	2.07968100	2.85093200	-0.61577400
O	-0.30155600	4.25942600	1.15197300
O	2.46976700	2.64125600	1.85161300
N	2.92668700	2.14213900	-1.39687200
C	3.76120400	1.13059100	-0.86335100
C	3.70630100	-0.15117400	-1.41529500
C	4.46453800	-1.16765600	-0.85533400
C	5.28505700	-0.93349900	0.24576400
C	5.33781100	0.35016400	0.76960800
C	4.59631200	1.39272300	0.21802400
C	-2.22937100	-0.42200300	2.72210300
C	-2.86612400	-1.53358900	3.28093000
C	-2.14869300	-2.27925300	4.21435000
C	-0.85806500	-1.91230300	4.60621900
C	-0.30705100	-0.74091700	4.08851900
C	-0.97930200	0.03247100	3.14192100
C	-0.41839700	1.33011300	2.63137400
C	-4.28947900	-1.86889600	2.92437900
C	-0.08031000	-2.76617900	5.57443800
C	4.34578200	-2.57048400	-1.38138000
F	3.80720700	-2.61155600	-2.60334600
F	3.58991500	-3.33875400	-0.57921400
F	5.54985600	-3.16496200	-1.44882000
C	6.19400300	0.65359600	1.96564100
F	6.75593300	-0.44989800	2.47975400

F	5.47888900	1.22972000	2.94751200
F	7.18517800	1.50597800	1.66501200
H	-1.14975900	5.80056200	-3.43300100
H	-1.16929400	2.31917200	-0.91177600
H	-3.60577300	5.60067100	-3.76727200
H	-3.94067000	0.99011000	-2.05104800
H	-6.19170500	1.76599000	-1.49832200
H	-5.48180800	2.93937000	-3.51353500
H	-5.91580500	4.30060200	-2.46417500
H	-6.56634000	1.88501200	0.91959100
H	-5.74163300	3.20484800	1.78471100
H	0.91990600	4.12803300	-2.88075700
H	2.62942700	1.99907400	-2.35718000
H	3.02877400	-0.36674300	-2.23341300
H	5.86074100	-1.73827600	0.68920200
H	4.64065800	2.38751500	0.64761000
H	-2.60966800	-3.16577600	4.64541300
H	0.67367800	-0.41686000	4.42570000
H	0.52629700	1.56710800	3.12537000
H	-0.23655800	1.27440900	1.55256100
H	-1.12028500	2.15328500	2.80578800
H	-4.97689500	-1.19068100	3.44124600
H	-4.46821500	-1.76849800	1.84903700
H	-4.52931000	-2.89391600	3.21471100
H	0.23453400	-3.69973900	5.09481600
H	0.82038600	-2.25065800	5.91906200
H	-0.68509900	-3.03218800	6.44671700
C	-3.23258500	-1.58725300	-3.52290700
C	-2.80484100	-0.74760100	-4.55549000
C	-2.79186600	-1.36896600	-2.19681100
C	-1.80012000	-0.39800400	-2.00023700
C	-1.30708700	0.38172700	-3.04144500
C	-1.84615600	0.23349600	-4.31099000
C	-3.17250800	-2.23412800	-1.05890800
C	-4.43442700	-2.85021800	-0.89232100
C	-4.56086500	-3.86764200	0.05326400
C	-3.50254400	-4.22913200	0.88359700
C	-2.32827900	-3.49205200	0.85988900
C	-2.17365700	-2.48560300	-0.09192900
C	-1.16069400	-0.27761500	-0.64016000
O	-0.21253300	0.50761200	-0.45564000
O	-4.02448200	-2.66390000	-3.73403300
C	-4.54894100	-2.87032600	-5.03223700
C	-5.68099200	-2.38863600	-1.61048700

N	-1.02903100	-1.64039500	-0.05757800
C	1.43271800	-1.96365600	1.20862600
C	1.72680700	-3.00158500	2.08686900
C	2.53639000	-2.73981400	3.18906300
C	3.05068300	-1.46112500	3.41547700
C	2.72026700	-0.43380000	2.52294100
C	1.91754000	-0.67436200	1.41546800
C	3.95445400	-1.18225100	4.58796400
S	0.50906800	-2.35572200	-0.25583500
O	1.16868800	-1.75128800	-1.40604400
O	0.28550800	-3.79575800	-0.26073800
H	-3.17641700	-0.88862900	-5.56300200
H	-0.50882300	1.08478300	-2.83404200
H	-1.50020100	0.85452100	-5.13128100
H	-5.52389000	-4.36025200	0.16298500
H	-3.62678400	-5.02517100	1.61133600
H	-1.53711000	-3.66745300	1.58034600
H	-5.17851900	-3.75682000	-4.96179000
H	-5.15386700	-2.01538400	-5.35434200
H	-3.74913700	-3.04745700	-5.75914800
H	-6.49105700	-2.28216700	-0.88177000
H	-5.53368100	-1.42227500	-2.09920000
H	-6.00421500	-3.10211600	-2.37261800
H	1.34390500	-3.99847200	1.89658300
H	2.77662600	-3.54510000	3.87813200
H	3.09167600	0.57296900	2.68995800
H	1.64853400	0.11978400	0.72656900
H	3.71306900	-0.21955400	5.04810300
H	3.87297200	-1.96330100	5.34805400
H	4.99922600	-1.13272100	4.26350700

IM2A

C	1.17355300	5.48704900	-0.79657900
C	0.64065600	4.21945000	-1.02995200
C	1.40906800	3.07034800	-0.85001000
C	2.70491400	3.21999000	-0.40902300
C	3.28233300	4.47224600	-0.20474600
C	2.50785900	5.61265800	-0.39085900
C	3.68485600	2.14089800	-0.02571000
C	5.03898800	2.86041800	-0.23635500
C	4.73164600	4.30070900	0.20298700
N	3.57158500	0.92185500	-0.84825800
C	4.39262700	0.74586100	-1.93158200
C	5.57448500	1.63763800	-2.16808300

O	5.34412500	2.91295800	-1.62148400
C	2.66619600	-0.09512000	-0.85032600
N	2.97156900	-0.80703300	-1.94055900
N	4.04493000	-0.29885200	-2.61471400
N	-0.71598600	4.00404900	-1.41681500
C	-1.74615000	4.41587700	-0.61774700
C	-2.23393700	5.69546000	-0.08453400
C	-3.40636300	4.90272300	0.52871000
C	-2.75783900	3.67609300	-0.02688300
O	-1.86814600	6.84668700	-0.07061400
O	-4.39886700	5.16547600	1.15902200
N	-2.88365700	2.33725900	0.04153700
C	-3.91573800	1.60373500	0.64066300
C	-3.64207000	0.28930600	1.01717800
C	-4.64987100	-0.48589000	1.57912500
C	-5.92455900	0.02524400	1.78494900
C	-6.17115500	1.34544700	1.41268000
C	-5.19074500	2.14248800	0.83707700
C	2.38535000	-2.00777400	-2.48386900
C	3.12066400	-3.19207900	-2.36161800
C	2.55355000	-4.34962600	-2.89202400
C	1.33508400	-4.32149200	-3.57640400
C	0.70545500	-3.09292500	-3.77345300
C	1.21757300	-1.90742800	-3.24390100
C	0.58478100	-0.57717500	-3.54356400
C	4.51194600	-3.20316400	-1.78525900
C	0.73316600	-5.59358700	-4.11271800
C	-4.30751400	-1.89113100	1.99205400
F	-3.66307300	-2.54861200	1.01555800
F	-5.40178600	-2.60518100	2.29761200
F	-3.50961100	-1.91109400	3.06825000
C	-7.53727800	1.91519600	1.67182500
F	-7.71137800	2.21340300	2.96989400
F	-8.50435700	1.04205100	1.34731400
F	-7.76134900	3.03780400	0.97658900
H	0.54860400	6.36544800	-0.93051700
H	0.96139400	2.08578100	-0.98771700
H	2.92818000	6.60189300	-0.23382700
H	3.57956300	1.86247500	1.02542200
H	5.85869100	2.38479800	0.31615600
H	4.85852600	4.39432000	1.28779300
H	5.41494400	5.00167100	-0.28342900
H	6.46220500	1.16980300	-1.71818700
H	5.74146900	1.75110700	-3.24034800

H	-0.84968900	3.04991700	-1.74751600
H	-2.05824400	1.79627000	-0.22160300
H	-2.64076000	-0.11970000	0.89544600
H	-6.70877800	-0.58412800	2.22064500
H	-5.41095700	3.16596600	0.55415800
H	3.08750400	-5.29171300	-2.78520400
H	-0.20964500	-3.04895900	-4.35901400
H	-0.28109500	-0.70874200	-4.19750000
H	0.27753500	-0.08773300	-2.61393400
H	1.30292000	0.07709400	-4.05382000
H	5.22765400	-2.88683000	-2.55315300
H	4.61774700	-2.52751000	-0.93214400
H	4.78465000	-4.20969600	-1.46082000
H	1.41541800	-6.08784100	-4.81270600
H	0.53385300	-6.29999400	-3.29947900
H	-0.20907700	-5.39751300	-4.63229300
C	2.77586100	0.32028400	3.57452000
C	2.07075800	1.46324800	3.96312000
C	2.62585400	-0.19808000	2.26723900
C	1.65609800	0.38543600	1.44156100
C	0.89544700	1.47158100	1.85520900
C	1.13266900	2.03111100	3.10359800
C	3.27055600	-1.45531900	1.82087800
C	4.57364200	-1.87292000	2.16889100
C	4.93116300	-3.20348500	1.93941900
C	4.05194300	-4.10208600	1.34084800
C	2.84067400	-3.64898300	0.83461800
C	2.47236500	-2.32090500	1.03719800
C	1.37355600	-0.26282200	0.08120400
O	0.32802100	0.16954300	-0.55940900
O	3.57678600	-0.37075500	4.41731400
C	3.77018900	0.13211900	5.72751900
C	5.62472900	-0.93059100	2.70375100
N	1.32361800	-1.77693800	0.38693400
C	-1.05393200	-2.82142200	-0.60885700
C	-1.14880100	-4.17621300	-0.90631200
C	-1.90925200	-4.57417600	-2.00423800
C	-2.57450400	-3.63636500	-2.79596800
C	-2.46850200	-2.27853500	-2.46290900
C	-1.71515600	-1.86176300	-1.37387700
C	-3.36805600	-4.06205400	-4.00210200
S	-0.19324800	-2.35724500	0.87703000
O	-0.95780500	-1.30783200	1.54173100
O	0.07076800	-3.58734100	1.61610800

H	2.21251600	1.88232300	4.95207600
H	0.12692900	1.85329600	1.19363700
H	0.56781100	2.89838400	3.43220700
H	5.92587000	-3.53331000	2.22998400
H	4.34611700	-5.13707200	1.19414400
H	2.19053400	-4.29973400	0.25985200
H	4.44791200	-0.56732900	6.21815500
H	4.22567400	1.12874600	5.70798200
H	2.82583200	0.17136800	6.28146600
H	6.55618300	-1.07793400	2.14656600
H	5.32490500	0.11526500	2.59805200
H	5.83482600	-1.11137600	3.76144700
H	-0.65159000	-4.90821800	-0.27850000
H	-1.99347200	-5.63151200	-2.24099100
H	-2.98914100	-1.53964600	-3.06765300
H	-1.61106900	-0.81345800	-1.11710900
H	-3.51100500	-5.14542500	-4.02203200
H	-4.35145300	-3.58195900	-4.01682900
H	-2.85198800	-3.77197800	-4.92519500

IM2B

C	-0.24579800	-4.22174900	-2.87425200
C	-0.15539300	-2.87203200	-2.52710900
C	-1.19947600	-2.24499600	-1.83979300
C	-2.27822600	-2.99750500	-1.45126800
C	-2.41864700	-4.33641800	-1.81747900
C	-1.39633400	-4.94571600	-2.53572400
C	-3.44101200	-2.58682800	-0.58634600
C	-4.54189800	-3.54989900	-1.08247500
C	-3.76560800	-4.84948600	-1.34797100
N	-3.85913700	-1.18698800	-0.72313300
C	-4.87980000	-0.82818300	-1.56183500
C	-5.70108400	-1.86573500	-2.27222100
O	-5.01453200	-3.09213200	-2.34273800
C	-3.42245500	-0.06521800	-0.09641500
N	-4.17511500	0.91671300	-0.60643200
N	-5.08370100	0.45341600	-1.51817100
N	0.95060400	-2.04296900	-2.77683000
C	2.20215500	-2.34981500	-3.14445600
C	2.93893200	-3.44147300	-3.81467000
C	4.19355900	-2.58136600	-3.68788400
C	3.35250600	-1.56990700	-3.00917000
O	2.69177300	-4.54006900	-4.25612900
O	5.35098400	-2.71040500	-4.02147200

N	3.46236100	-0.36598500	-2.39925700
C	4.57947100	0.46153500	-2.28490200
C	4.42621100	1.66381800	-1.58445700
C	5.51114500	2.51701500	-1.43613300
C	6.75485700	2.20904200	-1.97698700
C	6.88369600	1.01291500	-2.67531800
C	5.82029900	0.13361900	-2.83893100
C	-4.05099200	2.35460300	-0.48639800
C	-4.62773900	3.01927700	0.59984600
C	-4.46425800	4.40308100	0.66273500
C	-3.78491900	5.11396200	-0.32849000
C	-3.30321500	4.41427200	-1.43475600
C	-3.44250500	3.03020800	-1.55059100
C	-3.02690200	2.34885000	-2.82886800
C	-5.41506600	2.29569600	1.65494100
C	-3.58992600	6.60405000	-0.22204000
C	5.34995400	3.76502700	-0.61491900
F	4.11594600	4.28048100	-0.71499800
F	6.21692900	4.71986800	-0.97595700
F	5.56145800	3.52376900	0.69301100
C	8.20119300	0.68943400	-3.32279500
F	8.37433700	-0.62907400	-3.48319300
F	9.23517100	1.14721600	-2.60068200
F	8.30328400	1.24817600	-4.54055000
H	0.56509300	-4.69930600	-3.41666900
H	-1.15960900	-1.18430500	-1.63582600
H	-1.47716600	-5.98450600	-2.84315100
H	-3.22074300	-2.77303500	0.46899600
H	-5.37142200	-3.65088600	-0.37154200
H	-3.68067200	-5.42563500	-0.41903800
H	-4.28576500	-5.46399900	-2.08728700
H	-6.65191800	-1.97597200	-1.73121700
H	-5.91452700	-1.54289700	-3.29265500
H	0.84344000	-1.12766000	-2.34173800
H	2.63401600	-0.03373700	-1.90399500
H	3.45854600	1.92698900	-1.16413100
H	7.59805000	2.88042900	-1.85870900
H	5.95846100	-0.79855800	-3.37958200
H	-4.89218000	4.93880900	1.50682100
H	-2.81687900	4.95748800	-2.24248400
H	-2.13373100	2.82789500	-3.23847500
H	-2.81009200	1.28978700	-2.69532100
H	-3.82886900	2.44224300	-3.57048800
H	-6.12807200	1.59727500	1.20245300

H	-4.73818900	1.73014300	2.30050600
H	-5.97801300	3.00939600	2.26177600
H	-4.19938400	7.02731200	0.58056100
H	-2.54155600	6.84377600	-0.01136900
H	-3.85495000	7.10458600	-1.15857500
C	-3.14147000	-2.95870500	3.59069700
C	-4.52604600	-3.10810100	3.53791900
C	-2.51416100	-1.83170500	2.99949400
C	-3.27727400	-1.02408400	2.16019300
C	-4.67058300	-1.16401700	2.11944300
C	-5.28920300	-2.17870300	2.83250500
C	-1.08045900	-1.54470800	3.17766700
C	-0.42066800	-1.74515200	4.41115000
C	0.96655600	-1.61548400	4.47368000
C	1.69197800	-1.29356100	3.33814600
C	1.03612800	-0.96609000	2.15834700
C	-0.36293000	-1.00167100	2.08668700
C	-2.60156400	0.05717600	1.28634800
O	-2.57276500	1.23317200	1.75419400
O	-2.32103000	-3.87691600	4.14901000
C	-2.90462500	-4.91962500	4.91082300
C	-1.14364600	-1.99917100	5.71348800
N	-1.09975000	-0.52161000	0.94788400
C	0.36434300	1.99805300	0.82631100
C	1.72930100	2.07368900	1.10285000
C	2.24522700	3.25302900	1.62387300
C	1.41985600	4.35823200	1.85948500
C	0.05612600	4.25226900	1.56910300
C	-0.48399900	3.08062700	1.04979000
C	2.00915400	5.63128600	2.40251000
S	-0.27757300	0.57633000	-0.03682100
O	-1.26506600	1.05451400	-0.99811400
O	0.89679600	-0.11193400	-0.61263900
H	-5.01079000	-3.94491800	4.02612500
H	-5.27387500	-0.49396200	1.51489500
H	-6.36985900	-2.28030900	2.80867200
H	1.46993800	-1.77573100	5.42346500
H	2.77667000	-1.25225500	3.36766800
H	1.62895800	-0.70592300	1.29639300
H	-2.07254800	-5.49035100	5.32437900
H	-3.51654700	-4.51740300	5.72659800
H	-3.51622600	-5.57737900	4.28326500
H	-0.70366700	-1.36178300	6.48711600
H	-2.20942900	-1.76738600	5.65436000

H	-1.03987600	-3.03801100	6.03963900
H	2.38830400	1.24058200	0.88671900
H	3.30940900	3.32522300	1.82785800
H	-0.59554100	5.10468000	1.74102300
H	-1.54240400	2.99130800	0.82944100
H	1.28860300	6.45186100	2.36636000
H	2.32360200	5.49880800	3.44357700
H	2.89569600	5.91894900	1.82779500

2.6 General experiment procedures

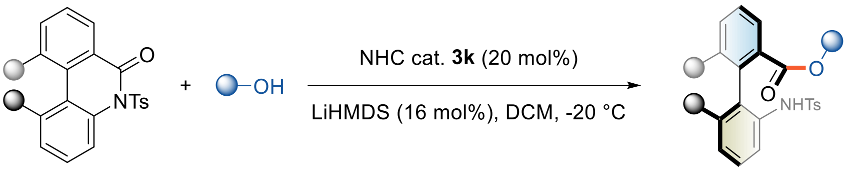

1 2 4

General procedure A for product 4 or 5

The catalyst precursor **3k** (7.4 mg, 0.01 mmol, 20 mol%) and cyclic biaryl lactam **1** (0.05 mmol, 1.0 eq.) were mixed in anhydrous DCM (0.5 mL, 0.1 M) in a 20-mL test tube. The mixture was degassed and back-filled with argon (3x) before adding LiHMDS (1.0 M in THF, 8 μL, 16 mol%). The test tube was stirred at -20 °C for 10 min. Alcohol **2** (0.1 mmol, 2.0 eq.) was directly added, and the mixture was stirred at -20 °C for 15 h. (Caution: If the reaction is incomplete, the reaction period was prolonged for another 5 hours at 0 °C.) Upon completely consuming **1**, the reaction was purified by flash column chromatography (eluent: PE/EA = 10/1 to 4/1) to afford product **4** or **5**. The ee was determined by chiral HPLC.

Racemic samples were prepared by the following procedure: cyclic biaryl lactam (0.05 mmol, 1.0 eq.) and alcohol (0.1 mmol, 2.0 eq.) were dissolved in 1.0 mL of DCM. Then DBU (0.2 mmol, 2.0 eq.) was added directly. The reaction mixture was stirred at r.t. for 8 h and purified by flash column chromatography.

General procedure B for product 5d, 5f, 5i, 5l, 5k

The catalyst precursor **3k** (7.4 mg, 0.01 mmol, 20 mol%) and cyclic biaryl lactam **1** (0.05 mmol, 1.0 eq.) were added in a 20-mL test tube. The tube was transferred to the glove box. KOAc (10.0 mg, 0.1 mmol, 2.0 eq.) and anhydrous DCM (0.5 mL, 0.1 M) were added sequentially. Then, the test tube was sealed with a rubber septum and stirred at -20 °C for 10 min. Alcohol **2** (0.1 mmol, 2.0 eq.) was directly added, and the mixture was stirred at -20 °C for 15 h. Upon complete consumption of **1**, the reaction was purified by flash column chromatography (eluent: PE/EA = 10/1 to 4/1) to afford product **5**. The ee was determined by chiral HPLC.

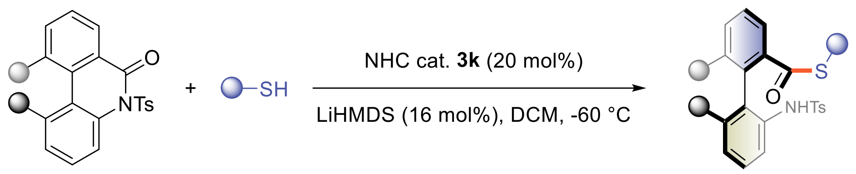

General procedure C for product 6a-6c, 6j, 6n, 6o

The catalyst precursor **3k** (7.4 mg, 0.01 mmol, 20 mol%) and cyclic biaryl lactam **1** (0.05 mmol, 1.0 eq.) were mixed in anhydrous DCM (0.5 mL, 0.1 M) in a 20-mL test tube. The mixture was degassed and back-filled with argon (3x) before adding LiHMDS (1.0 M in THF, 8 μL, 16 mol%). The test tube was stirred at -60 °C for 10 min. Thiol **2** (0.1 mmol, 2.0 eq.) was directly added, and the mixture was stirred at -60 °C for 15 h. Upon complete consumption of **1**, the reaction was purified by flash column chromatography (eluent: PE/EA = 10/1 to 4/1) to afford product **6**. The ee was determined by chiral HPLC.

General procedure D for product 6d-6g, 6k, 6m, 6q

The catalyst precursor **3k** (14.8 mg, 0.02 mmol, 20 mol%) and 4Å molecular sieves (100 mg) were mixed in anhydrous DCM (1.0 mL, 0.1 M) in an 8-mL test tube in the glovebox. LiHMDS (1.0 M in THF, 16 μL, 0.16 eq.) was added. After stirring for 10 min, cyclic biaryl lactam **1** (0.1 mmol, 1.0 eq.) was added, and the test tube was sealed with a rubber septum and taken out of the glovebox. The reaction mixture was cooled to -60 °C, and then thiol **2** (0.2 mmol, 2.0 eq.) was added via a micro-syringe. The resulting mixture was stirred at -60 °C for 1-4 days. Upon completion of **1**, the reaction mixture was quickly filtered through a plug of silica gel (eluted with pre-cooled dichloromethane) and then concentrated. The residue was purified by flash column chromatography (eluent: PE/DCM = 3/1 to 2/1) to afford the desired product. The ee was determined by chiral HPLC.

For product **6q**, catalyst precursor **3e** (20 mol%) combined with potassium pivalate/18-crown-6 (1:1 ratio, 16 mol%) was employed instead of **3k** with LiHMDS.

General procedure E for product 6h, 6i, 6l, 6p, 6r, 6s, 6t-6w

The catalyst precursor **3k** (14.8 mg, 0.02 mmol, 20 mol%) and 4Å molecular sieves (100 mg) were mixed in anhydrous DCM (1.0 mL, 0.1 M) in an 8-mL test tube in the glovebox. KHMDS (1.0 M in THF, 16 μL, 0.16 eq.) was added. After stirring for 10 min, cyclic biaryl lactam **1** (0.1 mmol, 1.0 eq.) was added, and the test tube was sealed with a rubber septum and taken out of the glovebox. Thiol **2** (0.2 mmol, 2.0 eq.) was

added via a micro-syringe, and the resulting mixture was stirred at room temperature for 24 h. Upon completion of **1**, the reaction mixture was directly purified by flash column chromatography (eluent: PE/DCM = 3/1 to 2/1) to afford the desired product. The ee was determined by chiral HPLC.

For **6t-6w**, 1.0 eq. thiol **2** was employed to avoid reversible lactamation.

2.7 Characterization of products

benzyl (*S*)-6-methoxy-2'-methyl-6'-((4-methylphenyl)sulfonamido)-[1,1'-biphenyl]-2-carboxylate (**4a)**

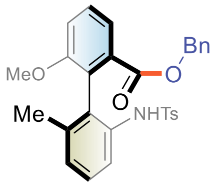

4a

White solid, 24.8 mg, 99% yield, 95% ee, **m.p.** 81-82 °C. **^{1}H NMR** (400 MHz, $CDCl_3$) δ 7.59 (dd, *J* = 7.9, 5.6 Hz, 3H), 7.46 (t, *J* = 8.1 Hz, 1H), 7.38 (d, *J* = 8.2 Hz, 1H), 7.32 – 7.22 (m, 3H), 7.15 (d, *J* = 8.0 Hz, 2H), 7.13 – 7.00 (m, 4H), 6.83 (d, *J* = 7.6 Hz, 1H), 6.18 (s, 1H), 4.97 (d, *J* = 12.2 Hz, 1H), 4.80 (d, *J* = 12.3 Hz, 1H), 3.54 (s, 3H), 2.34 (s, 3H), 1.72 (s, 3H). **^{13}C NMR** (101 MHz, $CDCl_3$) δ 166.5, 157.1, 143.4, 137.5, 137.3, 135.3, 134.7, 132.8, 130.1, 129.5, 128.5, 128.4, 128.3, 128.1, 127.6, 127.5, 125.8, 124.9, 123.0, 116.8, 114.9, 67.1, 56.1, 21.6, 20.2. **HRMS** (ESI-TOF) $[M+H]^+$ calculated for $[C_{29}H_{28}NO_5S]^+$ 502.1683, found 502.1684. **HPLC** (Chiralpak-AS-H column, Hexane/^{i}PrOH = 95/5, flow rate: 1.0 mL/min): t_{major} = 21.300 min; t_{minor} = 24.717 min. **Specific Rotation** $[\alpha]_D^{26}$ = +5.0 (c = 0.5 in $CHCl_3$).

3-methoxybenzyl (*S*)-6-methoxy-2'-methyl-6'-((4-methylphenyl)sulfonamido)-[1,1'-biphenyl]-2-carboxylate (**4b**)

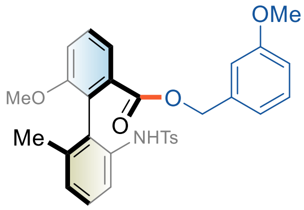

4b

White solid, 26.3 mg, 99% yield, 96% ee, **m.p.** 63-64 °C. **^{1}H NMR** (400 MHz, $CDCl_3$) δ 7.67 – 7.59 (m, 3H), 7.49 (t, *J* = 8.0 Hz, 1H), 7.40 (d, *J* = 8.4 Hz, 1H), 7.26 – 7.16 (m, 3H), 7.16 – 7.08 (m, 2H), 6.89 – 6.83 (m, 2H), 6.74 – 6.66 (m, 2H), 6.18 (s, 1H), 4.98 (d, *J* = 12.2 Hz, 1H), 4.81 (d, *J* = 12.1 Hz, 1H), 3.82 (s, 3H), 3.58 (s, 3H), 2.38 (s, 3H), 1.76 (s, 3H). **^{13}C NMR** (101 MHz, $CDCl_3$) δ 166.3, 159.6, 157.1, 143.3, 137.3, 137.2, 136.7, 134.5, 132.6, 129.9, 129.5, 129.4, 128.0, 127.4, 127.4, 125.6, 124.9, 122.9, 120.7, 116.6, 114.7, 113.9, 113.8, 66.8, 56.0, 55.3, 21.5, 20.1. **HRMS** (ESI-TOF) $[M+H]^+$ calculated for $[C_{30}H_{30}NO_6S]^+$ 532.1788, found 532.1792. **HPLC** (Chiralpak-IA-H

column, Hexane/^{i}PrOH = 90/10, flow rate: 1.0 mL/min): t_{minor} = 37.917 min; t_{major} = 43.087 min. **Specific Rotation** $[\alpha]_D^{26}$ = +13.0 (c = 0.1 in $CHCl_3$).

4-nitrobenzyl (*S*)-6-methoxy-2'-methyl-6'-((4-methylphenyl)sulfonamido)-[1,1'-biphenyl]-2-carboxylate (**4c**)

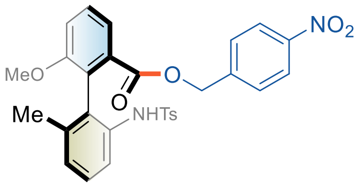

4c

Colorless oil, 27.0 mg, 99% yield, 86% ee. **^{1}H NMR** (400 MHz, $CDCl_3$) δ 8.17 – 8.03 (m, 2H), 7.71 – 7.60 (m, 3H), 7.60 – 7.48 (m, 1H), 7.34 (d, *J* = 8.2 Hz, 1H), 7.25 – 7.02 (m, 6H), 6.86 (d, *J* = 7.5 Hz, 1H), 6.18 (s, 1H), 5.09 (d, *J* = 13.2 Hz, 1H), 4.95 (d, *J* = 13.2 Hz, 1H), 3.60 (s, 3H), 2.38 (s, 3H), 1.77 (s, 3H). **^{13}C NMR** (101 MHz, $CDCl_3$) δ 166.5, 157.1, 143.6, 142.3, 137.5, 137.1, 134.8, 132.4, 130.2, 129.5, 128.6, 128.1, 127.4, 127.1, 127.0, 125.5, 124.5, 123.6, 123.0, 116.1, 115.0, 65.6, 56.0, 21.5, 20.2. **HRMS** (ESI-TOF) $[M+H]^+$ calculated for $[C_{29}H_{27}N_2O_7S]^+$ 547.1534, found 547.1538. **HPLC** (Chiralpak-IA-H column, Hexane/^{i}PrOH = 70/30, flow rate: 1.0 mL/min): t_{minor} = 23.320 min; t_{major} = 25.867 min. **Specific Rotation** $[\alpha]_D^{26}$ = +4.8 (c = 0.01 in $CHCl_3$).

2-iodobenzyl (*R*)-2',4,6-trimethyl-6'-((4-methylphenyl)sulfonamido)-[1,1'-biphenyl]-2-carboxylate (**4d**)

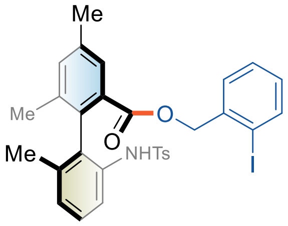

4d

Colorless oil, 30.9 mg, 99% yield, 90% ee. **^{1}H NMR** (400 MHz, $CDCl_3$) δ 7.88 – 7.75 (m, 2H), 7.72 – 7.60 (m, 2H), 7.38 (d, *J* = 8.3 Hz, 1H), 7.32 (s, 1H), 7.30 – 7.23 (m, 1H), 7.20 (d, *J* = 8.1 Hz, 2H), 7.09 (t, *J* = 7.9 Hz, 1H), 7.06 – 6.97 (m, 1H), 6.92 (d, *J* = 7.6, 1.7 Hz, 1H), 6.86 (d, *J* = 7.6 Hz, 1H), 6.11 (s, 1H), 5.02 (d, *J* = 13.1 Hz, 1H), 4.90 (d, *J* = 13.1 Hz, 1H), 2.46 (s, 3H), 2.38 (s, 3H), 1.79 (s, 3H), 1.66 (s, 3H). **^{13}C NMR** (101 MHz, $CDCl_3$) δ 166.5, 143.8, 139.2, 138.7, 138.4, 137.9, 137.0, 136.8, 135.7, 134.2, 132.5, 130.7, 129.7, 129.6, 129.6, 129.5, 129.2, 128.3, 127.9, 127.4, 125.5,

115.4, 98.1, 70.7, 21.6, 21.1, 20.2, 19.4. **HRMS** (ESI-TOF) $[M+H]^+$ calculated for $[C_{30}H_{29}INO_4S]^+$ 626.0857, found 626.0860. **HPLC** (Chiralpak-IC-H column, Hexane/^{i}PrOH = 70/30, flow rate: 1.0 mL/min): t_{major} = 13.270 min; t_{minor} = 21.593 min. **Specific Rotation** $[\alpha]_D^{26}$ = –12.1 (c = 0.03 in $CHCl_3$).

furan-3-ylmethyl (*S*)-6-methoxy-2'-methyl-6'-((4-methylphenyl)sulfonamido)-[1,1'-biphenyl]-2-carboxylate (**4e**)

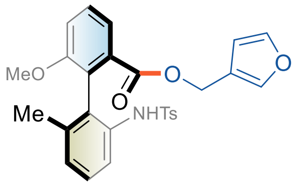

4e

White oil, 24.3 mg, 99% yield, 92% ee. **^{1}H NMR** (400 MHz, $CDCl_3$) δ 7.67 – 7.54 (m, 3H), 7.49 (t, *J* = 8.0 Hz, 1H), 7.41 (d, *J* = 8.1 Hz, 1H), 7.33 (t, *J* = 1.7 Hz, 1H), 7.22 (t, *J* = 8.5 Hz, 3H), 7.18 – 7.09 (m, 2H), 6.89 (d, *J* = 7.5 Hz, 1H), 6.20 (d, *J* = 5.2 Hz, 2H), 4.87 (d, *J* = 12.3 Hz, 1H), 4.69 (d, *J* = 12.3 Hz, 1H), 3.58 (s, 3H), 2.40 (s, 3H), 1.75 (s, 3H). **^{13}C NMR** (101 MHz, $CDCl_3$) δ 166.5, 157.0, 143.3, 143.1, 141.7, 137.3, 137.2, 134.6, 132.8, 130.0, 129.4, 128.0, 127.4, 127.4, 125.6, 124.7, 122.7, 119.6, 116.5, 114.7, 110.7, 58.2, 56.0, 21.5, 20.1. **HRMS** (ESI-TOF) $[M+H]^+$ calculated for $[C_{27}H_{26}NO_6S]^+$ 492.1475, found 492.1477. **HPLC** (Chiralpak-IA-H column, Hexane/^{i}PrOH = 70/30, flow rate: 1.0 mL/min): t_{minor} = 9.547 min; t_{major} = 10.563 min. **Specific Rotation** $[\alpha]_D^{26}$ = +11.7 (c = 0.08 in $CHCl_3$).

thiophen-2-ylmethyl (*R*)-2',4,6-trimethyl-6'-((4-methylphenyl)sulfonamido)-[1,1'-biphenyl]-2-carboxylate (**4f**)

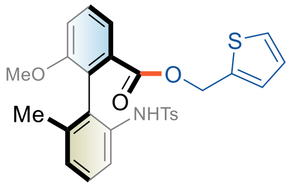

4f

White oil, 23.6 mg, 93% yield, 96% ee. **^{1}H NMR** (400 MHz, $CDCl_3$) δ 7.79 – 7.60 (m, 3H), 7.39 (d, *J* = 8.2 Hz, 1H), 7.34 – 7.21 (m, 4H), 7.11 (t, *J* = 7.9 Hz, 1H), 6.94 (dd, *J* = 5.1, 3.5 Hz, 1H), 6.90 – 6.82 (m, 2H), 6.09 (s, 1H), 5.15 (d, *J* = 12.7 Hz, 1H), 4.93 (d, *J* = 12.8 Hz, 1H), 2.44 (s, 3H), 2.42 (s, 3H), 1.72 (s, 3H), 1.65 (s, 3H). **^{13}C NMR** (101 MHz, $CDCl_3$) δ 166.7, 143.7, 138.6, 138.3, 137.2, 137.1, 136.7, 135.5, 134.2,

132.5, 130.9, 129.6, 129.2, 128.4, 127.9, 127.4, 126.8, 126.7, 125.5, 115.5, 60.7, 21.6, 21.0, 20.0, 19.3. **HRMS** (ESI-TOF) $[M+H]^+$ calculated for $[C_{27}H_{26}NO_5S_2]^+$ 508.1247, found 508.1251. **HPLC** (Chiralpak-IA-H column, Hexane/^{i}PrOH = 70/30, flow rate: 1.0 mL/min): t_{major} = 11.380 min; t_{minor} = 10.130 min. **Specific Rotation** $[\alpha]_D^{25}$ = +20.8 (c = 0.8 in $CHCl_3$).

ethyl (*S*)-6-methoxy-2'-methyl-6'-((4-methylphenyl)sulfonamido)-[1,1'-biphenyl]-2-carboxylate (**4g**)

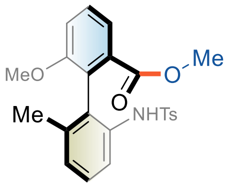

4g

White solid, 21.0 mg, 99% yield, 98% ee, **m.p.** 161-162 °C. **^{1}H NMR** (400 MHz, $CDCl_3$) δ 7.65 – 7.58 (m, 3H), 7.54 – 7.46 (m, 2H), 7.25 – 7.13 (m, 4H), 6.99 (d, *J* = 7.5 Hz, 1H), 6.24 (s, 1H), 3.62 (s, 3H), 3.48 (s, 3H), 2.41 (s, 3H), 1.83 (s, 3H). **^{13}C NMR** (101 MHz, $CDCl_3$) δ 166.9, 157.1, 143.3, 137.3, 137.2, 134.6, 132.6, 130.0, 129.4, 128.0, 127.6, 127.4, 125.6, 125.0, 122.7, 116.7, 114.7, 56.0, 52.1, 21.6, 20.2. **HRMS** (ESI-TOF) $[M+H]^+$ calculated for $[C_{23}H_{24}NO_5S]^+$ 426.1370, found 426.1370. **HPLC** (Chiralpak-AS-H column, Hexane/^{i}PrOH = 95/5, flow rate: 1.0 mL/min): t_{major} = 23.070 min; t_{minor} = 28.503 min. **Specific Rotation** $[\alpha]_D^{26}$ = +40.0 (c = 0.06 in $CHCl_3$).

methyl (*S*)-6-methoxy-2'-methyl-6'-((4-methylphenyl)sulfonamido)-[1,1'-biphenyl]-2-carboxylate (**4h**)

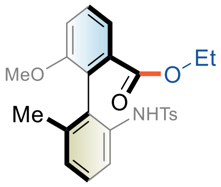

4h

White solid, 21.7 mg, 99% yield, 98% ee, **m.p.** 114-115 °C. **^{1}H NMR** (400 MHz, $CDCl_3$) δ 7.61 (t, *J* = 8.7 Hz, 3H), 7.54 – 7.41 (m, 2H), 7.25 – 7.09 (m, 4H), 6.96 (d, *J* = 7.5 Hz, 1H), 6.22 (s, 1H), 4.08 – 3.89 (m, 1H), 3.89 – 3.73 (m, 1H), 3.59 (s, 3H), 2.40 (s, 3H), 1.82 (s, 3H), 0.85 (t, *J* = 7.1 Hz, 3H). **^{13}C NMR** (101 MHz, $CDCl_3$) δ 166.8, 157.0, 143.3, 137.4, 137.2, 134.7, 133.2, 129.9, 129.4, 127.9, 127.6, 127.4, 125.5, 124.6, 122.7, 116.4, 114.5, 60.9, 56.0, 21.5, 20.1, 13.4. **HRMS** (ESI-TOF) $[M+H]^+$ calculated for

$[C_{24}H_{26}NO_5S]^+$ 440.1526, found 440.1525. **HPLC** (Chiralpak-IA-H column, Hexane/^{i}PrOH = 70/30, flow rate: 1.0 mL/min): t_{major} = 21.337 min; t_{minor} = 27.120 min. **Specific Rotation** $[\alpha]_D^{26}$ = +1.7 (c = 0.08 in $CHCl_3$).

cyclohexylmethyl (*S*)-6-methoxy-2'-methyl-6'-((4-methylphenyl)sulfonamido)-[1,1'-biphenyl]-2-carboxylate (**4i**)

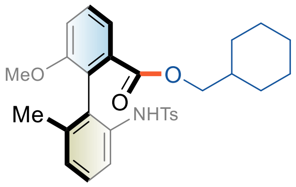

4i

Colorless oil, 25.1 mg, 99% yield, 96% ee. **^{1}H NMR** (500 MHz, $CDCl_3$) δ 7.67 – 7.56 (m, 3H), 7.53 – 7.41 (m, 2H), 7.25 – 7.13 (m, 3H), 7.10 (dd, *J* = 8.3, 1.1 Hz, 1H), 6.94 (d, *J* = 7.5 Hz, 1H), 6.21 (s, 1H), 3.76 (dd, *J* = 10.7, 6.7 Hz, 1H), 3.64 (dd, *J* = 10.7, 6.5 Hz, 1H), 3.54 (s, 3H), 2.38 (s, 3H), 1.82 (s, 3H), 1.70 – 1.56 (m, 4H), 1.52 – 1.39 (m, 2H), 1.17 – 1.05 (m, 3H), 0.82 – 0.67 (m, 2H). **^{13}C NMR** (126 MHz, $CDCl_3$) δ 166.9, 157.0, 143.3, 137.4, 137.2, 134.7, 133.2, 129.9, 129.4, 127.9, 127.4, 127.4, 125.5, 124.5, 122.8, 116.2, 114.5, 70.6, 55.9, 36.8, 29.6, 29.5, 26.2, 25.6, 21.5, 20.2. **HRMS** (ESI-TOF) $[M+H]^+$ calculated for $[C_{29}H_{34}NO_5S]^+$ 508.2152, found 508.2154. **HPLC** (Chiralpak-AS-H column, Hexane/^{i}PrOH = 95/5, flow rate: 1.0 mL/min): t_{major} = 24.133 min; t_{minor} = 29.347 min. **Specific Rotation** $[\alpha]_D^{26}$ = –2.0 (c = 0.1 in $CHCl_3$).

3-phenylpropyl (*S*)-6-methoxy-2'-methyl-6'-((4-methylphenyl)sulfonamido)-[1,1'-biphenyl]-2-carboxylate (**4j**)

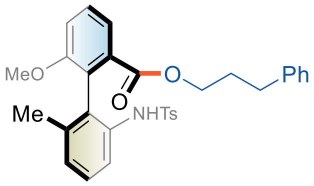

4j

Colorless oil, 26.2 mg, 99% yield, 96% ee. **^{1}H NMR** (400 MHz, $CDCl_3$) δ 7.69 – 7.58 (m, 3H), 7.58 – 7.41 (m, 2H), 7.35 – 7.26 (m, 2H), 7.26 – 7.05 (m, 7H), 6.97 (d, *J* = 7.5 Hz, 1H), 6.24 (s, 1H), 3.97 (dt, *J* = 10.9, 6.6 Hz, 1H), 3.83 (dt, *J* = 10.9, 6.6 Hz, 1H), 3.59 (s, 3H), 2.46 (dd, *J* = 8.7, 6.6 Hz, 2H), 2.38 (s, 3H), 1.85 (s, 3H), 1.75 – 1.50 (m, 2H). **^{13}C NMR** (101 MHz, $CDCl_3$) δ 166.8, 157.0, 143.4, 141.1, 137.4, 137.2, 134.7, 133.1, 130.0, 129.4, 128.4, 128.4, 128.0, 127.5, 127.4, 126.0, 125.5, 124.5, 122.8, 116.4,

114.6, 64.6, 56.0, 31.9, 29.5, 21.5, 20.2. **HRMS** (ESI-TOF) $[M+H]^+$ calculated for $[C_{31}H_{32}NO_5S]^+$ 530.1996, found 530.2000. **HPLC** (Chiralpak-IA-H column, Hexane/^{i}PrOH = 70/30, flow rate: 1.0 mL/min): t_{minor} = 9.620 min; t_{major} = 10.617 min. **Specific Rotation** $[\alpha]_D^{26}$ = +8.9 (c = 0.02 in $CHCl_3$).

2-cyclopropylethyl (*S*)-6-methoxy-2'-methyl-6'-((4-methylphenyl)sulfonamido)-[1,1'-biphenyl]-2-carboxylate (**4k**)

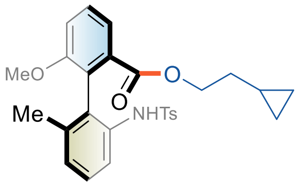

4k

White oil, 21.8 mg, 91% yield, 98% ee. **^{1}H NMR** (400 MHz, $CDCl_3$) δ 7.71 – 7.56 (m, 3H), 7.56 – 7.38 (m, 2H), 7.26 – 7.08 (m, 4H), 6.95 (d, *J* = 8.5 Hz, 1H), 6.22 (s, 1H), 4.06 – 3.94 (m, 1H), 3.92 – 3.81 (m, 1H), 3.58 (s, 3H), 2.40 (s, 3H), 1.82 (s, 3H), 1.15 (qd, *J* = 7.0, 3.3 Hz, 2H), 0.57 – 0.43 (m, 1H), 0.43 – 0.35 (m, 2H), 0.01 – -0.08 (m, 2H). **^{13}C NMR** (101 MHz, $CDCl_3$) δ 166.8, 157.0, 143.3, 137.4, 137.2, 134.7, 133.2, 130.0, 129.4, 127.9, 127.4, 125.5, 124.6, 122.7, 116.3, 114.5, 65.3, 56.0, 33.0, 21.5, 20.2, 7.4, 4.1. **HRMS** (ESI-TOF) $[M+H]^+$ calculated for $[C_{27}H_{30}NO_5S]^+$ 480.1839, found 480.1841. **HPLC** (Chiralpak-IC-H column, Hexane/^{i}PrOH = 70/30, flow rate: 1.0 mL/min): t_{major} = 19.547 min; t_{minor} = 23.710 min. **Specific Rotation** $[\alpha]_D^{26}$ = +8.9 (c = 0.04 in $CHCl_3$).

3-bromopropyl (*S*)-6-methoxy-2'-methyl-6'-((4-methylphenyl)sulfonamido)-[1,1'-biphenyl]-2-carboxylate (**4l**)

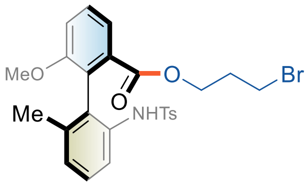

4l

Colorless oil, 26.0 mg, 98% yield, 94% ee. **^{1}H NMR** (400 MHz, $CDCl_3$) δ 7.68 – 7.60 (m, 3H), 7.53 (t, *J* = 8.1 Hz, 1H), 7.44 (d, *J* = 8.2 Hz, 1H), 7.27 – 7.12 (m, 4H), 7.02 – 6.94 (m, 1H), 6.17 (s, 1H), 4.07 (dt, *J* = 11.7, 6.0 Hz, 1H), 3.92 (dt, *J* = 11.5, 5.9 Hz, 1H), 3.60 (s, 3H), 3.09 (dt, *J* = 11.6, 6.6 Hz, 2H), 2.41 (s, 3H), 1.83 (s, 3H), 1.81 – 1.68 (m, 2H). **^{13}C NMR** (101 MHz, $CDCl_3$) δ 166.7, 157.0, 143.5, 137.4, 137.1, 134.7, 132.8,

130.1, 129.5, 128.1, 127.4, 125.5, 124.4, 123.1, 116.1, 114.8, 63.1, 56.0, 31.2, 29.6, 21.6, 20.2. **HRMS** (ESI-TOF) $[M+H]^+$ calculated for $[C_{25}H_{26}BrNO_5S]^+$ 532.0788, found 532.0791. **HPLC** (Chiralpak-AS-H column, Hexane/^{i}PrOH = 90/10, flow rate: 1.0 mL/min): t_{minor} = 35.940 min; t_{major} = 44.623 min. **Specific Rotation** $[\alpha]_D^{26}$ = +4.0 (c = 0.05 in $CHCl_3$).

3-iodopropyl (*S*)-6-methoxy-2'-methyl-6'-((4-methylphenyl)sulfonamido)-[1,1'-biphenyl]-2-carboxylate (**4m**)

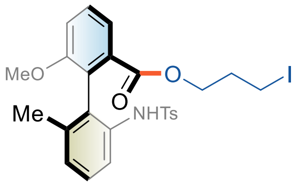

4m

Colorless oil, 28.6 mg, 99% yield, 96% ee. **^{1}H NMR** (400 MHz, $CDCl_3$) δ 7.69 – 7.60 (m, 3H), 7.52 (t, *J* = 8.1 Hz, 1H), 7.44 (d, *J* = 8.2 Hz, 1H), 7.22 (dd, *J* = 15.4, 7.9 Hz, 3H), 7.15 (dd, *J* = 8.3, 1.1 Hz, 1H), 6.98 (d, *J* = 7.6 Hz, 1H), 6.18 (s, 1H), 4.00 (dt, *J* = 11.6, 5.9 Hz, 1H), 3.85 (dt, *J* = 11.4, 5.9 Hz, 1H), 3.59 (s, 3H), 2.83 (qt, *J* = 10.0, 6.9 Hz, 2H), 2.41 (s, 3H), 1.81 (s, 3H), 1.76 – 1.66 (m, 2H). **^{13}C NMR** (101 MHz, $CDCl_3$) δ 166.8, 157.0, 143.5, 137.4, 137.1, 134.7, 132.7, 130.1, 129.5, 128.2, 127.4, 125.5, 124.4, 123.1, 116.1, 114.8, 65.1, 56.0, 31.9, 21.6, 20.2, 1.9. **HRMS** (ESI-TOF) $[M+H]^+$ calculated for $[C_{25}H_{26}INO_5SNa]^+$ 602.0469, found 602.0471. **HPLC** (Chiralpak-IC-H column, Hexane/^{i}PrOH = 70/30, flow rate: 1.0 mL/min): t_{minor} = 26.027 min; t_{major} = 21.837 min. **Specific Rotation** $[\alpha]_D^{25}$ = +8.2 (c = 1.0 in $CHCl_3$).

3-methoxy-3-oxopropyl (*S*)-6-methoxy-2'-methyl-6'-((4-methylphenyl)sulfonamido)-[1,1'-biphenyl]-2-carboxylate (**4n**)

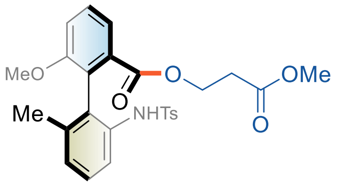

4n

Colorless oil, 24.6 mg, 99% yield, 86% ee. **^{1}H NMR** (400 MHz, $CDCl_3$) δ 7.62 (d, *J* = 8.3 Hz, 3H), 7.58 – 7.39 (m, 2H), 7.25 – 7.08 (m, 4H), 6.95 (d, *J* = 7.5 Hz, 1H), 6.17 (s, 1H), 4.28 – 4.10 (m, 1H), 4.07 – 3.94 (m, 1H), 3.69 (s, 3H), 3.58 (s, 3H), 2.40 (s, 3H), 2.22 – 2.13 (m, 2H), 1.80 (s, 3H). **^{13}C NMR** (101 MHz, $CDCl_3$) δ 170.8, 166.2,

157.0, 143.4, 137.5, 137.1, 134.6, 132.3, 130.0, 129.4, 128.0, 127.7, 127.4, 125.5, 124.7, 123.1, 116.4, 114.9, 60.1, 56.0, 51.8, 32.6, 21.5, 20.1. **HRMS** (ESI-TOF) $[M+H]^+$ calculated for $[C_{26}H_{28}NO_7S]^+$ 498.1581, found 498.1584. **HPLC** (Chiralpak-IA-H column, Hexane/^{i}PrOH = 70/30, flow rate: 1.0 mL/min): t_{minor} = 13.283 min; t_{major} = 15.093 min. **Specific Rotation** $[\alpha]_D^{26}$ = –3.3 (c = 0.04 in $CHCl_3$).

4,4,4-trifluorobutyl (*S*)-6-methoxy-2'-methyl-6'-((4-methylphenyl)sulfonamido)-[1,1'-biphenyl]-2-carboxylate (**4o**)

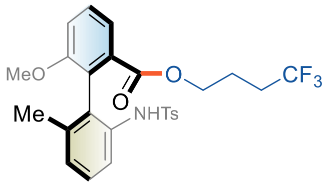

4o

Colorless oil, 25.8 mg, 99% yield, 96% ee. **^{1}H NMR** (400 MHz, $CDCl_3$) δ 7.70 – 7.59 (m, 3H), 7.52 (t, *J* = 8.1 Hz, 1H), 7.43 (d, *J* = 8.2 Hz, 1H), 7.27 – 7.11 (m, 4H), 6.97 (d, *J* = 7.5 Hz, 1H), 6.17 (s, 1H), 4.02 (dt, *J* = 11.8, 6.2 Hz, 1H), 3.84 (dt, *J* = 11.6, 6.2 Hz, 1H), 3.59 (s, 3H), 2.40 (s, 3H), 1.83 (s, 3H), 1.82 – 1.69 (m, 2H), 1.57 – 1.43 (m, 2H). **^{13}C NMR** (101 MHz, $CDCl_3$) δ 166.8, 157.0, 143.5, 137.4, 137.2, 132.7, 130.1, 129.5, 128.2, 127.4, 127.3, 125.5, 125.4, 123.0, 116.1, 114.8, 63.6, 56.0, 30.44 (q, *J* = 29.3 Hz), 21.5, 21.0 (q, *J* = 3.0 Hz), 20.1. **^{19}F NMR** (376 MHz, $CDCl_3$) δ -66.4. **HRMS** (ESI-TOF) $[M+H]^+$ calculated for $[C_{26}H_{27}F_3NO_5S]^+$ 522.1557, found 522.1561. **HPLC** (Chiralpak-IA-H column, Hexane/^{i}PrOH = 70/30, flow rate: 1.0 mL/min): t_{major} = 16.007 min; t_{minor} = 19.203 min. **Specific Rotation** $[\alpha]_D^{26}$ = –4.9 (c = 0.07 in $CHCl_3$).

2-(benzyloxy)ethyl (*S*)-6-methoxy-2'-methyl-6'-((4-methylphenyl)sulfonamido)-[1,1'-biphenyl]-2-carboxylate (**4p**)

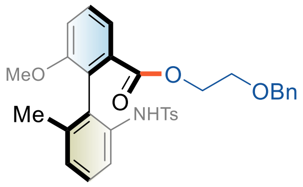

4p

Colorless oil, 27.0 mg, 99% yield, 92% ee. **^{1}H NMR** (400 MHz, $CDCl_3$) δ 7.67 – 7.58 (m, 3H), 7.55 – 7.41 (m, 2H), 7.41 – 7.30 (m, 5H), 7.25 – 7.09 (m, 4H), 6.94 (d, *J* = 7.6 Hz, 1H), 6.23 (s, 1H), 4.48 (d, *J* = 1.9 Hz, 2H), 4.13 (ddd, *J* = 11.8, 6.3, 4.1 Hz, 1H),

3.99 (ddd, J = 11.8, 5.9, 4.1 Hz, 1H), 3.59 (s, 3H), 3.39 – 3.24 (m, 2H), 2.39 (s, 3H), 1.81 (s, 3H). **^{13}C NMR** (101 MHz, $CDCl_3$) δ 166.4, 157.0, 143.3, 137.9, 137.5, 137.2, 134.6, 132.6, 129.9, 129.4, 128.4, 127.9, 127.8, 127.7, 127.7, 127.4, 125.6, 124.9, 122.9, 116.8, 114.7, 73.1, 67.4, 63.9, 56.0, 21.5, 20.2. **HRMS** (ESI-TOF) $[M+H]^+$ calculated for $[C_{31}H_{32}NO_6S]^+$ 546.1945, found 546.1950. **HPLC** (Chiralpak-IA-H column, Hexane/^{i}PrOH = 70/30, flow rate: 1.0 mL/min): t_{minor} = 11.663 min; t_{major} = 13.243 min. **Specific Rotation** $[\alpha]_D^{26}$ = +20.5 (c = 0.03 in $CHCl_3$).

but-3-yn-1-yl (*S*)-6-methoxy-2'-methyl-6'-((4-methylphenyl)sulfonamido)-[1,1'-biphenyl]-2-carboxylate (**4q**)

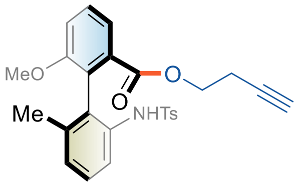

4q

White solid, 23.0 mg, 99% yield, 92% ee, **m.p.** 61-62 °C. **^{1}H NMR** (400 MHz, $CDCl_3$) δ 7.72 – 7.58 (m, 3H), 7.58 – 7.40 (m, 2H), 7.27 – 7.09 (m, 4H), 6.96 (d, J = 7.6 Hz, 1H), 6.18 (s, 1H), 4.09 – 3.91 (m, 1H), 3.89 – 3.71 (m, 1H), 3.61 (s, 3H), 2.41 (s, 3H), 1.99 – 1.92 (m, 3H), 1.82 (s, 3H). **^{13}C NMR** (101 MHz, $CDCl_3$) δ 166.2, 157.0, 143.5, 137.5, 137.1, 134.6, 132.4, 130.1, 129.5, 128.0, 127.5, 127.4, 125.5, 124.7, 123.0, 116.2, 114.9, 79.6, 70.0, 62.4, 56.0, 21.6, 20.2, 18.1. **HRMS** (ESI-TOF) $[M+H]^+$ calculated for $[C_{26}H_{26}NO_5S]^+$ 464.1526, found 464.1530. **HPLC** (Chiralpak-IA-H column, Hexane/^{i}PrOH = 80/20, flow rate: 1.0 mL/min): t_{minor} = 13.243 min; t_{major} = 14.627 min. **Specific Rotation** $[\alpha]_D^{26}$ = –5.3 (c = 0.2 in $CHCl_3$).

cinnamyl (*S*)-6-methoxy-2'-methyl-6'-((4-methylphenyl)sulfonamido)-[1,1'-biphenyl]-2-carboxylate (**4r**)

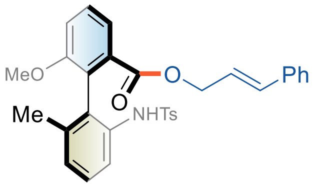

4r

White oil, 26.1 mg, 99% yield, 96% ee. **^{1}H NMR** (400 MHz, $CDCl_3$) δ 7.68 – 7.58 (m, 3H), 7.52 (t, *J* = 8.0 Hz, 1H), 7.43 (d, *J* = 8.1 Hz, 1H), 7.40 – 7.25 (m, 6H), 7.23 – 7.06 (m, 4H), 6.90 (d, *J* = 7.5 Hz, 1H), 6.41 (d, *J* = 15.9 Hz, 1H), 5.86 – 5.66 (m, 1H), 4.59 (ddd, *J* = 12.7, 6.8, 1.3 Hz, 1H), 4.40 (ddd, *J* = 12.7, 6.4, 1.4 Hz, 1H), 3.61 (s, 3H), 2.38 (s, 3H), 1.81 (s, 3H). **^{13}C NMR** (101 MHz, $CDCl_3$) δ 166.5, 157.0, 143.3, 137.4, 137.2, 136.2, 134.7, 134.2, 132.9, 130.0, 129.4, 128.5, 128.0, 128.0, 127.6, 127.4, 126.7, 125.6, 124.8, 122.8, 122.4, 116.6, 114.7, 65.6, 56.0, 21.5, 20.2. **HRMS** (ESI-TOF) $[M+H]^+$ calculated for $[C_{31}H_{30}NO_5S]^+$ 528.1839, found 528.1843. **HPLC** (Chiralpak-IA-H column, Hexane/^{i}PrOH = 70/30, flow rate: 1.0 mL/min): t_{minor} = 10.780 min; t_{major} = 12.710 min. **Specific Rotation** $[\alpha]_D^{26}$ = –1.2 (c = 0.1 in $CHCl_3$).

(*E*)-3,7-dimethylocta-2,6-dien-1-yl (*S*)-6-methoxy-2'-methyl-6'-((4-methylphenyl)sulfonamido)-[1,1'-biphenyl]-2-carboxylate (**4s**)

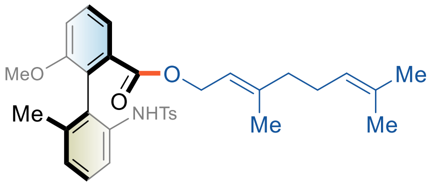

4s

Yellow oil, 27.1 mg, 99% yield, 96% ee. **^{1}H NMR** (400 MHz, $CDCl_3$) δ 7.61 (t, *J* = 8.5 Hz, 3H), 7.48 (q, *J* = 8.0 Hz, 2H), 7.24 – 7.05 (m, 4H), 6.95 (d, *J* = 7.5 Hz, 1H), 6.23 (s, 1H), 5.15 – 5.00 (m, 1H), 4.95 – 4.82 (m, 1H), 4.48 (dd, *J* = 12.4, 7.1 Hz, 1H), 4.27 (dd, *J* = 12.4, 7.0 Hz, 1H), 3.58 (s, 3H), 2.39 (s, 3H), 2.15 – 2.02 (m, 2H), 2.02 – 1.92 (m, 2H), 1.81 (s, 3H), 1.72 (s, 3H), 1.64 (s, 3H), 1.58 (s, 3H). **^{13}C NMR** (101 MHz, $CDCl_3$) δ 166.7, 157.0, 143.3, 142.0, 137.4, 137.2, 134.7, 133.2, 131.8, 129. 9, 129.4, 127.9, 127.7, 127.4, 125.6, 124.7, 123.9, 122.7, 117.6, 116.7, 114.5, 62.0, 56.0, 39.4, 26.3, 25.7, 21.5, 20.2, 17.7, 16.4. **HRMS** (ESI-TOF) $[M+H]^+$ calculated for $[C_{32}H_{37}KNO_5S]^+$ 586.2024, found 586.2028. **HPLC** (Chiralpak-IA-H column, Hexane/^{i}PrOH = 90/10, flow rate: 1.0 mL/min): t_{minor} = 15.163 min; t_{major} = 16.790 min. **Specific Rotation** $[\alpha]_D^{26}$ = +5.9 (c = 0.1 in $CHCl_3$).

3-hydroxy-3-methylbutyl (*S*)-6-methoxy-2'-methyl-6'-((4-methylphenyl)sulfonamido)-[1,1'-biphenyl]-2-carboxylate (**4t**)

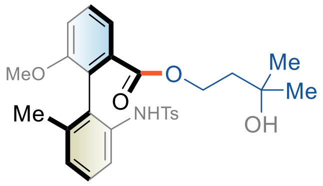

4t

Colorless oil, 22.1 mg, 89% yield, 86% ee. **^{1}H NMR** (400 MHz, $CDCl_3$) δ 7.67 – 7.55 (m, 3H), 7.50 (t, *J* = 8.0 Hz, 1H), 7.43 (d, *J* = 8.2 Hz, 1H), 7.20 (dd, *J* = 16.5, 8.4 Hz, 3H), 7.11 (d, *J* = 8.3 Hz, 1H), 6.97 (d, *J* = 7.5 Hz, 1H), 6.27 (s, 1H), 4.10 (dt, *J* = 11.0, 7.0 Hz, 1H), 4.00 (dt, *J* = 11.0, 7.1 Hz, 1H), 3.58 (s, 3H), 2.40 (s, 3H), 1.82 (s, 3H), 1.45 (t, *J* = 7.1 Hz, 2H), 1.18 (s, 6H). **^{13}C NMR** (101 MHz, $CDCl_3$) δ 166.8, 157.0, 143.4, 137.5, 137.2, 134.7, 133.1, 130.0, 129.4, 128.0, 127.7, 127.4, 125.6, 124.5, 122.6, 116.5, 114.6, 69.7, 62.0, 55.9, 41.0, 29.6, 29.5, 21.6, 20.2. **HRMS** (ESI-TOF) $[M+H]^+$ calculated for $[C_{27}H_{32}NO_6S]^+$ 498.1945, found 498.1947. **HPLC** (Chiralpak-IA-H column, Hexane/^{i}PrOH = 90/10, flow rate: 1.0 mL/min): t_{minor} = 39.617 min; t_{major} = 44.667 min. **Specific Rotation** $[\alpha]_D^{26}$ = +3.6 (c = 0.04 in $CHCl_3$).

oxetan-3-ylmethyl (*S*)-6-(benzyloxy)-2'-methyl-6'-((4-methylphenyl)sulfonamido)-[1,1'-biphenyl]-2-carboxylate (**4u**)

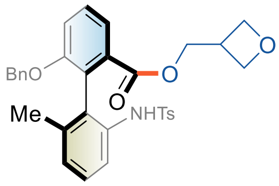

4u

Yellow oil, 27.6 mg, 99% yield, 92% ee. **^{1}H NMR** (400 MHz, $CDCl_3$) δ 7.74 – 7.65 (m, 1H), 7.58 (d, *J* = 8.4 Hz, 2H), 7.49 (q, *J* = 8.0 Hz, 2H), 7.26 – 7.16 (m, 5H), 7.06 – 6.95 (m, 3H), 6.95 – 6.86 (m, 2H), 6.23 (s, 1H), 4.86 (dd, *J* = 12.0, 8.0 Hz, 2H), 4.61 (ddd, *J* = 14.0, 7.8, 6.4 Hz, 2H), 4.34 – 4.22 (m, 1H), 4.18 – 4.01 (m, 3H), 2.78 (tt, *J* = 7.5, 5.7 Hz, 1H), 2.23 (s, 3H), 1.81 (s, 3H). **^{13}C NMR** (101 MHz, $CDCl_3$) δ 166.7, 156.0, 143.5, 137.3, 136.8, 136.3, 134.7, 132.5, 130.1, 129.4, 128.4, 128.1, 127.6, 127.5, 127.3, 125.9, 125.4, 125.4, 123.6, 116.8, 116.0, 74.2, 74.0, 70.0, 66.5, 33.5, 21.4, 20.2. **HRMS** (ESI-TOF) $[M+H]^+$ calculated for $[C_{32}H_{32}NO_6S]^+$ 558.1945, found 558.1949. **HPLC** (Chiralpak-IA-H column, Hexane/^{i}PrOH = 90/10, flow rate: 1.0 mL/min): t_{minor} = 64.907 min; t_{major} = 68.390 min. **Specific Rotation** $[\alpha]_D^{26}$ = +3.1 (c = 0.07 in $CHCl_3$).

(7*R*,11*R*,*E*)-3,7,11,15-tetramethylhexadec-2-en-1-yl (*S*)-6-(benzyloxy)-2'-methyl-6'-((4-methylphenyl)sulfonamido)-[1,1'-biphenyl]-2-carboxylate (**4v**)

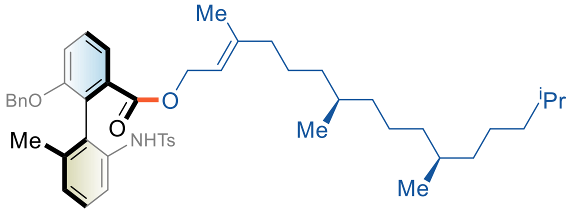

4v

Yellow oil, 37.9 mg, 99% yield, 99% ee. **^{1}H NMR** (400 MHz, $CDCl_3$) δ 7.62 (d, *J* = 1.1 Hz, 1H), 7.56 (d, *J* = 8.3 Hz, 2H), 7.53 – 7.42 (m, 2H), 7.27 – 7.12 (m, 5H), 7.05 – 6.88 (m, 5H), 6.33 (s, 1H), 4.98 – 4.79 (m, 3H), 4.53 (dd, *J* = 12.4, 7.1 Hz, 1H), 4.31 (dd, *J* = 12.3, 7.0 Hz, 1H), 2.23 (s, 3H), 1.99 – 1.87 (m, 2H), 1.81 (s, 3H), 1.63 (s, 1H), 1.60 – 1.50 (m, 4H), 1.47 – 1.20 (m, 13H), 1.13 – 1.00 (m, 4H), 0.97 – 0.80 (m, 12H). **^{13}C NMR** (101 MHz, $CDCl_3$) δ 166.6, 155.9, 143.2, 142.6, 137.4, 137.0, 136.4, 134.7, 133.1, 129.8, 129.3, 128.4, 127.9, 127.8, 127.5, 127.3, 126.0, 125.7, 125.5, 123.2, 117.3, 116.7, 116.5, 70.0, 62.0, 39.8, 39.4, 37.5, 37.4, 37.3, 36.8, 32.8, 32.8, 28.0, 25.0, 24.8, 24.5, 22.8, 22.7, 21.4, 20.2, 19.8, 19.8, 16.4. **HRMS** (ESI-TOF) $[M+Na]^+$ calculated for $[C_{48}H_{63}NO_5SNa]^+$ 788.4319, found 788.4323. **HPLC** (Chiralpak-IA-H column, Hexane/^{i}PrOH = 90/10, flow rate: 1.0 mL/min): t_{major} = 11.040 min; t_{minor} = 12.773 min. **Specific Rotation** $[\alpha]_D^{26}$ = +1.3 (c = 0.05 in $CHCl_3$).

benzyl (*S*)-6-(benzyloxy)-2'-methyl-6'-((4-methylphenyl)sulfonamido)-[1,1'-biphenyl]-2-carboxylate (**5a**)

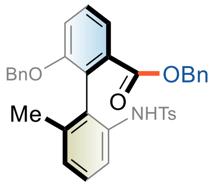

5a

Colorless oil, 28.3 mg, 98% yield, 94% ee. **^{1}H NMR** (400 MHz, $CDCl_3$) δ 7.66 (d, *J* = 1.1 Hz, 1H), 7.56 (d, *J* = 8.3 Hz, 2H), 7.51 – 7.43 (m, 2H), 7.40 (d, *J* = 4.4 Hz, 1H), 7.34 – 7.28 (m, 2H), 7.27 – 7.21 (m, 3H), 7.17 (dd, *J* = 8.3, 6.9 Hz, 2H), 7.09 (dd, *J* = 6.6, 2.9 Hz, 2H), 6.98 (d, *J* = 8.0 Hz, 2H), 6.96 – 6.91 (m, 2H), 6.89 (d, *J* = 7.6 Hz, 1H), 6.28 (s, 1H), 5.05 (d, *J* = 12.3 Hz, 1H), 4.95 – 4.82 (m, 3H), 2.22 (s, 3H), 1.76 (s, 3H). **^{13}C NMR** (101 MHz, $CDCl_3$) δ 166.3, 156.0, 143.2, 137.3, 137.0, 136.3, 135.2, 134.6, 132.6, 129.9, 129.3, 128.6, 128.4, 128.4, 128.3, 128.2, 128.0, 127.6, 127.3, 127.0, 126.0, 125.9, 125.7, 123.4, 116.7, 116.7, 70.0, 67.0, 21.4, 20.2. **HRMS** (ESI-TOF) $[M+H]^+$

calculated for $[C_{35}H_{32}NO_5S]^+$ 600.1815, found 60.1815. **HPLC** (Chiralpak-IA-H column, Hexane/^{i}PrOH = 85/15, flow rate: 1.0 mL/min): t_{minor} = 21.970 min; t_{major} = 26.287 min. **Specific Rotation** $[\alpha]_D^{21}$ = –11.4 (c = 0.1 in $CHCl_3$).

benzyl (*R*)-2',6-dimethoxy-6'-((4-methylphenyl)sulfonamido)-[1,1'-biphenyl]-2-carboxylate (**5b**)

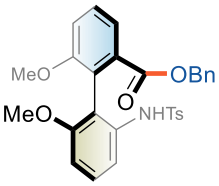

5b

White solid, 25.6 mg, 99% yield, 94% ee, **m.p.** 173-174 °C. **^{1}H NMR** (400 MHz, $CDCl_3$) δ 7.62 (d, *J* = 7.7 Hz, 1H), 7.57 – 7.42 (m, 3H), 7.35 – 7.24 (m, 4H), 7.19 (dd, *J* = 18.4, 8.1 Hz, 2H), 7.13 – 6.99 (m, 4H), 6.52 (d, *J* = 8.1 Hz, 1H), 6.42 (s, 1H), 5.00 (d, *J* = 12.3 Hz, 1H), 4.80 (d, *J* = 12.3 Hz, 1H), 3.68 (s, 3H), 3.42 (s, 3H), 2.34 (s, 3H). **^{13}C NMR** (101 MHz, $CDCl_3$) δ 166.4, 156.8, 143.2, 137.1, 135.4, 135.3, 133.3, 129.6, 129.3, 129.0, 128.4, 128.1, 127.3, 123.1, 122.2, 117.4, 115.0, 113.2, 107.0, 66.6, 56.3, 55.5, 21.5. **HRMS** (ESI-TOF) $[M+H]^+$ calculated for $[C_{29}H_{28}NO_6S]^+$ 518.1632, found 518.1633. **HPLC** (Chiralpak-IA-H column, Hexane/^{i}PrOH = 70/30, flow rate: 1.0 mL/min): t_{minor} = 13.643 min; t_{major} = 16.303 min. **Specific Rotation** $[\alpha]_D^{22}$ = +77.9 (c = 0.1 in $CHCl_3$).

benzyl (*R*)-2',6-dimethyl-6'-((4-methylphenyl)sulfonamido)-[1,1'-biphenyl]-2-carboxylate (**5c**)

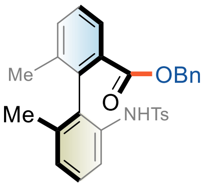

5c

Colorless oil, 24.0 mg, 99% yield, 88% ee. **^{1}H NMR** (400 MHz, $CDCl_3$) δ 7.89 (d, *J* = 7.5 Hz, 1H), 7.75 – 7.62 (m, 2H), 7.42 – 7.38 (m, 3H), 7.35 – 7.25 (m, 4H), 7.23 – 7.21 (m, 1H), 7.17 – 7.07 (m, 3H), 6.87 (d, *J* = 7.6 Hz, 1H), 6.10 (s, 1H), 5.02 (d, *J* = 12.3 Hz, 1H), 4.85 (d, *J* = 12.2 Hz, 1H), 2.39 (s, 3H), 1.72 (s, 3H), 1.67 (s, 3H). **^{13}C NMR** (101 MHz, $CDCl_3$) δ 166.8, 143.7, 138.6, 137.2, 136.5, 135.3, 134.6, 131.4, 129.8, 129.6, 128.7, 128.6, 128.6, 128.4, 128.3, 128.2, 128.0, 127.4, 127.0, 125.7, 115.9, 66.9,

21.5, 20.0, 19.4. **HRMS** (ESI-TOF) $[M+H]^+$ calculated for $[C_{29}H_{28}NO_4S]^+$ 486.1734, found 486.1737. **HPLC** (Chiralpak-IA-H column, Hexane/^{i}PrOH = 80/20, flow rate: 1.0 mL/min): t_{minor} = 10.513 min; t_{major} = 11.337 min. **Specific Rotation** $[\alpha]_D^{26}$ = –48.9 (c = 0.1 in $CHCl_3$).

benzyl (*R*)-2',5,6-trimethyl-6'-((4-methylphenyl)sulfonamido)-[1,1'-biphenyl]-2-carboxylate (**5d**)

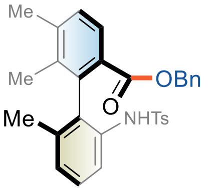

5d

Colorless oil, 21.2 mg, 85% yield, 90% ee. **^{1}H NMR** (400 MHz, $CDCl_3$) δ 7.83 (d, *J* = 8.0 Hz, 1H), 7.71 – 7.62 (m, 2H), 7.47 – 7.37 (m, 2H), 7.36 – 7.26 (m, 3H), 7.22 (d, *J* = 8.1 Hz, 2H), 7.18 – 7.04 (m, 3H), 6.87 (d, *J* = 7.6 Hz, 1H), 6.09 (s, 1H), 4.99 (d, *J* = 12.3 Hz, 1H), 4.81 (d, *J* = 12.3 Hz, 1H), 2.39 (s, 3H), 2.35 (s, 3H), 1.71 (s, 3H), 1.55 (s, 3H). **^{13}C NMR** (101 MHz, $CDCl_3$) δ 166.8, 143.6, 142.7, 137.2, 136.9, 136.7, 135.5, 135.4, 134.2, 130.2, 129.6, 128.6, 128.6, 128.4, 128.2, 128.1, 127.9, 127.4, 127.0, 125.6, 115.9, 66.7, 21.5, 21.1, 20.0, 15.5. **HRMS** (ESI-TOF) $[M+H]^+$ calculated for $[C_{30}H_{30}NO_4S]^+$ 500.1890, found 500.1894. **HPLC** (Chiralpak-IA-H column, Hexane/^{i}PrOH = 80/20, flow rate: 1.0 mL/min): t_{major} = 10.518 min; t_{minor} = 11.337 min. **Specific Rotation** $[\alpha]_D^{25}$ = +6.6 (c = 1.3 in $CHCl_3$).

methyl (*R*)-2',4,6-trimethyl-6'-((4-methylphenyl)sulfonamido)-[1,1'-biphenyl]-2-carboxylate (**5e**)

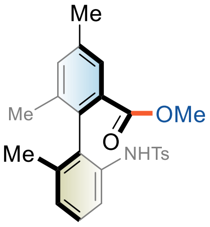

5e

White solid, 20.9 mg, 99% yield, 97% ee, **m.p.** 88-89 °C. **^{1}H NMR** (400 MHz, $CDCl_3$) δ 7.76 – 7.64 (m, 3H), 7.46 (d, *J* = 8.3 Hz, 1H), 7.33 – 7.29 (m, 1H), 7.26 (d, *J* = 8.0 Hz, 2H), 7.19 (t, *J* = 7.9 Hz, 1H), 6.97 (d, *J* = 7.5 Hz, 1H), 6.12 (s, 1H), 3.47 (s, 3H), 2.46 (s, 3H), 2.42 (s, 3H), 1.80 (s, 3H), 1.69 (s, 3H). **^{13}C NMR** (101 MHz, $CDCl_3$) δ

167.5, 143.8, 138.7, 138.4, 137.2, 136.8, 135.5, 134.4, 132.5, 131.2, 129.7, 129.2, 128.0, 127.5, 125.5, 115.4, 52.1, 21.7, 21.2, 20.1, 19.5. **HRMS** (ESI-TOF) $[M+H]^+$ calculated for $[C_{24}H_{26}NO_4S]^+$ 424.1577, found 424.1578. **HPLC** (Chiralpak-OD-H column, Hexane/^{i}PrOH = 99/1, flow rate: 1.0 mL/min): t_{major} = 16.065 min; t_{minor} = 19.399 min. **Specific Rotation** $[\alpha]_D^{26}$ = –15.6 (c = 0.1 in $CHCl_3$).

benzyl (*R*)-4-(benzyloxy)-2',6-dimethyl-6'-((4-methylphenyl)sulfonamido)-[1,1'-biphenyl]-2-carboxylate (**5f**)

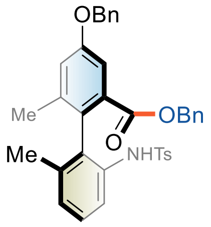

5f

Colorless oil, 29.3 mg, 99% yield, 99% ee. **^{1}H NMR** (400 MHz, $CDCl_3$) δ 7.68 (d, *J* = 8.0 Hz, 2H), 7.57 – 7.36 (m, 8H), 7.37 – 7.26 (m, 3H), 7.21 (d, *J* = 8.0 Hz, 2H), 7.18 – 7.01 (m, 3H), 6.86 (d, *J* = 7.6 Hz, 1H), 6.14 (s, 1H), 5.16 (s, 2H), 5.00 (d, *J* = 12.2 Hz, 1H), 4.82 (d, *J* = 12.2 Hz, 1H), 2.38 (s, 3H), 1.73 (s, 3H), 1.62 (s, 3H). **^{13}C NMR** (101 MHz, $CDCl_3$) δ 166.6, 158.5, 143.7, 140.2, 137.1, 136.4, 135.2, 134.5, 132.4, 129.6, 129.4, 128.8, 128.6, 128.4, 128.3, 128.3, 128.2, 127.9, 127.8, 127.4, 127.0, 125.5, 121.4, 115.5, 114.3, 70.3, 67.0, 21.6, 20.0, 19.7. **HRMS** (ESI-TOF) $[M+H]^+$ calculated for $[C_{36}H_{34}NO_5S]^+$ 592.2152, found 592.2156. **HPLC** (Chiralpak-IC-H column, Hexane/^{i}PrOH = 70/30, flow rate: 1.0 mL/min): t_{minor} = 18.020 min; t_{major} = 20.857 min. **Specific Rotation** $[\alpha]_D^{25}$ = –0.1 (c = 0.8 in $CHCl_3$).

methyl (*R*)-5-(benzyloxy)-2'-methyl-6'-((4-methylphenyl)sulfonamido)-[1,1'-biphenyl]-2-carboxylate (**5g**)

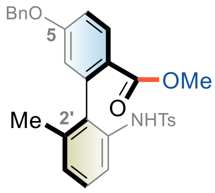

5g

Yellow oil, 24.5 mg, 98% yield, 96% ee. **^{1}H NMR** (400 MHz, $CDCl_3$) δ 8.07 (d, *J* = 8.8 Hz, 1H), 7.58 (d, *J* = 8.1 Hz, 1H), 7.52 (d, *J* = 8.3 Hz, 2H), 7.46 (d, *J* = 4.4 Hz, 4H), 7.44 – 7.38 (m, 1H), 7.23 (t, *J* = 7.9 Hz, 1H), 7.16 (d, *J* = 8.1 Hz, 2H), 7.06 (dd, *J* = 8.8,

2.6 Hz, 1H), 7.00 (d, J = 7.6 Hz, 1H), 6.12 (s, 1H), 6.04 (d, J = 2.6 Hz, 1H), 5.14 – 4.96 (m, 2H), 3.55 (s, 3H), 2.26 (s, 3H), 1.81 (s, 3H). **^{13}C NMR** (101 MHz, $CDCl_3$) δ 166.1, 161.9, 143.6, 139.4, 136.6, 136.3, 135.9, 133.6, 133.5, 133.1, 129.6, 128.8, 128.5, 128.0, 127.7, 127.2, 126.2, 122.6, 118.2, 117.7, 114.0, 70.2, 51.9, 21.4, 20.3. **HRMS** (ESI-TOF) $[M+H]^+$ calculated for $[C_{29}H_{28}NO_5S]^+$ 502.1683, found 502.1686. **HPLC** (Chiralpak-IC-H column, Hexane/^{i}PrOH = 70/30, flow rate: 1.0 mL/min): t_{major} = 23.937 min; t_{minor} = 27.220 min. **Specific Rotation** $[\alpha]_D^{23}$ = +5.1 (c = 0.3 in $CHCl_3$).

benzyl (*S*)-2',4'-dimethoxy-6-methyl-6'-((4-methylphenyl)sulfonamido)-[1,1'-biphenyl]-2-carboxylate (**5h**)

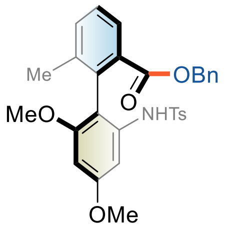

5h

Yellow oil, 26.3 mg, 99% yield, 86% ee. **^{1}H NMR** (400 MHz, $CDCl_3$) δ 7.88 (dd, J = 7.2, 2.0 Hz, 1H), 7.72 – 7.60 (m, 2H), 7.46 – 7.36 (m, 2H), 7.36 – 7.26 (m, 3H), 7.23 (d, J = 8.0 Hz, 2H), 7.17 – 7.04 (m, 2H), 6.83 (d, J = 2.3 Hz, 1H), 6.15 (s, 1H), 6.07 (d, J = 2.3 Hz, 1H), 5.06 (d, J = 12.2 Hz, 1H), 4.83 (d, J = 12.2 Hz, 1H), 3.80 (s, 3H), 3.45 (s, 3H), 2.39 (s, 3H), 1.70 (s, 3H). **^{13}C NMR** (101 MHz, $CDCl_3$) δ 167.1, 160.4, 157.3, 143.9, 139.9, 136.9, 135.5, 135.3, 134.2, 132.6, 132.2, 129.6, 128.5, 128.5, 128.3, 128.3, 128.1, 127.5, 111.6, 95.6, 94.6, 66.7, 55.5, 55.3, 21.6, 19.6. **HRMS** (ESI-TOF) $[M+H]^+$ calculated for $[C_{30}H_{30}NO_6S]^+$ 532.1788, found 532.1792. **HPLC** (Chiralpak-IC-H column, Hexane/^{i}PrOH = 80/20, flow rate: 1.0 mL/min): t_{major} = 25.787 min; t_{minor} = 29.823 min. **Specific Rotation** $[\alpha]_D^{25}$ = +21.5 (c = 0.1 in $CHCl_3$).

benzyl (*R*)-2',4',6-trimethyl-6'-((4-methylphenyl)sulfonamido)-[1,1'-biphenyl]-2-carboxylate (**5i**)

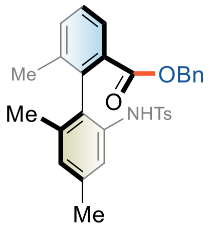

5i

White oil, 24.7 mg, 99% yield, 96% ee. **^{1}H NMR** (400 MHz, $CDCl_3$) δ 7.88 (dd, *J* = 7.3, 1.9 Hz, 1H), 7.71 – 7.63 (m, 2H), 7.48 – 7.37 (m, 2H), 7.35 – 7.26 (m, 3H), 7.23 (d, *J* = 8.1 Hz, 3H), 7.14 – 7.05 (m, 2H), 6.68 (s, 1H), 6.06 (s, 1H), 5.04 (d, *J* = 12.2 Hz, 1H), 4.86 (d, *J* = 12.2 Hz, 1H), 2.39 (s, 3H), 2.29 (s, 3H), 1.68 (s, 3H), 1.65 (s, 3H). **^{13}C NMR** (101 MHz, $CDCl_3$) δ 167.0, 143.7, 138.8, 137.7, 137.2, 136.1, 135.6, 135.3, 134.5, 133.8, 131.6, 129.6, 128.6, 128.5, 128.4, 128.4, 128.3, 128.1, 127.4, 127.0, 126.9, 126.7, 116.7, 66.9, 19.9, 19.5. **HRMS** (ESI-TOF) $[M+H]^+$ calculated for $[C_{30}H_{30}NO_4S]^+$ 500.1890, found 500.1893. **HPLC** (Chiralpak-IC-H column, Hexane/^{i}PrOH = 80/20, flow rate: 1.0 mL/min): t_{major} = 20.550 min; t_{minor} = 18.230 min. **Specific Rotation** $[\alpha]_D^{25}$ = –3.6 (c = 0.8 in $CHCl_3$).

(*R*)-2-((benzyloxy)carbonyl)-2',6-dimethyl-6'-((4-methylphenyl)sulfonamido)-[1,1'-biphenyl]-4-yl (1R,4S)-4,7,7-trimethyl-3-oxo-2-oxabicyclo[2.2.1]heptane-1-carboxylate (**5j**)

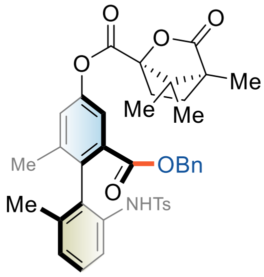

5j

Colorless oil, 22.8 mg, 67% yield, 90% de. **^{1}H NMR** (400 MHz, $CDCl_3$) δ 7.73 – 7.62 (m, 3H), 7.38 (d, *J* = 8.3 Hz, 1H), 7.33 – 7.27 (m, 4H), 7.24 (d, *J* = 8.0 Hz, 2H), 7.14 (t, *J* = 7.9 Hz, 1H), 7.10 – 7.02 (m, 2H), 6.87 (d, *J* = 7.6 Hz, 1H), 6.12 (s, 1H), 5.01 (d, *J* = 12.1 Hz, 1H), 4.85 (d, *J* = 12.1 Hz, 1H), 2.68 – 2.56 (m, 1H), 2.40 (s, 3H), 2.32 – 2.20 (m, 1H), 2.12 – 1.97 (m, 1H), 1.88 – 1.77 (m, 1H), 1.72 (s, 3H), 1.67 (s, 3H), 1.22 (s, 6H), 1.17 (s, 3H). **^{13}C NMR** (101 MHz, $CDCl_3$) δ 13C NMR (101 MHz, CDCl3) δ 177.8, 165.8, 165.8, 149.6, 143.8, 140.8, 137.2, 136.7, 134.9, 134.2, 133.8, 132.9, 129.7, 129.2, 128.5, 128.4, 128.3, 127.4, 127.1, 126.0, 121.3, 116.5, 90.8, 67.3, 55.0, 54.9, 30.8, 29.0, 21.6, 20.0, 19.6, 16.9, 9.8. **HRMS** (ESI-TOF) $[M+H]^+$ calculated for $[C_{39}H_{39}NO_8SNa]^+$ 704.2289, found 704.2288. **HPLC** (Chiralpak-IA-H column, Hexane/^{i}PrOH = 70/30, flow rate: 1.0 mL/min): t_{minor} = 8.453 min; t_{major} = 9.570 min. **Specific Rotation** $[\alpha]_D^{25}$ = +17.0 (c = 0.7 in $CHCl_3$).

benzyl (*R*)-4-((5-(2,5-dimethylphenoxy)-2,2-dimethylpentanoyl)oxy)-2',6-dimethyl-6'-((4-methylphenyl)sulfonamido)-[1,1'-biphenyl]-2-carboxylate (**5k**)

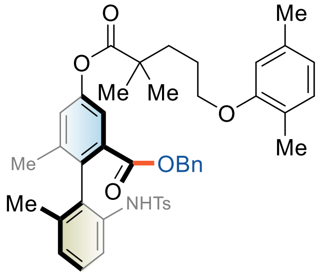

5k

Colorless oil, 35.9 mg, 98% yield, 92% ee. **^{1}H NMR** (400 MHz, $CDCl_3$) δ 7.76 – 7.66 (m, 2H), 7.62 (d, *J* = 2.5 Hz, 1H), 7.41 (d, *J* = 8.2 Hz, 1H), 7.37 – 7.18 (m, 6H), 7.14 (t, *J* = 7.9 Hz, 1H), 7.10 – 7.01 (m, 3H), 6.87 (d, *J* = 7.6 Hz, 1H), 6.78 – 6.64 (m, 2H), 6.20 (s, 1H), 5.00 (d, *J* = 12.2 Hz, 1H), 4.84 (d, *J* = 12.2 Hz, 1H), 4.11 – 3.96 (m, 2H), 2.39 (s, 3H), 2.35 (s, 3H), 2.23 (s, 3H), 1.96 (s, 4H), 1.73 (s, 3H), 1.66 (s, 3H), 1.45 (s, 6H). **^{13}C NMR** (101 MHz, $CDCl_3$) δ 176.1, 166.0, 156.9, 150.7, 143.7, 140.4, 137.2, 136.8, 136.5, 135.0, 134.3, 132.9, 132.7, 130.4, 129.6, 129.2, 128.4, 128.4, 128.2, 128.2, 127.6, 127.4, 125.8, 123.6, 121.6, 120.8, 116.2, 112.0, 67.7, 67.1, 42.6, 37.2, 25.3, 25.2, 21.5, 21.5, 20.0, 19.6, 15.9. **HRMS** (ESI-TOF) $[M+H]^+$ calculated for $[C_{44}H_{47}NO_7SNa]^+$ 756.2965, found 756.2964. **HPLC** (Chiralpak-IC-H column, Hexane/^{i}PrOH = 70/30, flow rate: 1.0 mL/min): t_{minor} = 10.013 min; t_{major} = 13.873 min. **Specific Rotation** $[\alpha]_D^{25}$ = +3.8 (c = 0.9 in $CHCl_3$).

benzyl (*R*)-3-methyl-2-(2-((4-methylphenyl)sulfonamido)naphthalen-1-yl)benzoate (**5l**)

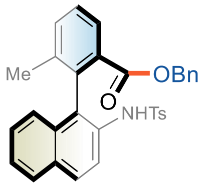

5l

White oil, 25.8 mg, 99% yield, 96% ee. **^{1}H NMR** (400 MHz, $CDCl_3$) δ 8.03 – 7.97 (m, 1H), 7.82 (d, *J* = 9.0 Hz, 1H), 7.79 – 7.71 (m, 2H), 7.72 – 7.65 (m, 2H), 7.56 – 7.49 (m, 2H), 7.40 (d, *J* = 4.7 Hz, 1H), 7.38 – 7.31 (m, 1H), 7.27 – 7.19 (m, 3H), 7.19 – 7.11 (m, 2H), 6.91 (d, *J* = 8.5 Hz, 1H), 6.83 – 6.75 (m, 2H), 6.41 (s, 1H), 4.86 (d, *J* = 12.2 Hz, 1H), 4.66 (d, *J* = 12.2 Hz, 1H), 2.38 (s, 3H), 1.52 (s, 3H). **^{13}C NMR** (101 MHz, $CDCl_3$) δ 166.7, 143.8, 139.6, 137.4, 135.0, 134.6, 134.3, 132.3, 132.1, 131.6, 130.6, 129.7, 129.0, 128.9, 128.6, 128.3, 128.1, 127.9, 127.3, 127.0, 126.8, 125.6, 124.8, 124.7, 118.4, 66.8, 21.5, 19.5. **HRMS** (ESI-TOF) $[M+H]^+$ calculated for $[C_{32}H_{28}NO_4S]^+$ 522.1734,

found 522.1737. **HPLC** (Chiralpak-IC-H column, Hexane/^{i}PrOH = 70/30, flow rate: 1.0 mL/min): t_{minor} = 17.210 min; t_{major} = 18.573 min. **Specific Rotation** $[\alpha]_D^{25}$ = –18.9 (c = 0.6 in $CHCl_3$).

benzyl (*S*)-4-(2-methyl-6-((4-methylphenyl)sulfonamido)phenyl)dibenzo[b,d]furan-3-carboxylate (**5m**)

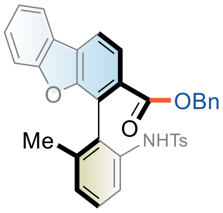

5m

Colorless oil, 27.8 mg, 99% yield, 96% ee. **^{1}H NMR** (400 MHz, $CDCl_3$) δ 8.13 (d, *J* = 8.1 Hz, 1H), 8.10 – 8.00 (m, 2H), 7.62 (d, *J* = 8.2 Hz, 1H), 7.59 – 7.48 (m, 1H), 7.48 – 7.37 (m, 2H), 7.38 – 7.25 (m, 6H), 7.24 – 7.12 (m, 2H), 7.05 (d, *J* = 7.6 Hz, 1H), 6.78 (d, *J* = 8.1 Hz, 2H), 6.40 (s, 1H), 5.20 (d, *J* = 12.2 Hz, 1H), 5.02 (d, *J* = 12.2 Hz, 1H), 2.06 (s, 3H), 1.75 (s, 3H). **^{13}C NMR** (101 MHz, $CDCl_3$) δ 166.4, 157.2, 153.8, 143.1, 137.5, 136.6, 135.1, 134.5, 129.1, 128.8, 128.7, 128.5, 128.5, 128.3, 128.1, 127.3, 127.0, 126.8, 126.7, 125.9, 123.4, 123.1, 121.8, 121.5, 120.5, 119.7, 112.3, 67.4, 21.4, 20.2. **HRMS** (ESI-TOF) $[M+H]^+$ calculated for $[C_{34}H_{28}NO_5S]^+$ 562.1683, found 562.1686. **HPLC** (Chiralpak-AS-H column, Hexane/^{i}PrOH = 60/40, flow rate: 1.0 mL/min): t_{minor} = 12.207 min; t_{major} = 20.967 min. **Specific Rotation** $[\alpha]_D^{23}$ = +64.0 (c = 0.05 in $CHCl_3$).

benzyl (*S*)-3-methoxy-2-(2-methyl-6-((4-methylphenyl)sulfonamido)phenyl)-1-naphthoate (**5n**)

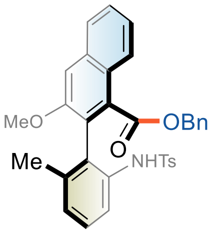

5n

White solid, 27.3 mg, 99% yield, 92% ee, **m.p.** 60-61 °C. **^{1}H NMR** (400 MHz, $CDCl_3$) δ 7.84 (d, *J* = 8.2 Hz, 1H), 7.79 – 7.70 (m, 1H), 7.65 – 7.52 (m, 3H), 7.45 (ddd, *J* = 8.3, 6.9, 1.3 Hz, 1H), 7.39 (d, *J* = 8.2 Hz, 1H), 7.34 – 7.25 (m, 3H), 7.25 – 7.08 (m, 6H), 6.93 (d, *J* = 7.6 Hz, 1H), 6.45 (s, 1H), 5.13 (d, *J* = 11.9 Hz, 1H), 4.99 (d, *J* = 11.9 Hz,

1H), 3.61 (s, 3H), 2.40 (s, 3H), 1.84 (s, 3H). **^{13}C NMR** (101 MHz, $CDCl_3$) δ 168.0, 154.2, 143.2, 138.6, 137.5, 135.0, 134.8, 134.7, 133.7, 129.3, 128.7, 128.6, 128.5, 128.3, 127.5, 127.3, 127.0, 126.6, 125.9, 125.2, 125.1, 125.0, 124.5, 117.5, 107.6, 67.5, 55.8, 21.6, 20.1. **HRMS** (ESI-TOF) $[M+H]^+$ calculated for $[C_{33}H_{30}NO_5S]^+$ 552.1839, found 552.1841. **HPLC** (Chiralpak-IC-H column, Hexane/^{i}PrOH = 70/30, flow rate: 1.0 mL/min): t_{major} = 23.937 min; t_{minor} = 27.220 min. **Specific Rotation** $[\alpha]_D^{23}$ = –68.7 (c = 0.1 in $CHCl_3$).

benzyl (*S*)-1-(2,4-dimethoxy-6-((4-methylphenyl)sulfonamido)phenyl)-2-naphthoate (**5o**)

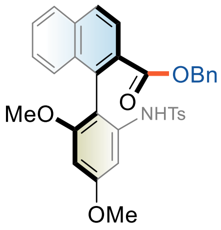

5o

White solid, 28.1 mg, 99% yield, 90% ee, **m.p.** 60-61 °C. **^{1}H NMR** (400 MHz, $CDCl_3$) δ 8.08 (d, *J* = 8.6 Hz, 1H), 7.97 (d, *J* = 8.6 Hz, 1H), 7.90 (d, *J* = 8.1 Hz, 1H), 7.54 (t, *J* = 8.1 Hz, 1H), 7.40 (d, *J* = 8.2 Hz, 2H), 7.36 – 7.30 (m, 3H), 7.20 – 7.09 (m, 3H), 7.09 – 6.94 (m, 4H), 6.14 (d, *J* = 2.3 Hz, 1H), 6.11 (s, 1H), 5.16 (d, *J* = 12.2 Hz, 1H), 4.94 (d, *J* = 12.2 Hz, 1H), 3.87 (s, 3H), 3.35 (s, 3H), 2.37 (s, 3H). **^{13}C NMR** (101 MHz, $CDCl_3$) δ 167.1, 160.8, 158.1, 143.6, 136.4, 136.0, 135.4, 135.2, 132.6, 132.5, 129.6, 129.4, 129.0, 128.5, 128.4, 128.2, 128.1, 127.7, 127.3, 127.2, 126.3, 126.3, 110.4, 96.2, 94.9, 67.0, 55.5, 55.4, 21.6. **HRMS** (ESI-TOF) $[M+H]^+$ calculated for $[C_{33}H_{30}NO_6S]^+$ 568.1788, found 568.1791. **HPLC** (Chiralpak-IC-H column, Hexane/^{i}PrOH = 70/30, flow rate: 1.0 mL/min): t_{major} = 24.070 min; t_{minor} = 27.367 min. **Specific Rotation** $[\alpha]_D^{22}$ = +15.0 (c = 0.04 in $CHCl_3$).

S-benzyl (*S*)-6-methoxy-2'-methyl-6'-((4-methylphenyl)sulfonamido)-[1,1'-biphenyl]-2-carbothioate (**6a**)

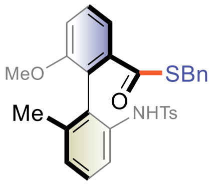

6a

Colorless oil, 24.8 mg, 96% yield, 96% ee. **^{1}H NMR** (400 MHz, $CDCl_3$) δ 7.71 – 7.64 (m, 2H), 7.49 (t, *J* = 8.0 Hz, 1H), 7.45 – 7.37 (m, 2H), 7.27 – 7.22 (m, 3H), 7.19 (dd, *J* = 8.0, 4.4 Hz, 3H), 7.14 – 7.06 (m, 3H), 6.96 (d, *J* = 7.6 Hz, 1H), 6.38 (s, 1H), 4.05 (s, 2H), 3.58 (s, 3H), 2.38 (s, 3H), 1.83 (s, 3H). **^{13}C NMR** (101 MHz, $CDCl_3$) δ 192.4, 157.2, 143.2, 139.9, 137.9, 137.4, 137.2, 135.3, 130.1, 129.4, 128.7, 128.6, 128.4, 127.4, 127.2, 126.5, 125.8, 122.2, 120.3, 117.1, 114.3, 56.0, 33.9, 21.6, 20.3. **HRMS** (ESI-TOF) $[M+H]^+$ calculated for $[C_{29}H_{28}NO_4S_2]^+$ 518.1454, found 518.1456. **HPLC** (Chiralpak-IC-H column, Hexane/^{i}PrOH = 80/20, flow rate: 1.0 mL/min): t_{minor} = 34.420 min; t_{major} = 37.573 min. **Specific Rotation** $[\alpha]_D^{25}$ = –1.4 (c = 0.7 in $CHCl_3$).

S-(2-methylbenzyl) (*S*)-6-methoxy-2'-methyl-6'-((4-methylphenyl)sulfonamido)-[1,1'-biphenyl]-2-carbothioate (**6b**)

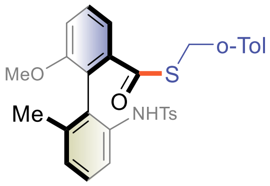

6b

Yellow oil, 26.3 mg, 99% yield, 92% ee. **^{1}H NMR** (400 MHz, $CDCl_3$) δ 7.67 (d, *J* = 8.3 Hz, 2H), 7.48 (t, *J* = 8.0 Hz, 1H), 7.41 (d, *J* = 7.9 Hz, 2H), 7.24 – 7.05 (m, 8H), 6.95 (d, *J* = 7.6 Hz, 1H), 6.39 (s, 1H), 4.17 – 3.97 (m, 2H), 3.57 (s, 3H), 2.40 (s, 3H), 2.22 (s, 3H), 1.83 (s, 3H). **^{13}C NMR** (101 MHz, $CDCl_3$) δ 192.6, 157.1, 143.2, 139.9, 137.9, 137.4, 136.7, 135.3, 134.5, 130.4, 130.1, 129.8, 129.4, 128.4, 127.7, 127.4, 126.6, 126.2, 125.8, 122.2, 120.3, 117.1, 114.3, 56.0, 32.2, 21.6, 20.3, 19.2. **HRMS** (ESI-TOF) $[M+H]^+$ calculated for $[C_{30}H_{30}NO_4S_2]^+$ 532.1611, found 532.1611. **HPLC** (Chiralpak-IC-H column, Hexane/^{i}PrOH = 80/20, flow rate: 1.0 mL/min): t_{minor} = 37.117 min; t_{major} = 40.387 min. **Specific Rotation** $[\alpha]_D^{21}$ = –12.0 (c = 0.1 in $CHCl_3$).

S-(2-bromobenzyl) (*S*)-6-methoxy-2'-methyl-6'-((4-methylphenyl)sulfonamido)-[1,1'-biphenyl]-2-carbothioate (**6c**)

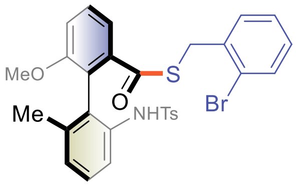

6c

Colorless oil, 23.8 mg, 80% yield, 86% ee. **^{1}H NMR** (400 MHz, $CDCl_3$) δ 7.66 (d, *J* = 8.4 Hz, 2H), 7.56 – 7.44 (m, 2H), 7.43 – 7.35 (m, 2H), 7.22 – 7.14 (m, 5H), 7.14 – 7.04 (m, 2H), 6.92 (d, *J* = 7.5 Hz, 1H), 6.37 (s, 1H), 4.26 – 4.08 (m, 2H), 3.57 (s, 3H), 2.39 (s, 3H), 1.81 (s, 3H). **^{13}C NMR** (101 MHz, $CDCl_3$) δ 192.1, 157.1, 143.2, 139.8, 137.9, 137.4, 136.7, 135.3, 132.7, 131.0, 130.1, 129.4, 128.9, 128.4, 127.7, 127.4, 126.5, 125.8, 124.4, 122.2, 120.2, 117.1, 114.3, 55.9, 34.4, 21.6, 20.2. **HRMS** (ESI-TOF) $[M+H]^+$ calculated for $[C_{29}H_{27}BrNO_4S_2]^+$ 596.0559, found 596.0558. **HPLC** (Chiralpak-IC-H column, Hexane/^{i}PrOH = 70/30, flow rate: 1.0 mL/min): t_{minor} = 20.070 min; t_{major} = 21.217 min. **Specific Rotation** $[\alpha]_D^{26}$ = +0.9 (c = 0.1 in $CHCl_3$).

S-(4-methoxybenzyl) (*S*)-6-methoxy-2'-methyl-6'-((4-methylphenyl)sulfonamido)-[1,1'-biphenyl]-2-carbothioate (**6d**)

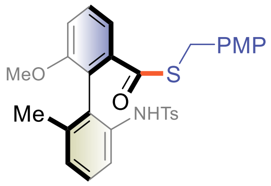

6d

White solid, 49.2 mg, 90% yield, 94% ee. **^{1}H NMR** (400 MHz, $CDCl_3$) δ 7.66 (d, *J* = 8.4 Hz, 2H), 7.46 (t, *J* = 8.0 Hz, 1H), 7.43 – 7.37 (m, 2H), 7.21 – 7.16 (m, 3H), 7.06 (dd, *J* = 8.3, 1.1 Hz, 1H), 7.04 – 7.00 (m, 2H), 6.95 (d, *J* = 7.6 Hz, 1H), 6.80 – 6.74 (m, 2H), 6.40 (s, 1H), 4.00 (s, 2H), 3.79 (s, 3H), 3.55 (s, 3H), 2.37 (s, 3H), 1.83 (s, 3H). **^{13}C NMR** (101 MHz, $CDCl_3$) δ 192.7, 158.8, 157.1, 143.2, 140.0, 137.9, 137.4, 135.3, 130.1, 129.8, 129.4, 129.1, 128.3, 127.4, 126.7, 125.8, 122.2, 120.2, 117.2, 114.2, 114.0, 55.9, 55.3, 33.4, 21.5, 20.3. **HRMS** (ESI-TOF) $[M+Na]^+$ calculated for $[C_{30}H_{29}NNaO_5S_2]^+$ 570.1379, found 570.1379. **HPLC** (Chiralpak-IA-H column, Hexane/Ethanol = 90/10, flow rate: 1.2 mL/min): t_{major} = 21.204 min; t_{minor} = 25.328 min. **Specific Rotation** $[\alpha]_D^{25}$ = –21.2 (c = 1.0 in $CHCl_3$).

S-phenethyl (*S*)-6-methoxy-2'-methyl-6'-((4-methylphenyl)sulfonamido)-[1,1'-biphenyl]-2-carbothioate (**6e**)

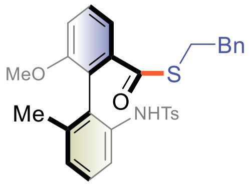

6e

White solid, 44.1 mg, 83% yield, 97% ee. **^{1}H NMR** (400 MHz, $CDCl_3$) δ 7.64 (d, *J* = 8.3 Hz, 2H), 7.46 (t, *J* = 8.0 Hz, 1H), 7.41 – 7.36 (m, 2H), 7.28 (d, *J* = 6.8 Hz, 1H), 7.25 (s, 1H), 7.22 – 7.14 (m, 4H), 7.13 – 7.09 (m, 2H), 7.04 (dd, *J* = 8.3, 1.1 Hz, 1H), 6.94 (d, *J* = 7.5 Hz, 1H), 6.37 (s, 1H), 3.54 (s, 3H), 3.08 – 2.94 (m, 2H), 2.69 (t, *J* = 7.6 Hz, 2H), 2.35 (s, 3H), 1.83 (s, 3H). **^{13}C NMR** (101 MHz, $CDCl_3$) δ 193.0, 157.2, 143.2, 140.3, 139.9, 138.0, 137.5, 135.3, 130.1, 129.4, 128.6, 128.5, 128.4, 127.5, 126.6, 126.5, 125.7, 122.2, 120.3, 117.1, 114.2, 56.0, 35.5, 31.1, 21.6, 20.3. **HRMS** (ESI-TOF) $[M+Na]^+$ calculated for $[C_{30}H_{29}NNaO_4S_2]^+$ 554.1430, found 554.1436. **HPLC** (Chiralpak-IA-H column, Hexane/Ethanol = 90/10, flow rate: 0.4 mL/min): t_{major} = 40.287 min; t_{minor} = 42.746 min. **Specific Rotation** $[\alpha]_D^{25}$ = –38.1 (c = 1.0 in $CHCl_3$).

S-hexadecyl (*S*)-6-methoxy-2'-methyl-6'-((4-methylphenyl)sulfonamido)-[1,1'-biphenyl]-2-carbothioate (**6f**)

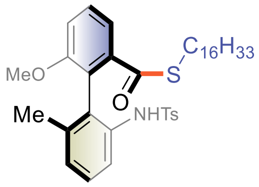

6f

White solid, 54.0 mg, 83% yield, 95% ee. **^{1}H NMR** (400 MHz, $CDCl_3$) δ 7.65 (d, *J* = 8.3 Hz, 2H), 7.47 (t, *J* = 8.0 Hz, 1H), 7.42 – 7.36 (m, 2H), 7.17 (dd, *J* = 15.2, 7.9 Hz, 3H), 7.05 (dd, *J* = 8.2, 1.1 Hz, 1H), 6.94 (d, *J* = 7.5 Hz, 1H), 6.38 (s, 1H), 3.55 (s, 3H), 2.77 (td, *J* = 7.1, 3.4 Hz, 2H), 2.39 (s, 3H), 1.85 (s, 3H), 1.42 (dd, *J* = 13.6, 6.6 Hz, 3H), 1.24 (d, *J* = 20.3 Hz, 25H), 0.88 (d, *J* = 7.0 Hz, 3H). **^{13}C NMR** (101 MHz, $CDCl_3$) δ 193.4, 157.1, 143.2, 140.5, 138.0, 137.5, 135.3, 130.1, 129.4, 128.4, 127.5, 126.7, 125.7, 122.2, 120.3, 117.2, 114.1, 56.0, 32.0, 29.8, 29.8, 29.8, 29.7, 29.7, 29.5, 29.4, 29.3, 29.2, 28.7, 22.8, 21.6, 20.3, 14.2. **HRMS** (ESI-TOF) $[M+Na]^+$ calculated for $[C_{38}H_{53}NNaO_4S_2]^+$ 674.3308, found 674.3308. **HPLC** (Chiralpak-IA-H column, Hexane/Ethanol = 97/3, flow rate: 1.2 mL/min): t_{major} = 10.603 min; t_{minor} = 12.077 min. **Specific Rotation** $[\alpha]_D^{25}$ = –16.6 (c = 1.0 in $CHCl_3$).

S-cyclopentyl (*S*)-6-methoxy-2'-methyl-6'-((4-methylphenyl)sulfonamido)-[1,1'-biphenyl]-2-carbothioate (**6g**)

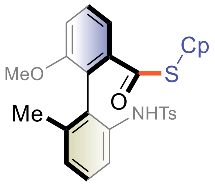

6g

White solid, 41.6 mg, 84% yield, 98% ee. **^{1}H NMR** (400 MHz, $CDCl_3$) δ 7.63 (d, *J* = 8.3 Hz, 2H), 7.46 (t, *J* = 8.0 Hz, 1H), 7.41 – 7.34 (m, 2H), 7.22 – 7.13 (m, 3H), 7.06 – 7.01 (m, 1H), 6.95 (d, *J* = 7.6 Hz, 1H), 6.39 (s, 1H), 3.59 (p, *J* = 7.2 Hz, 1H), 3.54 (s, 3H), 2.38 (s, 3H), 2.02 (dt, *J* = 13.2, 6.7 Hz, 1H), 1.97 – 1.86 (m, 1H), 1.85 (s, 3H), 1.66 – 1.53 (m, 4H), 1.44 (dt, *J* = 13.2, 6.3 Hz, 1H), 1.32 (dd, *J* = 13.6, 6.9 Hz, 1H). **^{13}C NMR** (101 MHz, $CDCl_3$) δ 194.1, 157.1, 143.1, 140.5, 137.9, 137.5, 135.2, 130.0, 129.4, 128.3, 127.4, 126.8, 125.7, 122.0, 120.2, 117.2, 113.9, 55.9, 43.2, 33.0, 32.7, 24.7, 24.6, 21.6, 20.3. **HRMS** (ESI-TOF) $[M+Na]^+$ calculated for $[C_{27}H_{29}NNaO_4S_2]^+$ 518.1430, found 518.1437. **HPLC** (Chiralpak-IA-H column, Hexane/Ethanol = 95/5, flow rate: 1.2 mL/min): t_{major} = 14.390 min; t_{minor} = 16.179 min. **Specific Rotation** $[\alpha]^{25}_{D}$ = –35.0 (c = 1.0 in $CHCl_3$).

S-cyclohexyl (*R*)-1-(2-methyl-6-((4-methylphenyl)sulfonamido)phenyl)naphthalene-2-carbothioate (**6h**)

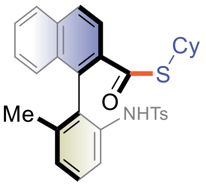

6h

White solid, 49.7 mg, 94% yield, 90% ee. **^{1}H NMR** (400 MHz, $CDCl_3$) δ 7.97 (d, *J* = 8.5 Hz, 1H), 7.86 (dd, *J* = 17.6, 8.4 Hz, 2H), 7.62 (d, *J* = 8.2 Hz, 1H), 7.51 (ddd, *J* = 8.2, 6.8, 1.2 Hz, 1H), 7.33 (dd, *J* = 10.6, 8.1 Hz, 3H), 7.10 (ddd, *J* = 8.3, 6.8, 1.3 Hz, 1H), 7.04 (d, *J* = 7.6 Hz, 1H), 6.95 (d, *J* = 8.0 Hz, 2H), 6.83 – 6.78 (m, 1H), 6.40 (s, 1H), 3.53 (ddt, *J* = 14.0, 9.4, 3.7 Hz, 1H), 2.33 (s, 3H), 1.93 – 1.85 (m, 1H), 1.79 – 1.72 (m, 1H), 1.71 (s, 3H), 1.70 – 1.52 (m, 4H), 1.44 – 1.31 (m, 4H). **^{13}C NMR** (101 MHz, $CDCl_3$) δ 194.1, 143.2, 138.3, 136.8, 136.7, 135.5, 134.6, 131.6, 131.6, 129.3, 129.2, 128.8, 128.4, 128.2, 127.5, 127.5, 127.0, 126.1, 126.1, 124.0, 118.0, 43.3, 32.8, 32.6, 25.9, 25.9, 25.5, 21.6, 20.2. **HRMS** (ESI-TOF) $[M+Na]^+$ calculated for $[C_{31}H_{31}NNaO_3S_2]^+$ 552.1638, found 552.1641. **HPLC** (Chiralpak-IA-H column,

Hexane/Ethanol = 90/10, flow rate: 1.2 mL/min): t_{major} = 7.793 min; t_{minor} = 9.133 min. **Specific Rotation** $[\alpha]_D^{25}$ = +39.0 (c = 1.0 in $CHCl_3$).

S-((3*S*,8*S*,9*S*,10*R*,13*R*,14*S*,17*R*)-10,13-dimethyl-17-((*R*)-6-methylheptan-2-yl)-2,3,4,7,8,9,10,11,12,13,14,15,16,17-tetradecahydro-1H-cyclopenta[a]phenanthren-3-yl) (*R*)-1-(2-methyl-6-((4-methylphenyl)sulfonamido)phenyl)naphthalene-2-carbothioate (**6i**)

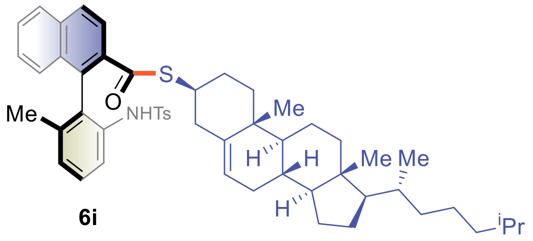

6i

White solid, 77.4 mg, 95% yield, 86% de. **^{1}H NMR** (400 MHz, $CDCl_3$) δ 7.97 (d, *J* = 8.6 Hz, 1H), 7.88 (d, *J* = 8.2 Hz, 1H), 7.83 (d, *J* = 8.6 Hz, 1H), 7.61 (d, *J* = 8.2 Hz, 1H), 7.51 (ddd, *J* = 8.1, 6.8, 1.2 Hz, 1H), 7.38 – 7.28 (m, 3H), 7.10 (ddd, *J* = 8.3, 6.8, 1.3 Hz, 1H), 7.04 (d, *J* = 7.6 Hz, 1H), 6.95 (d, *J* = 8.0 Hz, 2H), 6.82 (dd, *J* = 8.5, 1.1 Hz, 1H), 6.39 (s, 1H), 5.39 – 5.24 (m, 1H), 3.36 (tt, *J* = 12.7, 4.1 Hz, 1H), 2.33 (s, 3H), 2.28 – 2.18 (m, 1H), 2.09 – 1.92 (m, 3H), 1.91 – 1.79 (m, 3H), 1.71 (s, 3H), 1.58 – 1.33 (m, 9H), 1.25 – 1.01 (m, 9H), 0.99 (s, 4H), 0.94 (d, *J* = 6.5 Hz, 4H), 0.89 (dd, *J* = 6.6, 1.8 Hz, 7H), 0.69 (s, 3H). **^{13}C NMR** (101 MHz, $CDCl_3$) δ 193.9, 143.2, 140.9, 138.4, 136.9, 136.7, 135.5, 134.6, 131.6, 131.6, 129.3, 129.2, 128.8, 128.4, 128.2, 127.6, 127.5, 127.1, 126.1, 126.1, 123.9, 121.9, 118.0, 56.7, 56.2, 50.2, 44.2, 42.3, 39.8, 39.6, 39.5, 38.5, 36.6, 36.2, 35.8, 31.8, 31.8, 29.0, 28.3, 28.1, 24.3, 23.9, 22.9, 22.6, 21.6, 20.9, 20.3, 19.3, 18.8, 11.9. **HRMS** (ESI-TOF) $[M+Na]^+$ calculated for $[C_{52}H_{65}NNaO_3S_2]^+$ 838.4298, found 838.4310. **HPLC** (Chiralpak-IA-H column, Hexane/Ethanol = 95/5, flow rate: 1.5 mL/min): t_{major} = 6.752 min; t_{minor} = 8.447 min. **Specific Rotation** $[\alpha]_D^{25}$ = +20.5 (c = 1.0 in $CHCl_3$).

S-benzyl (*R*)-2',4,6-trimethyl-6'-((4-methylphenyl)sulfonamido)-[1,1'-biphenyl]-2-carbothioate (**6j**)

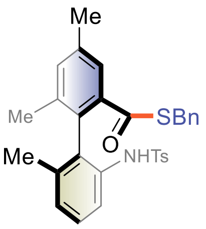

6j

White oil, 25.0 mg, 97% yield, 88% ee. **^{1}H NMR** (400 MHz, $CDCl_3$) δ 7.76 (d, *J* = 8.3 Hz, 2H), 7.50 (s, 1H), 7.38 (d, *J* = 8.2 Hz, 1H), 7.29 – 7.17 (m, 7H), 7.17 – 7.10 (m, 2H), 6.97 (d, *J* = 7.6 Hz, 1H), 6.34 (s, 1H), 4.06 (d, *J* = 1.6 Hz, 2H), 2.46 (s, 3H), 2.41 (s, 3H), 1.83 (s, 3H), 1.69 (s, 3H). **^{13}C NMR** (101 MHz, $CDCl_3$) δ 193.1, 143.7, 138.7, 138.7, 137.4, 137.4, 137.4, 135.2, 135.0, 129.9, 129.6, 128.9, 128.7, 128.6, 128.3, 127.5, 127.3, 126.7, 125.7, 116.1, 33.9, 21.6, 21.2, 20.2, 19.3. **HRMS** (ESI-TOF) $[M+H]^+$ calculated for $[C_{30}H_{30}NO_3S_2]^+$ 516.1662, found 516.1665. **HPLC** (Chiralpak-AD-H column, Hexane/^{i}PrOH = 90/10, flow rate: 1.0 mL/min): t_{major} = 9.537 min; t_{minor} = 11.752 min. **Specific Rotation** $[\alpha]_D^{25}$ = –20.5 (c = 1.0 in $CHCl_3$).

S-cyclopentyl (*R*)-4-(benzyloxy)-2',6-dimethyl-6'-((4-methylphenyl)sulfonamido)-[1,1'-biphenyl]-2-carbothioate (**6k**)

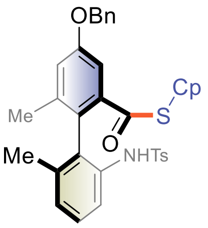

6k

White solid, 52.7 mg, 90% yield, 95% ee. **^{1}H NMR** (400 MHz, $CDCl_3$) δ 7.72 (d, *J* = 8.3 Hz, 2H), 7.55 – 7.51 (m, 2H), 7.49 – 7.44 (m, 2H), 7.43 – 7.39 (m, 1H), 7.37 (d, *J* = 8.9 Hz, 1H), 7.28 (d, *J* = 2.7 Hz, 1H), 7.22 (d, *J* = 8.1 Hz, 2H), 7.16 (t, *J* = 7.9 Hz, 1H), 7.02 (d, *J* = 2.6 Hz, 1H), 6.95 (d, *J* = 7.5 Hz, 1H), 6.38 (s, 1H), 5.15 (s, 2H), 3.60 (p, *J* = 7.1 Hz, 1H), 2.39 (s, 3H), 2.01 (dt, *J* = 13.4, 6.7 Hz, 1H), 1.97 – 1.88 (m, 1H), 1.85 (s, 3H), 1.69 – 1.62 (m, 2H), 1.61 (s, 3H), 1.60 – 1.52 (m, 2H), 1.45 (q, *J* = 6.5 Hz, 1H), 1.39 – 1.32 (m, 1H). **^{13}C NMR** (101 MHz, $CDCl_3$) δ 194.3, 158.4, 143.6, 140.4, 140.4, 137.7, 137.5, 136.4, 135.2, 129.6, 128.8, 128.8, 128.3, 128.2, 127.8, 127.4, 125.6, 124.9, 120.0, 116.3, 111.9, 70.3, 43.2, 33.1, 32.6, 24.6, 24.6, 21.6, 20.2, 19.6. **HRMS** (ESI-TOF) $[M+Na]^+$ calculated for $[C_{34}H_{35}NNaO_4S_2]^+$ 608.1900, found 608.1910.

HPLC (Chiralpak-IB-H column, Hexane/Ethanol = 90/10, flow rate: 1.2 mL/min): t_{major} = 6.174 min; t_{minor} = 7.053 min. **Specific Rotation** $[\alpha]_D^{25}$ = –25.8 (c = 1.0 in $CHCl_3$).

S-benzyl (*R*)-2',4',6-trimethyl-6'-((4-methylphenyl)sulfonamido)-[1,1'-biphenyl]-2-carbothioate (**6l**)

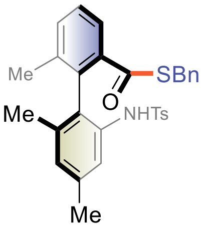

6l

White solid, 50.5 mg, 98% yield, 91% ee. ^{1}**H NMR** (400 MHz, $CDCl_3$) δ 7.70 (d, *J* = 8.4 Hz, 2H), 7.63 (dd, *J* = 6.3, 2.8 Hz, 1H), 7.39 (d, *J* = 6.1 Hz, 2H), 7.25 – 7.18 (m, 6H), 7.14 – 7.06 (m, 2H), 6.80 – 6.75 (m, 1H), 6.27 (s, 1H), 4.05 (s, 2H), 2.39 (s, 3H), 2.31 (s, 3H), 1.75 (s, 3H), 1.66 (s, 3H). ^{13}**C NMR** (101 MHz, $CDCl_3$) δ 193.3, 143.6, 139.2, 139.2, 138.2, 137.6, 137.3, 136.7, 134.7, 134.0, 133.1, 129.6, 128.7, 128.6, 128.5, 127.4, 127.2, 126.9, 126.3, 125.9, 117.5, 33.9, 21.6, 21.6, 20.1, 19.4. **HRMS** (ESI-TOF) $[M+Na]^+$ calculated for $[C_{30}H_{29}NNaO_3S_2]^+$ 538.1481, found 538.1491. **HPLC** (Chiralpak-AD-H column, Hexane/Ethanol = 98/2, flow rate: 1.5 mL/min): t_{major} = 20.038 min; t_{minor} = 16.112 min. **Specific Rotation** $[\alpha]_D^{25}$ = –20.5 (c = 1.0 in $CHCl_3$).

S-benzyl (*S*)-2',4'-dimethoxy-6-methyl-6'-((4-methylphenyl)sulfonamido)-[1,1'-biphenyl]-2-carbothioate (**6m**)

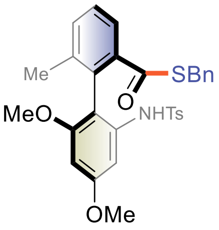

6m

White solid, 44.9 mg, 82% yield, 94% ee. ^{1}**H NMR** (400 MHz, $CDCl_3$) δ 7.69 (d, *J* = 8.3 Hz, 2H), 7.59 (dd, *J* = 5.8, 3.3 Hz, 1H), 7.38 – 7.33 (m, 2H), 7.23 – 7.14 (m, 5H), 7.10 (dd, *J* = 6.8, 2.9 Hz, 2H), 6.79 (d, *J* = 2.3 Hz, 1H), 6.34 (s, 1H), 6.18 (d, *J* = 2.3 Hz, 1H), 4.10 – 3.98 (m, 2H), 3.80 (s, 3H), 3.50 (s, 3H), 2.38 (s, 3H), 1.71 (s, 3H). ^{13}**C NMR** (101 MHz, $CDCl_3$) δ 193.5, 160.8, 157.8, 143.8, 140.2, 140.0, 137.6, 137.1, 136.3, 133.6, 130.0, 129.6, 128.8, 128.5, 128.5, 127.6, 127.2, 125.7, 110.6, 96.3, 94.9,

55.7, 55.4, 33.8, 21.6, 19.5. **HRMS** (ESI-TOF) $[M+Na]^+$ calculated for $[C_{30}H_{29}NNaO_5S_2]^+$ 570.1379, found 570.1381. **HPLC** (Chiralpak-IA-H column, Hexane/Ethanol = 95/5, flow rate: 1.5 mL/min): t_{major} = 16.818 min; t_{minor} = 20.206 min. **Specific Rotation** $[\alpha]_D^{25}$ = –22.5 (c = 1.0 in $CHCl_3$). Note: The sample was dissolved in isopropanol for chiral-HPLC.

S-(4-methoxybenzyl) (*S*)-6-methoxy-2'-methyl-6'-((4-methylphenyl)sulfonamido)-[1,1'-biphenyl]-2-carbothioate (**6n**)

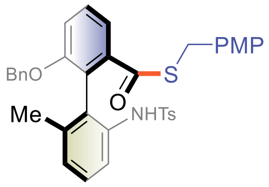

6n

Colorless oil, 19.1 mg, 70% yield, 96% ee. **^{1}H NMR** (400 MHz, $CDCl_3$) δ 7.61 (d, *J* = 8.2 Hz, 2H), 7.48 – 7.40 (m, 3H), 7.27 – 7.20 (m, 4H), 7.11 (dd, *J* = 7.2, 2.2 Hz, 1H), 7.06 (d, *J* = 8.6 Hz, 2H), 7.02 – 6.95 (m, 5H), 6.79 (d, *J* = 8.7 Hz, 2H), 6.50 (s, 1H), 4.87 (q, *J* = 12.6 Hz, 2H), 4.03 (d, *J* = 1.7 Hz, 2H), 3.81 (s, 3H), 2.24 (s, 3H), 1.80 (s, 3H). **^{13}C NMR** (101 MHz, $CDCl_3$) δ 192.7, 158.8, 156.1, 143.1, 140.0, 138.0, 137.2, 136.3, 135.3, 130.0, 129.9, 129.3, 129.1, 128.4, 128.3, 127.6, 127.3, 126.7, 126.0, 125.7, 123.1, 120.6, 117.1, 116.1, 114.0, 69.9, 55.3, 33.5, 21.4, 20.3. **HRMS** (ESI-TOF) $[M+Na]^+$ calculated for $[C_{36}H_{33}NO_5S_2Na]^+$ 646.1692, found 646.1688. **HPLC** (Chiralpak-AS-H column, Hexane/^{i}PrOH = 70/30, flow rate: 1.0 mL/min): t_{major} = 12.287 min; t_{minor} = 10.123 min. **Specific Rotation** $[\alpha]_D^{25}$ = +0.8 (c = 0.1 in $CHCl_3$).

S-(4-methoxybenzyl) (*R*)-2',6-dimethoxy-6'-((4-methylphenyl)sulfonamido)-[1,1'-biphenyl]-2-carbothioate (**6o**)

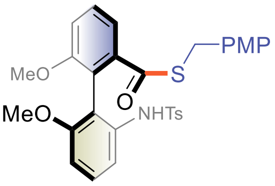

6o

Colorless oil, 16.9 mg, 60% yield, 89% ee. **^{1}H NMR** (400 MHz, $CDCl_3$) δ 7.63 (d, 2H), 7.45 (t, *J* = 7.9 Hz, 1H), 7.39 (dd, *J* = 7.8, 1.2 Hz, 1H), 7.27 – 7.21 (m, 2H), 7.16 – 7.05 (m, 5H), 6.81 – 6.74 (m, 2H), 6.61 (dd, *J* = 7.8, 1.5 Hz, 1H), 6.47 (s, 1H), 3.98 (s, 2H), 3.81 (s, 3H), 3.67 (s, 3H), 3.49 (s, 3H), 2.35 (s, 3H). **^{13}C NMR** (101 MHz, $CDCl_3$) δ

192.1, 158.7, 157.1, 156.9, 143.2, 140.5, 137.3, 136.2, 130.0, 129.9, 129.6, 129.4, 129.3, 127.4, 120.6, 119.5, 116.0, 114.6, 113.9, 112.7, 107.0, 56.2, 55.6, 55.3, 33.2, 21.5. **HRMS** (ESI-TOF) $[M+Na]^+$ calculated for $[C_{30}H_{29}NO_6S_2Na]^+$ 586.1329, found 586.1335. **HPLC** (Chiralpak-AD-H column, Hexane/^{i}PrOH = 70/30, flow rate: 1.0 mL/min): t_{major} = 26.920 min; t_{minor} = 14.380 min. **Specific Rotation** $[\alpha]_D^{25}$ = +31.9 (c = 0.1 in $CHCl_3$).

S-cyclopentyl (*R*)-3-methyl-2-(2-((4-methylphenyl)sulfonamido)naphthalen-1-yl)benzothioate (**6p**)

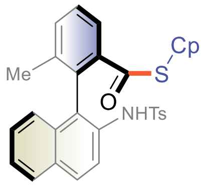

6p

White solid, 44.3 mg, 86% yield, 93% ee. **^{1}H NMR** (400 MHz, $CDCl_3$) δ 7.81 (d, *J* = 1.8 Hz, 2H), 7.79 – 7.75 (m, 1H), 7.72 – 7.67 (m, 3H), 7.47 (t, *J* = 7.6 Hz, 1H), 7.43 – 7.39 (m, 1H), 7.34 (ddd, *J* = 8.1, 6.8, 1.3 Hz, 1H), 7.27 (ddd, *J* = 8.3, 6.7, 1.4 Hz, 1H), 7.22 – 7.17 (m, 2H), 6.95 (dd, *J* = 8.5, 1.2 Hz, 1H), 6.70 (s, 1H), 3.49 – 3.40 (m, 1H), 2.37 (s, 3H), 1.92 (dtd, *J* = 13.4, 7.5, 5.7 Hz, 1H), 1.74 – 1.65 (m, 1H), 1.59 – 1.51 (m, 1H), 1.46 (s, 3H), 1.42 – 1.33 (m, 2H), 1.10 – 1.02 (m, 1H). **^{13}C NMR** (101 MHz, $CDCl_3$) δ 194.8, 143.7, 140.3, 139.8, 137.8, 133.7, 132.5, 132.3, 131.5, 130.7, 129.7, 129.1, 128.8, 128.0, 127.3, 126.8, 125.8, 125.4, 125.0, 125.0, 119.4, 43.2, 32.9, 32.4, 24.6, 24.4, 21.6, 19.4. **HRMS** (ESI-TOF) $[M+Na]^+$ calculated for $[C_{30}H_{29}NO_3S_2Na]^+$ 538.1481, found 538.1488. **HPLC** (Chiralpak-IA-H column, Hexane/Ethanol = 97/3, flow rate: 1.2 mL/min): t_{major} = 14.920 min; t_{minor} = 16.254 min. **Specific Rotation** $[\alpha]_D^{25}$ = +16.5 (c = 1.0 in $CHCl_3$).

S-benzyl (*S*)-1-(2,4-dimethoxy-6-((4-methylphenyl)sulfonamido)phenyl)naphthalene-2-carbothioate (**6q**)

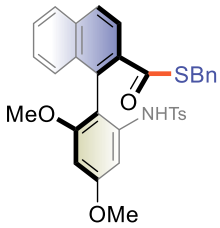

6q

White solid, 52.5 mg, 90% yield, 93% ee. **^{1}H NMR** (400 MHz, $CDCl_3$) δ 7.93 (d, *J* = 8.6 Hz, 1H), 7.86 (d, *J* = 8.2 Hz, 1H), 7.80 (d, *J* = 8.6 Hz, 1H), 7.50 (ddd, *J* = 8.1, 6.8, 1.2 Hz, 1H), 7.40 (d, *J* = 8.3 Hz, 2H), 7.26 – 7.21 (m, 3H), 7.19 – 7.11 (m, 3H), 7.03 – 6.96 (m, 4H), 6.30 (s, 1H), 6.24 (d, *J* = 2.2 Hz, 1H), 4.10 (d, *J* = 1.9 Hz, 2H), 3.87 (s, 3H), 3.39 (s, 3H), 2.32 (s, 3H). **^{13}C NMR** (101 MHz, $CDCl_3$) δ 193.5, 161.3, 158.6, 143.5, 137.5, 137.0, 137.0, 136.5, 134.7, 132.5, 129.4, 129.2, 129.2, 128.8, 128.5, 128.2, 127.5, 127.4, 127.3, 127.2, 126.4, 124.0, 109.3, 96.8, 95.1, 55.7, 55.5, 33.9, 21.6. **HRMS** (ESI-TOF) $[M+Na]^+$ calculated for $[C_{33}H_{29}NNaO_5S_2]^+$ 606.1379, found 570.1388. **HPLC** (Chiralpak-IB-H column, Hexane/Ethanol = 98/2, flow rate: 1.5 mL/min): t_{major} = 21.568 min; t_{minor} = 20.292 min. **Specific Rotation** $[\alpha]_D^{25}$ = +22.1 (c = 1.0 in $CHCl_3$). Note: The sample was dissolved in isopropanol for chiral-HPLC.

S-cyclopentyl (*R*)-1-(2-methyl-6-((4-methylphenyl)sulfonamido)phenyl)naphthalene-2-carbothioate (**6r**)

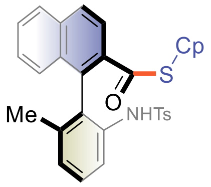

6r

White solid, 38.1 mg, 74% yield, 93% ee. **^{1}H NMR** (400 MHz, $CDCl_3$) δ 7.96 (d, *J* = 8.6 Hz, 1H), 7.87 (d, *J* = 8.2 Hz, 1H), 7.82 (d, *J* = 8.6 Hz, 1H), 7.60 (d, *J* = 8.2 Hz, 1H), 7.50 (ddd, *J* = 8.1, 6.8, 1.2 Hz, 1H), 7.31 (dd, *J* = 12.0, 8.1 Hz, 3H), 7.09 (ddd, *J* = 8.3, 6.8, 1.3 Hz, 1H), 7.03 (d, *J* = 7.6 Hz, 1H), 6.94 (d, *J* = 8.0 Hz, 2H), 6.83 – 6.78 (m, 1H), 6.36 (s, 1H), 3.69 (p, *J* = 7.1 Hz, 1H), 2.32 (s, 3H), 2.06 (dq, *J* = 13.5, 7.2 Hz, 1H), 2.00 – 1.91 (m, 1H), 1.69 (s, 3H), 1.64 (ddd, *J* = 18.8, 9.5, 4.1 Hz, 3H), 1.56 – 1.45 (m, 2H), 1.40 – 1.32 (m, 1H). **^{13}C NMR** (101 MHz, $CDCl_3$) δ 194.9, 143.2, 138.3, 136.9, 136.6, 135.5, 134.7, 131.7, 131.6, 129.3, 129.2, 128.8, 128.5, 128.2, 127.6, 127.5, 127.1, 126.2, 126.1, 124.0, 118.0, 43.5, 33.1, 32.8, 24.7, 24.7, 21.6, 20.2. **HRMS** (ESI-TOF) $[M+Na]^+$ calculated for $[C_{30}H_{29}NNaO_3S_2]^+$ 538.1481, found 538.1487. **HPLC** (Chiralpak-IA-H column, Hexane/Ethanol = 95/5, flow rate: 1.2 mL/min): t_{major} = 13.009 min; t_{minor} = 14.375 min. **Specific Rotation** $[\alpha]_D^{25}$ = +47.3 (c = 1.0 in $CHCl_3$).

S-cyclopentyl (*S*)-3-methoxy-2-(2-methyl-6-((4-methylphenyl)sulfonamido)phenyl)naphthalene-1-carbothioate (**6s**)

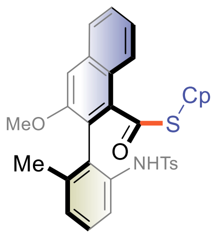

6s

White solid, 46.9 mg, 86% yield, 99% ee. **^{1}H NMR** (400 MHz, $CDCl_3$) δ 7.82 (t, *J* = 8.2 Hz, 2H), 7.61 (d, *J* = 8.2 Hz, 2H), 7.58 – 7.53 (m, 1H), 7.47 – 7.39 (m, 2H), 7.21 (t, *J* = 7.9 Hz, 1H), 7.15 (d, *J* = 7.5 Hz, 3H), 7.00 (d, *J* = 7.6 Hz, 1H), 6.62 (s, 1H), 3.80 – 3.73 (m, 1H), 3.61 (s, 3H), 2.39 (s, 3H), 1.95 (s, 3H), 1.94 – 1.87 (m, 1H), 1.81 (dq, *J* = 13.8, 7.0 Hz, 1H), 1.49 (ddp, *J* = 19.4, 6.7, 4.1 Hz, 4H), 1.27 – 1.21 (m, 1H), 1.03 (dq, *J* = 13.2, 6.6 Hz, 1H). **^{13}C NMR** (101 MHz, $CDCl_3$) δ 197.0, 154.2, 143.0, 139.4, 139.0, 137.7, 135.1, 134.8, 129.3, 128.8, 127.5, 127.3, 126.9, 126.7, 126.0, 125.0, 124.9, 124.7, 123.4, 118.4, 107.5, 55.7, 43.5, 32.8, 32.3, 24.6, 24.5, 21.6, 20.5. **HRMS** (ESI-TOF) $[M+Na]^+$ calculated for $[C_{31}H_{31}NNaO_4S_2]^+$ 568.1587, found 568.1594. **HPLC** (Chiralpak-AS-H column, Hexane/Ethanol = 95/5, flow rate: 1.2 mL/min): t_{major} = 19.259 min; t_{minor} = 15.863 min. **Specific Rotation** $[\alpha]_D^{25}$ = –82.0 (c = 1.0 in $CHCl_3$).

(*R*)-2-((cyclopentylthio)carbonyl)-2',6-dimethyl-6'-((4-methylphenyl)sulfonamido)-[1,1'-biphenyl]-4-yl 2-(1-(4-chlorobenzoyl)-5-methoxy-2-methyl-1H-indol-3-yl)acetate (**6t**)

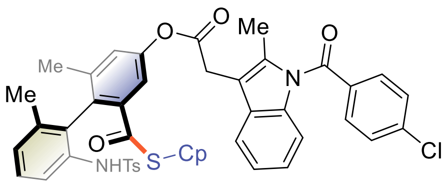

6t

White solid, 67.6 mg, 81% yield, 87% ee. **^{1}H NMR** (400 MHz, $CDCl_3$) δ 7.73 – 7.67 (m, 4H), 7.50 (d, *J* = 8.4 Hz, 2H), 7.39 (s, 1H), 7.34 (d, *J* = 8.1 Hz, 1H), 7.22 (d, *J* = 8.6 Hz, 2H), 7.16 (t, *J* = 7.9 Hz, 1H), 7.11 (d, *J* = 11.7 Hz, 2H), 6.94 (dd, *J* = 11.1, 8.5 Hz, 2H), 6.75 – 6.70 (m, 1H), 6.36 (s, 1H), 3.97 (s, 2H), 3.89 (d, *J* = 1.6 Hz, 3H), 3.63 – 3.53 (m, 1H), 2.51 (d, *J* = 1.5 Hz, 3H), 2.38 (s, 3H), 2.04 – 1.97 (m, 1H), 1.94 – 1.85 (m, 1H), 1.81 (d, *J* = 1.5 Hz, 3H), 1.61 (d, *J* = 1.5 Hz, 3H), 1.60 – 1.48 (m, 3H), 1.43 (dt, *J* = 13.4, 6.4 Hz, 1H), 1.31 (dd, *J* = 14.1, 7.6 Hz, 2H). **^{13}C NMR** (101 MHz, $CDCl_3$) δ 193.8, 168.9, 168.4, 156.3, 150.2, 143.6, 140.7, 140.4, 139.5, 137.6, 137.3, 136.4,

135.0, 133.8, 131.3, 130.9, 130.6, 130.5, 129.6, 129.3, 128.8, 128.6, 127.4, 126.4, 126.1, 118.7, 117.3, 115.2, 111.9, 111.7, 101.2, 55.9, 43.4, 33.1, 32.6, 30.6, 24.7, 24.6, 21.6, 20.2, 19.5, 13.5. **HRMS** (ESI-TOF) $[M+Na]^+$ calculated for $[C_{46}H_{43}ClN_2NaO_7S_2]^+$ 857.2092, found 857.2101. **HPLC** (Chiralpak-IB-H column, Hexane/Ethanol = 90/10, flow rate: 0.5 mL/min): t_{major} = 46.805 min; t_{minor} = 44.212 min. **Specific Rotation** $[\alpha]_D^{25}$ = –20.3 (c = 1.0 in $CHCl_3$).

(*R*)-2-((cyclopentylthio)carbonyl)-2',6-dimethyl-6'-((4-methylphenyl)sulfonamido)-[1,1'-biphenyl]-4-yl 2-(11-oxo-6,11-dihydrodibenzo[b,e]oxepin-2-yl)acetate (**6u**)

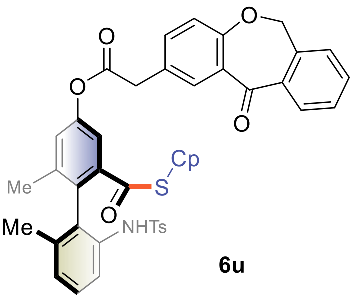

6u

White solid, 59.6 mg, 80% yield, 87% ee. **^{1}H NMR** (400 MHz, $CDCl_3$) δ 8.27 (s, 1H), 7.93 (d, *J* = 7.8 Hz, 1H), 7.68 (d, *J* = 6.4 Hz, 2H), 7.58 (d, *J* = 7.9 Hz, 2H), 7.50 (t, *J* = 7.6 Hz, 1H), 7.42 – 7.34 (m, 3H), 7.22 (d, *J* = 6.5 Hz, 2H), 7.20 – 7.10 (m, 3H), 6.95 (d, *J* = 7.6 Hz, 1H), 6.40 (s, 1H), 5.24 (s, 2H), 3.95 (s, 2H), 3.62 – 3.54 (m, 1H), 2.38 (s, 3H), 2.05 – 1.98 (m, 1H), 1.94 – 1.86 (m, 1H), 1.81 (s, 3H), 1.65 – 1.61 (m, 1H), 1.59 (s, 3H), 1.53 (dd, *J* = 13.3, 7.0 Hz, 2H), 1.47 – 1.40 (m, 1H), 1.32 (dd, *J* = 13.4, 6.9 Hz, 2H). **^{13}C NMR** (101 MHz, $CDCl_3$) δ 193.9, 190.9, 169.5, 160.8, 150.2, 143.6, 140.6, 140.5, 140.4, 137.7, 137.3, 136.4, 135.6, 135.0, 133.0, 132.8, 130.5, 129.6, 129.6, 129.4, 129.0, 128.6, 127.9, 127.4, 126.9, 126.4, 126.1, 125.4, 121.5, 118.8, 117.5, 73.7, 43.4, 40.4, 33.1, 32.6, 24.7, 24.6, 21.6, 20.2, 19.4. **HRMS** (ESI-TOF) $[M+Na]^+$ calculated for $[C_{43}H_{39}NNaO_7S_2]^+$ 768.2060, found 768.2066. **HPLC** (Chiralpak-OD-H column, Hexane/Ethanol = 95/5, flow rate: 0.8 mL/min): t_{major} = 100.178 min; t_{minor} = 91.106 min. **Specific Rotation** $[\alpha]_D^{25}$ = –18.8 (c = 1.0 in $CHCl_3$).

(*R*)-2-((cyclopentylthio)carbonyl)-2',6-dimethyl-6'-((4-methylphenyl)sulfonamido)-[1,1'-biphenyl]-4-yl 5-(2,5-dimethylphenoxy)-2,2-dimethylpentanoate (**6v**)

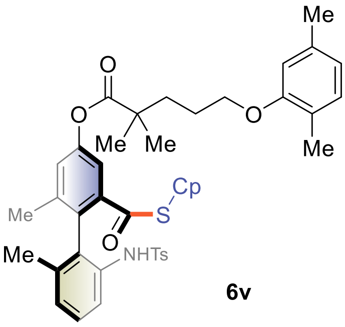

6v

White solid, 67.6 mg, 93% yield, 90% ee. **^{1}H NMR** (400 MHz, $CDCl_3$) δ 7.71 (d, *J* = 8.3 Hz, 2H), 7.40 – 7.34 (m, 2H), 7.24 (d, *J* = 8.1 Hz, 2H), 7.17 (t, *J* = 7.9 Hz, 1H), 7.11 (d, *J* = 2.4 Hz, 1H), 7.03 (d, *J* = 7.3 Hz, 1H), 6.96 (d, *J* = 7.6 Hz, 1H), 6.68 (d, *J* = 8.4 Hz, 2H), 6.40 (s, 1H), 4.04 (t, *J* = 5.1 Hz, 2H), 3.59 (p, *J* = 7.1 Hz, 1H), 2.40 (s, 3H), 2.33 (s, 3H), 2.22 (s, 3H), 2.04 – 1.98 (m, 1H), 1.95 (s, 4H), 1.92 – 1.87 (m, 1H), 1.84 (s, 3H), 1.63 (s, 3H), 1.56 (dddt, *J* = 11.8, 8.5, 5.9, 2.5 Hz, 3H), 1.44 (s, 7H), 1.34 (dd, *J* = 13.6, 6.7 Hz, 2H). **^{13}C NMR** (101 MHz, $CDCl_3$) δ 193.9, 176.0, 157.0, 150.6, 143.6, 140.5, 140.4, 137.7, 137.4, 136.6, 135.1, 130.4, 130.2, 129.6, 128.9, 128.5, 127.4, 126.5, 126.0, 123.6, 120.9, 118.8, 117.2, 112.0, 67.8, 43.4, 42.7, 37.2, 33.0, 32.6, 25.4, 25.2, 25.2, 24.7, 24.6, 21.6, 21.5, 20.2, 19.4, 15.9. **HRMS** (ESI-TOF) $[M+Na]^+$ calculated for $[C_{42}H_{49}NNaO_6S_2]^+$ 750.2894, found 750.2899. **HPLC** (Chiralpak-IA-H column, Hexane/Ethanol = 90/10, flow rate: 1.2 mL/min): t_{major} = 5.261 min; t_{minor} = 6.964 min. **Specific Rotation** $[\alpha]_D^{25}$ = –18.0 (c = 1.0 in $CHCl_3$).

(*R*)-2-((cyclopentylthio)carbonyl)-2',6-dimethyl-6'-((4-methylphenyl)sulfonamido)-[1,1'-biphenyl]-4-yl 4,7,7-trimethyl-3-oxo-2-oxabicyclo[2.2.1]heptane-1-carboxylate (**6w**)

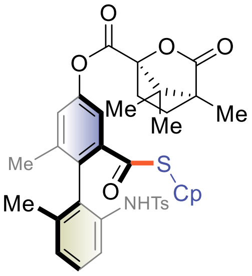

6w

White solid, 50.0 mg, 74% yield, 88% de. **^{1}H NMR** (400 MHz, $CDCl_3$) δ 7.70 (d, *J* = 8.3 Hz, 2H), 7.43 (d, *J* = 2.4 Hz, 1H), 7.35 (d, *J* = 8.2 Hz, 1H), 7.24 (d, *J* = 8.1 Hz, 2H), 7.21 (d, *J* = 2.4 Hz, 1H), 7.18 (t, *J* = 7.9 Hz, 1H), 6.97 (d, *J* = 7.6 Hz, 1H), 6.36 (s, 1H), 3.60 (p, *J* = 7.1 Hz, 1H), 2.62 (ddd, *J* = 13.5, 10.7, 4.2 Hz, 1H), 2.40 (s, 3H), 2.26 (ddd, *J* = 13.6, 9.3, 4.6 Hz, 1H), 2.07 – 1.98 (m, 2H), 1.94 – 1.86 (m, 1H), 1.84 (s, 3H), 1.80

(dd, J = 9.2, 4.1 Hz, 1H), 1.65 (s, 3H), 1.62 (dd, J = 6.3, 2.7 Hz, 1H), 1.54 (td, J = 7.7, 7.2, 5.3 Hz, 2H), 1.48 – 1.41 (m, 1H), 1.36 – 1.29 (m, 2H), 1.21 (d, J = 3.2 Hz, 6H), 1.17 (s, 3H). **^{13}C NMR** (101 MHz, $CDCl_3$) δ 193.9, 177.8, 165.8, 149.5, 143.7, 141.0, 140.7, 137.7, 137.3, 135.0, 131.2, 129.7, 128.8, 128.7, 127.4, 126.2, 126.1, 118.5, 117.6, 90.8, 55.0, 54.9, 43.5, 33.1, 32.7, 30.9, 29.1, 24.7, 24.7, 21.6, 20.2, 19.5, 17.1, 17.0, 9.8. **HRMS** (ESI-TOF) $[M+Na]^+$ calculated for $[C_{37}H_{41}NNaO_7S_2]^+$ 698.2217, found 698.2225. **HPLC** (Chiralpak-IA-H column, Hexane/Ethanol = 70/30, flow rate: 1.2 mL/min): t_{major} = 8.055 min; t_{minor} = 11.427 min. **Specific Rotation** $[\alpha]_D^{25}$ = –14.1 (c = 1.0 in $CHCl_3$).

(*R*)-N-(2-(2-formylnaphthalen-1-yl)-3-methylphenyl)-4-methylbenzenesulfonamide (**7a**)

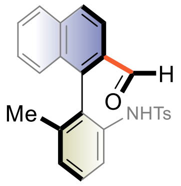

7a

White solid, 36.9 mg, 89% yield, 90% ee, **m.p.** 154-156 °C. **^{1}H NMR** (400 MHz, $CDCl_3$) δ 9.12 (s, 1H), 8.05 – 7.97 (m, 3H), 7.79 (d, J = 8.3 Hz, 1H), 7.68 (ddd, J = 8.1, 6.8, 1.2 Hz, 1H), 7.44 – 7.40 (m, 1H), 7.39 (dd, J = 3.2, 1.7 Hz, 1H), 7.37 – 7.34 (m, 2H), 7.19 (d, J = 8.5 Hz, 1H), 7.13 (dd, J = 8.0, 3.3 Hz, 3H), 5.83 (s, 1H), 2.42 (s, 3H), 1.73 (s, 3H). **^{13}C NMR** (101 MHz, $CDCl_3$) δ 189.9, 143.9, 138.7, 138.1, 135.9, 135.1, 134.8, 131.2, 130.6, 129.2, 129.1, 129.0, 129.0, 128.2, 127.5, 126.3, 125.8, 124.9, 124.6, 121.8, 117.3, 21.0, 19.8. **HRMS** (ESI-TOF) $[M+Na]^+$ calculated for $[C_{25}H_{21}NNaO_3S]^+$ 438.1134, found 438.1138. **HPLC** (Chiralpak-IA-H column, Hexane/Ethanol = 80/20, flow rate: 1.2 mL/min): t_{major} = 14.827 min; t_{minor} = 9.285 min. **Specific Rotation** $[\alpha]_D^{25}$ = +33.8 (c = 1.0 in $CHCl_3$).

(*R*)-N-(2-(2-(hydroxymethyl)naphthalen-1-yl)-3-methylphenyl)-4-methylbenzenesulfonamide (**7b**)

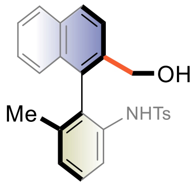

7b

White solid, 39.6 mg, 95% yield, 90% ee. **^{1}H NMR** (400 MHz, $CDCl_3$) δ 7.97 (d, *J* = 8.5 Hz, 1H), 7.89 (d, *J* = 8.2 Hz, 1H), 7.74 (d, *J* = 8.5 Hz, 1H), 7.62 (d, *J* = 8.2 Hz, 1H), 7.47 (ddd, *J* = 8.1, 6.8, 1.2 Hz, 1H), 7.41 – 7.35 (m, 2H), 7.32 (t, *J* = 8.0 Hz, 1H), 7.13 (ddd, *J* = 8.2, 6.8, 1.3 Hz, 1H), 7.09 (d, *J* = 7.5 Hz, 1H), 7.05 (d, *J* = 8.1 Hz, 2H), 6.78 (dd, *J* = 8.6, 1.2 Hz, 1H), 6.33 (s, 1H), 4.40 – 4.22 (m, 2H), 2.38 (s, 3H), 1.94 (s, 1H), 1.73 (s, 3H). **^{13}C NMR** (101 MHz, $CDCl_3$) δ 143.7, 138.5, 137.0, 136.5, 135.2, 133.4, 131.6, 130.7, 129.5, 129.5, 128.9, 128.4, 128.2, 127.2, 127.0, 126.3, 126.2, 126.1, 124.7, 117.5, 63.0, 21.6, 20.1. **HRMS** (ESI-TOF) $[M+Na]^+$ calculated for $[C_{25}H_{23}NNaO_3S]^+$ 440.1291, found 440.1295. **HPLC** (Chiralpak-IA-H column, Hexane/Ethanol = 80/20, flow rate: 1.2 mL/min): t_{major} = 7.569 min; t_{minor} = 6.599 min. **Specific Rotation** $[\alpha]_D^{25}$ = +67.1 (c = 1.0 in $CHCl_3$).

(*R*)-4-methyl-N-(3-methyl-2-(2-(5-methyl-1,3,4-oxadiazol-2-yl)naphthalen-1-yl)phenyl)benzenesulfonamide (**7c**)

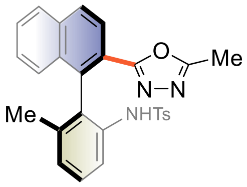

7c

White solid, 35.6 mg, 76% yield, 89% ee, **m.p.** 175-177 °C. **^{1}H NMR** (400 MHz, $CDCl_3$) δ 8.31 (d, *J* = 8.7 Hz, 1H), 8.08 (d, *J* = 8.7 Hz, 1H), 7.98 (d, *J* = 8.2 Hz, 1H), 7.67 (d, *J* = 8.3 Hz, 1H), 7.61 (ddd, *J* = 8.1, 6.8, 1.2 Hz, 1H), 7.37 – 7.28 (m, 4H), 7.11 (d, *J* = 8.4 Hz, 1H), 7.03 (d, *J* = 7.9 Hz, 3H), 5.99 (s, 1H), 2.36 (s, 3H), 2.12 (s, 3H), 1.65 (s, 3H). **^{13}C NMR** (101 MHz, $CDCl_3$) δ 164.4, 163.7, 143.8, 138.1, 136.4, 135.1, 134.9, 133.1, 131.8, 129.9, 129.4, 129.1, 128.6, 128.3, 128.1, 127.9, 127.1, 126.0, 125.8, 125.5, 121.5, 116.9, 21.6, 20.0, 10.6. **HRMS** (ESI-TOF) $[M+Na]^+$ calculated for $[C_{27}H_{23}N_3NaO_3S]^+$ 492.1352, found 492.1353. **HPLC** (Chiralpak-IA-H column, Hexane/Ethanol = 70/30, flow rate: 1.2 mL/min): t_{major} = 9.861 min; t_{minor} = 17.521 min. **Specific Rotation** $[\alpha]_D^{25}$ = –15.5 (c = 1.0 in $CHCl_3$).

2.8 X-ray crystal structure

Supplementary Table 6. X-ray structure data of compound **4h**.

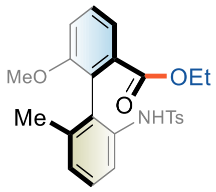

4h
CCDC 2252560

═

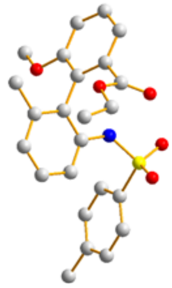

Identification code	CCDC 2252560
Formula	$C_{48}H_{50}N_2O_{10}S_2$
Formula weight	879.02
Temperature/K	295(2)
Crystal system	monoclinic
Space group	$P2_1$
a/Å	8.18710(10)
b/Å	18.96230(10)
c/Å	14.53710(10)
α/°	90
β/°	91.1770(10)
γ/°	90
Volume/Å^3	2256.35(3)
Z	2
ρ_{calc}g/cm^3	1.294
μ/mm^{-1}	1.567
F(000)	928.0
Crystal size/mm^3	0.2 x 0.1 x 0.1
Radiation	CuKα (λ = 1.54184)
2Θ range for data collection/°	6.082 to 154.562
Index ranges	$-10 \leq h \leq 10$, $-23 \leq k \leq 23$, $-18 \leq l \leq 12$
Reflections collected	27326
Independent reflections	9154 [R_{int} = 0.0254, R_{sigma} = 0.0237]
Data/restraints/parameters	9154/1/568

Goodness-of-fit on F^2	1.035
Final R indexes [I>=2σ (I)]	R_1 = 0.0323, wR_2 = 0.0926
Final R indexes [all data]	R_1 = 0.0340, wR_2 = 0.0941
Largest diff. peak/hole / e $Å^{-3}$	0.27 /-0.18
Flack parameter	-0.021 (5)

Supplementary Table 7. X-ray structure data of compound **7b**.

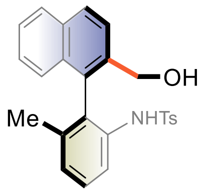

7b
CCDC 22263709

≡

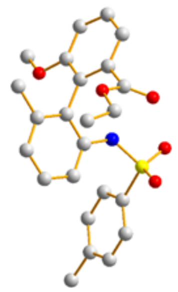

Identification code	CCDC 2263709
Formula	$C_{25}H_{23}NO_3S$
Formula weight	417.50
Temperature/K	100.01(10)
Crystal system	orthorhombic
Space group	$P2_12_12_1$
a/Å	8.5058(2)
b/Å	14.2403(4)
c/Å	17.6990(7)
α/°	90
β/°	90
γ/°	90
Volume/Å^3	2143.79(12)
Z	4
ρ_{calc}g/cm^3	1.294
μ/mm^{-1}	1.552
F(000)	880.0
Crystal size/mm^3	0.3 × 0.04 × 0.02
Radiation	CuKα (λ = 1.54184)
2Θ range for data collection/°	7.968 to 148.282
Index ranges	$-10 \leq h \leq 10, -17 \leq k \leq 9, -21 \leq l \leq 15$
Reflections collected	4969
Independent reflections	3478 [R_{int} = 0.0427, R_{sigma} = 0.0658]
Data/restraints/parameters	3478/0/275
Goodness-of-fit on F^2	0.996

Final R indexes [I>=2σ (I)]	R_1 = 0.0495, wR_2 = 0.1341
Final R indexes [all data]	R_1 = 0.0576, wR_2 = 0.1429
Largest diff. peak/hole / e $Å^{-3}$	0.82/-0.44
Flack parameter	0.00(2)

3. Supplementary Figures

3.1 NMR spectra

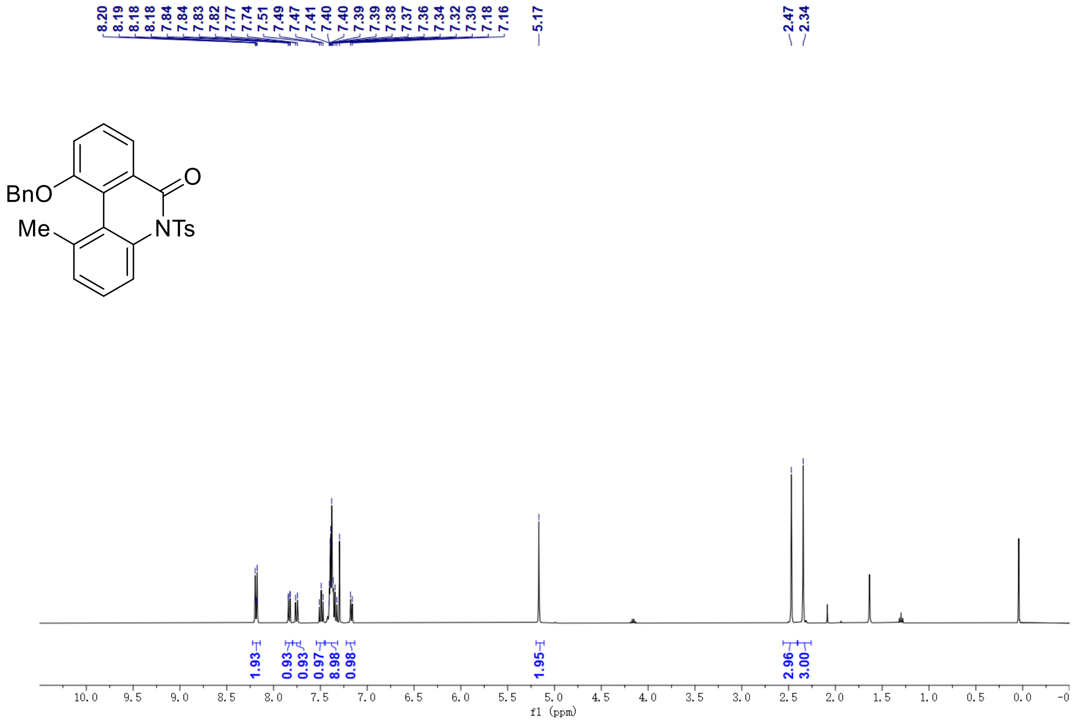

Supplementary Figure 7. ^{1}H NMR of the **1c** (400 MHz, $CDCl_3$)

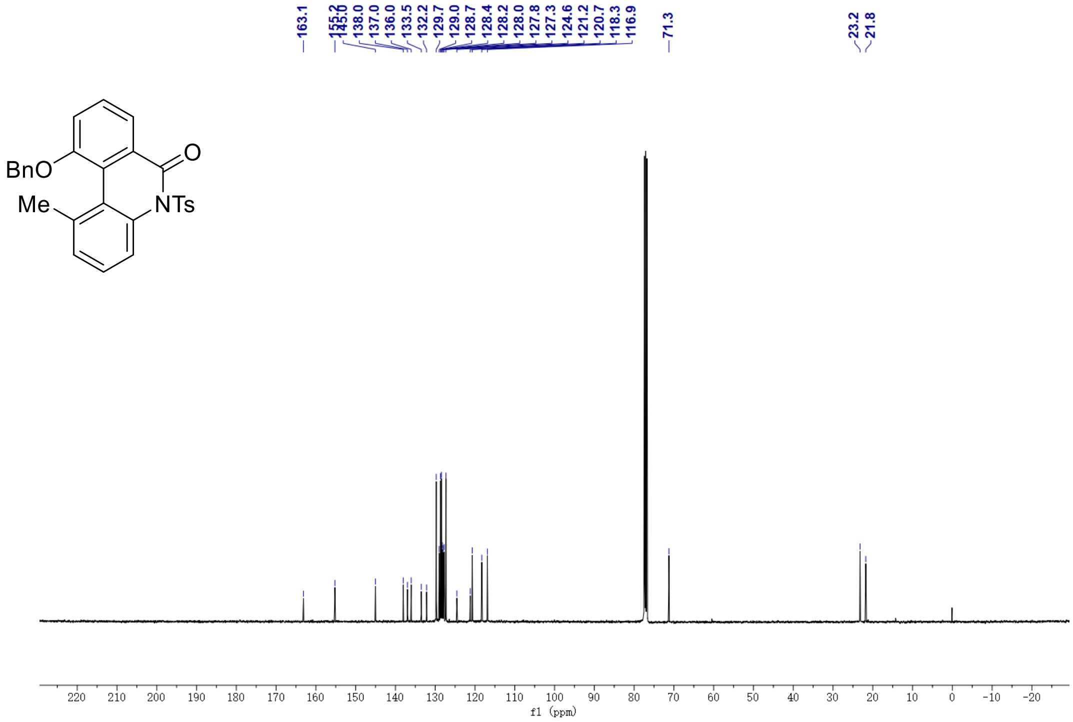

Supplementary Figure 8. ^{13}C NMR of the **1c** (101 MHz, $CDCl_3$)

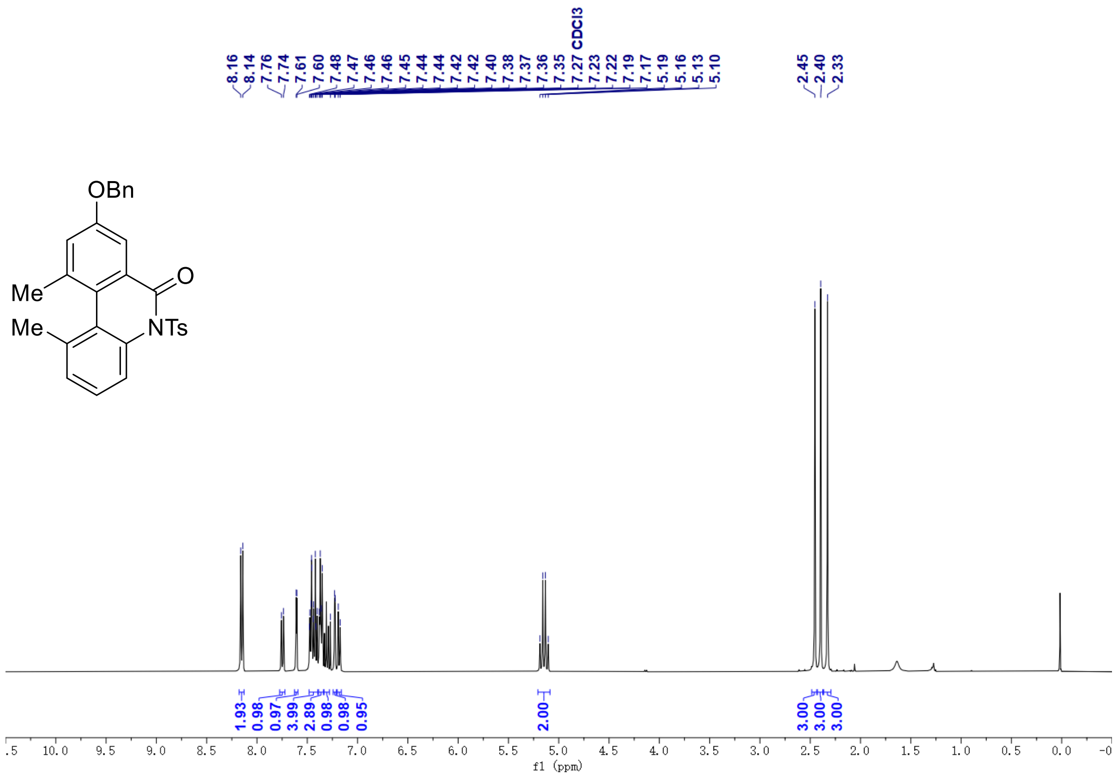

Supplementary Figure 9. ^{1}**H NMR** of the **1g** (400 MHz, $CDCl_3$)

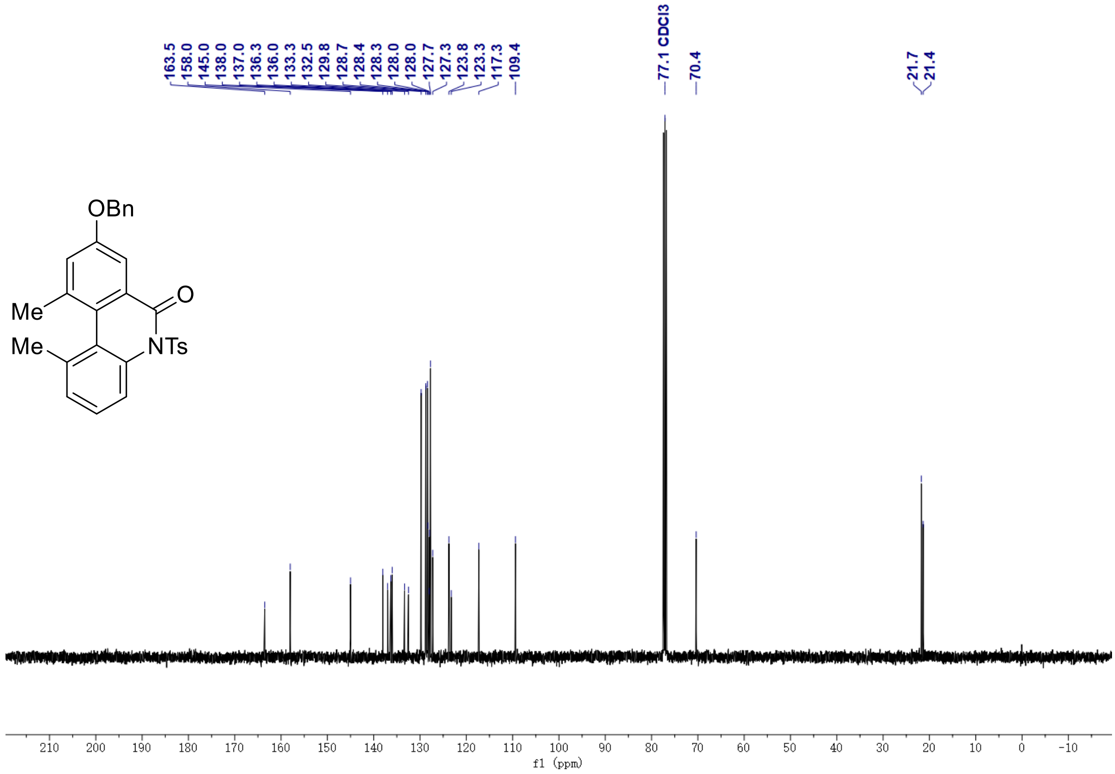

Supplementary Figure 10. ^{13}**C NMR** of the **1g** (101 MHz, $CDCl_3$)

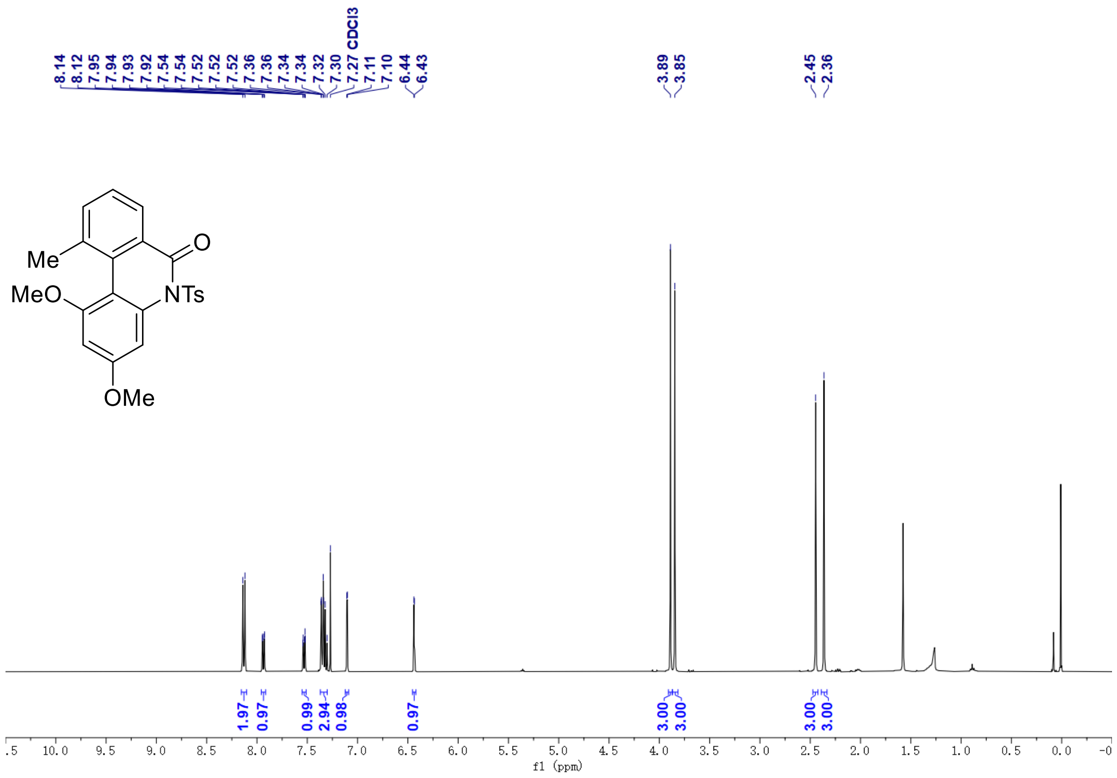

Supplementary Figure 11. ^{1}**H NMR** of the **1i** (400 MHz, $CDCl_3$)

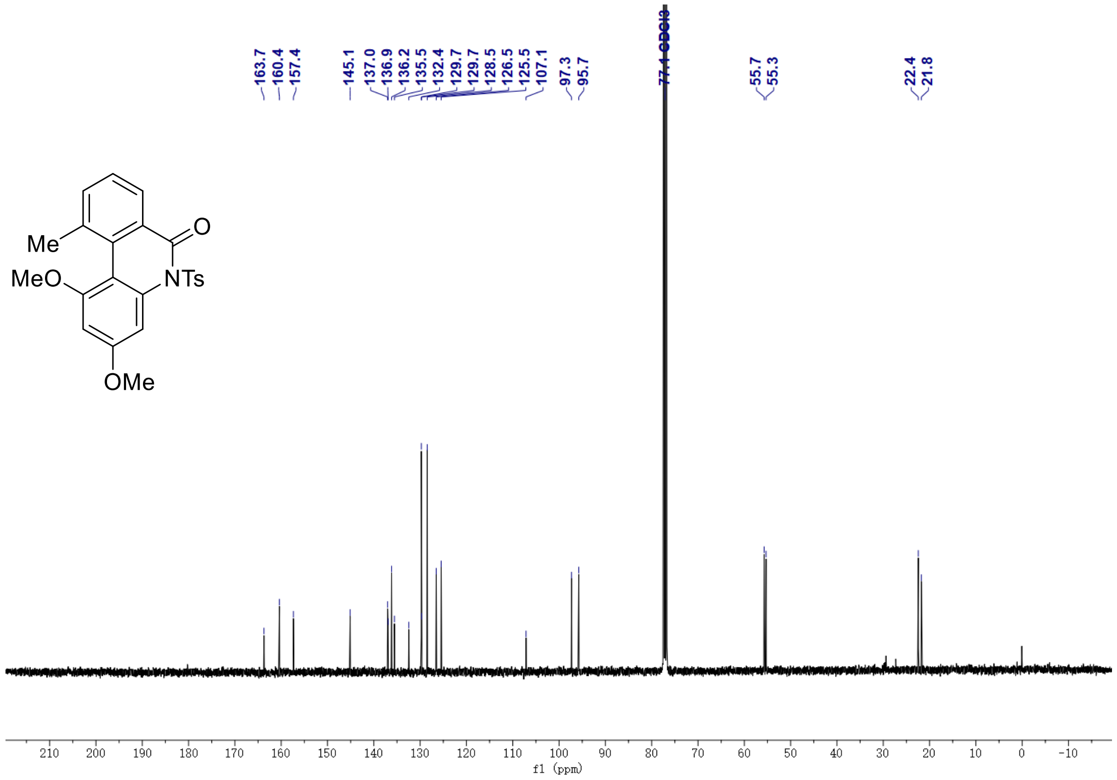

Supplementary Figure 12. ^{13}**C NMR** of the **1i** (101 MHz, $CDCl_3$)

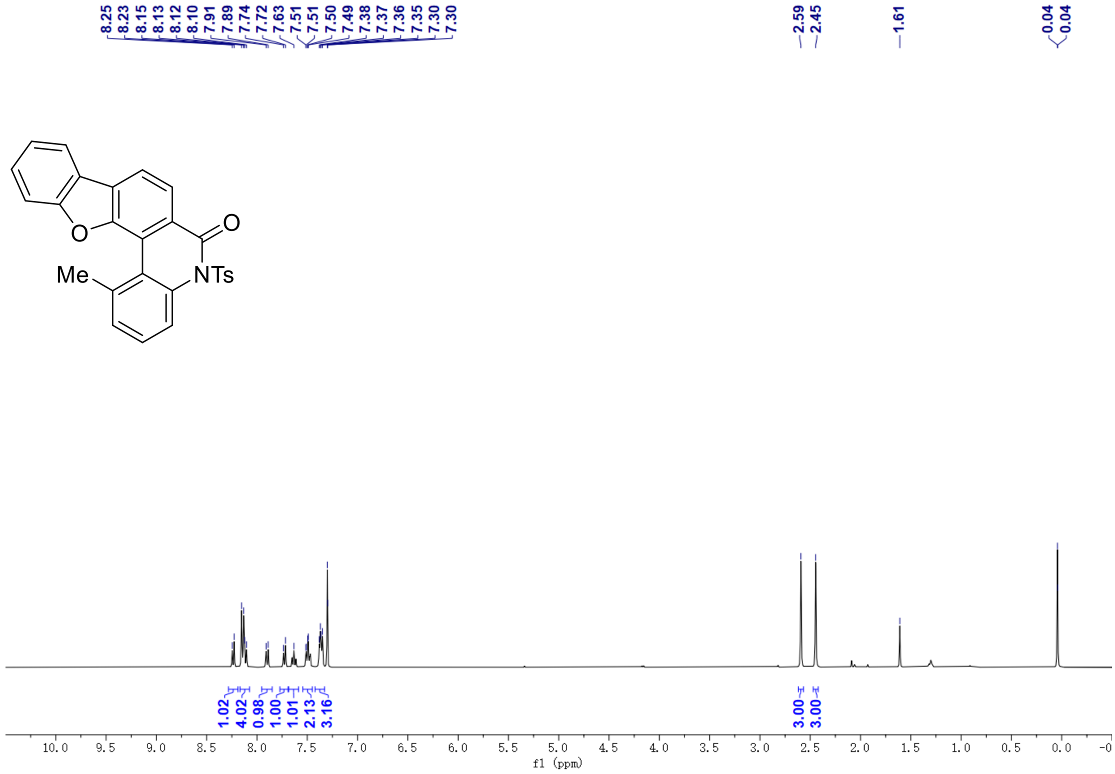

Supplementary Figure 13. ^{1}**H NMR** of the **1l** (400 MHz, $CDCl_3$)

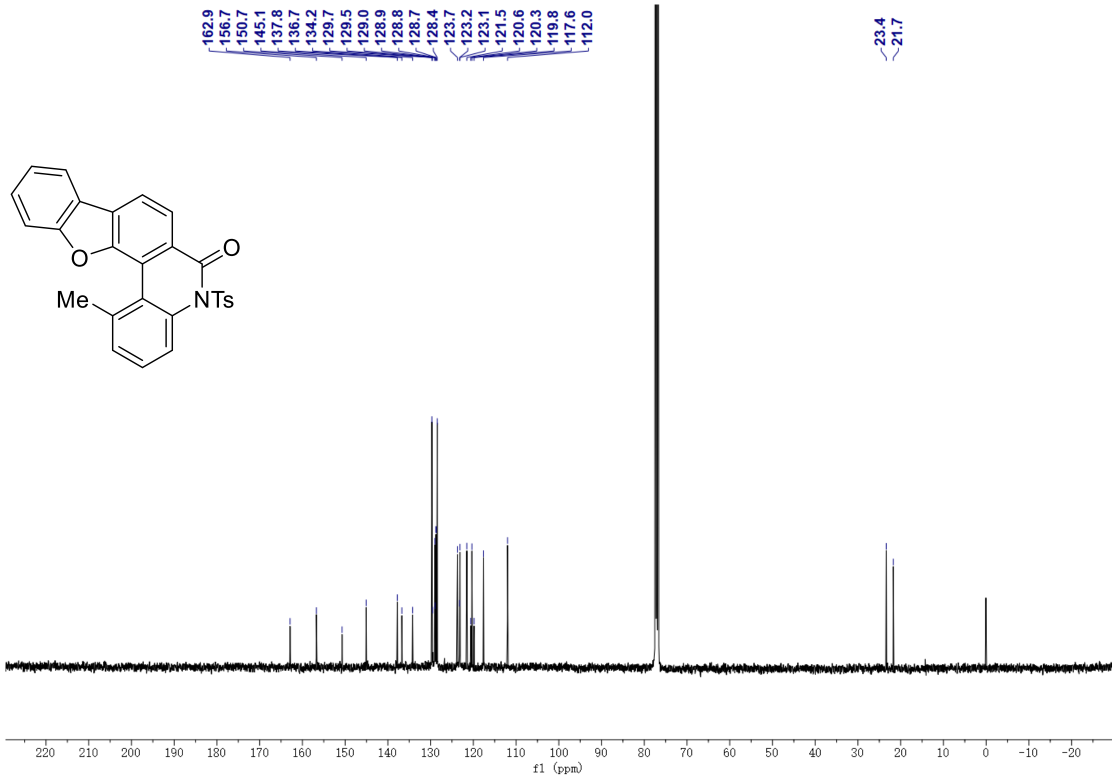

Supplementary Figure 14. ^{13}**C NMR** of the **1l** (101 MHz, $CDCl_3$)

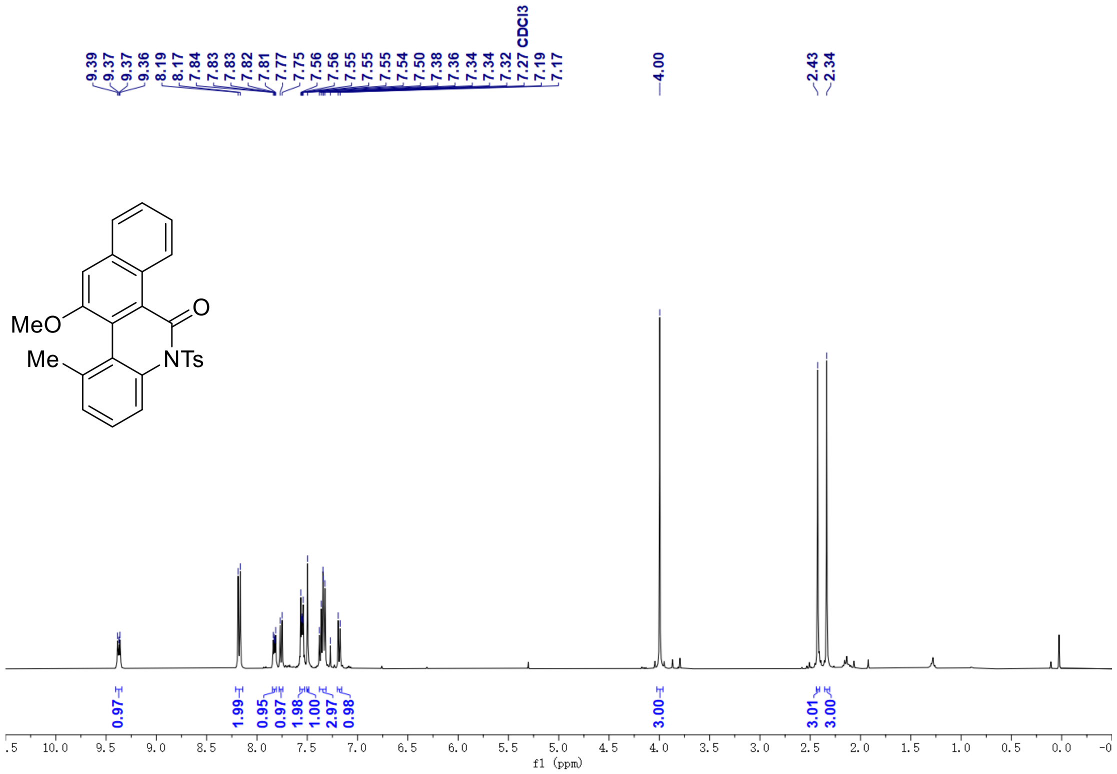

Supplementary Figure 15. ^{1}H NMR of the **1m** (400 MHz, $CDCl_3$)

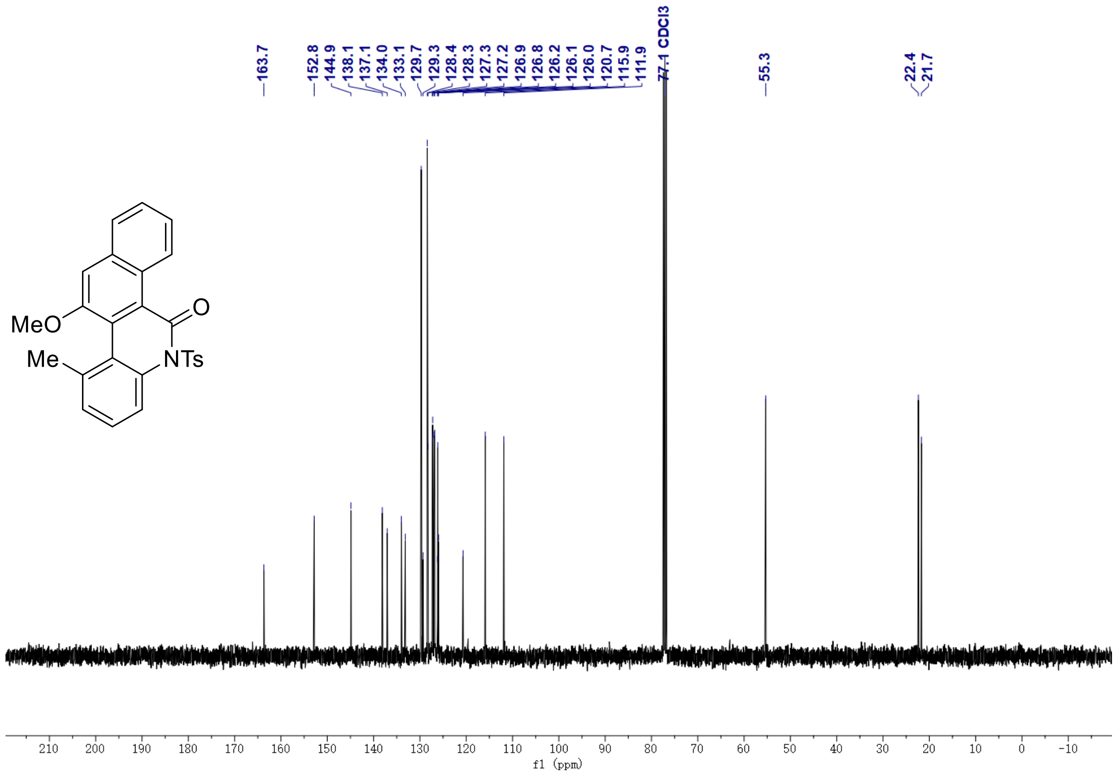

Supplementary Figure 16. ^{13}C NMR of the **1m** (101 MHz, $CDCl_3$)

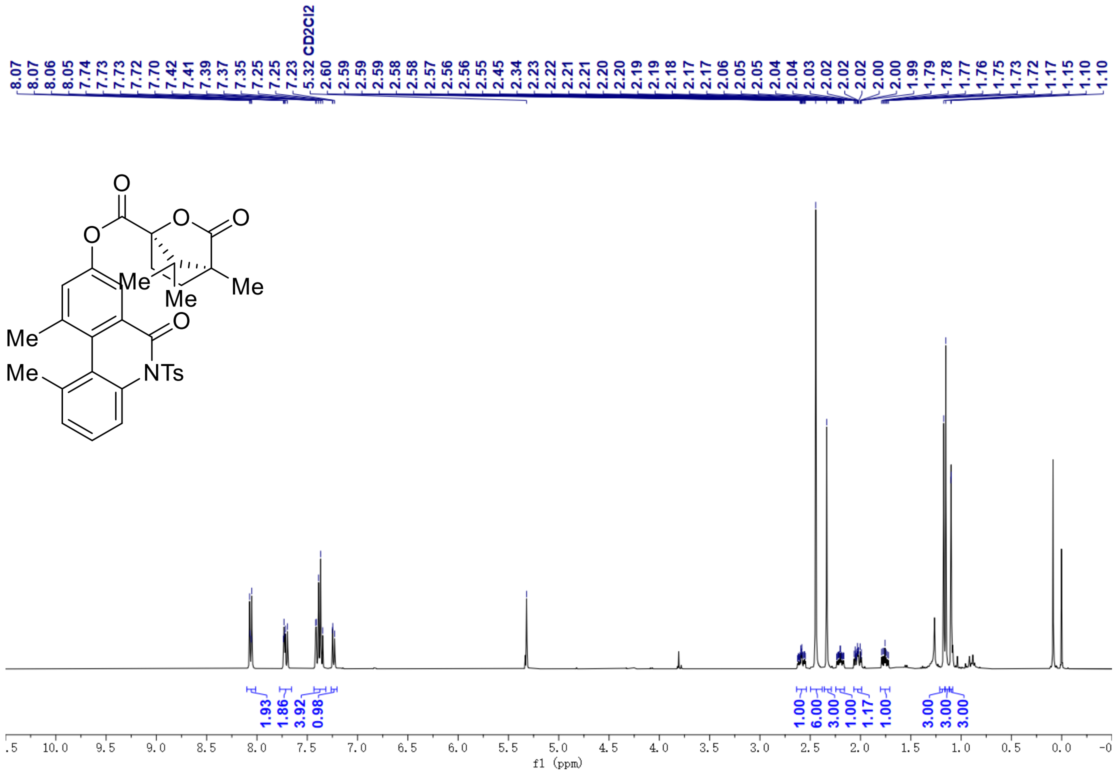

Supplementary Figure 17. ^{1}H NMR of the **1p** (400 MHz, $CDCl_3$)

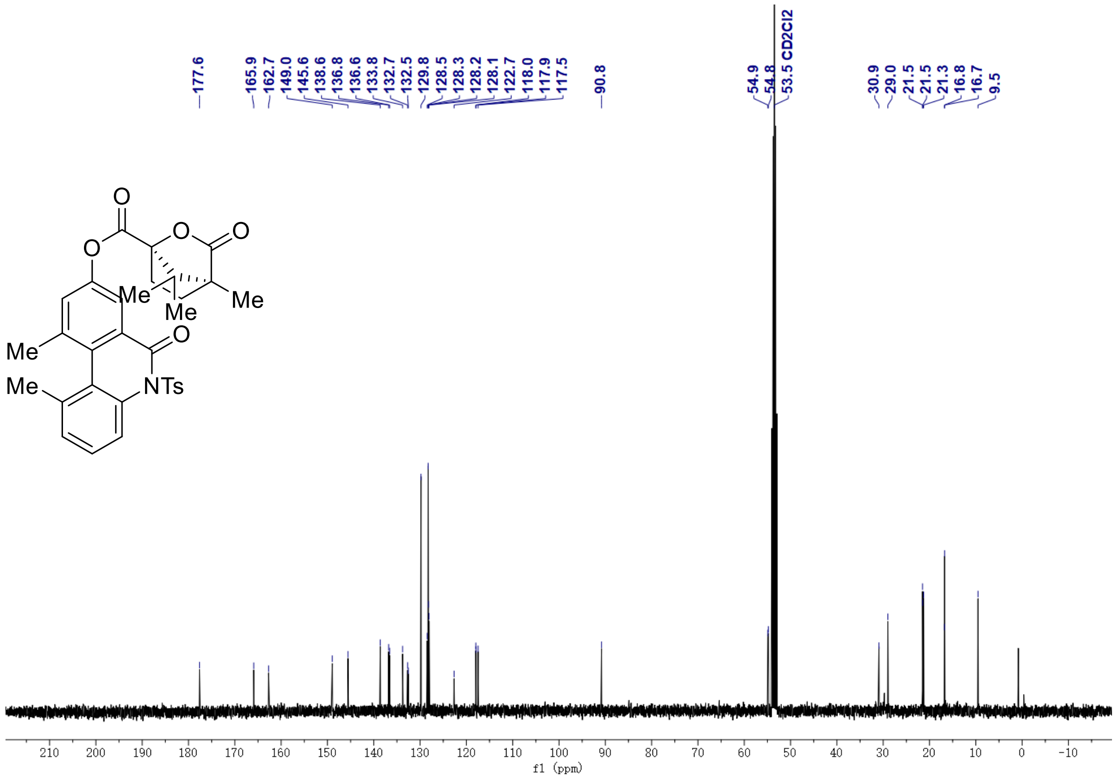

Supplementary Figure 18. ^{13}C NMR of the **1p** (101 MHz, $CDCl_3$)

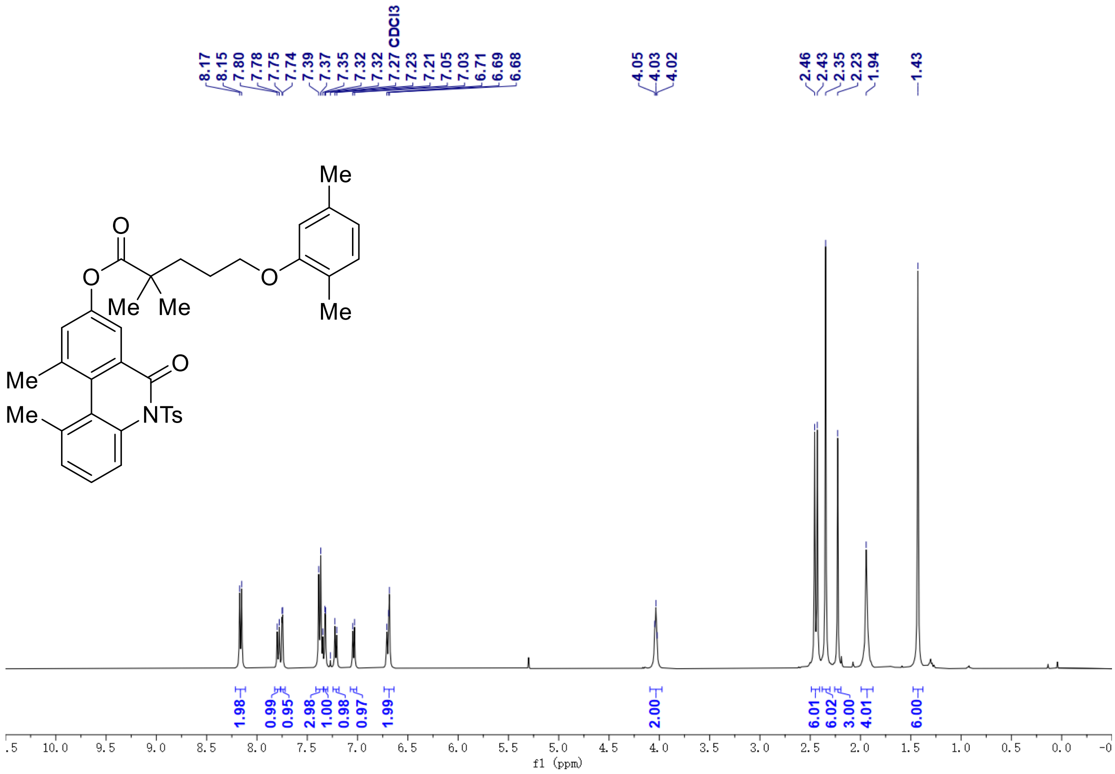

Supplementary Figure 19. **^{1}H NMR** of the **1q** (400 MHz, $CDCl_3$)

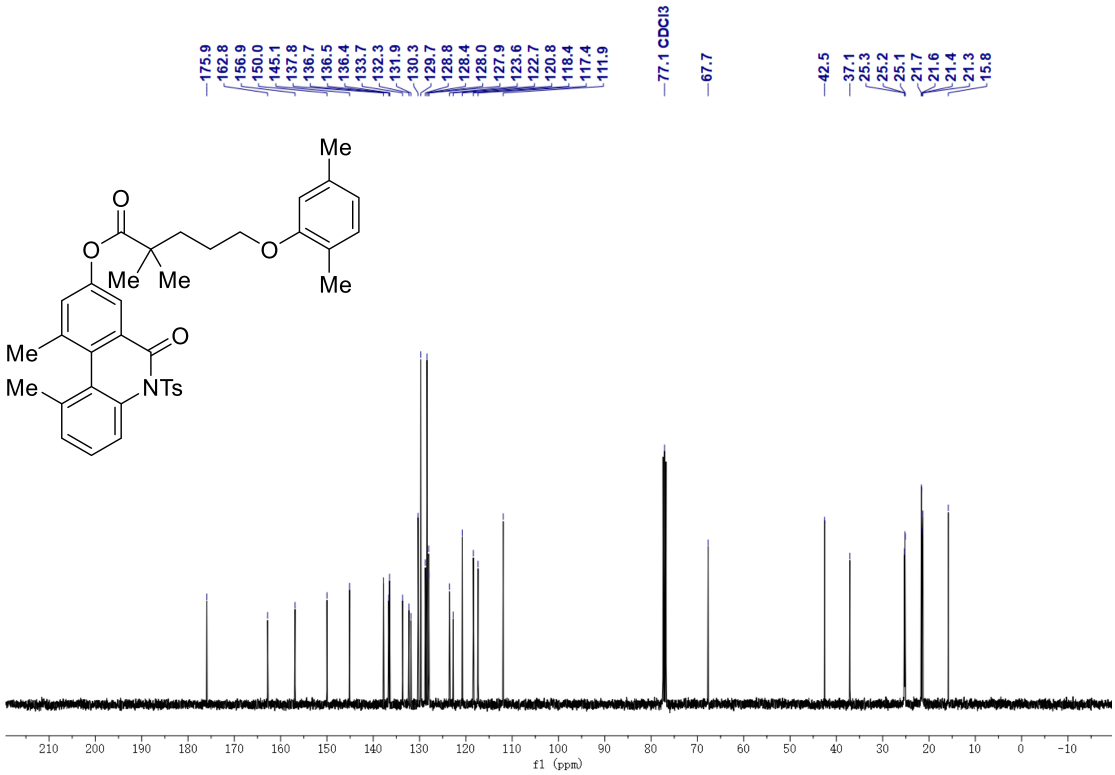

Supplementary Figure 20. **^{13}C NMR** of the **1q** (101 MHz, $CDCl_3$)

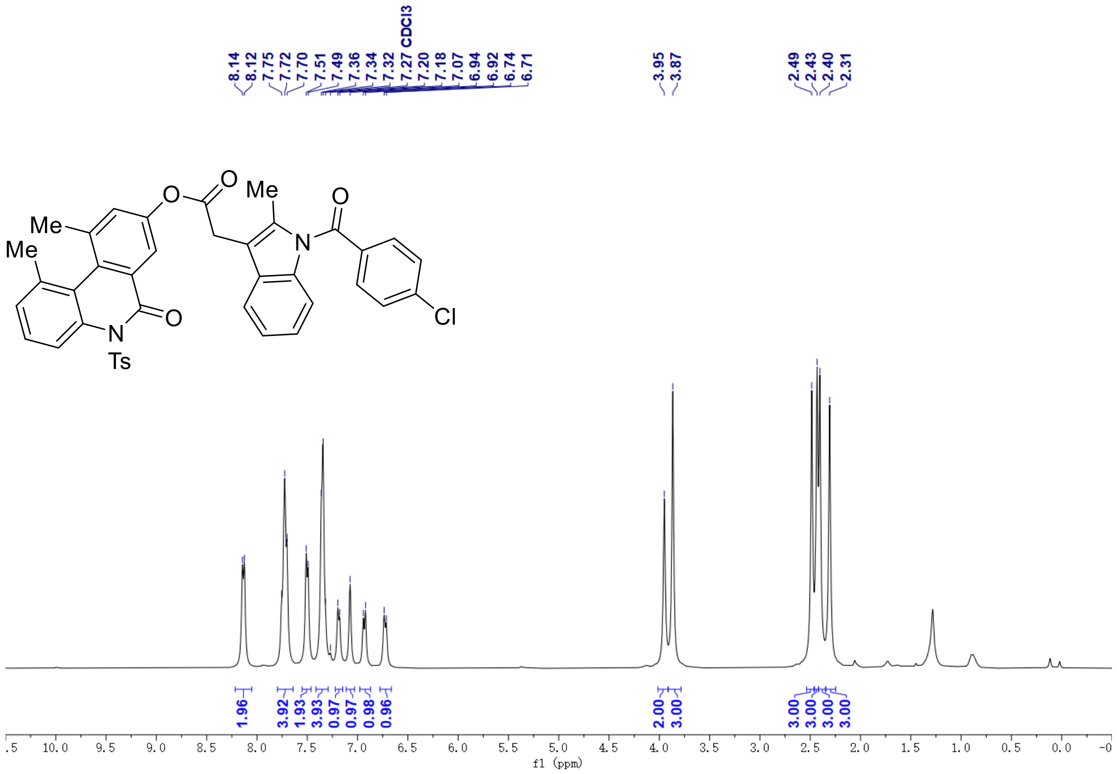

Supplementary Figure 21. ^{1}H NMR of the **1r** (400 MHz, $CDCl_3$)

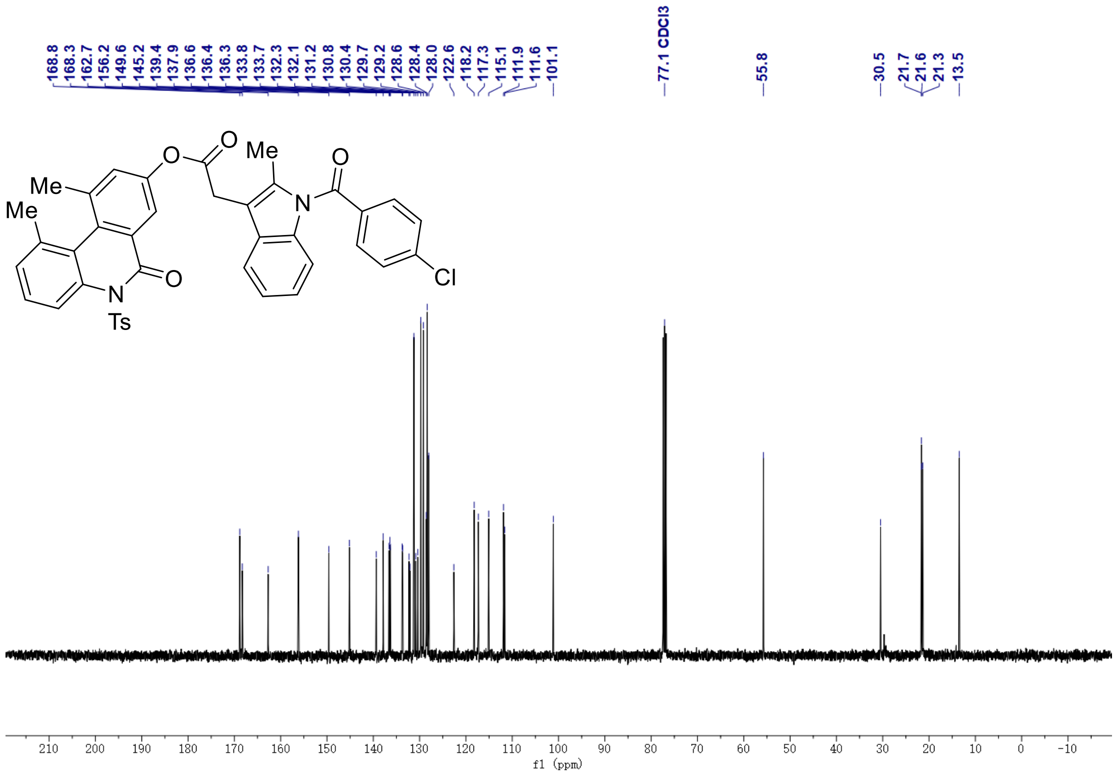

Supplementary Figure 22. ^{13}C NMR of the **1r** (101 MHz, $CDCl_3$)

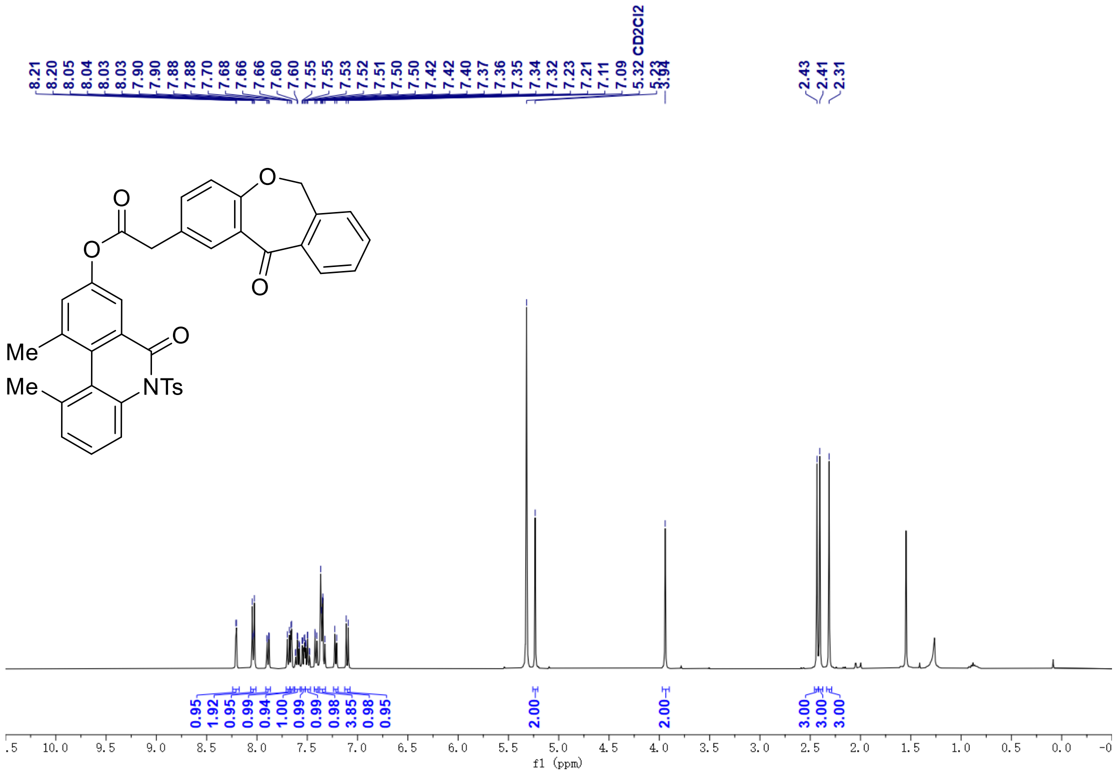

Supplementary Figure 23. ^{1}**H NMR** of the **1s** (400 MHz, CD_2Cl_2)

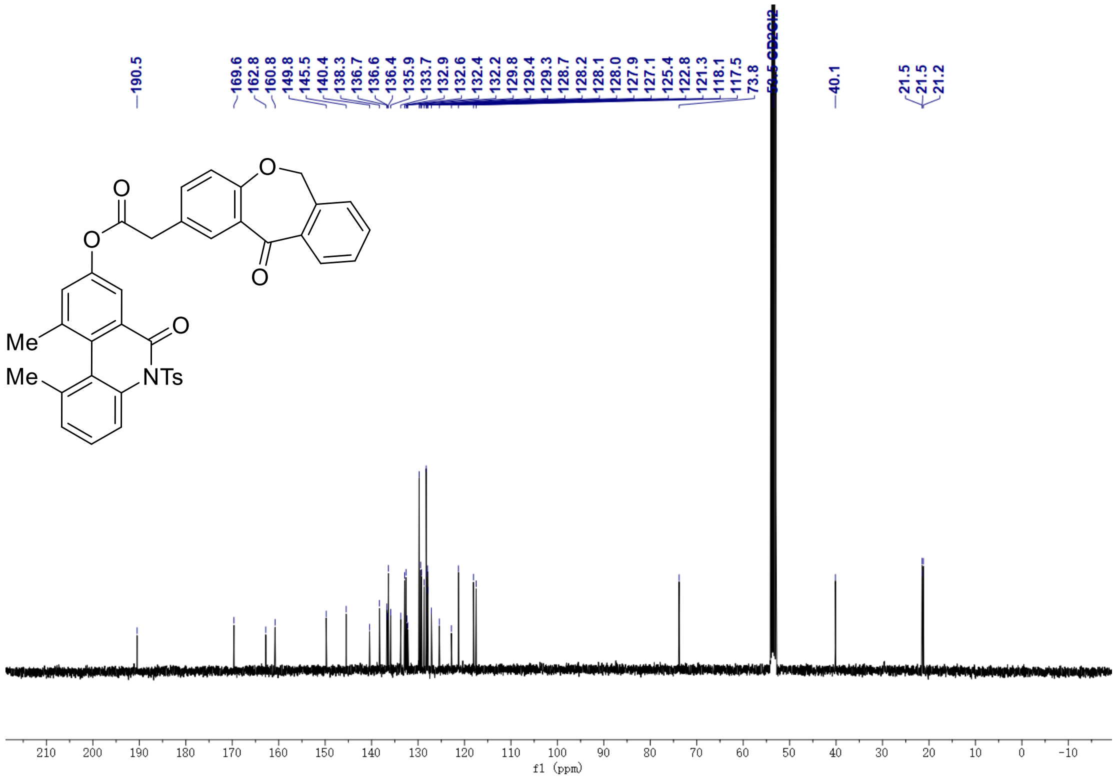

Supplementary Figure 24. ^{13}**C NMR** of the **1s** (101 MHz, CD_2Cl_2)

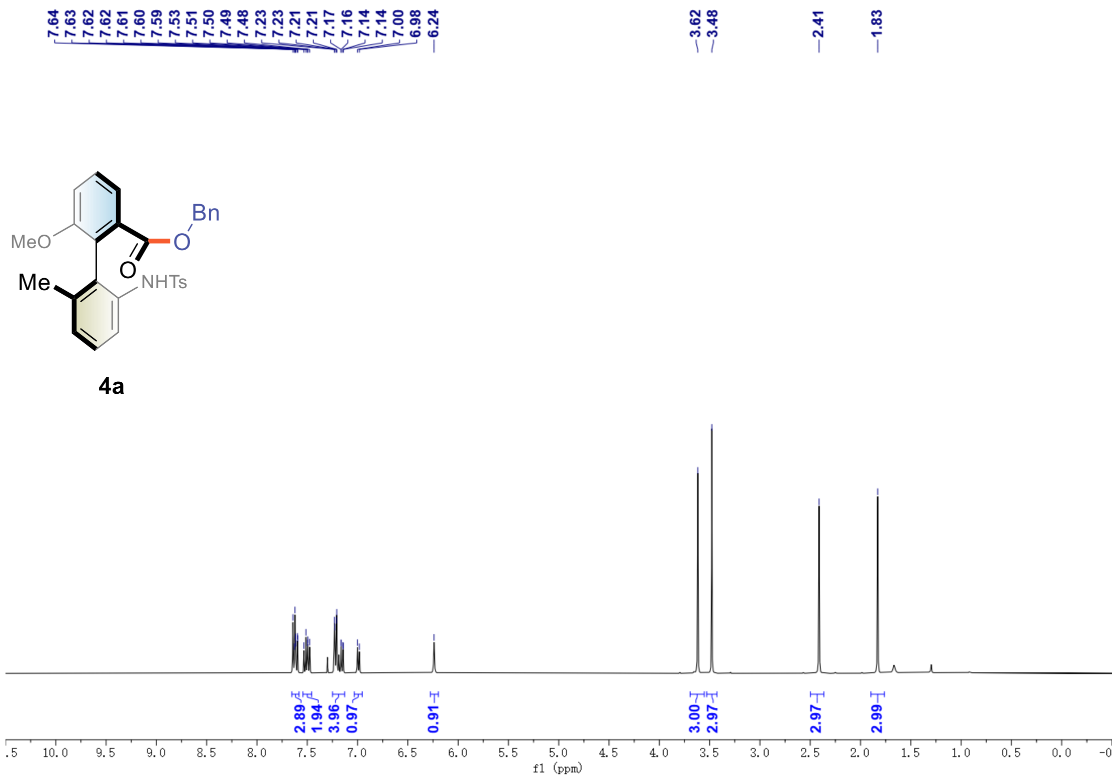

Supplementary Figure 25. ^{1}**H NMR** of the **4a** (400 MHz, $CDCl_3$)

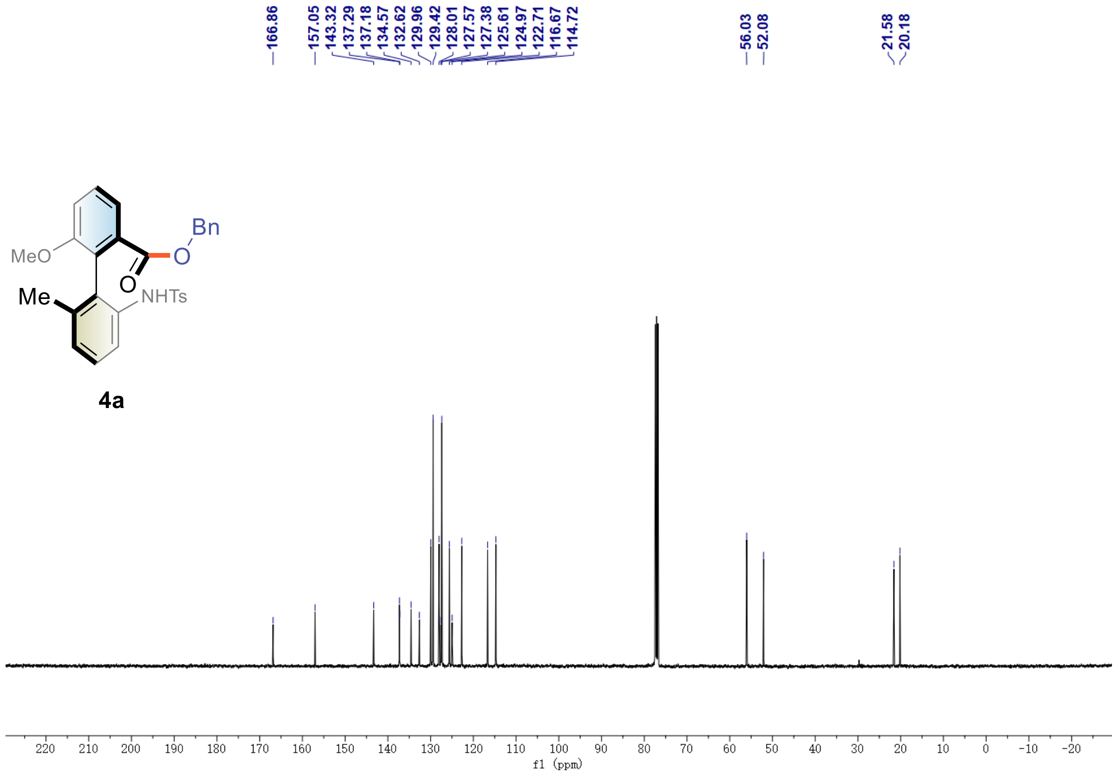

Supplementary Figure 26. ^{13}**C NMR** of the **4a** (101 MHz, $CDCl_3$)

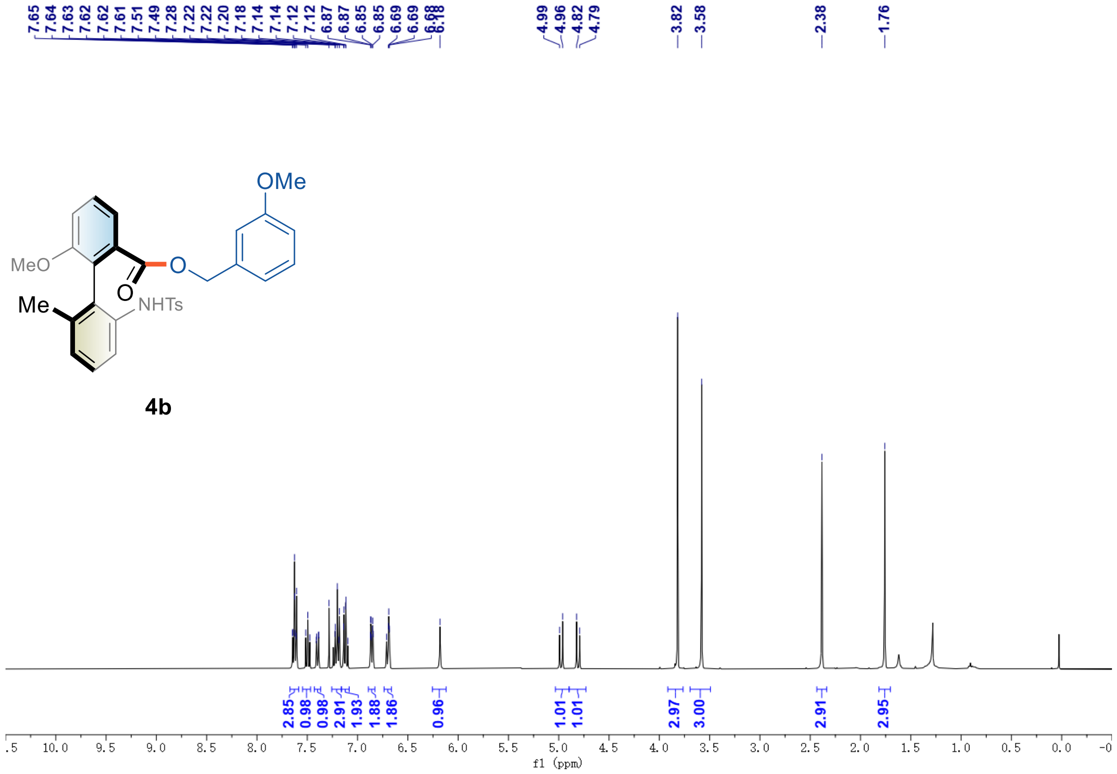

Supplementary Figure 27. ^{1}**H NMR** of the **4b** (400 MHz, CDCl$_3$)

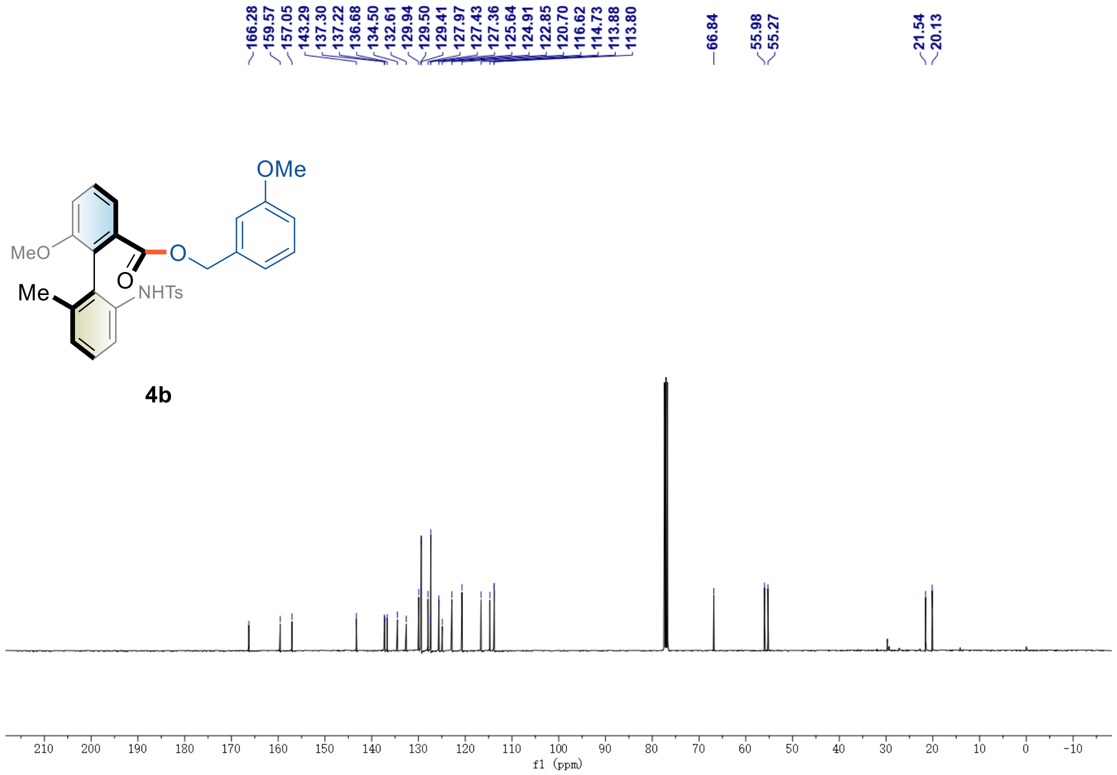

Supplementary Figure 28. ^{13}**C NMR** of the **4b** (101 MHz, CDCl$_3$)

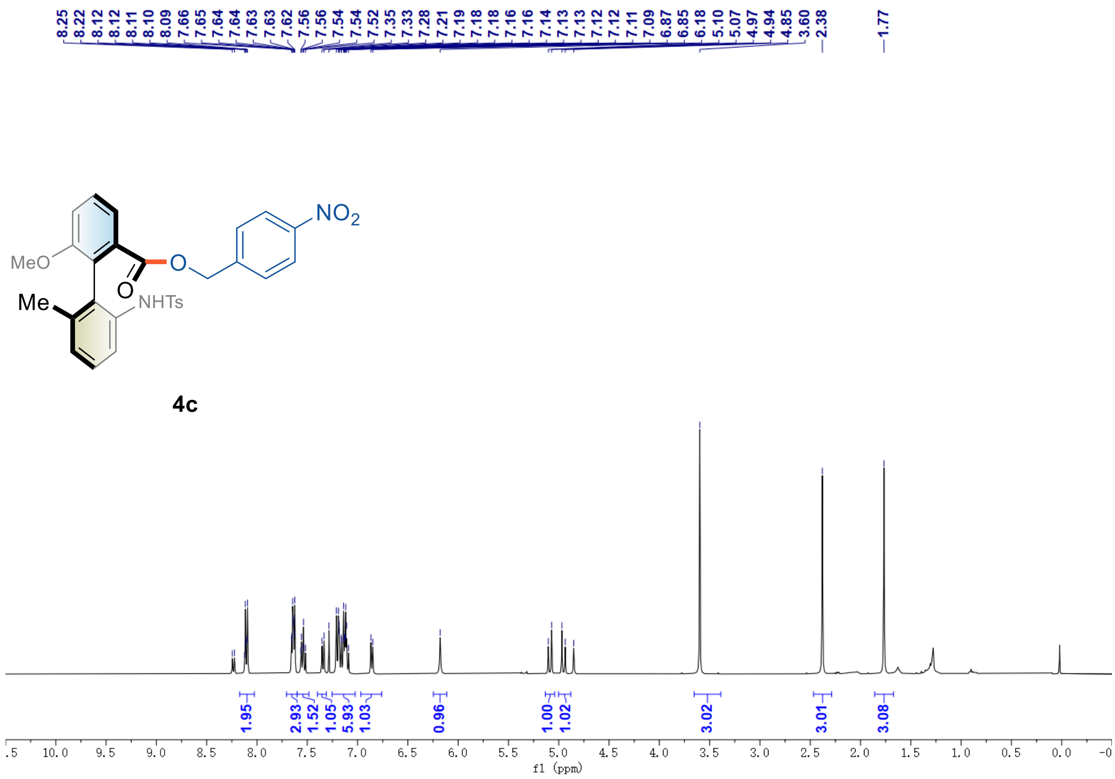

Supplementary Figure 29. ^{1}H NMR of the 4c (400 MHz, $CDCl_3$)

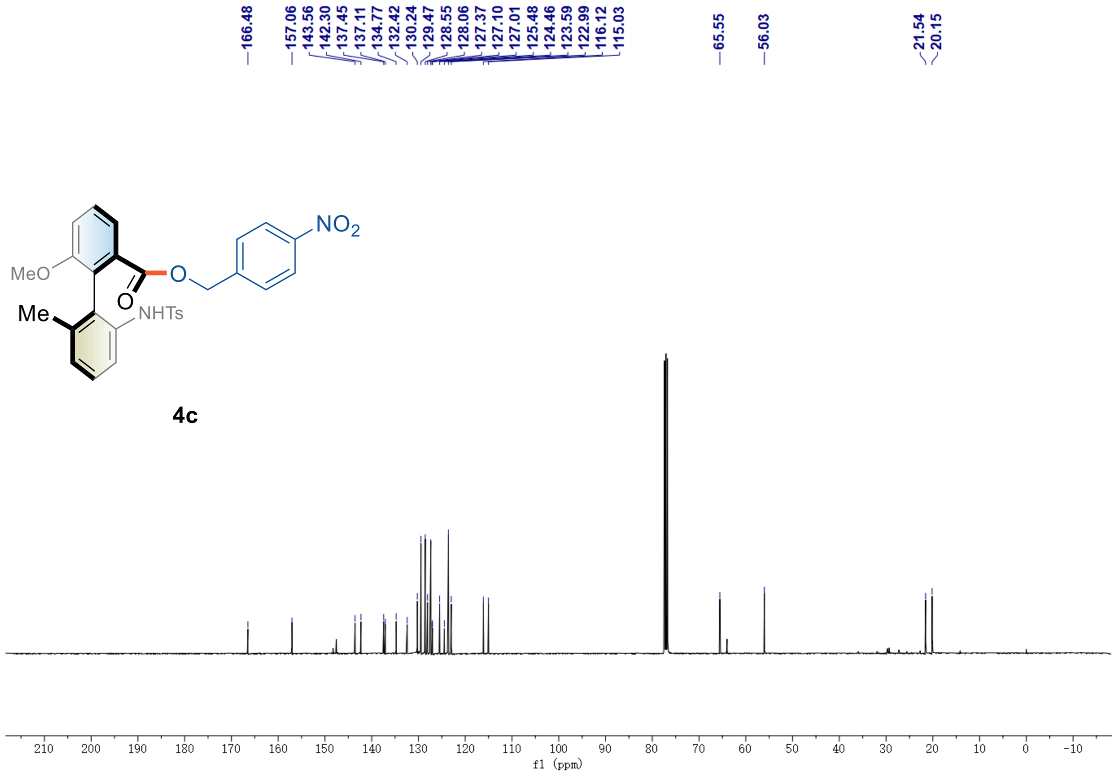

Supplementary Figure 30. ^{13}C NMR of the 4c (101 MHz, $CDCl_3$)

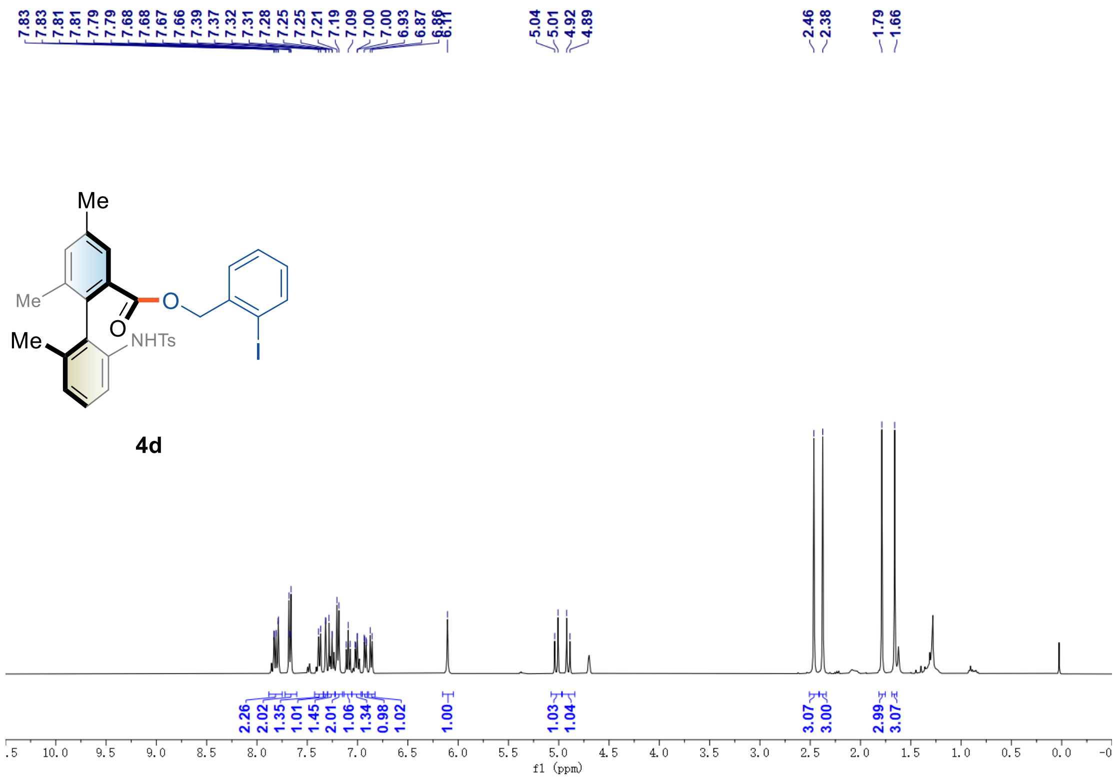

Supplementary Figure 31. ^{1}**H NMR** of the **4d** (400 MHz, $CDCl_3$)

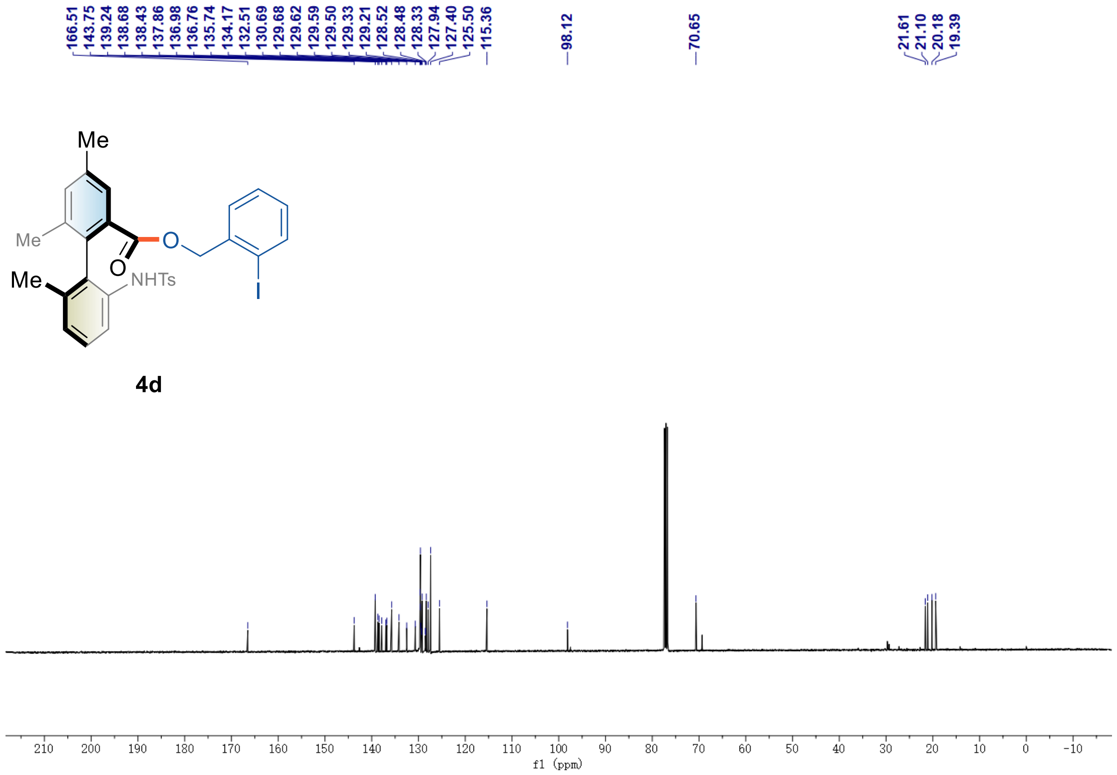

Supplementary Figure 32. ^{13}**C NMR** of the **4d** (101 MHz, $CDCl_3$)

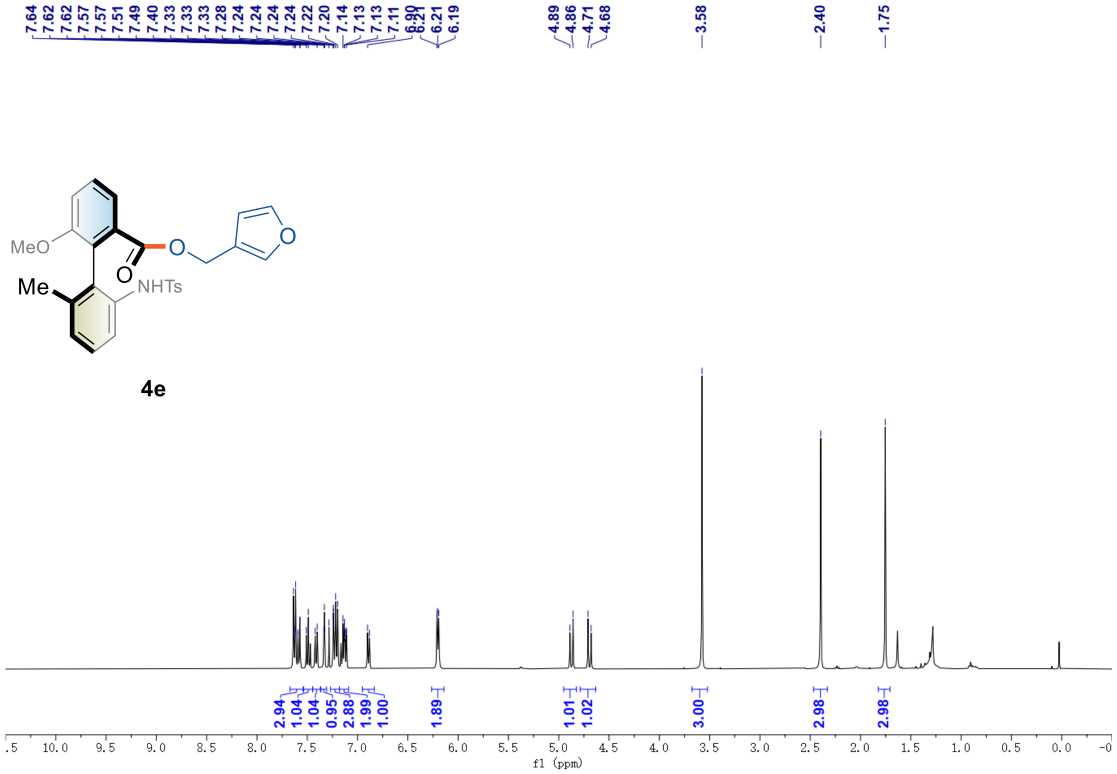

Supplementary Figure 33. ^{1}**H NMR** of the **4e** (400 MHz, $CDCl_3$)

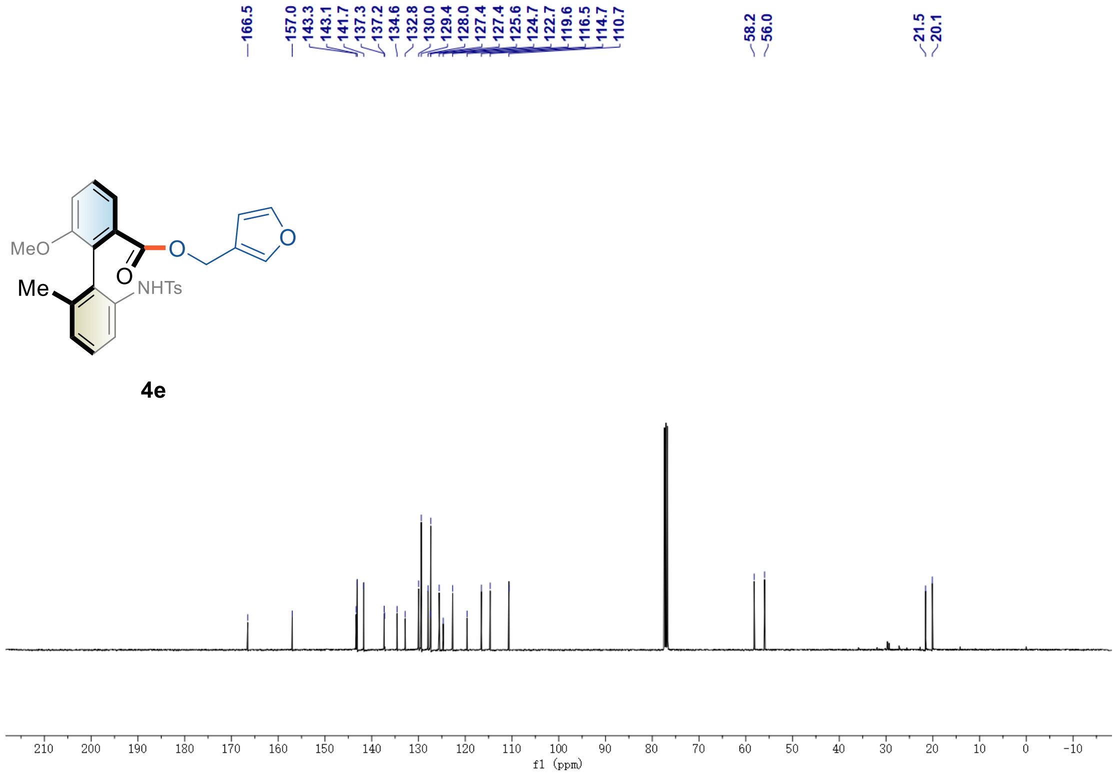

Supplementary Figure 34. ^{13}**C NMR** of the **4e** (101 MHz, $CDCl_3$)

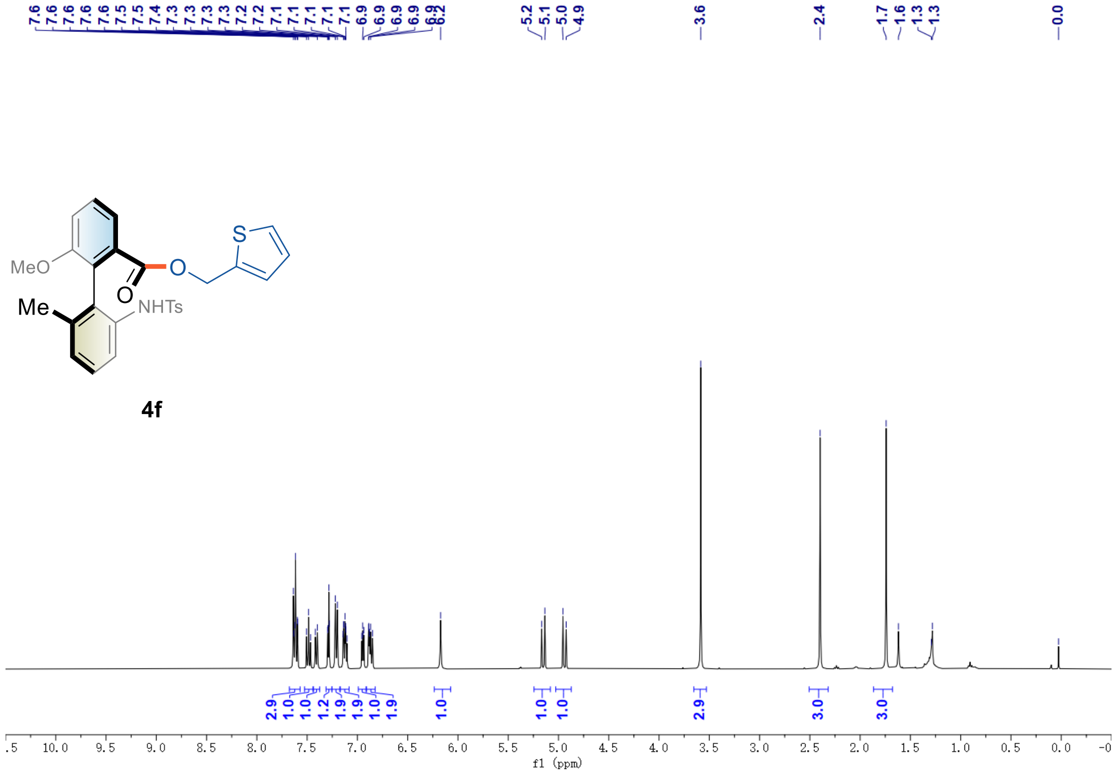

Supplementary Figure 35. ^{1}**H NMR** of the **4f** (400 MHz, $CDCl_3$)

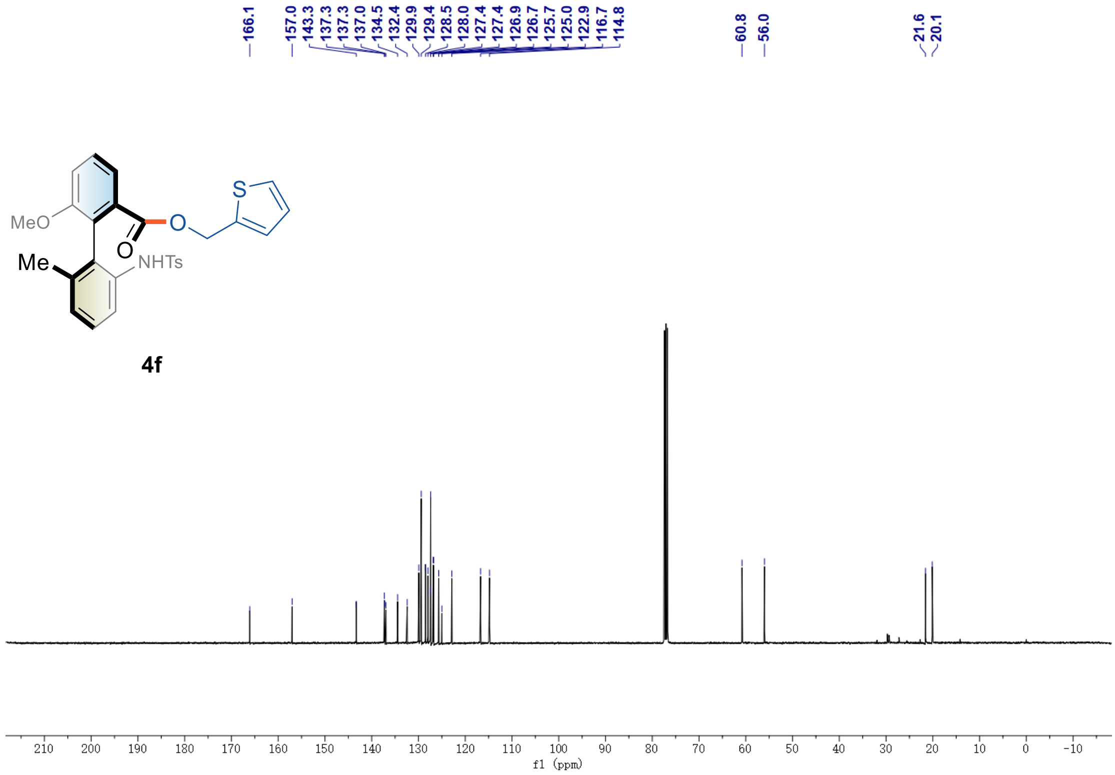

Supplementary Figure 36. ^{13}**C NMR** of the **4f** (101 MHz, $CDCl_3$)

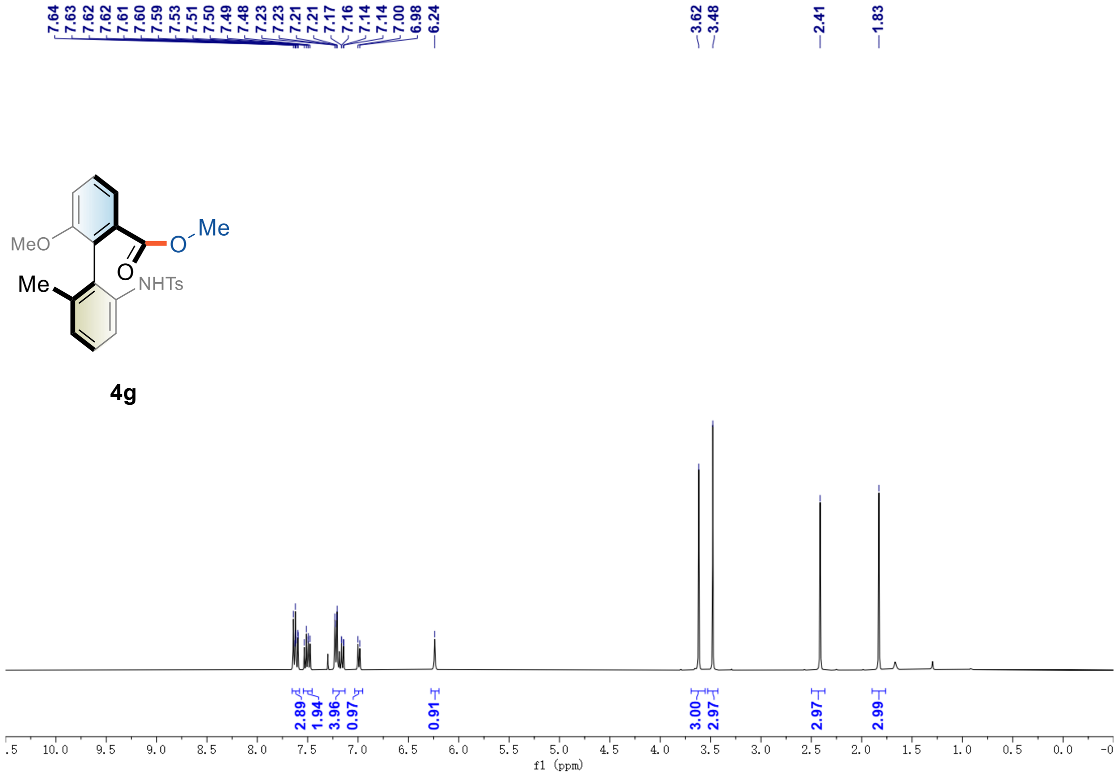

Supplementary Figure 37. **^{1}H NMR** of the **4g** (400 MHz, $CDCl_3$)

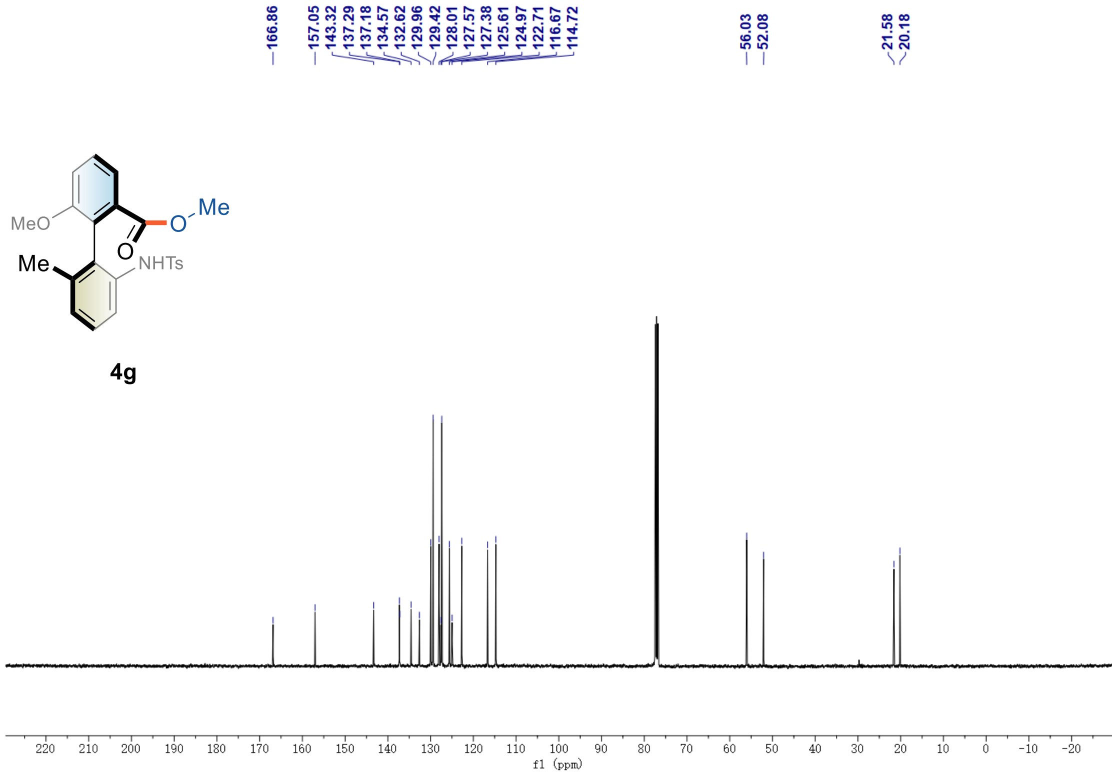

Supplementary Figure 38. **^{13}C NMR** of the **4g** (101 MHz, $CDCl_3$)

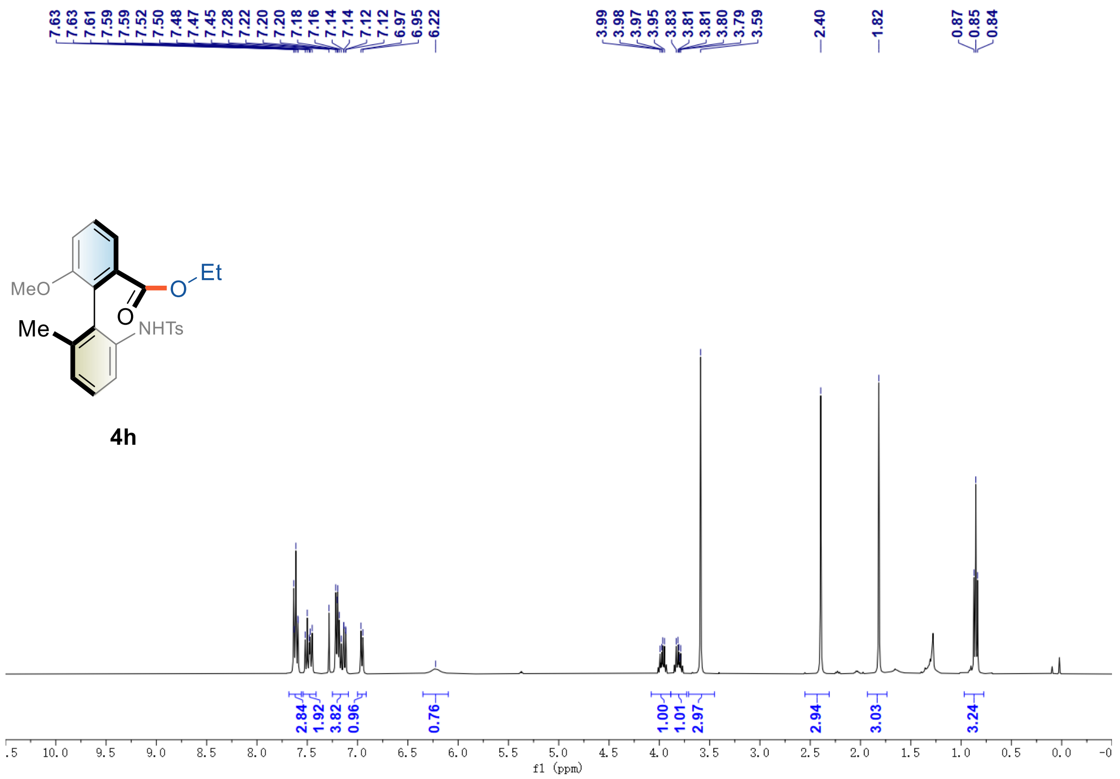

Supplementary Figure 39. **^{1}H NMR** of the **4h** (400 MHz, $CDCl_3$)

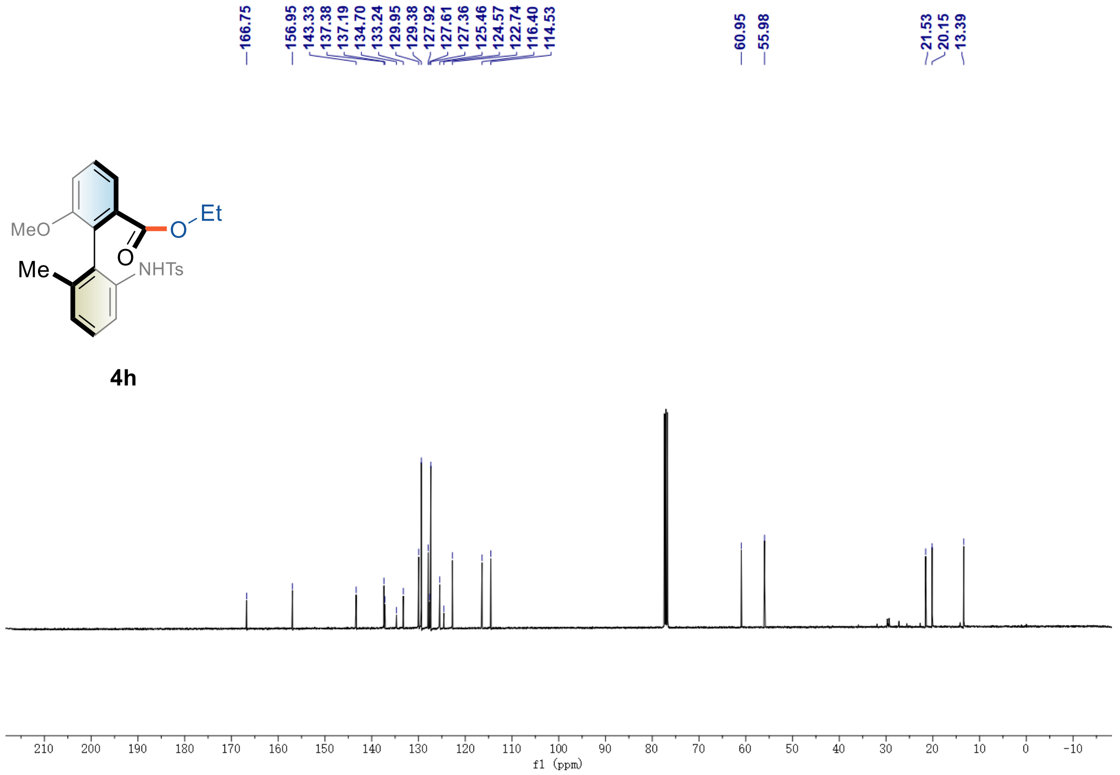

Supplementary Figure 40. **^{13}C NMR** of the **4h** (101 MHz, $CDCl_3$)

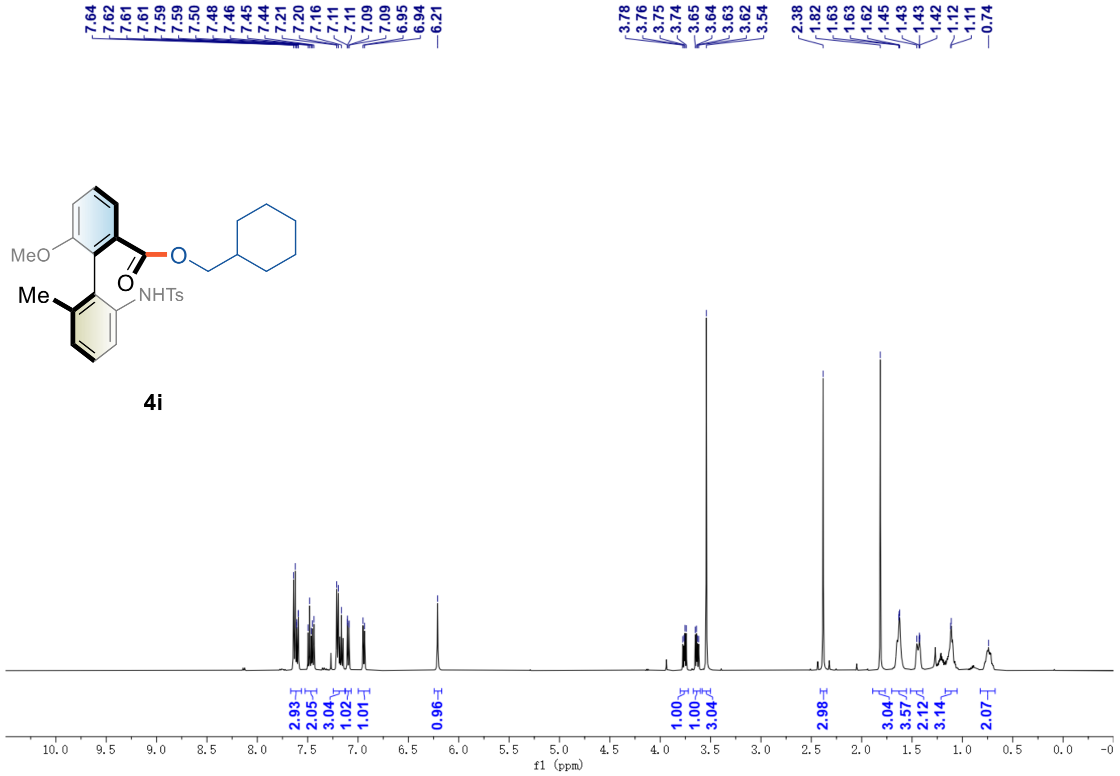

Supplementary Figure 41. ^{1}**H NMR** of the **4i** (500 MHz, $CDCl_3$)

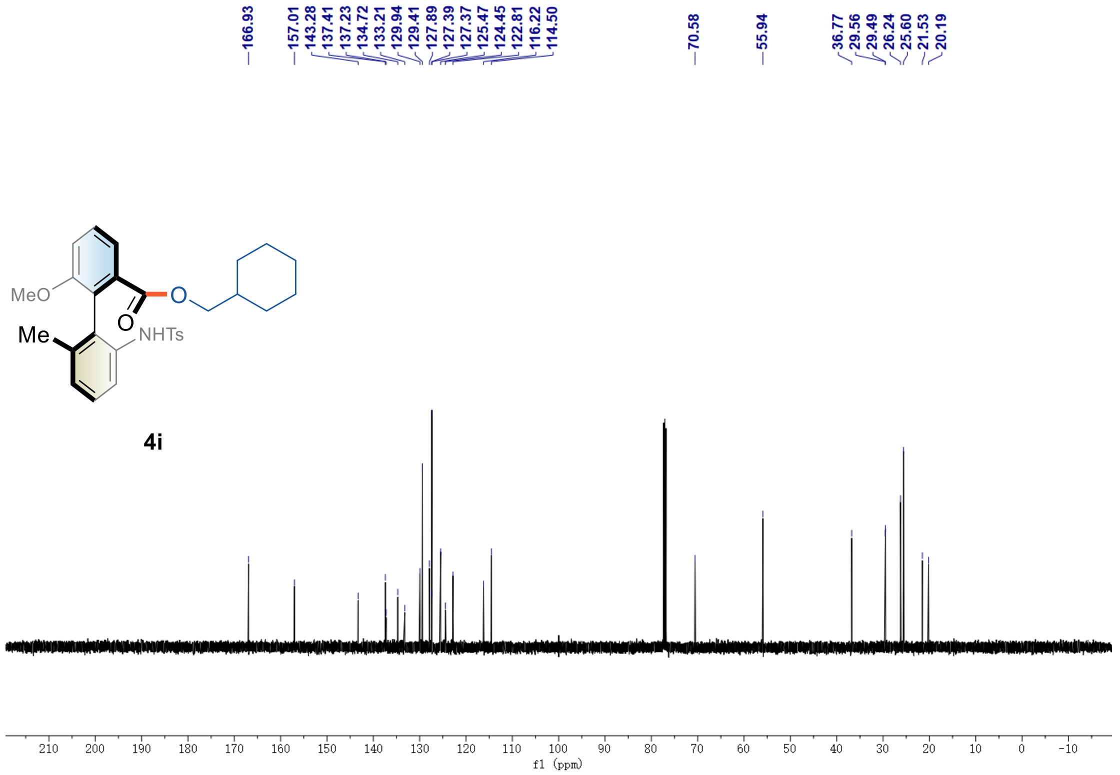

Supplementary Figure 42. ^{13}**C NMR** of the **4i** (126 MHz, $CDCl_3$)

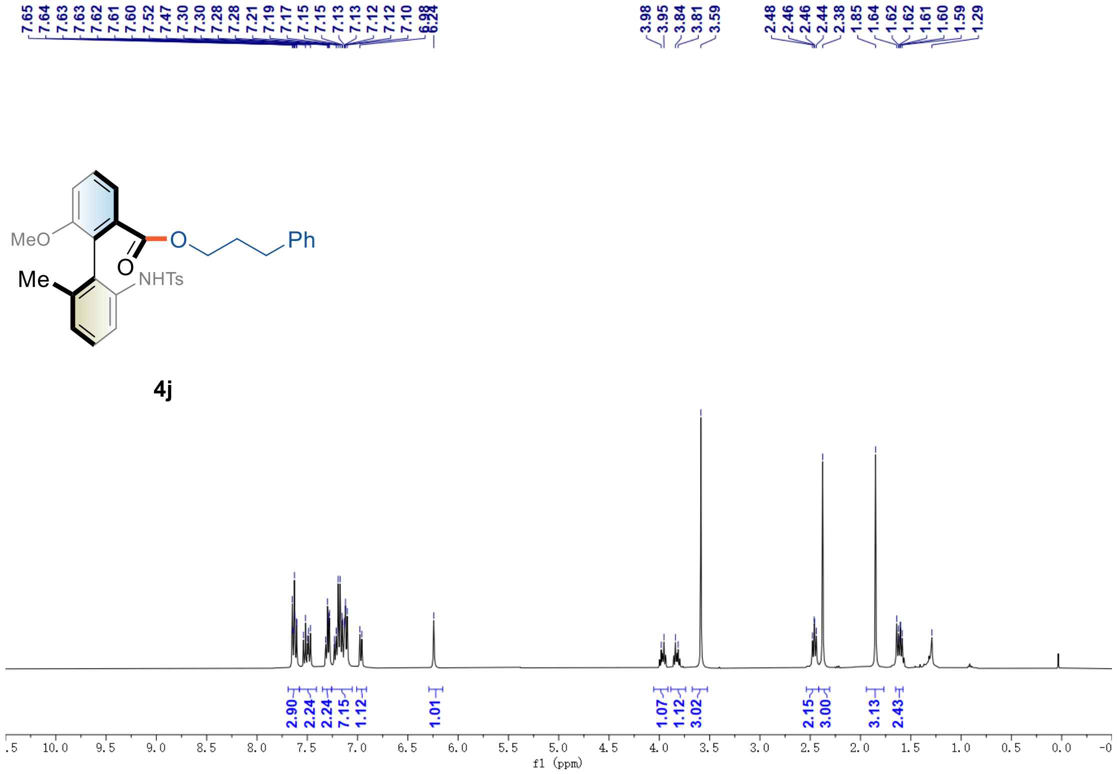

Supplementary Figure 43. **^{1}H NMR** of the **4j** (400 MHz, $CDCl_3$)

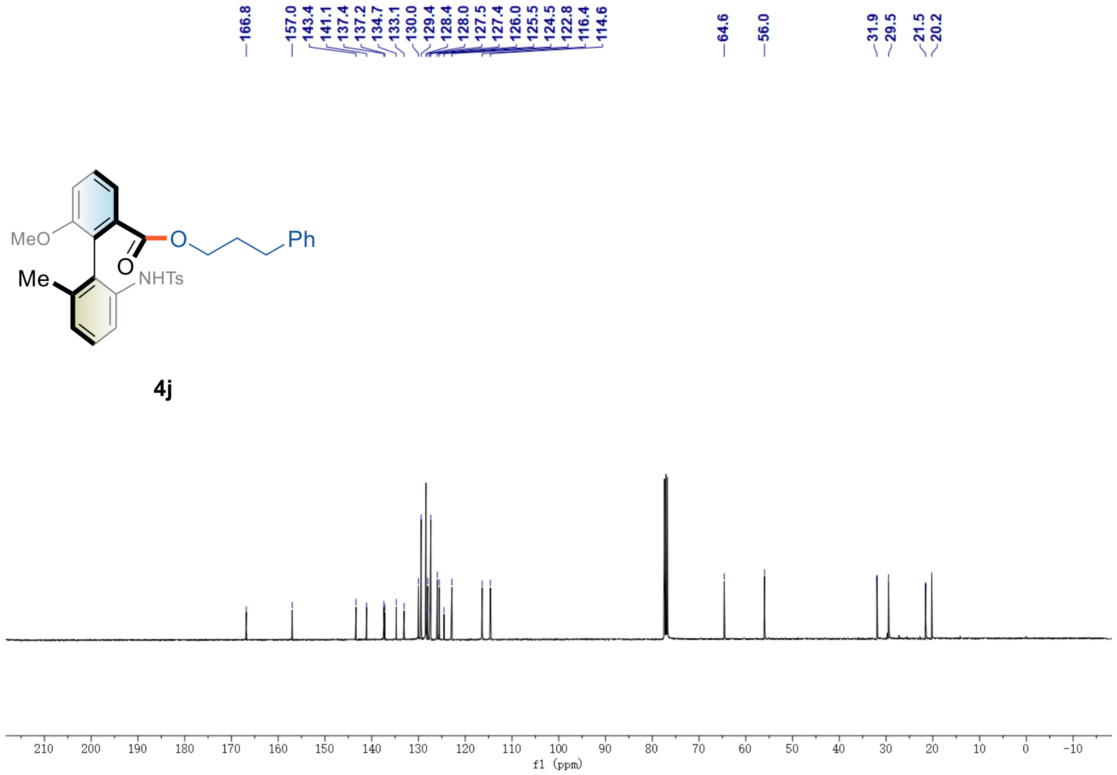

Supplementary Figure 44. **^{13}C NMR** of the **4j** (101 MHz, $CDCl_3$)

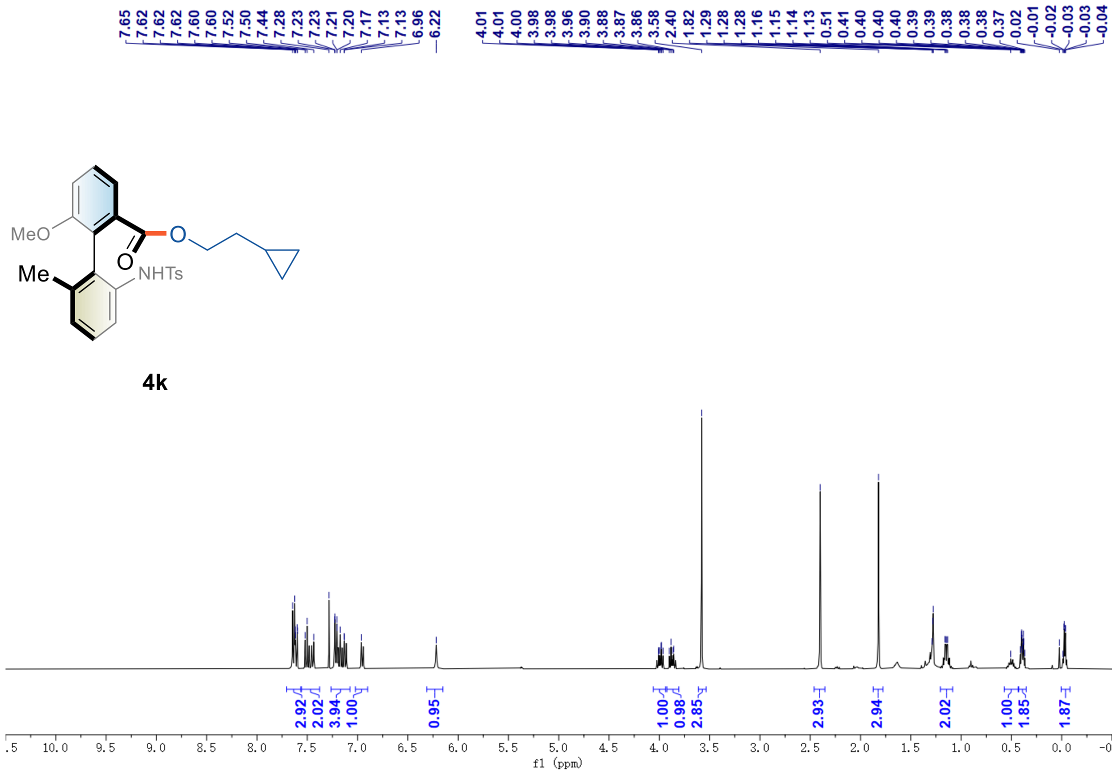

Supplementary Figure 45. **^{1}H NMR** of the **4k** (400 MHz, $CDCl_3$)

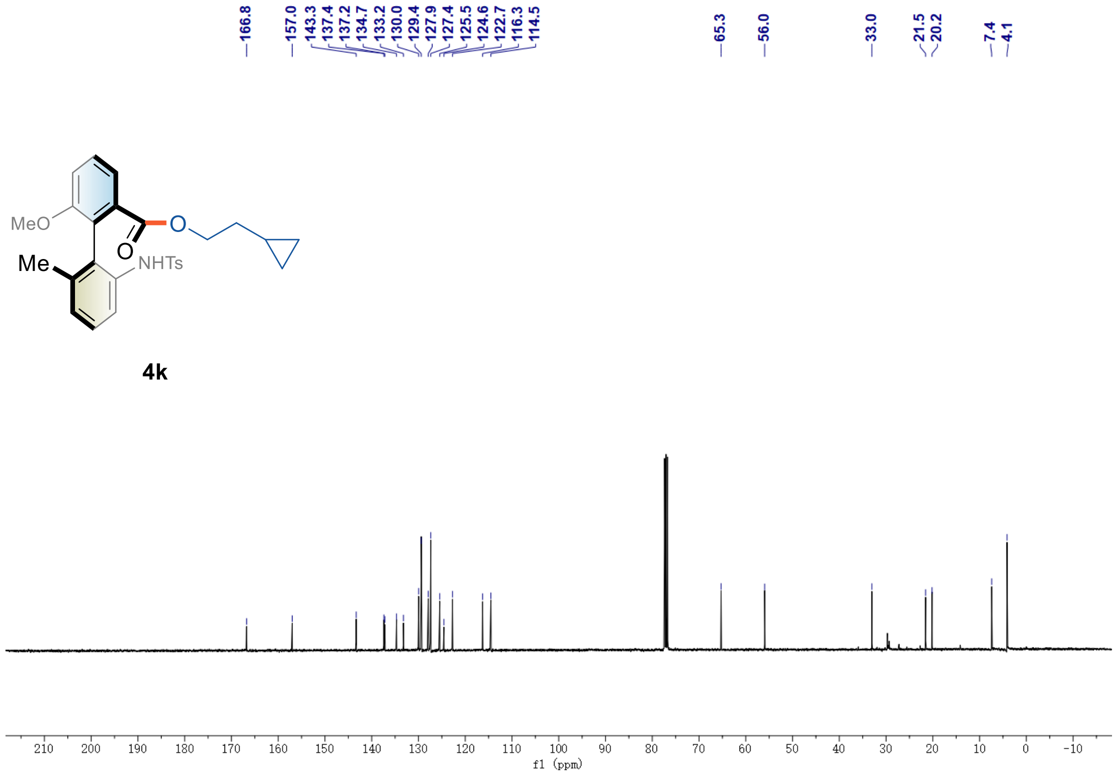

Supplementary Figure 46. **^{13}C NMR** of the **4k** (101 MHz, $CDCl_3$)

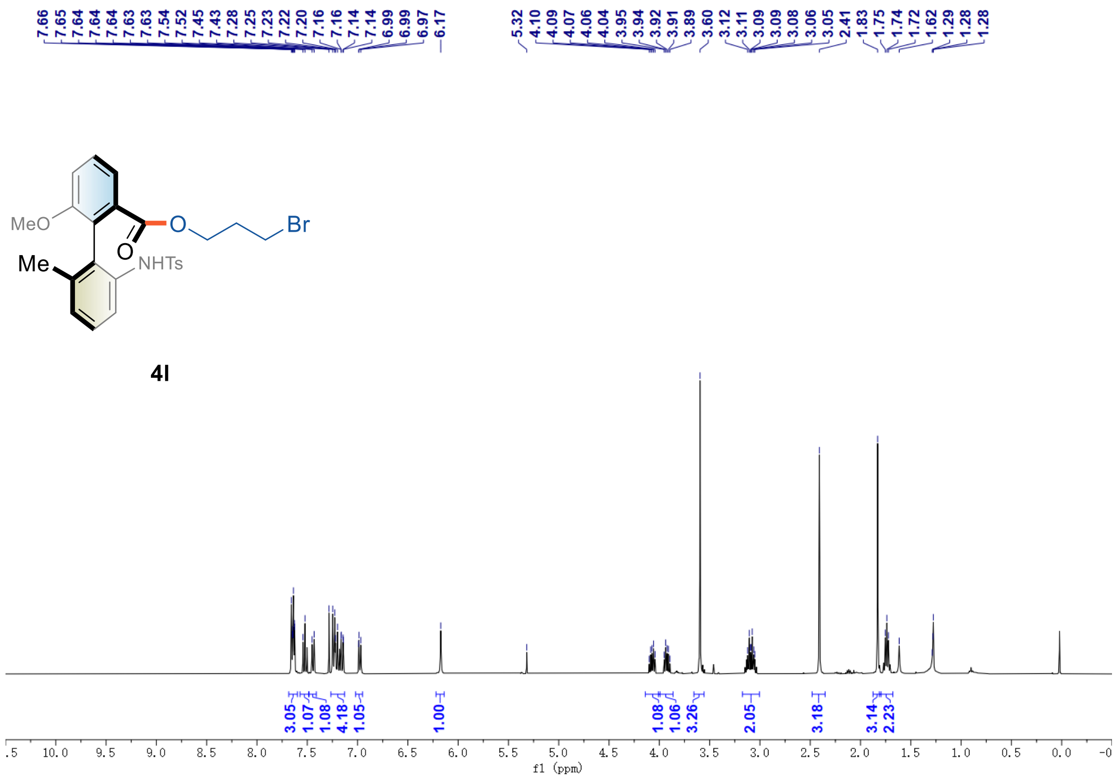

Supplementary Figure 47. ^{1}**H NMR** of the **4l** (400 MHz, $CDCl_3$)

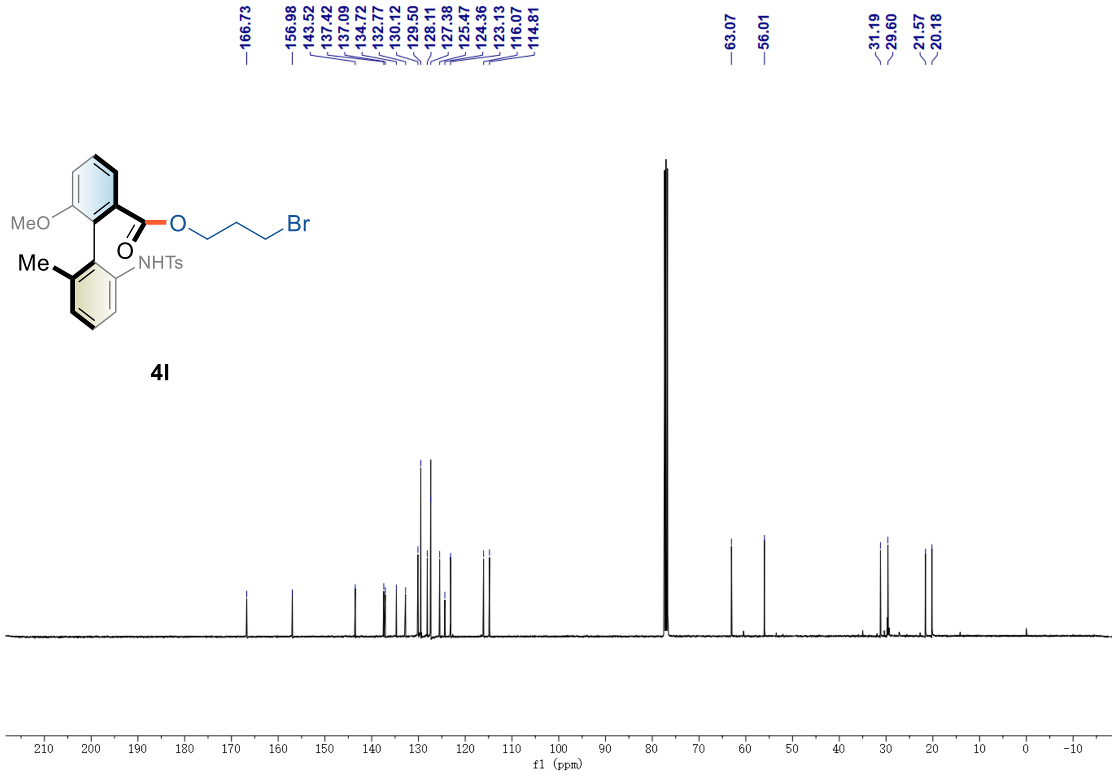

Supplementary Figure 48. ^{13}**C NMR** of the **4l** (101 MHz, $CDCl_3$)

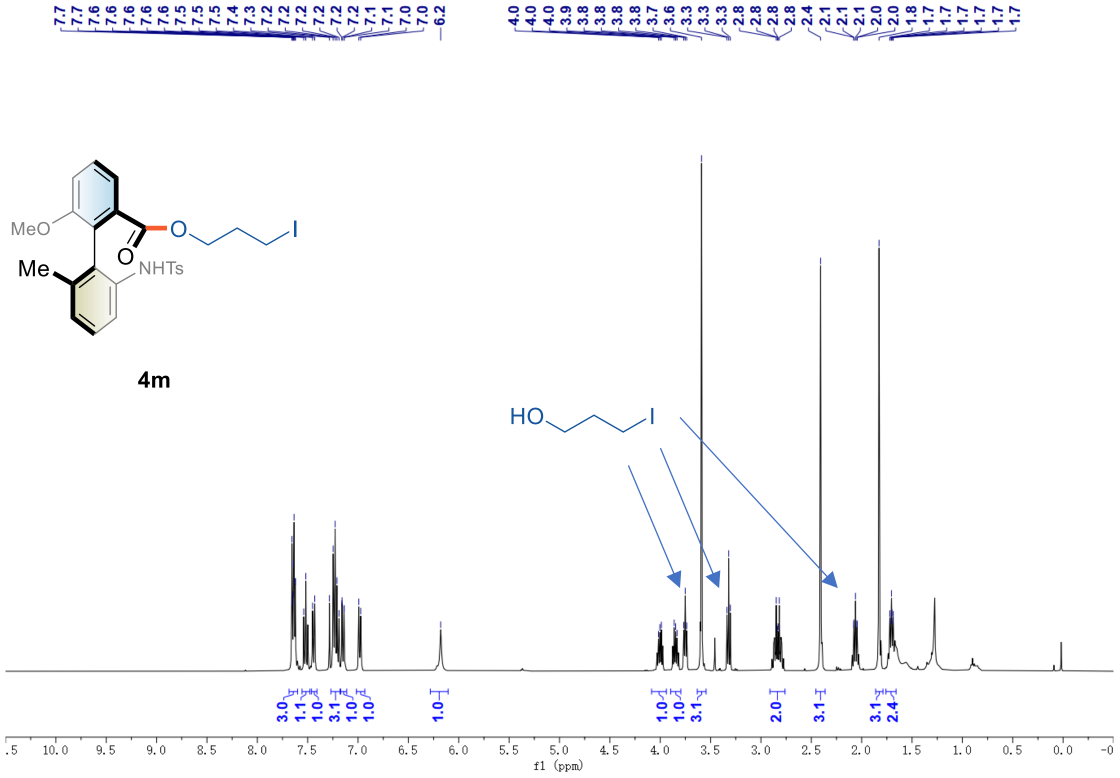

Supplementary Figure 49. **^{1}H NMR** of the **4m** (400 MHz, $CDCl_3$)

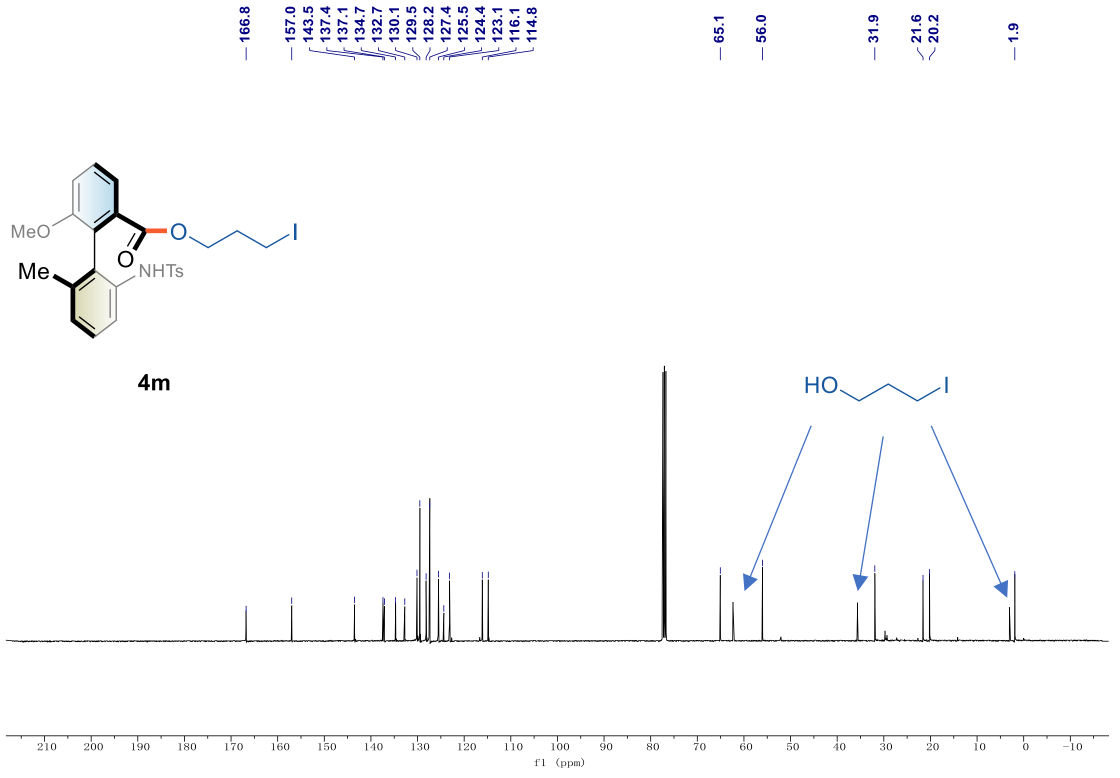

Supplementary Figure 50. **^{13}C NMR** of the **4m** (101 MHz, $CDCl_3$)

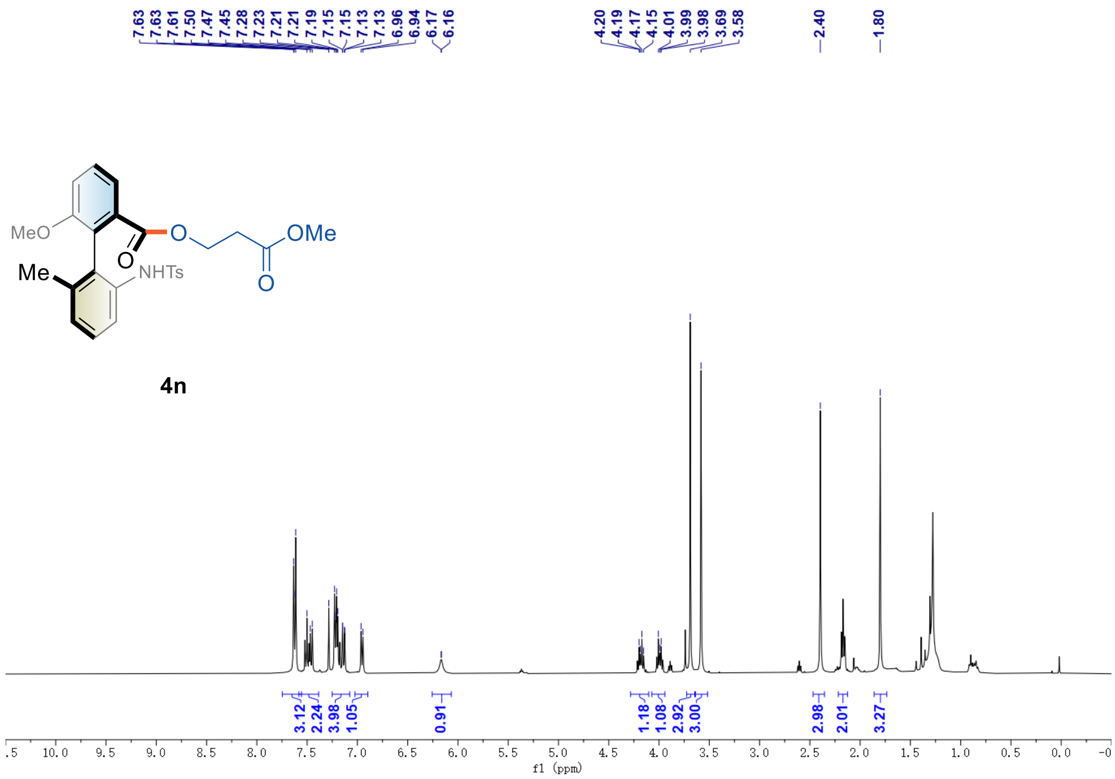

Supplementary Figure 51. ^{1}**H NMR** of the **4n** (400 MHz, $CDCl_3$)

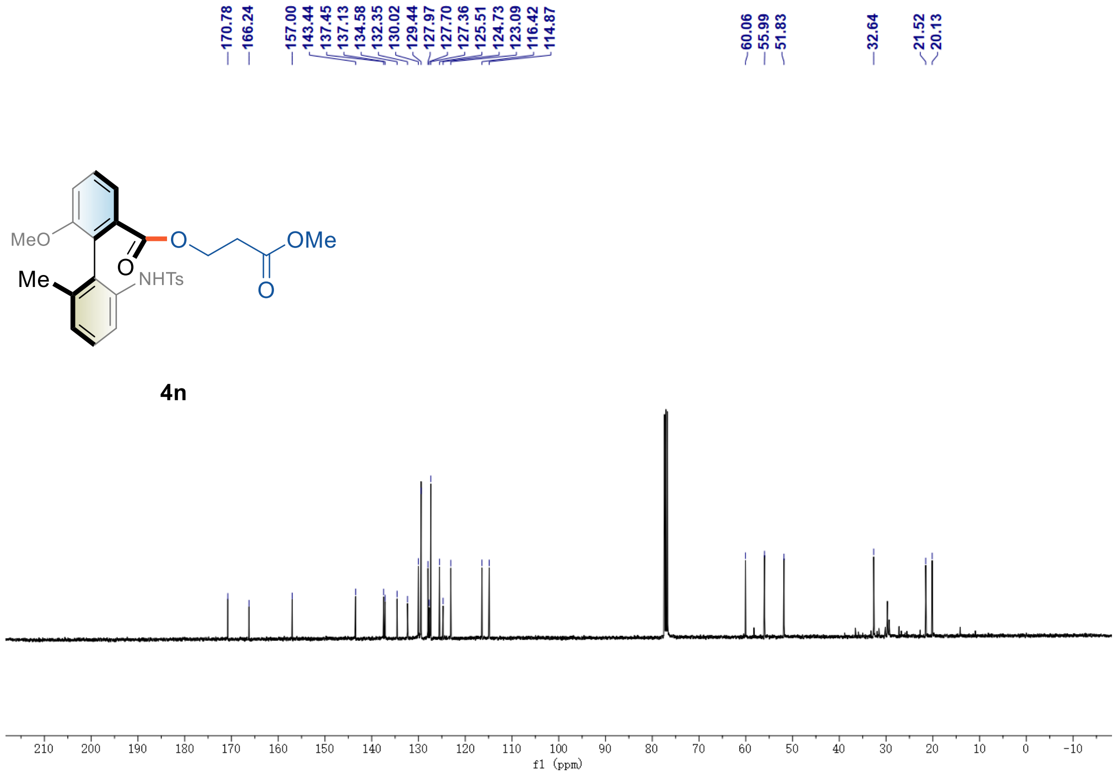

Supplementary Figure 52. ^{13}**C NMR** of the **4n** (101 MHz, $CDCl_3$)

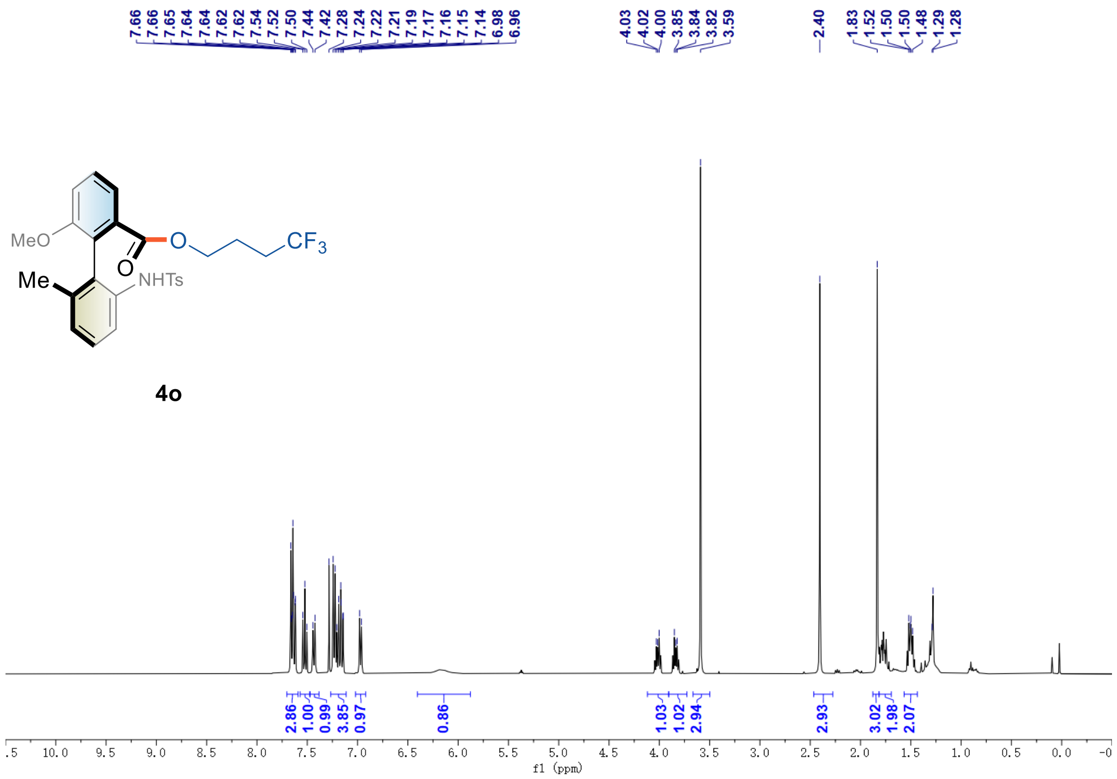

Supplementary Figure 53. ^{1}**H NMR** of the **4o** (400 MHz, $CDCl_3$)

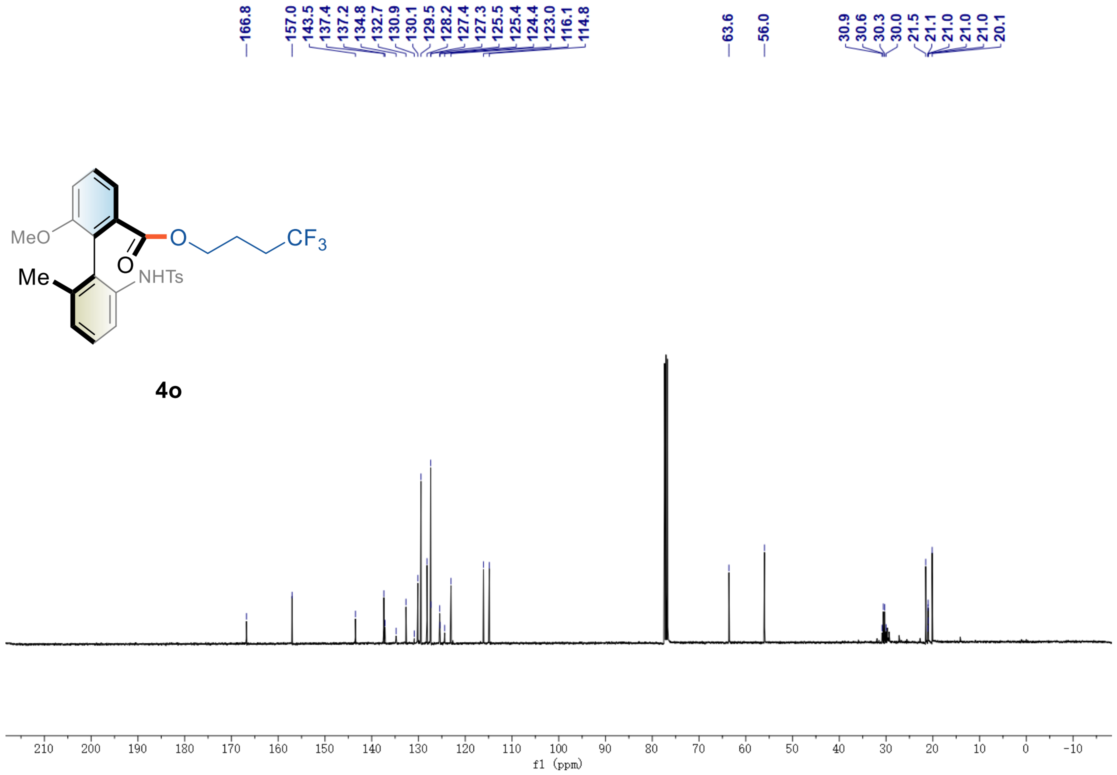

Supplementary Figure 54. ^{13}**C NMR** of the **4o** (101 MHz, $CDCl_3$)

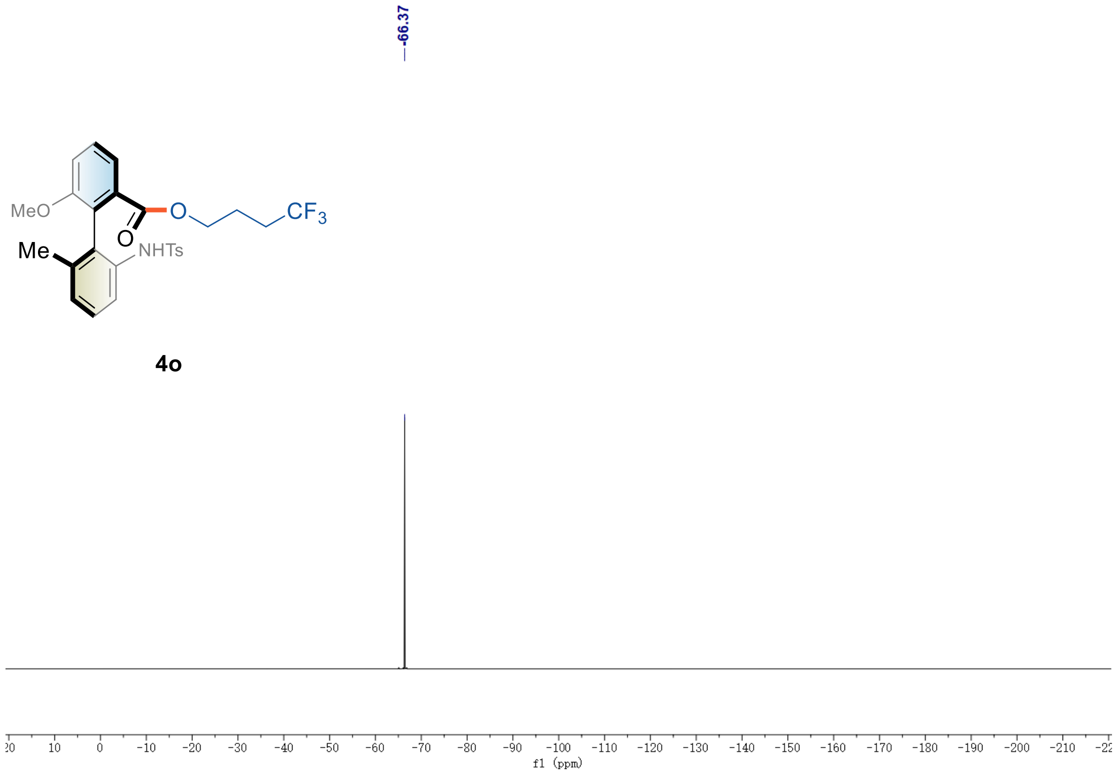

Supplementary Figure 55. ^{19}F NMR of the **4o** (376 MHz, $CDCl_3$)

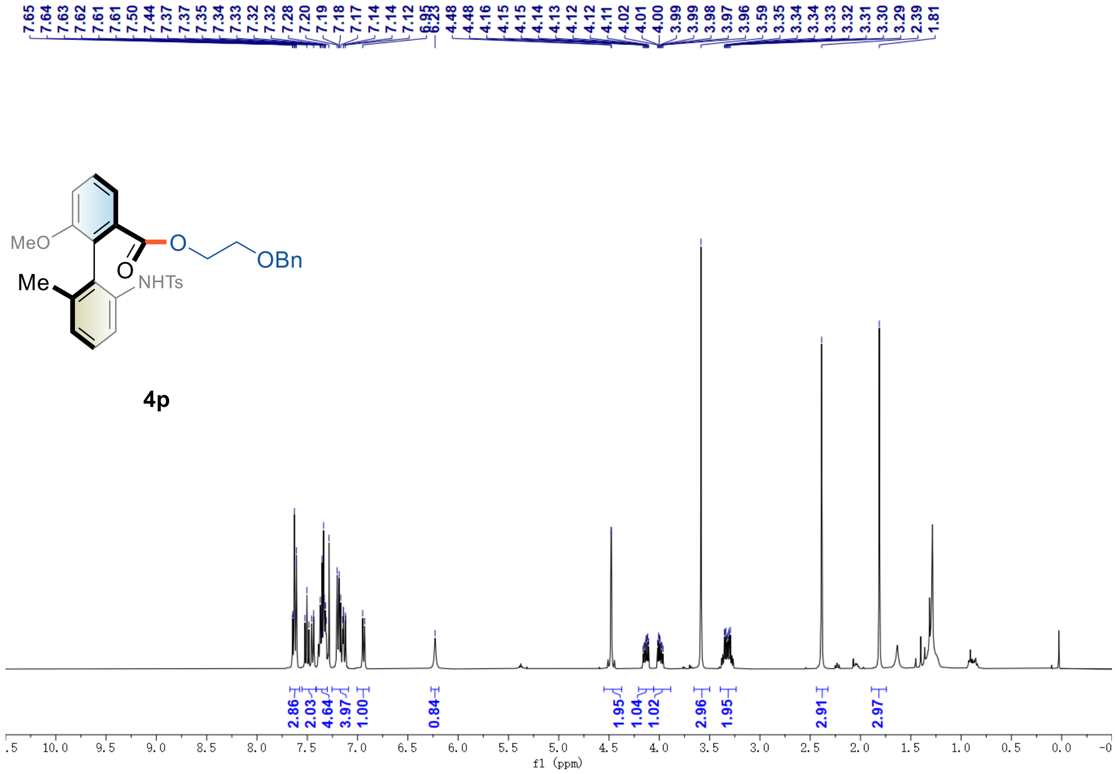

Supplementary Figure 56. ^{1}**H NMR** of the **4p** (400 MHz, $CDCl_3$)

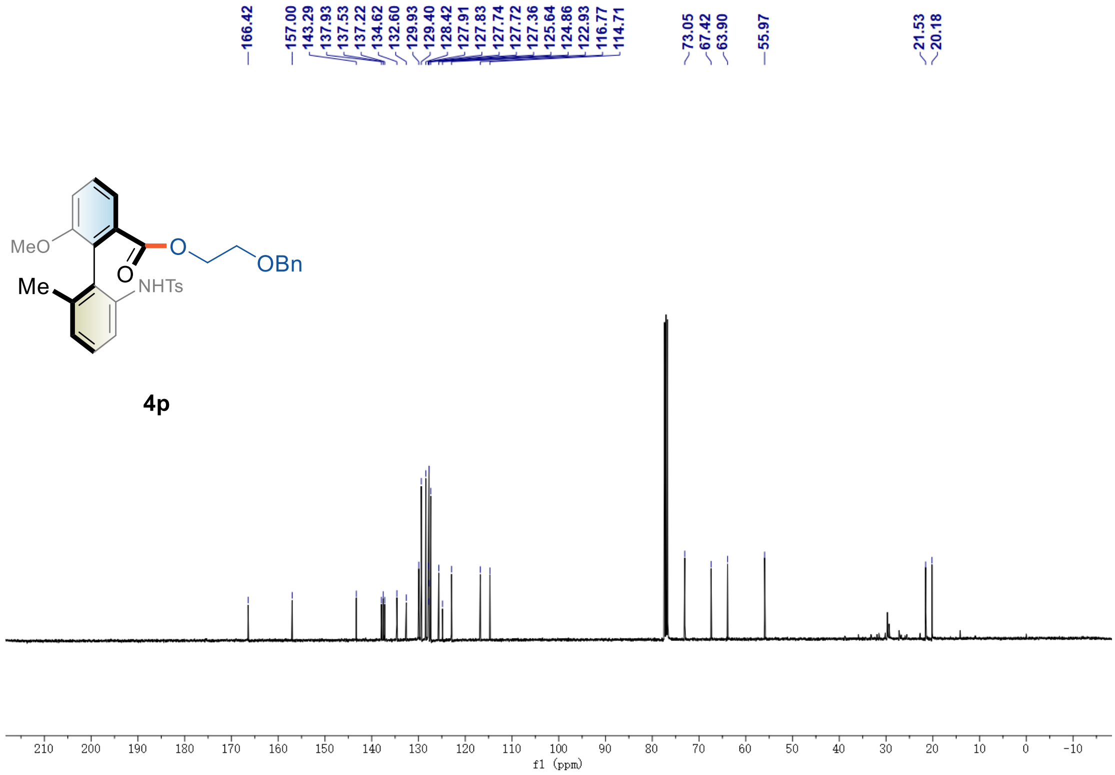

Supplementary Figure 57. ^{13}**C NMR** of the **4p** (101 MHz, $CDCl_3$)

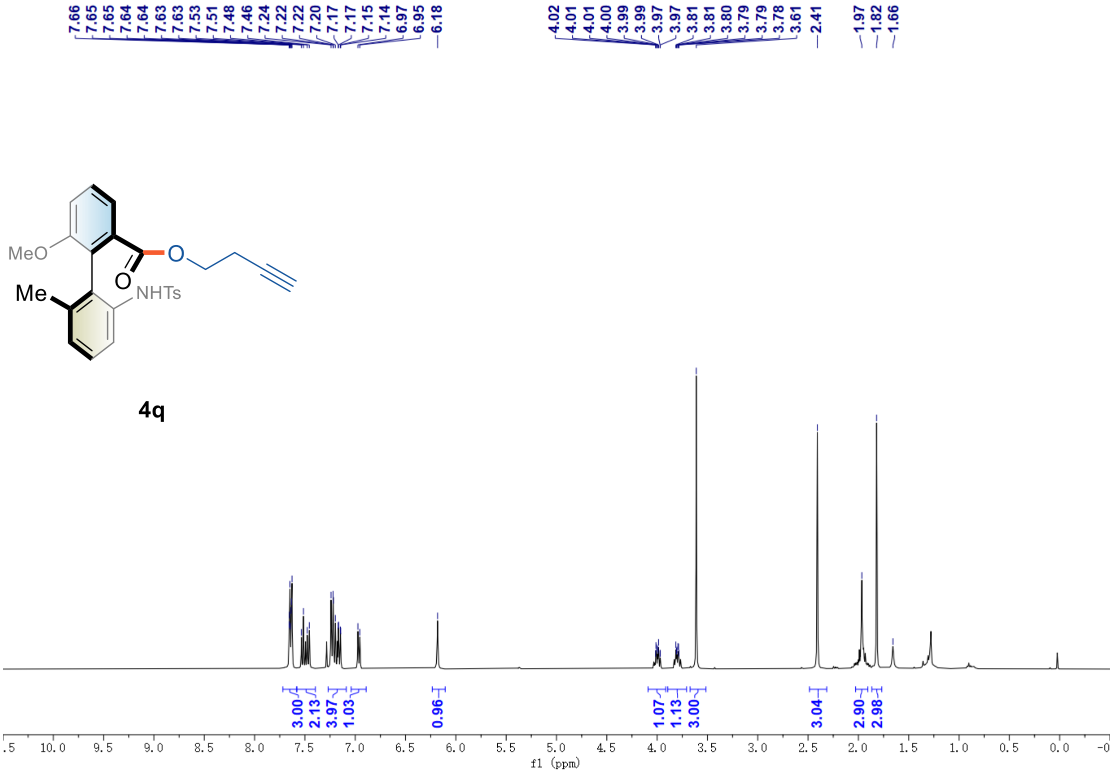

Supplementary Figure 58. ^{1}**H NMR** of the **4q** (400 MHz, $CDCl_3$)

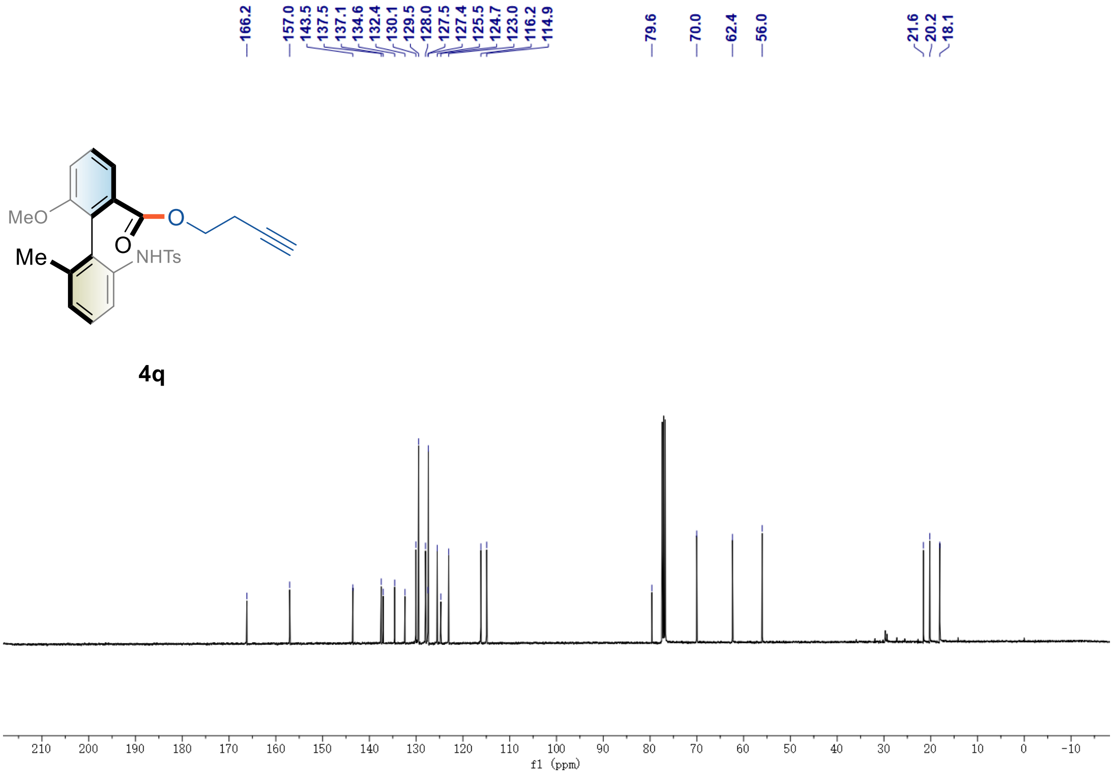

Supplementary Figure 59. ^{13}**C NMR** of the **4q** (101 MHz, $CDCl_3$)

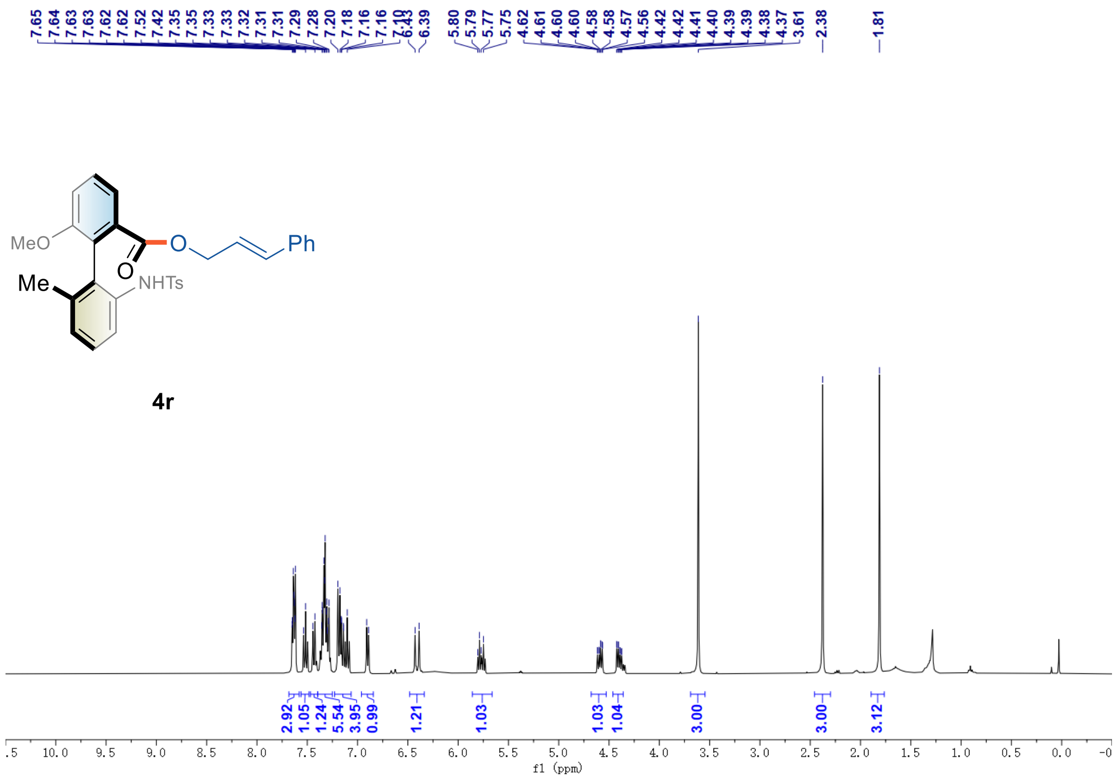

Supplementary Figure 60. ^{1}**H NMR** of the **4r** (400 MHz, $CDCl_3$)

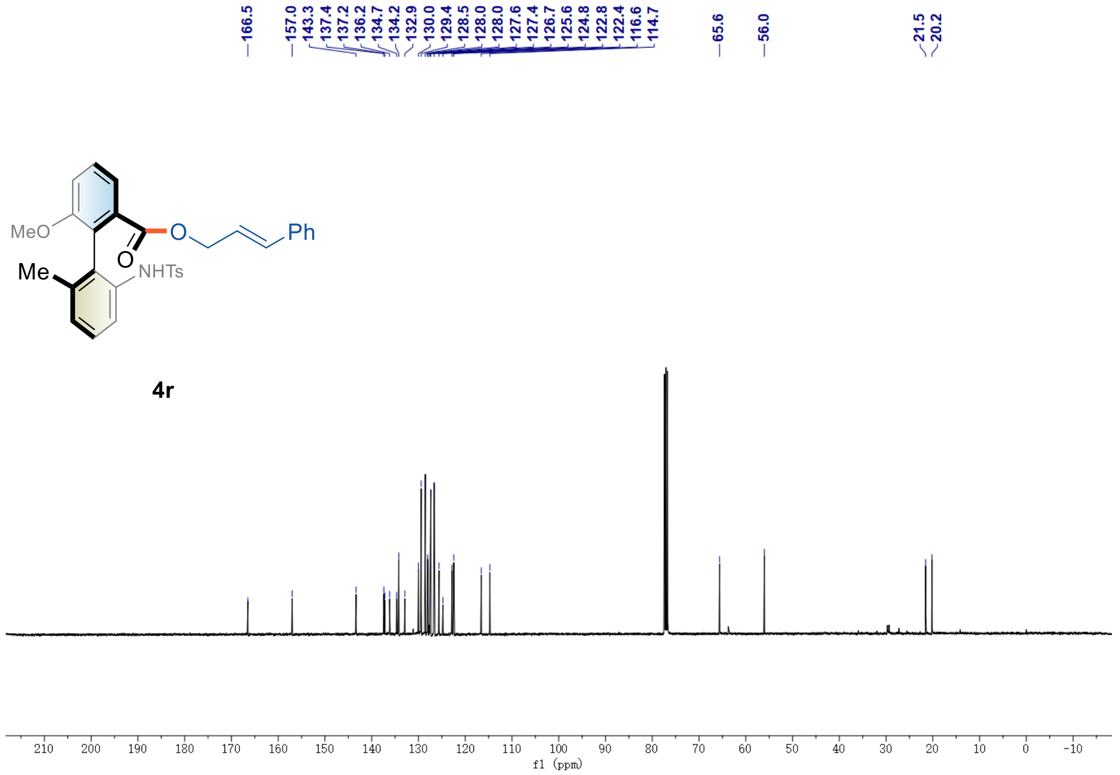

Supplementary Figure 61. ^{13}**C NMR** of the **4r** (101 MHz, $CDCl_3$)

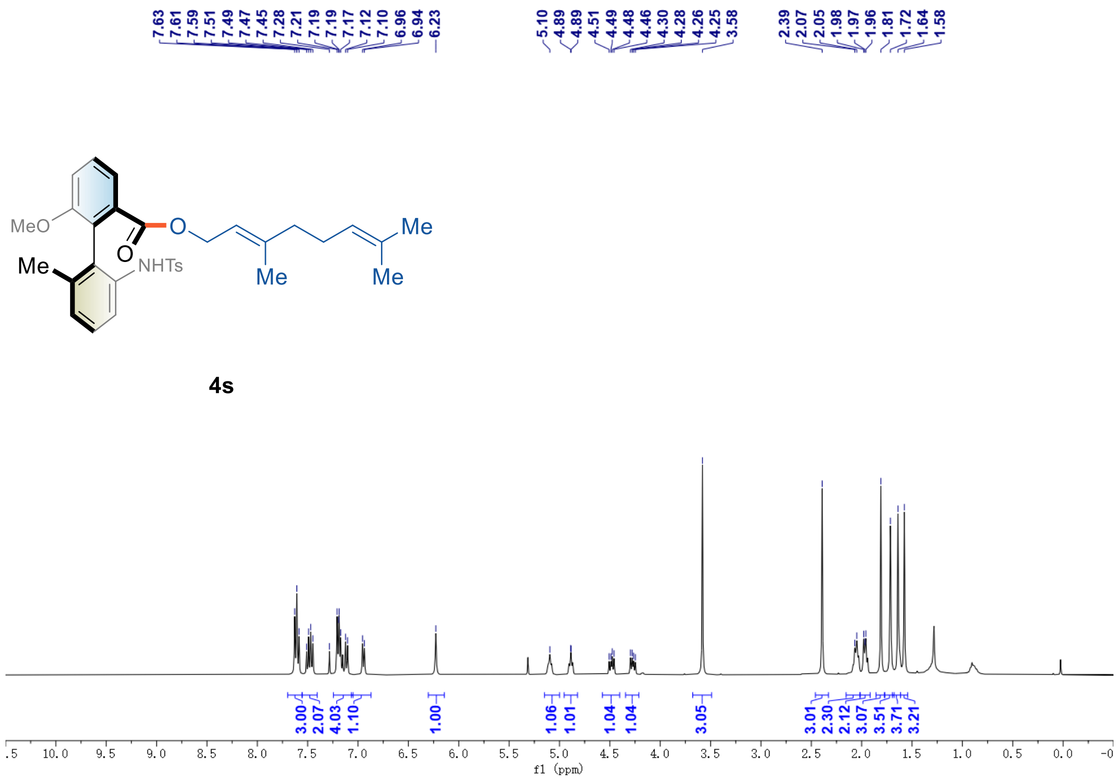

Supplementary Figure 62. ^{1}**H NMR** of the **4s** (400 MHz, $CDCl_3$)

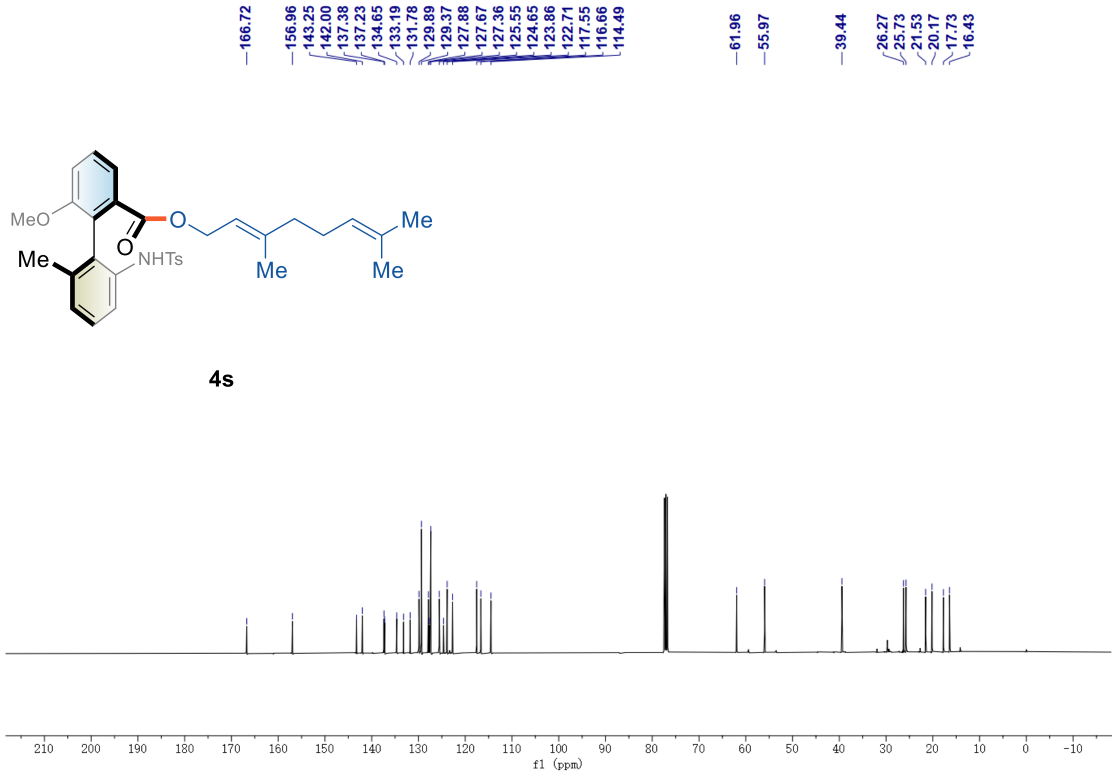

Supplementary Figure 63. ^{13}**C NMR** of the **4s** (101 MHz, $CDCl_3$)

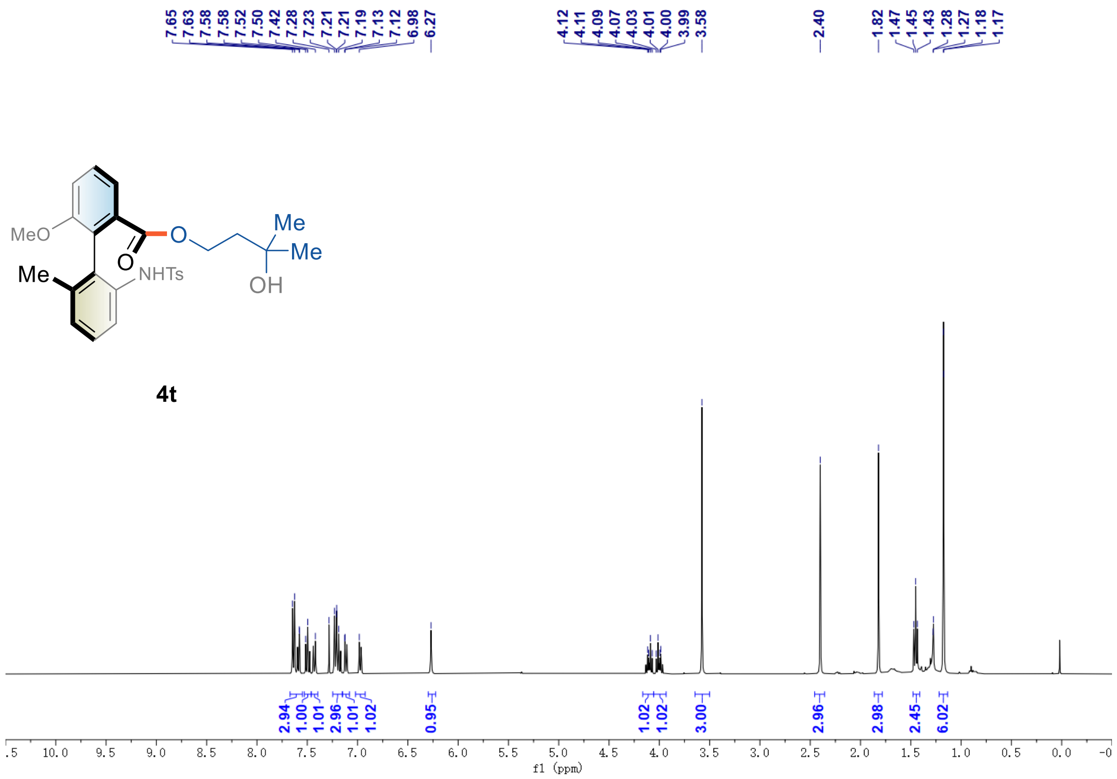

Supplementary Figure 64. ^{1}H NMR of the **4t** (400 MHz, $CDCl_3$)

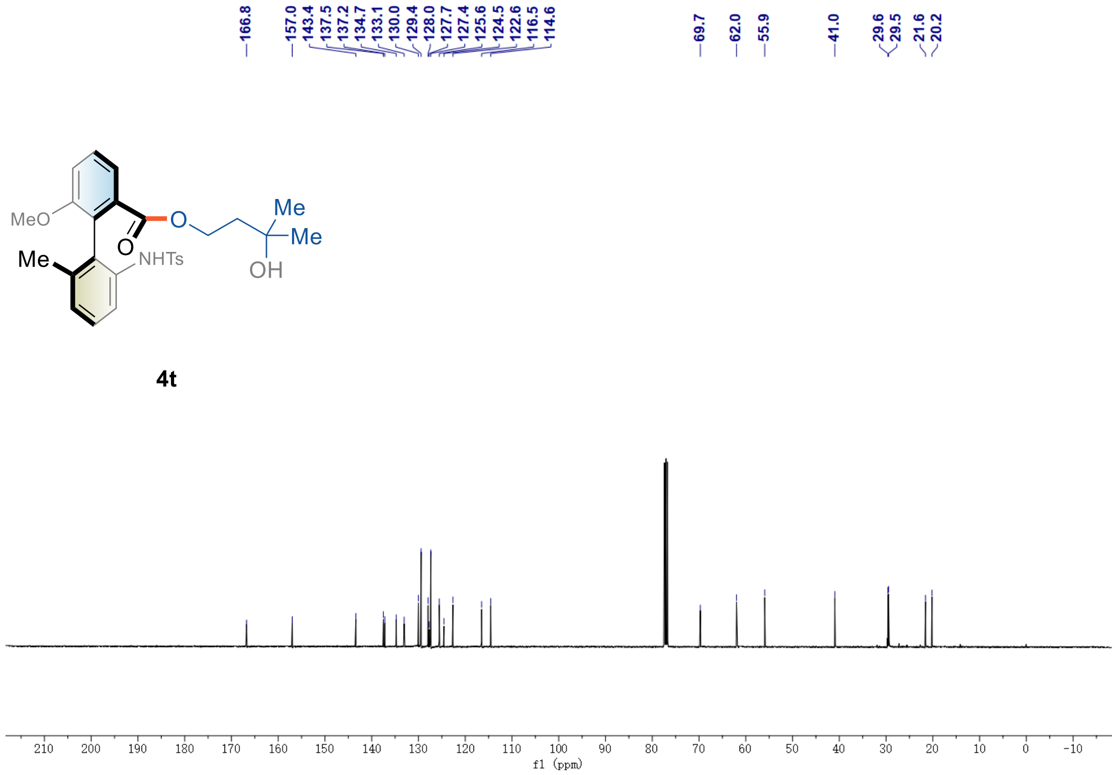

Supplementary Figure 65. ^{13}C NMR of the **4t** (101 MHz, $CDCl_3$)

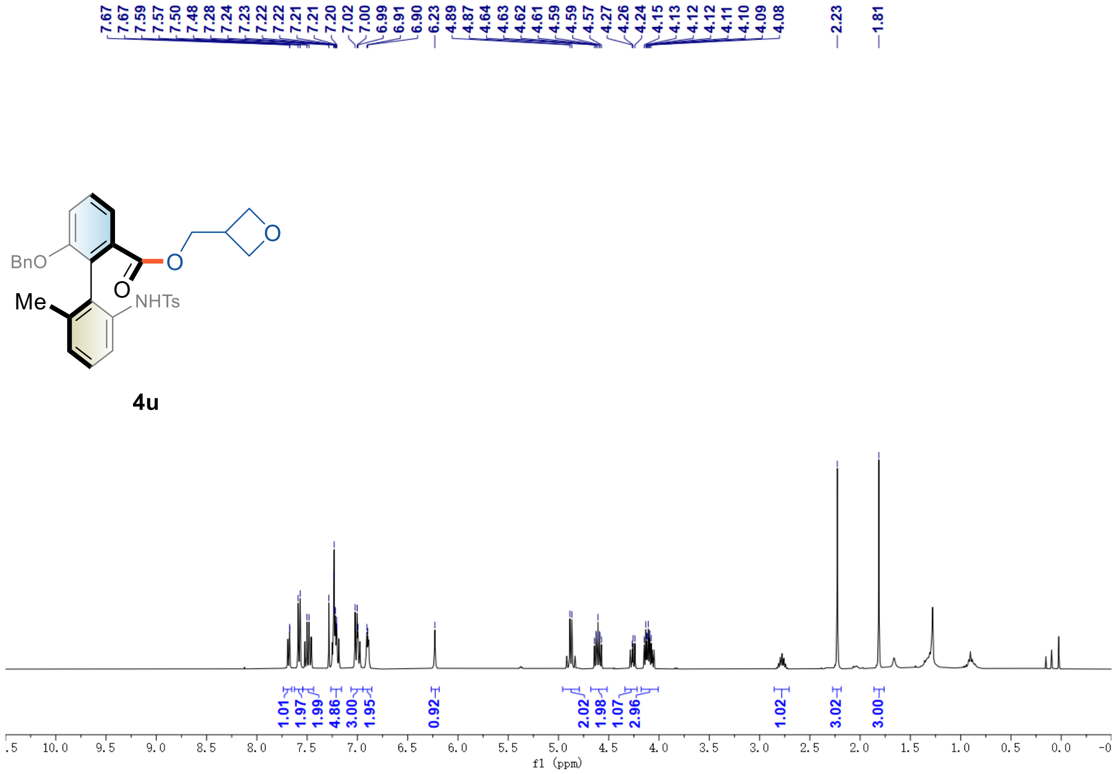

Supplementary Figure 66. ^{1}**H NMR** of the **4u** (400 MHz, $CDCl_3$)

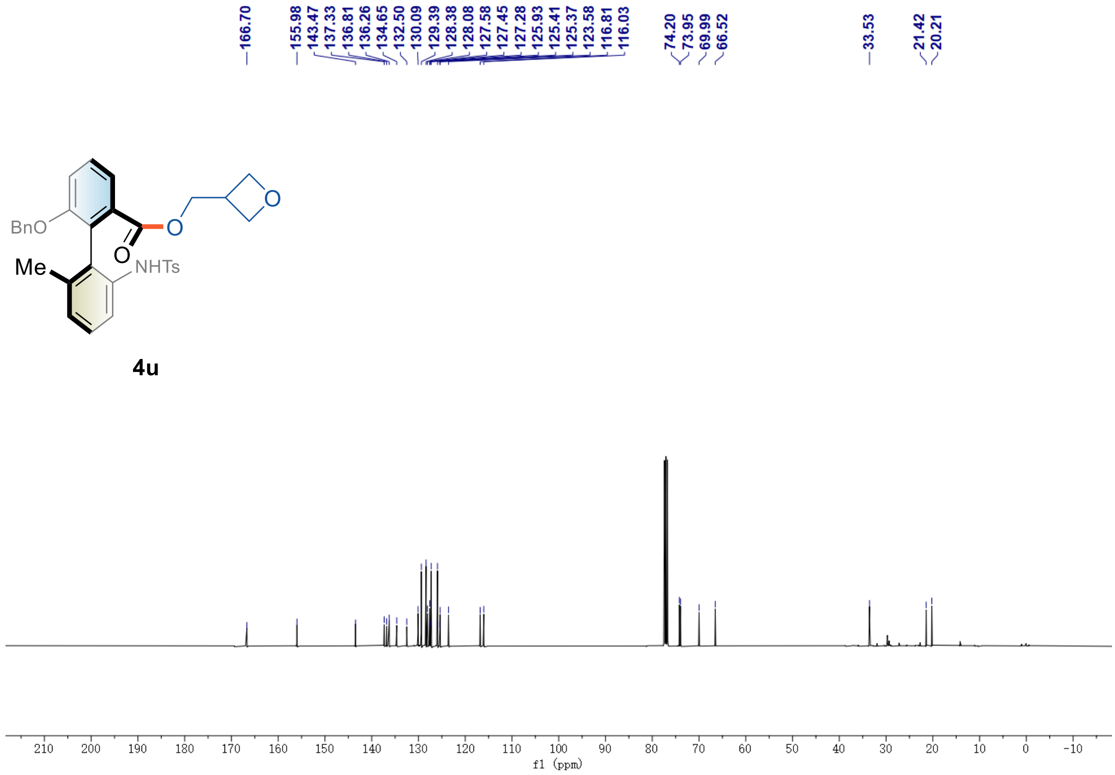

Supplementary Figure 67. ^{13}**C NMR** of the **4u** (101 MHz, $CDCl_3$)

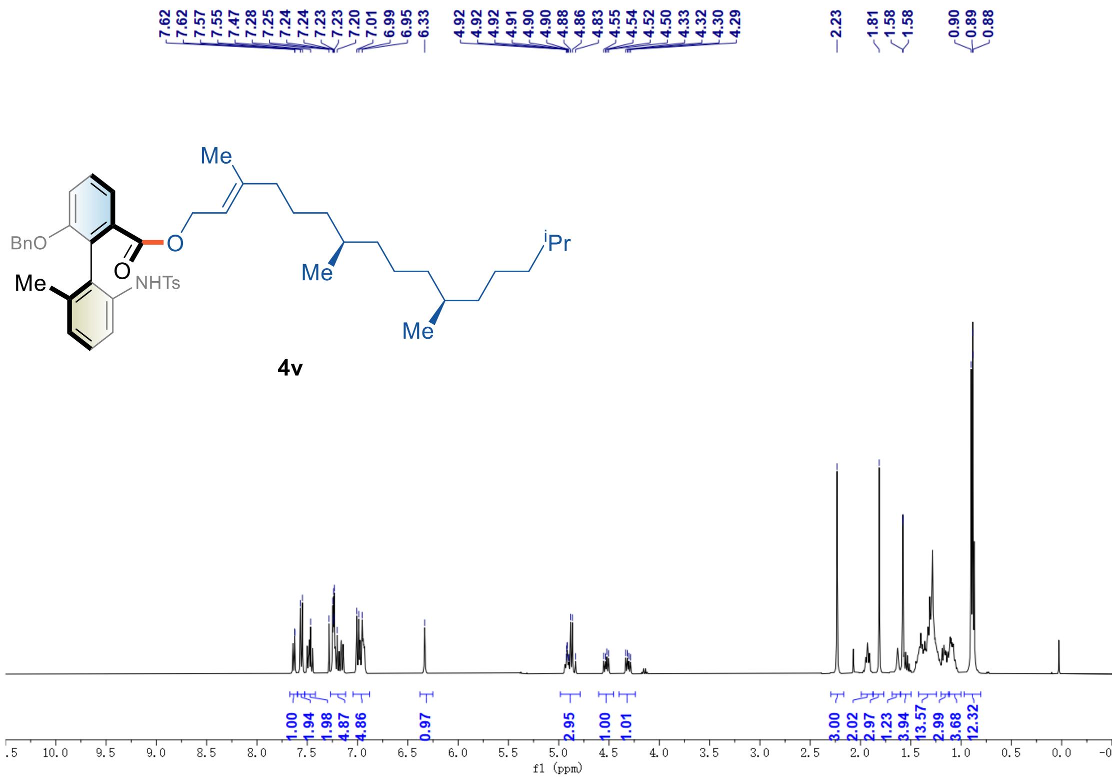

Supplementary Figure 68. ^{1}**H NMR** of the **4v** (400 MHz, $CDCl_3$)

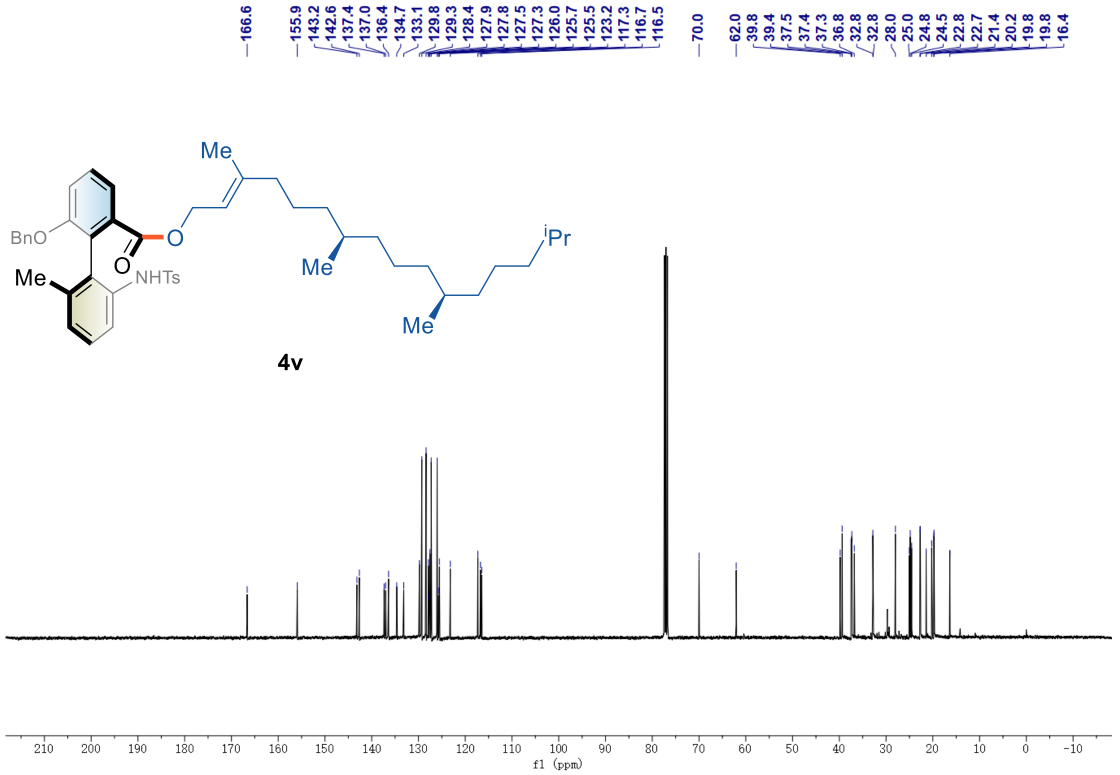

Supplementary Figure 69. ^{13}**C NMR** of the **4v** (101 MHz, $CDCl_3$)

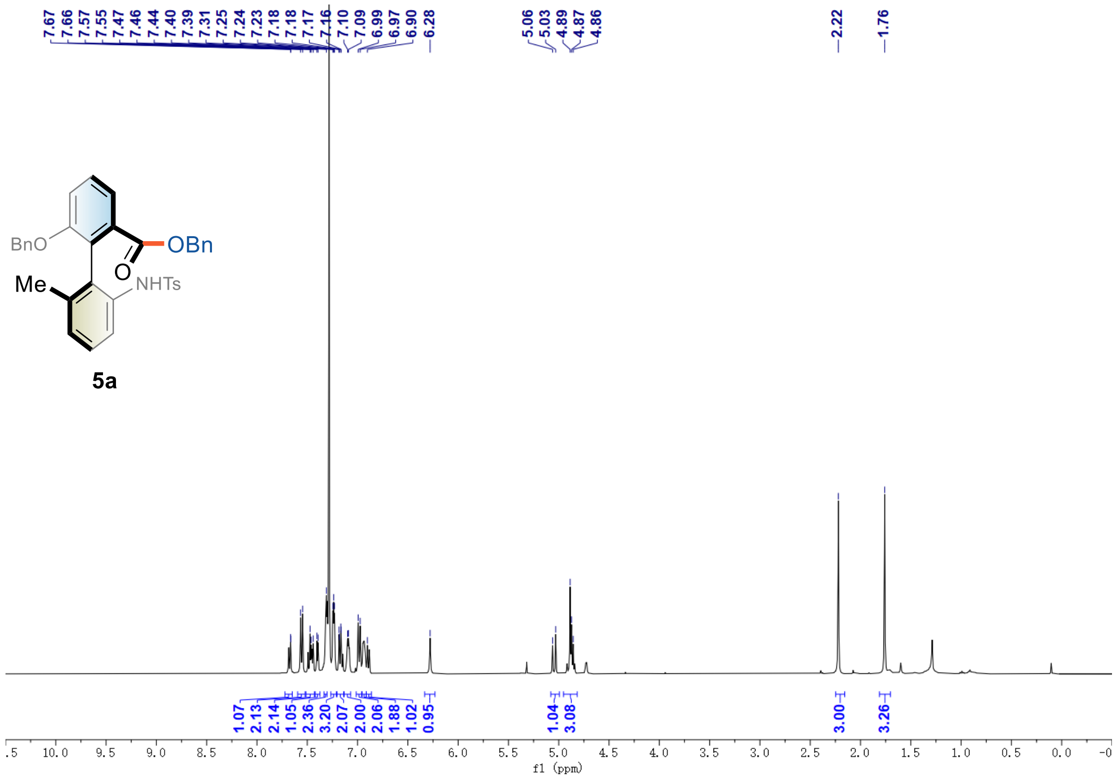

Supplementary Figure 70. ^{1}**H NMR** of the **5a** (400 MHz, $CDCl_3$)

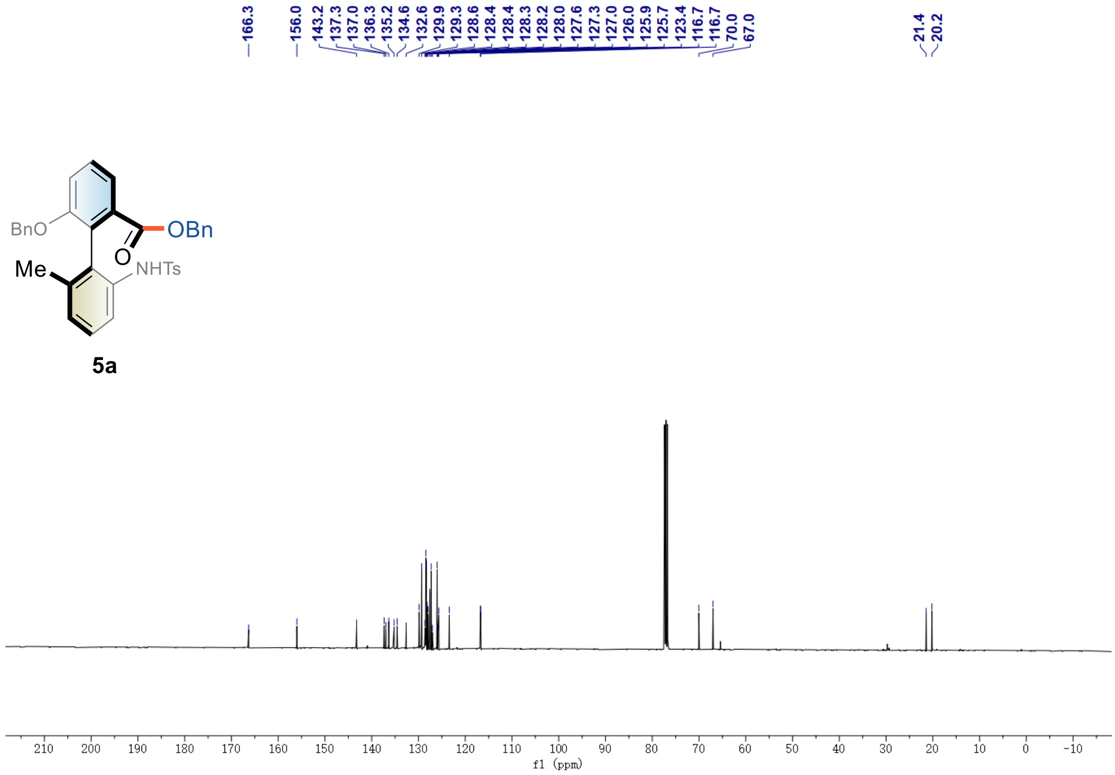

Supplementary Figure 71. ^{13}**C NMR** of the **5a** (101 MHz, $CDCl_3$)

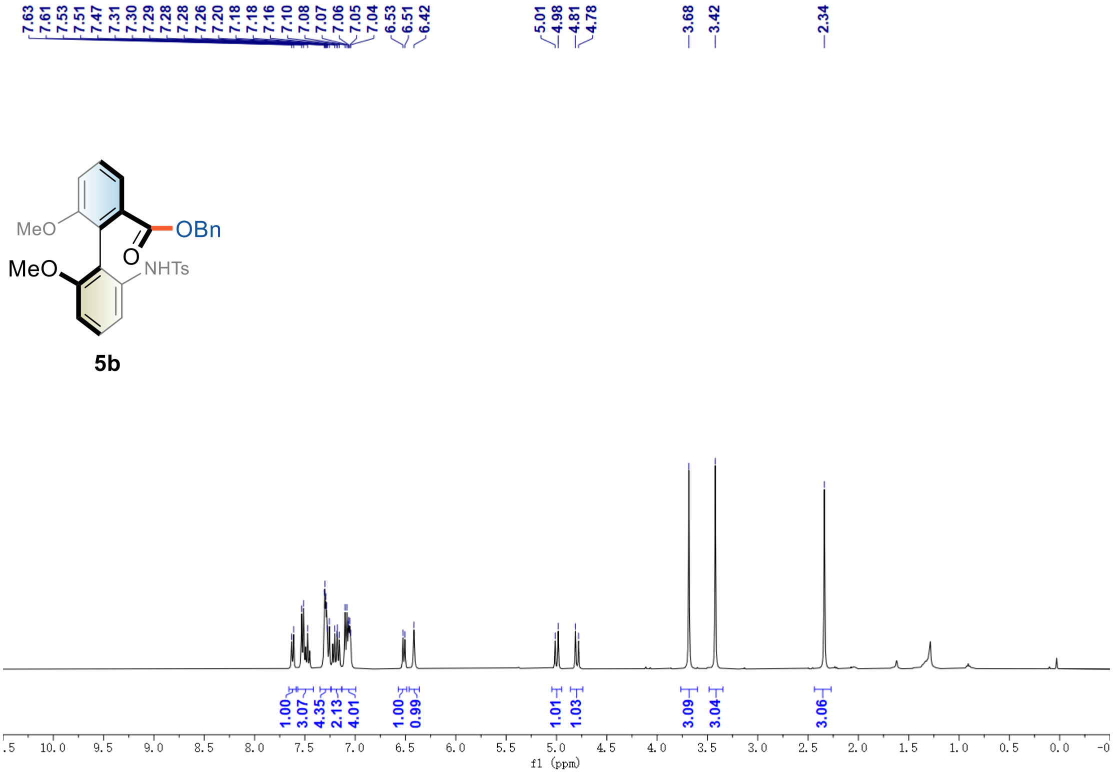

Supplementary Figure 72. ^{1}**H NMR** of the **5b** (400 MHz, $CDCl_3$)

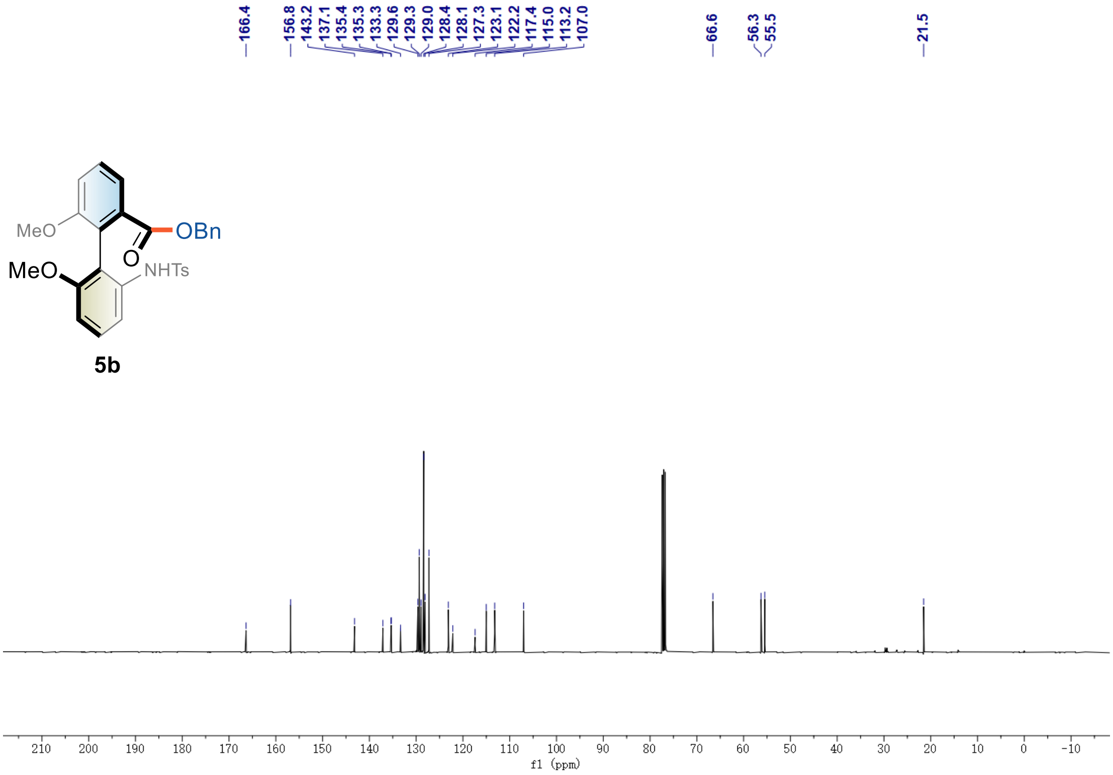

Supplementary Figure 73. ^{13}**C NMR** of the **5b** (101 MHz, $CDCl_3$)

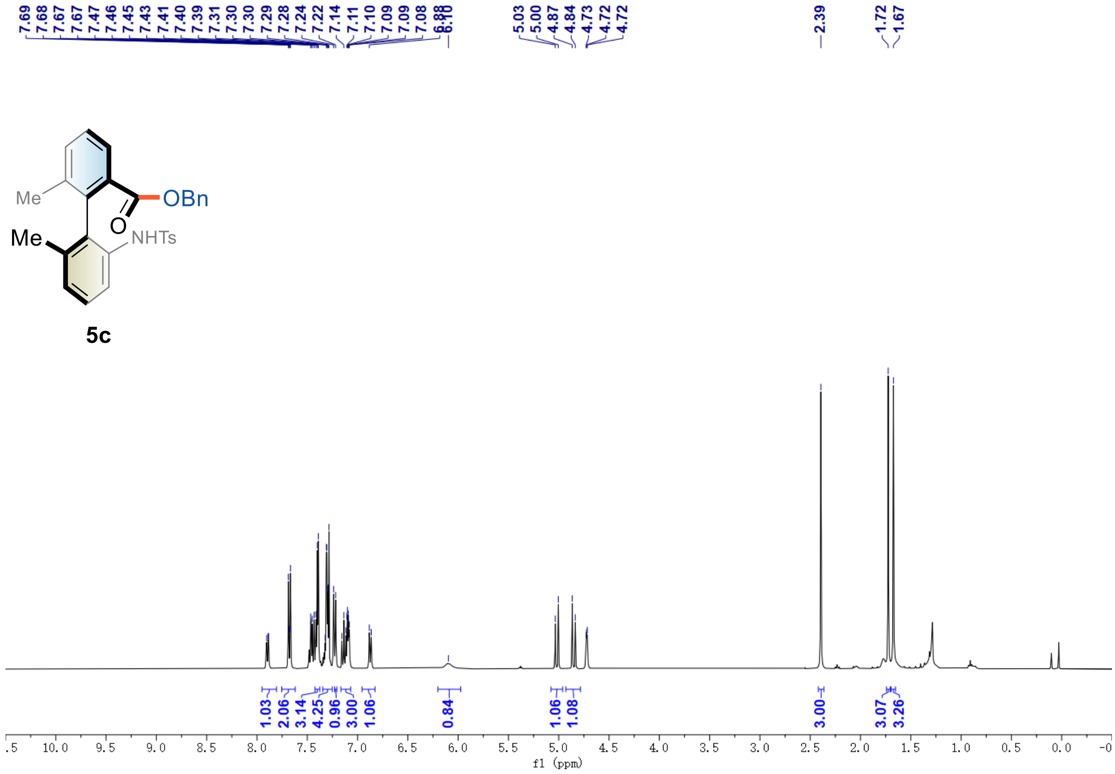

Supplementary Figure 74. ^{1}**H NMR** of the **5c** (400 MHz, $CDCl_3$)

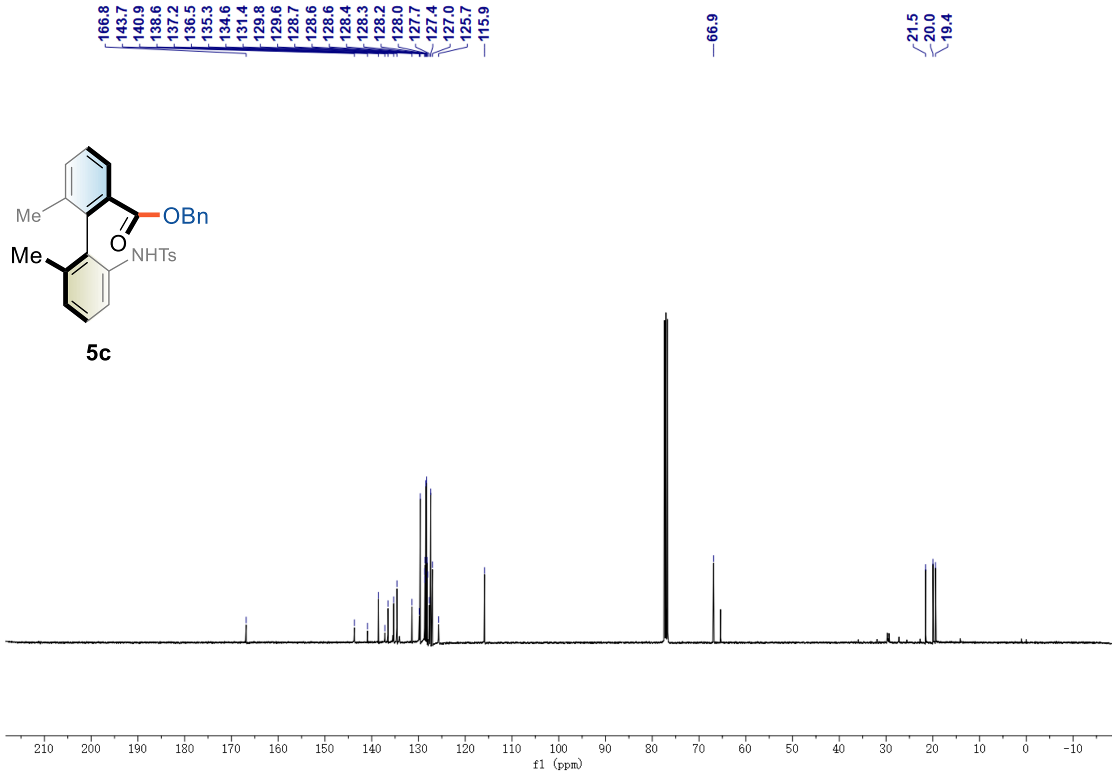

Supplementary Figure 75. ^{13}**C NMR** of the **5c** (101 MHz, $CDCl_3$)

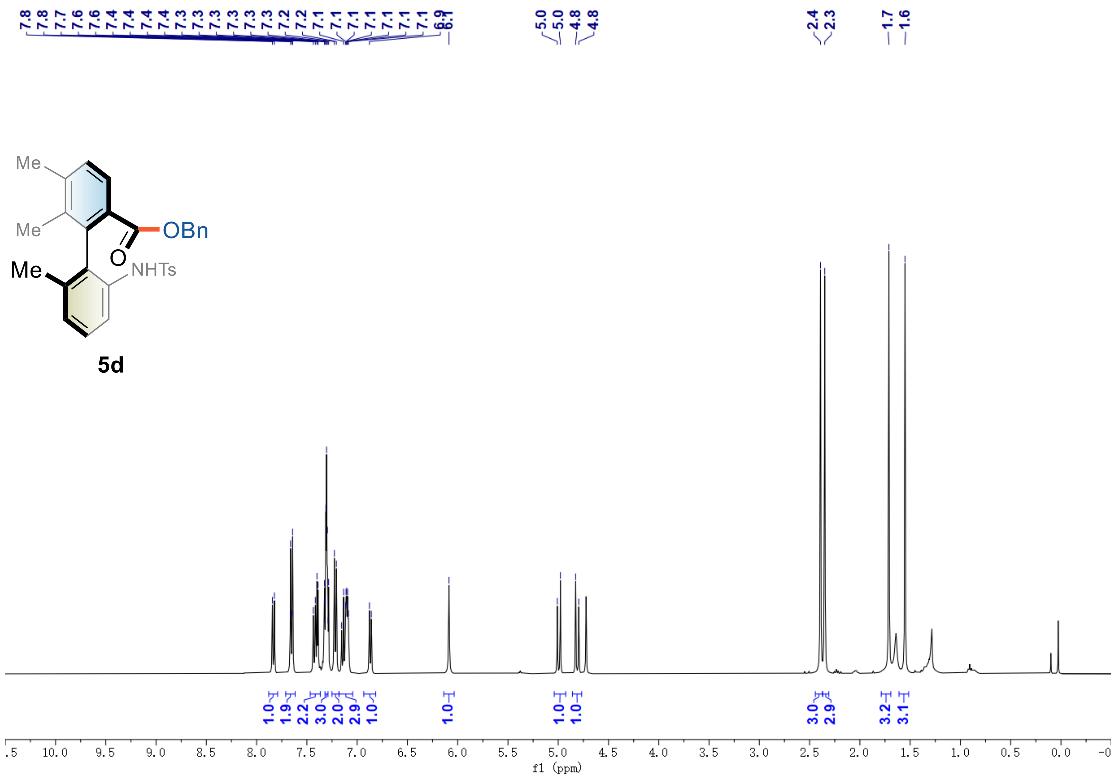

Supplementary Figure 76. ^{1}H NMR of the **5d** (400 MHz, $CDCl_3$)

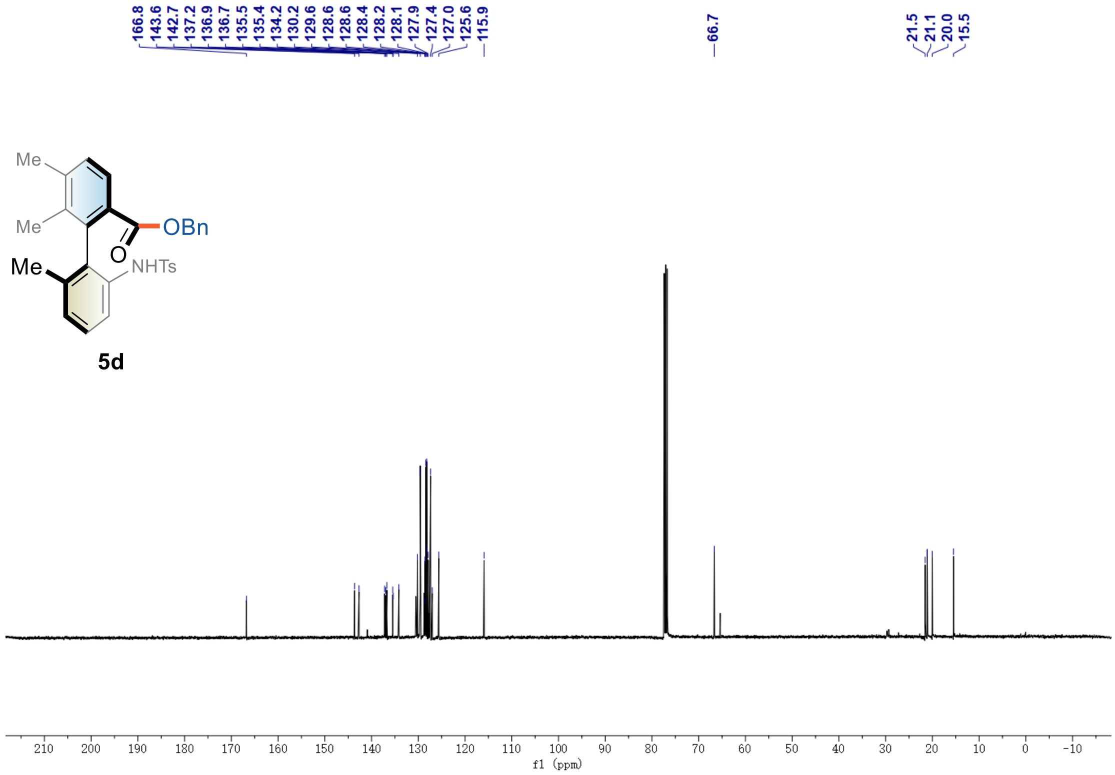

Supplementary Figure 77. ^{13}C NMR of the **5d** (101 MHz, $CDCl_3$)

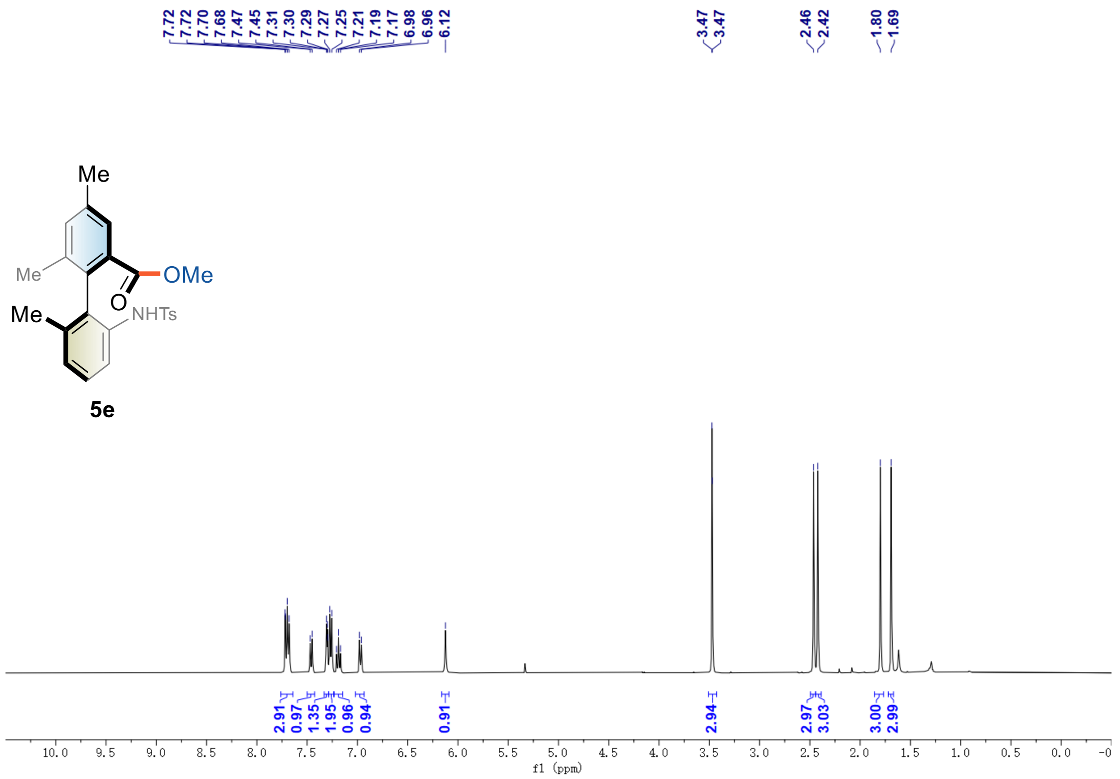

Supplementary Figure 78. ^{1}**H NMR** of the **5e** (400 MHz, $CDCl_3$)

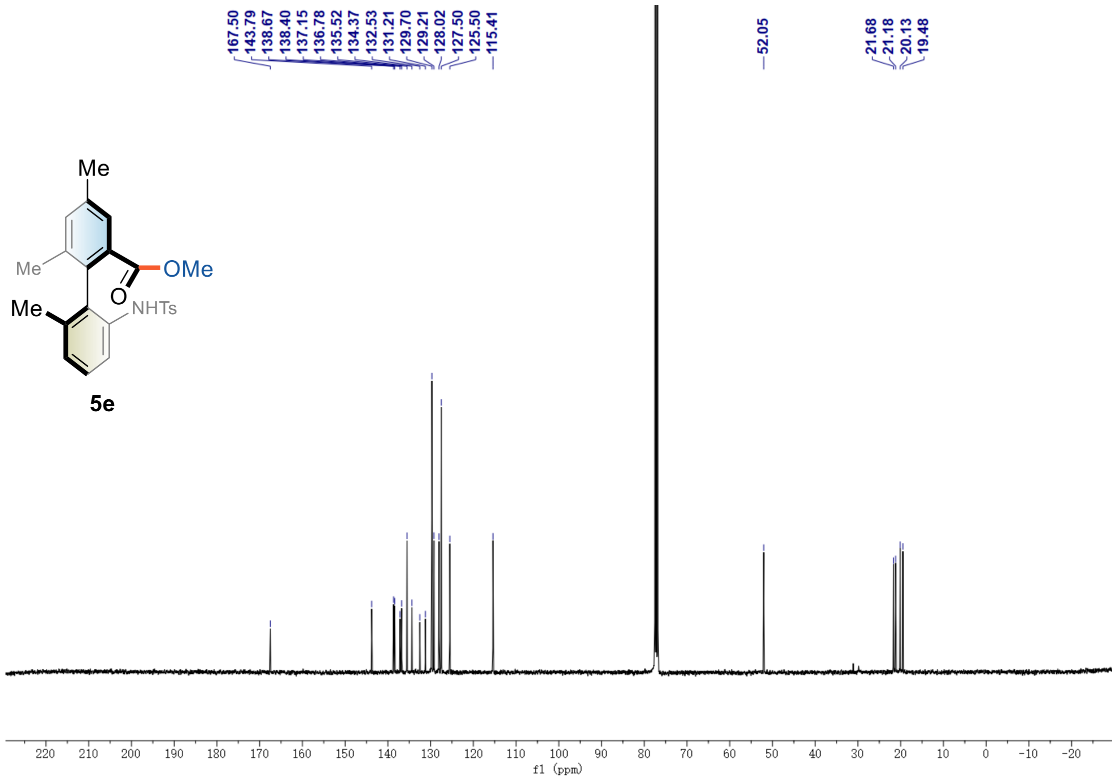

Supplementary Figure 79. ^{13}**C NMR** of the **5e** (101 MHz, $CDCl_3$)

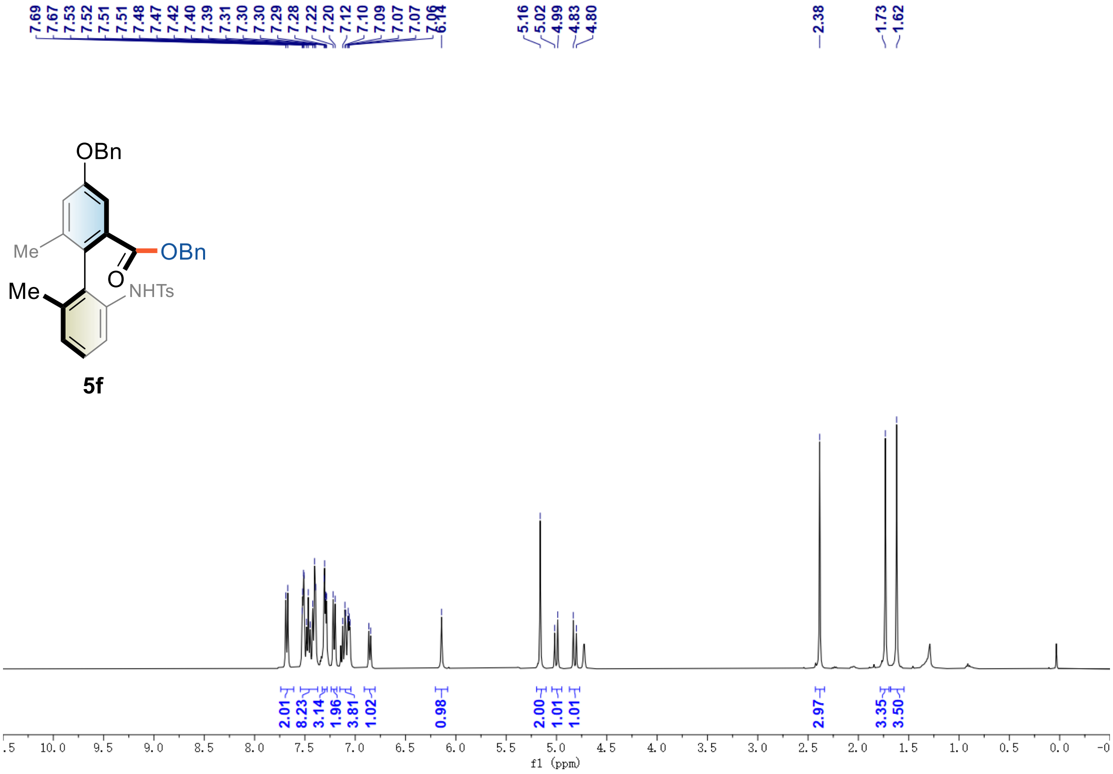

Supplementary Figure 80. ^{1}**H NMR** of the **5f** (400 MHz, $CDCl_3$)

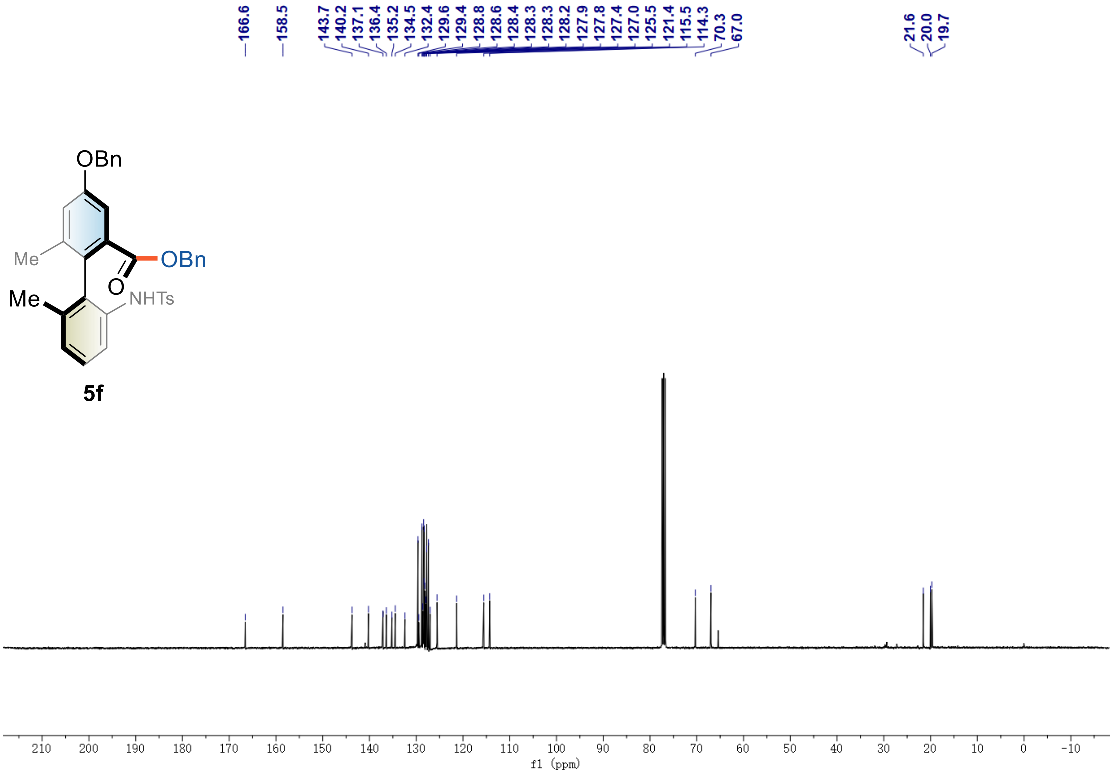

Supplementary Figure 81. ^{13}**C NMR** of the **5f** (101 MHz, $CDCl_3$)

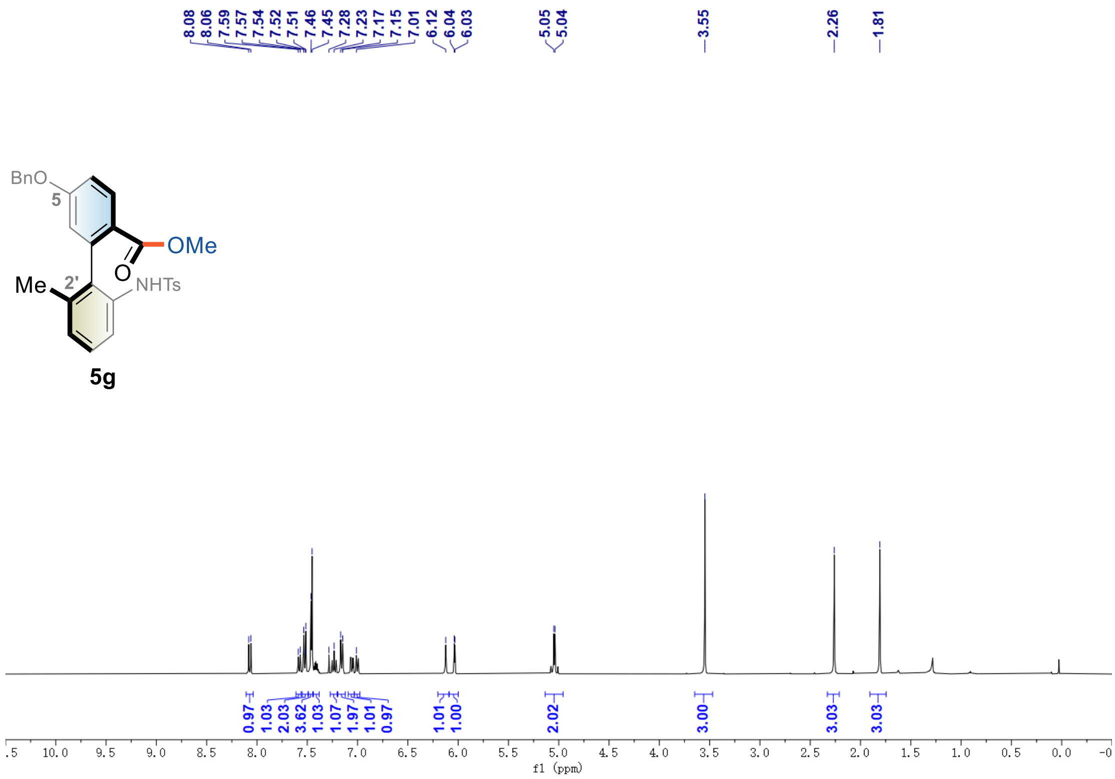

Supplementary Figure 82. **^{1}H NMR** of the **5g** (400 MHz, $CDCl_3$)

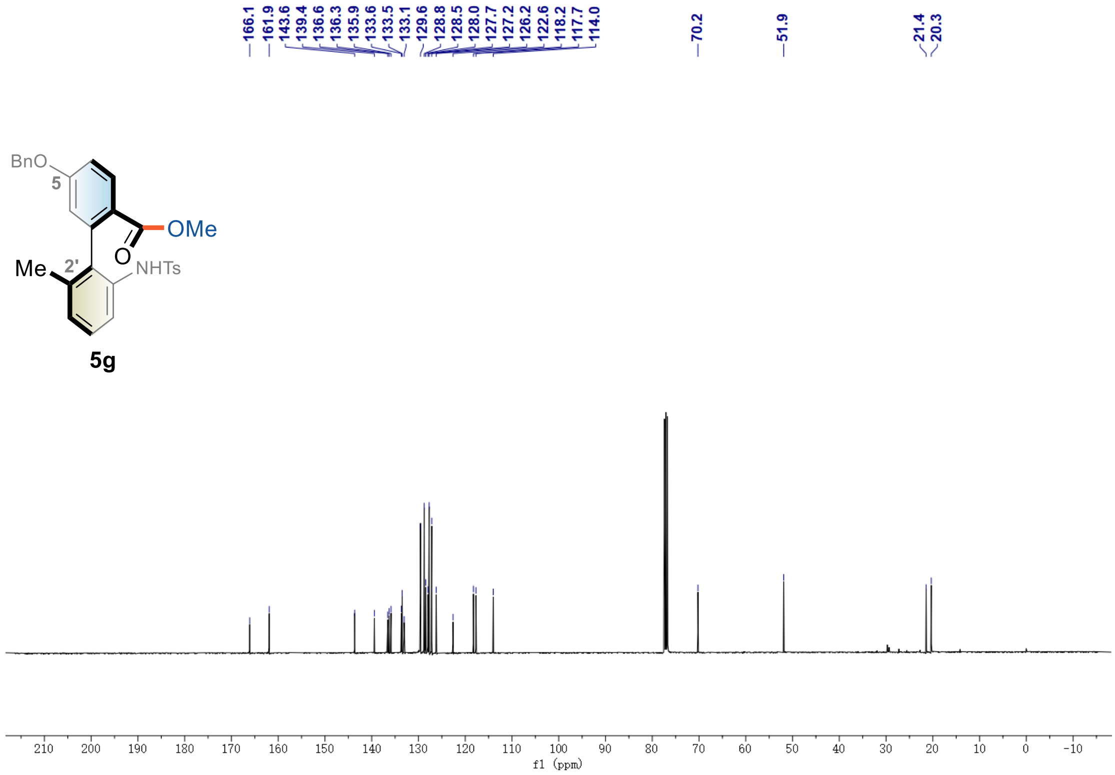

Supplementary Figure 83. **^{13}C NMR** of the **5g** (101 MHz, $CDCl_3$)

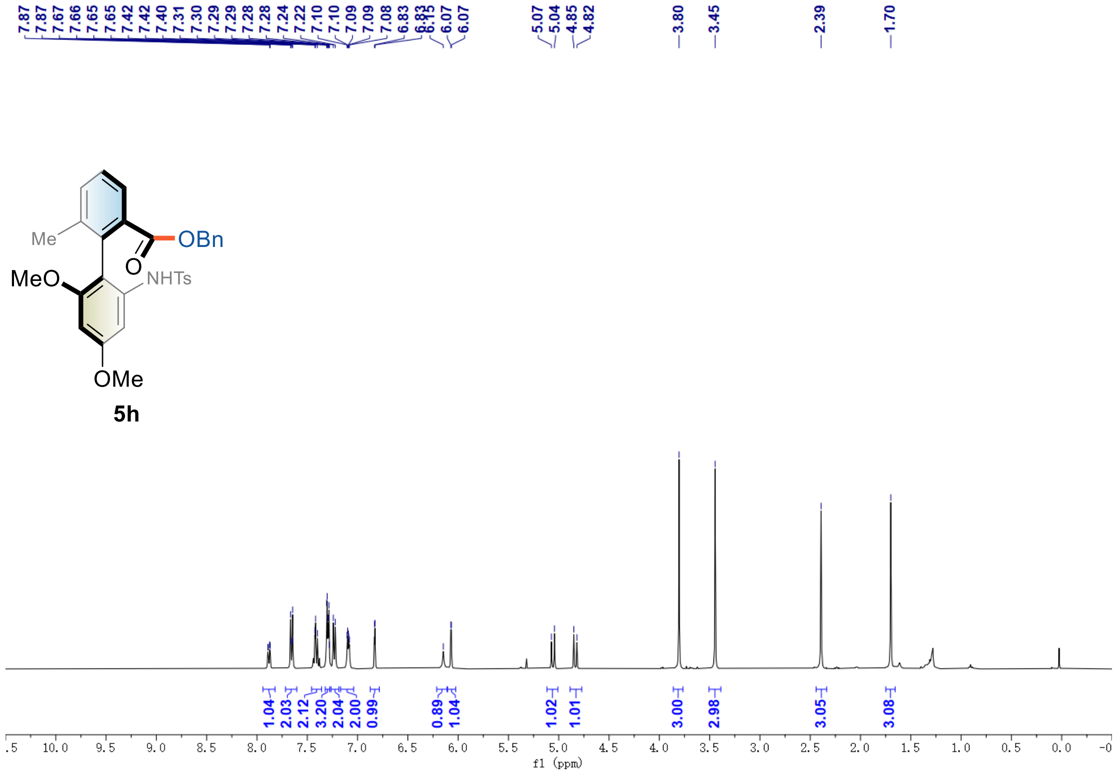

Supplementary Figure 84. **^{1}H NMR** of the **5h** (400 MHz, $CDCl_3$)

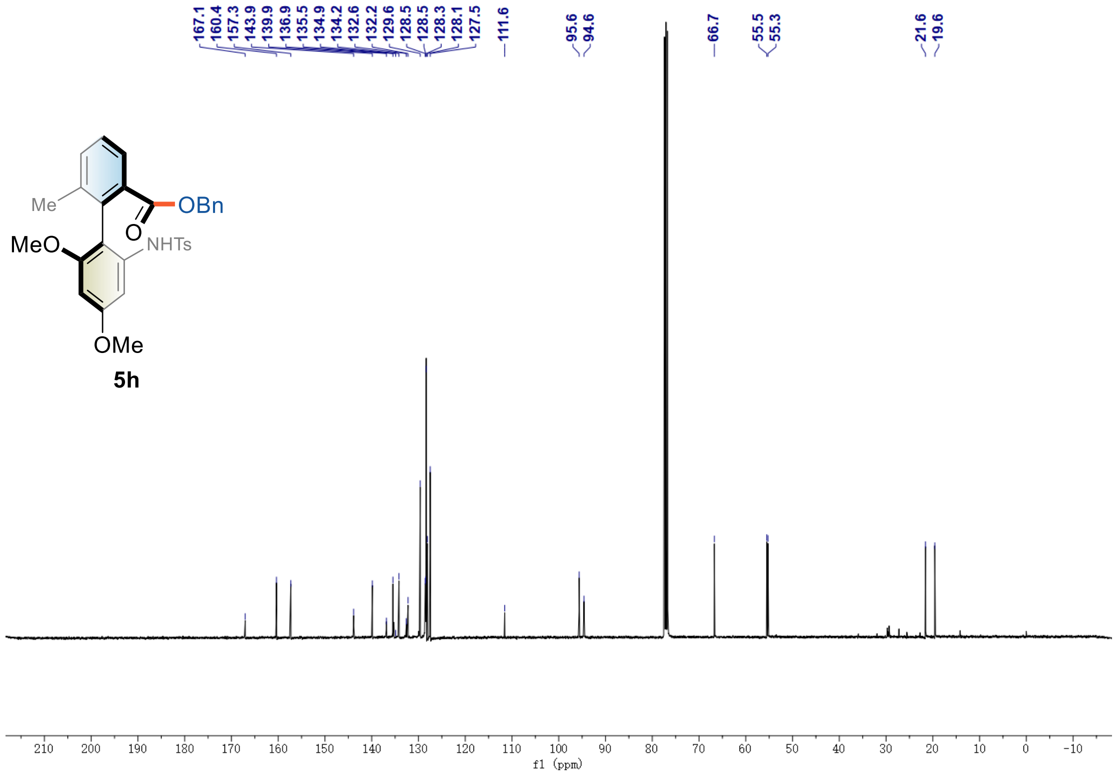

Supplementary Figure 85. **^{13}C NMR** of the **5h** (101 MHz, $CDCl_3$)

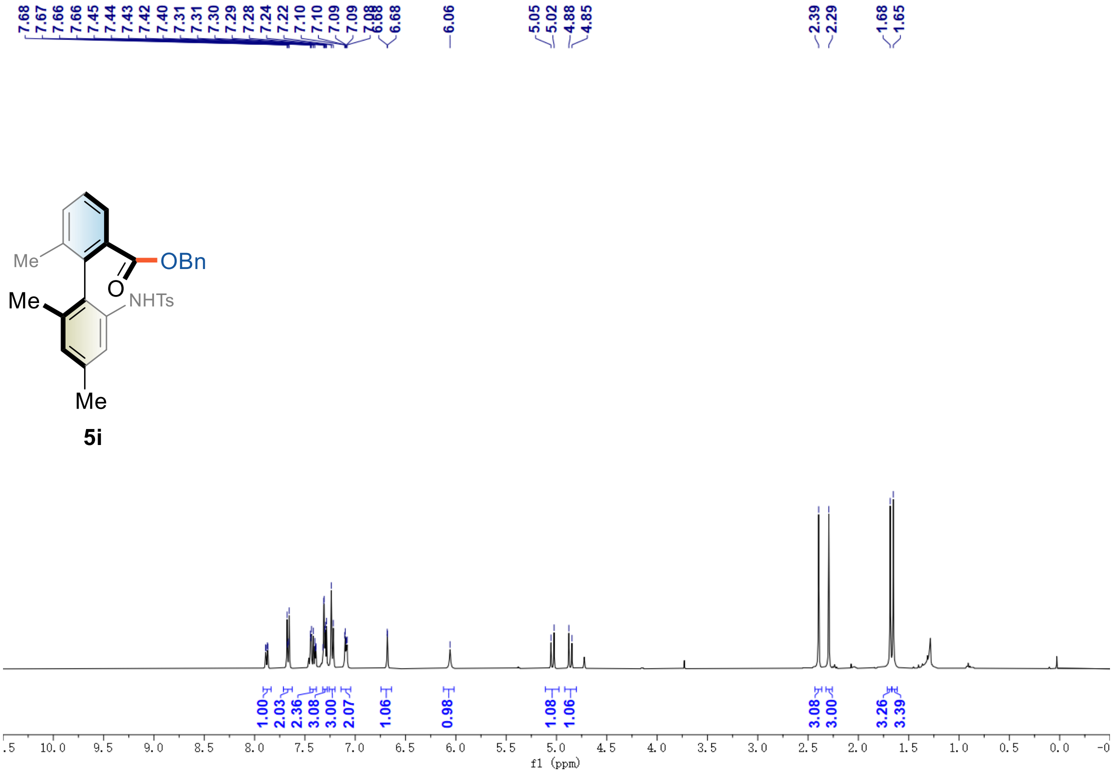

Supplementary Figure 86. ^{1}**H NMR** of the **5i** (400 MHz, $CDCl_3$)

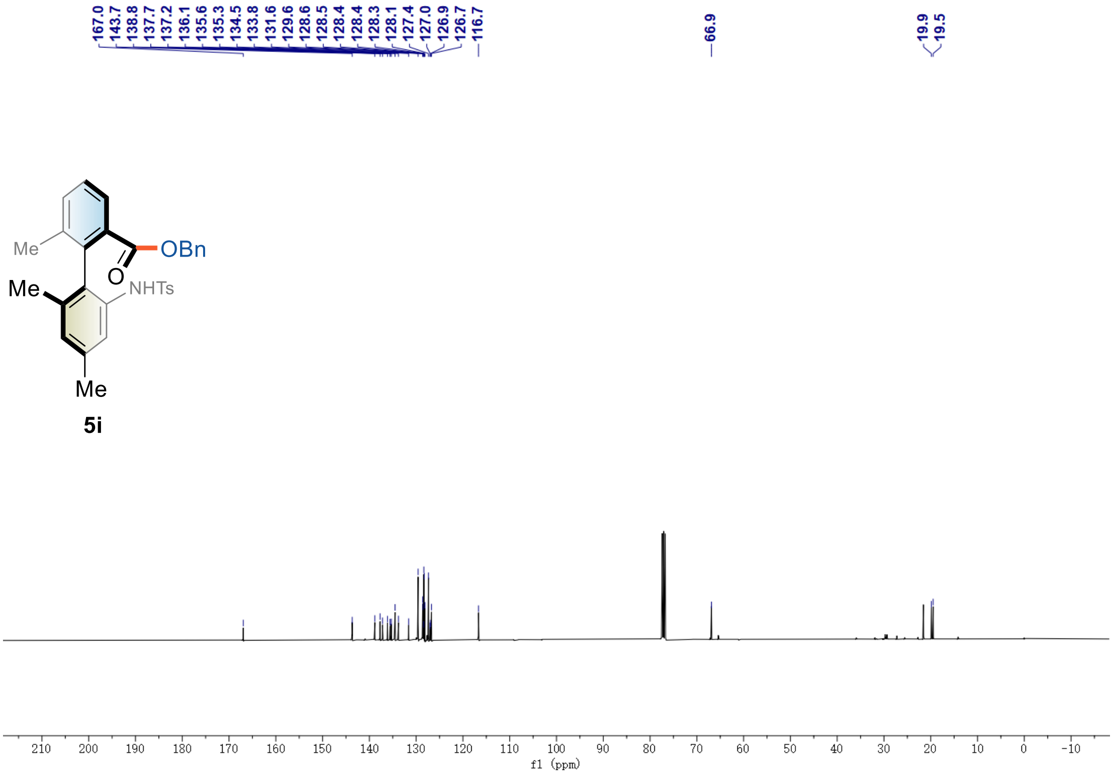

Supplementary Figure 87. ^{13}**C NMR** of the **5i** (101 MHz, $CDCl_3$)

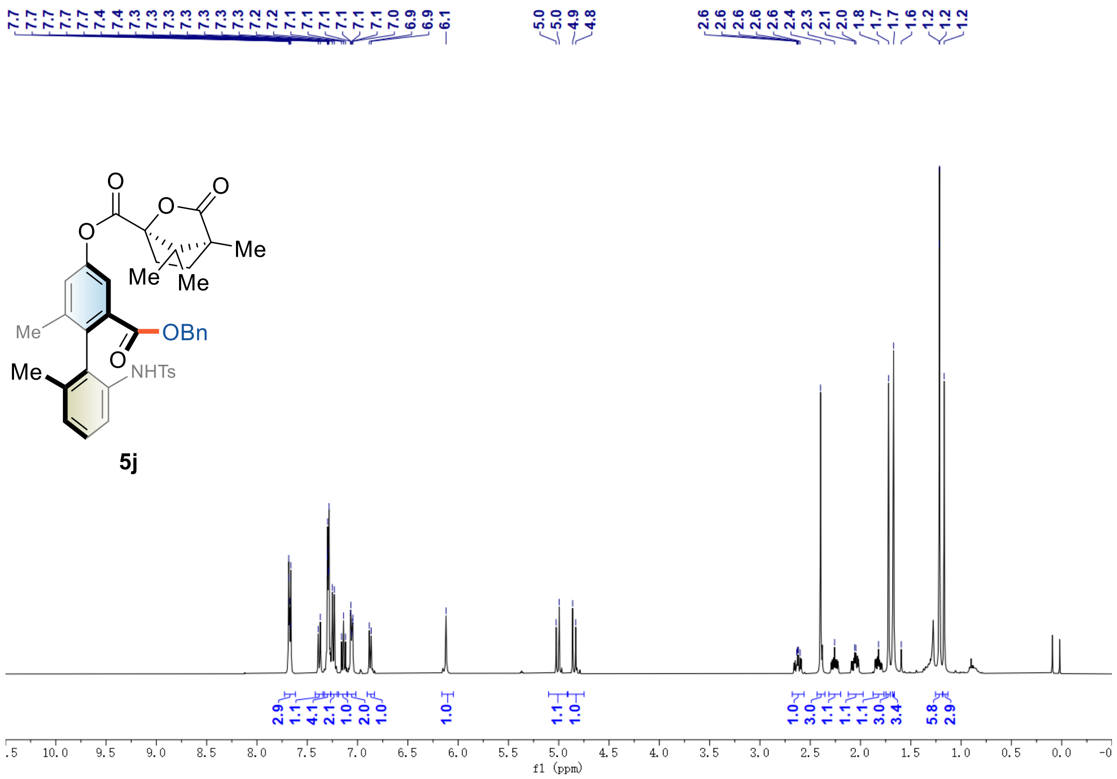

Supplementary Figure 88. ^{1}H NMR of the **5j** (400 MHz, $CDCl_3$)

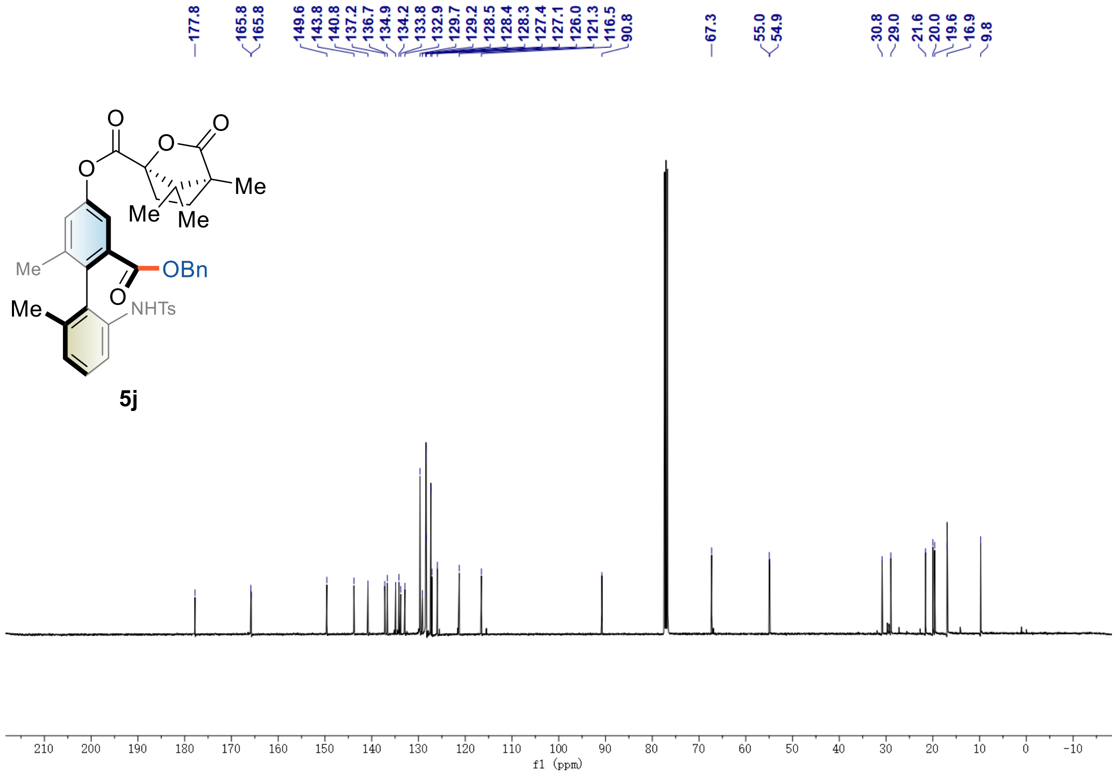

Supplementary Figure 89. ^{13}C NMR of the **5j** (101 MHz, $CDCl_3$)

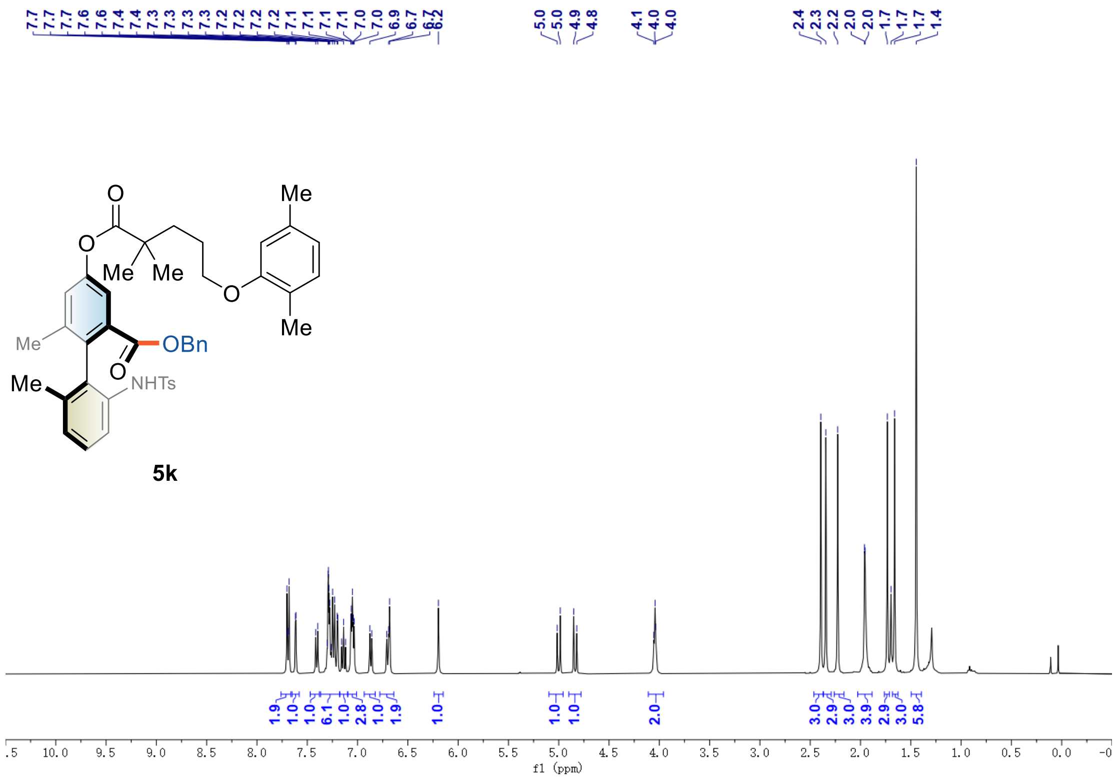

Supplementary Figure 90. **^{1}H NMR** of the **5k** (400 MHz, $CDCl_3$)

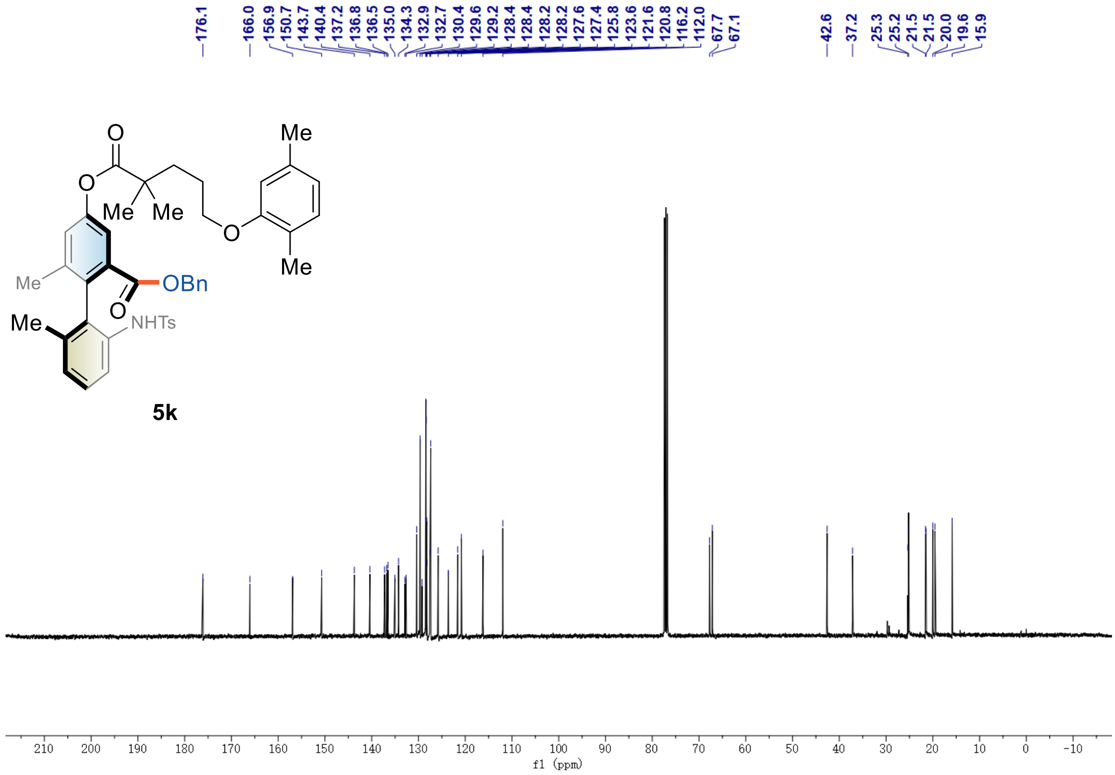

Supplementary Figure 91. **^{13}C NMR** of the **5k** (101 MHz, $CDCl_3$)

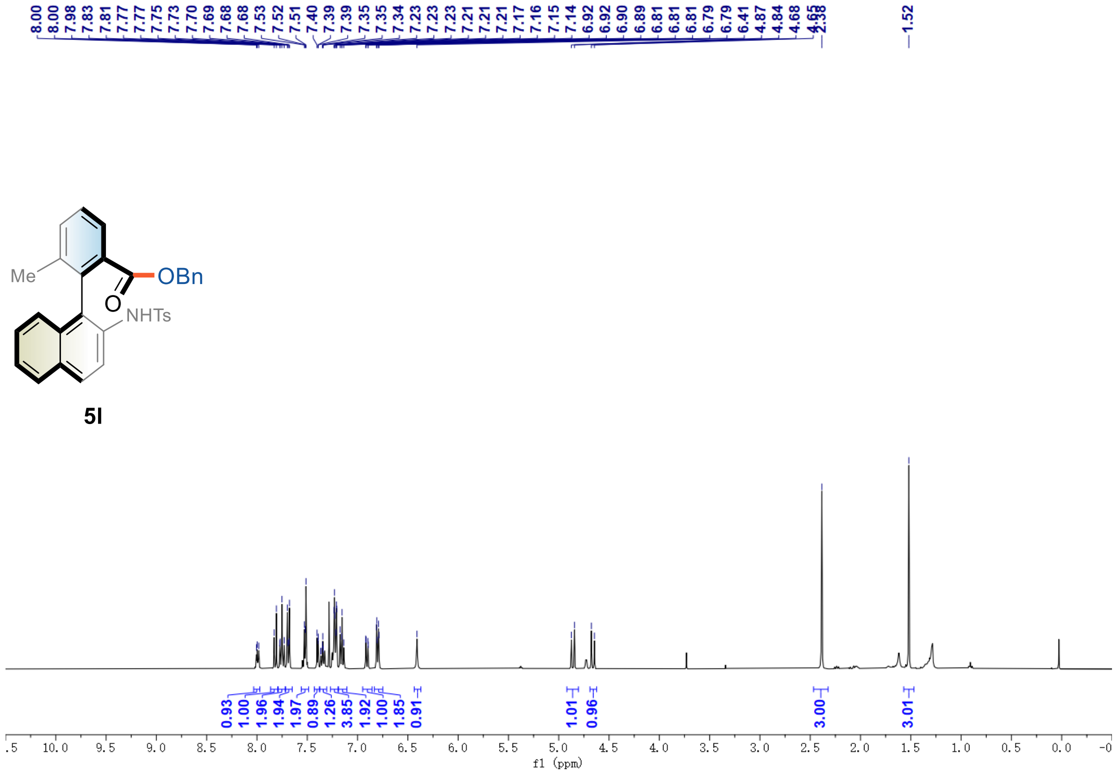

Supplementary Figure 92. ^{1}H NMR of the **5l** (400 MHz, $CDCl_3$)

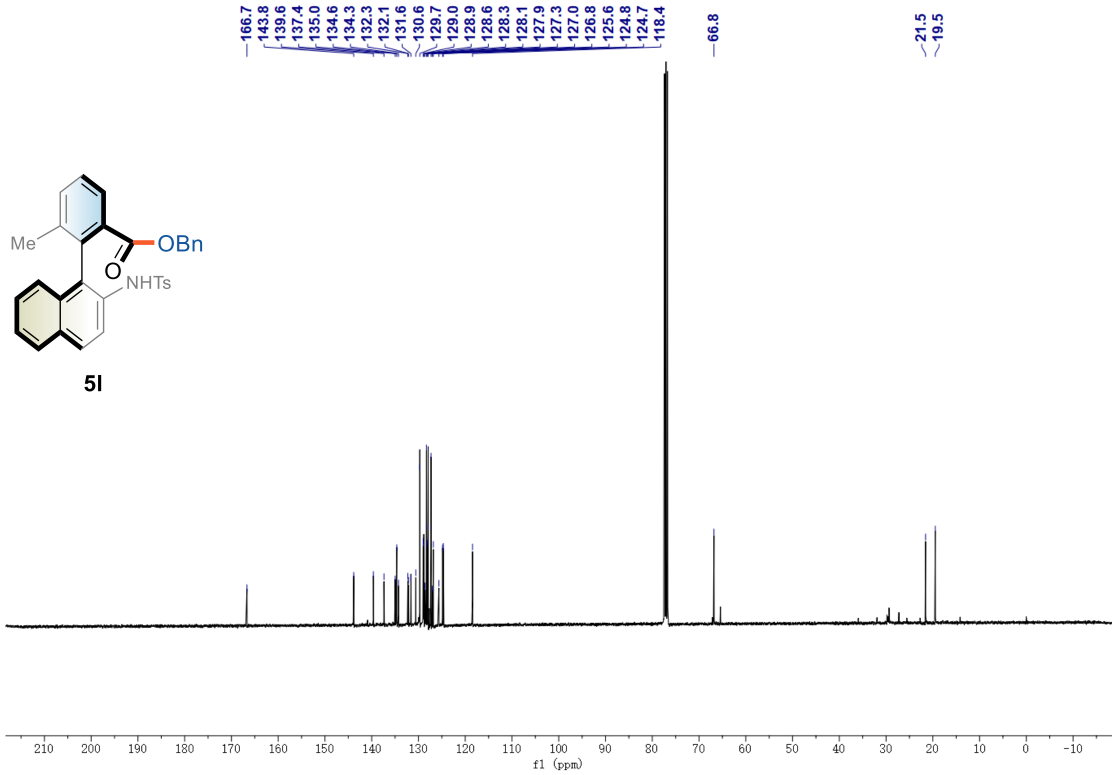

Supplementary Figure 93. ^{13}C NMR of the **5l** (101 MHz, $CDCl_3$)

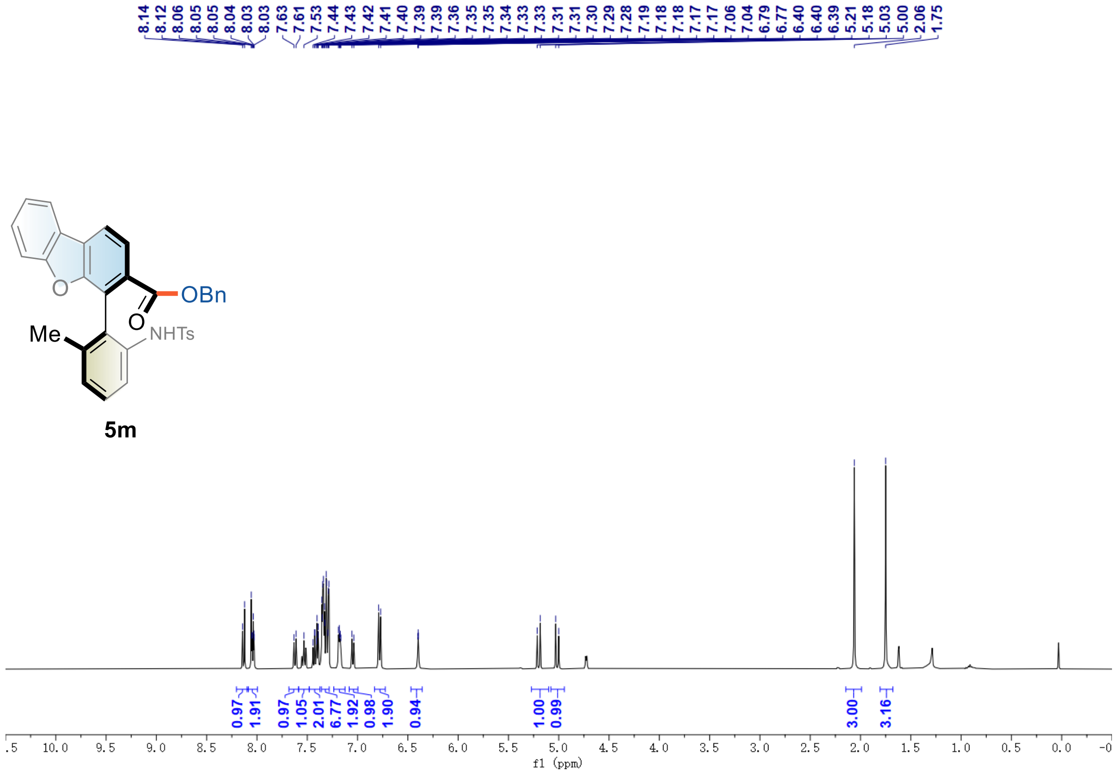

Supplementary Figure 94. **^{1}H NMR** of the **5m** (400 MHz, $CDCl_3$)

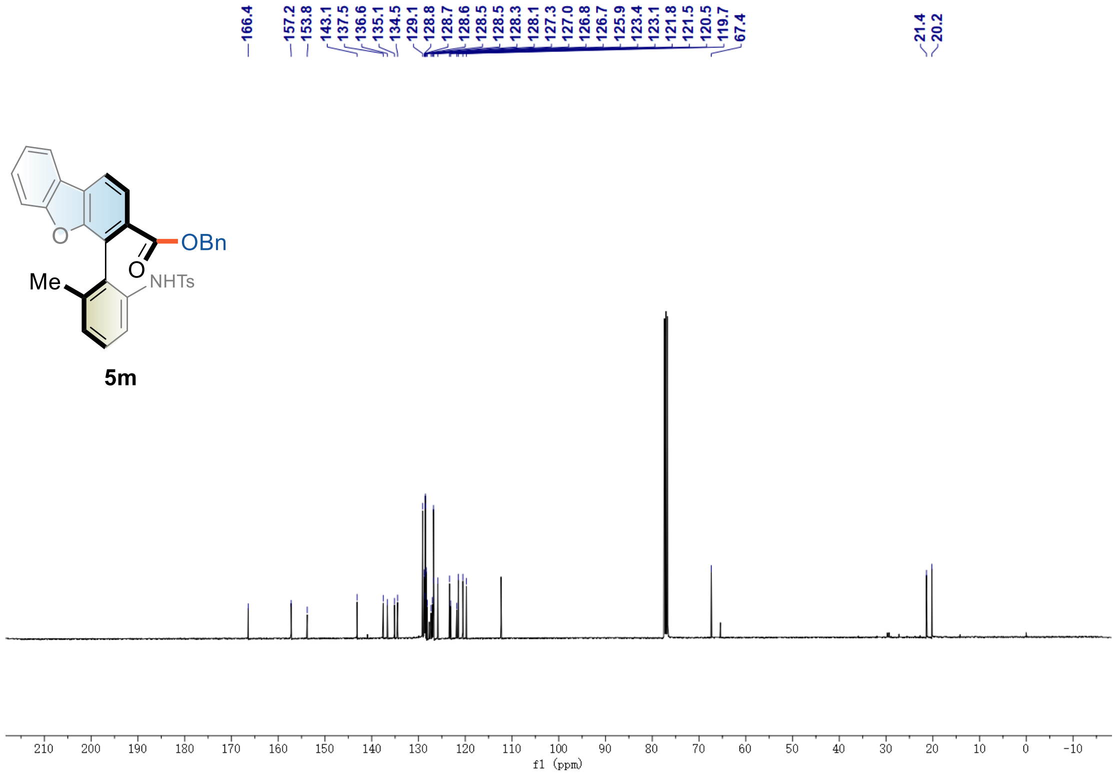

Supplementary Figure 95. **^{13}C NMR** of the **5m** (101 MHz, $CDCl_3$)

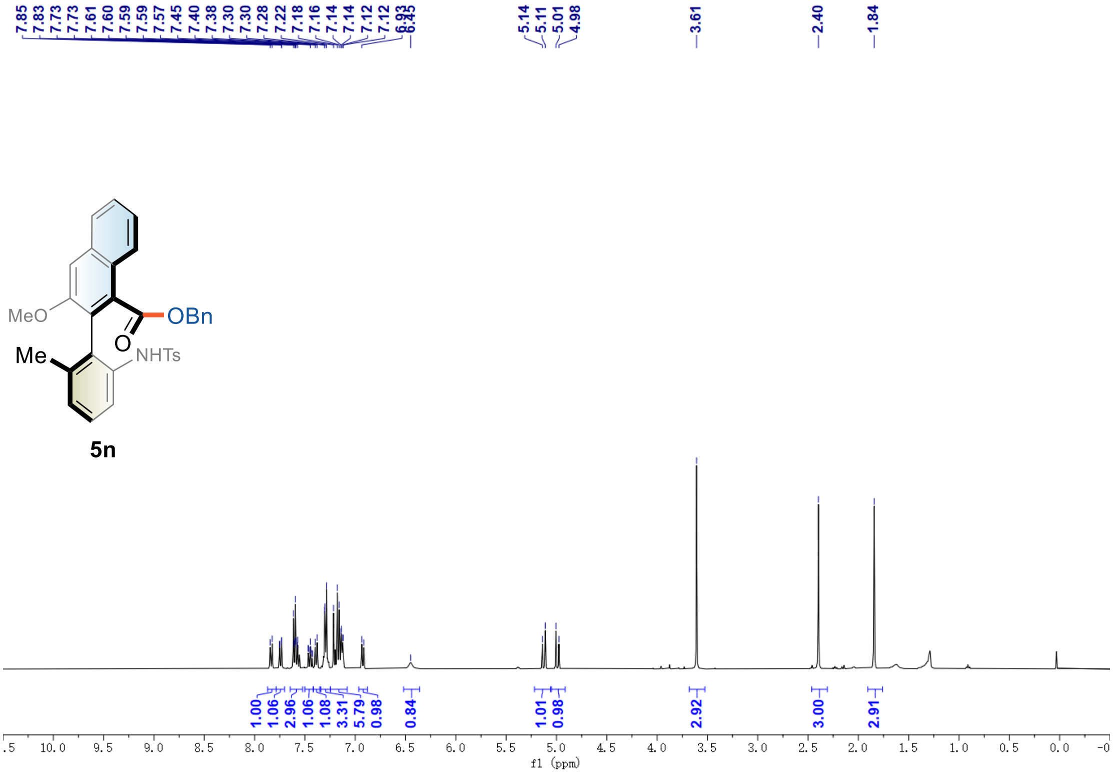

Supplementary Figure 96. ^{1}**H NMR** of the **5n** (400 MHz, $CDCl_3$)

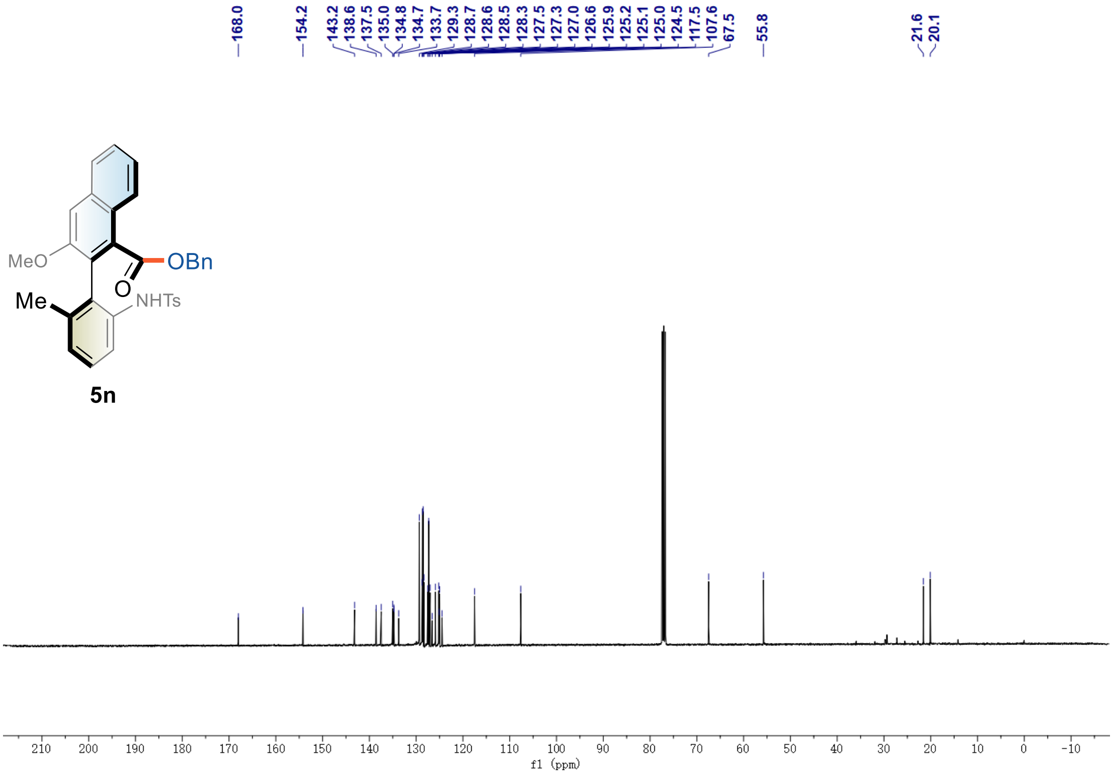

Supplementary Figure 97. ^{13}**C NMR** of the **5n** (101 MHz, $CDCl_3$)

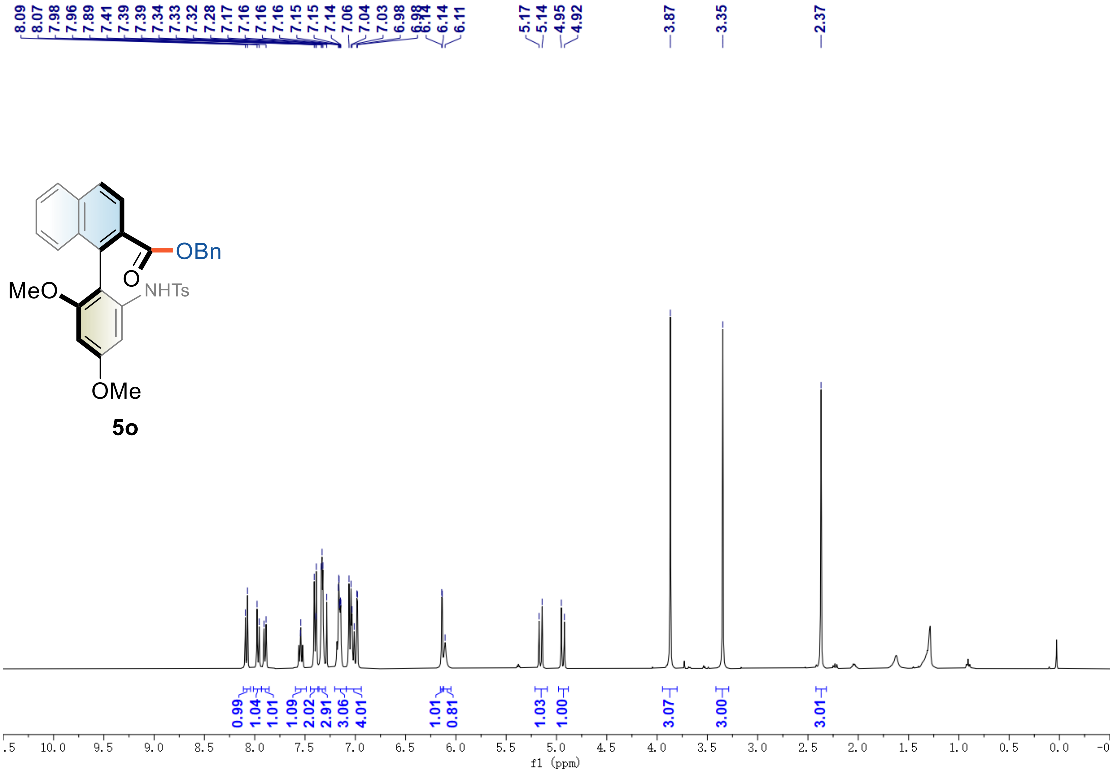

Supplementary Figure 98. ^{1}**H NMR** of the **5o** (400 MHz, $CDCl_3$)

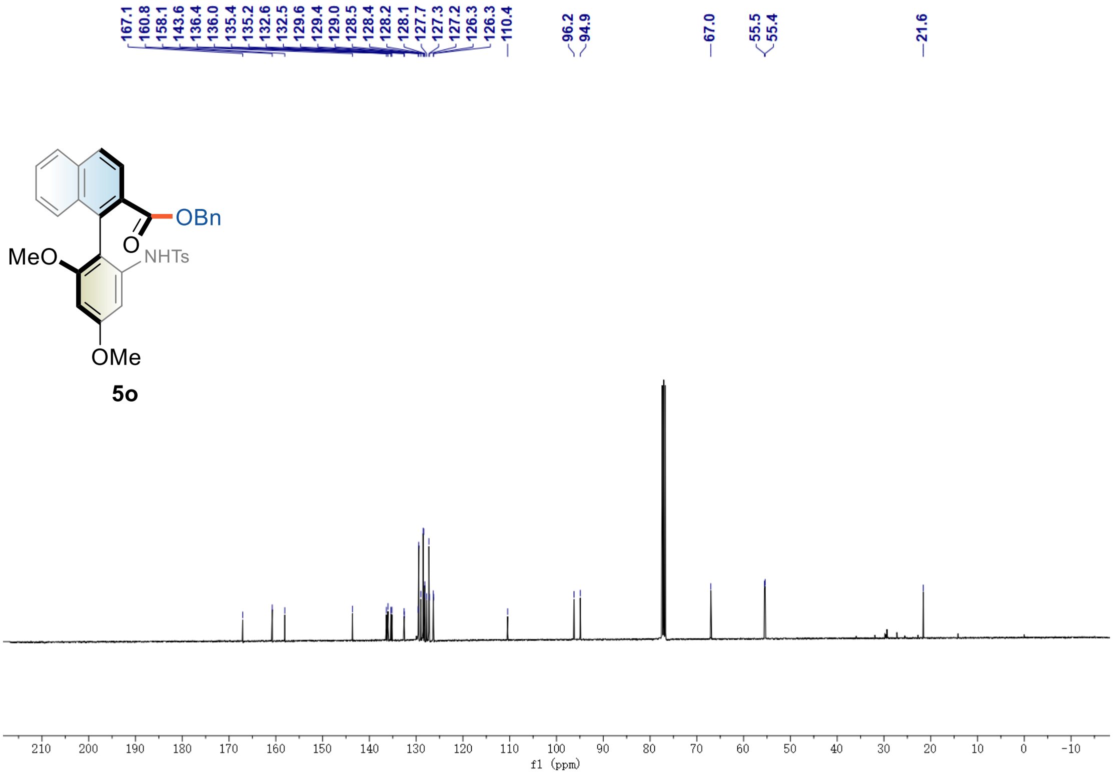

Supplementary Figure 99. ^{13}**C NMR** of the **5o** (101 MHz, $CDCl_3$)

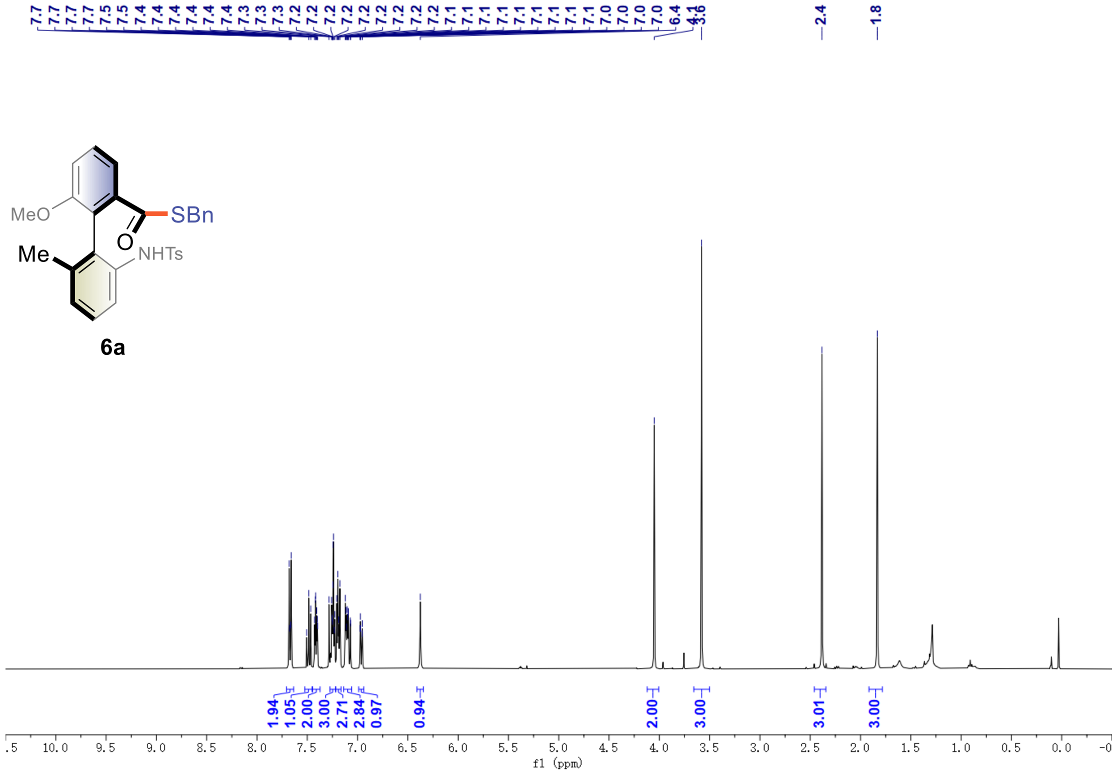

Supplementary Figure 100. ^{1}**H NMR** of the **6a** (400 MHz, $CDCl_3$)

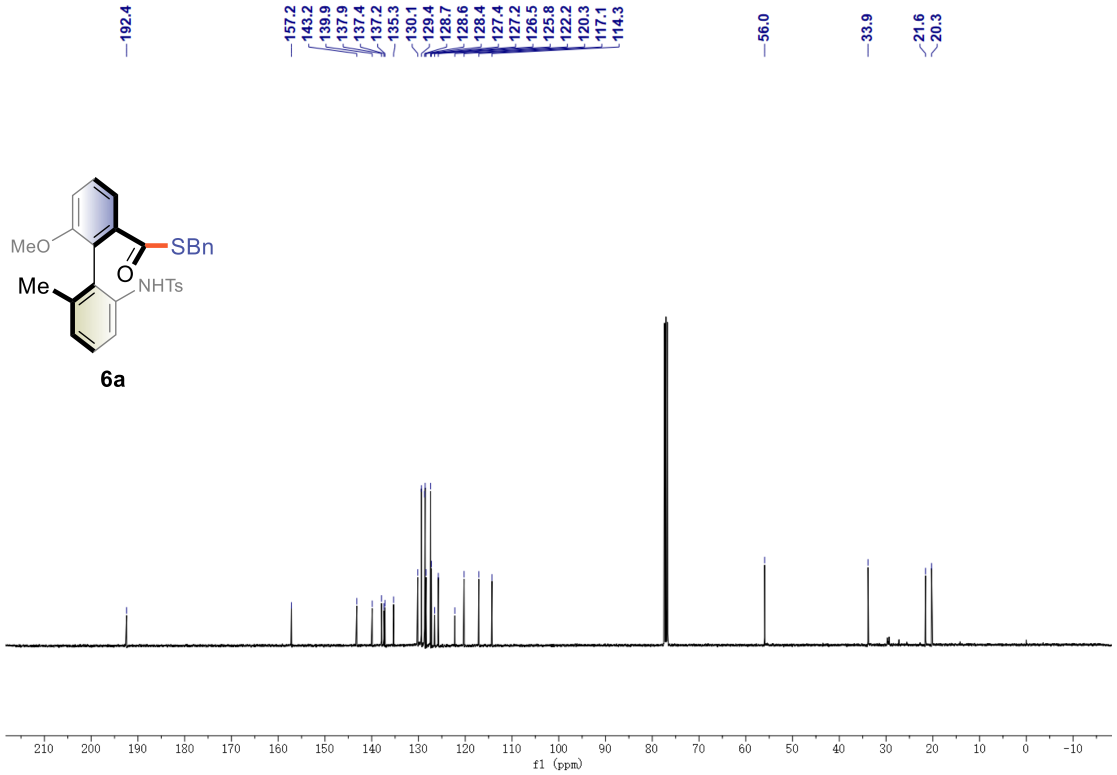

Supplementary Figure 101. ^{13}**C NMR** of the **6a** (101 MHz, $CDCl_3$)

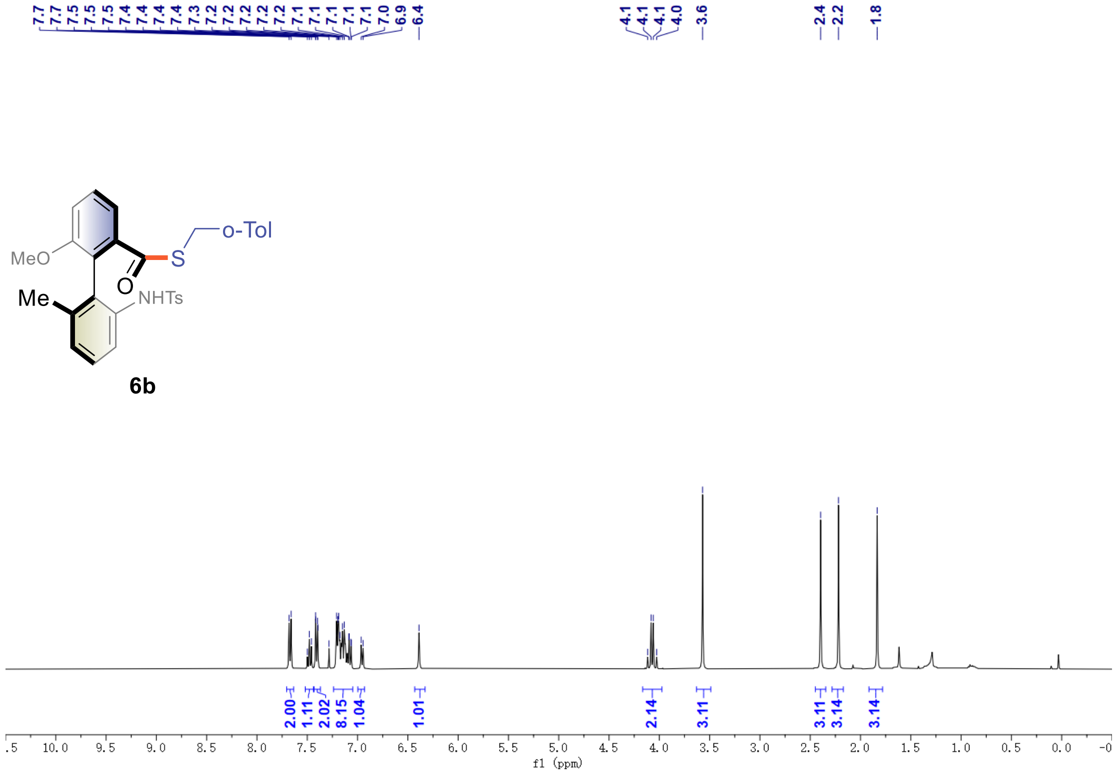

Supplementary Figure 102. **^{1}H NMR** of the **6b** (400 MHz, $CDCl_3$)

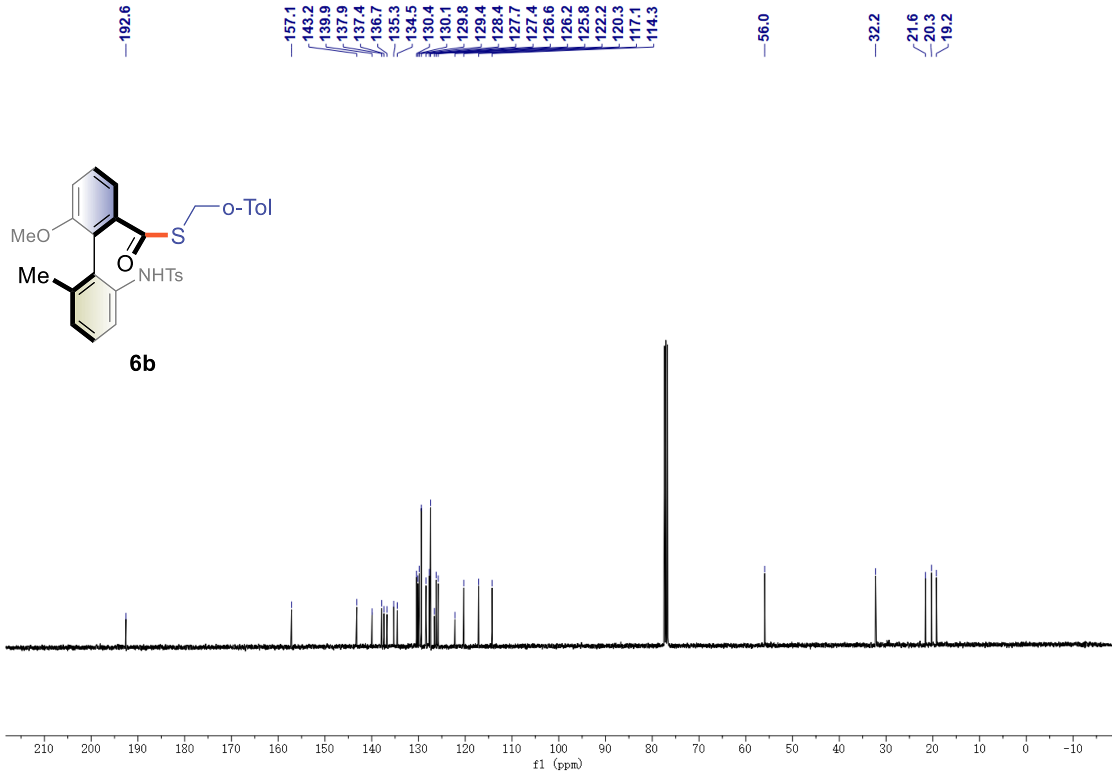

Supplementary Figure 103. **^{13}C NMR** of the **6b** (101 MHz, $CDCl_3$)

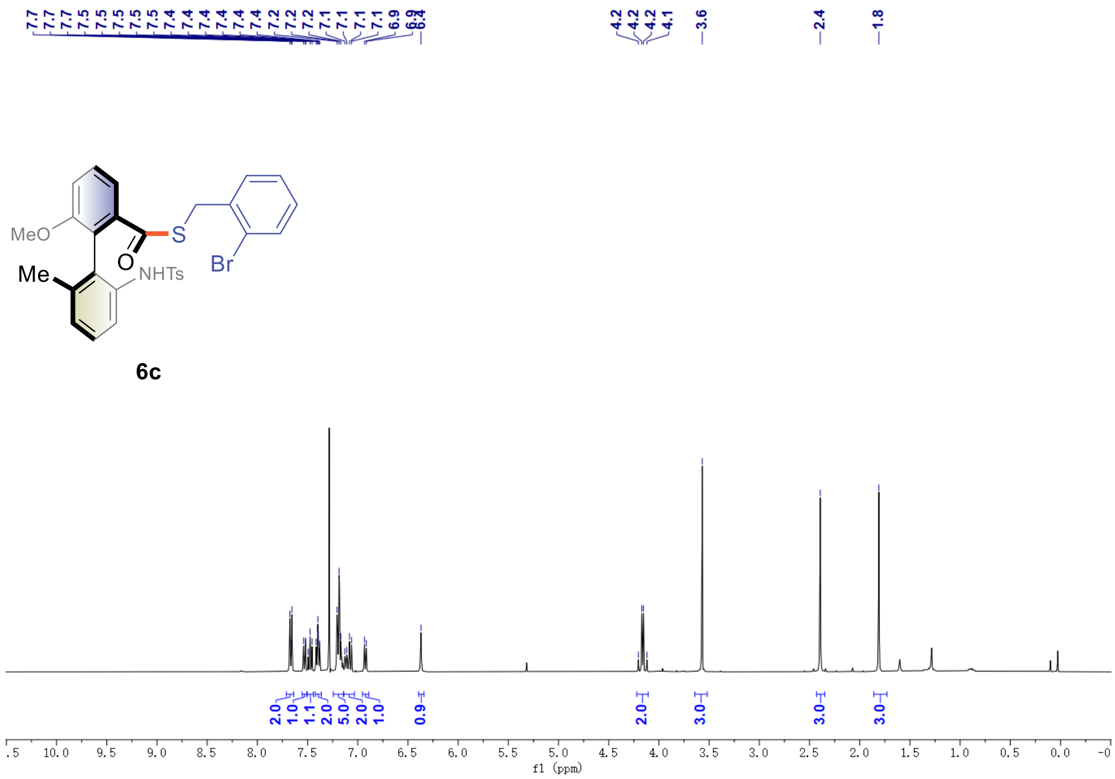

Supplementary Figure 104. ^{1}H NMR of the **6c** (400 MHz, $CDCl_3$)

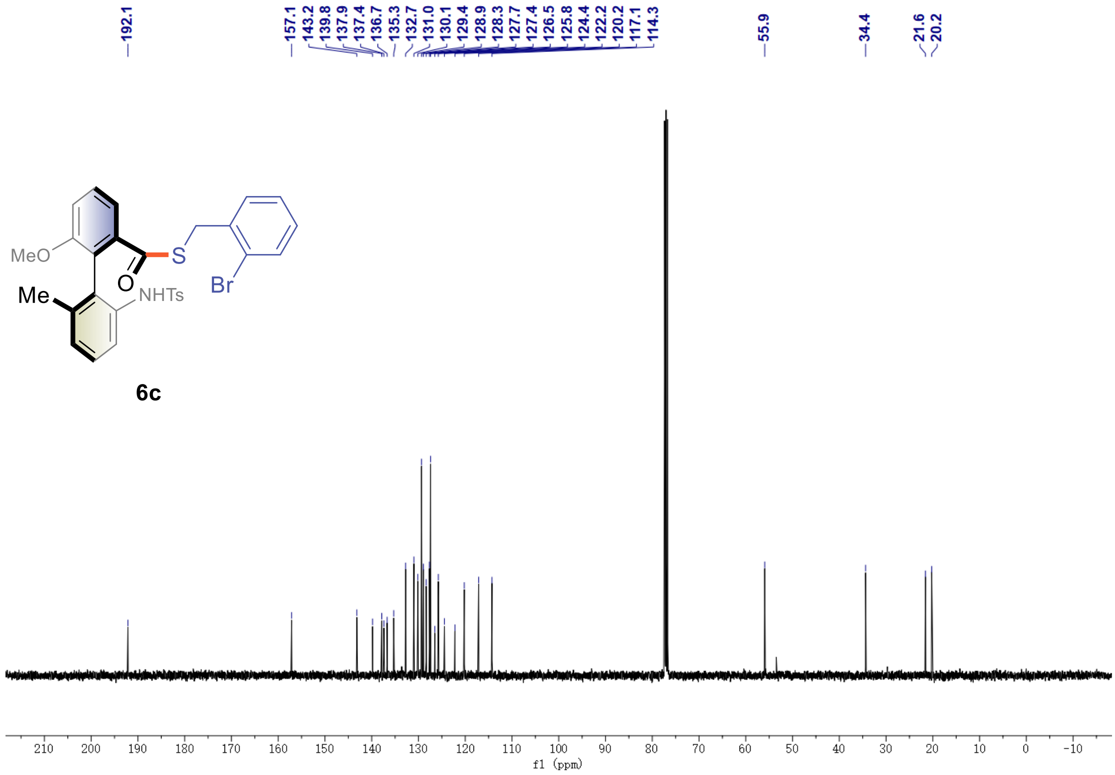

Supplementary Figure 105. ^{13}C NMR of the **6c** (101 MHz, $CDCl_3$)

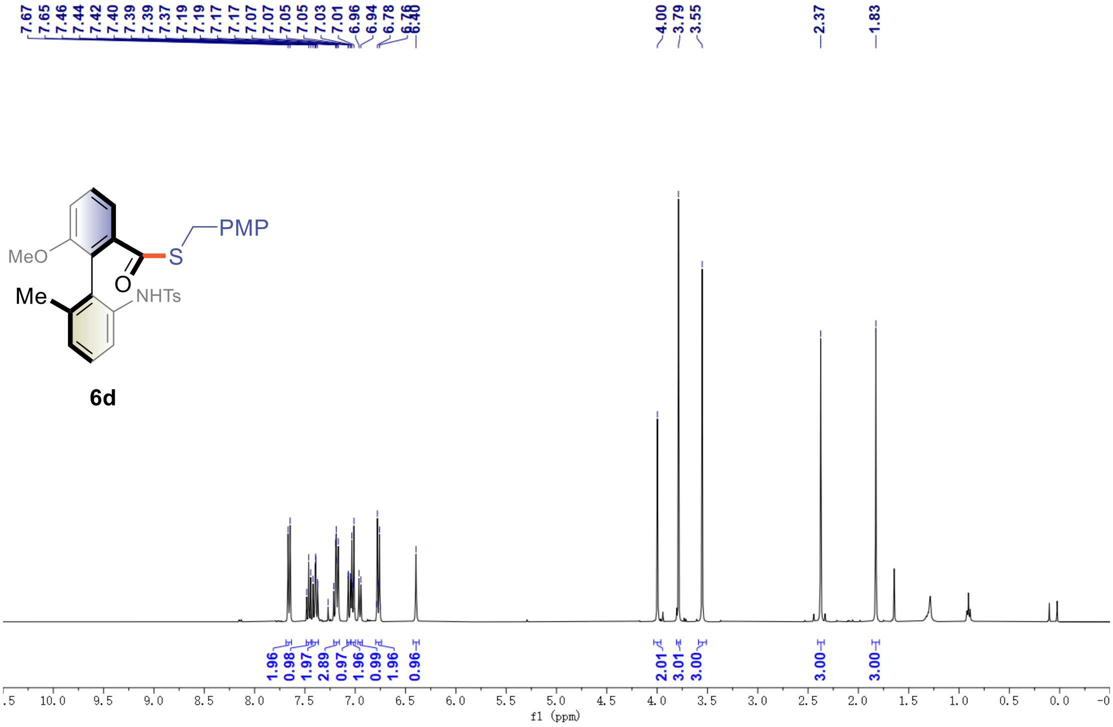

Supplementary Figure 106. **^{1}H NMR** of the **6d** (400 MHz, $CDCl_3$)

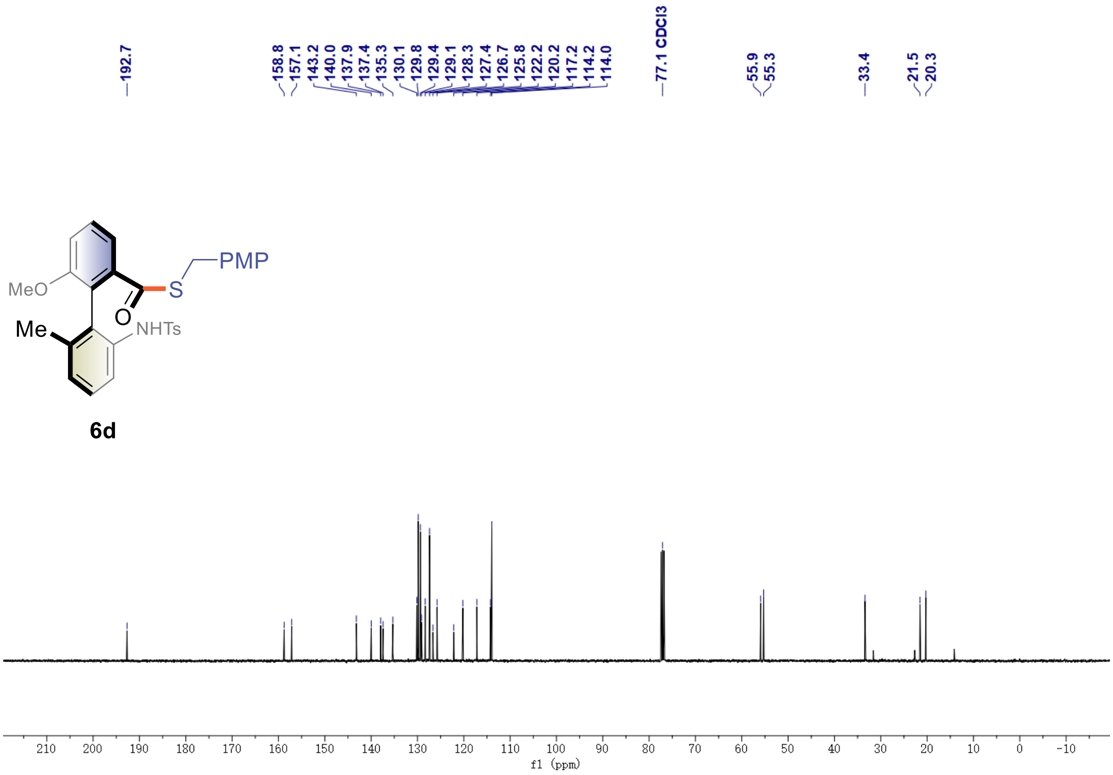

Supplementary Figure 107. **^{13}C NMR** of the **6d** (101 MHz, $CDCl_3$)

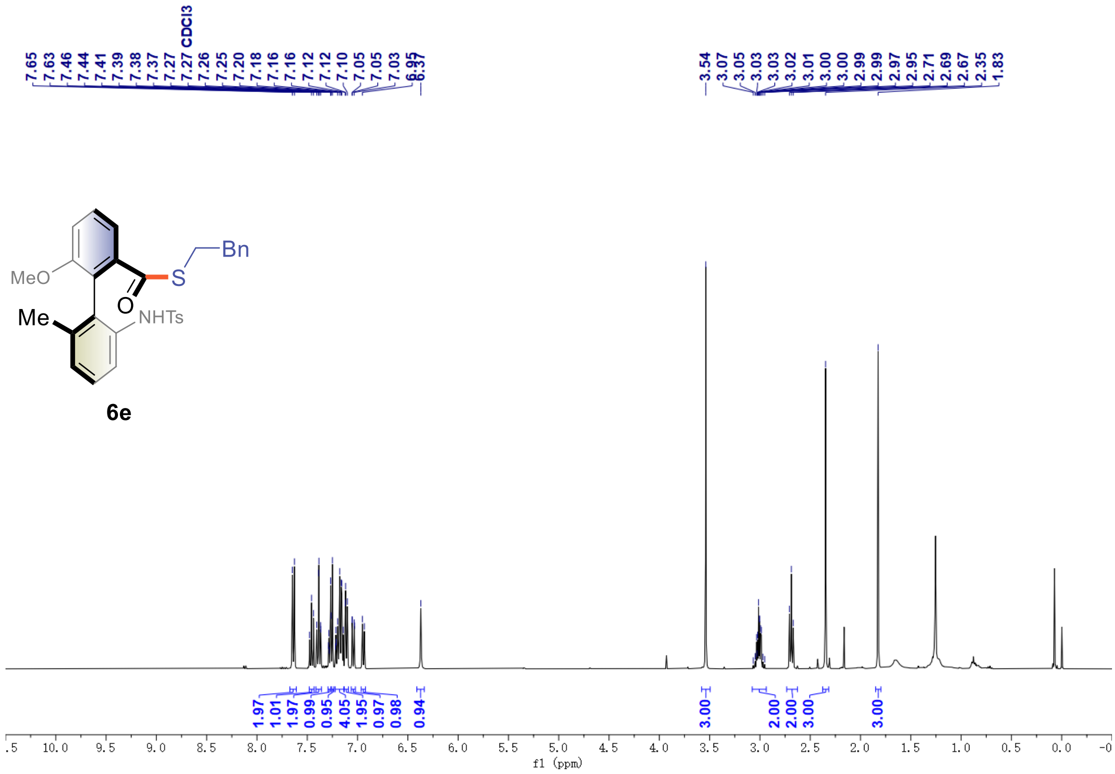

Supplementary Figure 108. ^{1}**H NMR** of the **6e** (400 MHz, $CDCl_3$)

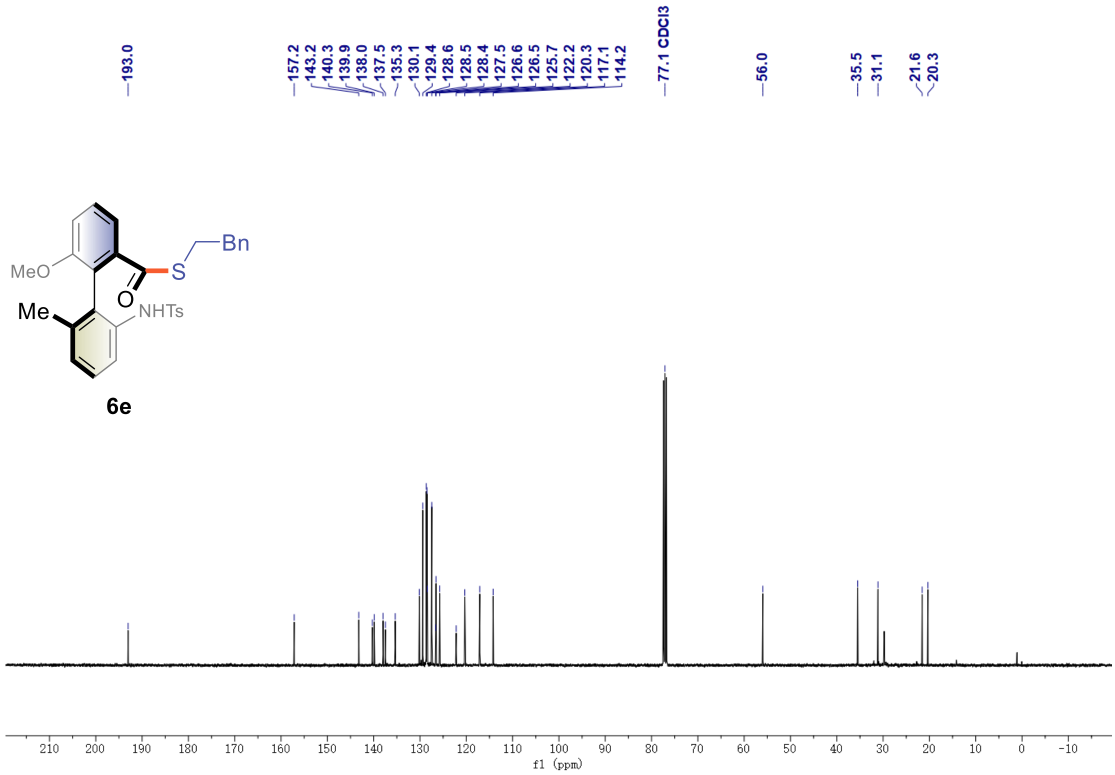

Supplementary Figure 109. ^{13}**C NMR** of the **6e** (101 MHz, $CDCl_3$)

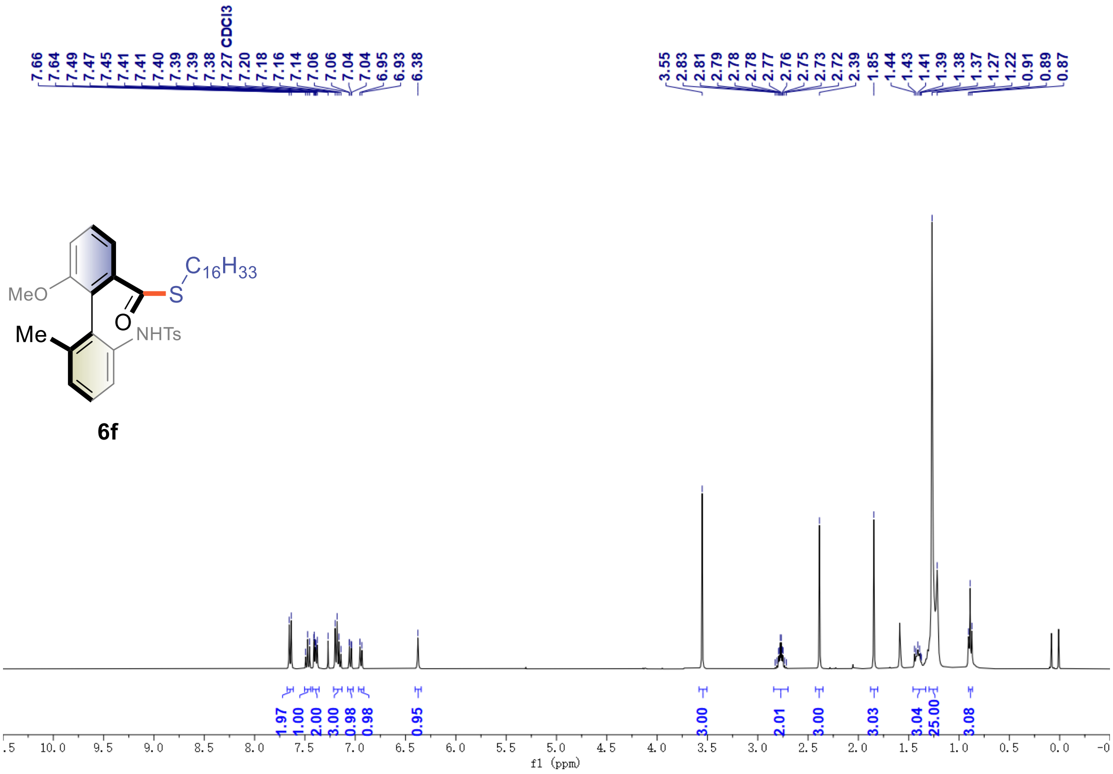

Supplementary Figure 110. **^{1}H NMR** of the **6f** (400 MHz, $CDCl_3$)

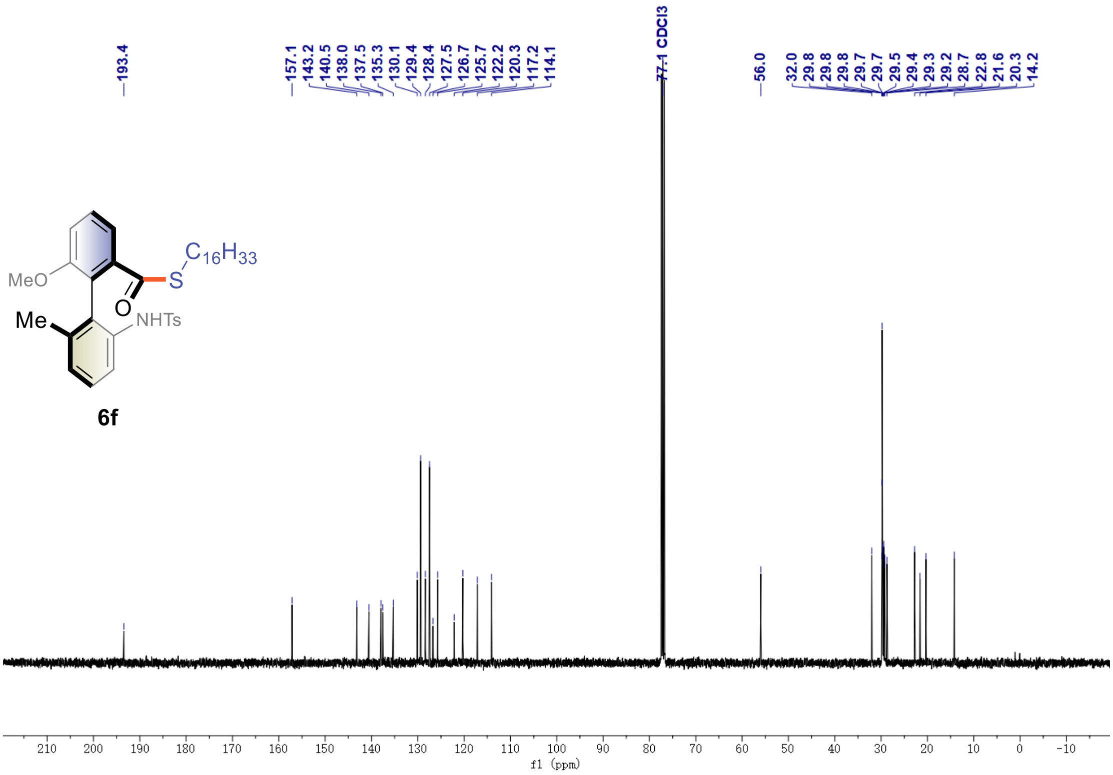

Supplementary Figure 111. **^{13}C NMR** of the **6f** (101 MHz, $CDCl_3$)

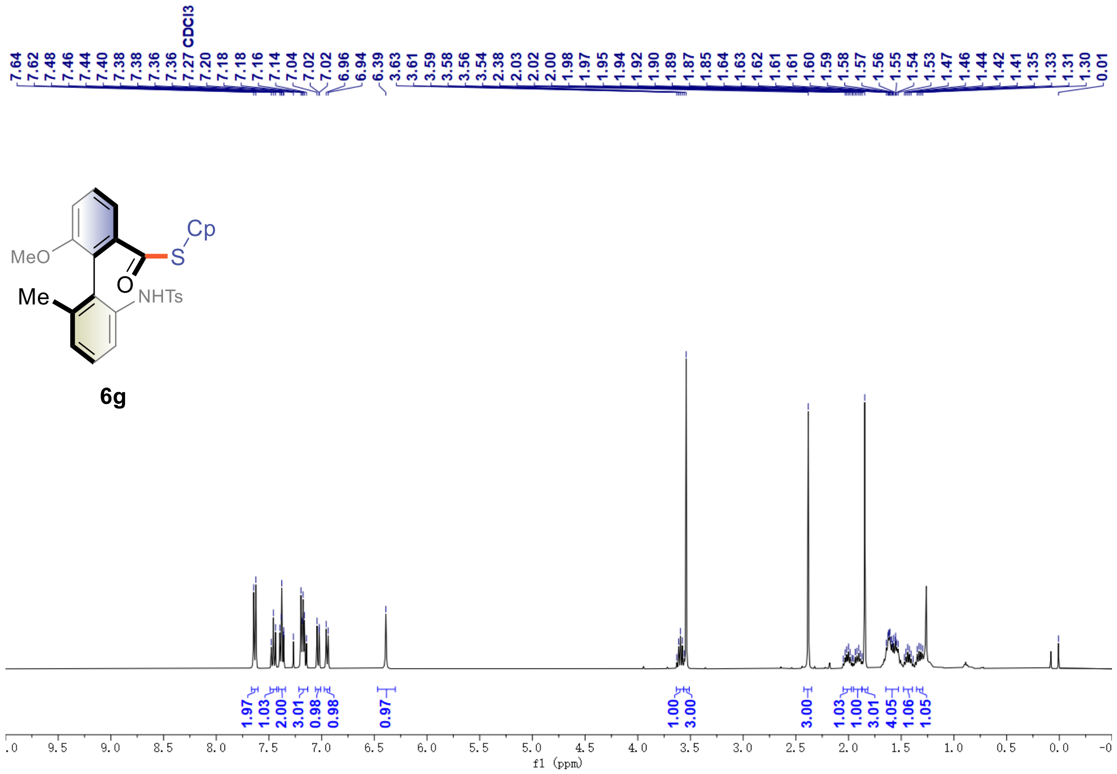

Supplementary Figure 112. ^{1}**H NMR** of the **6g** (400 MHz, $CDCl_3$)

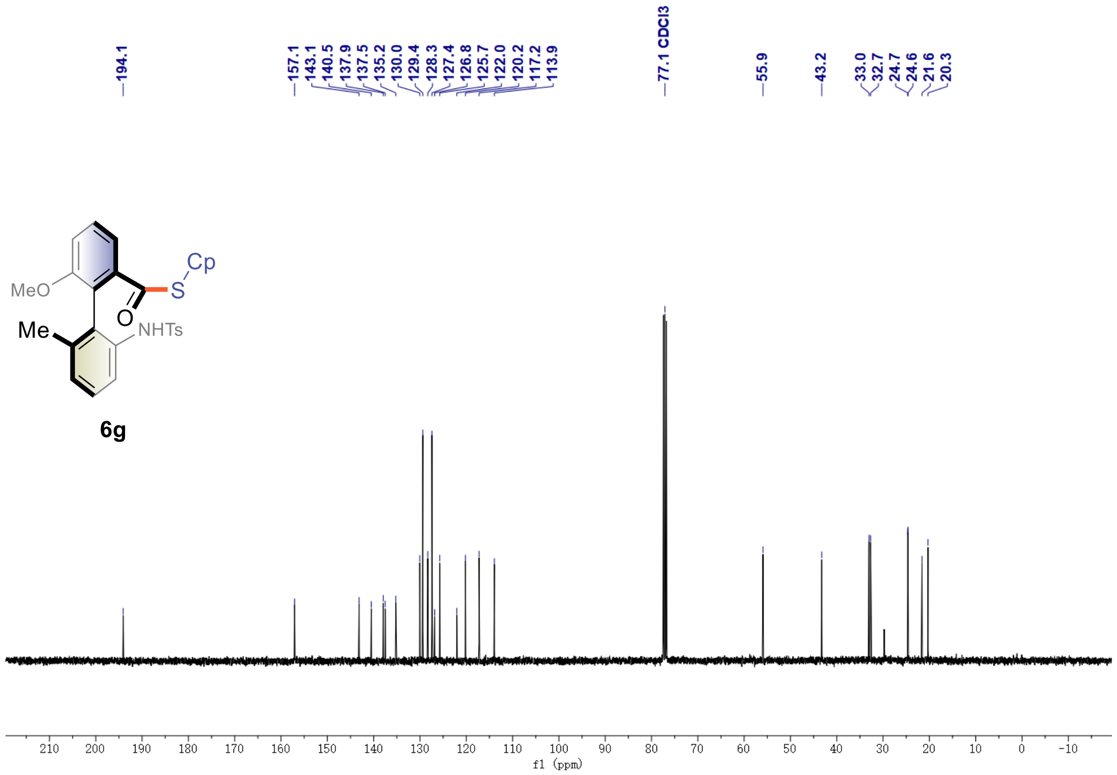

Supplementary Figure 113. ^{13}**C NMR** of the **6g** (101 MHz, $CDCl_3$)

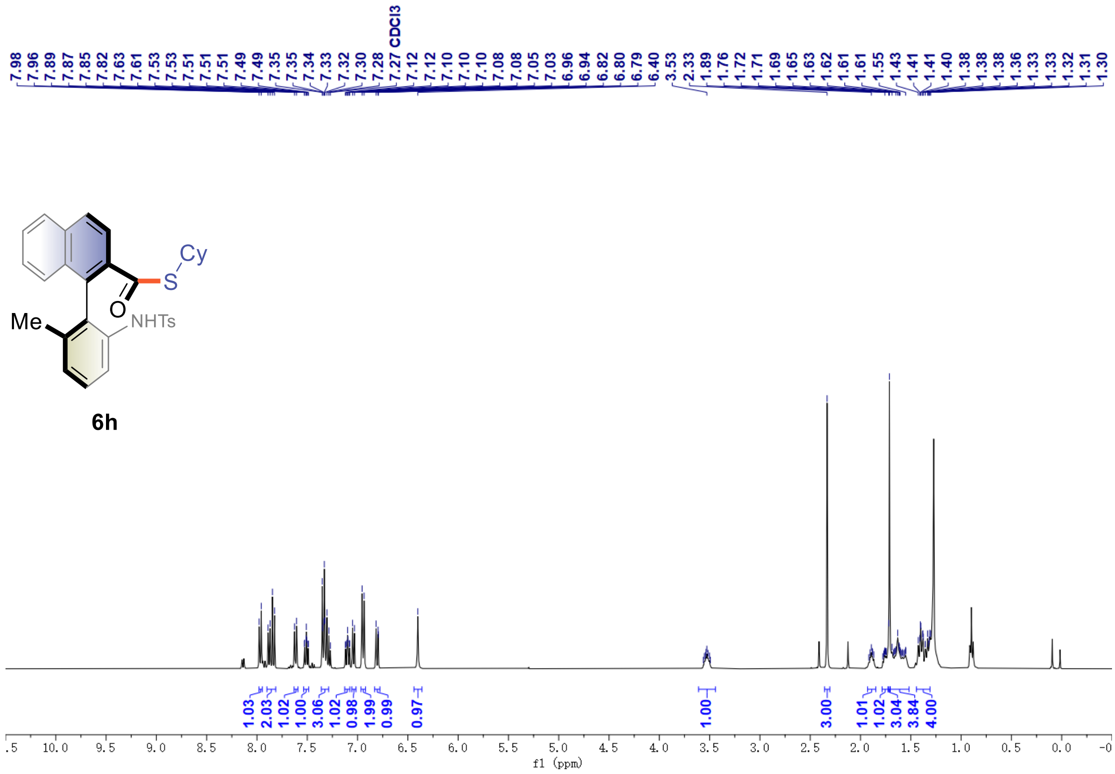

Supplementary Figure 114. ^{1}**H NMR** of the **6h** (400 MHz, $CDCl_3$)

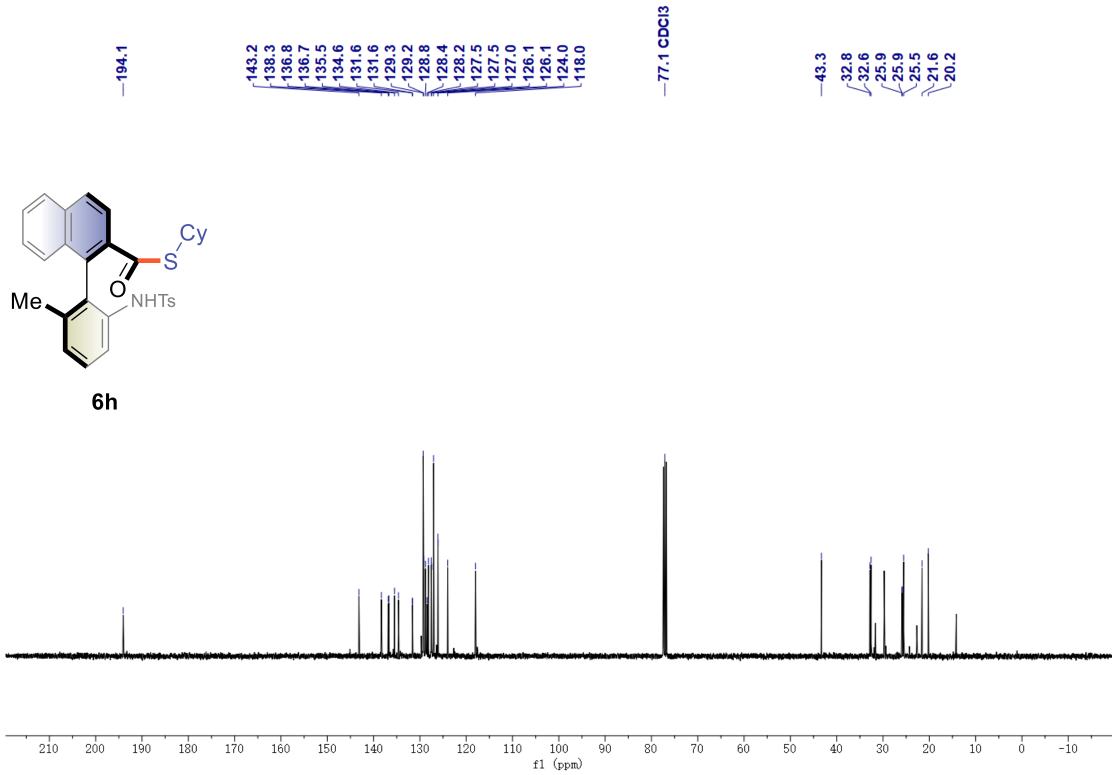

Supplementary Figure 115. ^{13}**C NMR** of the **6h** (101 MHz, $CDCl_3$)

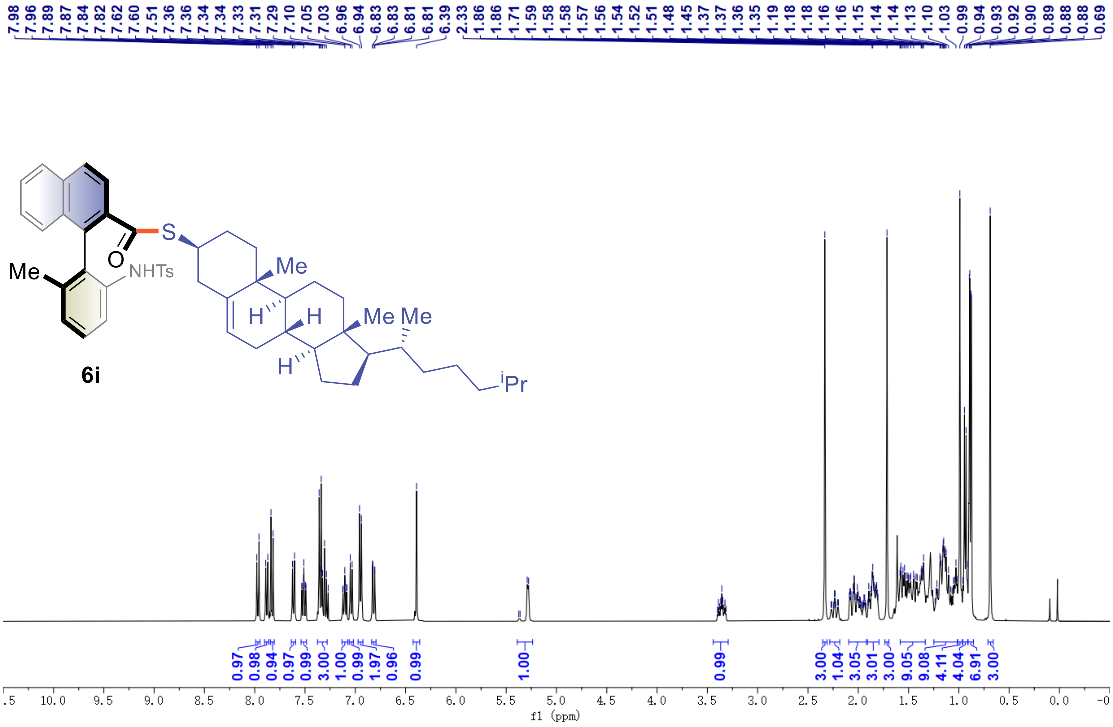

Supplementary Figure 116. **^{1}H NMR** of the **6i** (400 MHz, $CDCl_3$)

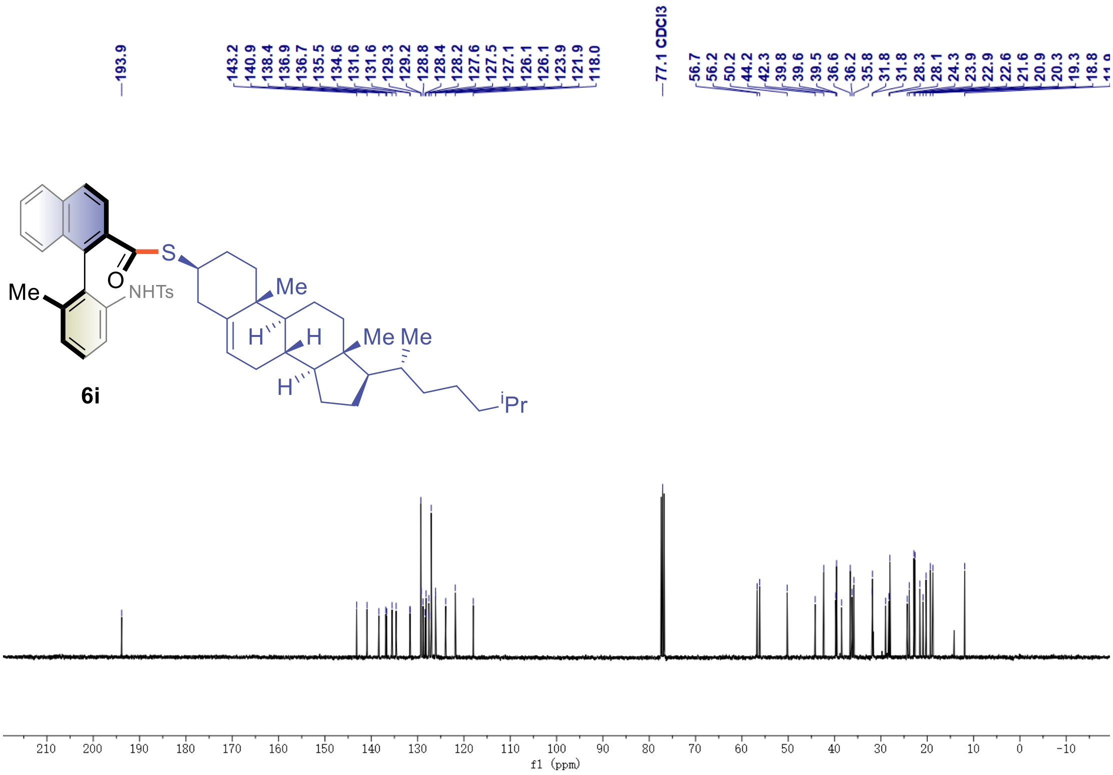

Supplementary Figure 117. **^{13}C NMR** of the **6i** (101 MHz, $CDCl_3$)

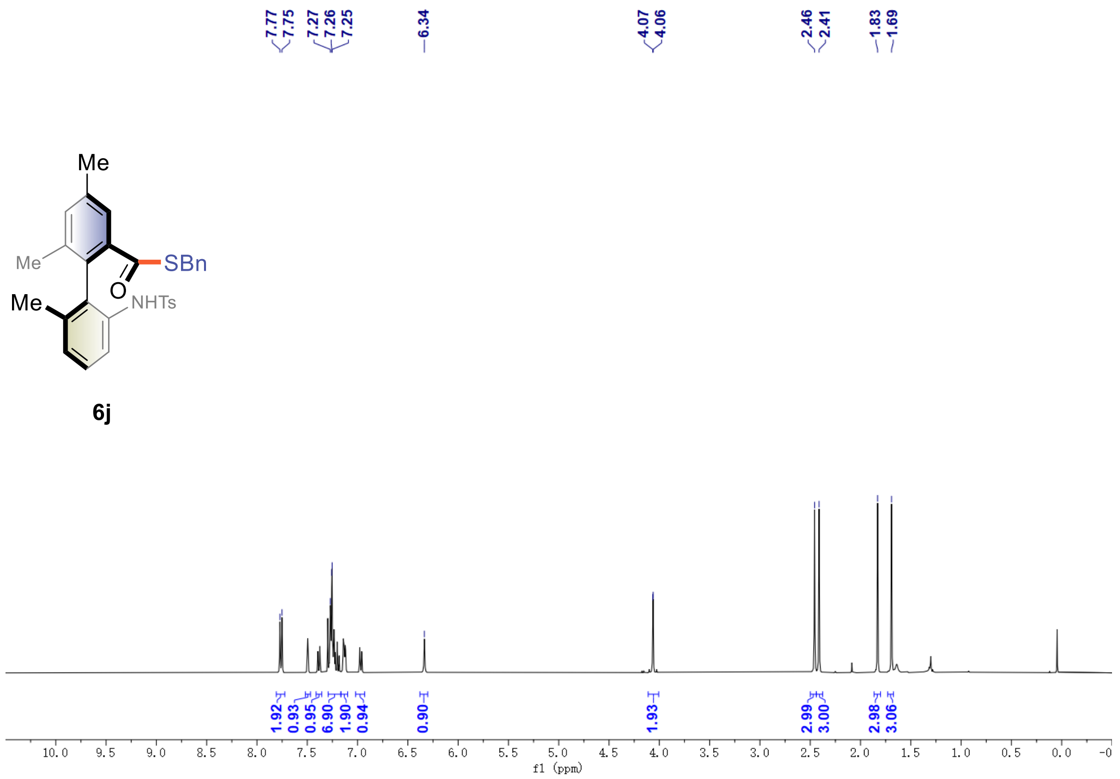

Supplementary Figure 118. ^{1}H NMR of the **6j** (400 MHz, $CDCl_3$)

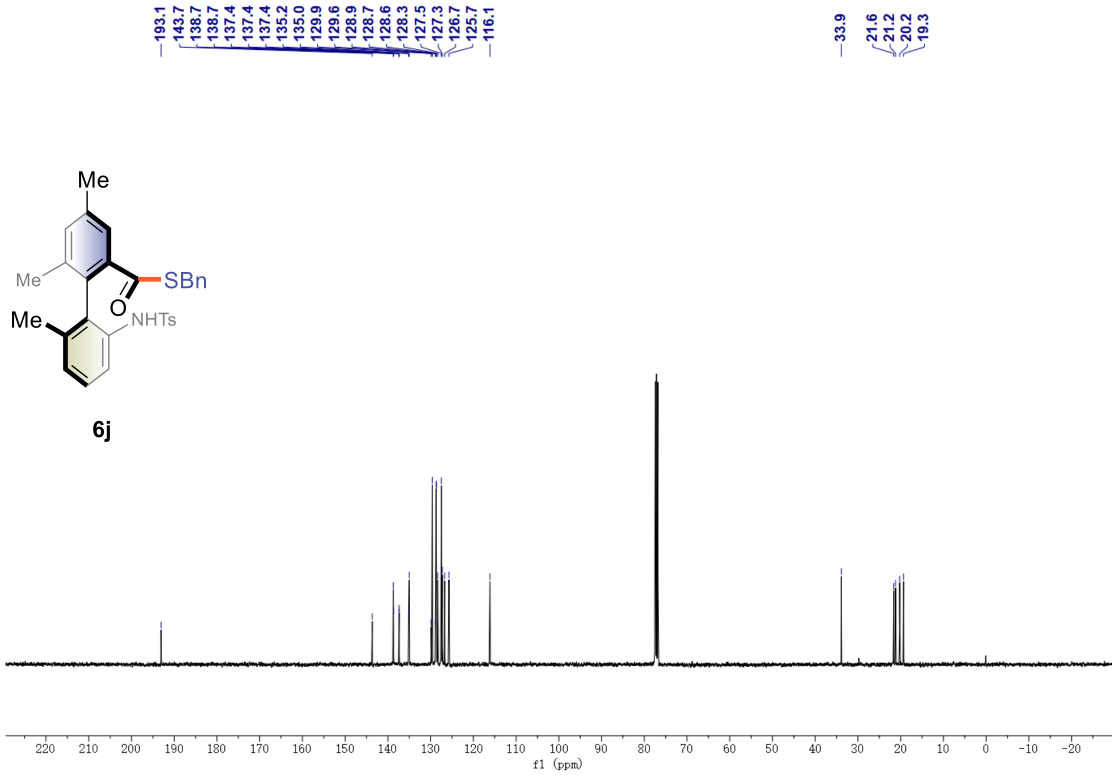

Supplementary Figure 119. ^{13}C NMR of the **6j** (101 MHz, $CDCl_3$)

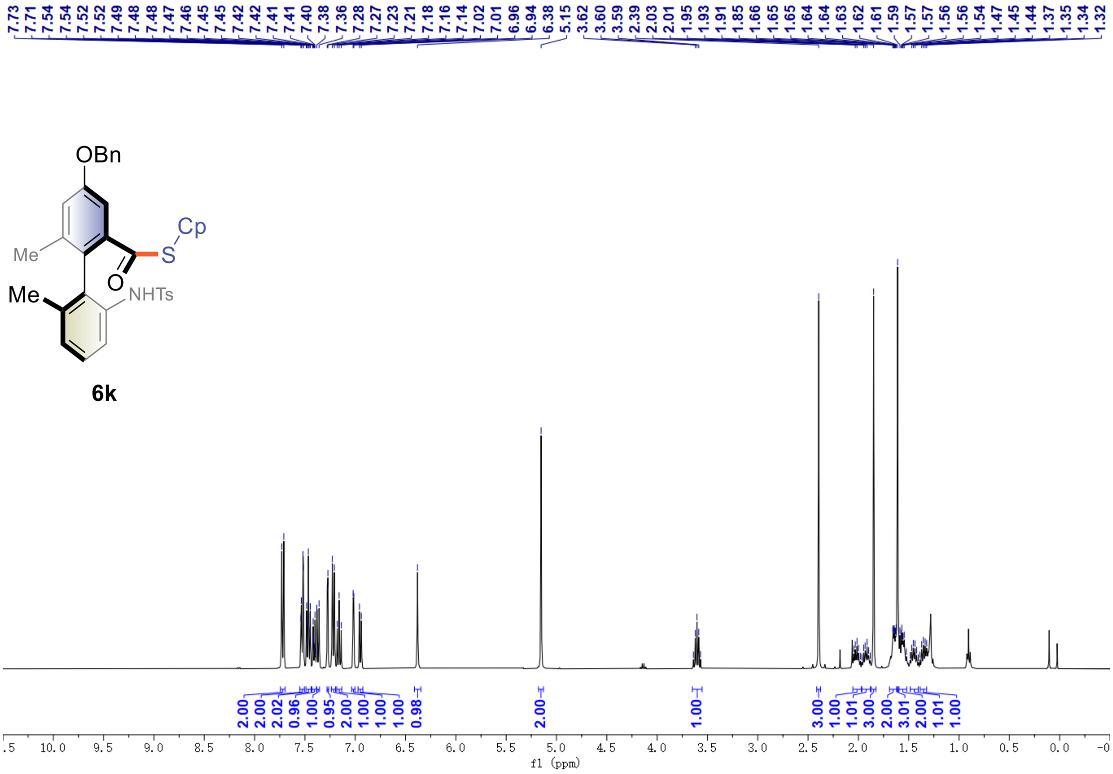

Supplementary Figure 120. ^{1}**H NMR** of the **6k** (400 MHz, $CDCl_3$)

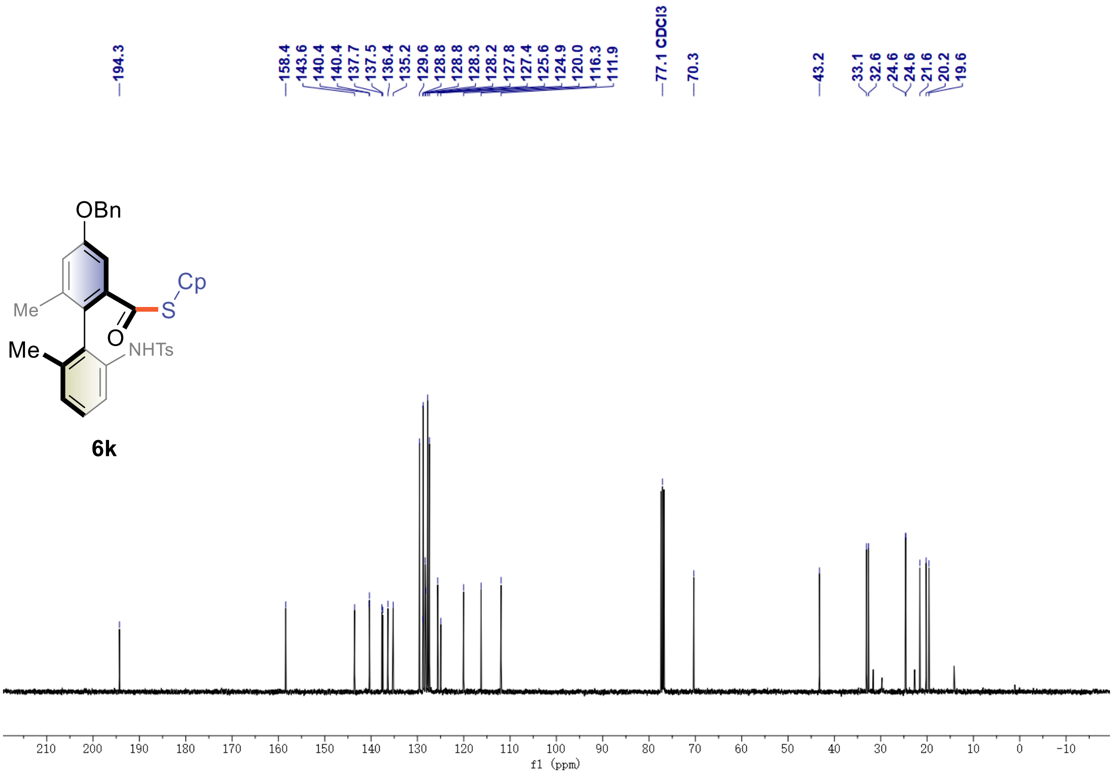

Supplementary Figure 121. ^{13}**C NMR** of the **6k** (101 MHz, $CDCl_3$)

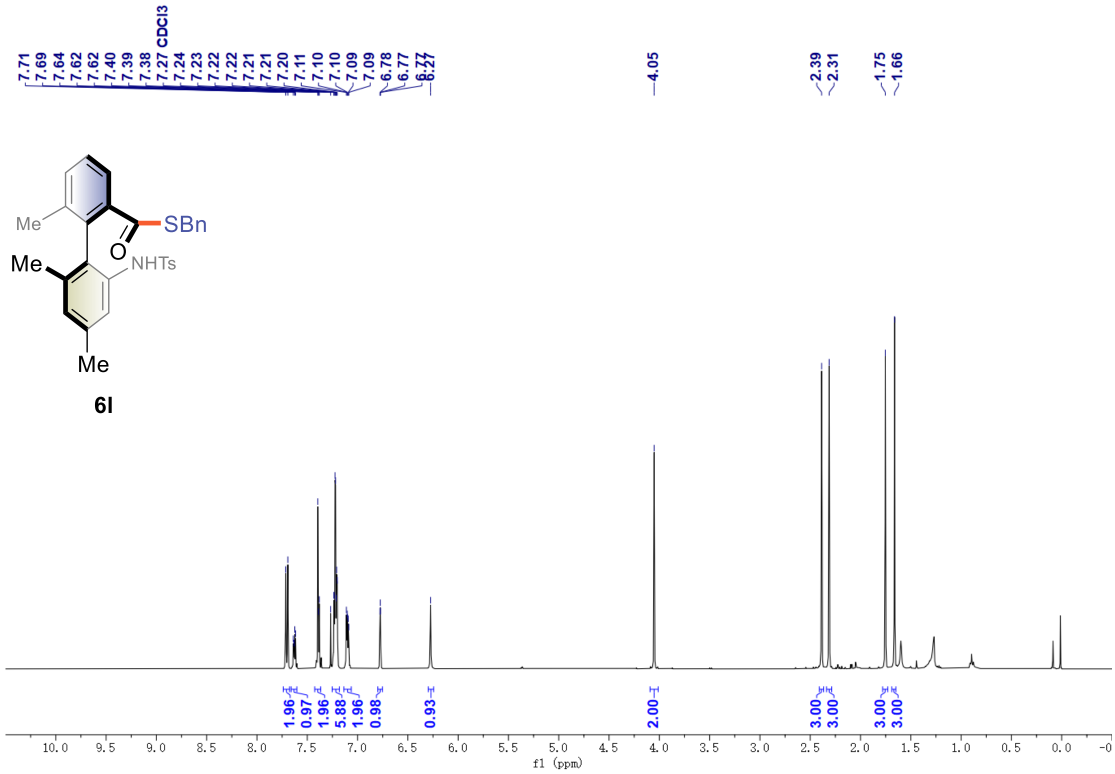

Supplementary Figure 122. ^{1}**H NMR** of the **6l** (400 MHz, $CDCl_3$)

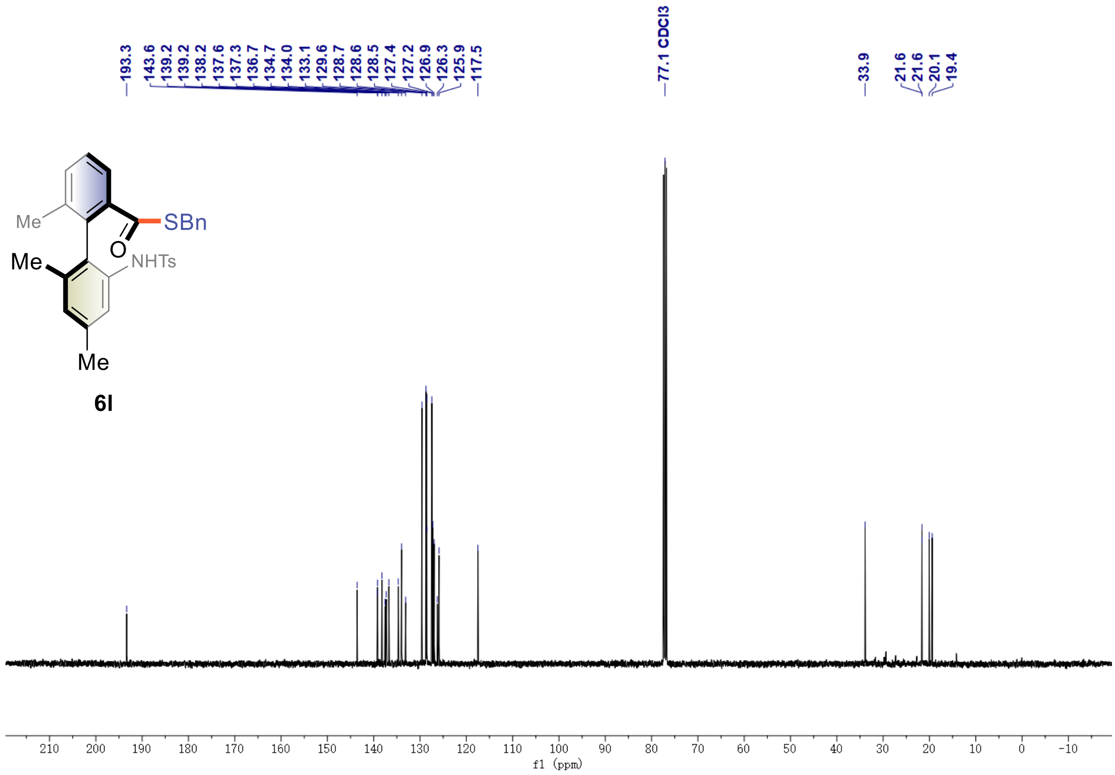

Supplementary Figure 123. ^{13}**C NMR** of the **6l** (101 MHz, $CDCl_3$)

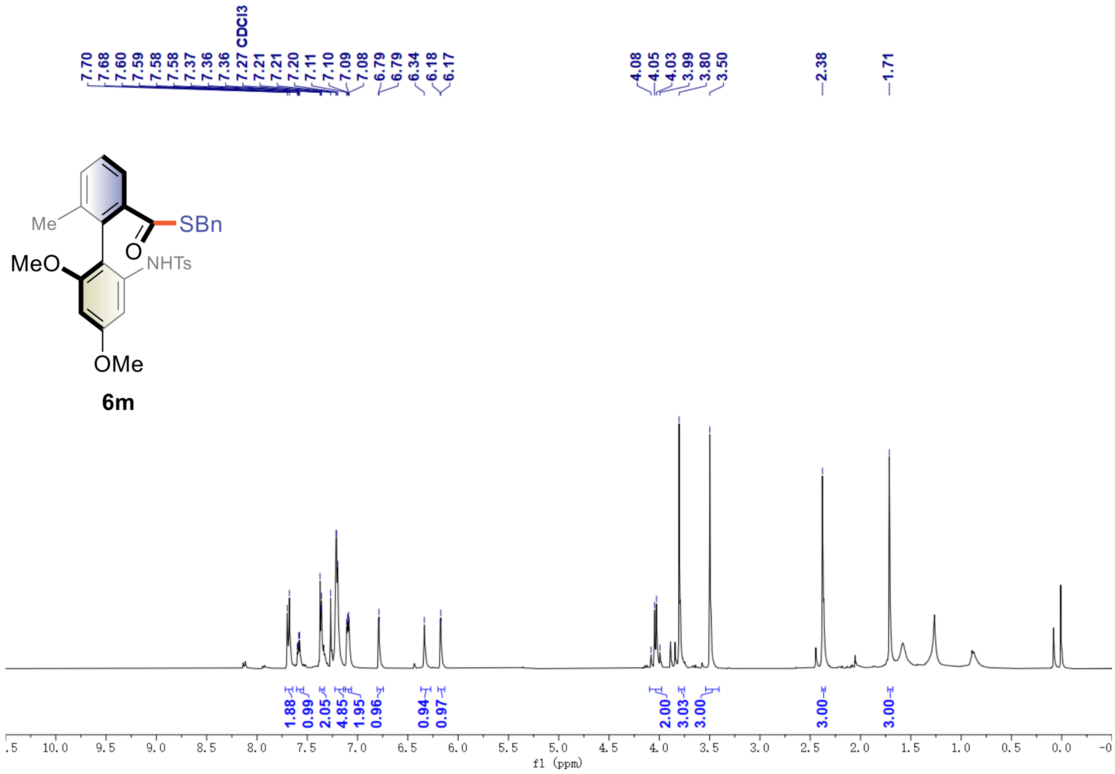

Supplementary Figure 124. ^{1}H NMR of the **6m** (400 MHz, $CDCl_3$)

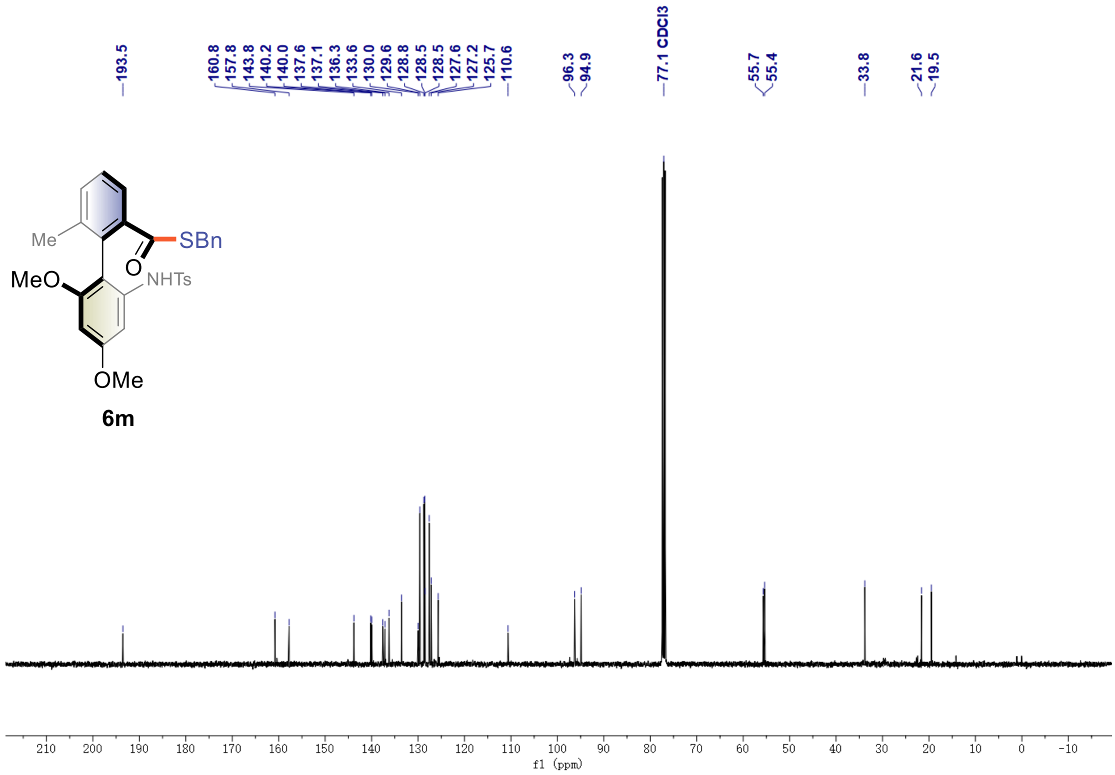

Supplementary Figure 125. ^{13}C NMR of the **6m** (101 MHz, $CDCl_3$)

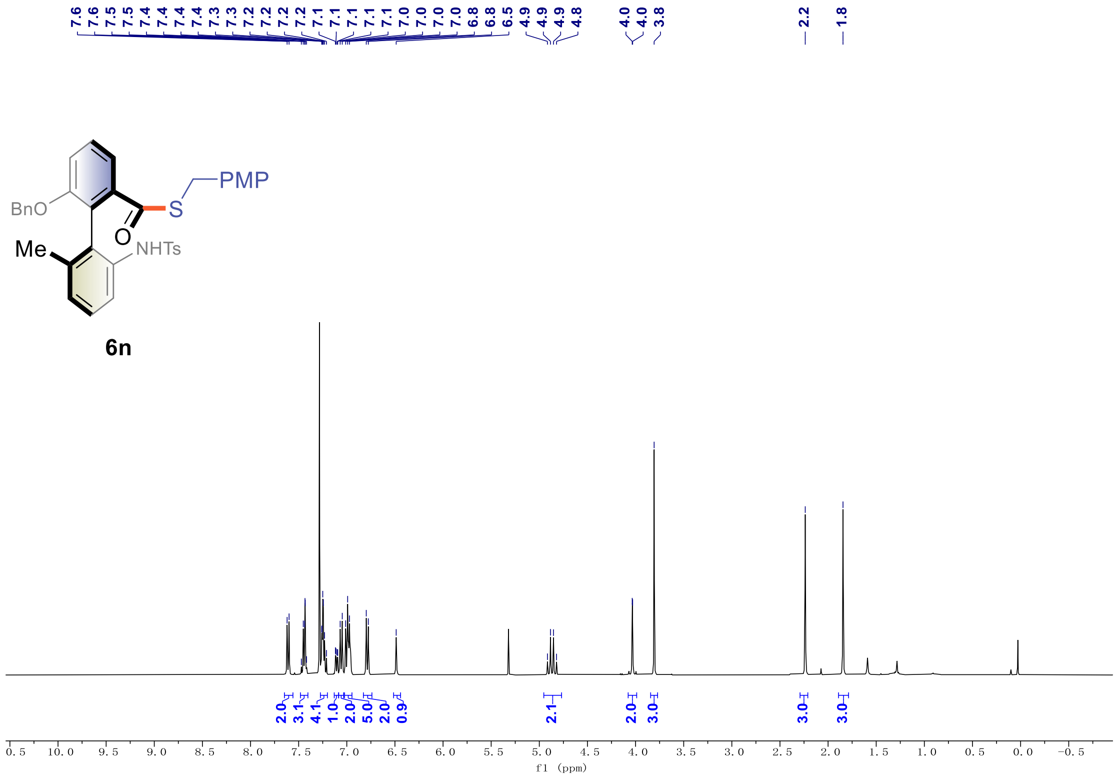

Supplementary Figure 126. ^{1}H NMR of the **6n** (400 MHz, $CDCl_3$)

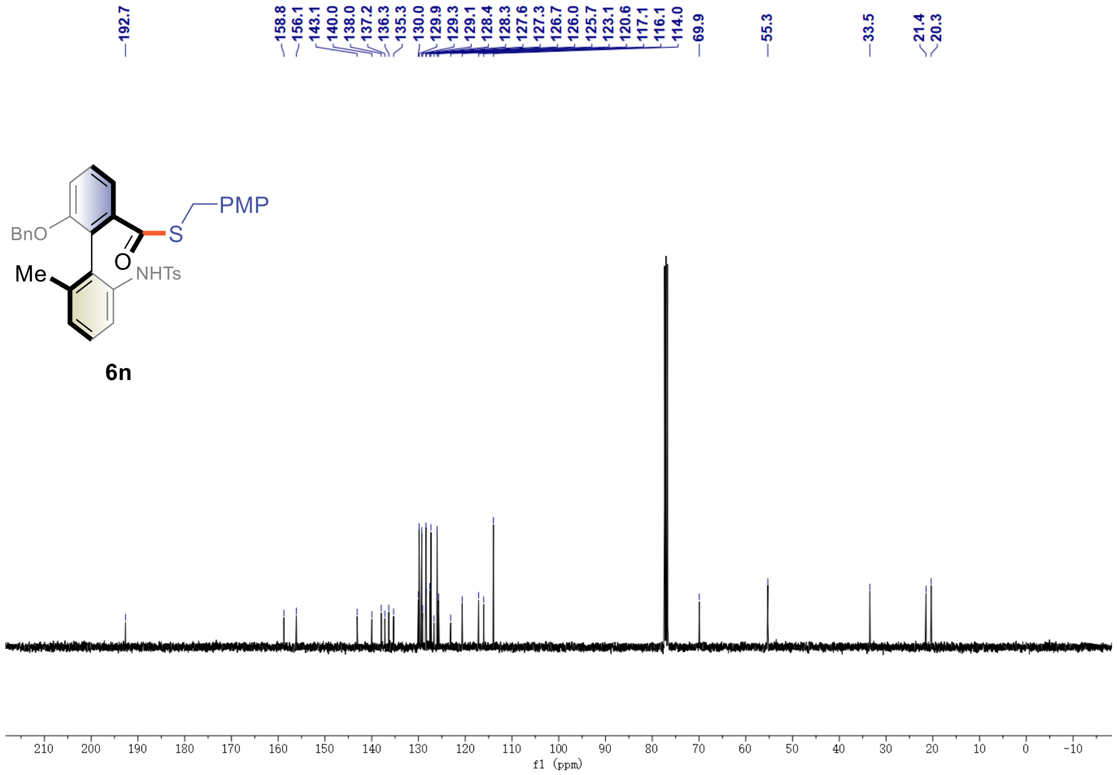

Supplementary Figure 127. ^{13}C NMR of the **6n** (101 MHz, $CDCl_3$)

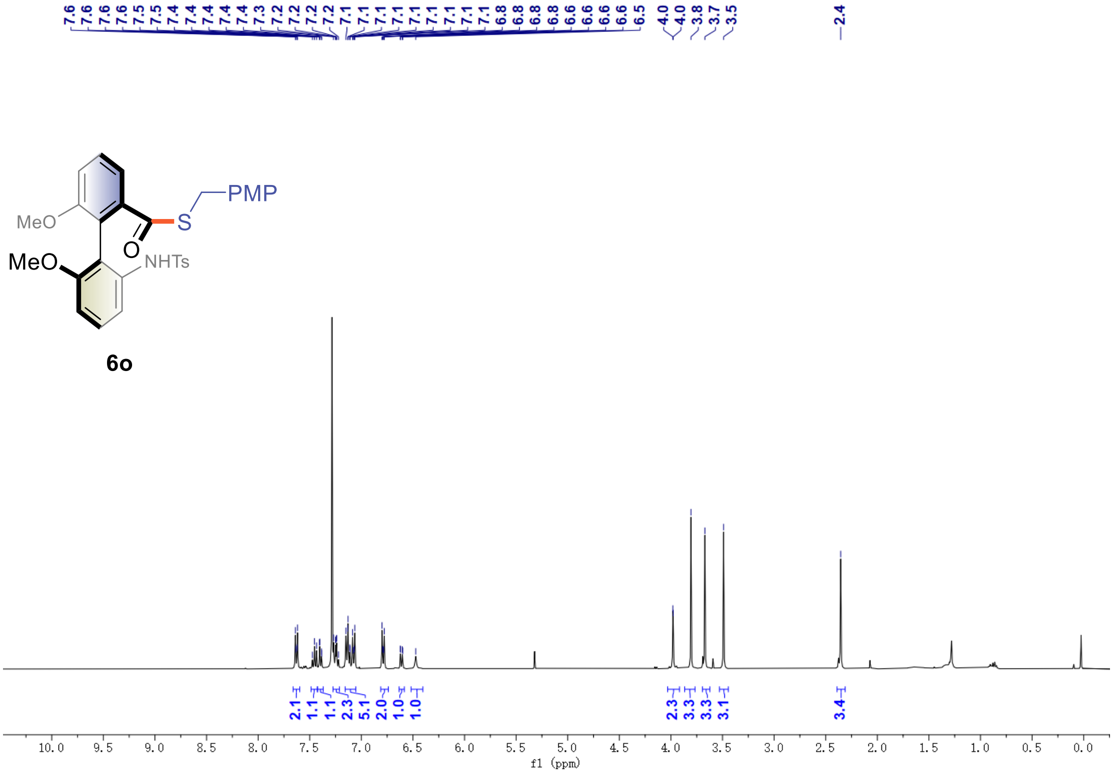

Supplementary Figure 128. **^{1}H NMR** of the **6o** (400 MHz, $CDCl_3$)

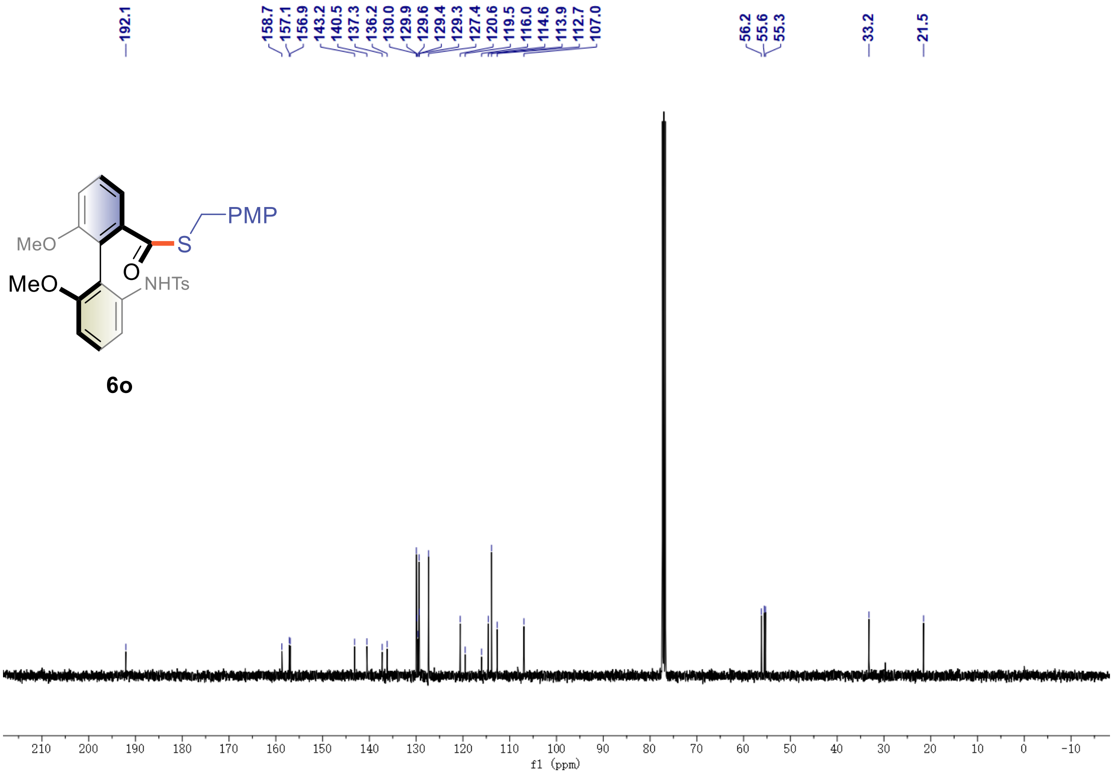

Supplementary Figure 129. **^{13}C NMR** of the **6o** (101 MHz, $CDCl_3$)

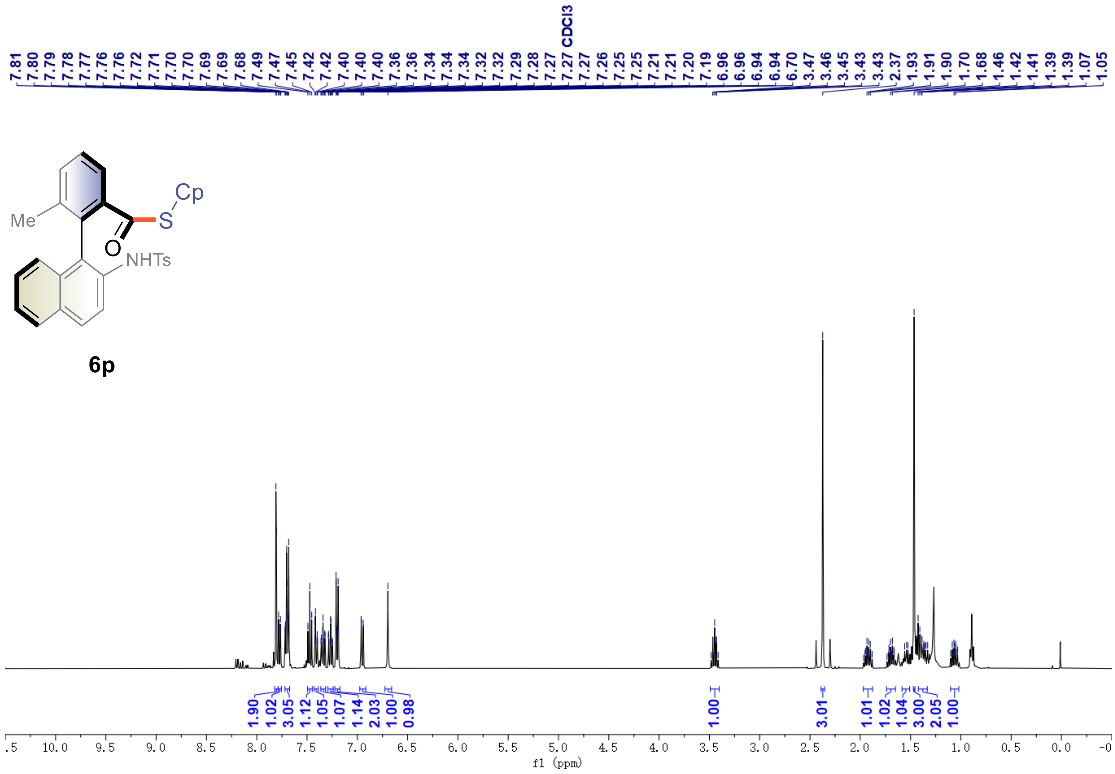

Supplementary Figure 130. **^{1}H NMR** of the **6p** (400 MHz, $CDCl_3$)

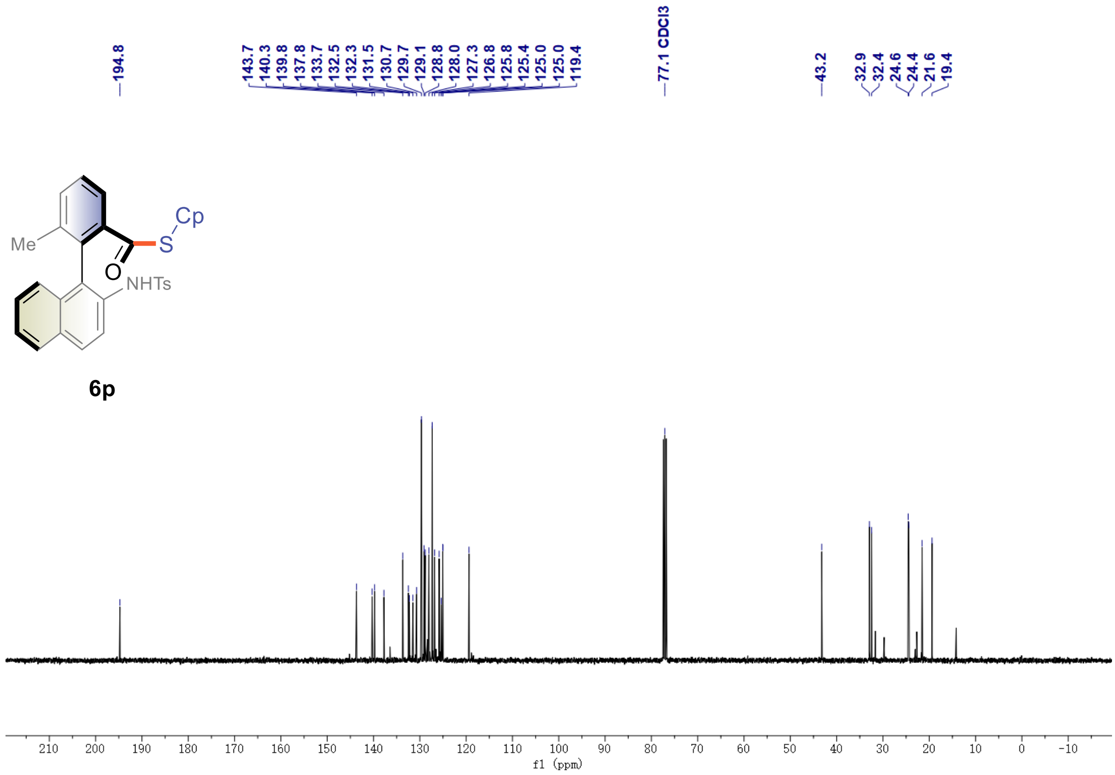

Supplementary Figure 131. **^{13}C NMR** of the **6p** (101 MHz, $CDCl_3$)

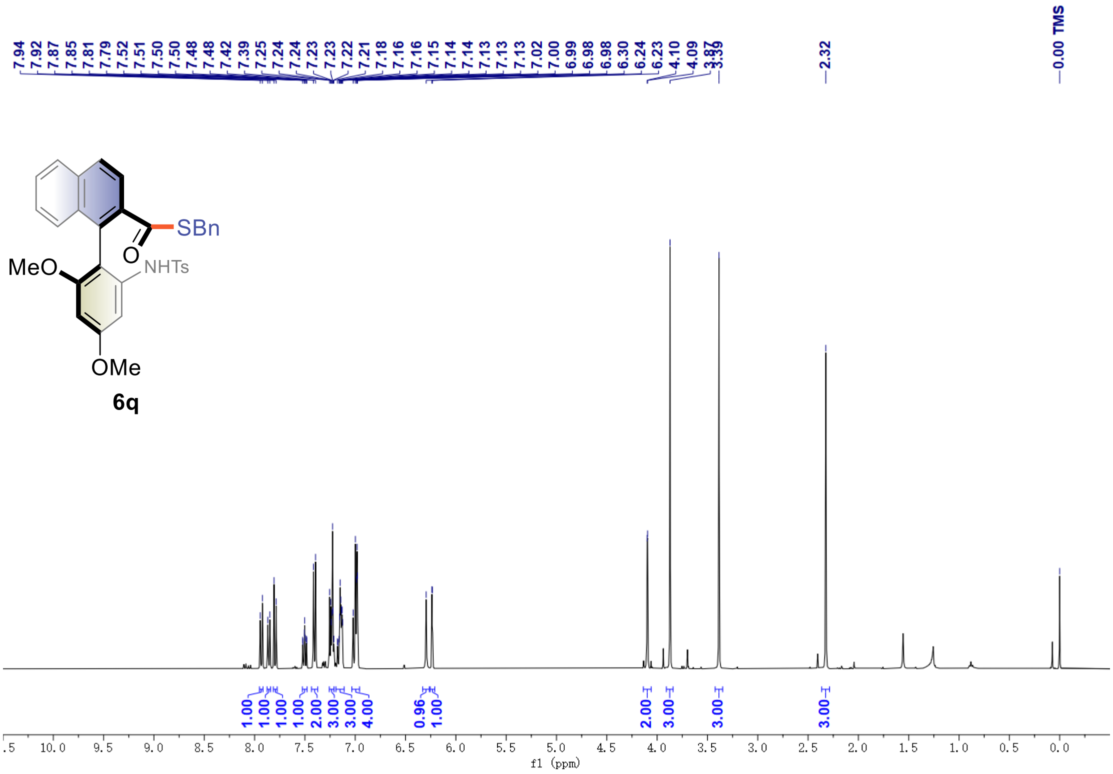

Supplementary Figure 132. **^{1}H NMR** of the **6q** (400 MHz, $CDCl_3$)

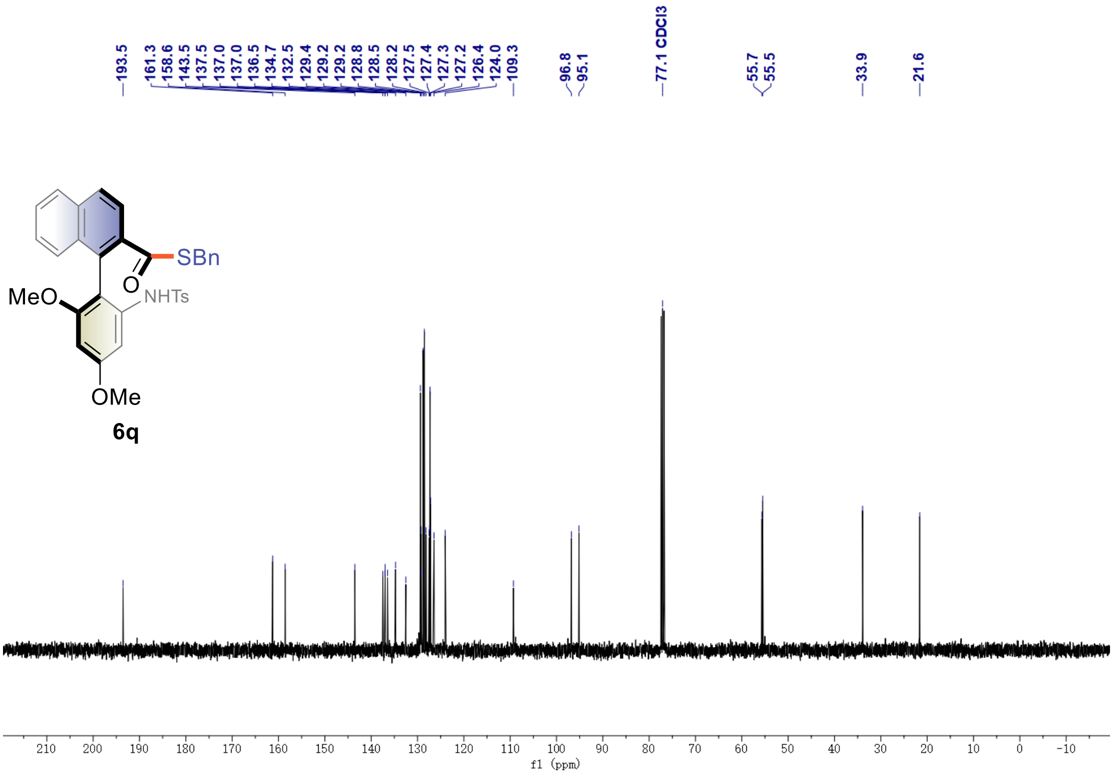

Supplementary Figure 133. **^{13}C NMR** of the **6q** (101 MHz, $CDCl_3$)

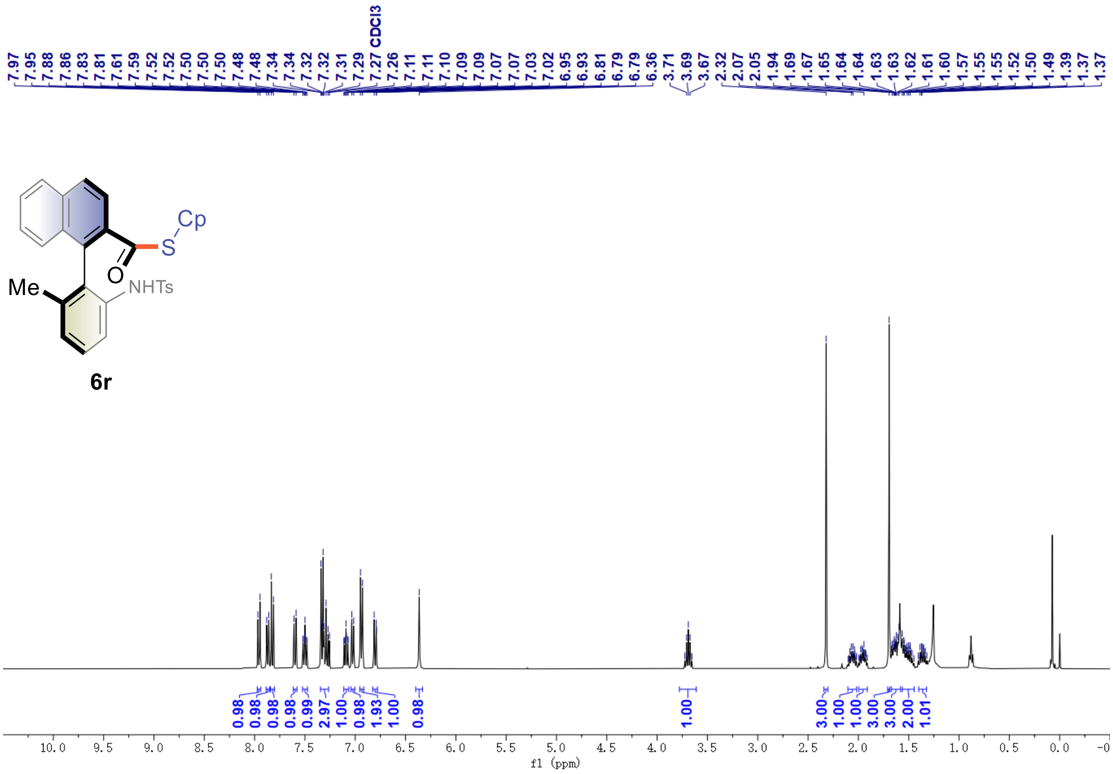

Supplementary Figure 134. ^{1}H NMR of the **6r** (400 MHz, $CDCl_3$)

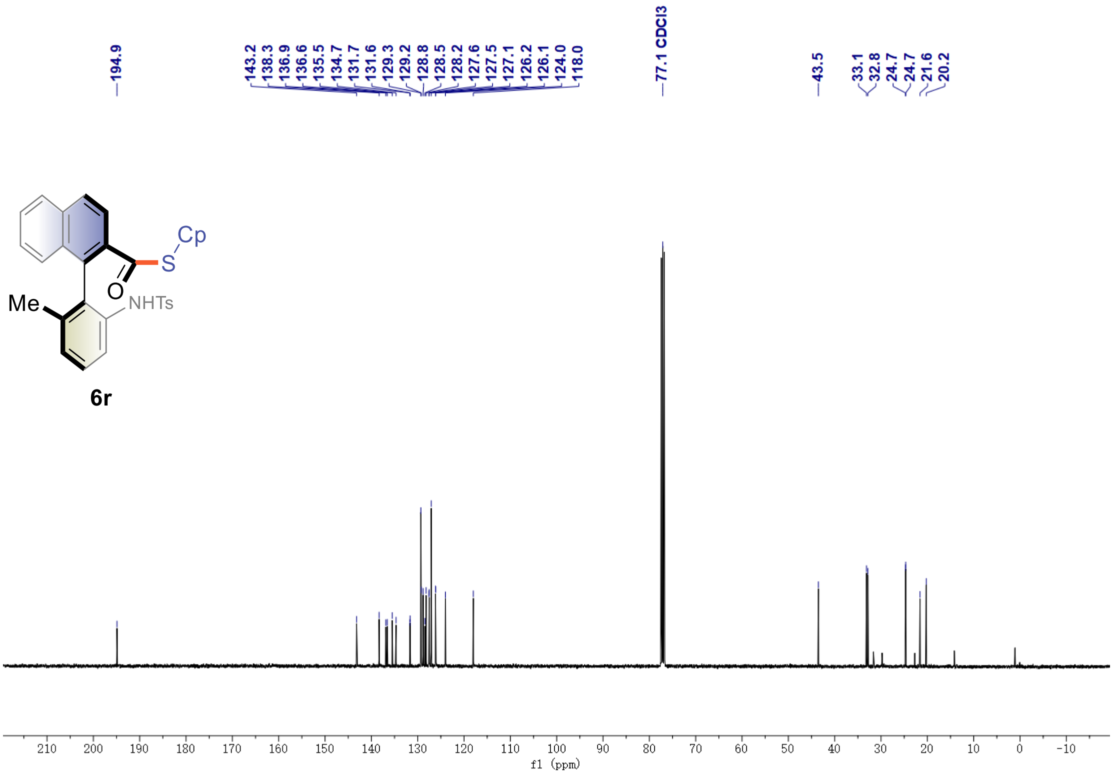

Supplementary Figure 135. ^{13}C NMR of the **6r** (101 MHz, $CDCl_3$)

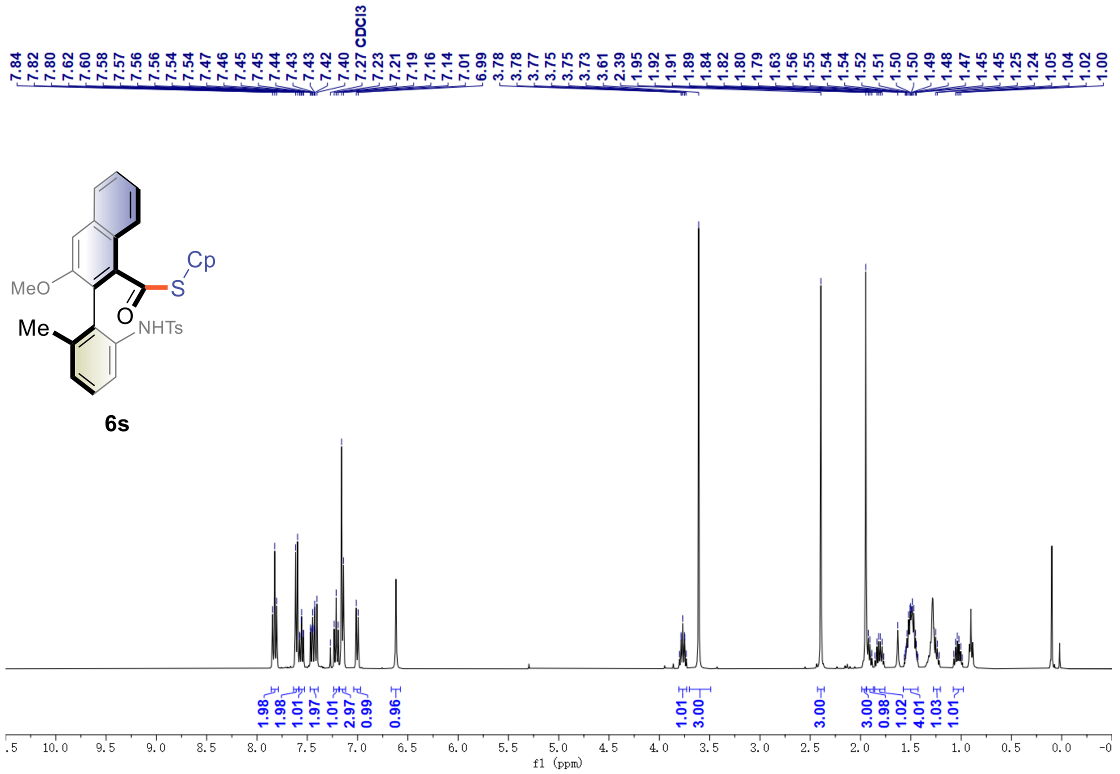

Supplementary Figure 136. **^{1}H NMR** of the **6s** (400 MHz, $CDCl_3$)

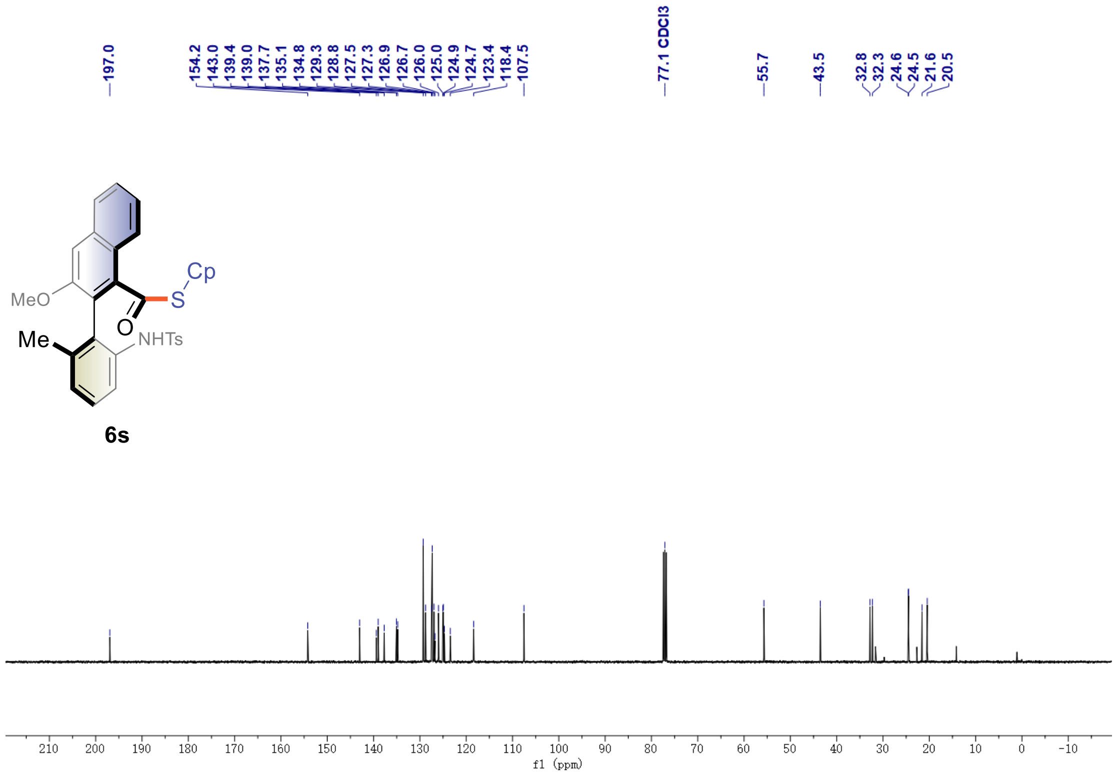

Supplementary Figure 137. **^{13}C NMR** of the **6s** (101 MHz, $CDCl_3$)

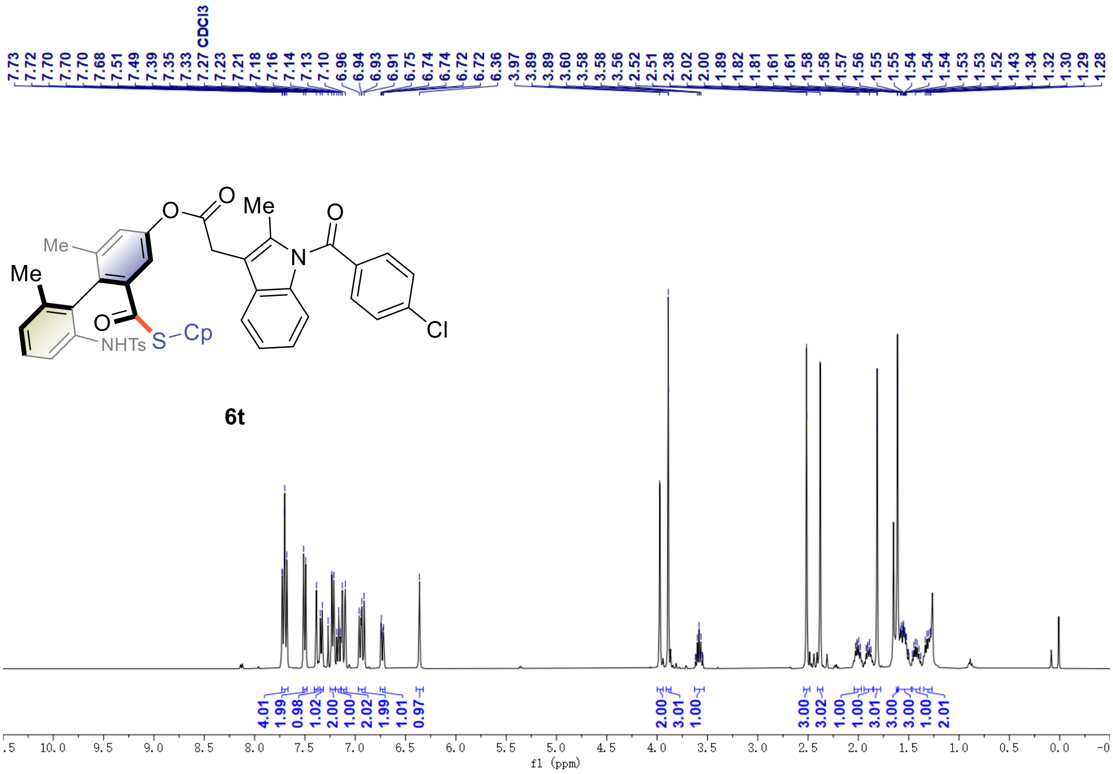

Supplementary Figure 138. ^{1}**H NMR** of the **6t** (400 MHz, $CDCl_3$)

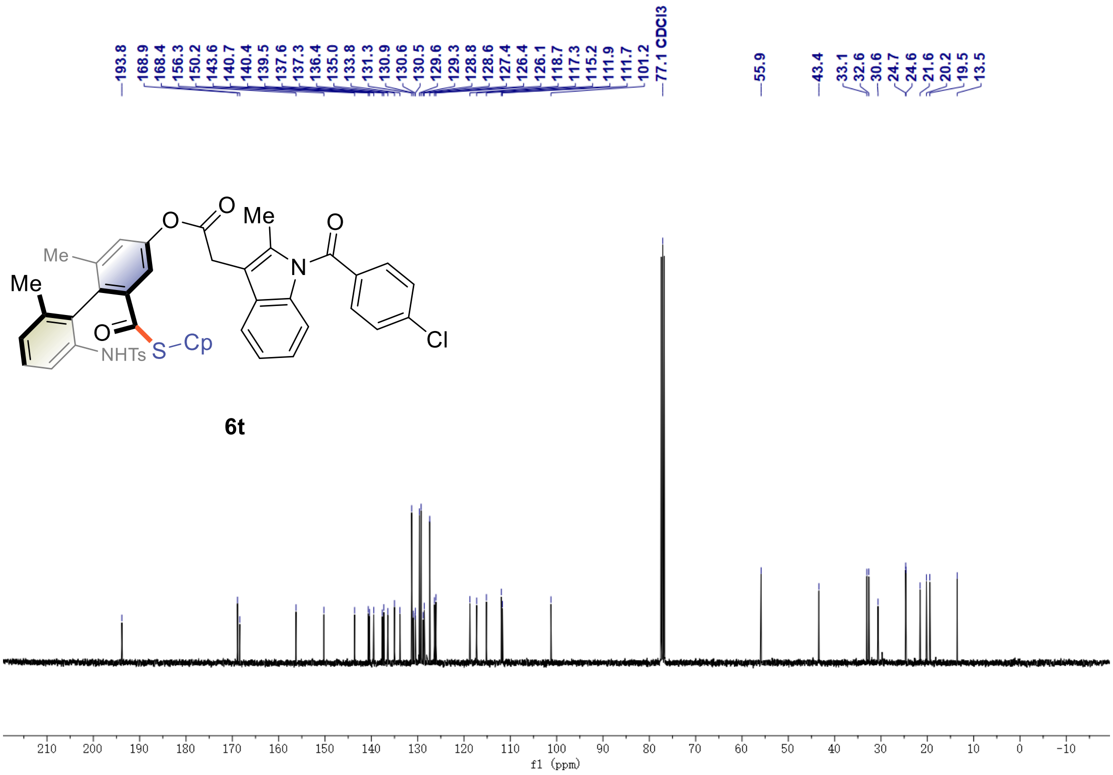

Supplementary Figure 139. ^{13}**C NMR** of the **6t** (101 MHz, $CDCl_3$)

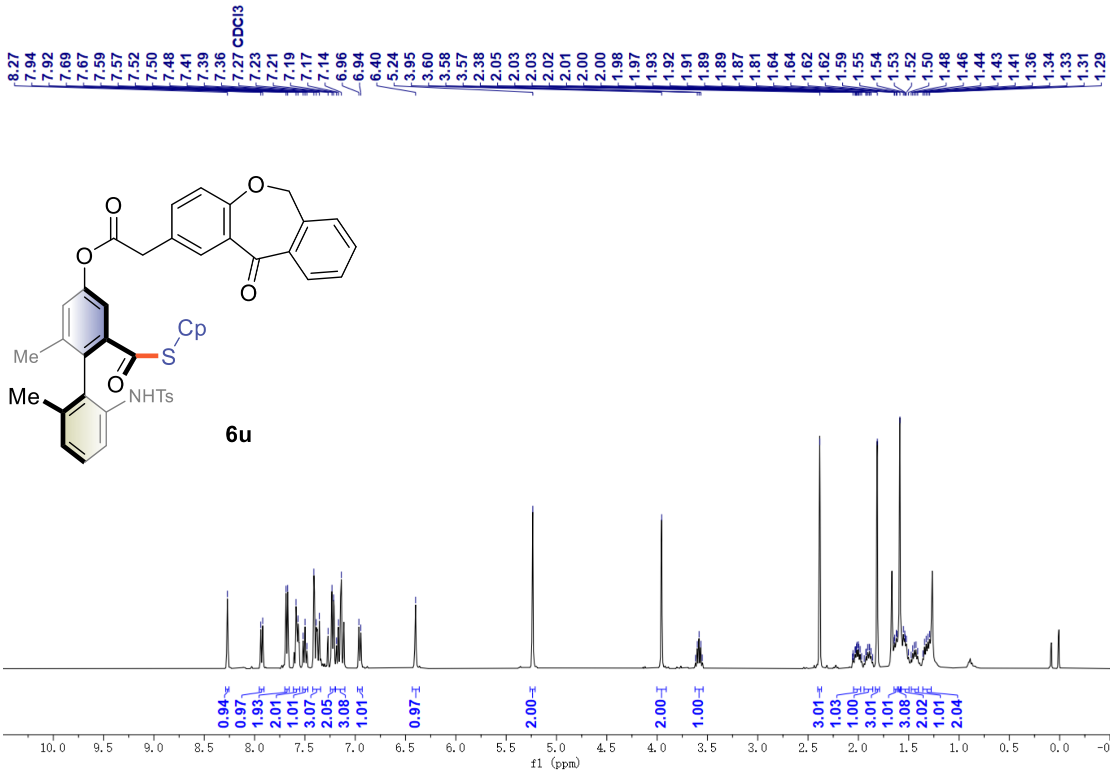

Supplementary Figure 140. ^{1}**H NMR** of the **6u** (400 MHz, $CDCl_3$)

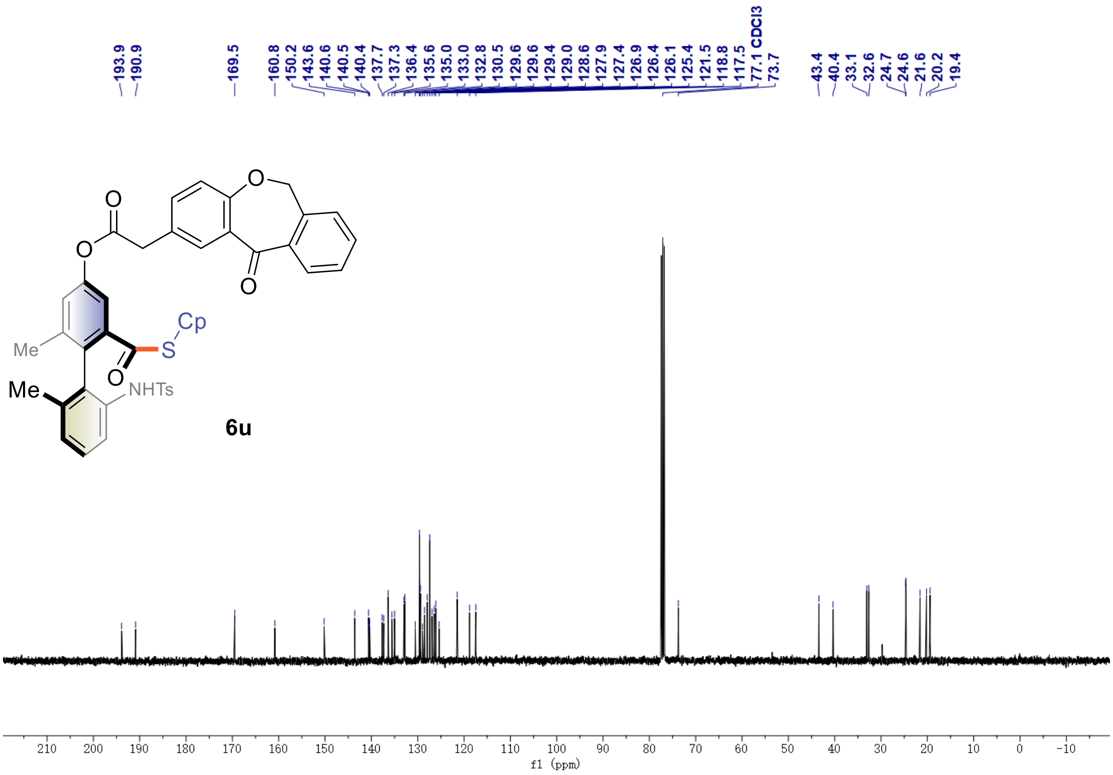

Supplementary Figure 141. ^{13}**C NMR** of the **6u** (101 MHz, $CDCl_3$)

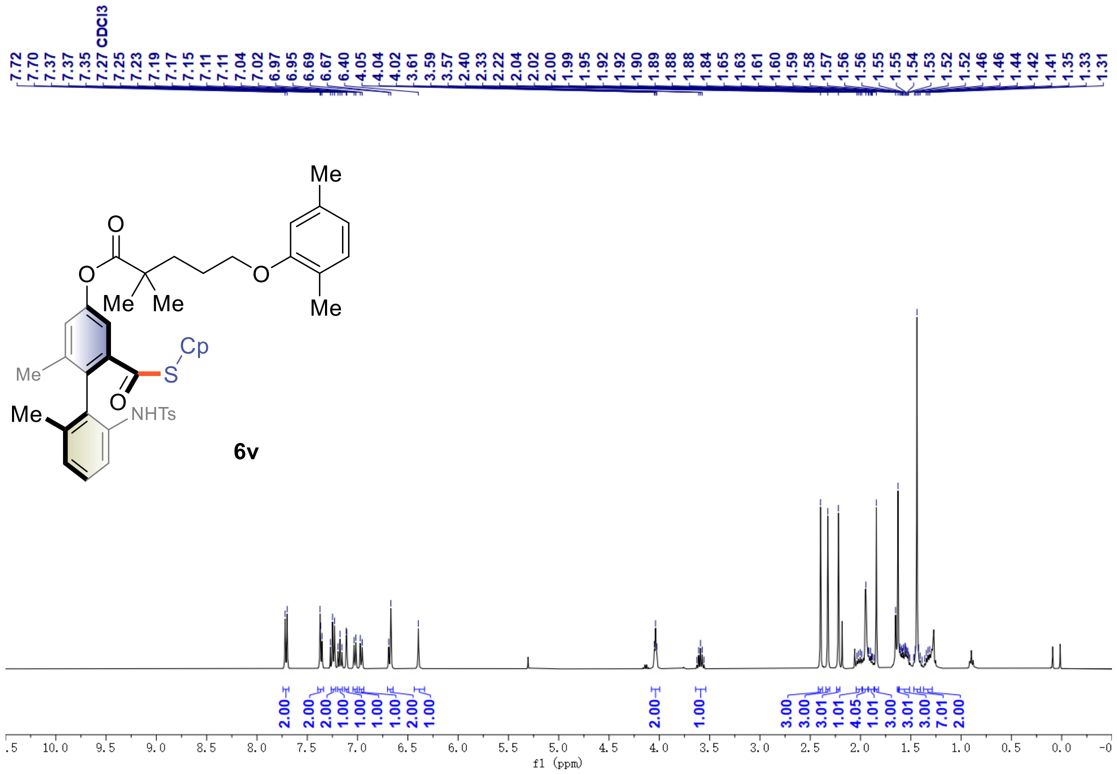

Supplementary Figure 142. ^{1}**H NMR** of the **6v** (400 MHz, $CDCl_3$)

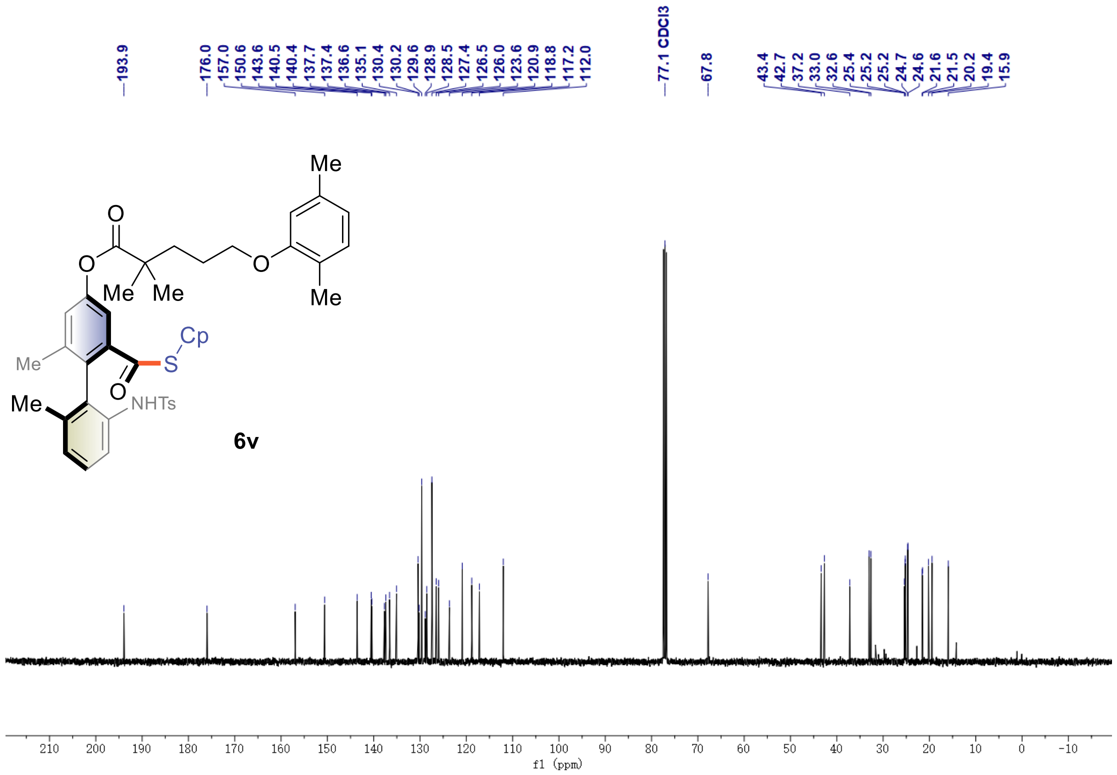

Supplementary Figure 143. ^{13}**C NMR** of the **6v** (101 MHz, $CDCl_3$)

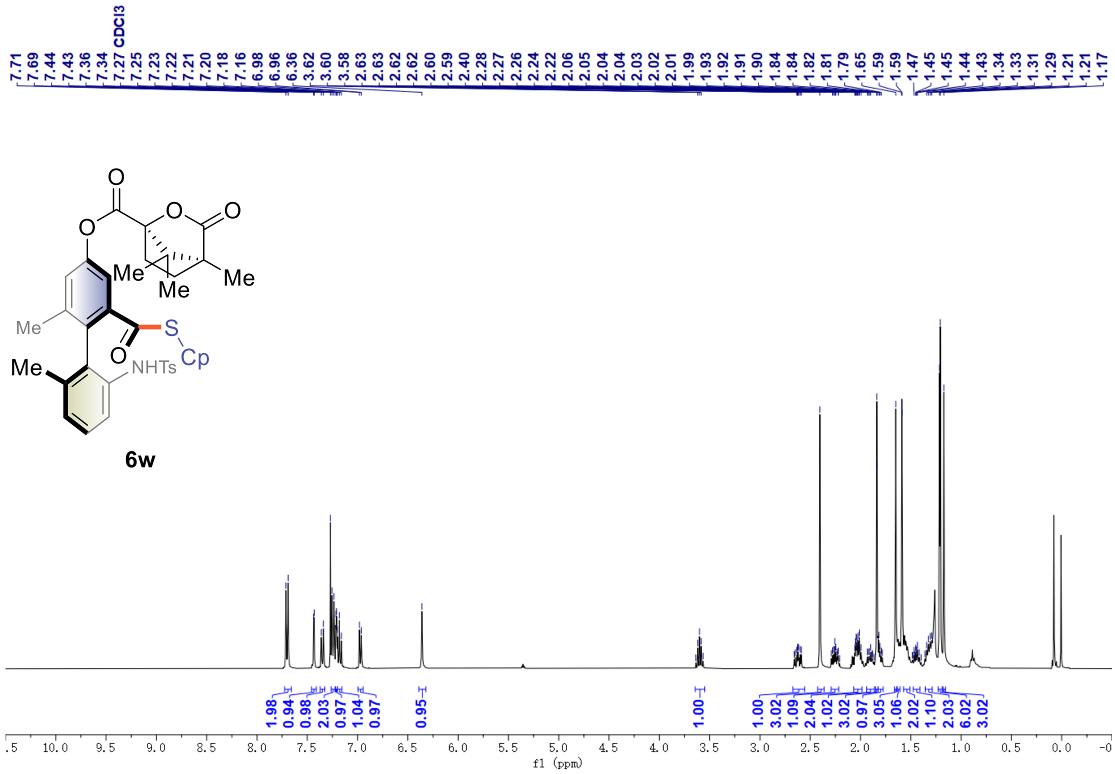

Supplementary Figure 144. ^{1}H NMR of the **6w** (400 MHz, $CDCl_3$)

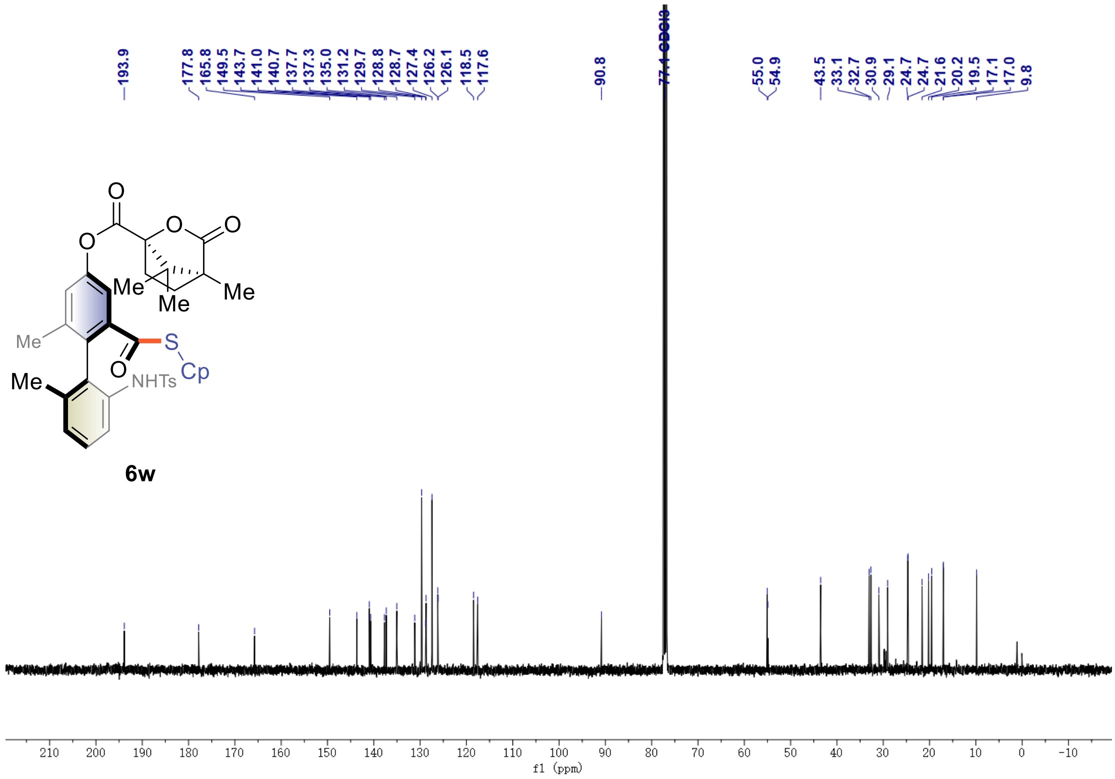

Supplementary Figure 145. ^{13}C NMR of the **6w** (101 MHz, $CDCl_3$)

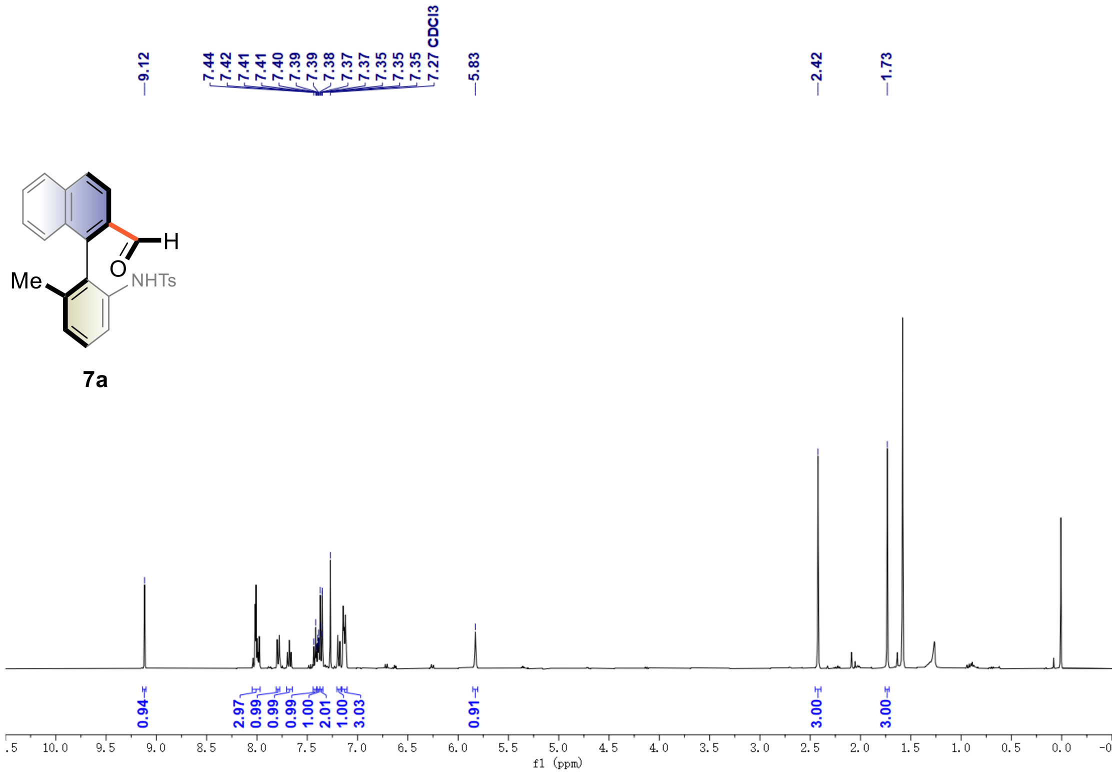

Supplementary Figure 146. ^{1}**H NMR** of the **7a** (400 MHz, $CDCl_3$)

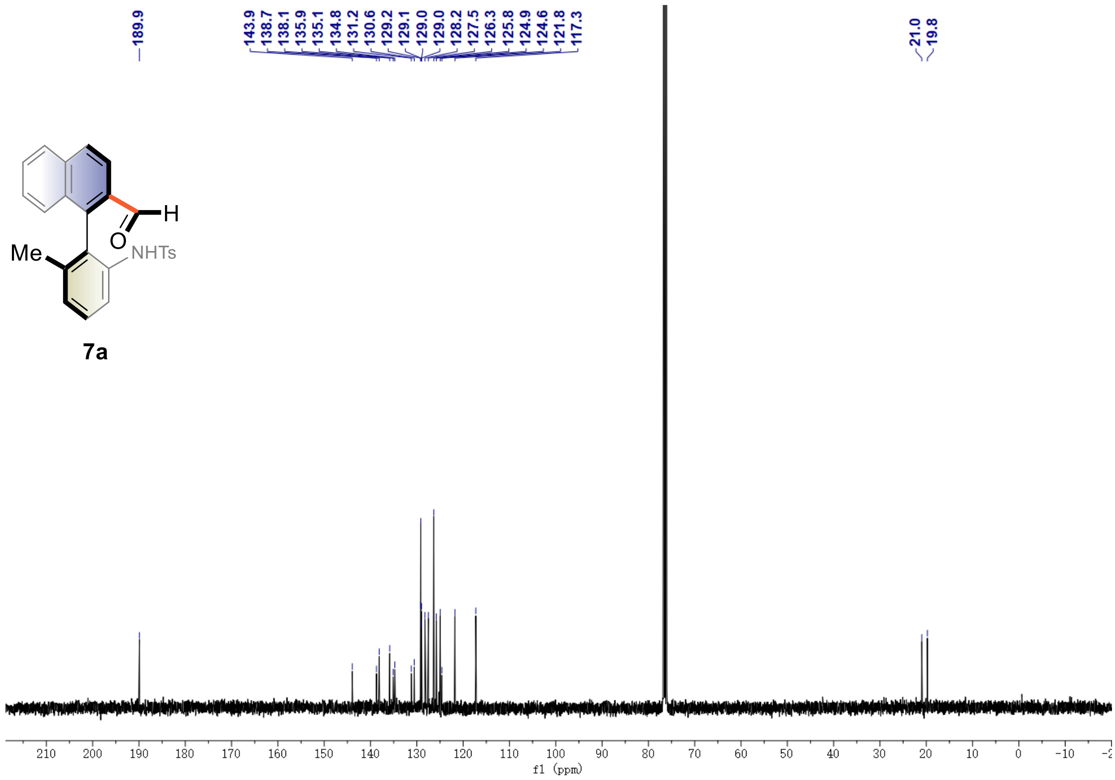

Supplementary Figure 147. ^{13}**C NMR** of the **7a** (101 MHz, $CDCl_3$)

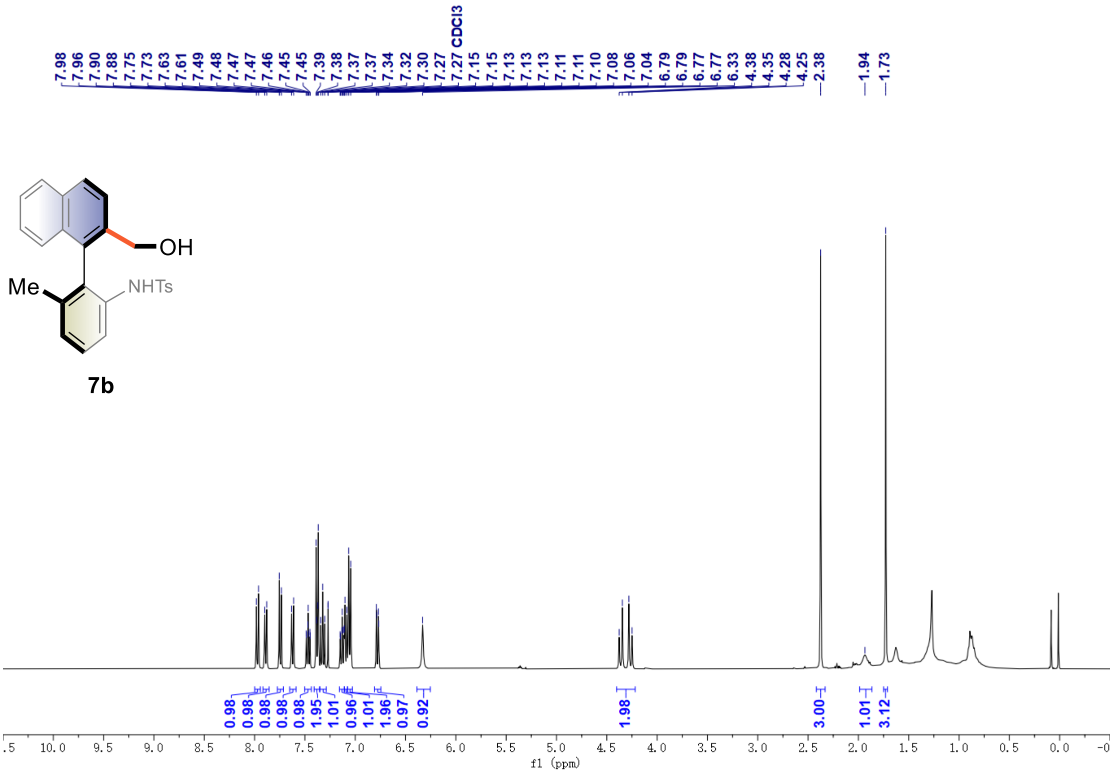

Supplementary Figure 148. ^{1}**H NMR** of the **7b** (400 MHz, $CDCl_3$)

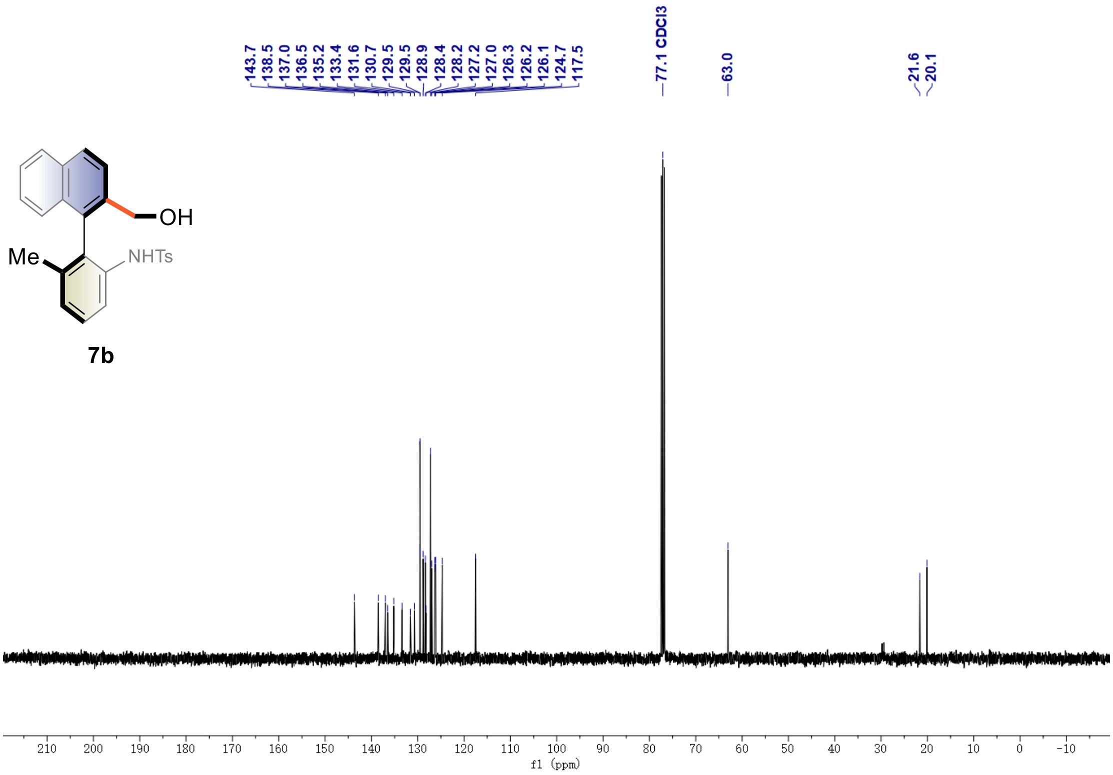

Supplementary Figure 149. ^{13}**C NMR** of the **7b** (101 MHz, $CDCl_3$)

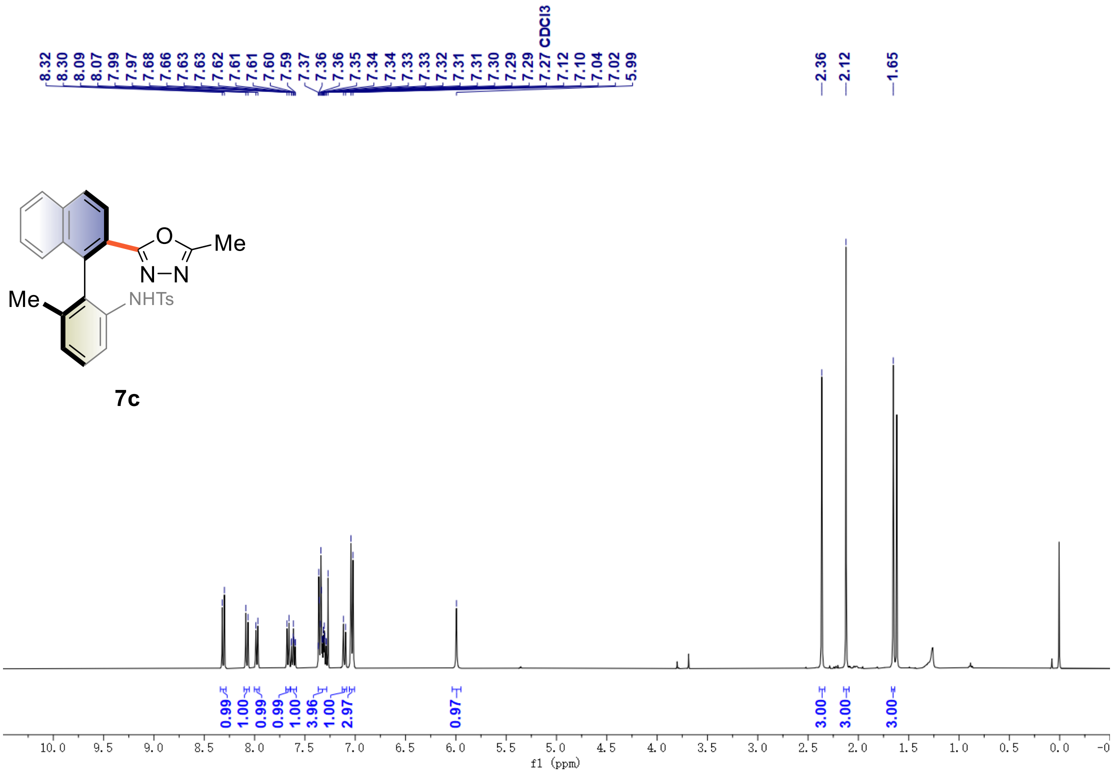

Supplementary Figure 150. ^{1}**H NMR** of the **7c** (400 MHz, $CDCl_3$)

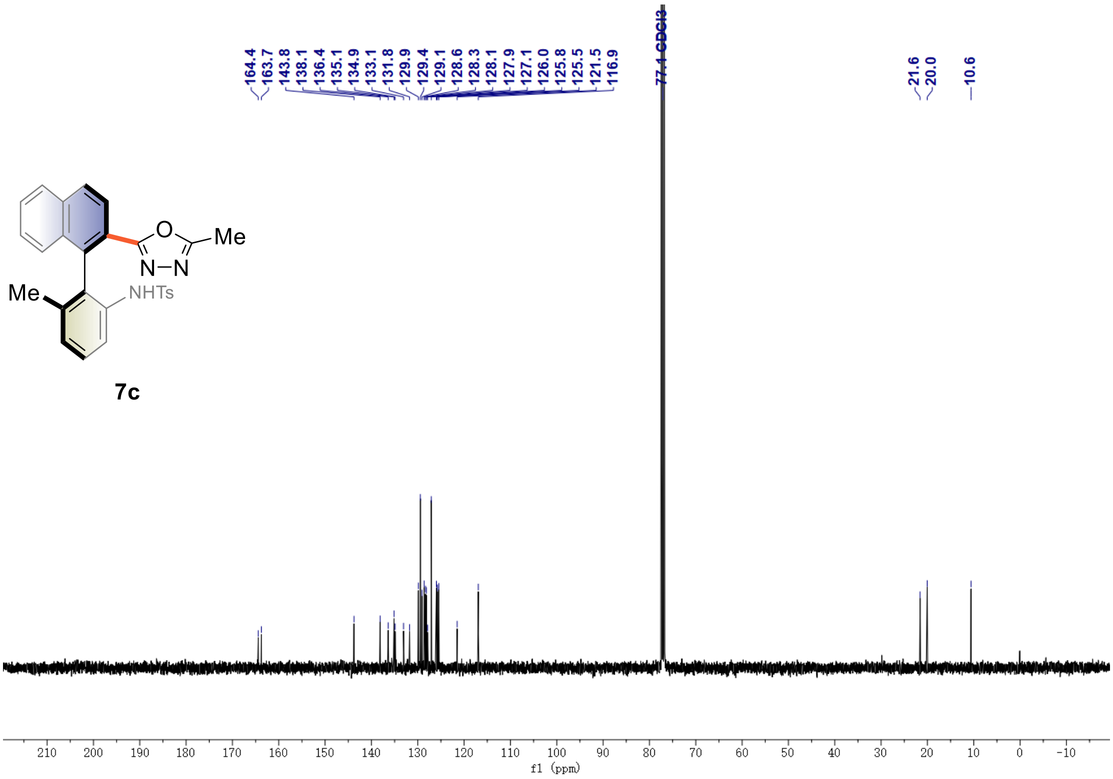

Supplementary Figure 151. ^{13}**C NMR** of the **7c** (101 MHz, $CDCl_3$)

3.2 HPLC datas

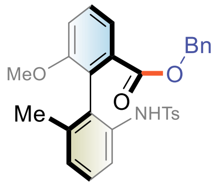

4a (95% ee)

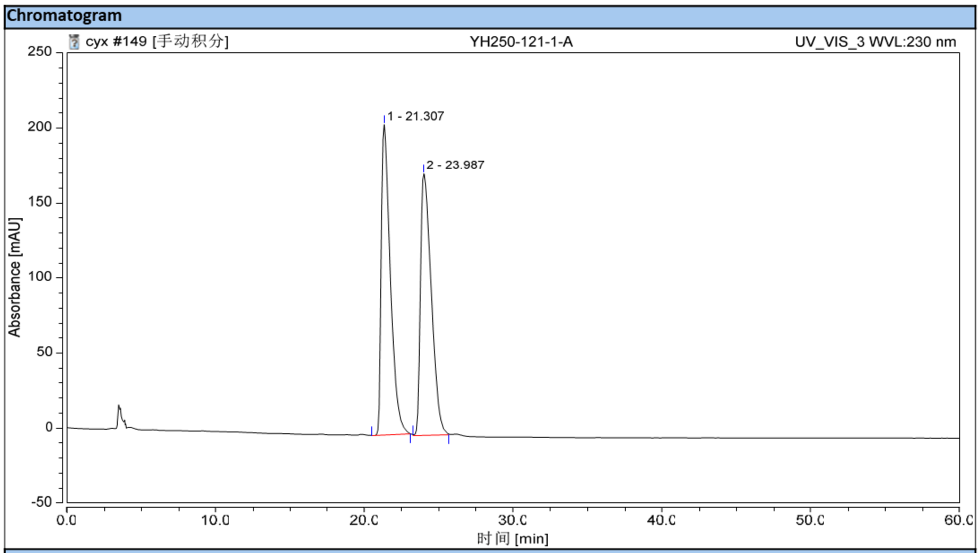

Integration Results

No.	Peak Name	Retention Time min	Area mAu*min	Height mAU	Relative Area %	Relative Height %	Amount n.a.
1		21.307	146.558	206.823	50.19	54.33	n.a.
2		23.987	145.435	173.836	49.81	45.67	n.a.
**Total:**			**291.993**	**380.659**	**100.00**	**100.00**	

Supplementary Figure 152. HPLC spectrum of racemic **4a**

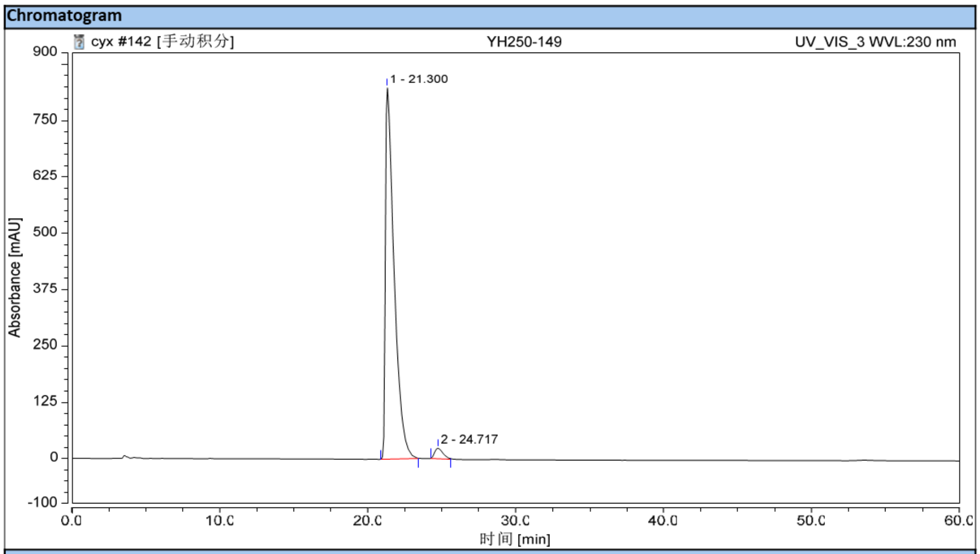

Integration Results

No.	Peak Name	Retention Time min	Area mAu*min	Height mAU	Relative Area %	Relative Height %	Amount n.a.
1		21.300	561.628	824.667	97.64	97.39	n.a.
2		24.717	13.561	22.115	2.36	2.61	n.a.
**Total:**			**575.190**	**846.782**	**100.00**	**100.00**	

Supplementary Figure 153. HPLC spectrum of **4a**

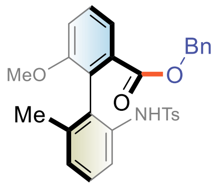

4a (97% ee)

Chromatogram

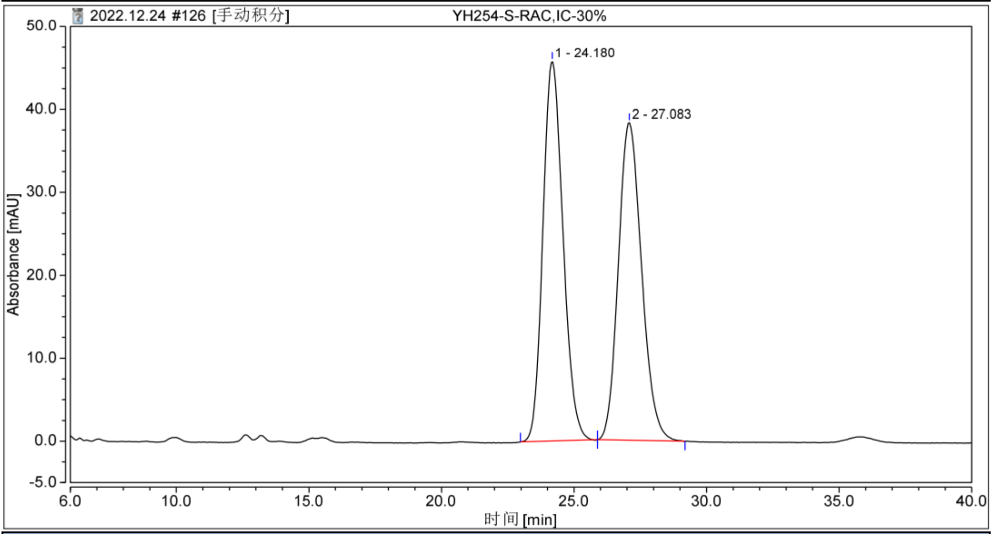

Integration Results

No.	Peak Name	Retention Time min	Area mAu*min	Height mAU	Relative Area %	Relative Height %	Amount n.a.
1		24.180	40.180	45.796	50.79	54.45	n.a.
2		27.083	38.934	38.304	49.21	45.55	n.a.
**Total:**			**79.114**	**84.100**	**100.00**	**100.00**	

Supplementary Figure 154. HPLC spectrum of racemic **4a**

Chromatogram

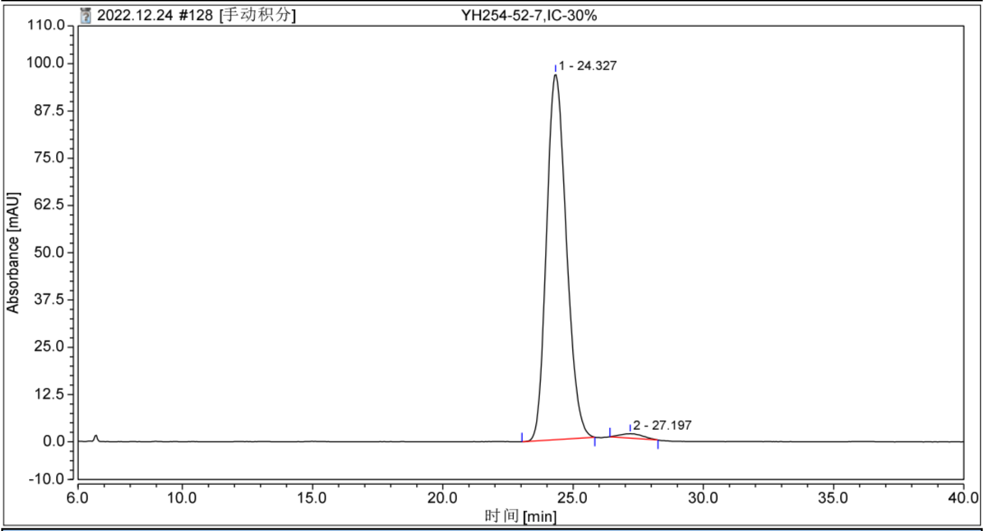

Integration Results

No.	Peak Name	Retention Time min	Area mAu*min	Height mAU	Relative Area %	Relative Height %	Amount n.a.
1		24.327	85.427	96.741	98.62	98.81	n.a.
2		27.197	1.198	1.166	1.38	1.19	n.a.
**Total:**			**86.625**	**97.906**	**100.00**	**100.00**	

Supplementary Figure 155. HPLC spectrum of **4a**

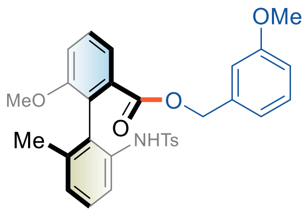

4b

Chromatogram

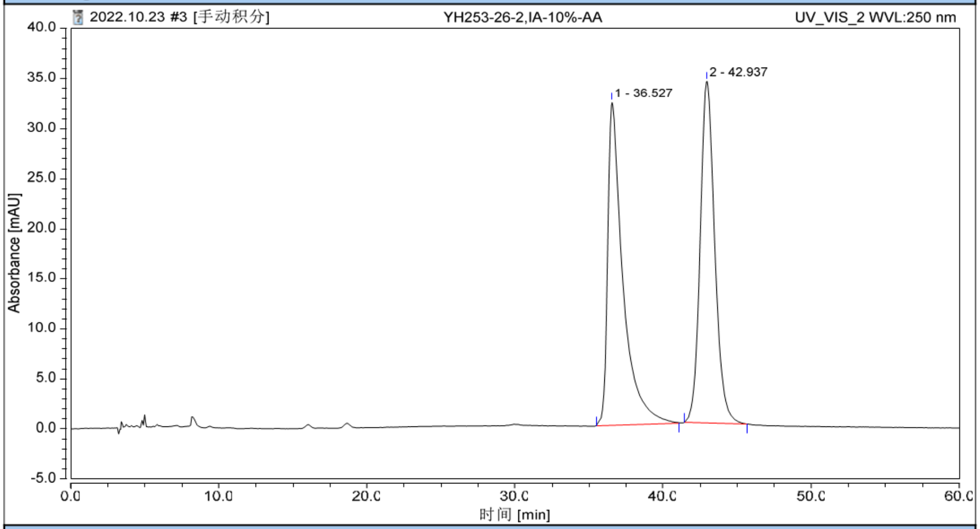

Integration Results

No.	Peak Name	Retention Time min	Area mAu*min	Height mAU	Relative Area %	Relative Height %	Amount n.a.
1		36.527	38.332	32.261	49.68	48.58	n.a.
2		42.937	38.828	34.140	50.32	51.42	n.a.
**Total:**			**77.161**	**66.400**	**100.00**	**100.00**	

Supplementary Figure 156. HPLC spectrum of racemic **4b**

Chromatogram

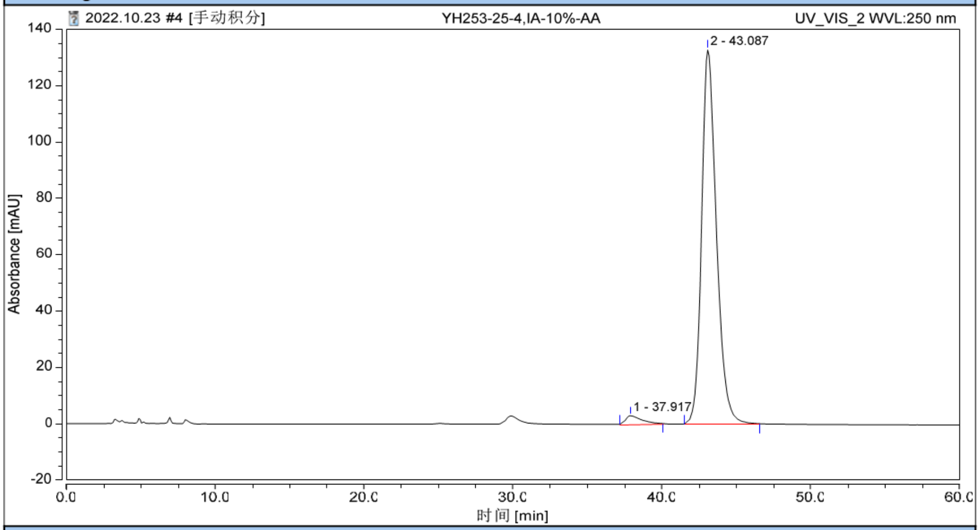

Integration Results

No.	Peak Name	Retention Time min	Area mAu*min	Height mAU	Relative Area %	Relative Height %	Amount n.a.
1		37.917	3.516	2.960	2.24	2.18	n.a.
2		43.087	153.167	132.966	97.76	97.82	n.a.
**Total:**			**156.683**	**135.926**	**100.00**	**100.00**	

Supplementary Figure 157. HPLC spectrum of **4b**

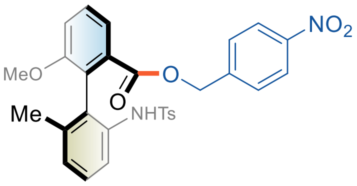

4c

Chromatogram

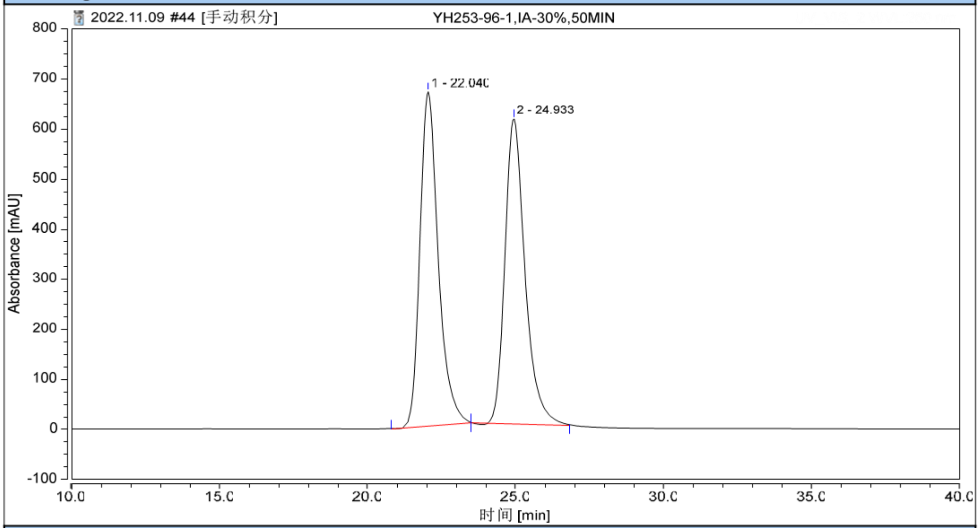

Integration Results

No.	Peak Name	Retention Time min	Area mAu*min	Height mAU	Relative Area %	Relative Height %	Amount n.a.
1		22.040	477.272	667.837	50.05	52.29	n.a.
2		24.933	476.257	609.342	49.95	47.71	n.a.
**Total:**			**953.529**	**1277.179**	**100.00**	**100.00**	

Supplementary Figure 158. HPLC spectrum of racemic **4c**

Chromatogram

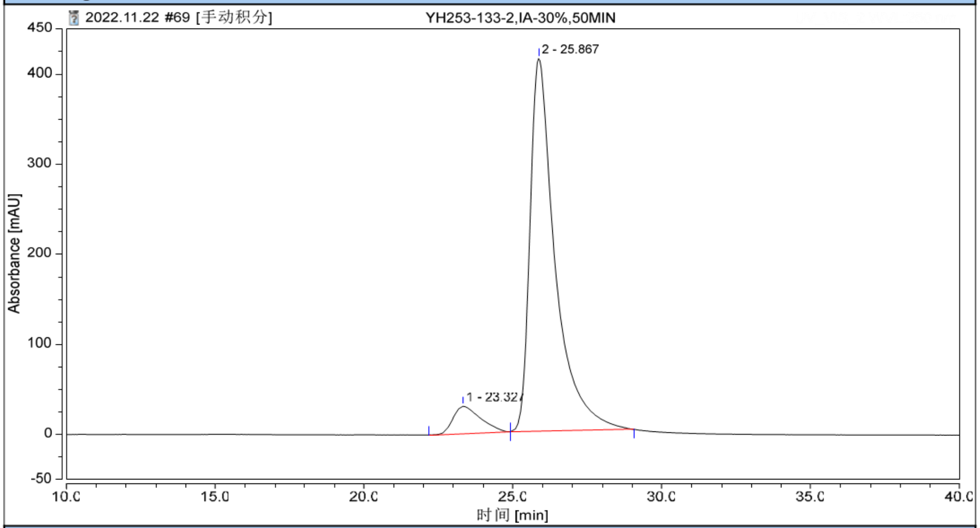

Integration Results

No.	Peak Name	Retention Time min	Area mAu*min	Height mAU	Relative Area %	Relative Height %	Amount n.a.
1		23.327	32.274	30.356	7.38	6.83	n.a.
2		25.867	405.028	414.102	92.62	93.17	n.a.
**Total:**			**437.302**	**444.458**	**100.00**	**100.00**	

Supplementary Figure 159. HPLC spectrum of **4c**

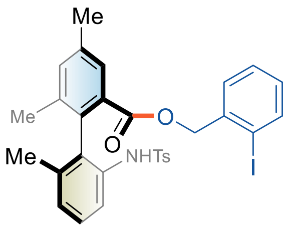

4d

Chromatogram

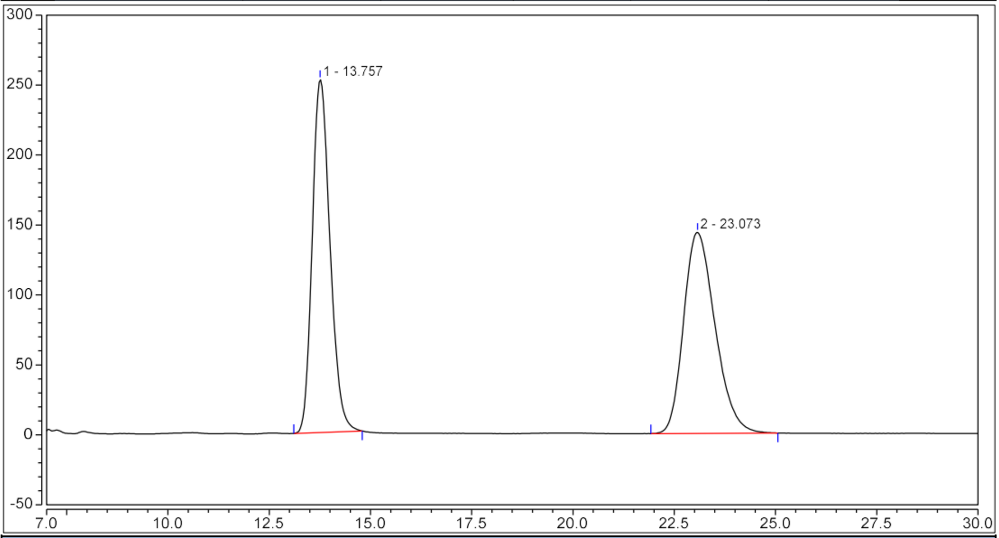

Integration Results

No.	Peak Name	Retention Time min	Area mAu*min	Height mAU	Relative Area %	Relative Height %	Amount n.a.
1		13.757	128.321	252.209	49.77	63.69	n.a.
2		23.073	129.511	143.760	50.23	36.31	n.a.
**Total:**			**257.832**	**395.969**	**100.00**	**100.00**	

Supplementary Figure 160. HPLC spectrum of racemic **4d**

Chromatogram

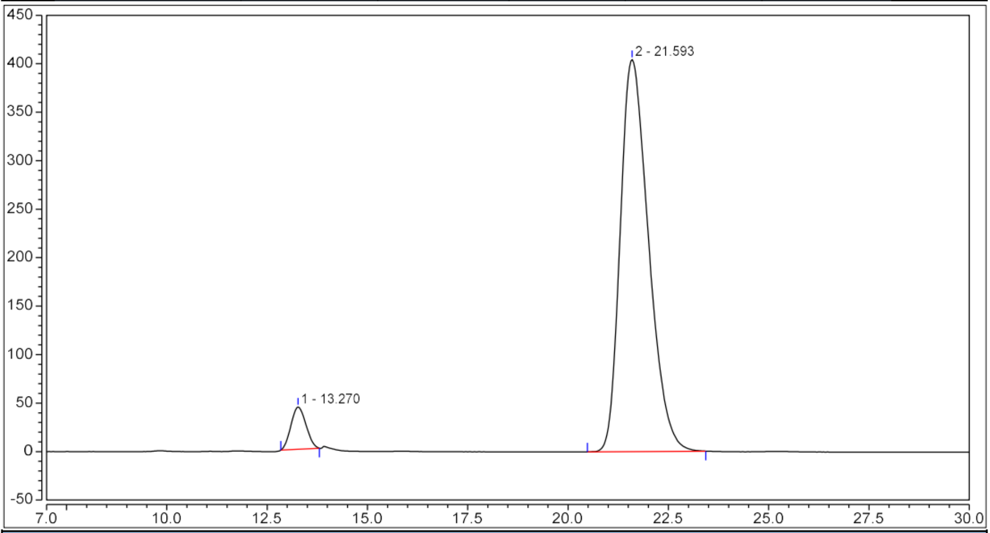

Integration Results

No.	Peak Name	Retention Time min	Area mAu*min	Height mAU	Relative Area %	Relative Height %	Amount n.a.
1		13.270	18.953	43.536	5.42	9.72	n.a.
2		21.593	330.947	404.334	94.58	90.28	n.a.
**Total:**			**349.900**	**447.870**	**100.00**	**100.00**	

Supplementary Figure 161. HPLC spectrum of **4d**

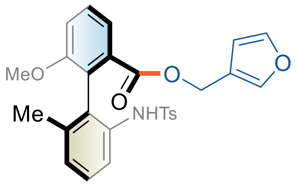

4e

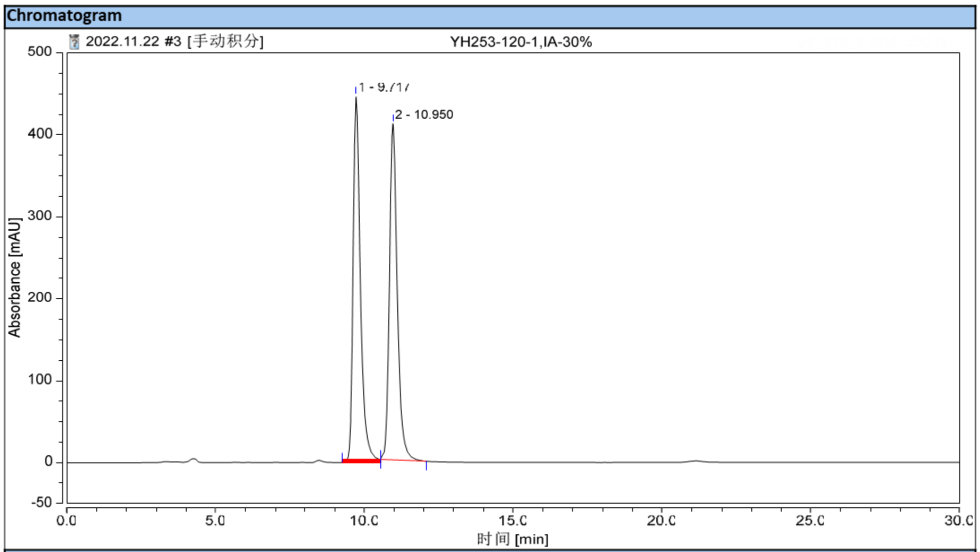

Integration Results

No.	Peak Name	Retention Time<br>min	Area<br>mAu*min	Height<br>mAU	Relative Area<br>%	Relative Height<br>%	Amount<br>n.a.
1		9.717	125.071	444.831	49.84	52.01	n.a.
2		10.950	125.863	410.397	50.16	47.99	n.a.
**Total:**			**250.934**	**855.228**	**100.00**	**100.00**	

Supplementary Figure 162. HPLC spectrum of racemic **4e**

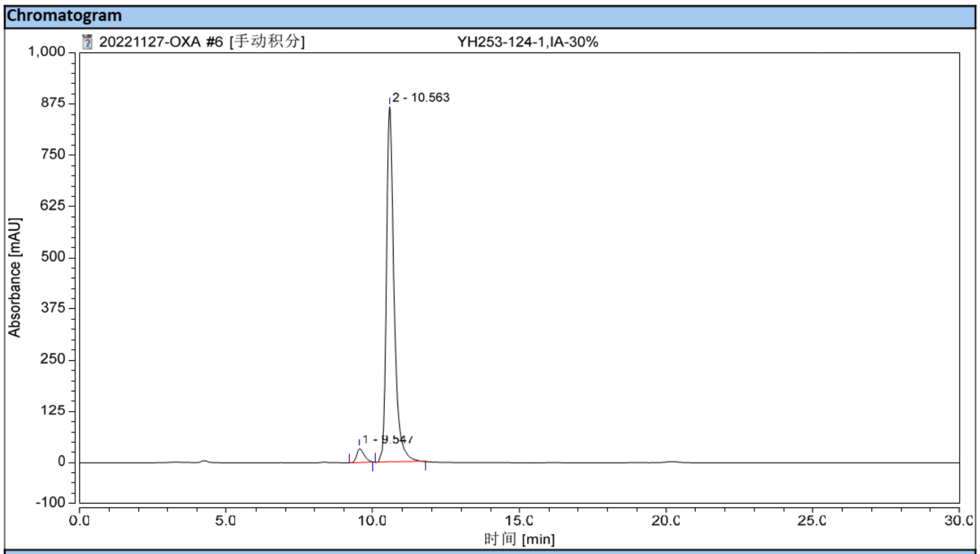

Integration Results

No.	Peak Name	Retention Time<br>min	Area<br>mAu*min	Height<br>mAU	Relative Area<br>%	Relative Height<br>%	Amount<br>n.a.
1		9.547	9.840	33.478	3.64	3.71	n.a.
2		10.563	260.433	867.922	96.36	96.29	n.a.
**Total:**			**270.273**	**901.400**	**100.00**	**100.00**	

Supplementary Figure 163. HPLC spectrum of **4e**

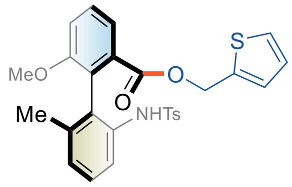

4f

Chromatogram

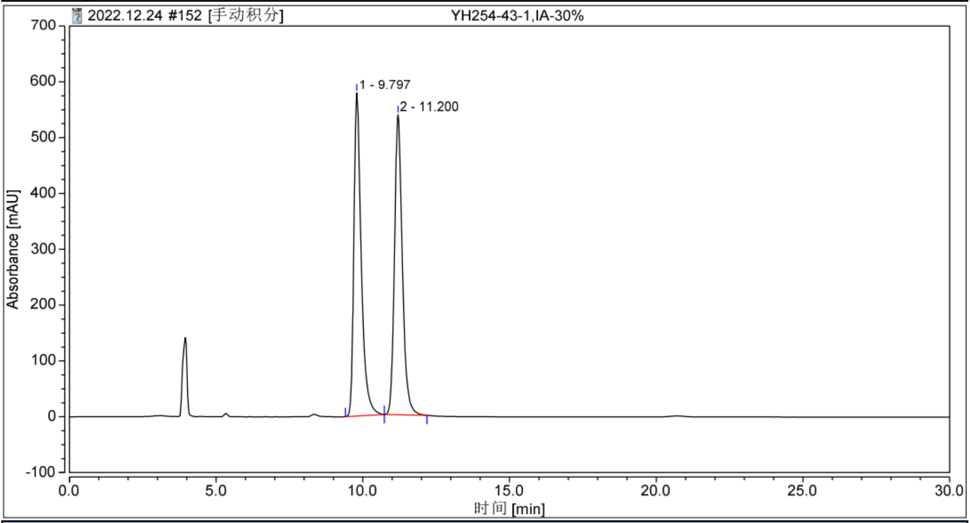

Integration Results

No.	Peak Name	Retention Time<br>min	Area<br>mAu*min	Height<br>mAU	Relative Area<br>%	Relative Height<br>%	Amount<br>n.a.
1		9.797	167.850	578.883	50.05	51.85	n.a.
2		11.200	167.514	537.610	49.95	48.15	n.a.
**Total:**			**335.364**	**1116.493**	**100.00**	**100.00**	

Supplementary Figure 164. HPLC spectrum of racemic **4f**

Chromatogram

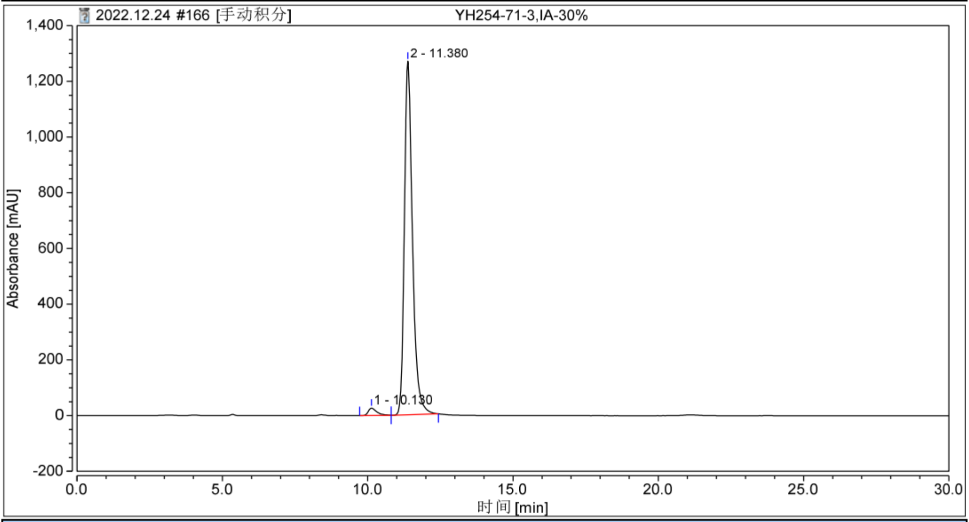

Integration Results

No.	Peak Name	Retention Time<br>min	Area<br>mAu*min	Height<br>mAU	Relative Area<br>%	Relative Height<br>%	Amount<br>n.a.
1		10.130	9.054	26.642	2.16	2.06	n.a.
2		11.380	410.426	1269.303	97.84	97.94	n.a.
**Total:**			**419.479**	**1295.945**	**100.00**	**100.00**	

Supplementary Figure 165. HPLC spectrum of **4f**

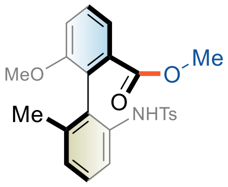

4g

Chromatogram

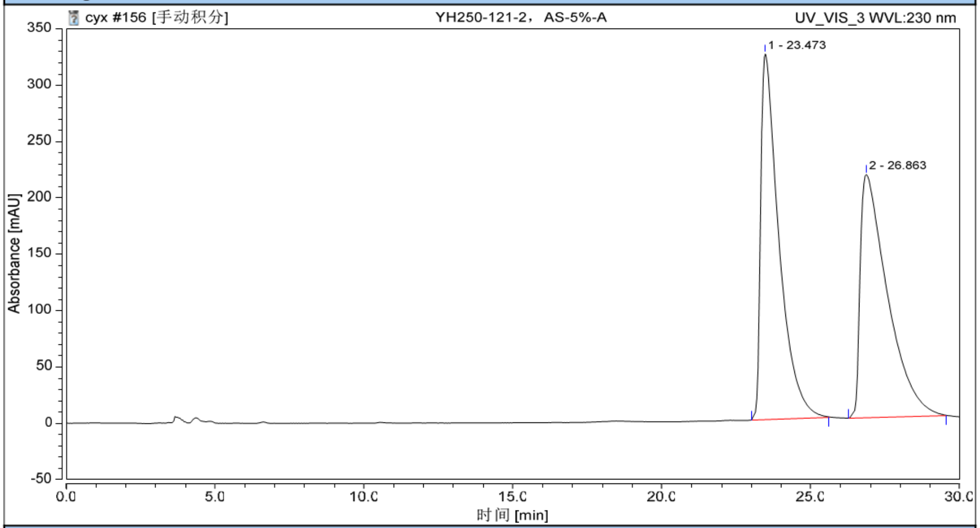

Integration Results

No.	Peak Name	Retention Time min	Area mAu*min	Height mAU	Relative Area %	Relative Height %	Amount n.a.
1		23.473	230.083	324.238	50.24	60.02	n.a.
2		26.863	227.888	216.005	49.76	39.98	n.a.
**Total:**			**457.971**	**540.243**	**100.00**	**100.00**	

Supplementary Figure 166. HPLC spectrum of racemic **4g**

Chromatogram

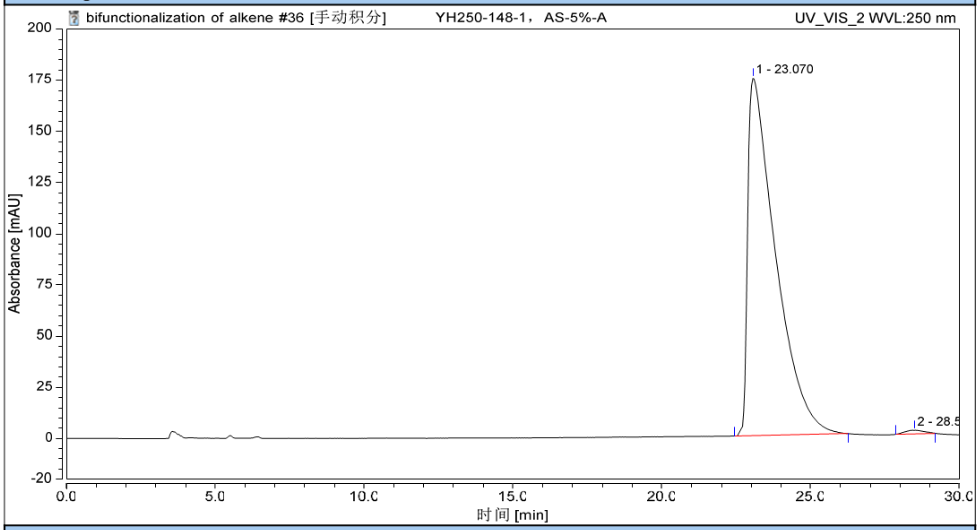

Integration Results

No.	Peak Name	Retention Time min	Area mAu*min	Height mAU	Relative Area %	Relative Height %	Amount n.a.
1		23.070	189.324	174.811	99.31	99.02	n.a.
2		28.503	1.312	1.738	0.69	0.98	n.a.
**Total:**			**190.637**	**176.550**	**100.00**	**100.00**	

Supplementary Figure 167. HPLC spectrum of **4g**

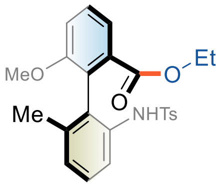

4h

Chromatogram

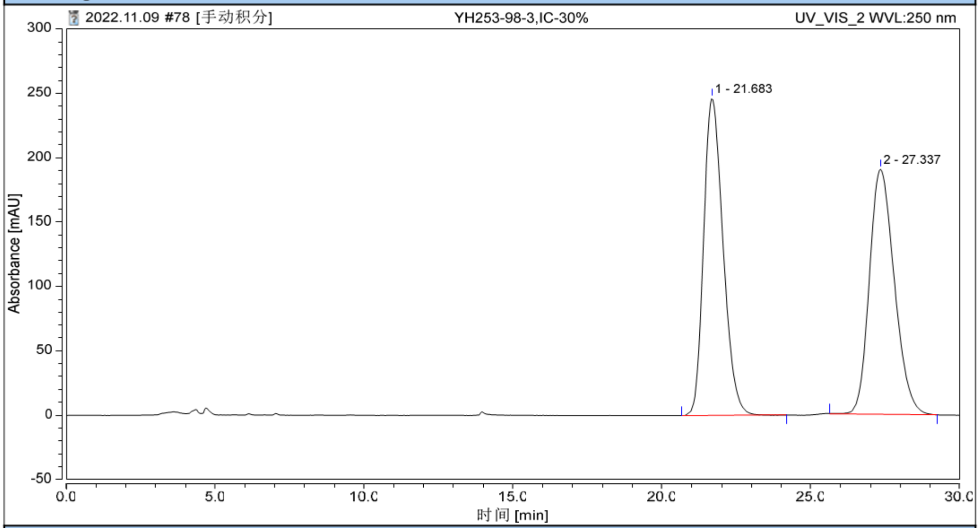

Integration Results

No.	Peak Name	Retention Time min	Area mAu*min	Height mAU	Relative Area %	Relative Height %	Amount n.a.
1		21.683	186.788	246.601	50.24	56.43	n.a.
2		27.337	184.997	190.439	49.76	43.57	n.a.
**Total:**			**371.786**	**437.041**	**100.00**	**100.00**	

Supplementary Figure 168. HPLC spectrum of racemic **4h**

Chromatogram

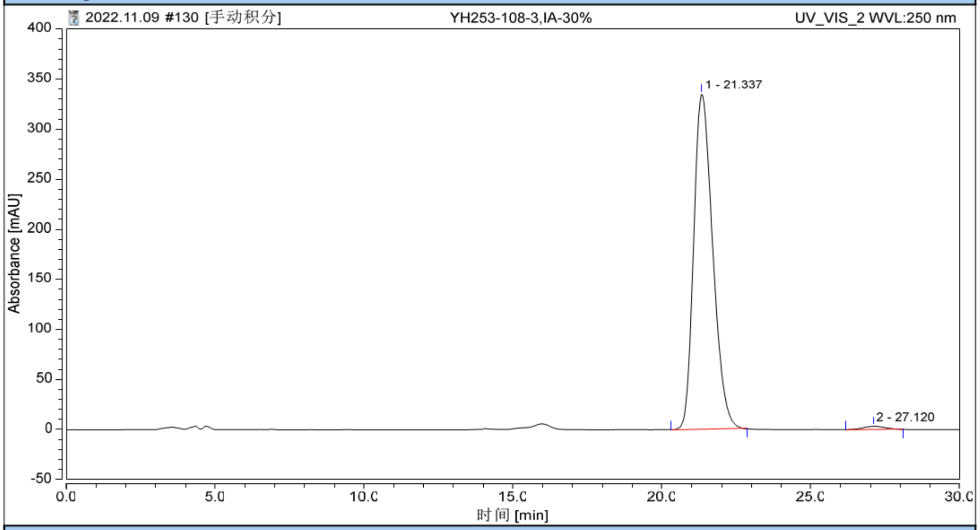

Integration Results

No.	Peak Name	Retention Time min	Area mAu*min	Height mAU	Relative Area %	Relative Height %	Amount n.a.
1		21.337	248.713	335.065	98.85	99.02	n.a.
2		27.120	2.905	3.302	1.15	0.98	n.a.
**Total:**			**251.618**	**338.368**	**100.00**	**100.00**	

Supplementary Figure 169. HPLC spectrum of **4h**

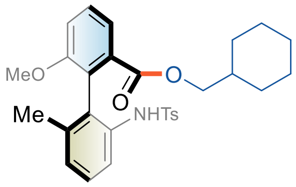

4i

Chromatogram

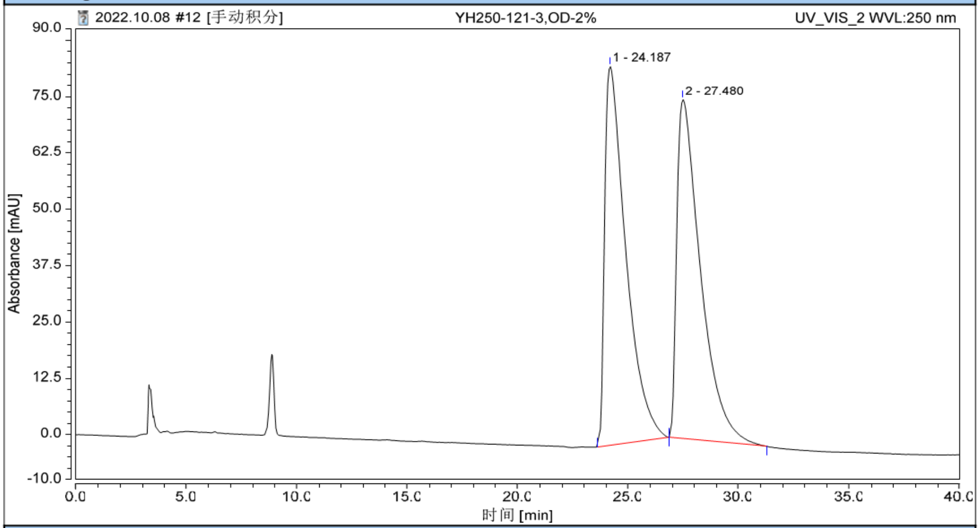

Integration Results

No.	Peak Name	Retention Time min	Area mAu*min	Height mAU	Relative Area %	Relative Height %	Amount n.a.
1		24.187	93.343	84.088	49.95	52.79	n.a.
2		27.480	93.534	75.200	50.05	47.21	n.a.
**Total:**			**186.876**	**159.288**	**100.00**	**100.00**	

Supplementary Figure 170. HPLC spectrum of racemic **4i**

Chromatogram

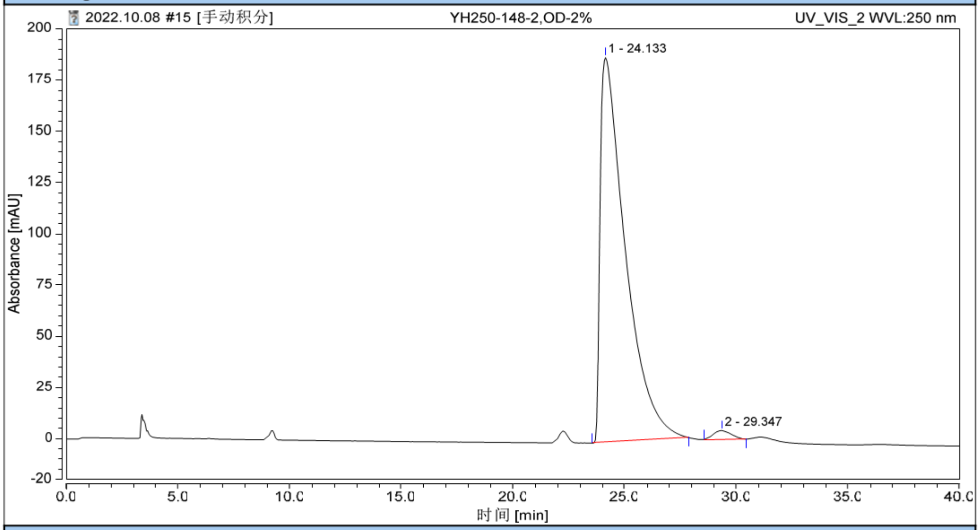

Integration Results

No.	Peak Name	Retention Time min	Area mAu*min	Height mAU	Relative Area %	Relative Height %	Amount n.a.
1		24.133	245.598	187.928	98.45	97.82	n.a.
2		29.347	3.859	4.182	1.55	2.18	n.a.
**Total:**			**249.457**	**192.110**	**100.00**	**100.00**	

Supplementary Figure 171. HPLC spectrum of **4i**

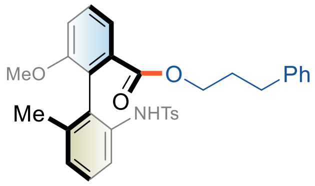

4j

Chromatogram

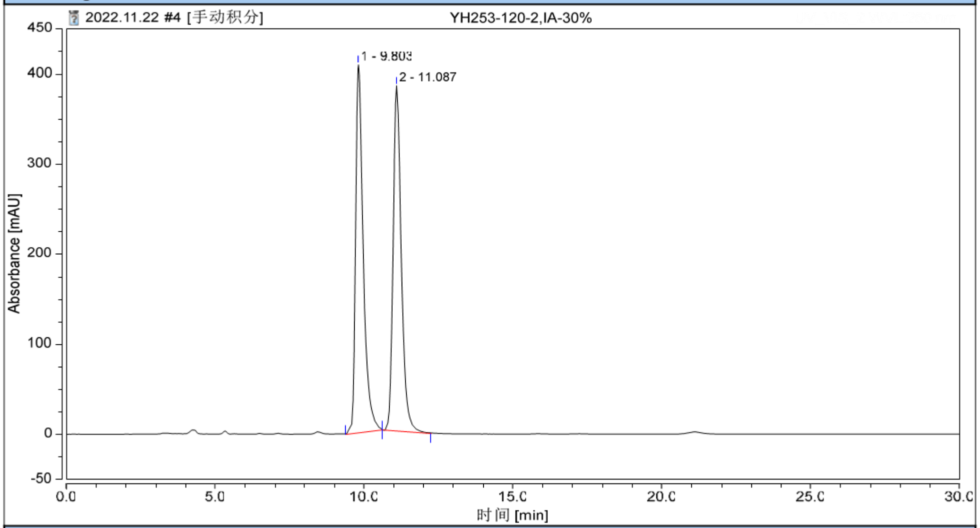

Integration Results

No.	Peak Name	Retention Time min	Area mAu*min	Height mAU	Relative Area %	Relative Height %	Amount n.a.
1		9.803	120.648	408.034	49.84	51.58	n.a.
2		11.087	121.444	382.990	50.16	48.42	n.a.
Total:			242.092	791.024	100.00	100.00	

Supplementary Figure 172. HPLC spectrum of racemic **4j**

Chromatogram

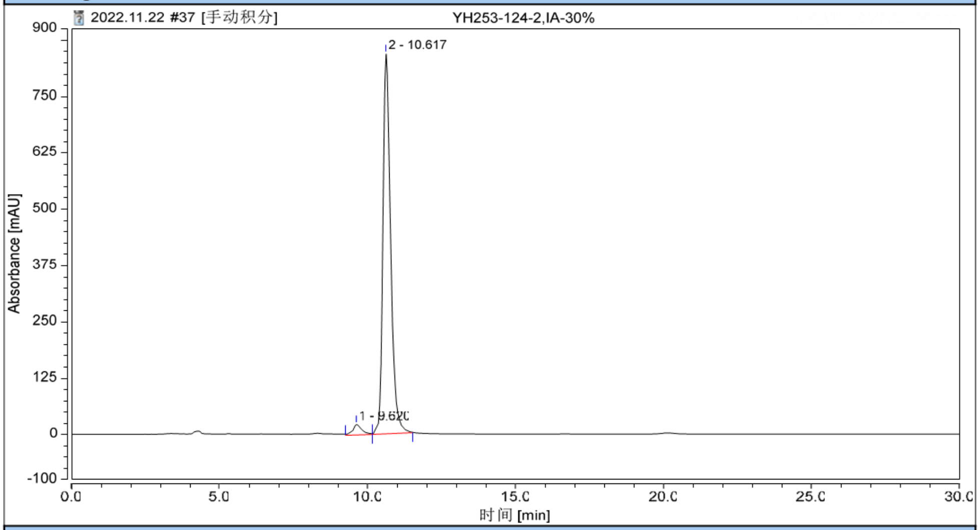

Integration Results

No.	Peak Name	Retention Time min	Area mAu*min	Height mAU	Relative Area %	Relative Height %	Amount n.a.
1		9.620	6.241	20.765	2.42	2.40	n.a.
2		10.617	251.253	842.926	97.58	97.60	n.a.
Total:			257.494	863.691	100.00	100.00	

Supplementary Figure 173. HPLC spectrum of **4j**

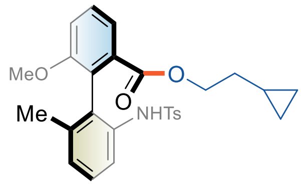

4k

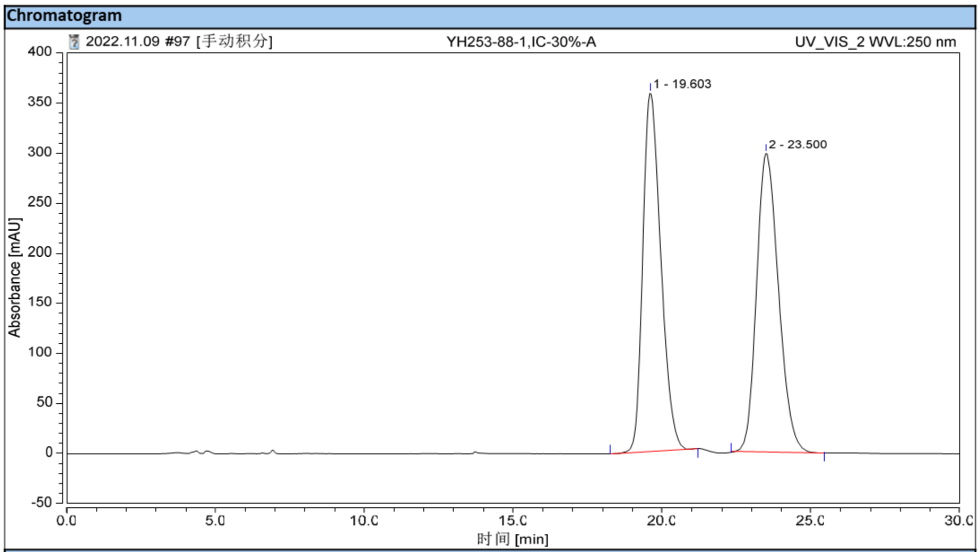

Integration Results

No.	Peak Name	Retention Time min	Area mAu*min	Height mAU	Relative Area %	Relative Height %	Amount n.a.
1		19.603	250.293	357.652	49.44	54.50	n.a.
2		23.500	255.991	298.539	50.56	45.50	n.a.
**Total:**			**506.285**	**656.191**	**100.00**	**100.00**	

Supplementary Figure 174. HPLC spectrum of racemic **4k**

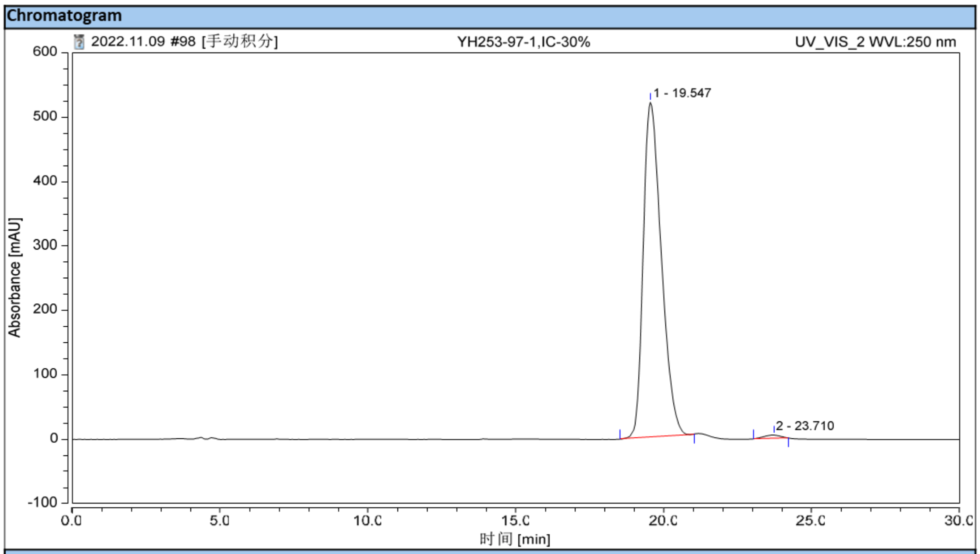

Integration Results

No.	Peak Name	Retention Time min	Area mAu*min	Height mAU	Relative Area %	Relative Height %	Amount n.a.
1		19.547	367.914	520.081	99.12	99.05	n.a.
2		23.710	3.280	4.970	0.88	0.95	n.a.
**Total:**			**371.194**	**525.051**	**100.00**	**100.00**	

Supplementary Figure 175. HPLC spectrum of **4k**

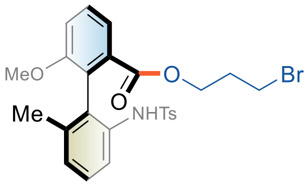

4l

Chromatogram

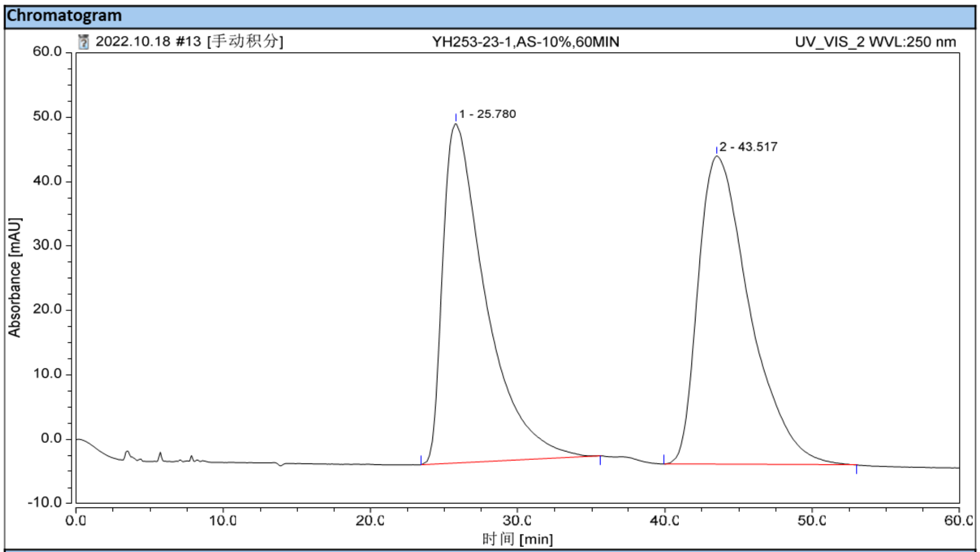

Integration Results

No.	Peak Name	Retention Time min	Area mAu*min	Height mAU	Relative Area %	Relative Height %	Amount n.a.
1		25.780	178.509	52.793	47.81	52.40	n.a.
2		43.517	194.861	47.965	52.19	47.60	n.a.
**Total:**			**373.370**	**100.758**	**100.00**	**100.00**	

Supplementary Figure 176. HPLC spectrum of racemic **4l**

Chromatogram

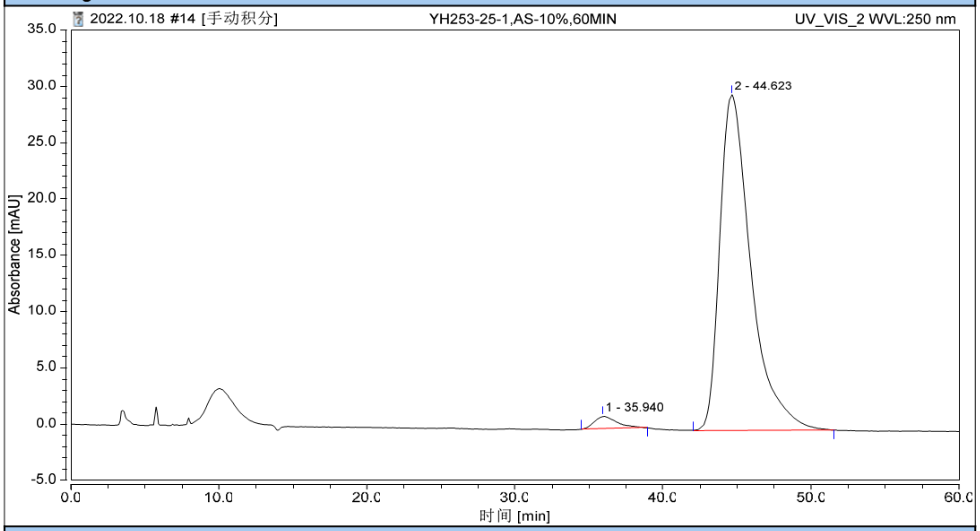

Integration Results

No.	Peak Name	Retention Time min	Area mAu*min	Height mAU	Relative Area %	Relative Height %	Amount n.a.
1		35.940	1.908	1.107	2.56	3.57	n.a.
2		44.623	72.722	29.853	97.44	96.43	n.a.
**Total:**			**74.630**	**30.960**	**100.00**	**100.00**	

Supplementary Figure 177. HPLC spectrum of **4l**

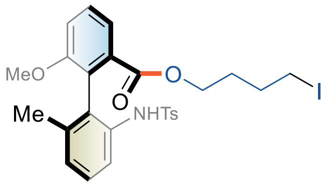

4m

Chromatogram

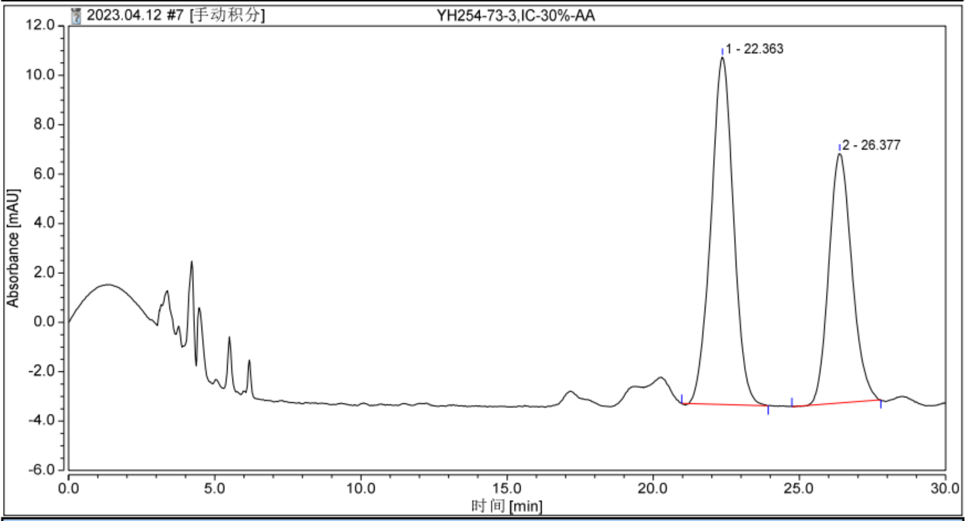

Integration Results

No.	Peak Name	Retention Time min	Area mAu*min	Height mAU	Relative Area %	Relative Height %	Amount n.a.
1		22.363	12.590	14.065	57.71	58.17	n.a.
2		26.377	9.225	10.114	42.29	41.83	n.a.
**Total:**			**21.815**	**24.179**	**100.00**	**100.00**	

Supplementary Figure 178. HPLC spectrum of racemic **4m**

Chromatogram

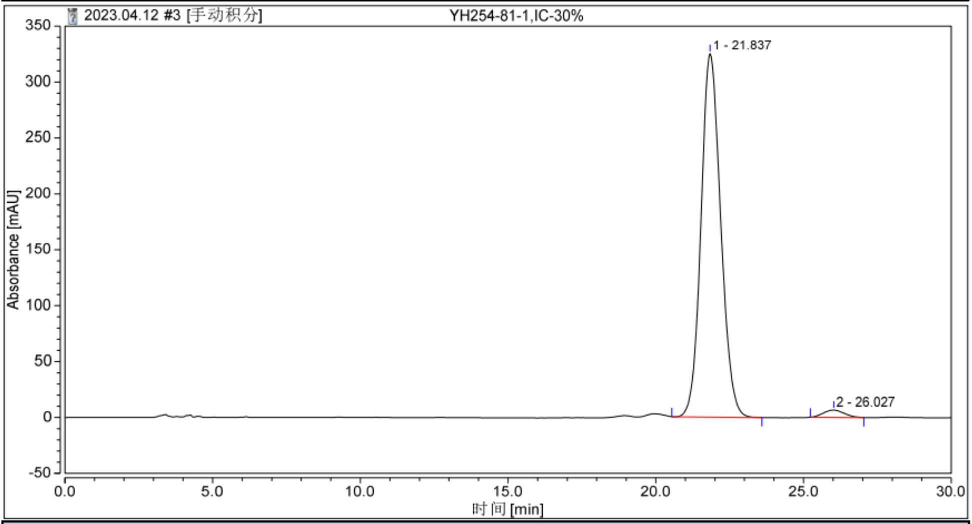

Integration Results

No.	Peak Name	Retention Time min	Area mAu*min	Height mAU	Relative Area %	Relative Height %	Amount n.a.
1		21.837	250.854	325.163	97.91	98.04	n.a.
2		26.027	5.359	6.493	2.09	1.96	n.a.
**Total:**			**256.213**	**331.656**	**100.00**	**100.00**	

Supplementary Figure 179. HPLC spectrum of **4m**

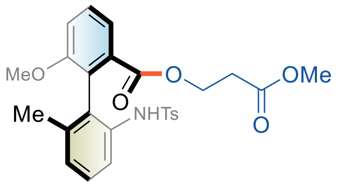

4n

Chromatogram

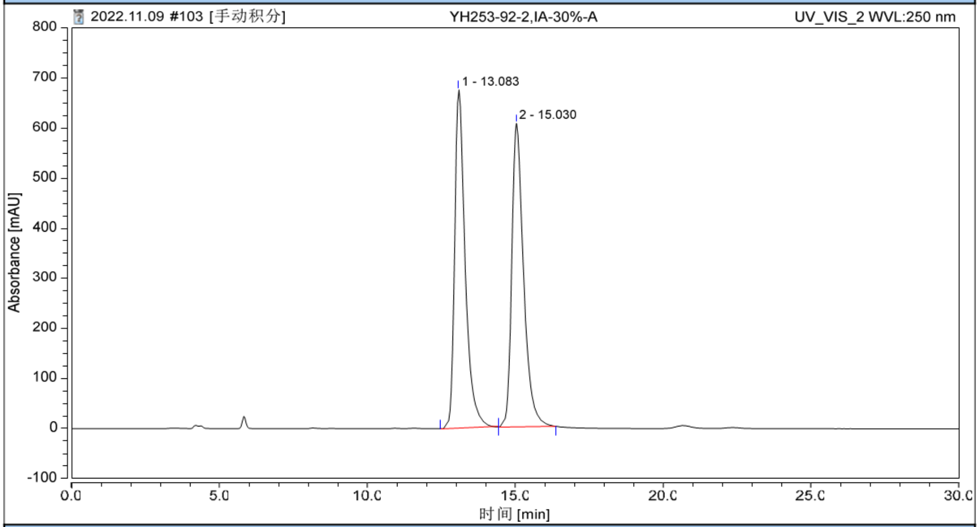

Integration Results

No.	Peak Name	Retention Time min	Area mAu*min	Height mAU	Relative Area %	Relative Height %	Amount n.a.
1		13.083	278.367	674.795	49.95	52.74	n.a.
2		15.030	278.881	604.785	50.05	47.26	n.a.
**Total:**			**557.248**	**1279.580**	**100.00**	**100.00**	

Supplementary Figure 180. HPLC spectrum of racemic **4n**

Chromatogram

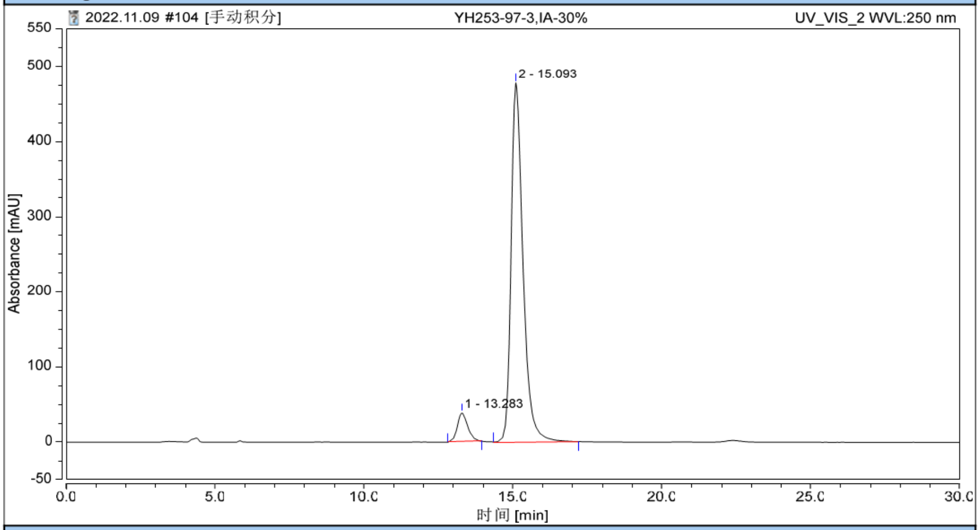

Integration Results

No.	Peak Name	Retention Time min	Area mAu*min	Height mAU	Relative Area %	Relative Height %	Amount n.a.
1		13.283	15.425	38.195	6.53	7.40	n.a.
2		15.093	220.958	477.744	93.47	92.60	n.a.
**Total:**			**236.384**	**515.939**	**100.00**	**100.00**	

Supplementary Figure 181. HPLC spectrum of **4n**

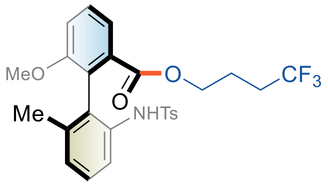

4o

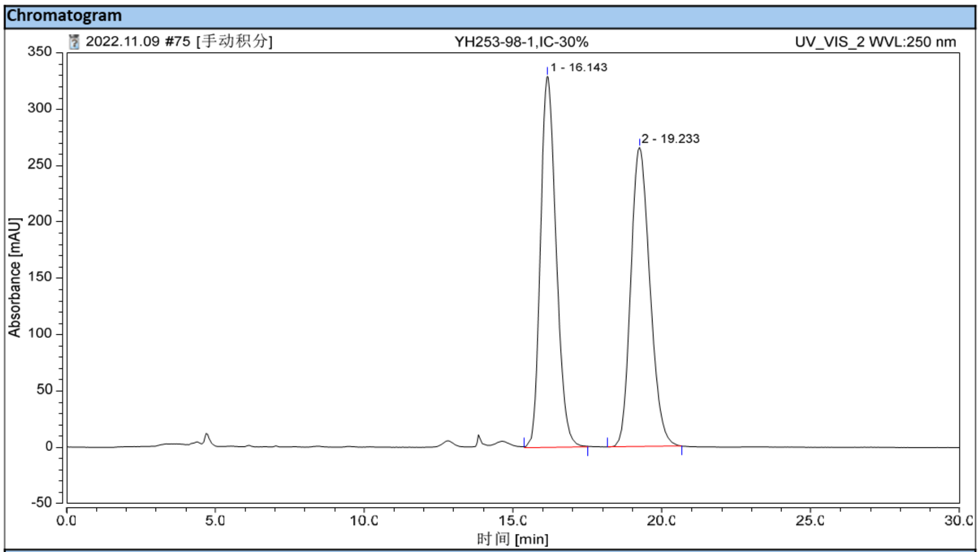

Integration Results

No.	Peak Name	Retention Time min	Area mAu*min	Height mAU	Relative Area %	Relative Height %	Amount n.a.
1		16.143	199.465	328.901	50.15	55.31	n.a.
2		19.233	198.249	265.741	49.85	44.69	n.a.
**Total:**			**397.714**	**594.642**	**100.00**	**100.00**	

Supplementary Figure 182. HPLC spectrum of racemic **4o**

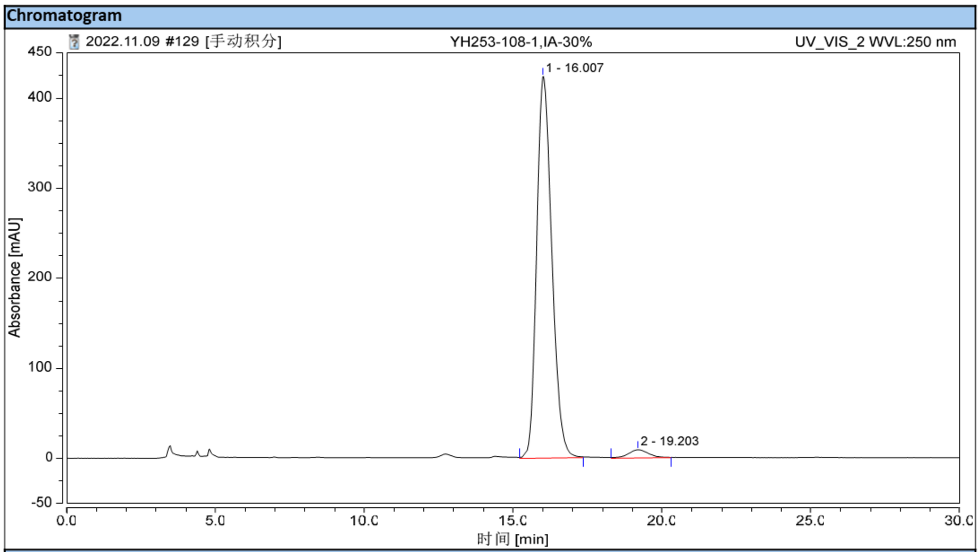

Integration Results

No.	Peak Name	Retention Time min	Area mAu*min	Height mAU	Relative Area %	Relative Height %	Amount n.a.
1		16.007	247.309	422.588	97.59	98.03	n.a.
2		19.203	6.108	8.483	2.41	1.97	n.a.
**Total:**			**253.417**	**431.071**	**100.00**	**100.00**	

Supplementary Figure 183. HPLC spectrum of **4o**

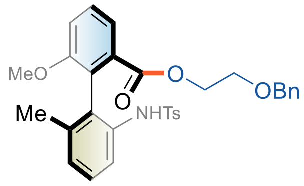

4p

Chromatogram

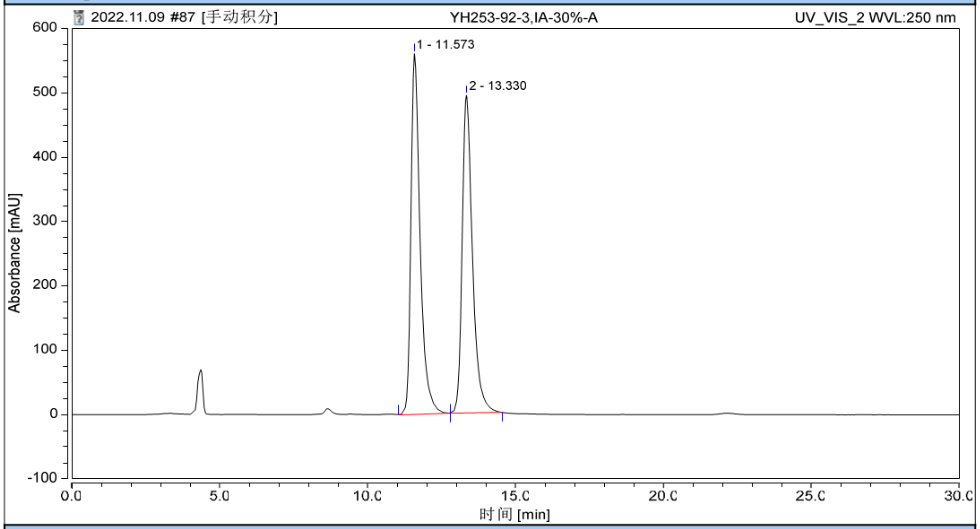

Integration Results

No.	Peak Name	Retention Time min	Area mAu*min	Height mAU	Relative Area %	Relative Height %	Amount n.a.
1		11.573	201.652	561.251	50.23	53.16	n.a.
2		13.330	199.790	494.504	49.77	46.84	n.a.
**Total:**			**401.442**	**1055.756**	**100.00**	**100.00**	

Supplementary Figure 184. HPLC spectrum of racemic **4p**

Chromatogram

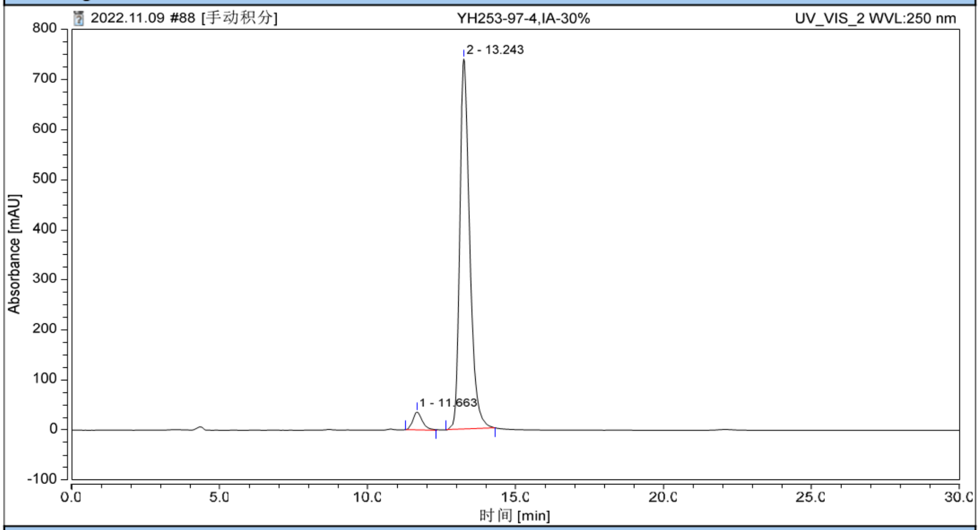

Integration Results

No.	Peak Name	Retention Time min	Area mAu*min	Height mAU	Relative Area %	Relative Height %	Amount n.a.
1		11.663	12.787	35.341	4.24	4.56	n.a.
2		13.243	288.771	739.924	95.76	95.44	n.a.
**Total:**			**301.558**	**775.265**	**100.00**	**100.00**	

Supplementary Figure 185. HPLC spectrum of **4p**

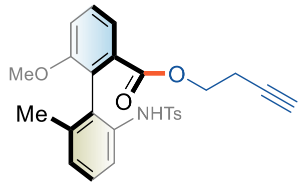

4q

Chromatogram

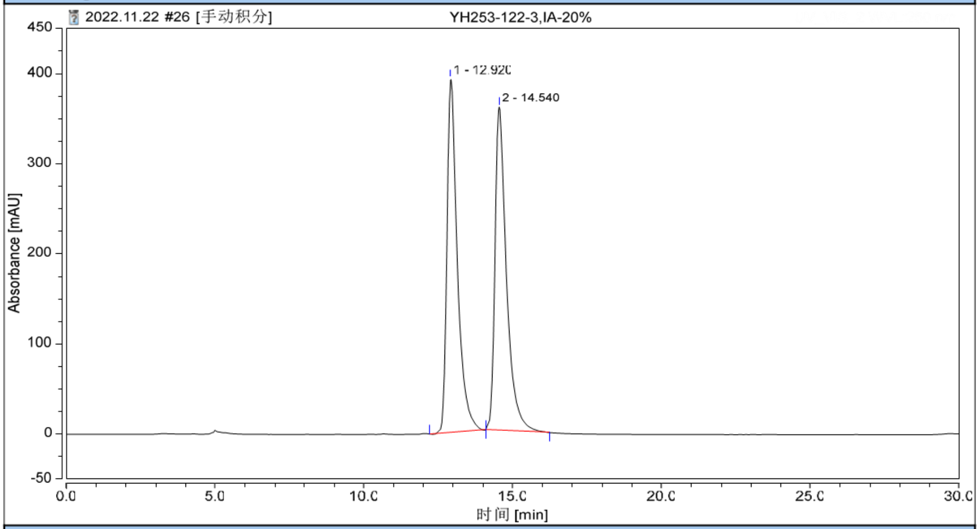

Integration Results

No.	Peak Name	Retention Time min	Area mAu*min	Height mAU	Relative Area %	Relative Height %	Amount n.a.
1		12.920	159.023	391.989	49.78	52.19	n.a.
2		14.540	160.419	359.107	50.22	47.81	n.a.
**Total:**			**319.442**	**751.096**	**100.00**	**100.00**	

Supplementary Figure 186. HPLC spectrum of racemic **4q**

Chromatogram

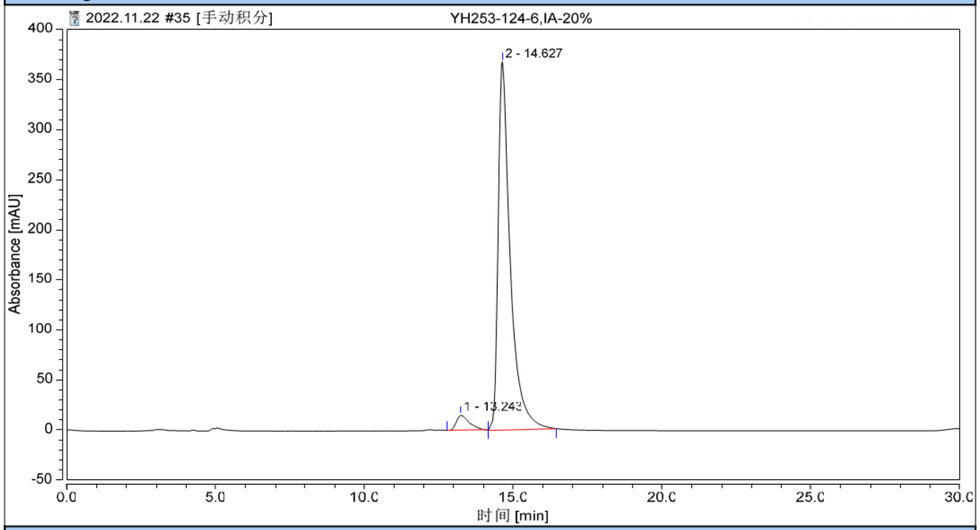

Integration Results

No.	Peak Name	Retention Time min	Area mAu*min	Height mAU	Relative Area %	Relative Height %	Amount n.a.
1		13.243	7.493	14.886	4.17	3.89	n.a.
2		14.627	172.134	367.382	95.83	96.11	n.a.
**Total:**			**179.627**	**382.267**	**100.00**	**100.00**	

Supplementary Figure 187. HPLC spectrum of **4q**

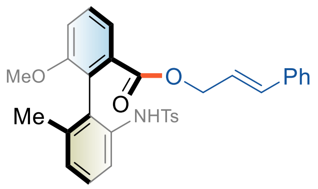

4r

Chromatogram

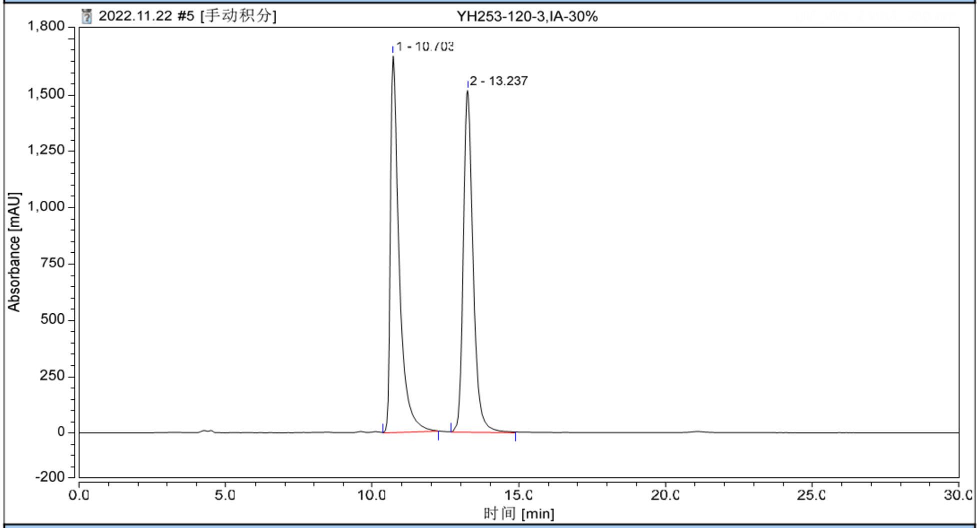

Integration Results

No.	Peak Name	Retention Time<br>min	Area<br>mAu*min	Height<br>mAU	Relative Area<br>%	Relative Height<br>%	Amount<br>n.a.
1		10.703	579.691	1672.318	49.79	52.40	n.a.
2		13.237	584.686	1519.280	50.21	47.60	n.a.
**Total:**			**1164.377**	**3191.598**	**100.00**	**100.00**	

Supplementary Figure 188. HPLC spectrum of racemic **4r**

Chromatogram

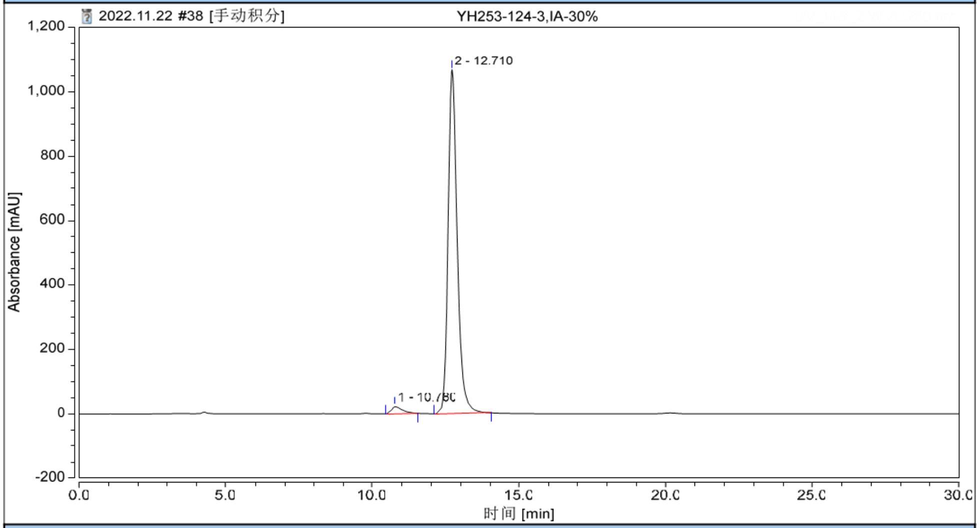

Integration Results

No.	Peak Name	Retention Time<br>min	Area<br>mAu*min	Height<br>mAU	Relative Area<br>%	Relative Height<br>%	Amount<br>n.a.
1		10.780	9.231	21.799	2.34	2.00	n.a.
2		12.710	385.625	1067.713	97.66	98.00	n.a.
**Total:**			**394.856**	**1089.512**	**100.00**	**100.00**	

Supplementary Figure 189. HPLC spectrum of **4r**

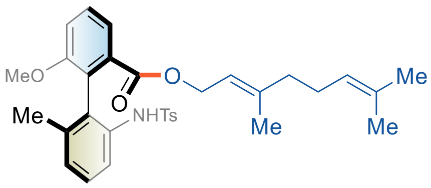

4s

Chromatogram

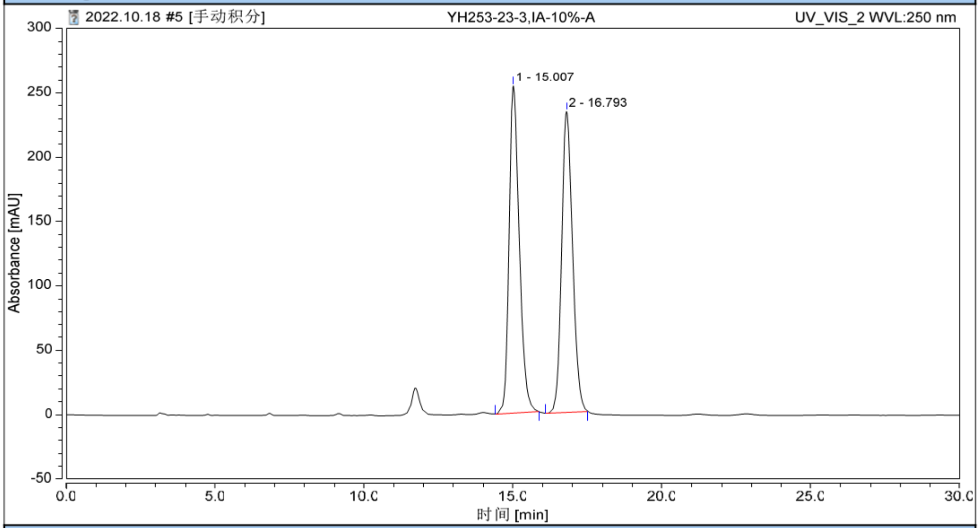

Integration Results

No.	Peak Name	Retention Time min	Area mAu*min	Height mAU	Relative Area %	Relative Height %	Amount n.a.
1		15.007	100.942	254.245	50.39	52.09	n.a.
2		16.793	99.363	233.832	49.61	47.91	n.a.
**Total:**			**200.306**	**488.077**	**100.00**	**100.00**	

Supplementary Figure 190. HPLC spectrum of racemic **4s**

Chromatogram

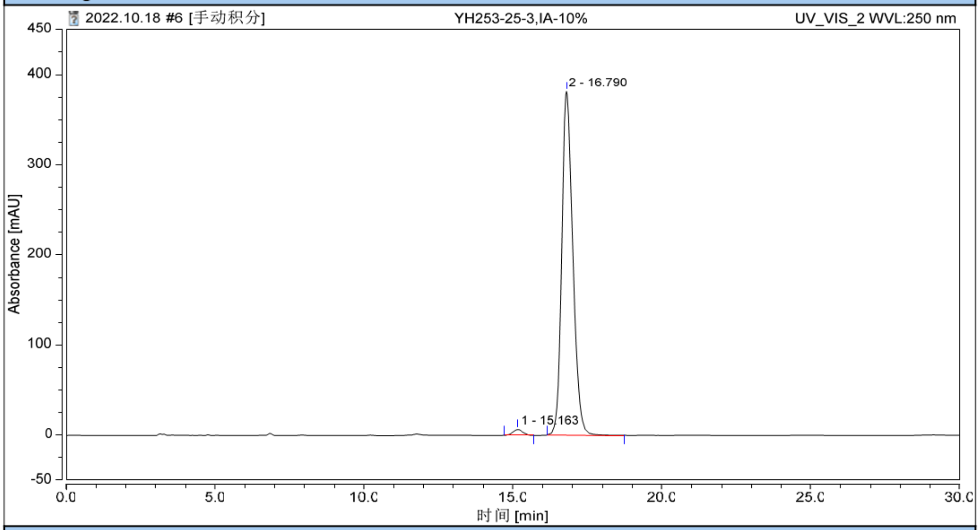

Integration Results

No.	Peak Name	Retention Time min	Area mAu*min	Height mAU	Relative Area %	Relative Height %	Amount n.a.
1		15.163	2.623	6.651	1.58	1.72	n.a.
2		16.790	163.898	381.059	98.42	98.28	n.a.
**Total:**			**166.521**	**387.710**	**100.00**	**100.00**	

Supplementary Figure 191. HPLC spectrum of **4s**

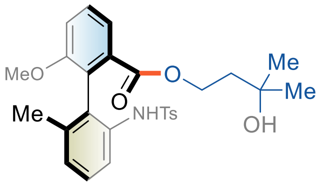

4t

Chromatogram

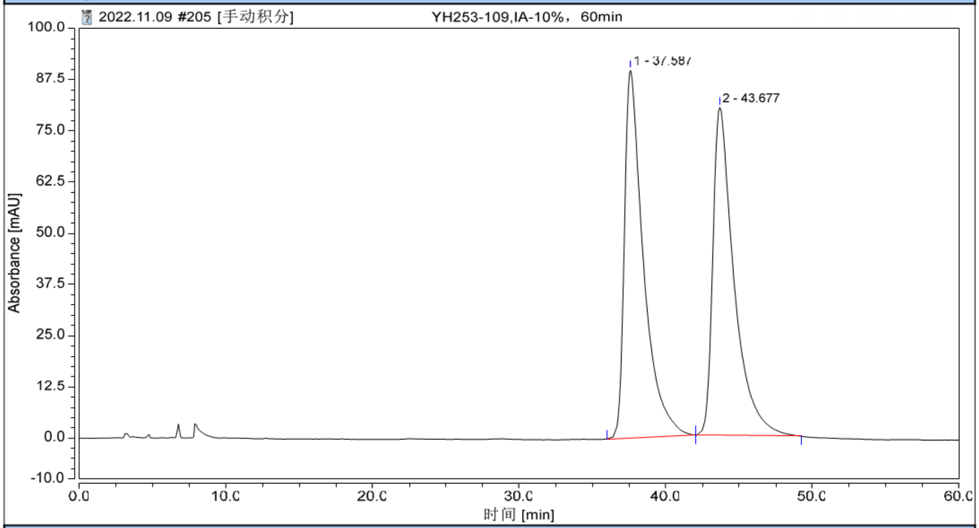

Integration Results

No.	Peak Name	Retention Time min	Area mAu*min	Height mAU	Relative Area %	Relative Height %	Amount n.a.
1		37.587	135.428	89.817	50.15	52.83	n.a.
2		43.677	134.631	80.179	49.85	47.17	n.a.
**Total:**			**270.058**	**169.996**	**100.00**	**100.00**	

Supplementary Figure 192. HPLC spectrum of racemic **4t**

Chromatogram

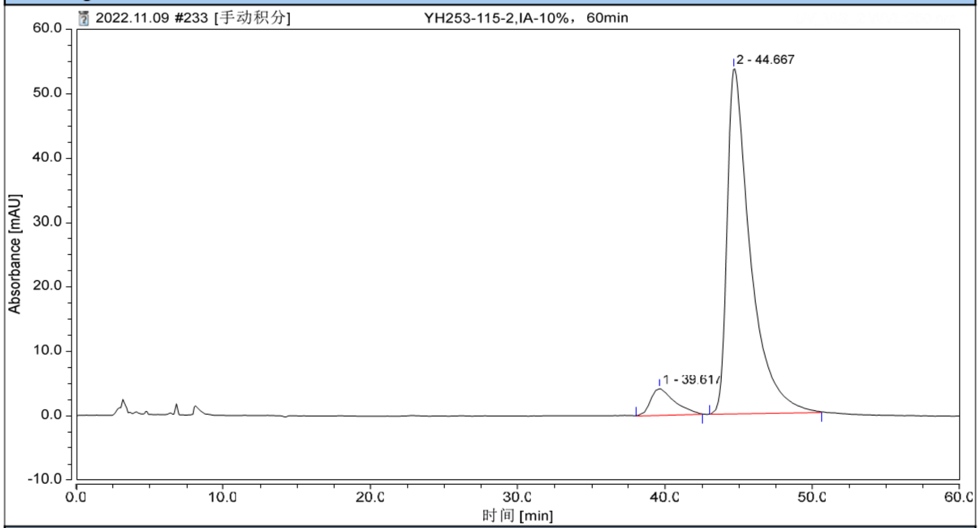

Integration Results

No.	Peak Name	Retention Time min	Area mAu*min	Height mAU	Relative Area %	Relative Height %	Amount n.a.
1		39.617	7.639	4.141	7.24	7.16	n.a.
2		44.667	97.929	53.697	92.76	92.84	n.a.
**Total:**			**105.568**	**57.837**	**100.00**	**100.00**	

Supplementary Figure 193. HPLC spectrum of **4t**

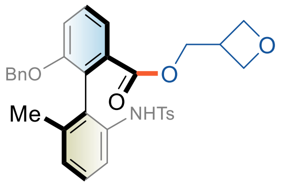

4u

Chromatogram

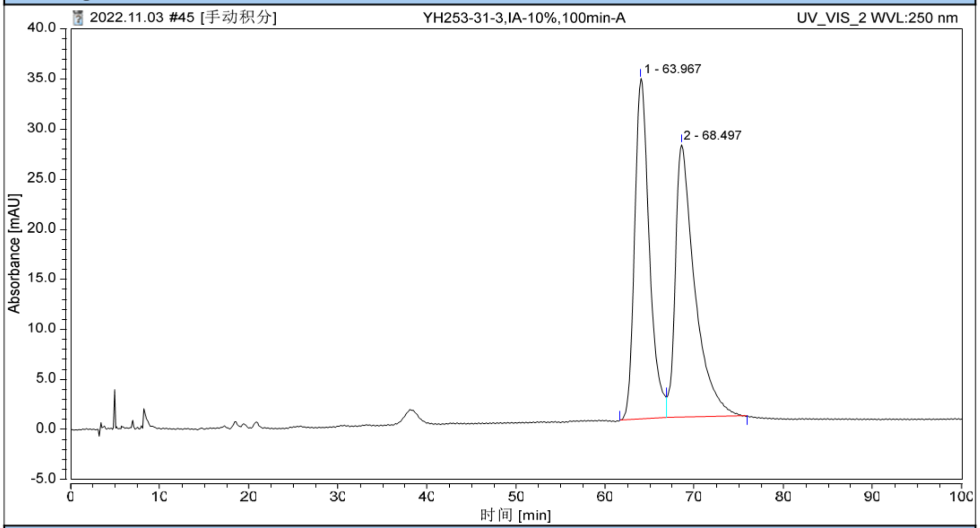

Integration Results

No.	Peak Name	Retention Time min	Area mAu*min	Height mAU	Relative Area %	Relative Height %	Amount n.a.
1		63.967	65.015	34.034	48.50	55.54	n.a.
2		68.497	69.046	27.246	51.50	44.46	n.a.
**Total:**			**134.061**	**61.281**	**100.00**	**100.00**	

Supplementary Figure 194. HPLC spectrum of racemic **4u**

Chromatogram

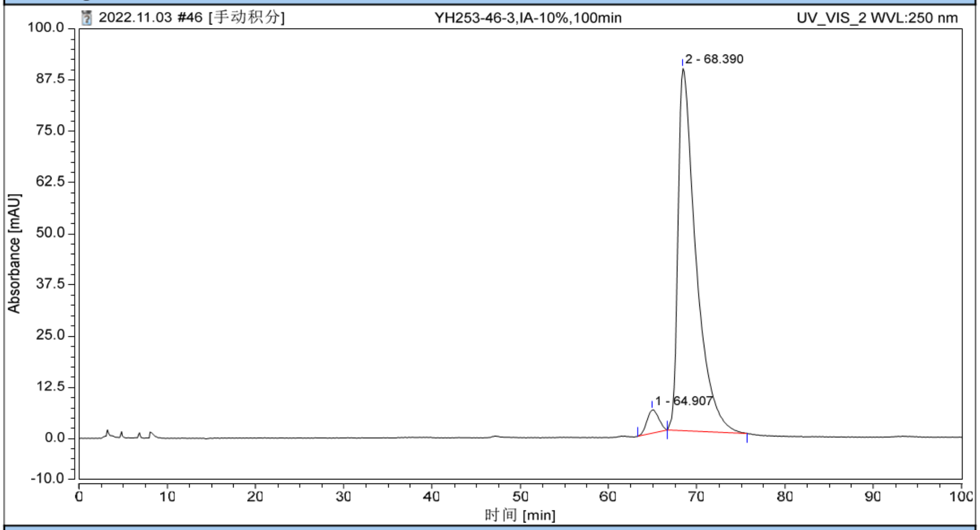

Integration Results

No.	Peak Name	Retention Time min	Area mAu*min	Height mAU	Relative Area %	Relative Height %	Amount n.a.
1		64.907	8.784	5.732	4.07	6.08	n.a.
2		68.390	206.959	88.478	95.93	93.92	n.a.
**Total:**			**215.742**	**94.210**	**100.00**	**100.00**	

Supplementary Figure 195. HPLC spectrum of **4u**

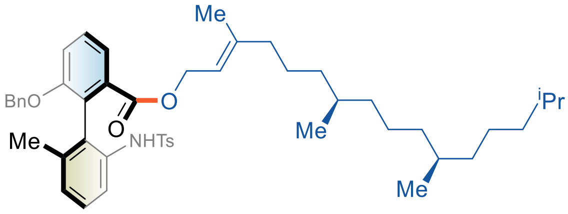

4v

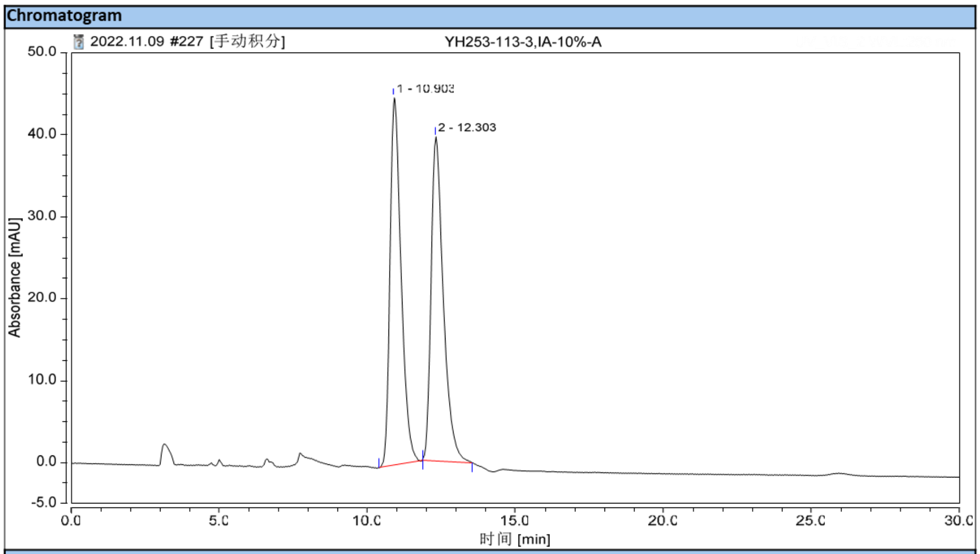

Integration Results

No.	Peak Name	Retention Time min	Area mAu*min	Height mAU	Relative Area %	Relative Height %	Amount n.a.
1		10.903	19.386	44.846	50.58	53.13	n.a.
2		12.303	18.940	39.567	49.42	46.87	n.a.
**Total:**			**38.326**	**84.413**	**100.00**	**100.00**	

Supplementary Figure 196. HPLC spectrum of racemic **4v**

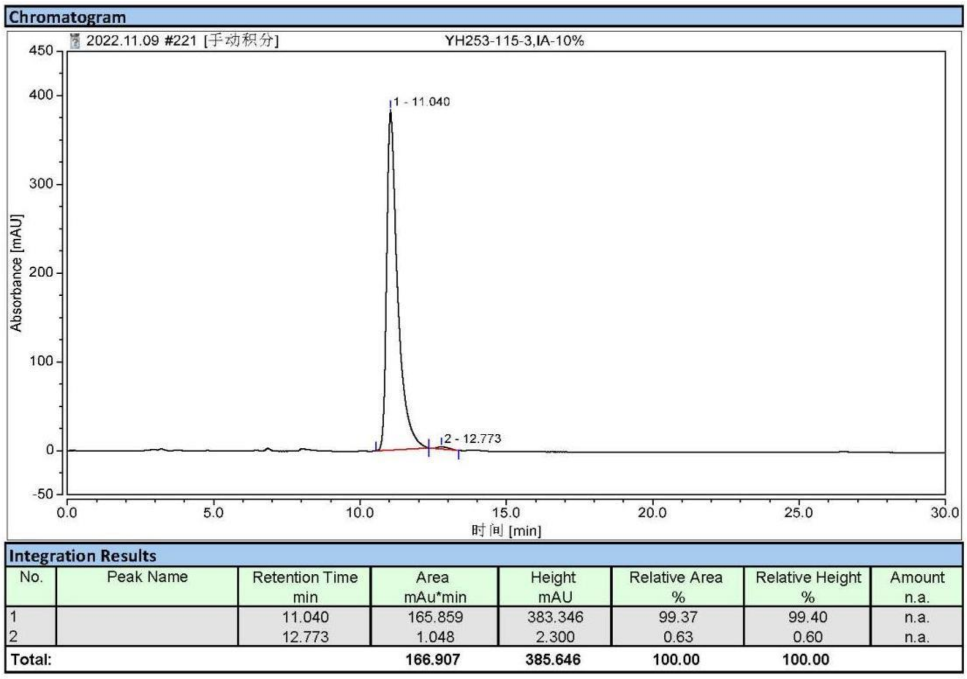

Integration Results

No.	Peak Name	Retention Time min	Area mAu*min	Height mAU	Relative Area %	Relative Height %	Amount n.a.
1		11.040	165.859	383.346	99.37	99.40	n.a.
2		12.773	1.048	2.300	0.63	0.60	n.a.
**Total:**			**166.907**	**385.646**	**100.00**	**100.00**	

Supplementary Figure 197. HPLC spectrum of **4v**

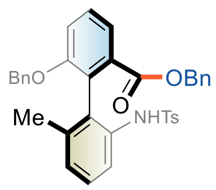

5a

Chromatogram

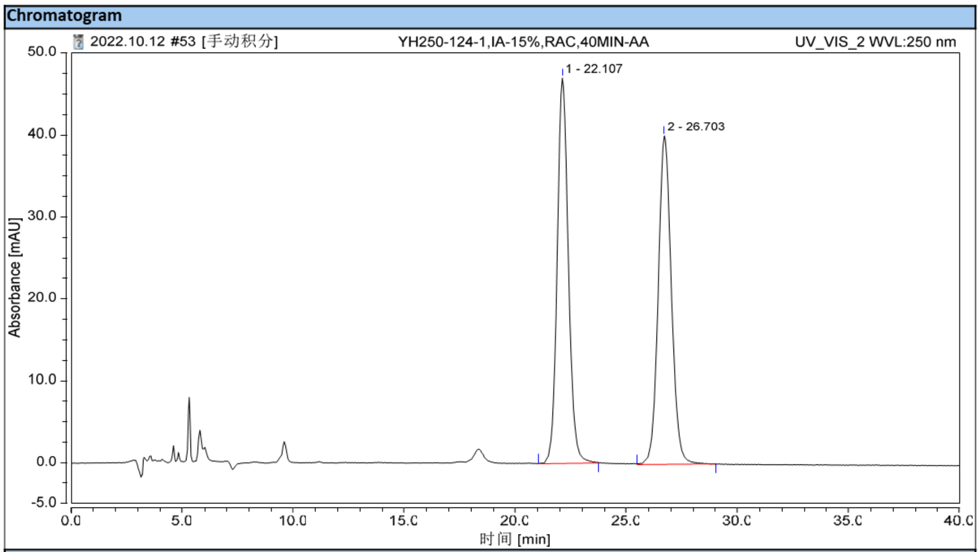

Integration Results

No.	Peak Name	Retention Time min	Area mAu*min	Height mAU	Relative Area %	Relative Height %	Amount n.a.
1		22.107	27.286	46.974	49.91	54.00	n.a.
2		26.703	27.381	40.021	50.09	46.00	n.a.
**Total:**			**54.667**	**86.995**	**100.00**	**100.00**	

Supplementary Figure 198. HPLC spectrum of racemic **5a**

Chromatogram

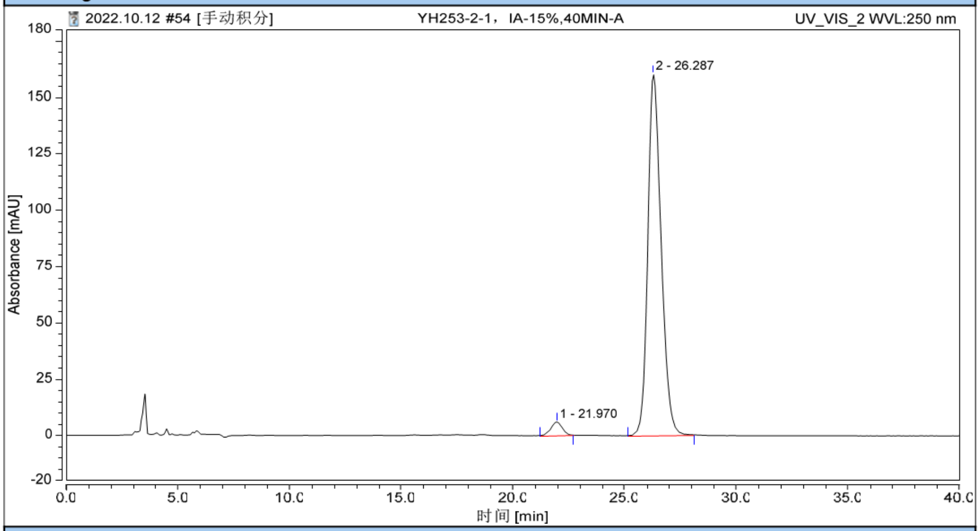

Integration Results

No.	Peak Name	Retention Time min	Area mAu*min	Height mAU	Relative Area %	Relative Height %	Amount n.a.
1		21.970	3.438	5.954	3.01	3.58	n.a.
2		26.287	110.909	160.461	96.99	96.42	n.a.
**Total:**			**114.347**	**166.414**	**100.00**	**100.00**	

Supplementary Figure 199. HPLC spectrum of **5a**

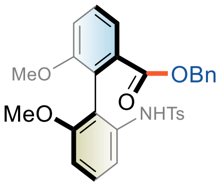

5b

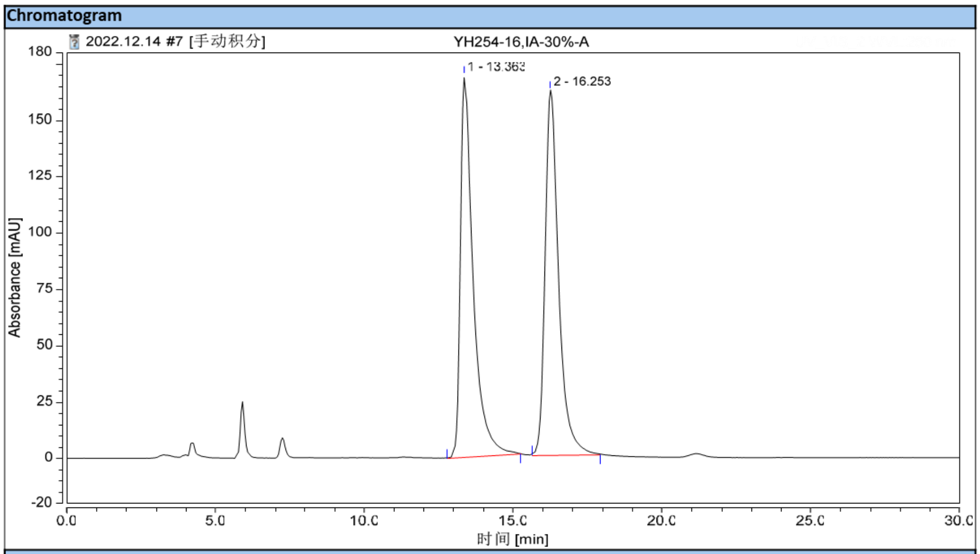

Integration Results

No.	Peak Name	Retention Time min	Area mAu*min	Height mAU	Relative Area %	Relative Height %	Amount n.a.
1		13.363	83.974	169.410	49.84	51.15	n.a.
2		16.253	84.530	161.792	50.16	48.85	n.a.
**Total:**			**168.504**	**331.202**	**100.00**	**100.00**	

Supplementary Figure 200. HPLC spectrum of racemic **5b**

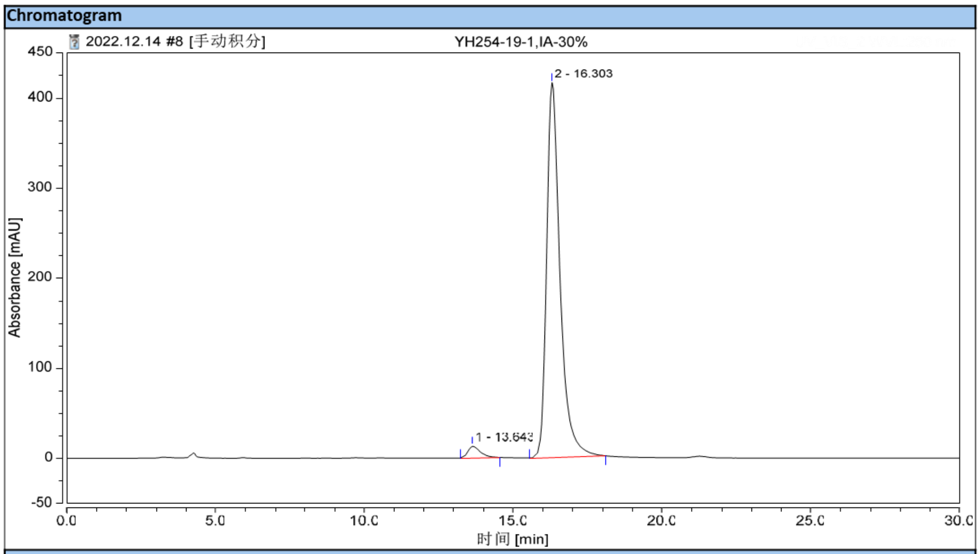

Integration Results

No.	Peak Name	Retention Time min	Area mAu*min	Height mAU	Relative Area %	Relative Height %	Amount n.a.
1		13.643	6.246	12.803	2.80	2.98	n.a.
2		16.303	216.914	416.188	97.20	97.02	n.a.
**Total:**			**223.160**	**428.991**	**100.00**	**100.00**	

Supplementary Figure 201. HPLC spectrum of **5b**

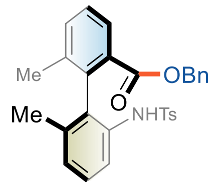

5c

Chromatogram

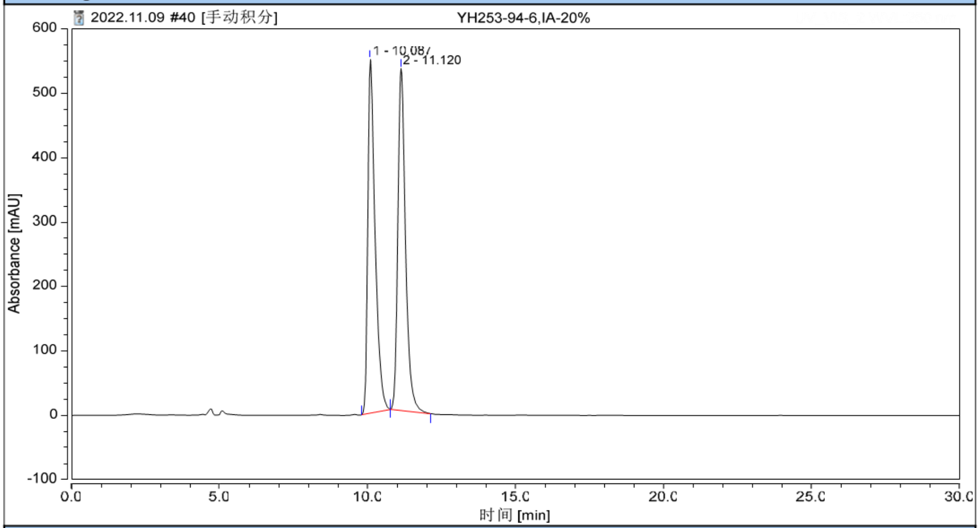

Integration Results

No.	Peak Name	Retention Time min	Area mAu*min	Height mAU	Relative Area %	Relative Height %	Amount n.a.
1		10.087	157.068	548.482	49.72	50.86	n.a.
2		11.120	158.809	530.007	50.28	49.14	n.a.
**Total:**			**315.877**	**1078.489**	**100.00**	**100.00**	

Supplementary Figure 202. HPLC spectrum of racemic **5c**

Chromatogram

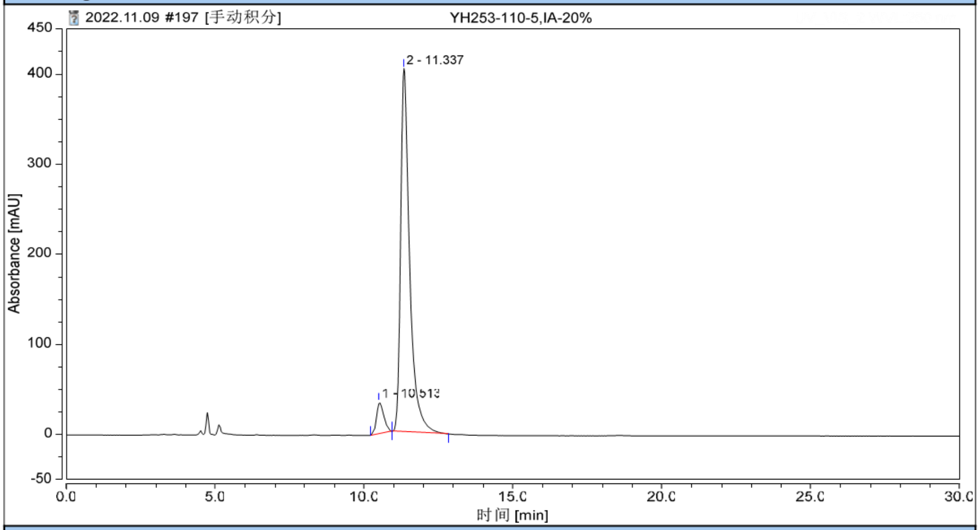

Integration Results

No.	Peak Name	Retention Time min	Area mAu*min	Height mAU	Relative Area %	Relative Height %	Amount n.a.
1		10.513	9.608	34.349	6.37	7.86	n.a.
2		11.337	141.172	402.553	93.63	92.14	n.a.
**Total:**			**150.780**	**436.902**	**100.00**	**100.00**	

Supplementary Figure 203. HPLC spectrum of **5c**

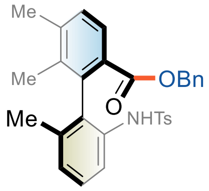

5d

Chromatogram

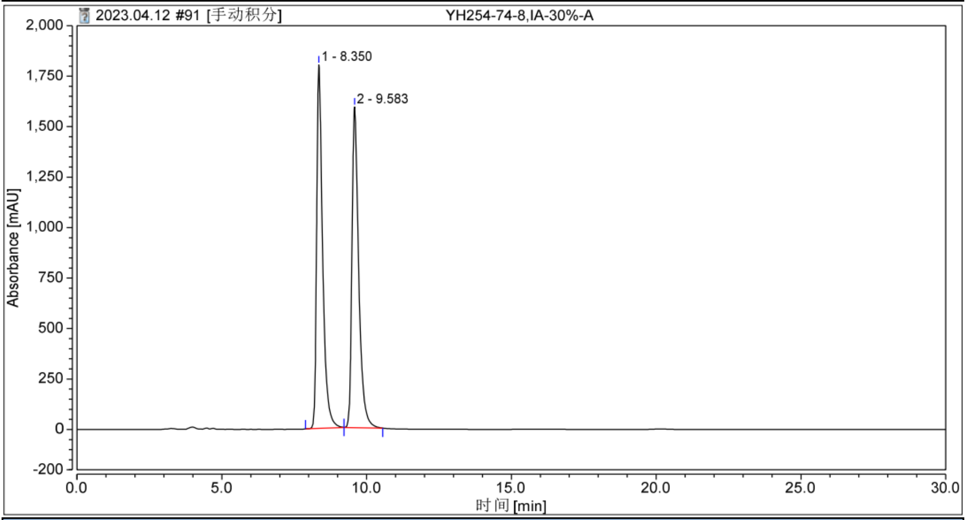

Integration Results

No.	Peak Name	Retention Time min	Area mAu*min	Height mAU	Relative Area %	Relative Height %	Amount n.a.
1		8.350	429.709	1800.891	50.07	53.12	n.a.
2		9.583	428.499	1589.154	49.93	46.88	n.a.
**Total:**			**858.208**	**3390.044**	**100.00**	**100.00**	

Supplementary Figure 204. HPLC spectrum of racemic **5d**

Chromatogram

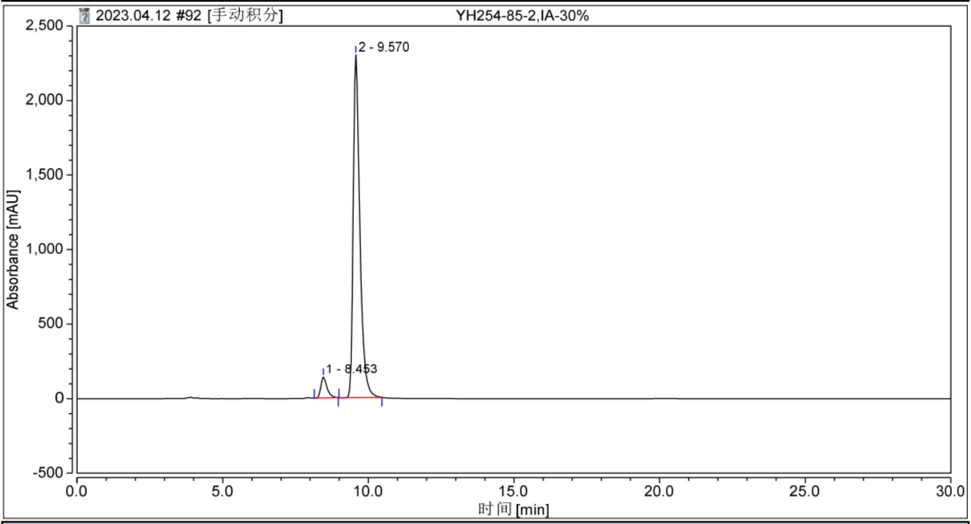

Integration Results

No.	Peak Name	Retention Time min	Area mAu*min	Height mAU	Relative Area %	Relative Height %	Amount n.a.
1		8.453	34.449	139.704	5.37	5.73	n.a.
2		9.570	607.560	2298.035	94.63	94.27	n.a.
**Total:**			**642.009**	**2437.740**	**100.00**	**100.00**	

Supplementary Figure 205. HPLC spectrum of racemic **5d**

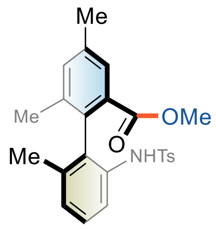

5e

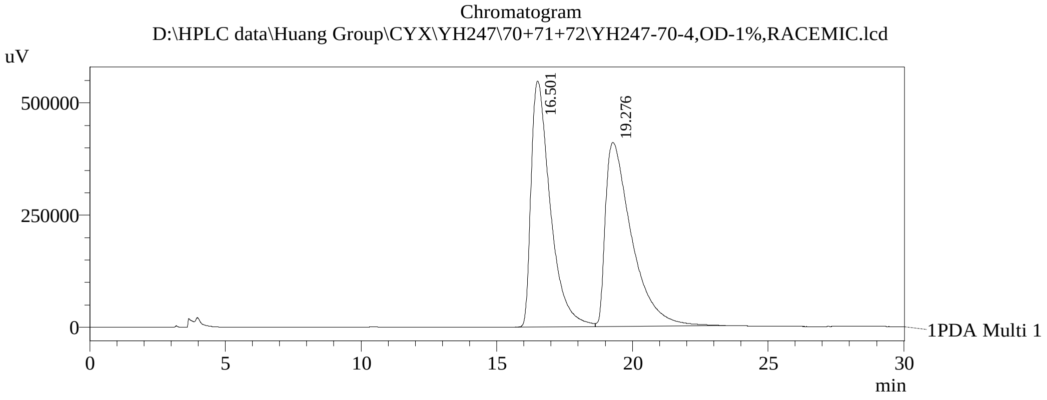

1 PDA Multi 1 / 210nm 4nm

PeakTable

Ch1 210nm 4nm

Peak#	Ret. Time	Area	Height	Area %	Height %
1	16.501	27033041	548106	49.458	57.201
2	19.276	27625438	410100	50.542	42.799
Total		54658479	958205	100.000	100.000

Supplementary Figure 206. HPLC spectrum of racemic **5e**

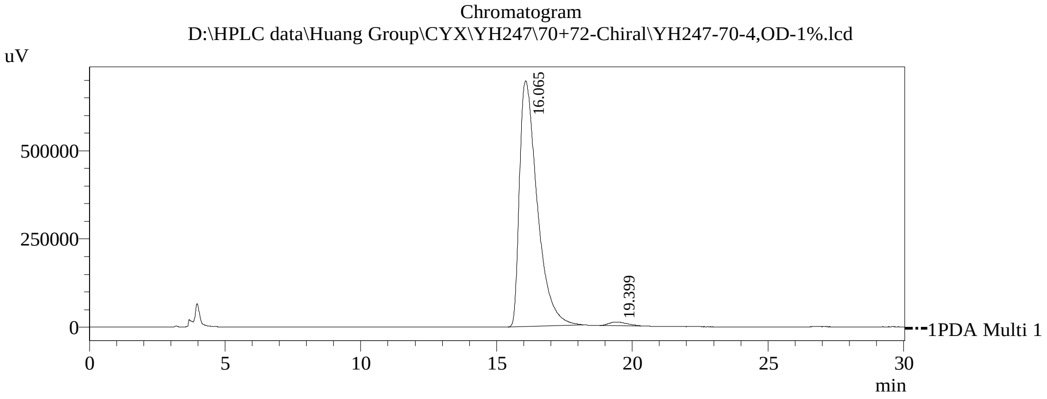

1 PDA Multi 1 / 210nm 4nm

PeakTable

Ch1 210nm 4nm

Peak#	Ret. Time	Area	Height	Area %	Height %
1	16.065	31647957	696301	98.557	98.538
2	19.399	463502	10330	1.443	1.462
Total		32111459	706631	100.000	100.000

Supplementary Figure 207. HPLC spectrum of **5e**

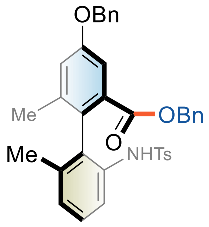

5f

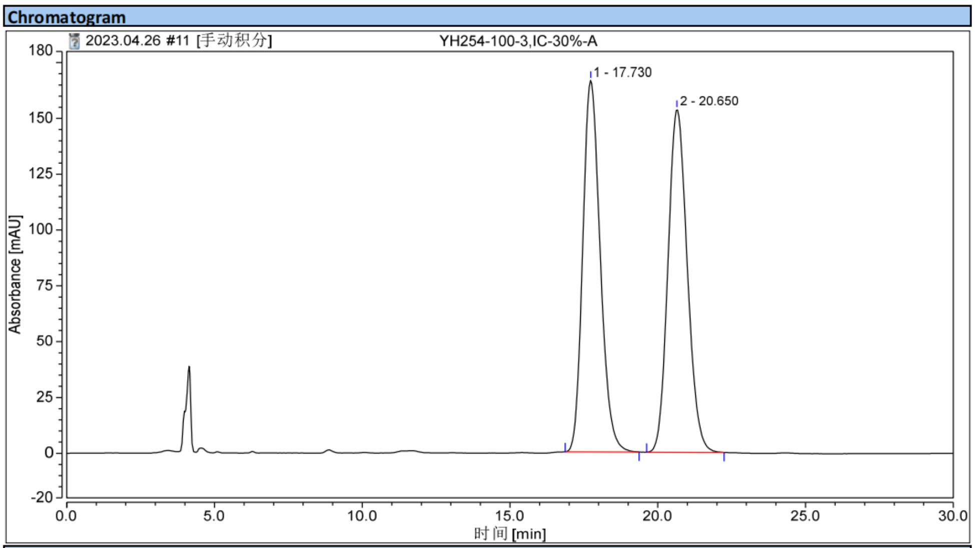

Integration Results

No.	Peak Name	Retention Time min	Area mAu*min	Height mAU	Relative Area %	Relative Height %	Amount n.a.
1		17.730	113.806	166.526	49.50	52.00	n.a.
2		20.650	116.092	153.694	50.50	48.00	n.a.
**Total:**			**229.898**	**320.220**	**100.00**	**100.00**	

Supplementary Figure 208. HPLC spectrum of racemic **5f**

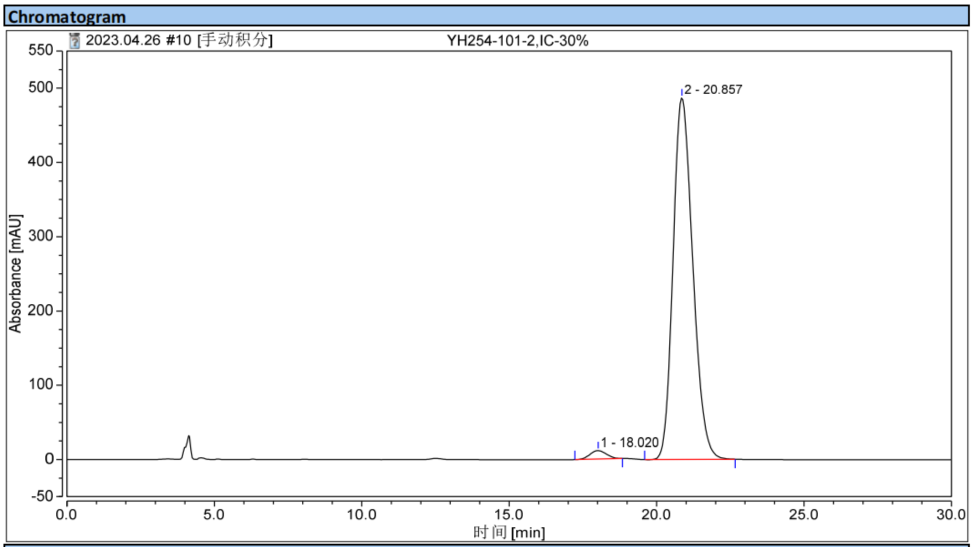

Integration Results

No.	Peak Name	Retention Time min	Area mAu*min	Height mAU	Relative Area %	Relative Height %	Amount n.a.
1		18.020	7.340	11.387	1.90	2.29	n.a.
2		20.857	377.991	486.852	98.10	97.71	n.a.
**Total:**			**385.331**	**498.239**	**100.00**	**100.00**	

Supplementary Figure 209. HPLC spectrum of **5f**

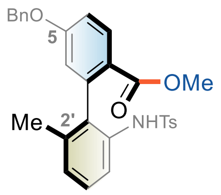

5g

Chromatogram

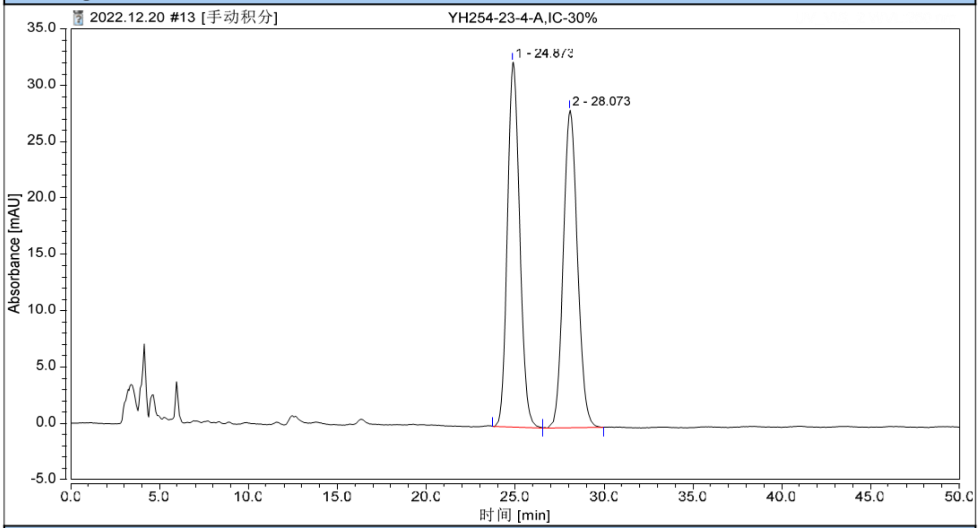

Integration Results

No.	Peak Name	Retention Time min	Area mAu*min	Height mAU	Relative Area %	Relative Height %	Amount n.a.
1		24.873	25.921	32.365	50.01	53.47	n.a.
2		28.073	25.911	28.162	49.99	46.53	n.a.
Total:			51.833	60.527	100.00	100.00	

Supplementary Figure 210. HPLC spectrum of racemic **5g**

Chromatogram

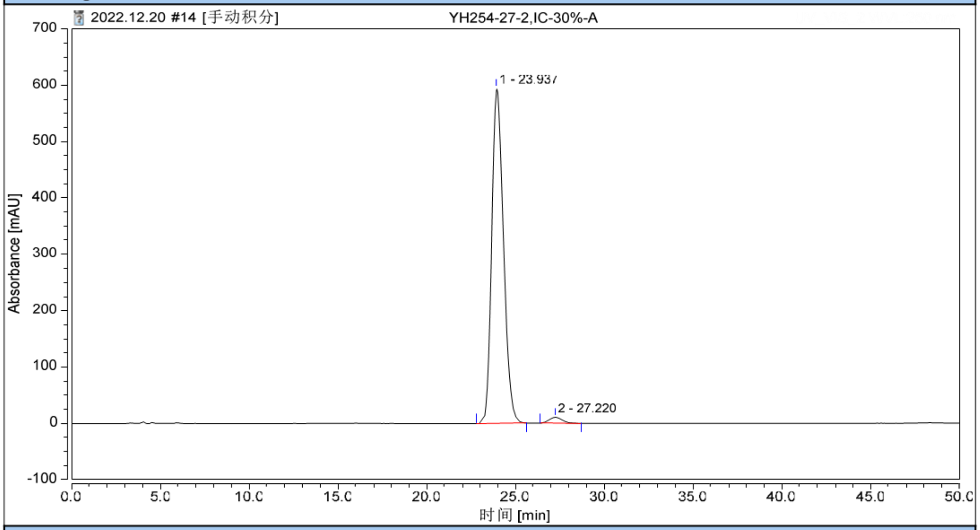

Integration Results

No.	Peak Name	Retention Time min	Area mAu*min	Height mAU	Relative Area %	Relative Height %	Amount n.a.
1		23.937	454.398	593.317	98.05	98.27	n.a.
2		27.220	9.042	10.458	1.95	1.73	n.a.
Total:			463.439	603.775	100.00	100.00	

Supplementary Figure 211. HPLC spectrum of **5g**

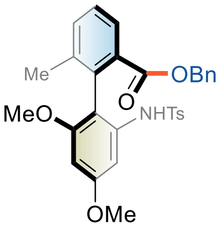

5h

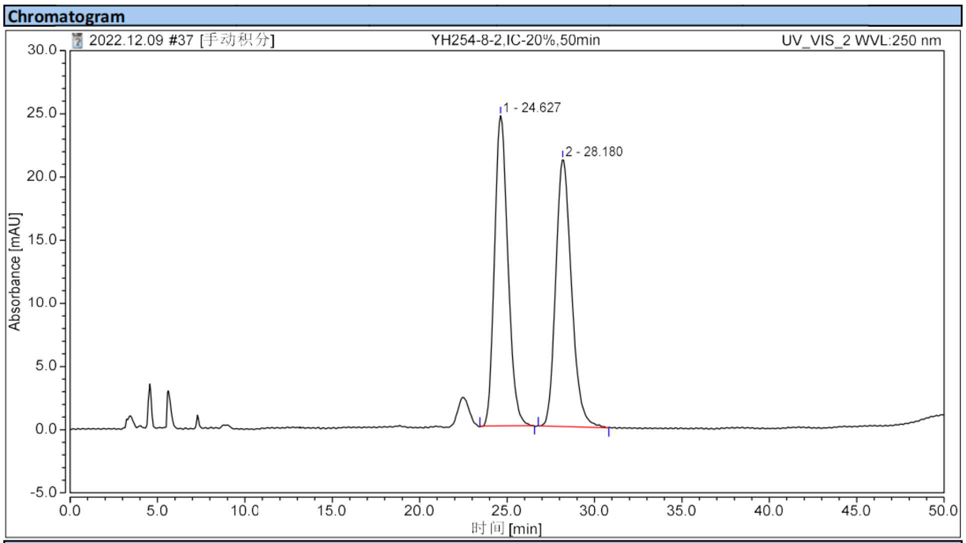

Integration Results

No.	Peak Name	Retention Time min	Area mAu*min	Height mAU	Relative Area %	Relative Height %	Amount n.a.
1		24.627	21.840	24.563	49.61	53.75	n.a.
2		28.180	22.186	21.133	50.39	46.25	n.a.
Total:			44.026	45.696	100.00	100.00	

Supplementary Figure 212. HPLC spectrum of racemic **5h**

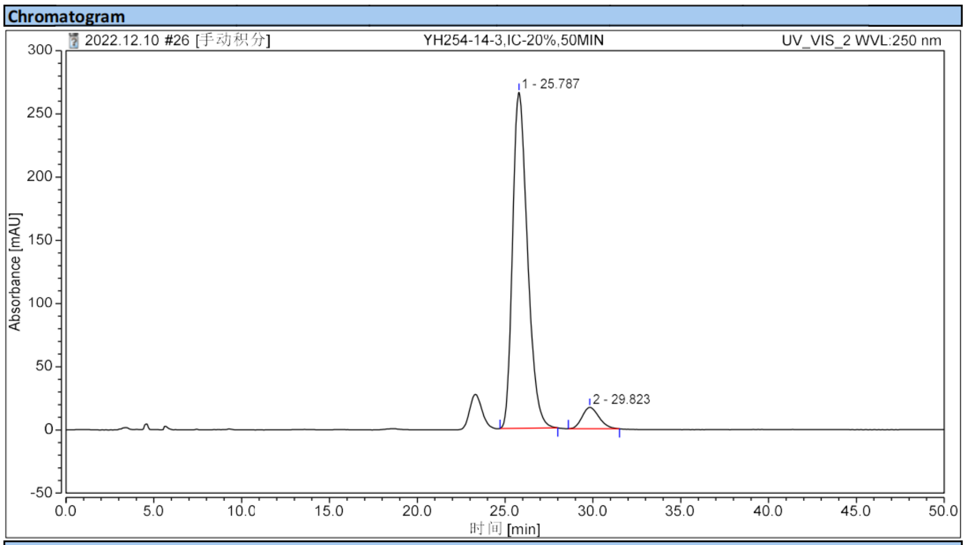

Integration Results

No.	Peak Name	Retention Time min	Area mAu*min	Height mAU	Relative Area %	Relative Height %	Amount n.a.
1		25.787	261.788	265.805	93.28	94.01	n.a.
2		29.823	18.871	16.948	6.72	5.99	n.a.
Total:			280.659	282.753	100.00	100.00	

Supplementary Figure 213. HPLC spectrum of **5h**

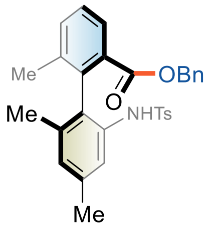

5i

Chromatogram

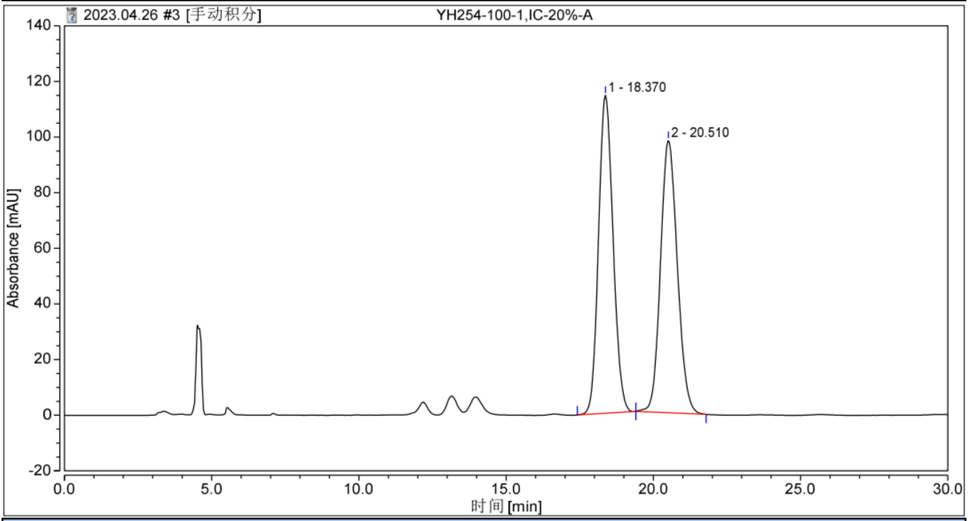

Integration Results

No.	Peak Name	Retention Time min	Area mAu*min	Height mAU	Relative Area %	Relative Height %	Amount n.a.
1		18.370	65.269	114.351	50.19	53.88	n.a.
2		20.510	64.785	97.895	49.81	46.12	n.a.
**Total:**			**130.054**	**212.245**	**100.00**	**100.00**	

Supplementary Figure 214. HPLC spectrum of racemic **5i**

Chromatogram

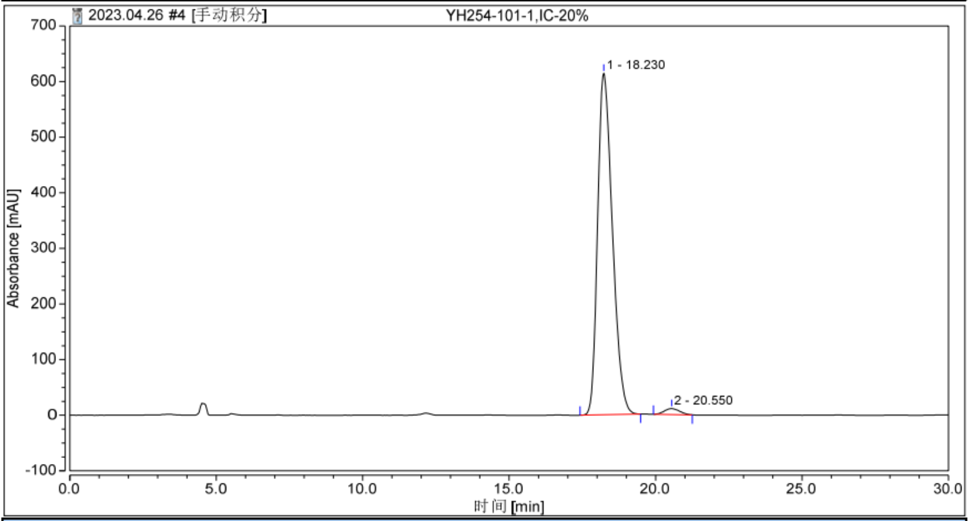

Integration Results

No.	Peak Name	Retention Time min	Area mAu*min	Height mAU	Relative Area %	Relative Height %	Amount n.a.
1		18.230	358.947	614.303	98.21	98.28	n.a.
2		20.550	6.555	10.772	1.79	1.72	n.a.
**Total:**			**365.502**	**625.076**	**100.00**	**100.00**	

Supplementary Figure 215. HPLC spectrum of **5i**

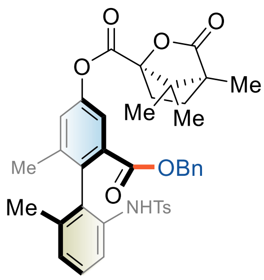

5j

Chromatogram

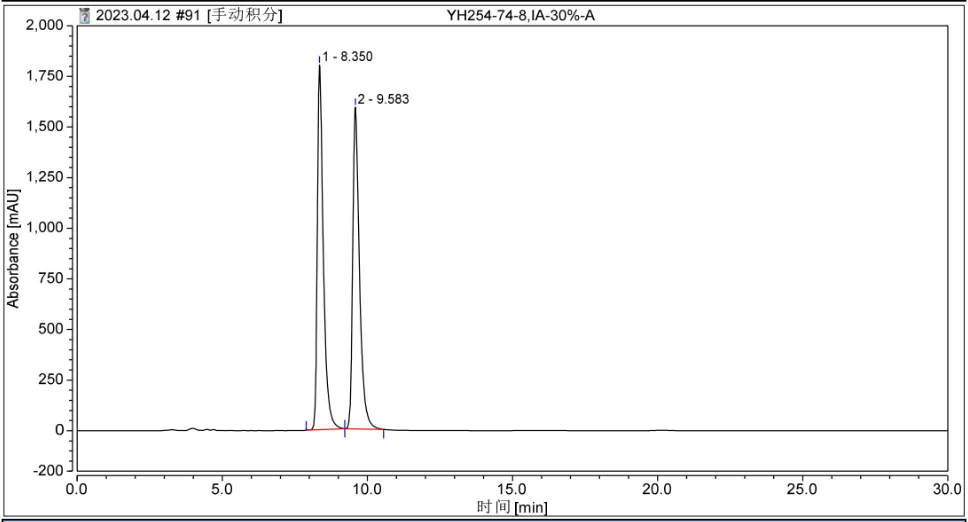

Integration Results

No.	Peak Name	Retention Time<br>min	Area<br>mAu*min	Height<br>mAU	Relative Area<br>%	Relative Height<br>%	Amount<br>n.a.
1		8.350	429.709	1800.891	50.07	53.12	n.a.
2		9.583	428.499	1589.154	49.93	46.88	n.a.
**Total:**			**858.208**	**3390.044**	**100.00**	**100.00**	

Supplementary Figure 216. HPLC spectrum of racemic **5j**

Chromatogram

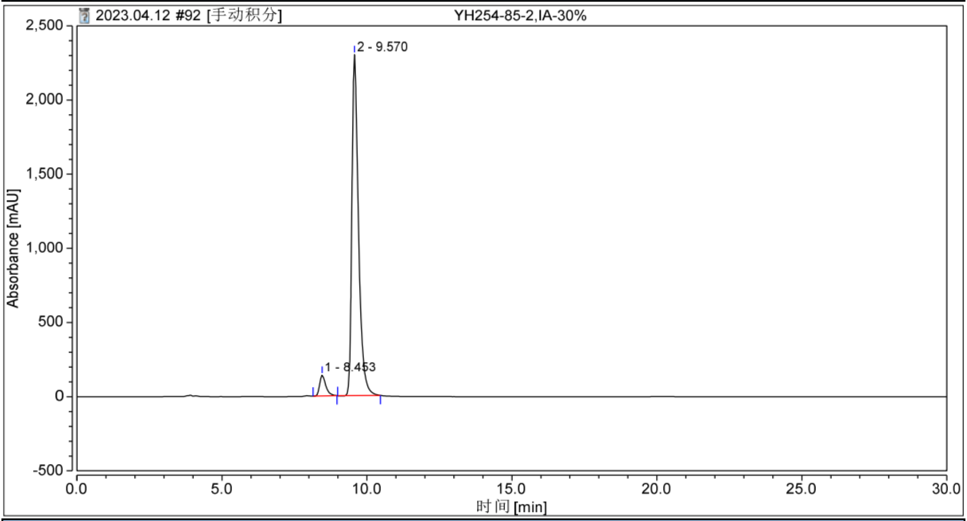

Integration Results

No.	Peak Name	Retention Time<br>min	Area<br>mAu*min	Height<br>mAU	Relative Area<br>%	Relative Height<br>%	Amount<br>n.a.
1		8.453	34.449	139.704	5.37	5.73	n.a.
2		9.570	607.560	2298.035	94.63	94.27	n.a.
**Total:**			**642.009**	**2437.740**	**100.00**	**100.00**	

Supplementary Figure 217. HPLC spectrum of **5j**

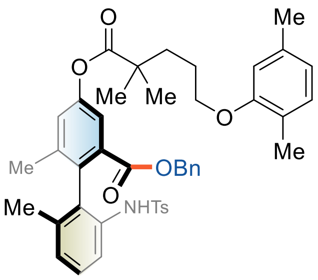

5k

Chromatogram

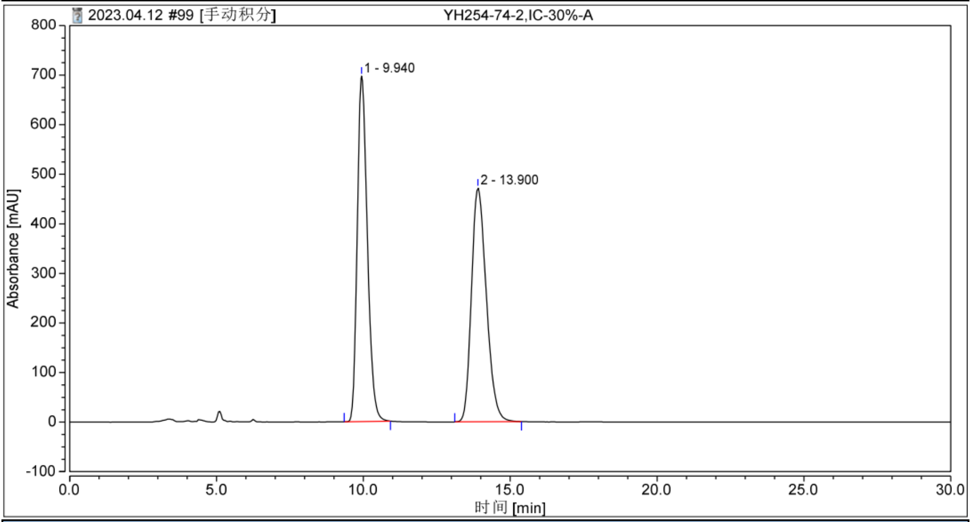

Integration Results

No.	Peak Name	Retention Time min	Area mAu*min	Height mAU	Relative Area %	Relative Height %	Amount n.a.
1		9.940	277.419	697.133	50.00	59.64	n.a.
2		13.900	277.403	471.804	50.00	40.36	n.a.
**Total:**			**554.822**	**1168.937**	**100.00**	**100.00**	

Supplementary Figure 218. HPLC spectrum of racemic **5k**

Chromatogram

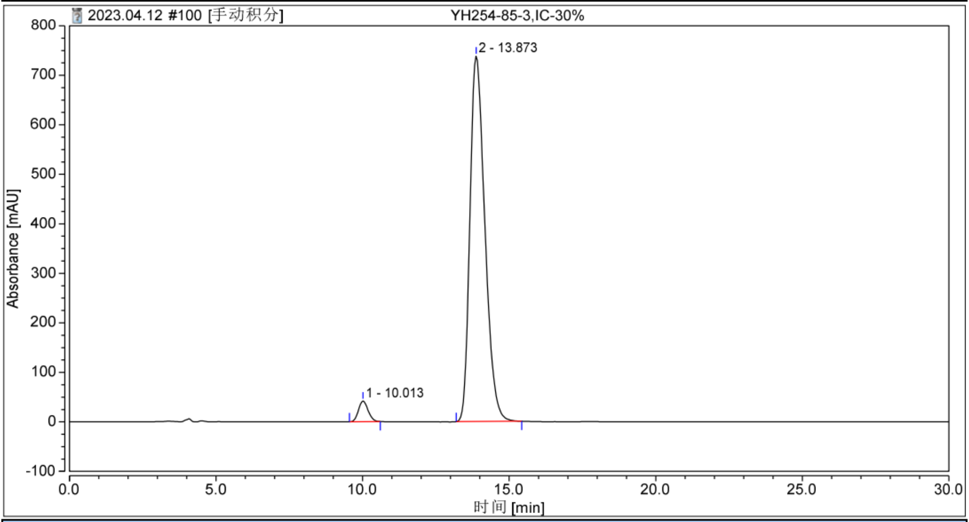

Integration Results

No.	Peak Name	Retention Time min	Area mAu*min	Height mAU	Relative Area %	Relative Height %	Amount n.a.
1		10.013	15.926	41.777	3.52	5.35	n.a.
2		13.873	436.206	738.405	96.48	94.65	n.a.
**Total:**			**452.132**	**780.183**	**100.00**	**100.00**	

Supplementary Figure 219. HPLC spectrum of **5k**

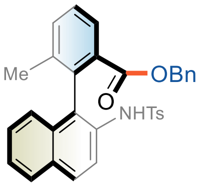

5l

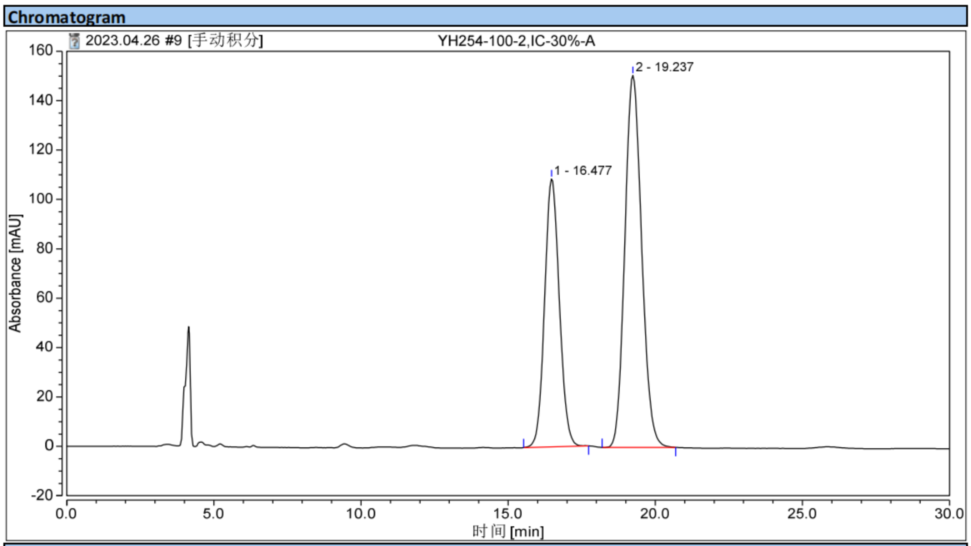

Integration Results

No.	Peak Name	Retention Time min	Area mAu*min	Height mAU	Relative Area %	Relative Height %	Amount n.a.
1		16.477	62.935	108.594	38.65	41.88	n.a.
2		19.237	99.889	150.686	61.35	58.12	n.a.
**Total:**			**162.823**	**259.280**	**100.00**	**100.00**	

Supplementary Figure 220. HPLC spectrum of racemic **5l**

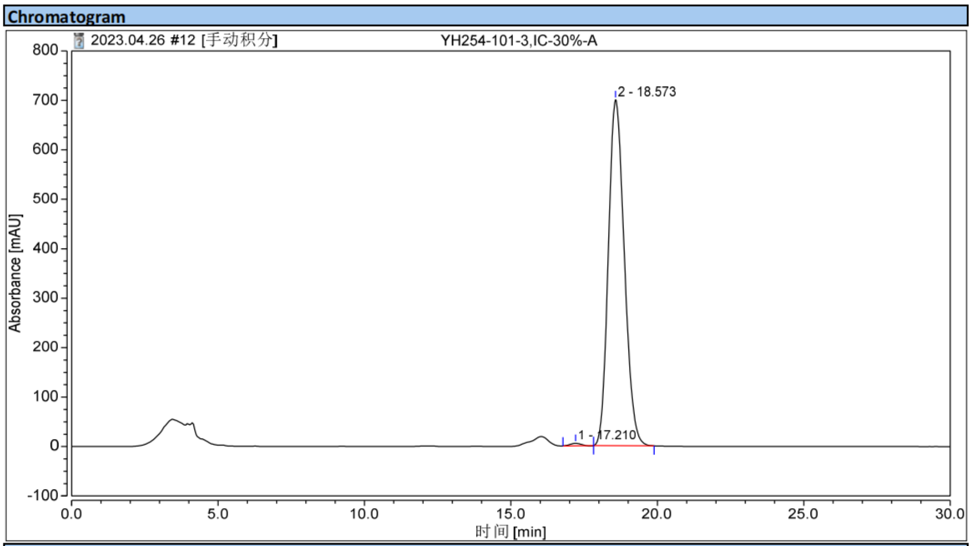

Integration Results

No.	Peak Name	Retention Time min	Area mAu*min	Height mAU	Relative Area %	Relative Height %	Amount n.a.
1		17.210	2.019	5.061	0.45	0.72	n.a.
2		18.573	442.326	699.544	99.55	99.28	n.a.
**Total:**			**444.345**	**704.605**	**100.00**	**100.00**	

Supplementary Figure 221. HPLC spectrum of **5l**

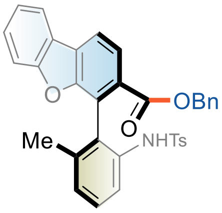

5m

Chromatogram

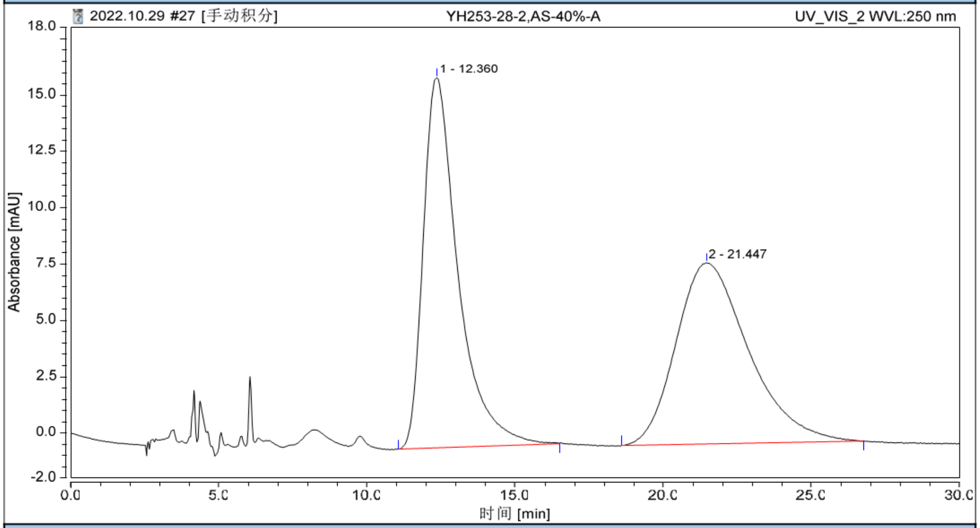

Integration Results

No.	Peak Name	Retention Time<br>min	Area<br>mAu*min	Height<br>mAU	Relative Area<br>%	Relative Height<br>%	Amount<br>n.a.
1		12.360	22.283	16.433	49.61	67.20	n.a.
2		21.447	22.633	8.020	50.39	32.80	n.a.
**Total:**			**44.916**	**24.454**	**100.00**	**100.00**	

Supplementary Figure 222. HPLC spectrum of racemic **5m**

Chromatogram

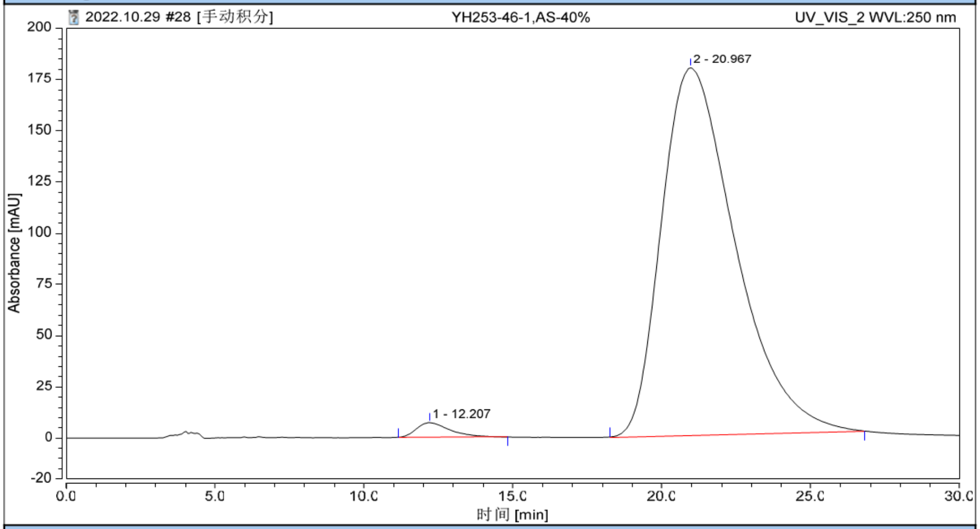

Integration Results

No.	Peak Name	Retention Time<br>min	Area<br>mAu*min	Height<br>mAU	Relative Area<br>%	Relative Height<br>%	Amount<br>n.a.
1		12.207	9.345	7.099	1.78	3.81	n.a.
2		20.967	516.003	179.332	98.22	96.19	n.a.
**Total:**			**525.348**	**186.431**	**100.00**	**100.00**	

Supplementary Figure 223. HPLC spectrum of **5m**

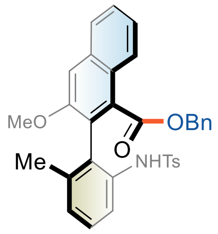

5n

Chromatogram

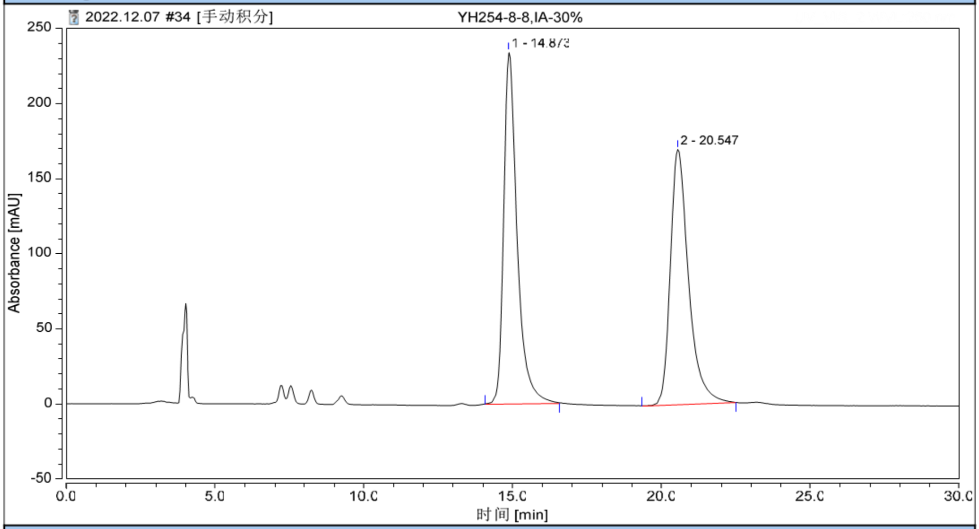

Integration Results

No.	Peak Name	Retention Time min	Area mAu*min	Height mAU	Relative Area %	Relative Height %	Amount n.a.
1		14.873	121.886	233.901	50.34	57.88	n.a.
2		20.547	120.230	170.202	49.66	42.12	n.a.
**Total:**			**242.116**	**404.103**	**100.00**	**100.00**	

Supplementary Figure 224. HPLC spectrum of racemic **5n**

Chromatogram

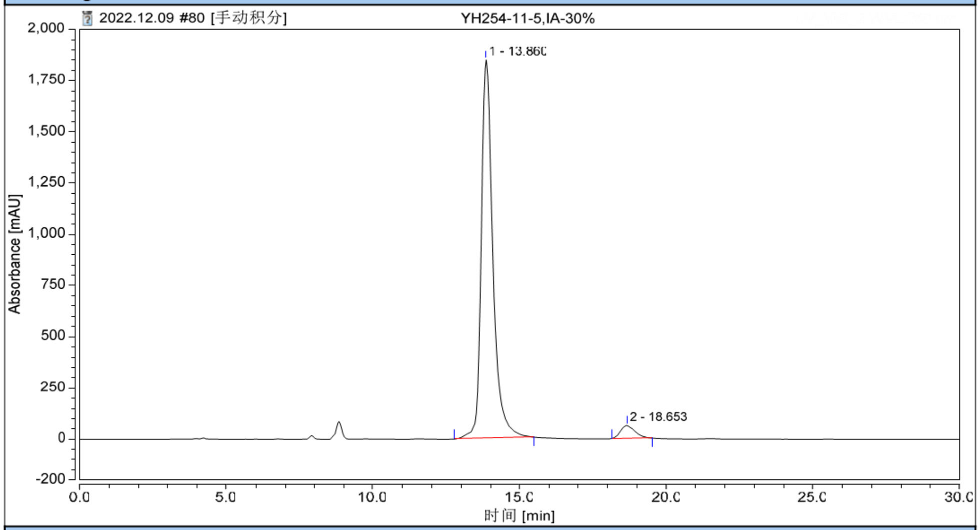

Integration Results

No.	Peak Name	Retention Time min	Area mAu*min	Height mAU	Relative Area %	Relative Height %	Amount n.a.
1		13.860	850.393	1844.337	95.90	96.71	n.a.
2		18.653	36.317	62.659	4.10	3.29	n.a.
**Total:**			**886.710**	**1906.996**	**100.00**	**100.00**	

Supplementary Figure 225. HPLC spectrum of **5n**

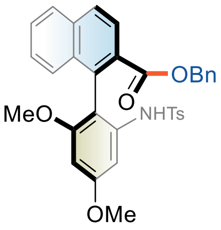

5o

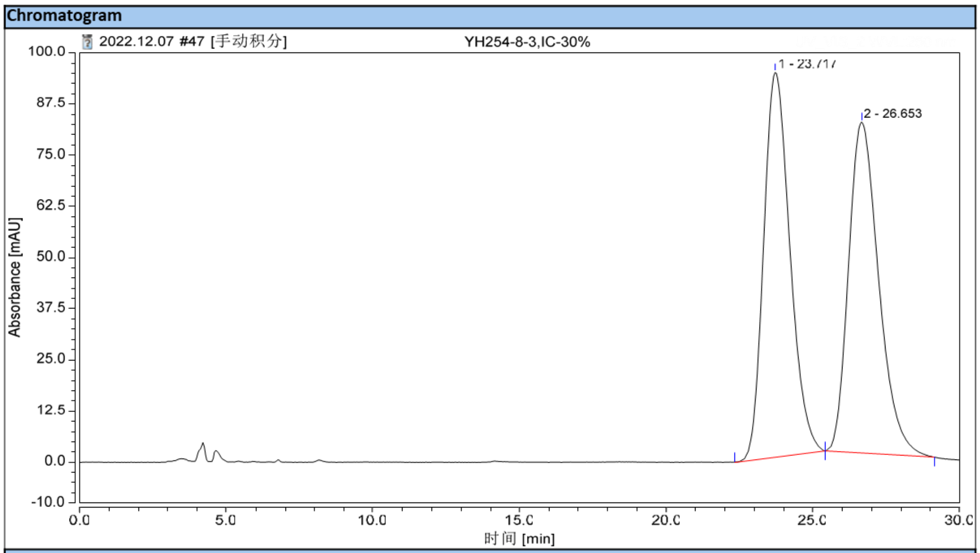

Integration Results

No.	Peak Name	Retention Time min	Area mAu*min	Height mAU	Relative Area %	Relative Height %	Amount n.a.
1		23.717	97.259	93.882	50.29	53.75	n.a.
2		26.653	96.143	80.784	49.71	46.25	n.a.
**Total:**			**193.402**	**174.666**	**100.00**	**100.00**	

Supplementary Figure 226. HPLC spectrum of racemic **5o**

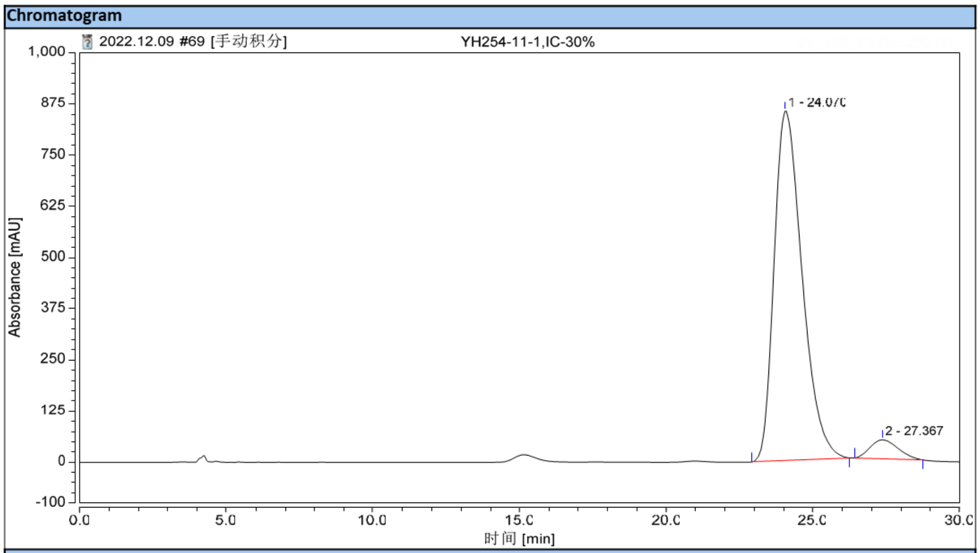

Integration Results

No.	Peak Name	Retention Time min	Area mAu*min	Height mAU	Relative Area %	Relative Height %	Amount n.a.
1		24.070	942.331	852.913	94.87	94.87	n.a.
2		27.367	50.936	46.087	5.13	5.13	n.a.
**Total:**			**993.267**	**899.000**	**100.00**	**100.00**	

Supplementary Figure 227. HPLC spectrum of **5o**

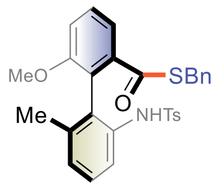

6a

Chromatogram

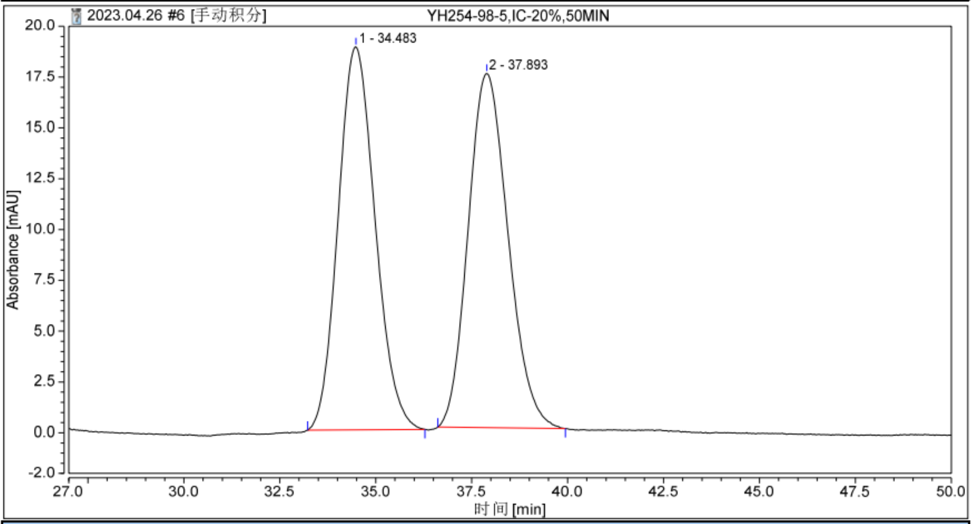

Integration Results

No.	Peak Name	Retention Time min	Area mAu*min	Height mAU	Relative Area %	Relative Height %	Amount n.a.
1		34.483	21.021	18.852	50.03	51.96	n.a.
2		37.893	21.000	17.432	49.97	48.04	n.a.
**Total:**			**42.021**	**36.284**	**100.00**	**100.00**	

Supplementary Figure 228. HPLC spectrum of racemic **6a**

Chromatogram

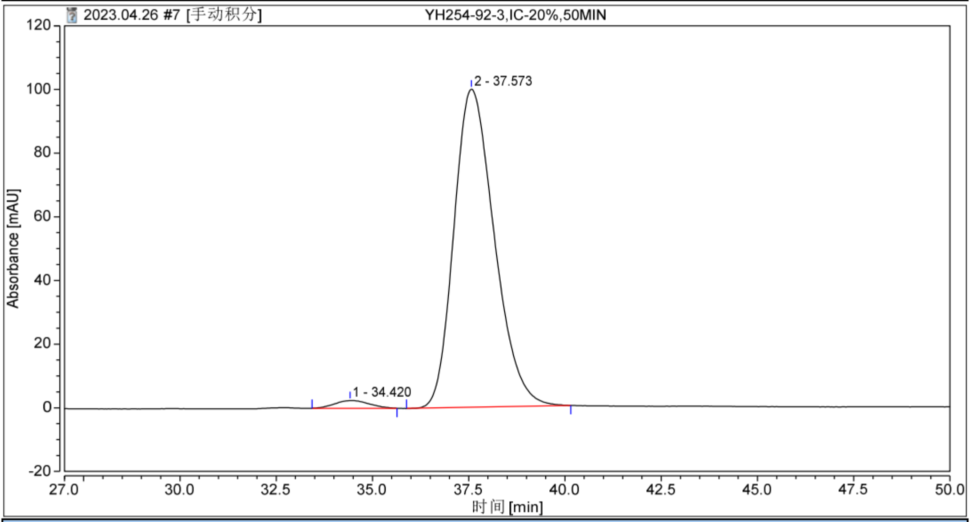

Integration Results

No.	Peak Name	Retention Time min	Area mAu*min	Height mAU	Relative Area %	Relative Height %	Amount n.a.
1		34.420	2.512	2.440	2.03	2.38	n.a.
2		37.573	121.503	99.933	97.97	97.62	n.a.
**Total:**			**124.015**	**102.373**	**100.00**	**100.00**	

Supplementary Figure 229. HPLC spectrum of **6a**

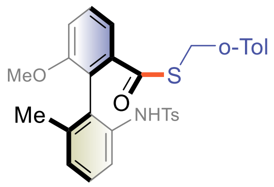

6b

Chromatogram

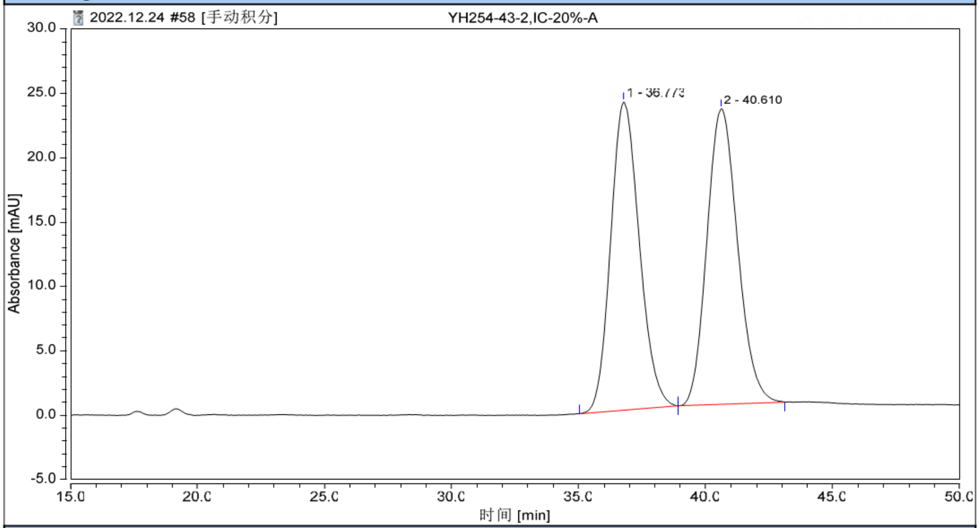

Integration Results

No.	Peak Name	Retention Time min	Area mAu*min	Height mAU	Relative Area %	Relative Height %	Amount n.a.
1		36.773	31.293	23.921	49.30	51.04	n.a.
2		40.610	32.185	22.944	50.70	48.96	n.a.
**Total:**			**63.478**	**46.865**	**100.00**	**100.00**	

Supplementary Figure 230. HPLC spectrum of racemic **6b**

Chromatogram

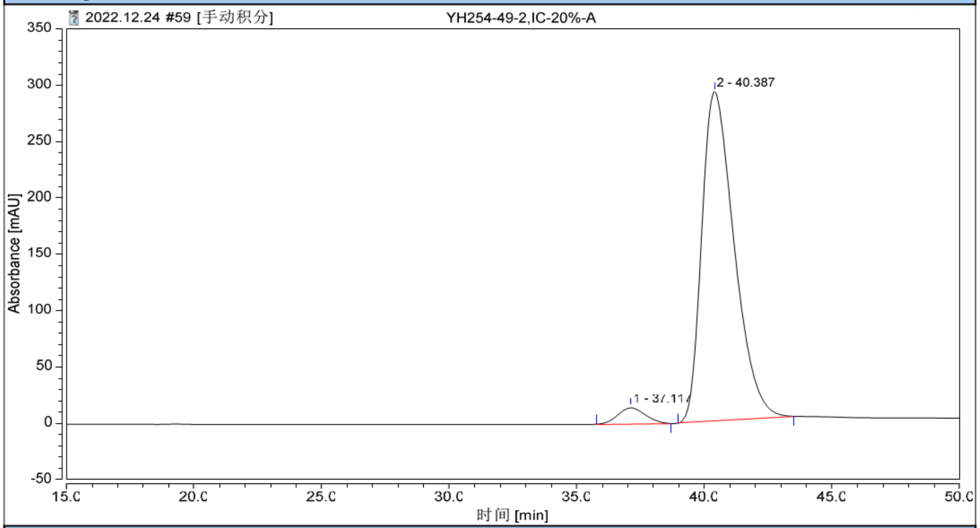

Integration Results

No.	Peak Name	Retention Time min	Area mAu*min	Height mAU	Relative Area %	Relative Height %	Amount n.a.
1		37.117	17.745	14.147	3.95	4.62	n.a.
2		40.387	430.986	292.311	96.05	95.38	n.a.
**Total:**			**448.731**	**306.459**	**100.00**	**100.00**	

Supplementary Figure 231. HPLC spectrum of **6b**

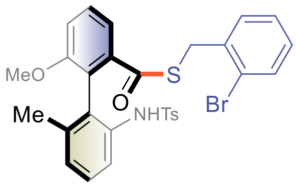

6c

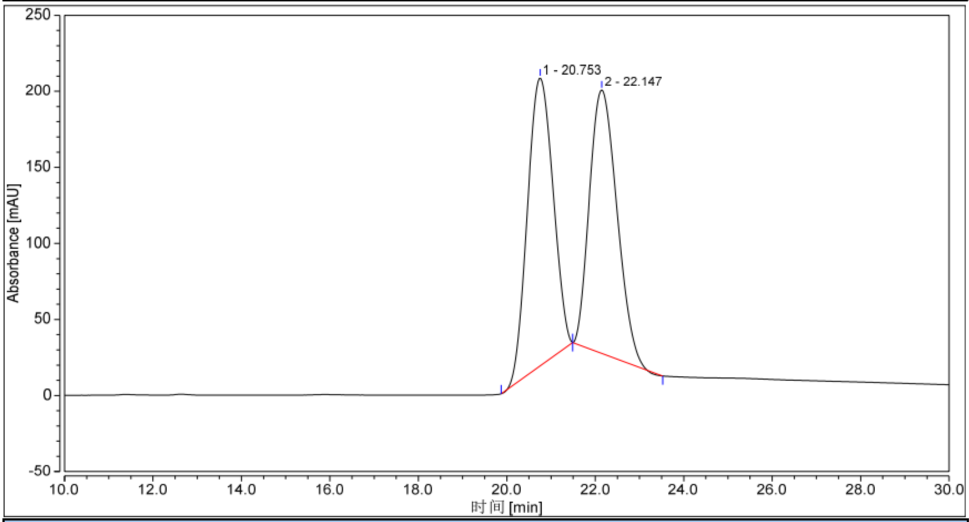

Integration Results

No.	Peak Name	Retention Time min	Area mAu*min	Height mAU	Relative Area %	Relative Height %	Amount n.a.
1		20.753	126.864	189.305	50.43	52.22	n.a.
2		22.147	124.703	173.183	49.57	47.78	n.a.
**Total:**			**251.567**	**362.488**	**100.00**	**100.00**	

Supplementary Figure 232. HPLC spectrum of racemic **6c**

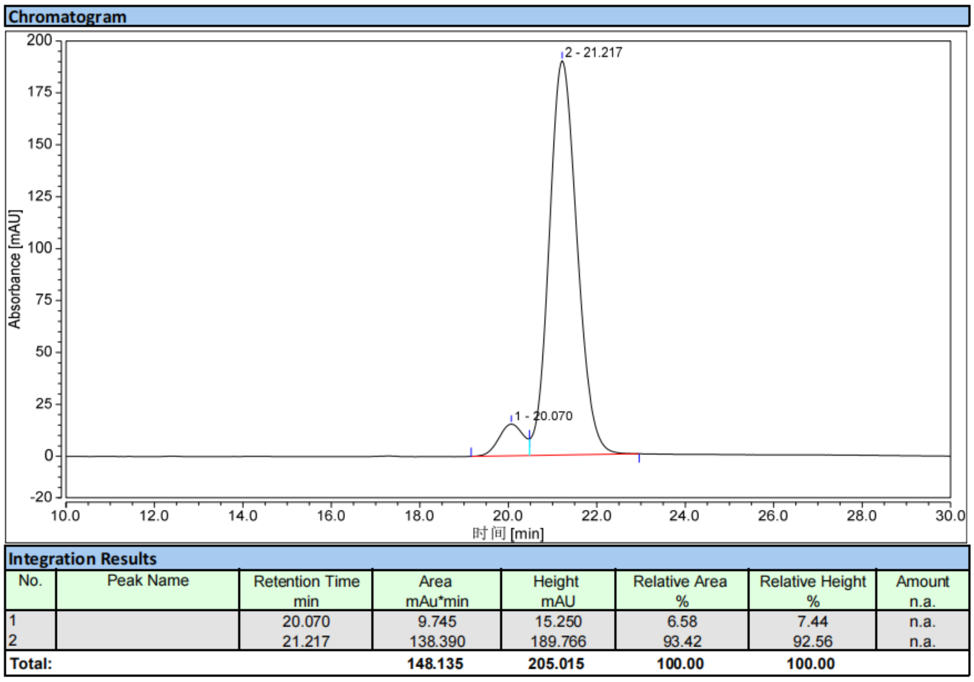

Integration Results

No.	Peak Name	Retention Time min	Area mAu*min	Height mAU	Relative Area %	Relative Height %	Amount n.a.
1		20.070	9.745	15.250	6.58	7.44	n.a.
2		21.217	138.390	189.766	93.42	92.56	n.a.
**Total:**			**148.135**	**205.015**	**100.00**	**100.00**	

Supplementary Figure 233. HPLC spectrum of **6c**

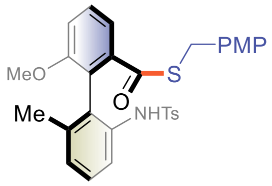

6d

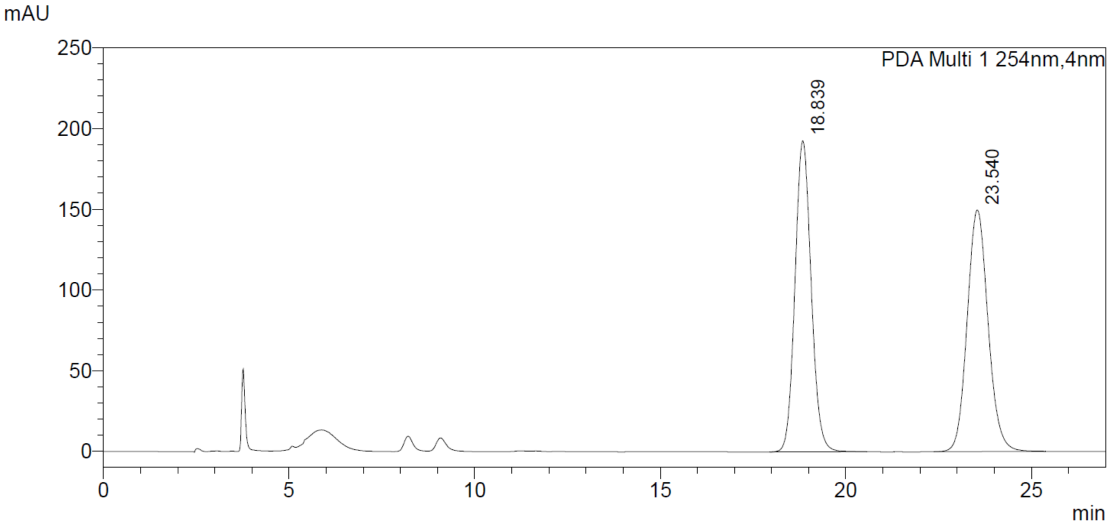

<Peak Table>

PDA Ch1 254nm

Peak#	Ret. Time	Area	Height	Area%
1	18.839	5849191	192678	49.940
2	23.540	5863221	149723	50.060
Total		11712412	342401	100.000

Supplementary Figure 234. HPLC spectrum of racemic **6d**

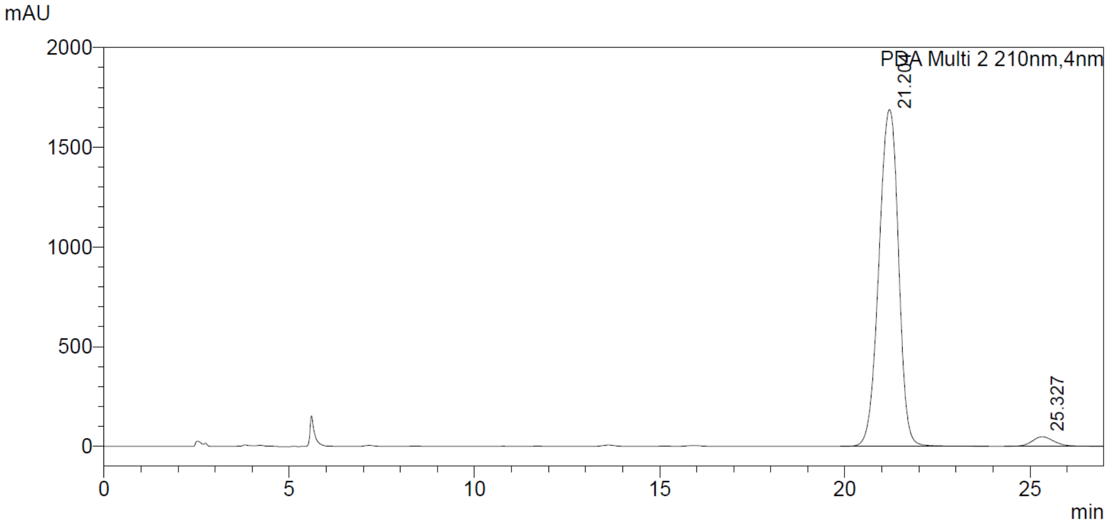

<Peak Table>

PDA Ch1 254nm

Peak#	Ret. Time	Area	Height	Area%
1	21.204	15734809	462473	97.207
2	25.328	452088	11016	2.793
Total		16186898	473490	100.000

Supplementary Figure 235. HPLC spectrum of **6d**

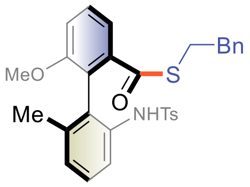

6e

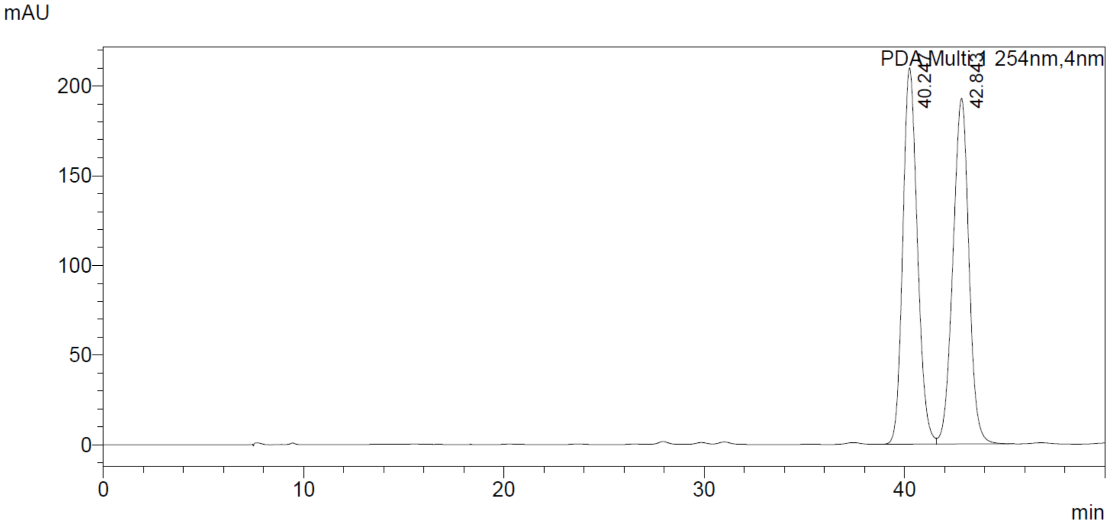

<Peak Table>

PDA Ch1 254nm

Peak#	Ret. Time	Area	Height	Area%
1	40.247	10652918	209725	49.838
2	42.843	10722232	192800	50.162
Total		21375150	402524	100.000

Supplementary Figure 236. HPLC spectrum of racemic **6e**

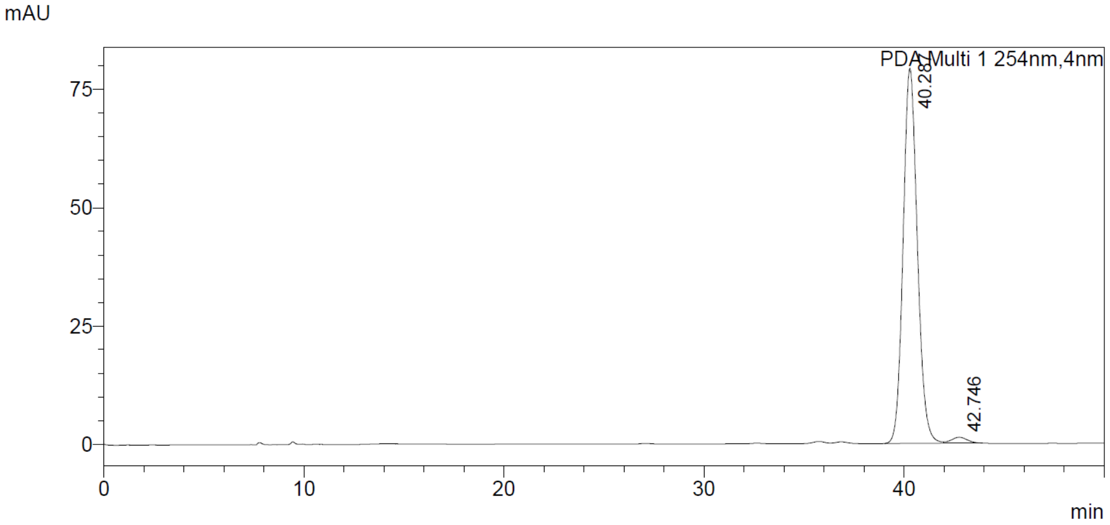

<Peak Table>

PDA Ch1 254nm

Peak#	Ret. Time	Area	Height	Area%
1	40.287	3911042	79256	98.226
2	42.746	70641	1264	1.774
Total		3981683	80521	100.000

Supplementary Figure 237. HPLC spectrum of **6e**

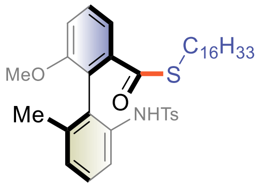

6f

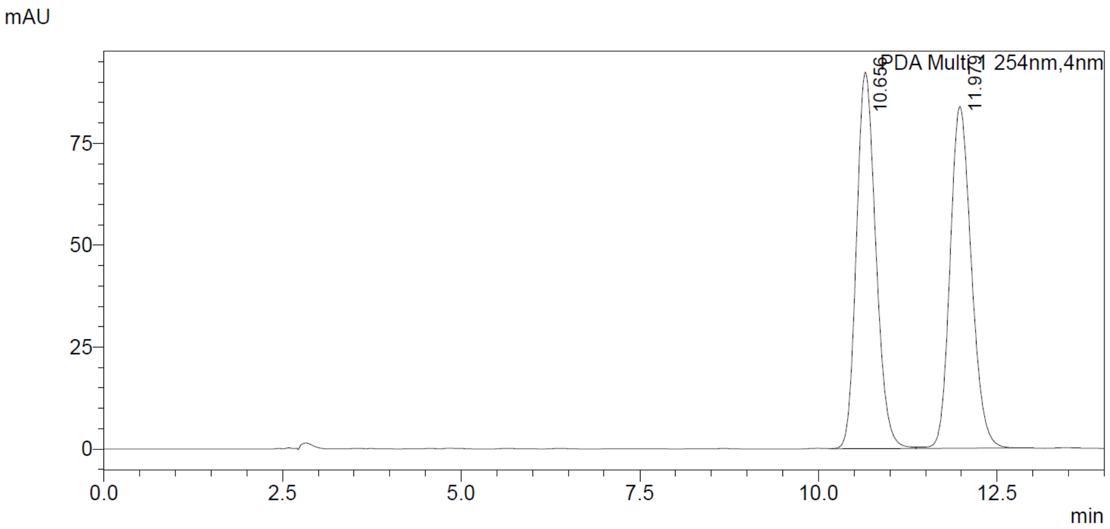

<Peak Table>

PDA Ch1 254nm

Peak#	Ret. Time	Area	Height	Area%
1	10.656	1742756	92370	50.058
2	11.979	1738741	83895	49.942
Total		3481496	176265	100.000

Supplementary Figure 238. HPLC spectrum of racemic **6f**

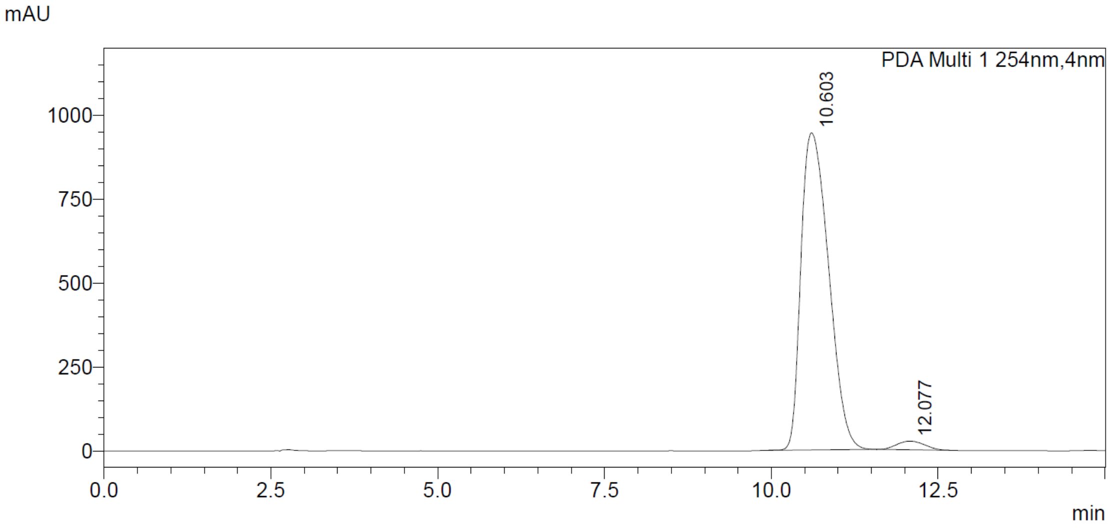

<Peak Table>

PDA Ch1 254nm

Peak#	Ret. Time	Area	Height	Area%
1	10.603	27274683	945436	97.317
2	12.077	751973	25673	2.683
Total		28026656	971109	100.000

Supplementary Figure 239. HPLC spectrum of **6f**

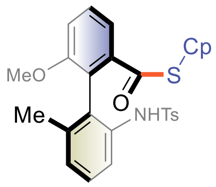

6g

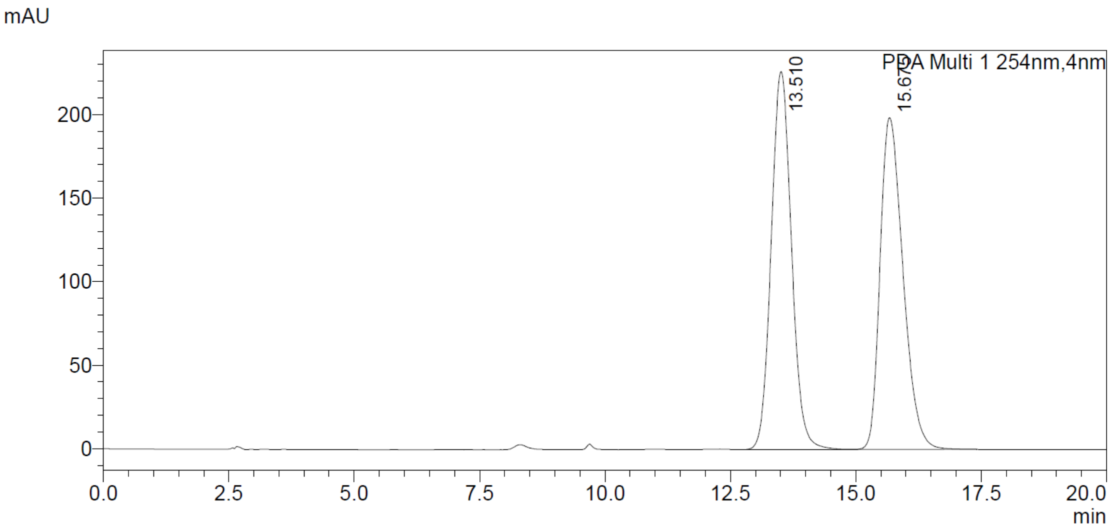

<Peak Table>

PDA Ch1 254nm

Peak#	Ret. Time	Area	Height	Area%
1	13.510	6367018	226249	50.209
2	15.675	6314070	198500	49.791
Total		12681088	424749	100.000

Supplementary Figure 240. HPLC spectrum of racemic **6g**

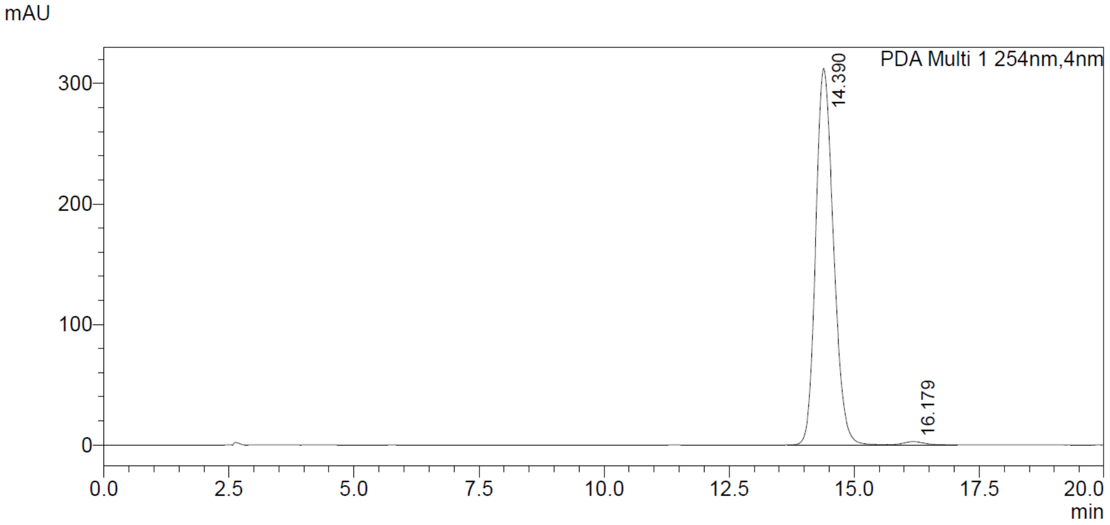

<Peak Table>

PDA Ch1 254nm

Peak#	Ret. Time	Area	Height	Area%
1	14.390	7722791	312307	99.001
2	16.179	77901	2688	0.999
Total		7800692	314996	100.000

Supplementary Figure 241. HPLC spectrum of **6g**

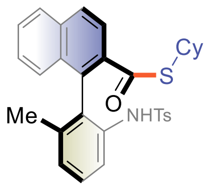

6h

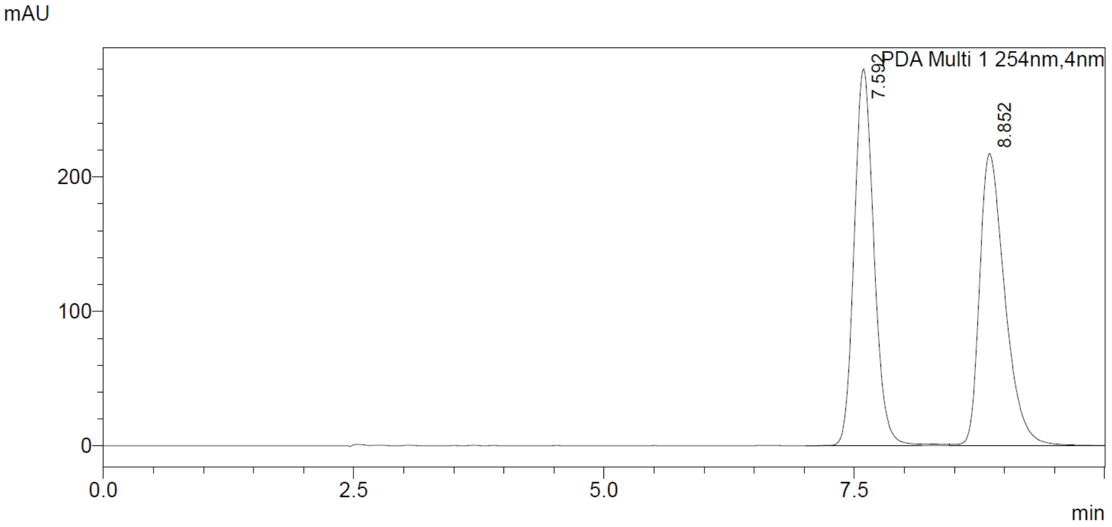

<Peak Table>

PDA Ch1 254nm

Peak#	Ret. Time	Area	Height	Area%
1	7.592	3851830	280271	50.269
2	8.852	3810579	217304	49.731
Total		7662409	497575	100.000

Supplementary Figure 242. HPLC spectrum of racemic **6h**

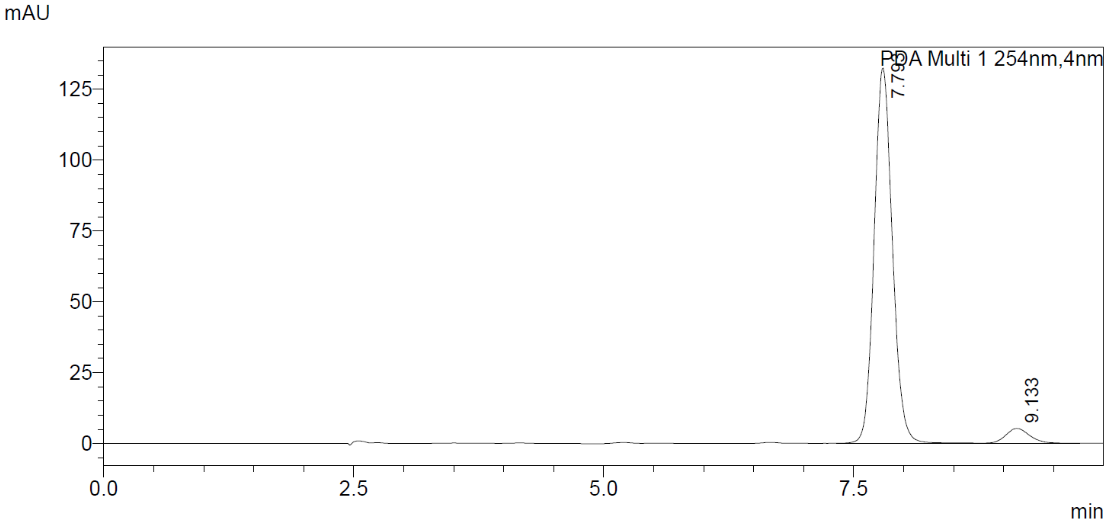

<Peak Table>

PDA Ch1 254nm

Peak#	Ret. Time	Area	Height	Area%
1	7.793	1684252	132380	95.034
2	9.133	88015	5251	4.966
Total		1772267	137632	100.000

Supplementary Figure 243. HPLC spectrum of **6h**

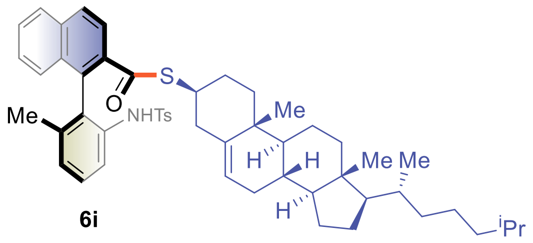

6i

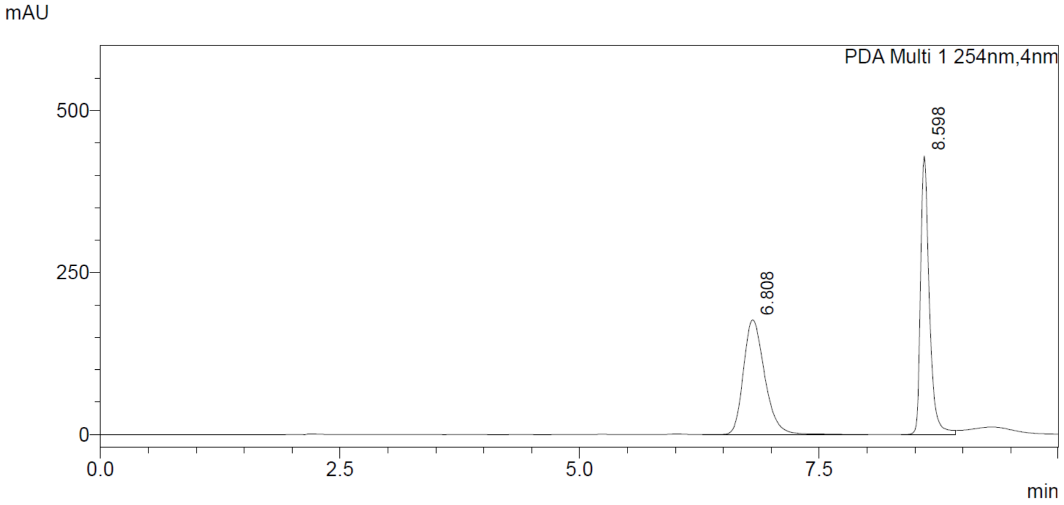

<Peak Table>

PDA Ch1 254nm

Peak#	Ret. Time	Area	Height	Area%
1	6.808	2731106	176869	49.930
2	8.598	2738751	429952	50.070
Total		5469857	606822	100.000

Supplementary Figure 244. HPLC spectrum of racemic **6i**

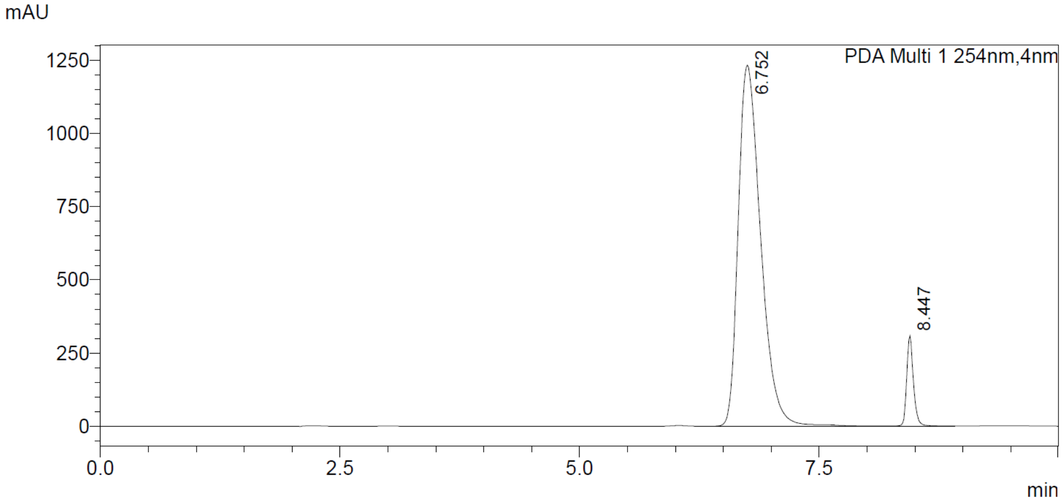

<Peak Table>

PDA Ch1 254nm

Peak#	Ret. Time	Area	Height	Area%
1	6.752	20131594	1232709	92.960
2	8.447	1524519	309138	7.040
Total		21656113	1541846	100.000

Supplementary Figure 245. HPLC spectrum of **6i**

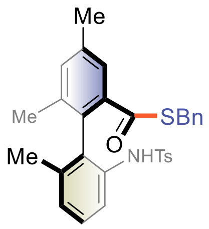

6j

Chromatogram
D:\HPLC data\Huang Group\CYX\YH250\91\YH250-91-2,AD-10%-racemic.lcd

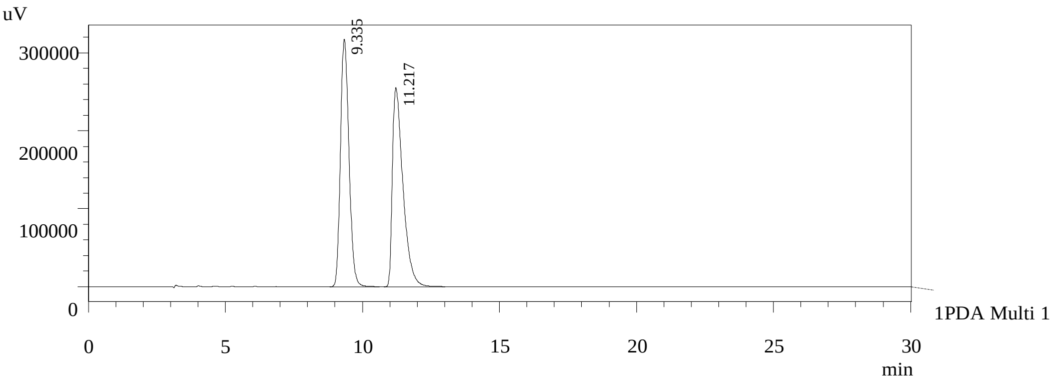

1 PDA Multi 1 / 254nm 4nm

PeakTable

Ch1 254nm 4nm

Peak#	Ret. Time	Area	Height	Area %	Height %
1	9.335	6852621	317728	50.018	55.437
2	11.217	6847589	255408	49.982	44.563
Total		13700210	573136	100.000	100.000

Supplementary Figure 246. HPLC spectrum of racemic **6j**

Chromatogram
D:\HPLC data\Huang Group\CYX\YH250\90\YH250-90-2,AD-10%.lcd

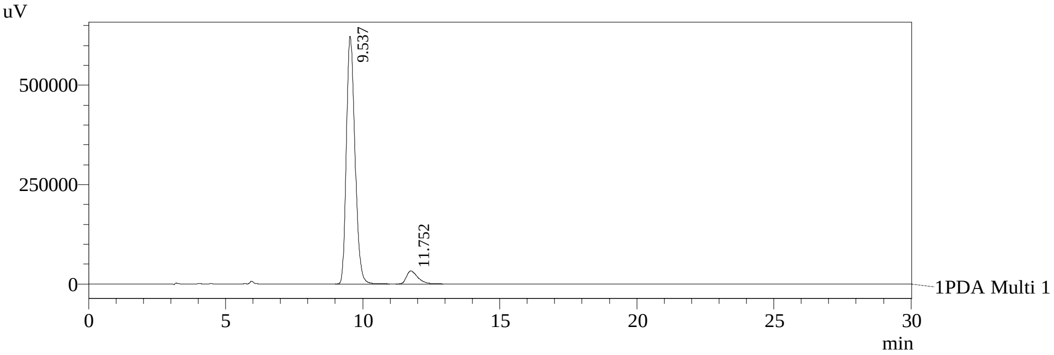

1 PDA Multi 1 / 254nm 4nm

PeakTable

Ch1 254nm 4nm

Peak#	Ret. Time	Area	Height	Area %	Height %
1	9.537	13594604	623242	93.640	95.081
2	11.752	923373	32242	6.360	4.919
Total		14517976	655484	100.000	100.000

Supplementary Figure 247. HPLC spectrum of **6j**

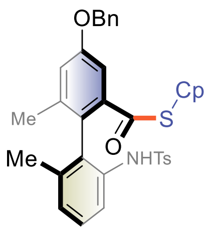

6k

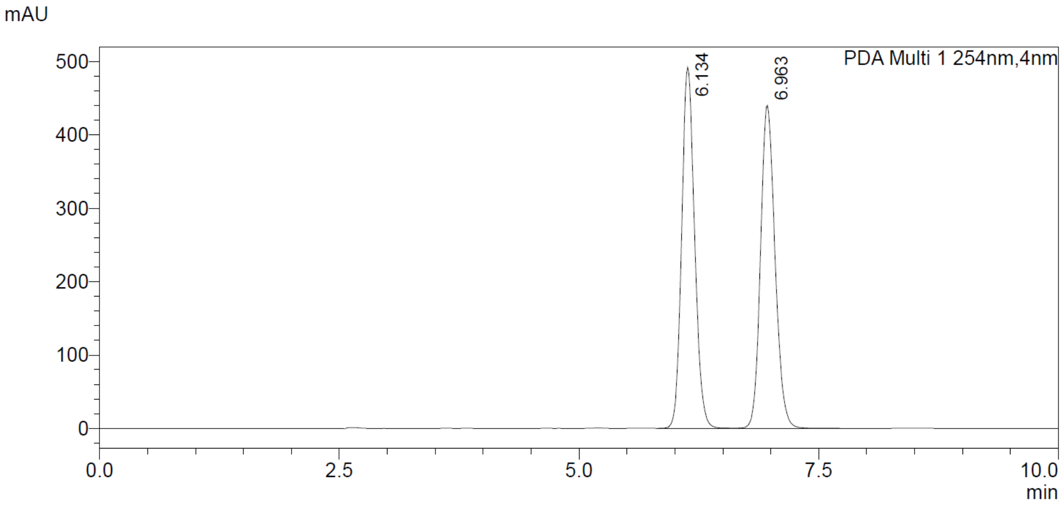

<Peak Table>

PDA Ch1 254nm

Peak#	Ret. Time	Area	Height	Area%
1	6.134	4624497	491940	49.971
2	6.963	4629939	440059	50.029
Total		9254436	931999	100.000

Supplementary Figure 248. HPLC spectrum of racemic **6k**

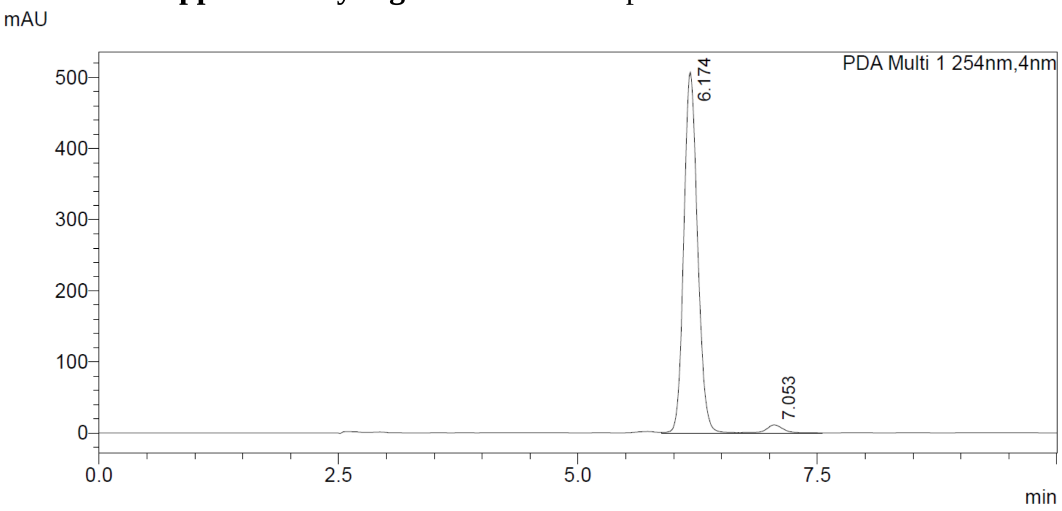

<Peak Table>

PDA Ch1 254nm

Peak#	Ret. Time	Area	Height	Area%
1	6.174	5055958	507064	97.426
2	7.053	133599	11204	2.574
Total		5189556	518268	100.000

Supplementary Figure 249. HPLC spectrum of **6k**

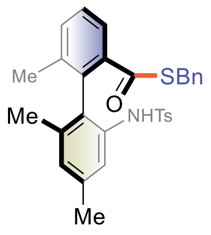

6l

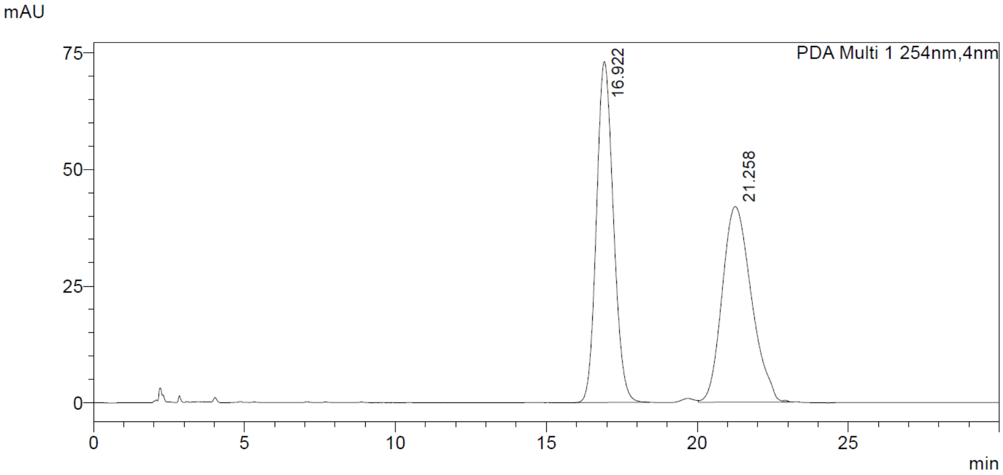

<Peak Table>

PDA Ch1 254nm

Peak#	Ret. Time	Area	Height	Area%
1	16.922	2854555	73083	50.213
2	21.258	2830326	41969	49.787
Total		5684880	115051	100.000

Supplementary Figure 250. HPLC spectrum of racemic **6l**

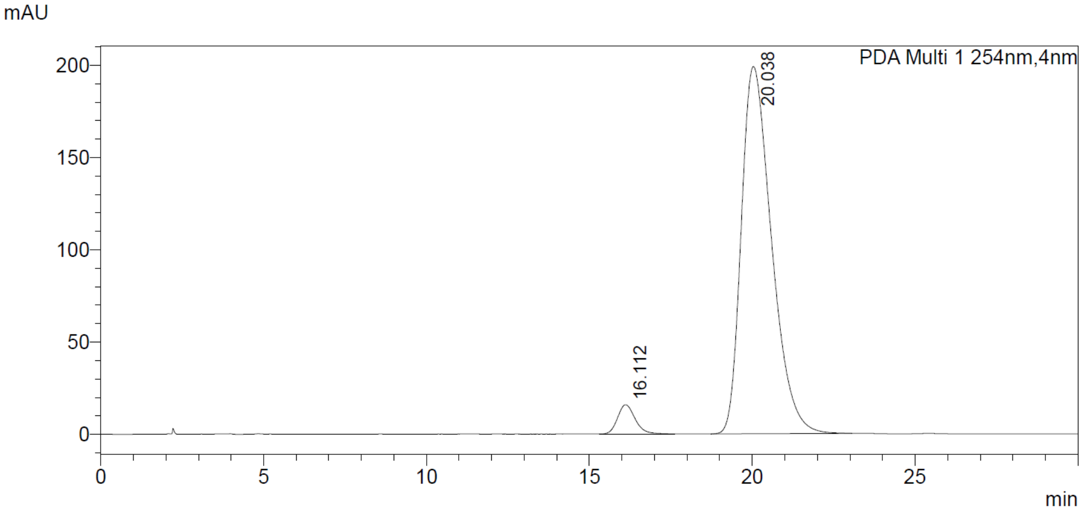

<Peak Table>

PDA Ch1 254nm

Peak#	Ret. Time	Area	Height	Area%
1	16.112	592952	15856	4.408
2	20.038	12859178	199071	95.592
Total		13452130	214927	100.000

Supplementary Figure 251. HPLC spectrum of **6l**

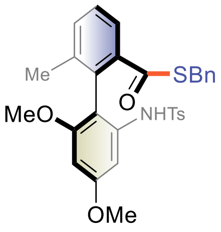

6m

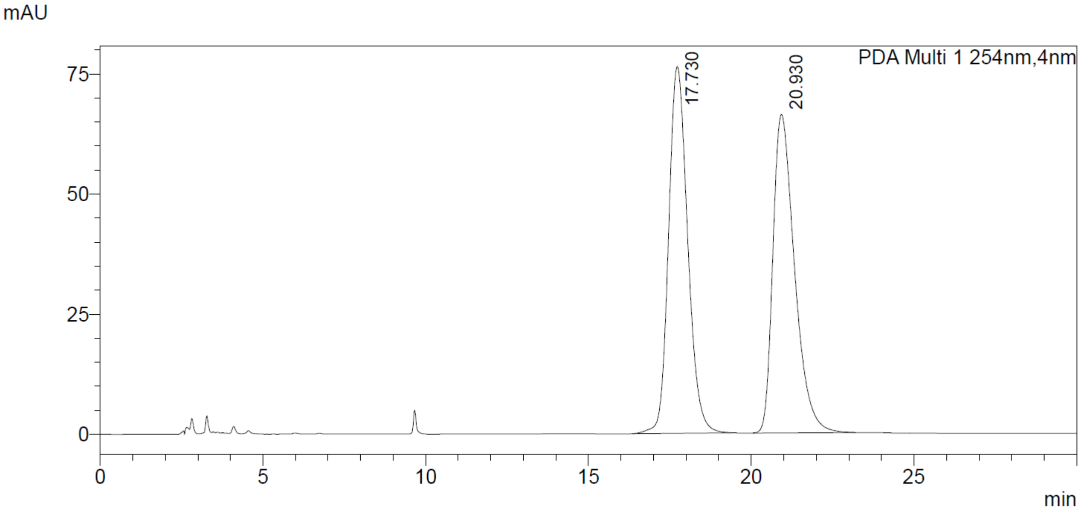

<Peak Table>

PDA Ch1 254nm

Peak#	Ret. Time	Area	Height	Area%
1	17.730	3119027	76389	50.402
2	20.930	3069327	66327	49.598
Total		6188353	142715	100.000

Supplementary Figure 252. HPLC spectrum of racemic **6m**

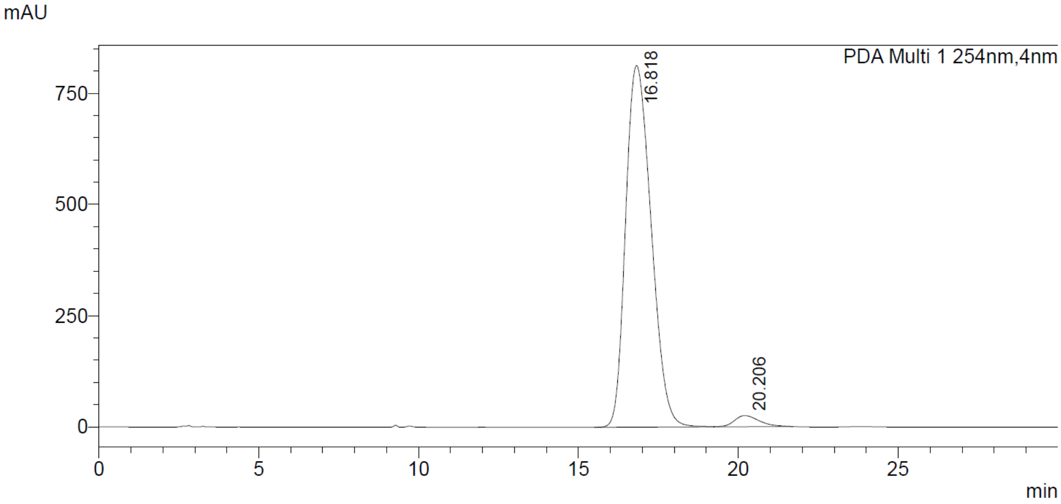

<Peak Table>

PDA Ch1 254nm

Peak#	Ret. Time	Area	Height	Area%
1	16.818	45184341	812976	97.079
2	20.206	1359352	24984	2.921
Total		46543693	837960	100.000

Supplementary Figure 253. HPLC spectrum of **6m**

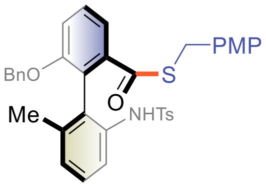

6n

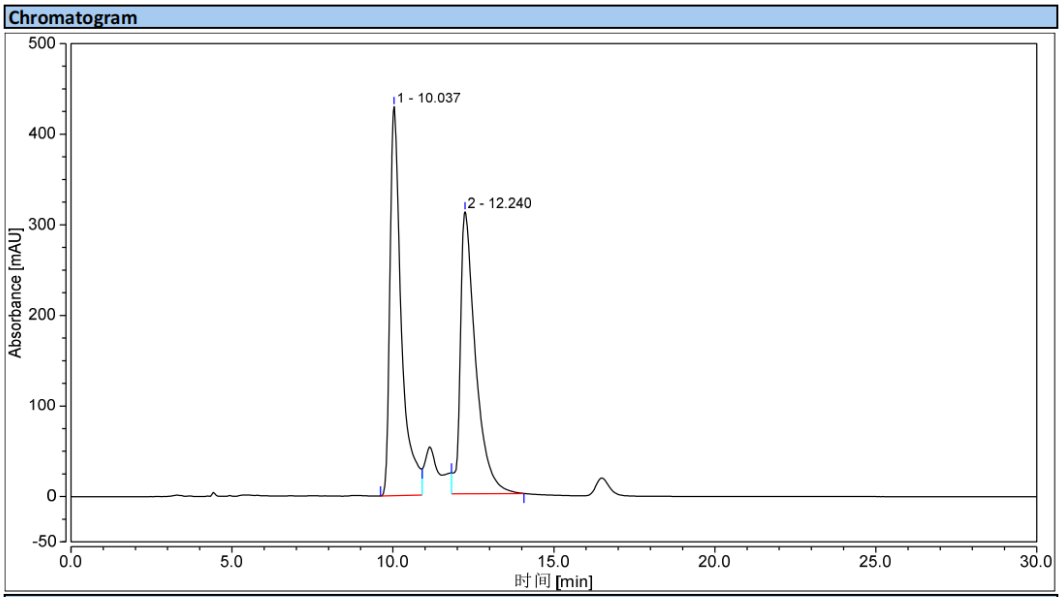

Integration Results

No.	Peak Name	Retention Time min	Area mAu*min	Height mAU	Relative Area %	Relative Height %	Amount n.a.
1		10.037	176.868	429.304	50.94	57.98	n.a.
2		12.240	170.338	311.159	49.06	42.02	n.a.
**Total:**			**347.206**	**740.462**	**100.00**	**100.00**	

Supplementary Figure 254. HPLC spectrum of racemic **6n**

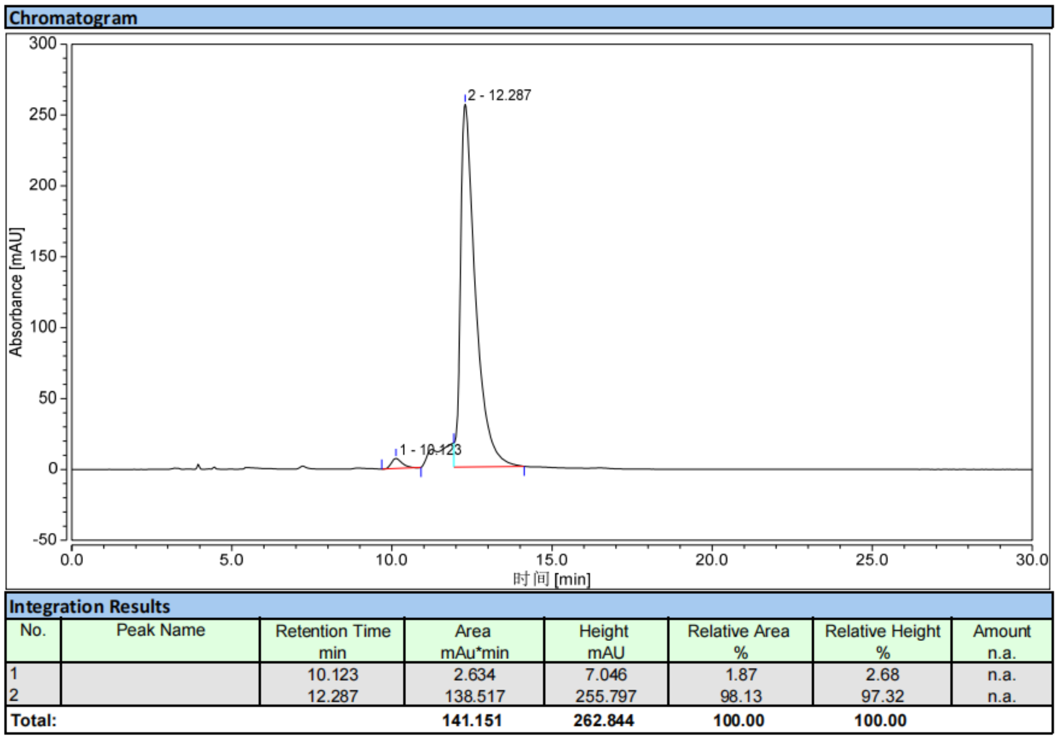

Integration Results

No.	Peak Name	Retention Time min	Area mAu*min	Height mAU	Relative Area %	Relative Height %	Amount n.a.
1		10.123	2.634	7.046	1.87	2.68	n.a.
2		12.287	138.517	255.797	98.13	97.32	n.a.
**Total:**			**141.151**	**262.844**	**100.00**	**100.00**	

Supplementary Figure 255. HPLC spectrum of **6n**

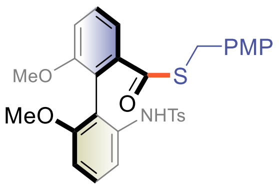

6o

Chromatogram

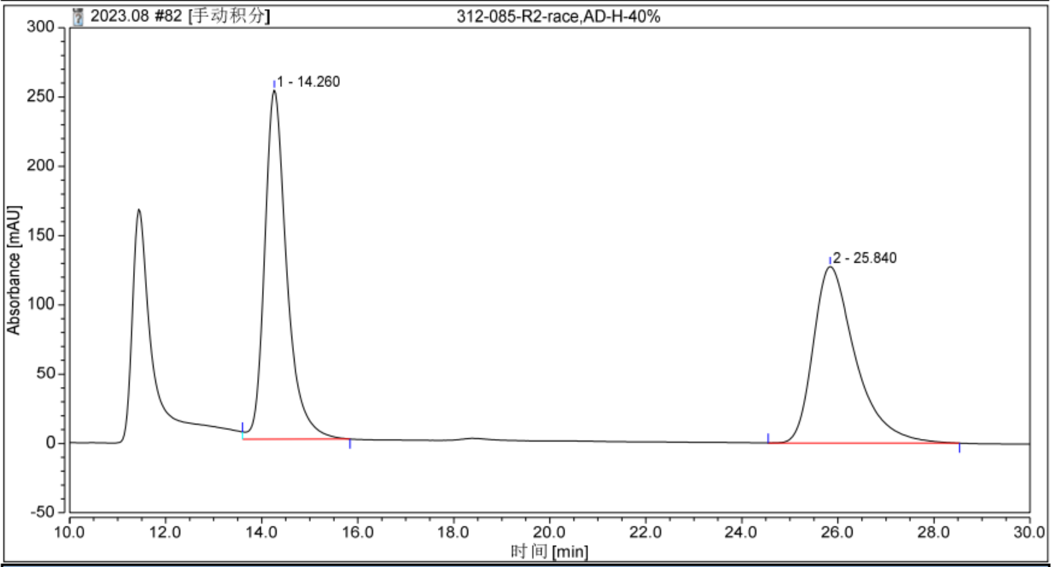

Integration Results

No.	Peak Name	Retention Time min	Area mAu*min	Height mAU	Relative Area %	Relative Height %	Amount n.a.
1		14.260	134.431	252.020	50.89	66.44	n.a.
2		25.840	129.724	127.293	49.11	33.56	n.a.
Total:			264.154	379.313	100.00	100.00	

Supplementary Figure 256. HPLC spectrum of racemic **6o**

Chromatogram

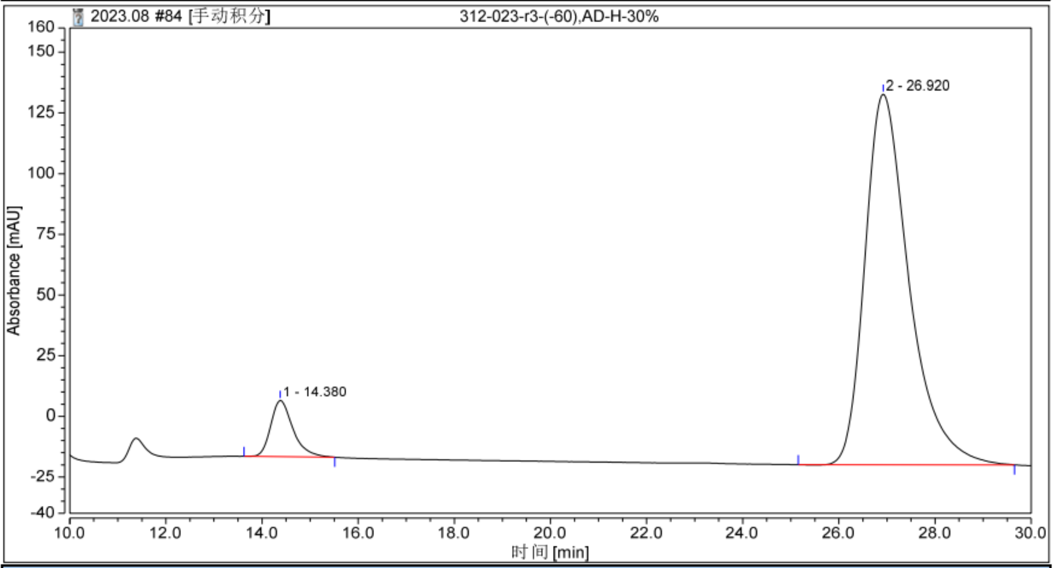

Integration Results

No.	Peak Name	Retention Time min	Area mAu*min	Height mAU	Relative Area %	Relative Height %	Amount n.a.
1		14.380	12.423	23.183	7.10	13.18	n.a.
2		26.920	162.460	152.724	92.90	86.82	n.a.
Total:			174.883	175.907	100.00	100.00	

Supplementary Figure 257. HPLC spectrum of **6o**

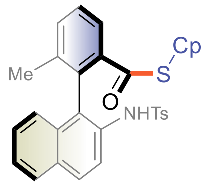

6p

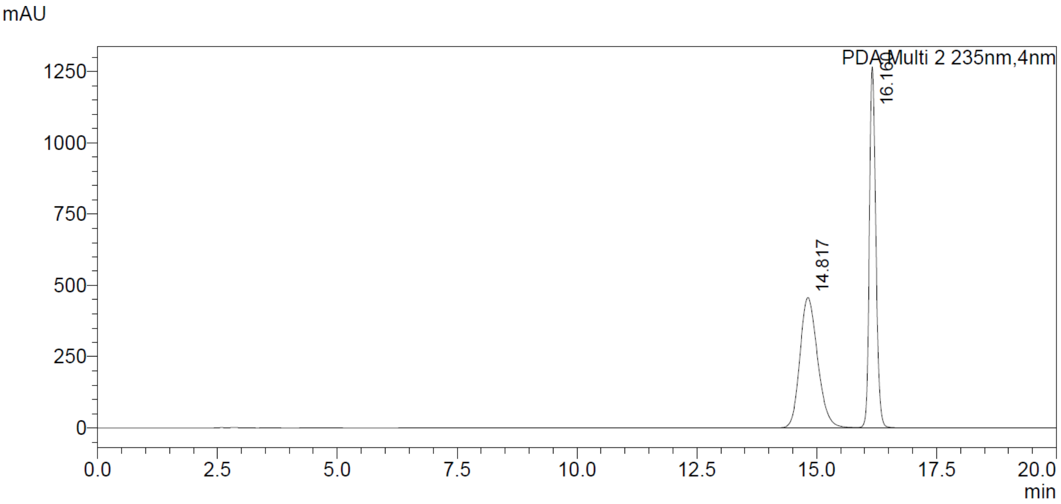

<Peak Table>

PDA Ch2 235nm

Peak#	Ret. Time	Area	Height	Area%
1	14.817	11771874	457347	49.853
2	16.160	11841112	1266230	50.147
Total		23612986	1723577	100.000

Supplementary Figure 258. HPLC spectrum of racemic **6p**

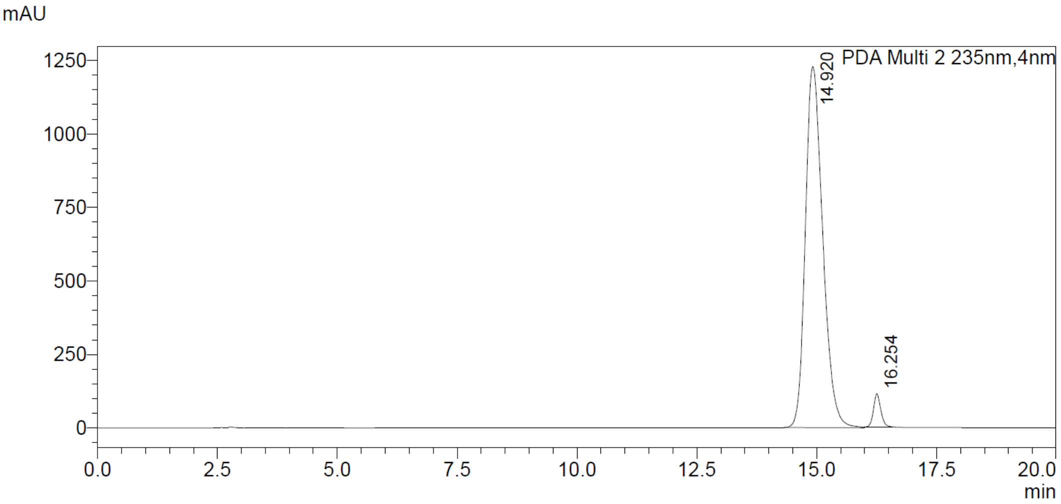

<Peak Table>

PDA Ch2 235nm

Peak#	Ret. Time	Area	Height	Area%
1	14.920	30941127	1227732	96.297
2	16.254	1189867	113196	3.703
Total		32130994	1340928	100.000

Supplementary Figure 259. HPLC spectrum of **6p**

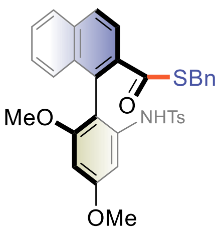

6q

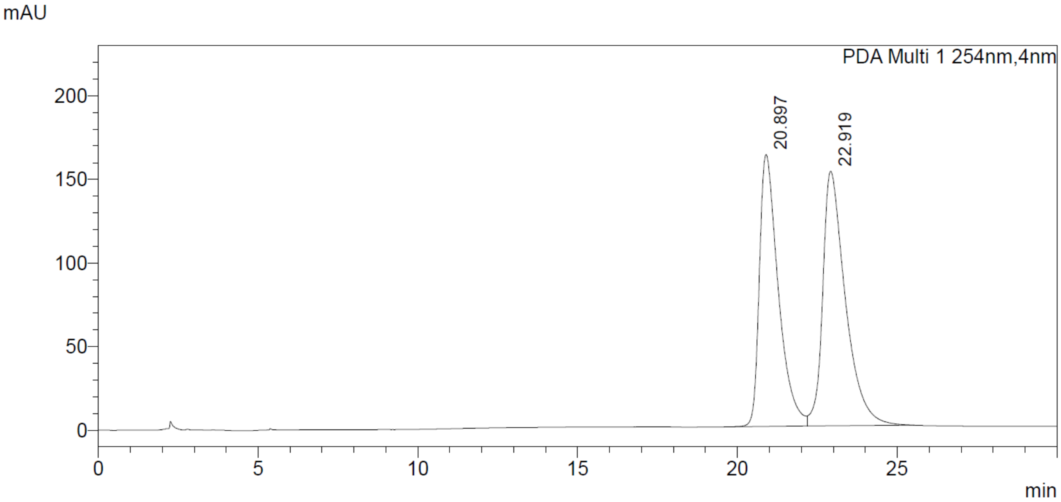

<Peak Table>

PDA Ch1 254nm

Peak#	Ret. Time	Area	Height	Area%
1	20.897	6704178	162599	47.054
2	22.919	7543649	152276	52.946
Total		14247827	314875	100.000

Supplementary Figure 260. HPLC spectrum of racemic **6q**

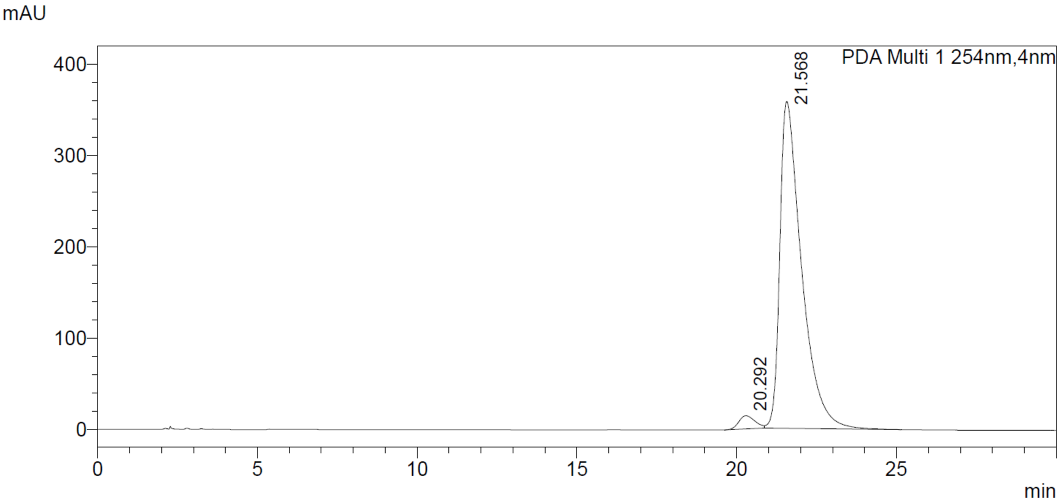

<Peak Table>

PDA Ch1 254nm

Peak#	Ret. Time	Area	Height	Area%
1	20.292	500386	14355	2.868
2	21.568	16948405	358225	97.132
Total		17448791	372580	100.000

Supplementary Figure 261. HPLC spectrum of **6q**

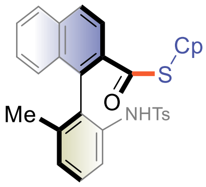

6r

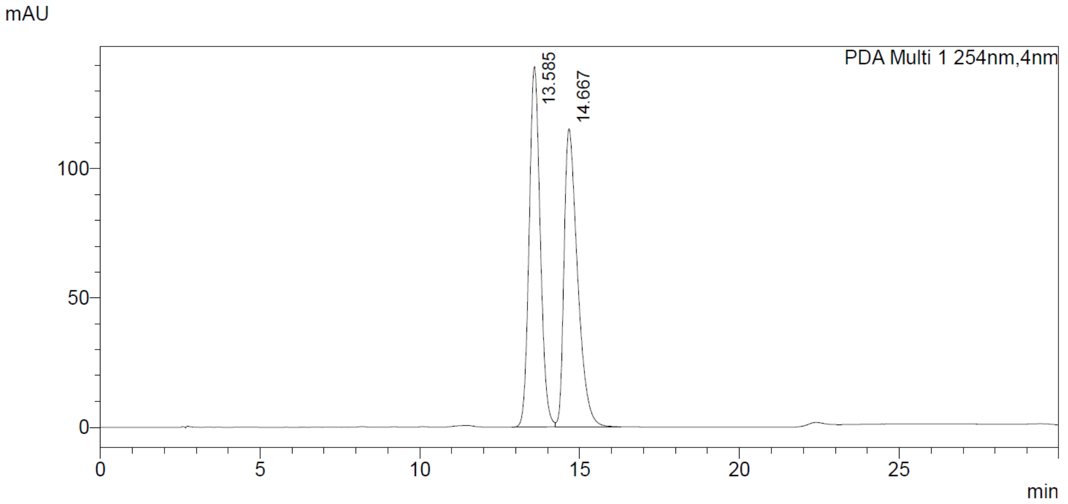

<Peak Table>

PDA Ch1 254nm

Peak#	Ret. Time	Area	Height	Area%
1	13.585	3364847	139441	49.884
2	14.667	3380498	115331	50.116
Total		6745345	254773	100.000

Supplementary Figure 262. HPLC spectrum of racemic **6r**

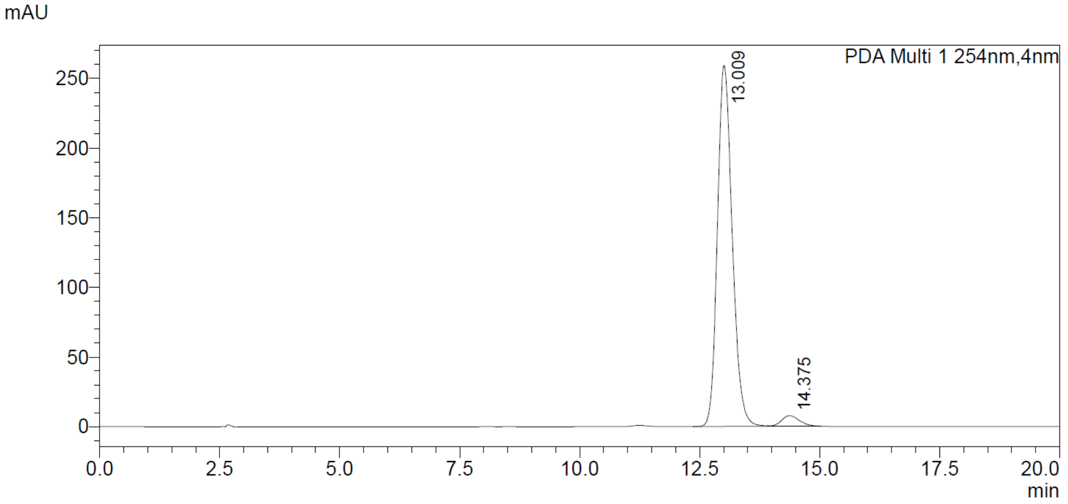

<Peak Table>

PDA Ch1 254nm

Peak#	Ret. Time	Area	Height	Area%
1	13.009	5578223	259191	96.554
2	14.375	199080	7709	3.446
Total		5777303	266900	100.000

Supplementary Figure 263. HPLC spectrum of **6r**

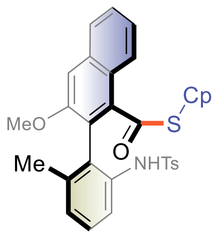

6s

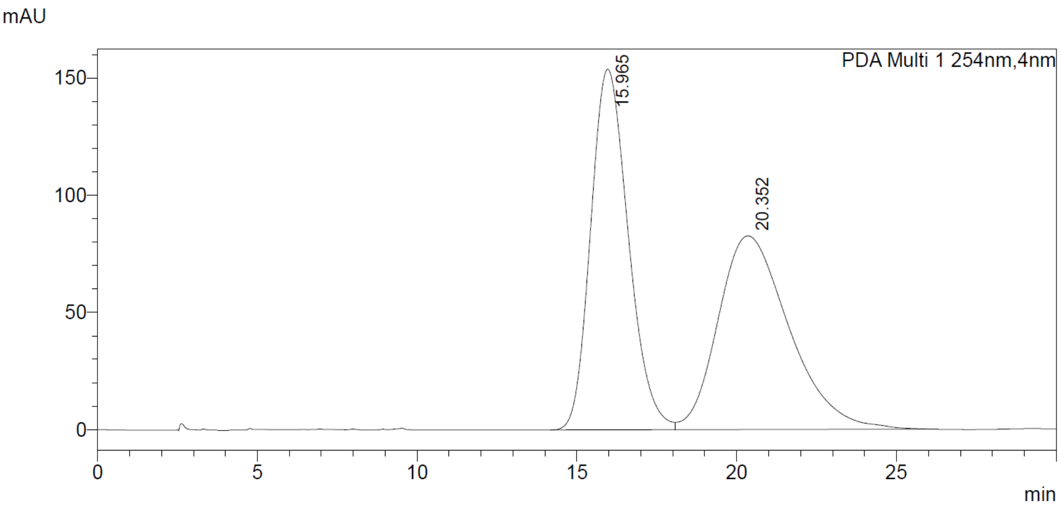

<Peak Table>

PDA Ch1 254nm

Peak#	Ret. Time	Area	Height	Area%
1	15.965	12626102	153802	49.632
2	20.352	12813383	82616	50.368
Total		25439485	236418	100.000

Supplementary Figure 264. HPLC spectrum of racemic **6s**

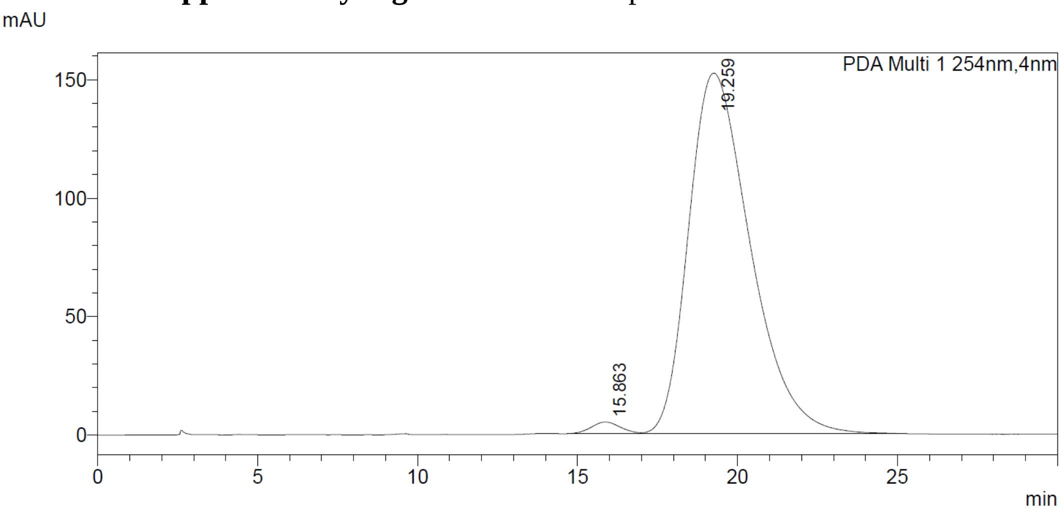

<Peak Table>

PDA Ch1 254nm

Peak#	Ret. Time	Area	Height	Area%
1	15.863	314549	4942	1.515
2	19.259	20441890	152250	98.485
Total		20756439	157192	100.000

Supplementary Figure 265. HPLC spectrum of **6s**

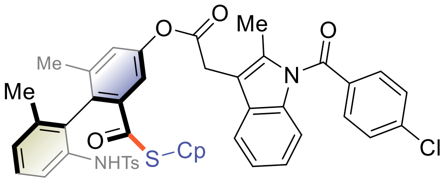

6t

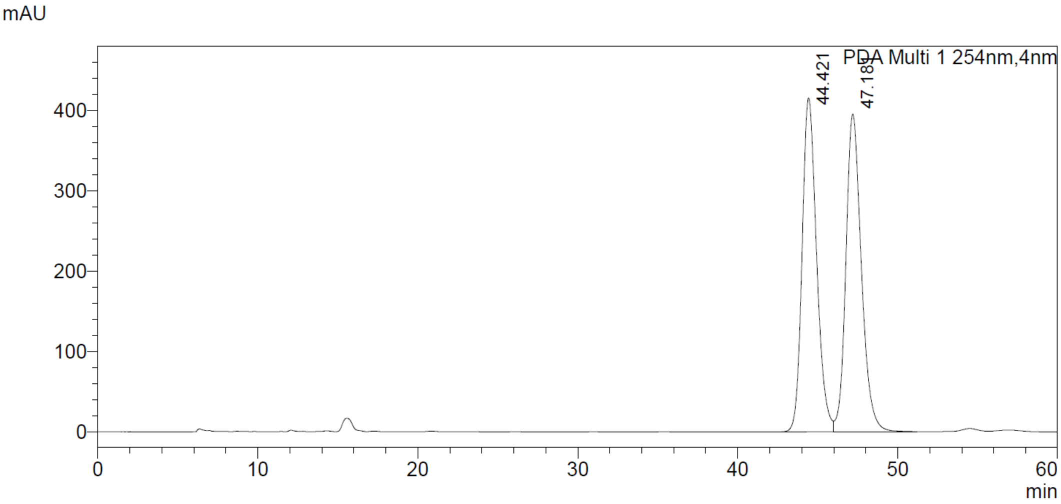

<Peak Table>

PDA Ch1 254nm

Peak#	Ret. Time	Area	Height	Area%
1	44.421	26181228	415844	49.588
2	47.181	26616015	395735	50.412
Total		52797243	811579	100.000

Supplementary Figure 266. HPLC spectrum of racemic **6t**

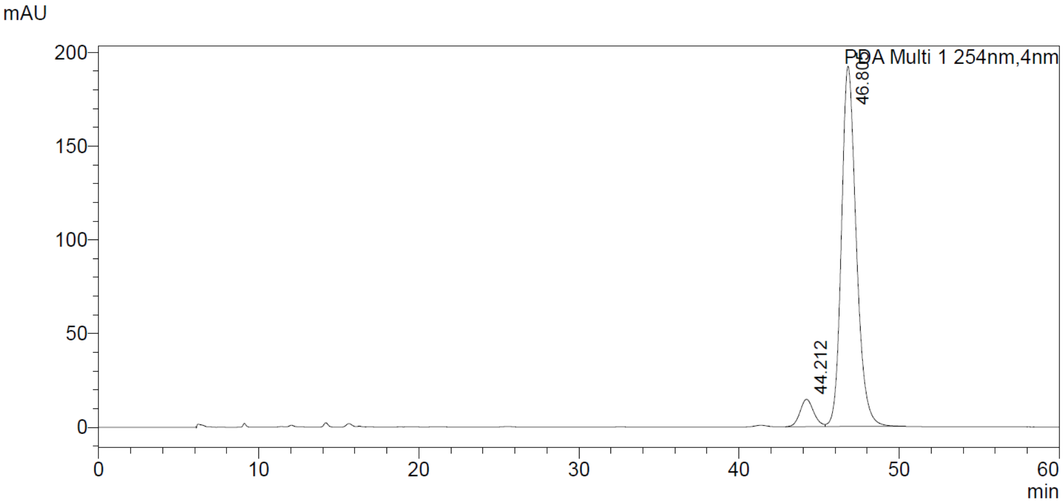

<Peak Table>

PDA Ch1 254nm

Peak#	Ret. Time	Area	Height	Area%
1	44.212	868752	14562	6.547
2	46.805	12400021	192240	93.453
Total		13268773	206801	100.000

Supplementary Figure 267. HPLC spectrum of **6t**

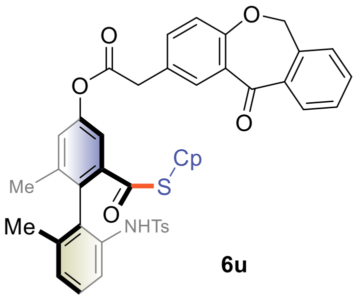

6u

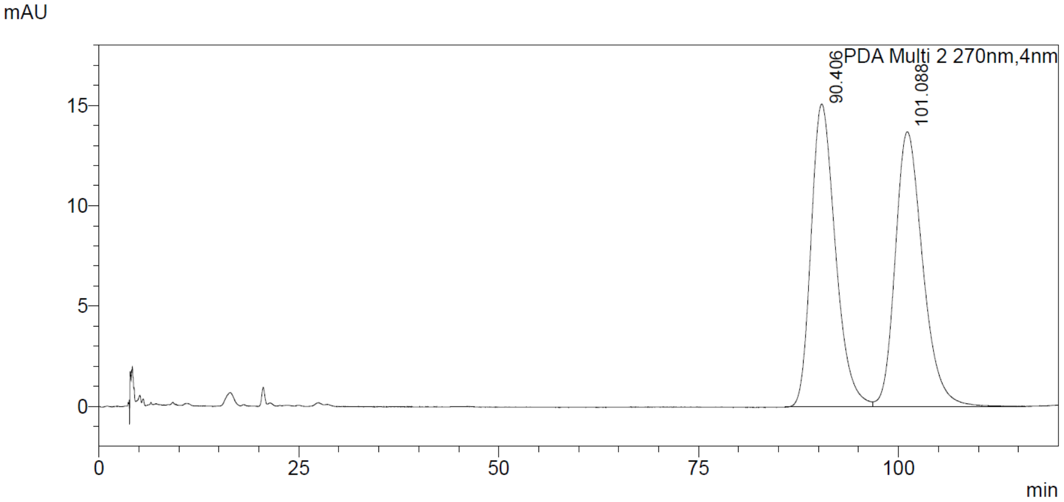

<Peak Table>

PDA Ch2 270nm

Peak#	Ret. Time	Area	Height	Area%
1	90.406	3189768	15068	49.274
2	101.088	3283770	13685	50.726
Total		6473538	28753	100.000

Supplementary Figure 268. HPLC spectrum of racemic **6u**

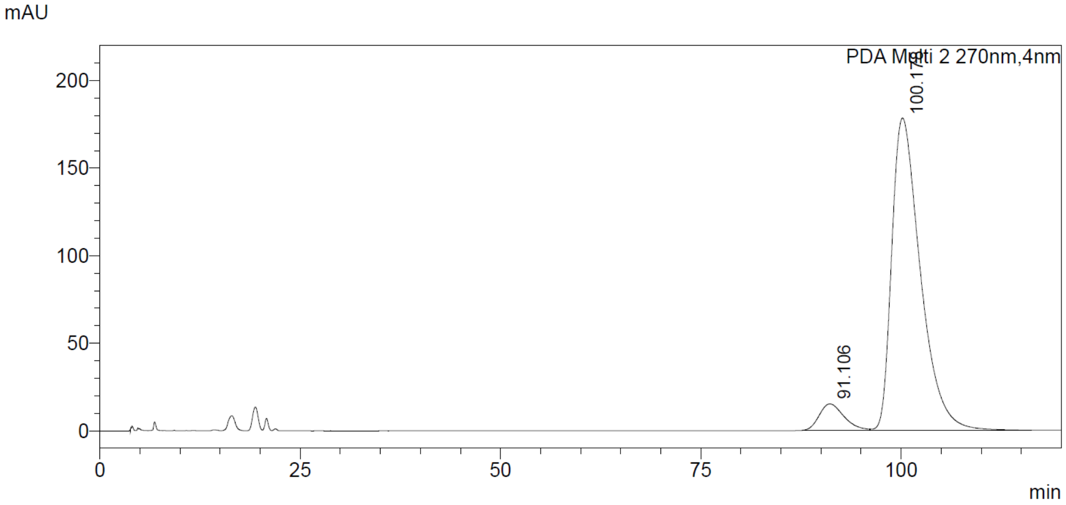

<Peak Table>

PDA Ch2 270nm

Peak#	Ret. Time	Area	Height	Area%
1	91.106	3200584	15059	6.742
2	100.178	44271610	178206	93.258
Total		47472194	193265	100.000

Supplementary Figure 269. HPLC spectrum of **6u**

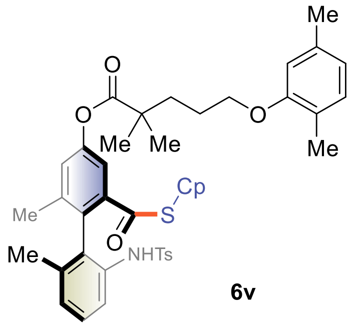

6v

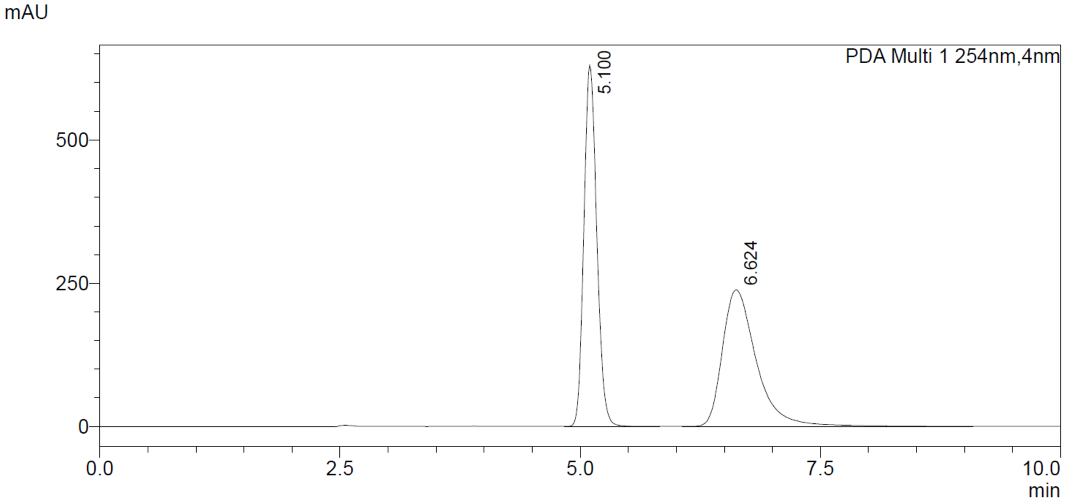

<Peak Table>

PDA Ch1 254nm

Peak#	Ret. Time	Area	Height	Area%
1	5.100	5880090	630165	50.074
2	6.624	5862752	238425	49.926
Total		11742842	868590	100.000

Supplementary Figure 270. HPLC spectrum of racemic **6v**

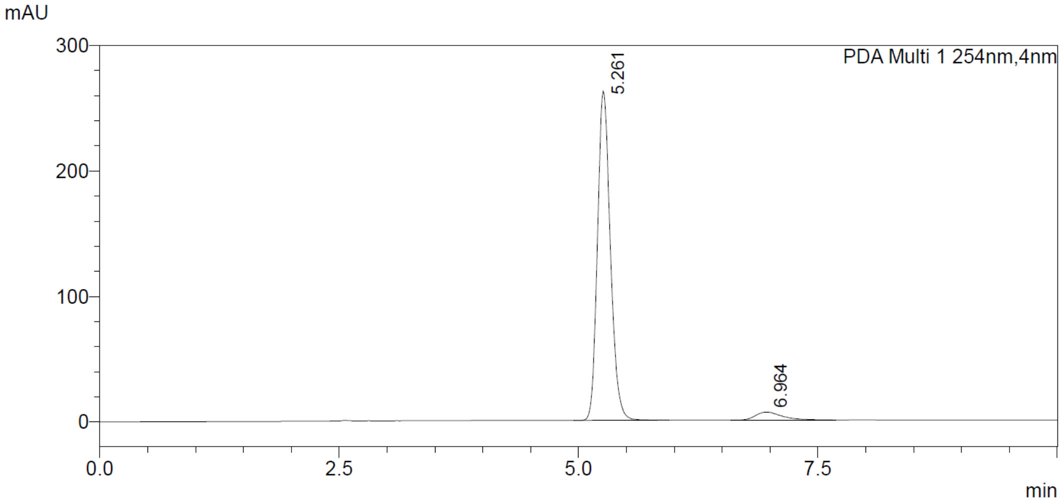

<Peak Table>

PDA Ch1 254nm

Peak#	Ret. Time	Area	Height	Area%
1	5.261	2461777	262418	95.088
2	6.964	127156	6466	4.912
Total		2588933	268884	100.000

Supplementary Figure 271. HPLC spectrum of **6v**

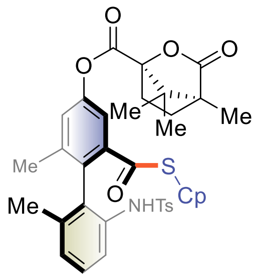

6w

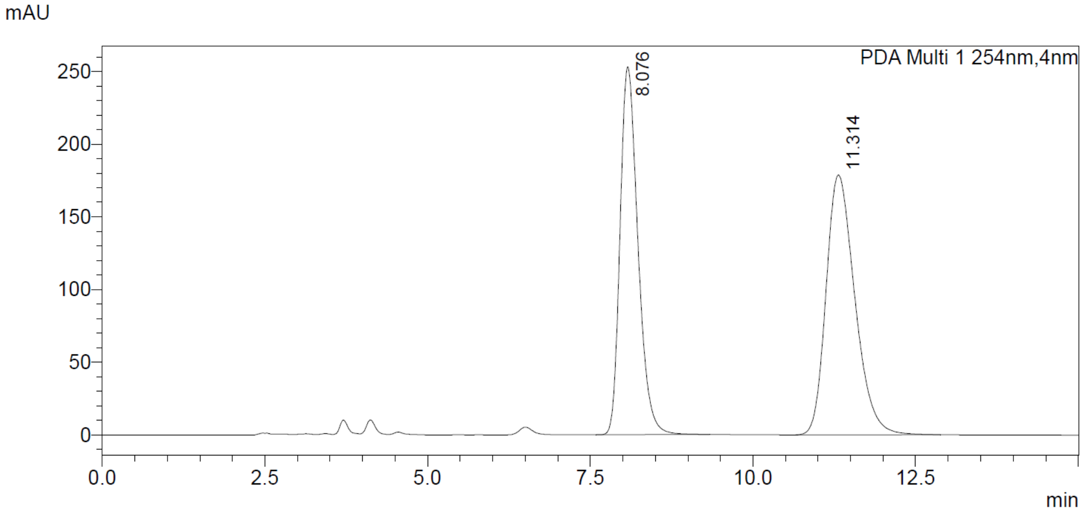

<Peak Table>

PDA Ch1 254nm

Peak#	Ret. Time	Area	Height	Area%
1	8.076	4840162	253231	46.791
2	11.314	5504142	178822	53.209
Total		10344304	432053	100.000

Supplementary Figure 272. HPLC spectrum of racemic **6w**

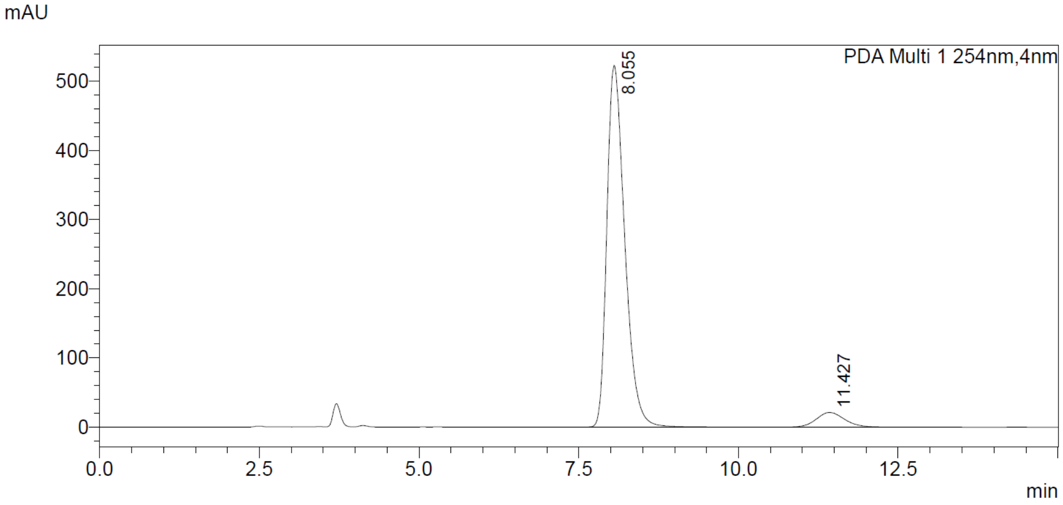

<Peak Table>

PDA Ch1 254nm

Peak#	Ret. Time	Area	Height	Area%
1	8.055	10179713	522933	93.904
2	11.427	660795	21235	6.096
Total		10840508	544168	100.000

Supplementary Figure 273. HPLC spectrum of **6w**

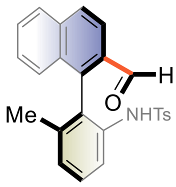

7a

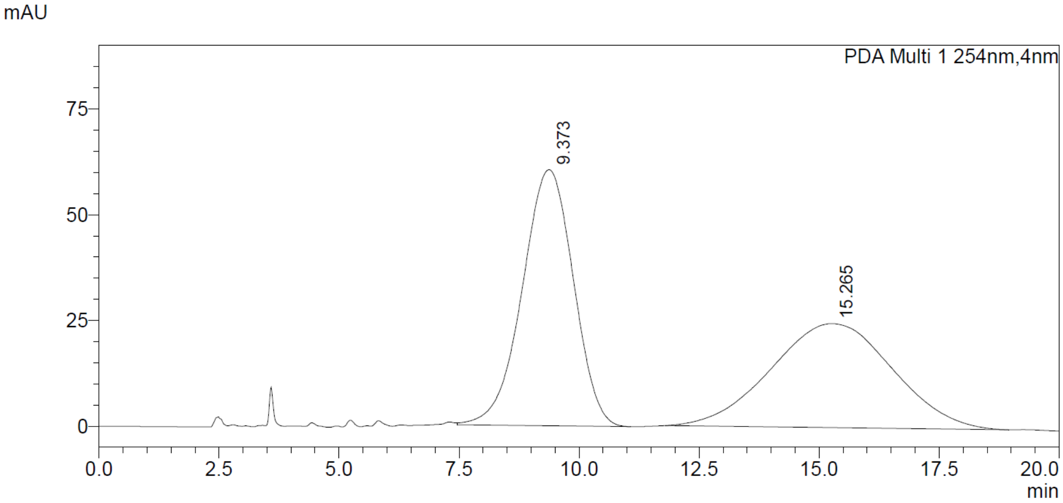

<Peak Table>

PDA Ch1 254nm

Peak#	Ret. Time	Area	Height	Area%
1	9.373	4485792	60509	50.885
2	15.265	4329798	24574	49.115
Total		8815590	85083	100.000

Supplementary Figure 274. HPLC spectrum of racemic **7a**

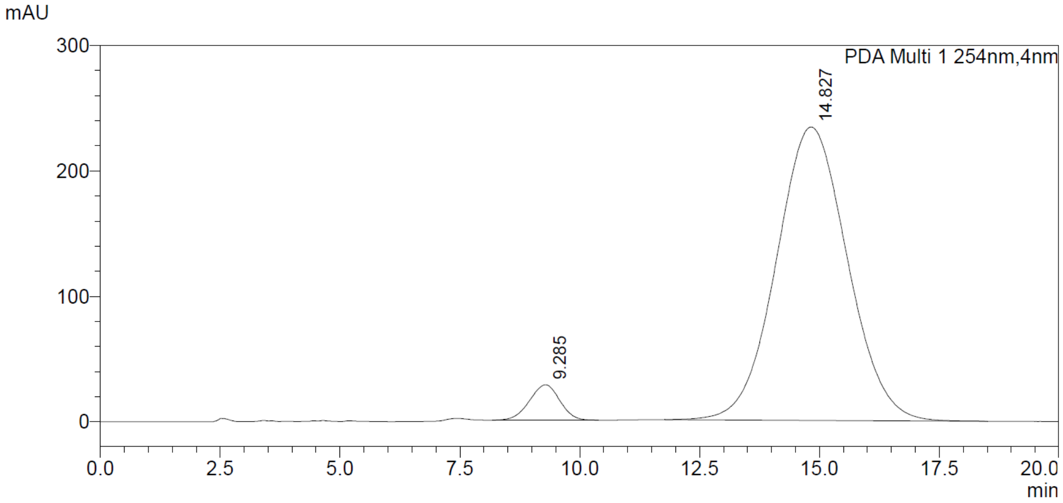

<Peak Table>

PDA Ch1 254nm

Peak#	Ret. Time	Area	Height	Area%
1	9.285	1238443	28168	4.875
2	14.827	24164473	233994	95.125
Total		25402917	262163	100.000

Supplementary Figure 275. HPLC spectrum of **7a**

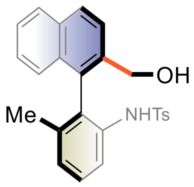

7b

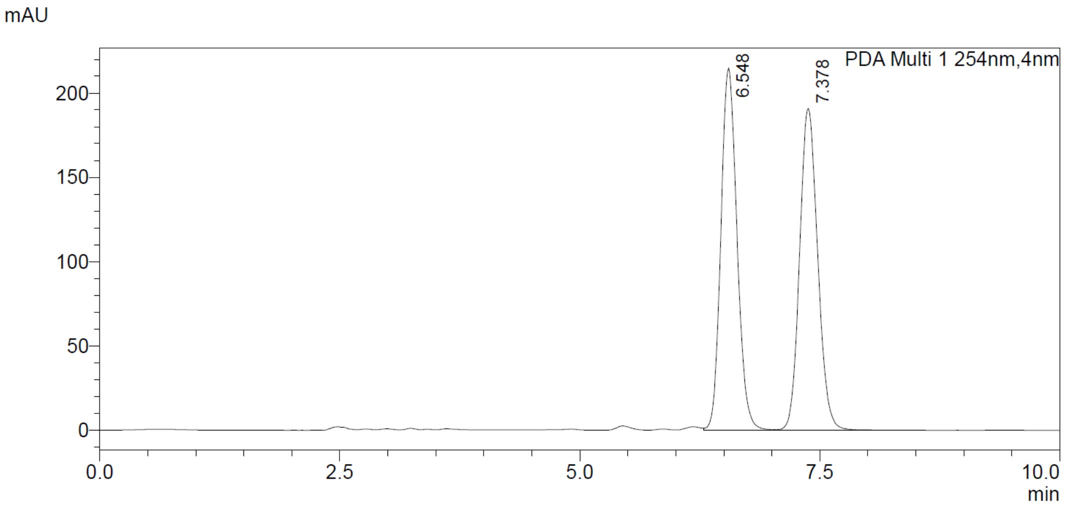

<Peak Table>

PDA Ch1 254nm

Peak#	Ret. Time	Area	Height	Area%
1	6.548	2508397	214744	50.047
2	7.378	2503714	190912	49.953
Total		5012111	405656	100.000

Supplementary Figure 276. HPLC spectrum of racemic **7b**

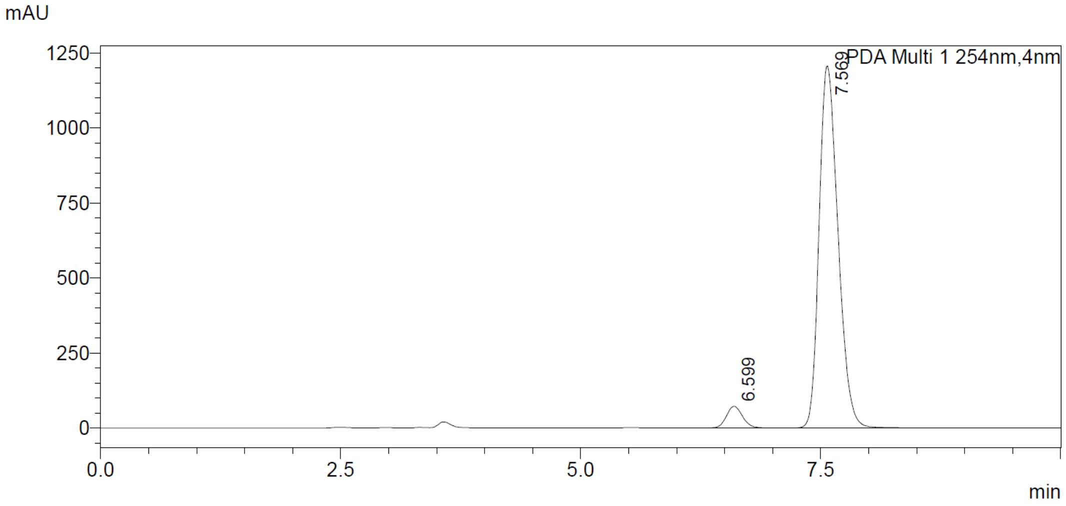

<Peak Table>

PDA Ch1 254nm

Peak#	Ret. Time	Area	Height	Area%
1	6.599	842152	72237	4.843
2	7.569	16546819	1206643	95.157
Total		17388971	1278880	100.000

Supplementary Figure 277. HPLC spectrum of **7b**

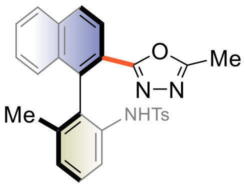

7c

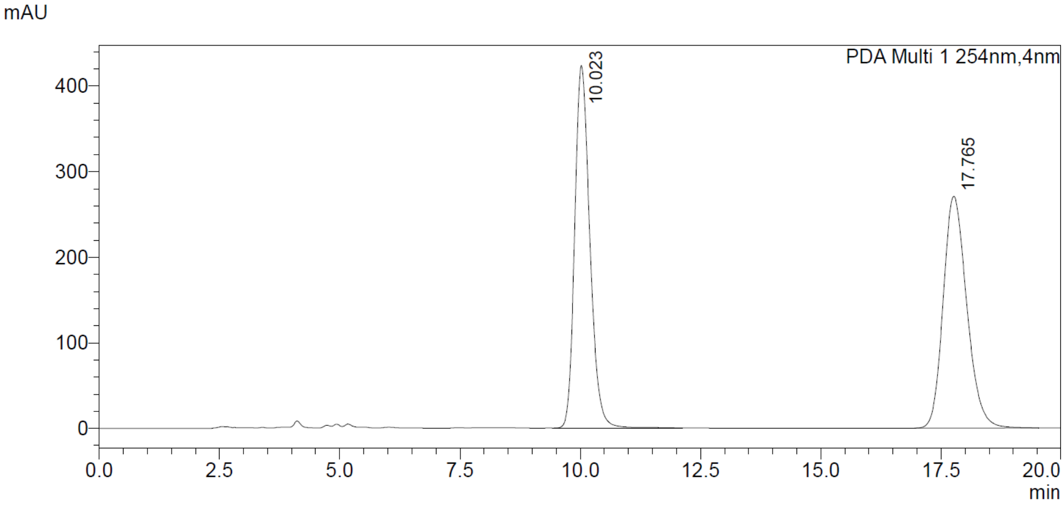

<Peak Table>

PDA Ch1 254nm

Peak#	Ret. Time	Area	Height	Area%
1	10.023	9268735	423550	49.992
2	17.765	9271739	271011	50.008
Total		18540474	694561	100.000

Supplementary Figure 278. HPLC spectrum of racemic **7c**

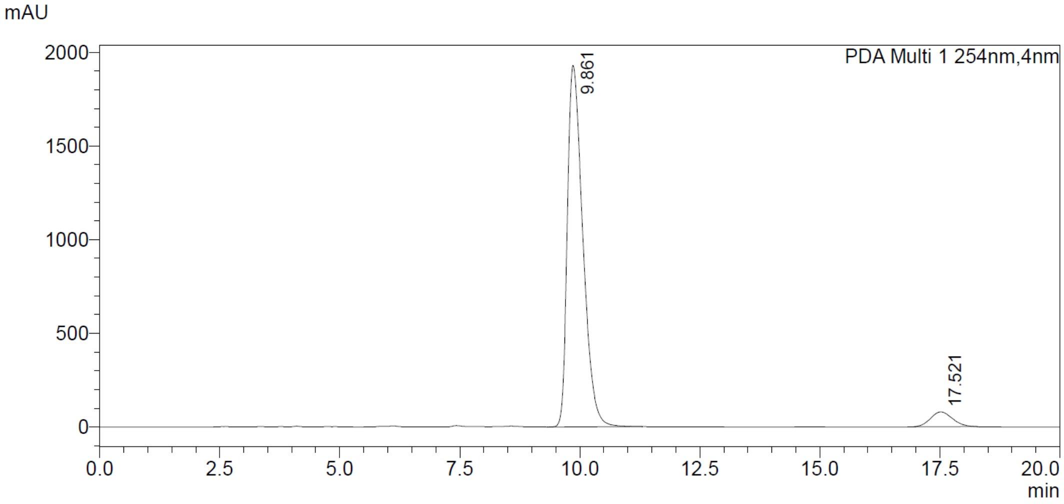

<Peak Table>

PDA Ch1 254nm

Peak#	Ret. Time	Area	Height	Area%
1	9.861	43255528	1928975	94.518
2	17.521	2508875	78946	5.482
Total		45764403	2007921	100.000

Supplementary Figure 279. HPLC spectrum of **7c**

4. Supplementary References

[1] Wang, G.; Shi, Q.; Hu, W.; Chen, T.;Guo, Y.; Hu, Z.; Gong, M.; Guo, J.; Wei, D.; Fu, Z.; Huang, W. Organocatalytic asymmetric N-sulfonyl amide C-N bond activation to access axially chiral biaryl amino acids. *Nat. Commun.* **2020**, *11*, 946.

[2] Gaussian 16 Rev. A.03, Frisch, M. J.,et. al. Gaussian Inc. Wallingford CT, 2016.61.

[3] Bergner, A.; Dolg, M.; Küchle, W.; Stoll, H.; Preuss, H. Mol. Phys. 1993, 80, 1431–1441.

[4] Ditchfield, R.;Hehre, W. J.; Pople, J. A. J. Chem. Phys. 1971, 54, 724–728.

[5] Clark, T.; Chandrasekhar, J.; Spitznagel, G. W.; Schleyer, P.v.R. J. Comput.. Chem. 1983, 4, 294–301.

[6] D. G. Truhlaret. al. Theor. Chem. Acc., 2008, 120, 215-241.

[7] H. Krieget. al. J. Chem. Phys., 2010, 132, 154104.

[8] F. Weigend, et. al. Phys. Chem. Chem. Phys., 2006,8, 1057-1065.

[9] CYLview, 1.0b, Legault, C. Y., Université de Sherbrooke, 2009. （http://www.cylview.org/.)
